# Analysing the impact of the two most common SARS-CoV-2 nucleocapsid protein variants on interactions with membrane protein in silico

**DOI:** 10.1186/s43141-021-00233-z

**Published:** 2021-09-20

**Authors:** Syeda Tasnim Quayum, Saam Hasan

**Affiliations:** Dhaka, Bangladesh

**Keywords:** SARS-CoV-2, Proteomics, N-M protein interactions, N protein

## Abstract

As the body of scientific research focusing on the severe acute respiratory syndrome coronavirus 2 or SARS-CoV-2 continues to grow, several mutations have been reported as very common across the globe. In this study, we analysed the SARS-CoV-2 nucleocapsid protein (N protein) with respect to the widely observed 28881-28883 GGG to AAC variant. One of the major functions of the SARS-CoV-2 nucleocapsid protein is virion packaging through its interactions with the membrane protein (M protein). Our goal was to investigate, using in silico studies, the interaction between the mutant nucleocapsid protein and the M protein and how it differed from that of wild type N-M protein interaction. The results showed significant differences in interactions between the two. The mutant protein was predicted to form 3 salt bridges with the M protein, while the wild type only formed 2. The mutant protein was also predicted to display less temperature sensitivity than its wild type counterpart.

**Supplementary Information:**

The online version contains supplementary material available at 10.1186/s43141-021-00233-z.

## Introduction

The 204 G>R and 203 R>K amino acid substitutions are two of the most commonly observed mutations in the SARS-CoV-2 proteome. Owing to its global distribution, we raise the question whether this is an advantageous mutation that became a stable change over time, or vice versa. To narrow our focus toward a more specific goal, we analysed the effect of these substitutions on the nucleocapsid protein’s interactions with the membrane protein, a process vital for virion packaging.

The severe acute respiratory syndrome coronavirus 2, or SARS-CoV-2, caused COVID-19 pandemic continues to affect the world [11]. As of April 20, 2021, the virus, originating from Wuhan, China, has spread to 227 countries, infecting over 141 million people and leading to over 3 million deaths [30]. Symptom identification and disease management is still proving complicated, with a multitude of ever-evolving factors such as age dependence, making treatment difficult [1, 2, 14, 16]. Meanwhile, a vast volume of research on the virus has accumulated, focussing on the viral genome, proteome, as well as treatment options [5, 9, 22].

A lot of the research has focussed on the presence and distribution of genomic variants. Many of these have been found in isolates from all parts of the world. Genome characterization of SARS-CoV-2 isolates has revealed well over 100 globally distributed mutations [17]. Variants such as the 23403 A to G that causes the 614 D to G mutation in the spike glycoprotein have received a lot of attention [20]. The N gene has also been the subject of a fair amount of scrutiny [15, 25]. The 28881-28883 GGG to AAC variant has also been observed widely in isolates from across the globe [24].

The two variants in focus occur within the intrinsically disordered region (IDR) of the N protein. This region has been shown to be involved in liquid-liquid phase separation (LLPS), a process that plays a part in RNA packaging. Furthermore, the disordered regions are all known to take part in protein-protein interactions [8, 28, 29]. Straightaway, this raises the question whether the amino acid substitutions can lead to functional changes, despite the substituted amino acids also being disorder promoting.

In this study, we wished to explore if this common variant can have a potential impact on the interactions between the N protein and the membrane protein (M protein), something that has not been widely investigated yet. These interactions are vital for RNA packaging and virion assembly; hence, any factor that has a significant influence on them should be investigated to the utmost, as it may be associated with changes in stability of virion structure and consequently its transmissibility and survival rates.

Here, we explored these interactions by predicting the 3D structures of both the mutant (28881-28883 variant containing) and wild type (accession ID: YP_009724393.1) (Wuhan RefSeq) N-proteins using amino acid sequences. Subsequently, we carried out an in silico docking with M protein (accession ID: YP_009724393.1). This was followed by analysis and comparison of 2 N-M complexes. Both the N and M proteins are key structural components of the virus [4, 7]. Hence, we hypothesize that any changes in interactions between them could have a potential impact on viral functions. The purpose of this initial study was to analyse and ascertain these differences resulting from the aforementioned multiple nucleotide variant or MNV, in order to identify any advantageous or disadvantageous nature of said variant. Both of these proteins are predicted to exist as multimers in their innate native states (Masters 2019). This was also taken into account when predicting protein 3 dimensional structure and docking.

## Methodology

Protein structure prediction for the M and both the N proteins were carried out using Robetta server (https://robetta.bakerlab.org/) [18]. The structures were refined using 3Drefine (http://sysbio.rnet.missouri.edu/3Drefine/) [6]. The pdb structures obtained from 3Drefine were further refined using Galaxyrefine tool [13] (http://galaxy.seoklab.org/). Structures with the lowest Molprobity, RMSD values and Clash scores were chosen and then further validated using Ramachandran plot analysis. Protein-protein docking was carried out using Cluspro server (https://cluspro.bu.edu/login.php) [19]. Each of the N proteins (mutant) and (Wuhan reference) were docked separately with the M protein. The docking results were first visualized using PyMol software (https://pymol.org/2/). For confirmation, the protein-protein interactions between the 2 docked complexes were visualized using separate tools so as to ensure all possible interactions were properly identified. In addition to PyMol version 1.2, PDBSum (http://www.ebi.ac.uk/thorntonsrv/databases/cgibin/pdbsum) [21], CoCoMaps (https://www.molnac.unisa.it/BioTools/cocomaps/) [27] and Disovery studio visualizer (https://discover.3ds.com/discovery-studio-visualizer-download) were used. Temperature sensitivity of both the mutant and wild type protein complexes were also checked using the Prodigy tool [31].

## Results

**Fig. 1:**
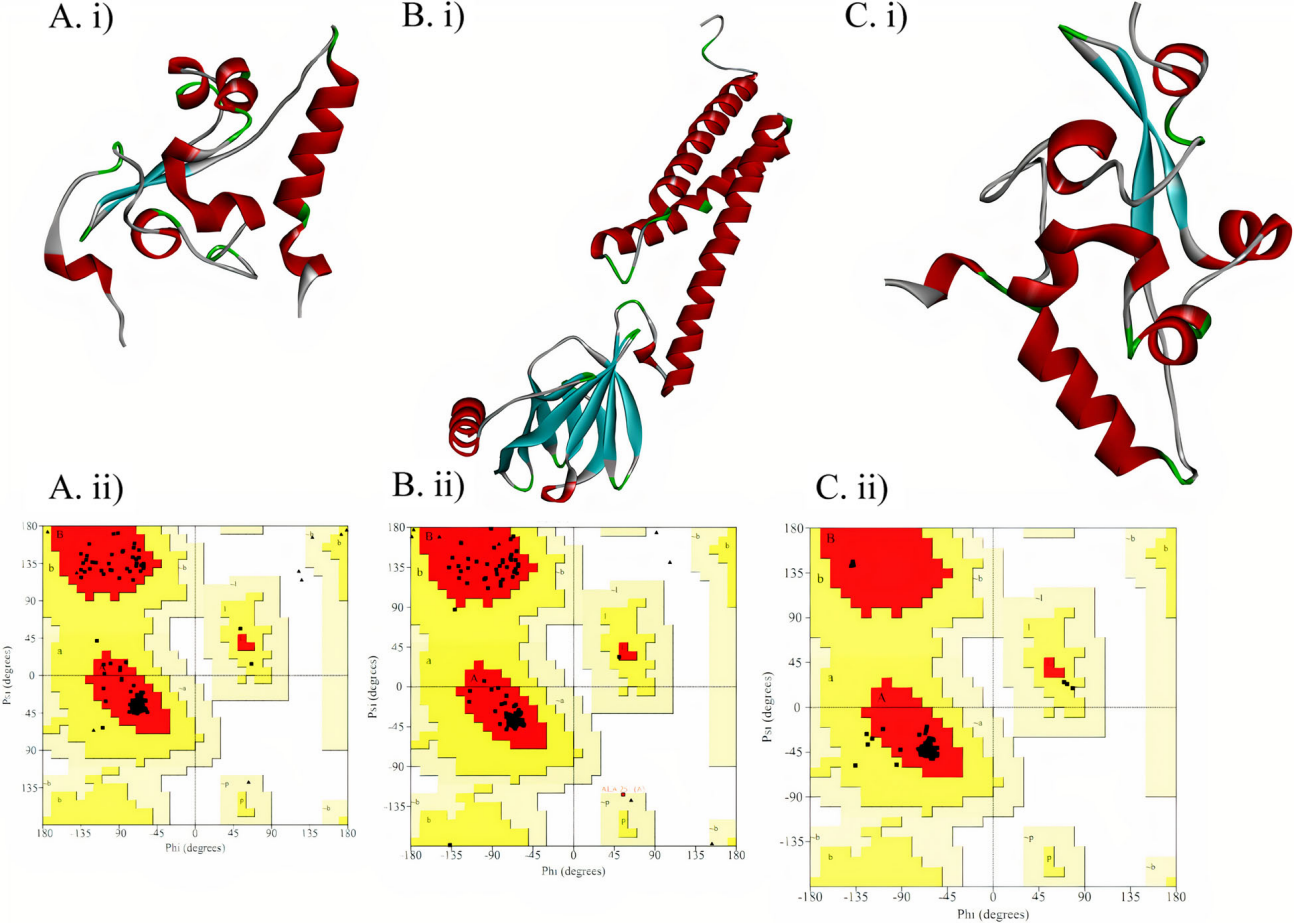
**A. i**) Mutant isolate N-protein 3D predicted structure. **A. ii)** Ramachandran plot analysis of predicted 3D Mutant isolate N-protein. **B. i)** Wuhan wild type N-protein 3D predicted structure. **B. ii)** Ramachandran plot of Wuhan N-protein**. C. i)** M-protein 3D predicted structure. **C. ii)** Ramachandran plot of M-protein

**Fig. 2:**
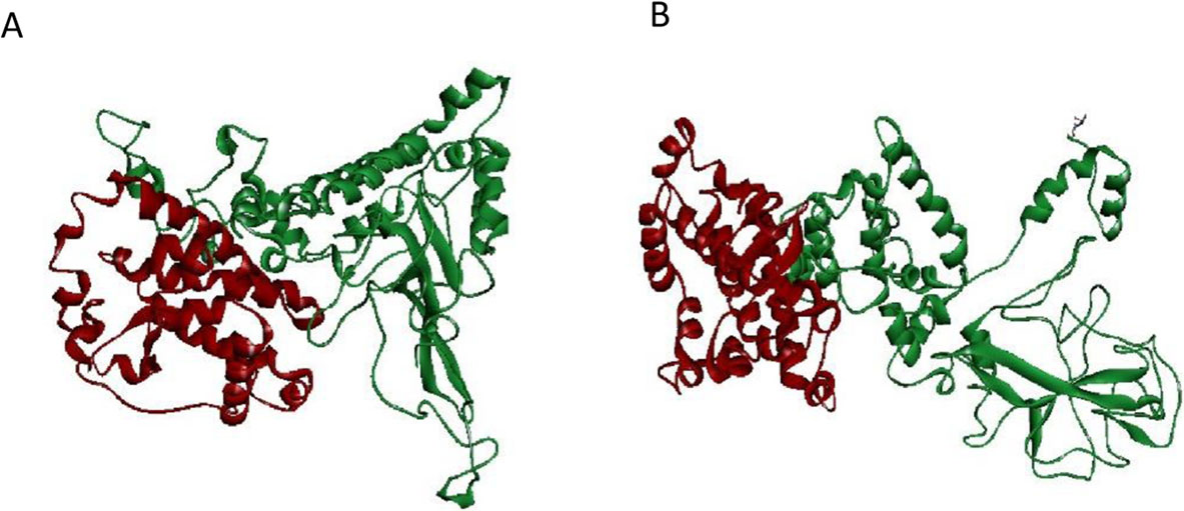
**A** Bangladeshi isolate N & M-protein docking complex. **B** Wuhan wild type N &M-protein docking complex

The structures of M and both N-proteins were predicted, refined and validated. The final predicted structure of M protein has a Molprobity score of 1.736, clash score of 11.7 and RMSD value of 0.290. Validity of the structures analysed by Ramachandran plot shows 94% residues residing in most favoured regions, 1% in disallowed and 4% in additional acceptable regions. The mutant N-protein has a MolProbity score of 1.660 RMSD value of 0.264 and Clash score of 10.4. Ramachandran plot analysis carried out on the Mutant isolate N-protein showed 92.8% residues residing in highly favoured regions, 0.6% in moderately allowed and 0.3% in disfavoured regions. Wild type N-protein has a MolProbity score of 1.451 RMSD value of 0.236 and Clash score of 8.3. Ramachandran plot analysis carried out on the wild type N protein showed 91.6% residues residing in highly favoured regions, 0.0% in moderately allowed and 0.6% in disfavoured regions (Fig. 1). After the docking was carried out between the M and each of the N-proteins, the structures with lowest energy levels and most number of interacting members were chosen. Energy level of Fig. 2A was − 1059.1 KJ with 128 members acting and of Fig. 2B docked complex was − 1221.0 KJ with 57 members. Supplementary files 1 and 2 provide the final PDB structures generated for the mutant-M protein and wild type-M protein complexes respectively.

Table 1 and Table 2 list all contact residues for each of the N-M docked complexes. Overall, there were 25 amino acid residues from the mutant N-protein that interact with the M, while for the wild type, there are 27 residues. The number of hydrophobic interactions between residues was also higher for the wild type (139), compared to the mutant (104). The binding affinity and dissociation constant (K_D_) were both lower for the mutant protein. There were also differences in the key residue interactions. For the wild type, there were two salt bridges predicted between the M and N proteins (LYS162-ASP341 and LYS162-ASP348). The number of salt bridges observed for the mutant protein was 3, and they involved different residues (ARG42-ASP288, ARG146-GLU323, ARG150-GLU323). The number of hydrogen bonds was also higher for the mutant at 15. The wild type shared 14 hydrogen bonds with the M protein. Lastly, the number of non-bonded contacts (Van der Waals forces) was also higher for the mutant (162) than the wild type (134). Other important residues include the Serine at position 318, which was shown to be an interacting residue for both the complexes. The list of major interacting residues is shown in Tables 1 and 2. For size constraints, only the hydrogen bonds and salt bridges are included here. The complete lists can be found in Supplementary Tables 1A and 1B.

**Table 1:** Residues involved in hydrogen bonds and salt bridges between the mutant N protein and the M protein

Bond type	M protein atom	M protein residue	M protein residue position	N protein atom	N protein residue	N protein residue position	Distance
Hydrogen bond	N	ALA	2	OE2	GLU	323	3.04
Hydrogen bond	ND2	ASN	41	O	ARG	319	3.01
Hydrogen bond	NE	ARG	42	O	GLN	283	2.9
Hydrogen bond	NH1	ARG	42	O	ILE	320	2.71
Hydrogen bond	NH2	ARG	42	O	PHE	286	2.81
Hydrogen bond	NH2	ARG	42	O	GLY	287	2.66
Hydrogen bond	NH2	ARG	42	OD1	ASP	288	2.91
Hydrogen bond	NH2	ARG	44	OG	SER	312	2.77
Hydrogen bond	N	ILE	144	O	GLY	284	2.8
Hydrogen bond	N	ARG	146	OD1	ASP	288	3.19
Hydrogen bond	NH1	ARG	146	OE1	GLU	323	2.76
Hydrogen bond	N	GLY	147	OE1	GLU	290	2.84
Hydrogen bond	NH1	ARG	150	OE2	GLU	323	2.79
Hydrogen bond	NH2	ARG	150	OE1	GLU	323	2.73
Salt bridge	NH1	ARG	42	OD1	ASP	288	2.75
Salt bridge	NH1	ARG	146	OE2	GLU	323	2.76
Salt bridge	NH2	ARG	150	OE2	GLU	323	2.73

**Table 2:** Residues involved in hydrogen bonds and salt bridges between the wild type N protein and the M protein

Bond type	M protein atom	M protein residue	M protein residue position	N protein atom	N protein residue	N protein residue position	Distance
Hydrogen bond	OD2	ASP	3	OG	SER	318	2.83
Hydrogen bond	ND2	ASN	41	O	SER	310	2.81
Hydrogen bond	ND2	ASN	41	O	ALA	311	2.88
Hydrogen bond	NE	ARG	42	O	PHE	315	2.83
Hydrogen bond	NH2	ARG	42	O	PHE	315	2.69
Hydrogen bond	NH2	ARG	42	O	GLY	316	2.67
Hydrogen bond	NE	ARG	44	OE1	GLN	306	3.17
Hydrogen bond	NH2	ARG	44	OE1	GLN	306	2.69
Hydrogen bond	NH2	ARG	44	OG	SER	312	2.77
Hydrogen bond	NE	ARG	101	OE1	GLN	390	2.8
Hydrogen bond	NH1	ARG	101	OE1	GLN	386	2.67
Hydrogen bond	NH1	ARG	101	OE1	GLN	389	2.77
Hydrogen bond	NH2	ARG	101	OE1	GLN	389	2.75
Hydrogen bond	NH2	ARG	101	OE1	GLN	390	2.8
Hydrogen bond	NH1	ARG	158	O	LEU	339	2.67
Salt bridge	NZ	LYS	162	OD1	ASP	341	2.69
Salt bridge	NZ	LYS	162	OD2	ASP	348	2.56

**Table 3:** The affinity for the mutant and wild type N proteins for the M protein at 25 and 35 °C. As it can be seen, the mutant appears to display more temperature sensitivity than the wild type, with its affinity for the M protein decreasing over this range

Protein	Temperature (°C)	Affinity for M protein (M)
Mutant isolate N protein	25	5.30E−11
	35	1.10E−10
Wuhan virus N protein	25	1.30E−09
	35	2.50E−09

Another interesting observation here was the fact that increasing the temperature value affected the two complexes differently. For the mutant protein, a simulation that increased temperature from 25 to 35 °C resulted in a weaker affinity with the M protein. For the wild type, however, there was no discernible difference. This could suggest that the mutant protein is more sensitive to higher temperatures than the wild type. Table 3 summarizes the affinities for the two complexes at different temperatures.

**Fig. 3:**
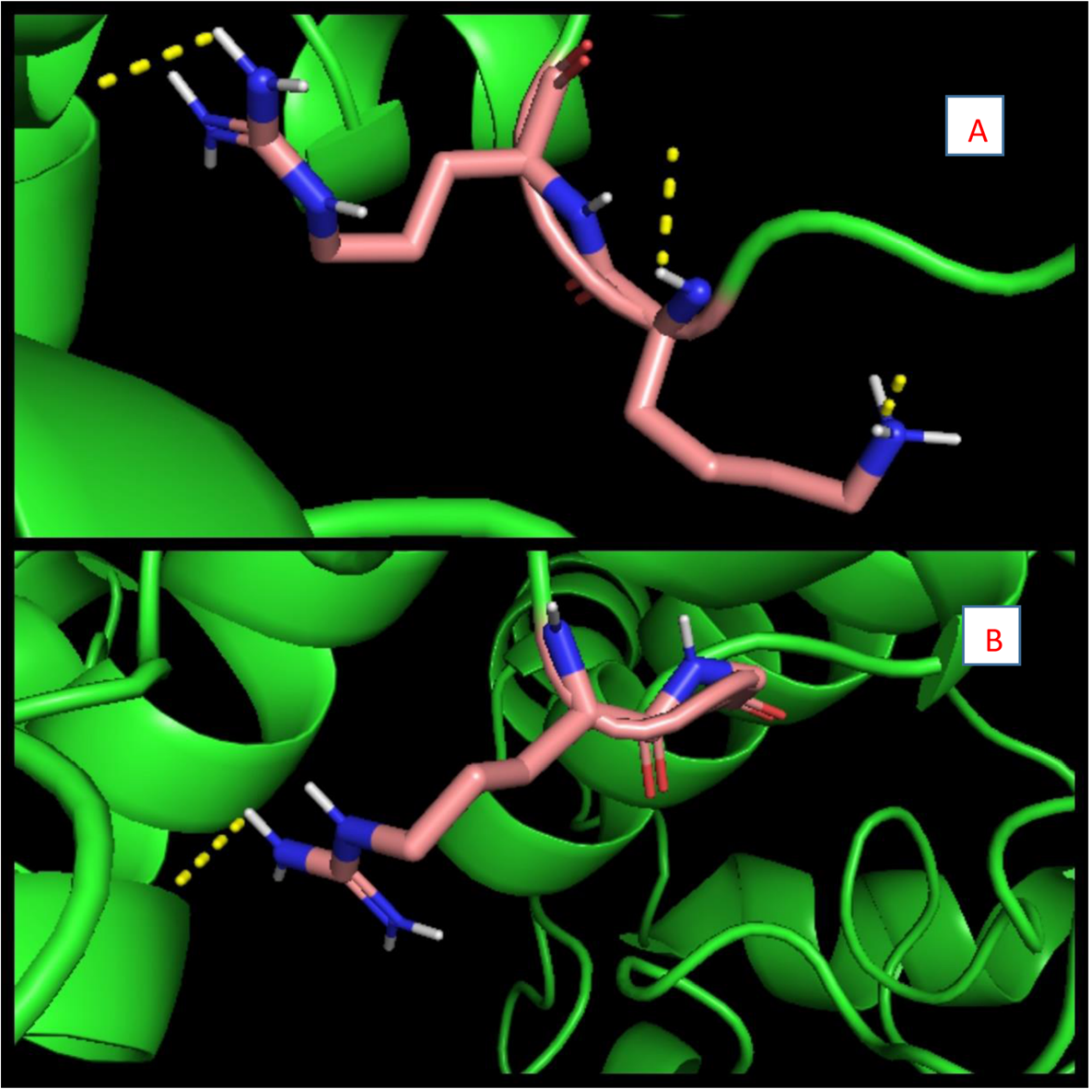
**A** Determination of Hydrogen bonds over 2.5 Å for the mutated residues, using Pymol. Mutated residues shown as sticks (atoms: nitrogen-blue, carbon-pink, hydrogen-white), N protein in green and hydrogen bonding as yellow dots. **B** Determination of hydrogen bonds over a 2.5 Å range for wild type residues. Original wild type residues shown as sticks (atoms: nitrogen-blue, carbon-pink, hydrogen-white, oxygen-red), N protein in green and hydrogen bonding as yellow dots

In order to observe whether the mutated residues formed any strong hydrogen bonds, pymol was used to determine whether the altered amino acid residues (K replacing an R and an R replacing a G) formed polar contacts. A cutoff value 2.5 was used, with all possible atomic contacts occurring within this range being taken into consideration (Fig. 3A). Figure 2A shows that the mutated residues are capable of forming at least 3 minimum polar interactions. To determine whether the mutated residues formed stronger contacts than the wild type ones (R, G), the same step was repeated for them. Only 1 hydrogen bond (Fig. 3B) was observed at this cutoff value, indicating the mutated residues displayed stronger polar interaction with the M protein than the wild type residues.

## Discussion

Our primary research question was whether the two N protein variants in focus alter the protein’s interactions with the M protein. To that end, we highlighted two major observations that can hold potential significance. The first is the supposed temperature sensitivity of the mutant N protein. This would seem to indicate that the mutations may be of a disadvantageous nature. An N protein that is adversely affected by increased temperatures may be more vulnerable to having its bonds with the M proteins disrupted by increased heat. The end result could be a loss of viral transmission and survival chances in hotter climates. This is especially important given that this variant is found across the globe, including warmer regions [26]. Secondly, the gain of a salt bridge can potentially strengthen the N-M protein interactions for the mutant. Given the importance of this interaction to virion structure, this could theoretically further stabilize the nucleocapsid. In addition, the salt bridges for the wild type involved only one residue on the M protein (the lysine at position 162). In contrast, the mutant protein interacts with three separate residues on the M protein through salt bridges. These are ARG42, ARG146 and ARG150. The strengthened interaction theory is further backed up by the fact that the mutant had more potential hydrogen bonds and more potential Van der Waal’s interactions with the M protein. An important caveat here is that in all of these cases, the atomic distances between the interacting atoms was over 2.5 Å. The average length of a hydrogen bond is taken to be 2–2.5 Å. This would seem to call the existence of these hydrogen bonds into question somewhat. However, this does give even more importance to the higher number of salt bridges for the mutant protein, as they may potentially be creating a more stable complex with the M protein.

Other studies have also highlighted this variant and the two resultant amino acid substitutions as potentially destabilizing the protein structure [3]. These have suggested viruses containing these variants may be less adept at transmission, owing to the conserved nature of this region of the protein [12, 23, 28, 29]. This agrees with our temperature sensitivity analysis, although the additional salt bridges we predicted would seem to contradict this.

How these additional interactions and the resulting stronger nucleocapsid structure could impact virion transmission and pathogenesis is an area that should be targeted by future research. These findings do make for difficult interpretation in terms of the original question we posed. The potential for the 28881-28883 variant to enhance pathogenic potential of the virus is still very much a possibility. Moreover, this MNV is now distributed globally, suggesting this may have been an advantageous variant that became stably integrated into the genome over time.

Regardless of how severe the symptoms of infection are, a more stable virus will be able to survive for longer and infect more people. The real effect of the changes in salt bridge bonds is of course something that needs to be confirmed and determined by experimental evidence. One major factor behind throwing that into doubt is the fact that while the number of bridges was more for the mutant protein, the participating atoms were further apart compared to the two salt bridges for the wild type.

As the battle against SARS-CoV-2 continues to complicate, it becomes important for the research community to once more pay close attention to the fundamental aspects of the virus. The more practical consequences of this variant, such as vaccine effectiveness, remains difficult to ascertain. The ones that do not rely on detection of N protein components, such as the BNT162b1 or ChAdOx1 nCoV-19 vaccines (both Spike protein reliant), should not theoretically be affected. However, if future vaccines choose to use the N protein for eliciting an immune reaction, then this could change. The nature of the impact demands future investigation [10].

## Conclusions

We believe there is evidence to suggest that the SARS-CoV-2 isolates harbouring the 28881-28883 mutation have the potential to increase stability of the viral nucleocapsid. This interpretation is derived from the gain of certain amino acid interactions that may strengthen its complex with the viral M protein, potentially leading to a more stable virus structure. Though causality cannot yet be established, this does nonetheless deserve further investigation with regards to its potential impact on viral survival and transmission.

## Supplementary informations

Additional file 1: **Supplementary Table 1A.** – Complete list of closely situated residues from mutant N protein and M protein in the docked complex.

Additional file 2: **Supplementary Table 1B.** – Complete list of closely situated residues from wild type N protein and M protein in the docked complex.

Additional file 3: **Supplementary File 1.** – Docked complex between mutant N protein and M protein.

Additional file 4: **Supplementary File 2.** – Docked complex between wild type N protein and M protein.

## Data Availability

No new genomic or proteomic sequences were generated through this study. The in silico predicted structures are made available in the supplementary material.

## Abbreviations

MNV: Multiple nucleotide variant
SARS-CoV-2: Severe acute respiratory syndrome coronavirus 2
N protein: Nucleocapsid protein
M protein: Membrane protein
LLPS: Liquid-liquid phase separation
