# Supplementary material for: Analysing the impact of the two most common SARS-CoV-2 nucleocapsid protein variants on interactions with membrane protein in silico
Source: J Genet Eng Biotechnol. 2021 Sep 20;19:138. doi: 10.1186/s43141-021-00233-z (PMC8451389; doi:10.1186/s43141-021-00233-z)
Supplement: Supplementary file 3 — Supplementary File 1. – Docked complex between mutant N protein and M protein. [file 43141_2021_233_MOESM3_ESM.pdf]

```
ATOM      1  N   MET A   1      -0.117 -11.301  -6.333  1.00  0.00           N
ATOM      2  H   MET A   1       0.274 -11.528  -7.224  1.00  0.00           H
ATOM      3  CA  MET A   1      -1.407 -10.594  -6.393  1.00  0.00           C
ATOM      4  CB  MET A   1      -1.224  -9.088  -6.600  1.00  0.00           C
ATOM      5  CG  MET A   1      -2.497  -8.291  -6.285  1.00  0.00           C
ATOM      6  SD  MET A   1      -3.065  -8.538  -4.594  1.00  0.00           S
ATOM      7  CE  MET A   1      -4.601  -7.606  -4.689  1.00  0.00           C
ATOM      8  C   MET A   1      -2.223 -11.237  -7.469  1.00  0.00           C
ATOM      9  O   MET A   1      -2.463 -10.673  -8.536  1.00  0.00           O
ATOM     10  N   ALA A   2      -2.698 -12.461  -7.153  1.00  0.00           N
ATOM     11  H   ALA A   2      -2.580 -12.724  -6.191  1.00  0.00           H
ATOM     12  CA  ALA A   2      -3.482 -13.308  -8.017  1.00  0.00           C
ATOM     13  CB  ALA A   2      -3.713 -14.673  -7.366  1.00  0.00           C
ATOM     14  C   ALA A   2      -4.807 -12.712  -8.387  1.00  0.00           C
ATOM     15  O   ALA A   2      -5.259 -12.894  -9.519  1.00  0.00           O
ATOM     16  N   ASP A   3      -5.415 -11.939  -7.452  1.00  0.00           N
ATOM     17  H   ASP A   3      -4.996 -11.913  -6.543  1.00  0.00           H
ATOM     18  CA  ASP A   3      -6.703 -11.312  -7.622  1.00  0.00           C
ATOM     19  CB  ASP A   3      -7.098 -10.501  -6.384  1.00  0.00           C
ATOM     20  CG  ASP A   3      -8.147 -11.214  -5.554  1.00  0.00           C
ATOM     21  OD1 ASP A   3      -8.768 -12.166  -6.013  1.00  0.00           O
ATOM     22  OD2 ASP A   3      -8.317 -10.872  -4.392  1.00  0.00           O
ATOM     23  C   ASP A   3      -6.757 -10.389  -8.797  1.00  0.00           C
ATOM     24  O   ASP A   3      -7.773 -10.370  -9.495  1.00  0.00           O
ATOM     25  N   SER A   4      -5.684  -9.602  -9.030  1.00  0.00           N
ATOM     26  H   SER A   4      -4.903  -9.591  -8.405  1.00  0.00           H
ATOM     27  CA  SER A   4      -5.640  -8.708 -10.147  1.00  0.00           C
ATOM     28  CB  SER A   4      -5.120  -7.452  -9.498  1.00  0.00           C
ATOM     29  OG  SER A   4      -5.298  -7.700  -8.096  1.00  0.00           O
ATOM     30  HG  SER A   4      -6.237  -7.707  -7.946  1.00  0.00           H
ATOM     31  C   SER A   4      -4.837  -9.246 -11.304  1.00  0.00           C
ATOM     32  O   SER A   4      -5.139  -8.920 -12.451  1.00  0.00           O
ATOM     33  N   ASN A   5      -3.844 -10.140 -11.045  1.00  0.00           N
ATOM     34  H   ASN A   5      -3.660 -10.373 -10.088  1.00  0.00           H
ATOM     35  CA  ASN A   5      -2.994 -10.728 -12.065  1.00  0.00           C
ATOM     36  CB  ASN A   5      -1.903 -11.619 -11.488  1.00  0.00           C
ATOM     37  CG  ASN A   5      -1.281 -12.401 -12.627  1.00  0.00           C
ATOM     38  OD1 ASN A   5      -0.527 -11.871 -13.437  1.00  0.00           O
ATOM     39  ND2 ASN A   5      -1.601 -13.711 -12.622  1.00  0.00           N
ATOM     40 HD21 ASN A   5      -1.221 -14.344 -13.297  1.00  0.00           H
ATOM     41 HD22 ASN A   5      -2.222 -14.095 -11.937  1.00  0.00           H
ATOM     42  C   ASN A   5      -3.829 -11.582 -12.977  1.00  0.00           C
ATOM     43  O   ASN A   5      -3.618 -11.567 -14.190  1.00  0.00           O
ATOM     44  N   GLY A   6      -4.857 -12.263 -12.413  1.00  0.00           N
ATOM     45  H   GLY A   6      -5.030 -12.181 -11.428  1.00  0.00           H
ATOM     46  CA  GLY A   6      -5.753 -13.128 -13.134  1.00  0.00           C
ATOM     47  C   GLY A   6      -6.555 -12.397 -14.171  1.00  0.00           C
ATOM     48  O   GLY A   6      -7.066 -13.038 -15.087  1.00  0.00           O
ATOM     49  N   THR A   7      -6.726 -11.059 -14.020  1.00  0.00           N
ATOM     50  H   THR A   7      -6.367 -10.581 -13.220  1.00  0.00           H
ATOM     51  CA  THR A   7      -7.456 -10.259 -14.964  1.00  0.00           C
ATOM     52  CB  THR A   7      -8.212  -9.369 -14.013  1.00  0.00           C
ATOM     53  OG1 THR A   7      -8.057  -9.990 -12.724  1.00  0.00           O
ATOM     54  HG1 THR A   7      -8.655  -9.577 -12.110  1.00  0.00           H
ATOM     55  CG2 THR A   7      -9.684  -9.200 -14.388  1.00  0.00           C
ATOM     56  C   THR A   7      -6.527  -9.554 -15.922  1.00  0.00           C
ATOM     57  O   THR A   7      -6.824  -9.481 -17.113  1.00  0.00           O
ATOM     58  N   ILE A   8      -5.353  -9.076 -15.431  1.00  0.00           N
ATOM     59  H   ILE A   8      -5.179  -9.220 -14.454  1.00  0.00           H
```

```
ATOM     60  CA  ILE A   8      -4.370  -8.338 -16.206  1.00  0.00           C
ATOM     61  CB  ILE A   8      -3.310  -7.736 -15.288  1.00  0.00           C
ATOM     62  CG2 ILE A   8      -2.147  -7.111 -16.059  1.00  0.00           C
ATOM     63  CG1 ILE A   8      -3.996  -6.700 -14.400  1.00  0.00           C
ATOM     64  CD1 ILE A   8      -3.060  -6.095 -13.363  1.00  0.00           C
ATOM     65  C   ILE A   8      -3.786  -9.187 -17.318  1.00  0.00           C
ATOM     66  O   ILE A   8      -3.473  -8.673 -18.394  1.00  0.00           O
ATOM     67  N   THR A   9      -3.647 -10.507 -17.071  1.00  0.00           N
ATOM     68  H   THR A   9      -3.957 -10.895 -16.199  1.00  0.00           H
ATOM     69  CA  THR A   9      -3.158 -11.474 -18.022  1.00  0.00           C
ATOM     70  CB  THR A   9      -2.851 -12.737 -17.242  1.00  0.00           C
ATOM     71  OG1 THR A   9      -3.914 -12.954 -16.318  1.00  0.00           O
ATOM     72  HG1 THR A   9      -3.549 -12.830 -15.448  1.00  0.00           H
ATOM     73  CG2 THR A   9      -1.518 -12.635 -16.500  1.00  0.00           C
ATOM     74  C   THR A   9      -4.146 -11.718 -19.148  1.00  0.00           C
ATOM     75  O   THR A   9      -3.734 -12.058 -20.258  1.00  0.00           O
ATOM     76  N   VAL A  10      -5.467 -11.578 -18.862  1.00  0.00           N
ATOM     77  H   VAL A  10      -5.717 -11.192 -17.973  1.00  0.00           H
ATOM     78  CA  VAL A  10      -6.565 -11.795 -19.783  1.00  0.00           C
ATOM     79  CB  VAL A  10      -7.873 -11.950 -18.997  1.00  0.00           C
ATOM     80  CG1 VAL A  10      -9.123 -12.034 -19.873  1.00  0.00           C
ATOM     81  CG2 VAL A  10      -7.763 -13.185 -18.108  1.00  0.00           C
ATOM     82  C   VAL A  10      -6.661 -10.733 -20.858  1.00  0.00           C
ATOM     83  O   VAL A  10      -6.543  -9.532 -20.605  1.00  0.00           O
ATOM     84  N   GLU A  11      -6.941 -11.209 -22.098  1.00  0.00           N
ATOM     85  H   GLU A  11      -7.126 -12.188 -22.173  1.00  0.00           H
ATOM     86  CA  GLU A  11      -7.063 -10.442 -23.315  1.00  0.00           C
ATOM     87  CB  GLU A  11      -7.165 -11.432 -24.484  1.00  0.00           C
ATOM     88  CG  GLU A  11      -7.105 -10.823 -25.886  1.00  0.00           C
ATOM     89  CD  GLU A  11      -8.443 -10.198 -26.181  1.00  0.00           C
ATOM     90  OE1 GLU A  11      -9.439 -10.729 -25.711  1.00  0.00           O
ATOM     91  OE2 GLU A  11      -8.511  -9.157 -26.823  1.00  0.00           O
ATOM     92  C   GLU A  11      -8.162  -9.411 -23.232  1.00  0.00           C
ATOM     93  O   GLU A  11      -7.994  -8.299 -23.739  1.00  0.00           O
ATOM     94  N   GLU A  12      -9.286  -9.750 -22.554  1.00  0.00           N
ATOM     95  H   GLU A  12      -9.405 -10.712 -22.325  1.00  0.00           H
ATOM     96  CA  GLU A  12     -10.417  -8.870 -22.395  1.00  0.00           C
ATOM     97  CB  GLU A  12     -11.590  -9.541 -21.682  1.00  0.00           C
ATOM     98  CG  GLU A  12     -12.331 -10.536 -22.575  1.00  0.00           C
ATOM     99  CD  GLU A  12     -12.687  -9.859 -23.887  1.00  0.00           C
ATOM    100  OE1 GLU A  12     -12.913  -8.654 -23.911  1.00  0.00           O
ATOM    101  OE2 GLU A  12     -12.669 -10.502 -24.930  1.00  0.00           O
ATOM    102  C   GLU A  12     -10.029  -7.642 -21.617  1.00  0.00           C
ATOM    103  O   GLU A  12     -10.451  -6.546 -21.987  1.00  0.00           O
ATOM    104  N   LEU A  13      -9.208  -7.790 -20.541  1.00  0.00           N
ATOM    105  H   LEU A  13      -8.793  -8.680 -20.356  1.00  0.00           H
ATOM    106  CA  LEU A  13      -8.783  -6.646 -19.776  1.00  0.00           C
ATOM    107  CB  LEU A  13      -8.306  -7.007 -18.372  1.00  0.00           C
ATOM    108  CG  LEU A  13      -8.096  -5.753 -17.515  1.00  0.00           C
ATOM    109  CD1 LEU A  13      -9.384  -4.940 -17.374  1.00  0.00           C
ATOM    110  CD2 LEU A  13      -7.482  -6.067 -16.155  1.00  0.00           C
ATOM    111  C   LEU A  13      -7.726  -5.903 -20.545  1.00  0.00           C
ATOM    112  O   LEU A  13      -7.704  -4.680 -20.471  1.00  0.00           O
ATOM    113  N   LYS A  14      -6.876  -6.607 -21.344  1.00  0.00           N
ATOM    114  H   LYS A  14      -6.962  -7.604 -21.352  1.00  0.00           H
ATOM    115  CA  LYS A  14      -5.818  -6.001 -22.132  1.00  0.00           C
ATOM    116  CB  LYS A  14      -5.013  -7.066 -22.865  1.00  0.00           C
ATOM    117  CG  LYS A  14      -4.020  -7.747 -21.931  1.00  0.00           C
ATOM    118  CD  LYS A  14      -3.460  -9.030 -22.534  1.00  0.00           C
```

```
ATOM    119  CE  LYS A  14      -2.340  -9.609 -21.678  1.00  0.00           C
ATOM    120  NZ  LYS A  14      -2.142 -11.020 -22.033  1.00  0.00           N
ATOM    121  HZ1 LYS A  14      -2.949 -11.563 -21.652  1.00  0.00           H
ATOM    122  HZ2 LYS A  14      -2.096 -11.136 -23.064  1.00  0.00           H
ATOM    123  HZ3 LYS A  14      -1.275 -11.374 -21.587  1.00  0.00           H
ATOM    124  C   LYS A  14      -6.343  -4.969 -23.092  1.00  0.00           C
ATOM    125  O   LYS A  14      -5.670  -3.966 -23.335  1.00  0.00           O
ATOM    126  N   LYS A  15      -7.584  -5.168 -23.605  1.00  0.00           N
ATOM    127  H   LYS A  15      -8.020  -6.051 -23.415  1.00  0.00           H
ATOM    128  CA  LYS A  15      -8.242  -4.258 -24.513  1.00  0.00           C
ATOM    129  CB  LYS A  15      -9.638  -4.764 -24.876  1.00  0.00           C
ATOM    130  CG  LYS A  15      -9.721  -6.086 -25.639  1.00  0.00           C
ATOM    131  CD  LYS A  15     -11.165  -6.341 -26.077  1.00  0.00           C
ATOM    132  CE  LYS A  15     -11.377  -7.648 -26.843  1.00  0.00           C
ATOM    133  NZ  LYS A  15     -11.086  -8.762 -25.943  1.00  0.00           N
ATOM    134  HZ1 LYS A  15     -11.589  -9.646 -26.161  1.00  0.00           H
ATOM    135  HZ2 LYS A  15     -10.077  -9.030 -25.917  1.00  0.00           H
ATOM    136  HZ3 LYS A  15     -11.440  -8.576 -24.984  1.00  0.00           H
ATOM    137  C   LYS A  15      -8.415  -2.916 -23.836  1.00  0.00           C
ATOM    138  O   LYS A  15      -8.279  -1.878 -24.484  1.00  0.00           O
ATOM    139  N   LEU A  16      -8.738  -2.934 -22.518  1.00  0.00           N
ATOM    140  H   LEU A  16      -8.760  -3.823 -22.059  1.00  0.00           H
ATOM    141  CA  LEU A  16      -8.919  -1.772 -21.686  1.00  0.00           C
ATOM    142  CB  LEU A  16      -9.856  -2.107 -20.530  1.00  0.00           C
ATOM    143  CG  LEU A  16     -11.215  -2.600 -21.026  1.00  0.00           C
ATOM    144  CD1 LEU A  16     -12.072  -3.150 -19.886  1.00  0.00           C
ATOM    145  CD2 LEU A  16     -11.944  -1.524 -21.832  1.00  0.00           C
ATOM    146  C   LEU A  16      -7.602  -1.247 -21.177  1.00  0.00           C
ATOM    147  O   LEU A  16      -7.427  -0.035 -21.078  1.00  0.00           O
ATOM    148  N   LEU A  17      -6.634  -2.157 -20.890  1.00  0.00           N
ATOM    149  H   LEU A  17      -6.869  -3.113 -21.052  1.00  0.00           H
ATOM    150  CA  LEU A  17      -5.305  -1.887 -20.384  1.00  0.00           C
ATOM    151  CB  LEU A  17      -4.571  -3.209 -20.139  1.00  0.00           C
ATOM    152  CG  LEU A  17      -3.066  -3.111 -19.869  1.00  0.00           C
ATOM    153  CD1 LEU A  17      -2.732  -2.274 -18.634  1.00  0.00           C
ATOM    154  CD2 LEU A  17      -2.427  -4.499 -19.798  1.00  0.00           C
ATOM    155  C   LEU A  17      -4.548  -1.002 -21.321  1.00  0.00           C
ATOM    156  O   LEU A  17      -3.811  -0.134 -20.860  1.00  0.00           O
ATOM    157  N   GLU A  18      -4.744  -1.172 -22.647  1.00  0.00           N
ATOM    158  H   GLU A  18      -5.276  -1.963 -22.957  1.00  0.00           H
ATOM    159  CA  GLU A  18      -4.083  -0.371 -23.645  1.00  0.00           C
ATOM    160  CB  GLU A  18      -4.394  -0.878 -25.054  1.00  0.00           C
ATOM    161  CG  GLU A  18      -3.888  -2.298 -25.314  1.00  0.00           C
ATOM    162  CD  GLU A  18      -2.384  -2.355 -25.162  1.00  0.00           C
ATOM    163  OE1 GLU A  18      -1.689  -1.547 -25.770  1.00  0.00           O
ATOM    164  OE2 GLU A  18      -1.878  -3.228 -24.460  1.00  0.00           O
ATOM    165  C   GLU A  18      -4.478   1.082 -23.509  1.00  0.00           C
ATOM    166  O   GLU A  18      -3.668   1.962 -23.795  1.00  0.00           O
ATOM    167  N   GLN A  19      -5.738   1.356 -23.094  1.00  0.00           N
ATOM    168  H   GLN A  19      -6.302   0.590 -22.785  1.00  0.00           H
ATOM    169  CA  GLN A  19      -6.240   2.691 -22.912  1.00  0.00           C
ATOM    170  CB  GLN A  19      -7.757   2.669 -23.058  1.00  0.00           C
ATOM    171  CG  GLN A  19      -8.186   1.858 -24.278  1.00  0.00           C
ATOM    172  CD  GLN A  19      -9.684   1.665 -24.235  1.00  0.00           C
ATOM    173  OE1 GLN A  19     -10.448   2.576 -23.935  1.00  0.00           O
ATOM    174  NE2 GLN A  19     -10.060   0.412 -24.541  1.00  0.00           N
ATOM    175 HE21 GLN A  19     -11.017   0.127 -24.554  1.00  0.00           H
ATOM    176 HE22 GLN A  19      -9.359  -0.275 -24.759  1.00  0.00           H
ATOM    177  C   GLN A  19      -5.866   3.270 -21.572  1.00  0.00           C
```

```
ATOM    178  O   GLN A  19      -5.485   4.438 -21.484  1.00  0.00             O
ATOM    179  N   TRP A  20      -5.958   2.442 -20.504  1.00  0.00             N
ATOM    180  H   TRP A  20      -6.243   1.499 -20.686  1.00  0.00             H
ATOM    181  CA  TRP A  20      -5.719   2.836 -19.137  1.00  0.00             C
ATOM    182  CB  TRP A  20      -6.348   1.786 -18.214  1.00  0.00             C
ATOM    183  CG  TRP A  20      -7.852   1.713 -18.404  1.00  0.00             C
ATOM    184  CD2 TRP A  20      -8.766   0.756 -17.824  1.00  0.00             C
ATOM    185  CE2 TRP A  20     -10.068   1.070 -18.273  1.00  0.00             C
ATOM    186  CE3 TRP A  20      -8.583  -0.319 -16.965  1.00  0.00             C
ATOM    187  CD1 TRP A  20      -8.671   2.556 -19.174  1.00  0.00             C
ATOM    188  NE1 TRP A  20      -9.974   2.180 -19.104  1.00  0.00             N
ATOM    189  HE1 TRP A  20     -10.730   2.627 -19.537  1.00  0.00             H
ATOM    190  CZ2 TRP A  20     -11.147   0.303 -17.849  1.00  0.00             C
ATOM    191  CZ3 TRP A  20      -9.668  -1.079 -16.546  1.00  0.00             C
ATOM    192  CH2 TRP A  20     -10.948  -0.768 -16.986  1.00  0.00             C
ATOM    193  C   TRP A  20      -4.259   3.091 -18.827  1.00  0.00             C
ATOM    194  O   TRP A  20      -3.913   4.121 -18.243  1.00  0.00             O
ATOM    195  N   ASN A  21      -3.371   2.170 -19.250  1.00  0.00             N
ATOM    196  H   ASN A  21      -3.734   1.374 -19.736  1.00  0.00             H
ATOM    197  CA  ASN A  21      -1.938   2.237 -19.104  1.00  0.00             C
ATOM    198  CB  ASN A  21      -1.413   3.162 -20.213  1.00  0.00             C
ATOM    199  CG  ASN A  21       0.090   3.152 -20.234  1.00  0.00             C
ATOM    200  OD1 ASN A  21       0.729   4.033 -19.675  1.00  0.00             O
ATOM    201  ND2 ASN A  21       0.603   2.091 -20.872  1.00  0.00             N
ATOM    202 HD21 ASN A  21       1.562   1.855 -20.711  1.00  0.00             H
ATOM    203 HD22 ASN A  21       0.039   1.504 -21.453  1.00  0.00             H
ATOM    204  C   ASN A  21      -1.384   2.495 -17.725  1.00  0.00             C
ATOM    205  O   ASN A  21      -1.363   1.593 -16.883  1.00  0.00             O
ATOM    206  N   LEU A  22      -0.970   3.766 -17.476  1.00  0.00             N
ATOM    207  H   LEU A  22      -1.040   4.423 -18.224  1.00  0.00             H
ATOM    208  CA  LEU A  22      -0.320   4.292 -16.302  1.00  0.00             C
ATOM    209  CB  LEU A  22      -0.310   5.821 -16.341  1.00  0.00             C
ATOM    210  CG  LEU A  22       0.330   6.488 -17.555  1.00  0.00             C
ATOM    211  CD1 LEU A  22       0.050   7.991 -17.576  1.00  0.00             C
ATOM    212  CD2 LEU A  22       1.823   6.200 -17.644  1.00  0.00             C
ATOM    213  C   LEU A  22      -1.026   3.937 -15.040  1.00  0.00             C
ATOM    214  O   LEU A  22      -0.400   3.504 -14.073  1.00  0.00             O
ATOM    215  N   VAL A  23      -2.368   4.043 -15.076  1.00  0.00             N
ATOM    216  H   VAL A  23      -2.780   4.278 -15.957  1.00  0.00             H
ATOM    217  CA  VAL A  23      -3.211   3.788 -13.949  1.00  0.00             C
ATOM    218  CB  VAL A  23      -4.624   4.307 -14.210  1.00  0.00             C
ATOM    219  CG1 VAL A  23      -4.559   5.780 -14.611  1.00  0.00             C
ATOM    220  CG2 VAL A  23      -5.361   3.486 -15.259  1.00  0.00             C
ATOM    221  C   VAL A  23      -3.169   2.360 -13.471  1.00  0.00             C
ATOM    222  O   VAL A  23      -3.294   2.141 -12.268  1.00  0.00             O
ATOM    223  N   ILE A  24      -2.980   1.362 -14.375  1.00  0.00             N
ATOM    224  H   ILE A  24      -2.738   1.577 -15.322  1.00  0.00             H
ATOM    225  CA  ILE A  24      -2.929  -0.008 -13.926  1.00  0.00             C
ATOM    226  CB  ILE A  24      -3.479  -0.953 -14.996  1.00  0.00             C
ATOM    227  CG2 ILE A  24      -3.288  -2.416 -14.597  1.00  0.00             C
ATOM    228  CG1 ILE A  24      -4.942  -0.654 -15.297  1.00  0.00             C
ATOM    229  CD1 ILE A  24      -5.547  -1.733 -16.194  1.00  0.00             C
ATOM    230  C   ILE A  24      -1.523  -0.409 -13.575  1.00  0.00             C
ATOM    231  O   ILE A  24      -1.331  -1.029 -12.530  1.00  0.00             O
ATOM    232  N   GLY A  25      -0.510  -0.010 -14.386  1.00  0.00             N
ATOM    233  H   GLY A  25      -0.730   0.534 -15.199  1.00  0.00             H
ATOM    234  CA  GLY A  25       0.856  -0.413 -14.144  1.00  0.00             C
ATOM    235  C   GLY A  25       1.432   0.062 -12.851  1.00  0.00             C
ATOM    236  O   GLY A  25       2.035  -0.718 -12.112  1.00  0.00             O
```

```
ATOM    237  N   PHE A  26       1.167   1.347 -12.541  1.00  0.00           N
ATOM    238  H   PHE A  26       0.606   1.889 -13.172  1.00  0.00           H
ATOM    239  CA  PHE A  26       1.651   2.003 -11.359  1.00  0.00           C
ATOM    240  CB  PHE A  26       1.233   3.465 -11.473  1.00  0.00           C
ATOM    241  CG  PHE A  26       1.699   4.317 -10.326  1.00  0.00           C
ATOM    242  CD1 PHE A  26       3.047   4.394 -10.011  1.00  0.00           C
ATOM    243  CD2 PHE A  26       0.768   5.057  -9.608  1.00  0.00           C
ATOM    244  CE1 PHE A  26       3.460   5.245  -8.998  1.00  0.00           C
ATOM    245  CE2 PHE A  26       1.184   5.909  -8.595  1.00  0.00           C
ATOM    246  CZ  PHE A  26       2.535   6.013  -8.300  1.00  0.00           C
ATOM    247  C   PHE A  26       1.094   1.380 -10.115  1.00  0.00           C
ATOM    248  O   PHE A  26       1.854   1.042  -9.204  1.00  0.00           O
ATOM    249  N   LEU A  27      -0.233   1.136 -10.105  1.00  0.00           N
ATOM    250  H   LEU A  27      -0.779   1.326 -10.921  1.00  0.00           H
ATOM    251  CA  LEU A  27      -0.892   0.605  -8.949  1.00  0.00           C
ATOM    252  CB  LEU A  27      -2.392   0.841  -9.034  1.00  0.00           C
ATOM    253  CG  LEU A  27      -2.690   2.330  -9.183  1.00  0.00           C
ATOM    254  CD1 LEU A  27      -4.180   2.572  -9.366  1.00  0.00           C
ATOM    255  CD2 LEU A  27      -2.111   3.164  -8.044  1.00  0.00           C
ATOM    256  C   LEU A  27      -0.576  -0.824  -8.689  1.00  0.00           C
ATOM    257  O   LEU A  27      -0.210  -1.164  -7.568  1.00  0.00           O
ATOM    258  N   PHE A  28      -0.635  -1.663  -9.744  1.00  0.00           N
ATOM    259  H   PHE A  28      -0.878  -1.291 -10.642  1.00  0.00           H
ATOM    260  CA  PHE A  28      -0.413  -3.078  -9.643  1.00  0.00           C
ATOM    261  CB  PHE A  28      -0.796  -3.752 -10.959  1.00  0.00           C
ATOM    262  CG  PHE A  28      -0.687  -5.253 -10.852  1.00  0.00           C
ATOM    263  CD1 PHE A  28      -1.439  -5.944  -9.909  1.00  0.00           C
ATOM    264  CD2 PHE A  28       0.148  -5.948 -11.719  1.00  0.00           C
ATOM    265  CE1 PHE A  28      -1.380  -7.332  -9.860  1.00  0.00           C
ATOM    266  CE2 PHE A  28       0.205  -7.335 -11.669  1.00  0.00           C
ATOM    267  CZ  PHE A  28      -0.571  -8.028 -10.749  1.00  0.00           C
ATOM    268  C   PHE A  28       0.980  -3.456  -9.257  1.00  0.00           C
ATOM    269  O   PHE A  28       1.133  -4.208  -8.294  1.00  0.00           O
ATOM    270  N   LEU A  29       2.015  -2.898  -9.928  1.00  0.00           N
ATOM    271  H   LEU A  29       1.845  -2.229 -10.656  1.00  0.00           H
ATOM    272  CA  LEU A  29       3.361  -3.284  -9.594  1.00  0.00           C
ATOM    273  CB  LEU A  29       4.347  -2.942 -10.702  1.00  0.00           C
ATOM    274  CG  LEU A  29       4.032  -3.666 -12.007  1.00  0.00           C
ATOM    275  CD1 LEU A  29       5.125  -3.404 -13.035  1.00  0.00           C
ATOM    276  CD2 LEU A  29       3.807  -5.166 -11.806  1.00  0.00           C
ATOM    277  C   LEU A  29       3.817  -2.806  -8.258  1.00  0.00           C
ATOM    278  O   LEU A  29       4.490  -3.557  -7.548  1.00  0.00           O
ATOM    279  N   THR A  30       3.420  -1.571  -7.866  1.00  0.00           N
ATOM    280  H   THR A  30       2.801  -1.022  -8.427  1.00  0.00           H
ATOM    281  CA  THR A  30       3.808  -1.040  -6.587  1.00  0.00           C
ATOM    282  CB  THR A  30       3.554   0.453  -6.504  1.00  0.00           C
ATOM    283  OG1 THR A  30       4.108   1.104  -7.657  1.00  0.00           O
ATOM    284  HG1 THR A  30       3.376   1.199  -8.262  1.00  0.00           H
ATOM    285  CG2 THR A  30       4.162   1.016  -5.218  1.00  0.00           C
ATOM    286  C   THR A  30       3.116  -1.838  -5.502  1.00  0.00           C
ATOM    287  O   THR A  30       3.748  -2.181  -4.502  1.00  0.00           O
ATOM    288  N   TRP A  31       1.831  -2.217  -5.723  1.00  0.00           N
ATOM    289  H   TRP A  31       1.357  -1.925  -6.559  1.00  0.00           H
ATOM    290  CA  TRP A  31       1.066  -2.972  -4.768  1.00  0.00           C
ATOM    291  CB  TRP A  31      -0.405  -3.084  -5.177  1.00  0.00           C
ATOM    292  CG  TRP A  31      -1.275  -3.436  -3.991  1.00  0.00           C
ATOM    293  CD2 TRP A  31      -1.572  -2.626  -2.831  1.00  0.00           C
ATOM    294  CE2 TRP A  31      -2.453  -3.391  -1.995  1.00  0.00           C
ATOM    295  CE3 TRP A  31      -1.173  -1.332  -2.430  1.00  0.00           C
```

ATOM	296	CD1	TRP	A	31	-1.983	-4.633	-3.798	1.00	0.00	C
ATOM	297	NE1	TRP	A	31	-2.677	-4.611	-2.628	1.00	0.00	N
ATOM	298	HE1	TRP	A	31	-3.246	-5.334	-2.289	1.00	0.00	H
ATOM	299	CZ2	TRP	A	31	-2.916	-2.846	-0.778	1.00	0.00	C
ATOM	300	CZ3	TRP	A	31	-1.642	-0.798	-1.210	1.00	0.00	C
ATOM	301	CH2	TRP	A	31	-2.508	-1.552	-0.389	1.00	0.00	C
ATOM	302	C	TRP	A	31	1.679	-4.332	-4.551	1.00	0.00	C
ATOM	303	O	TRP	A	31	1.664	-4.796	-3.414	1.00	0.00	O
ATOM	304	N	ILE	A	32	2.270	-4.969	-5.601	1.00	0.00	N
ATOM	305	H	ILE	A	32	2.213	-4.547	-6.506	1.00	0.00	H
ATOM	306	CA	ILE	A	32	2.902	-6.272	-5.464	1.00	0.00	C
ATOM	307	CB	ILE	A	32	3.367	-6.817	-6.816	1.00	0.00	C
ATOM	308	CG2	ILE	A	32	4.290	-8.025	-6.642	1.00	0.00	C
ATOM	309	CG1	ILE	A	32	2.181	-7.168	-7.709	1.00	0.00	C
ATOM	310	CD1	ILE	A	32	2.656	-7.764	-9.032	1.00	0.00	C
ATOM	311	C	ILE	A	32	4.087	-6.145	-4.532	1.00	0.00	C
ATOM	312	O	ILE	A	32	4.236	-6.970	-3.630	1.00	0.00	O
ATOM	313	N	CYS	A	33	4.900	-5.069	-4.687	1.00	0.00	N
ATOM	314	H	CYS	A	33	4.725	-4.432	-5.441	1.00	0.00	H
ATOM	315	CA	CYS	A	33	6.073	-4.837	-3.872	1.00	0.00	C
ATOM	316	CB	CYS	A	33	6.835	-3.623	-4.398	1.00	0.00	C
ATOM	317	SG	CYS	A	33	7.226	-3.760	-6.160	1.00	0.00	S
ATOM	318	C	CYS	A	33	5.660	-4.657	-2.437	1.00	0.00	C
ATOM	319	O	CYS	A	33	6.298	-5.190	-1.526	1.00	0.00	O
ATOM	320	N	LEU	A	34	4.530	-3.943	-2.225	1.00	0.00	N
ATOM	321	H	LEU	A	34	4.046	-3.571	-3.020	1.00	0.00	H
ATOM	322	CA	LEU	A	34	3.994	-3.691	-0.919	1.00	0.00	C
ATOM	323	CB	LEU	A	34	2.888	-2.645	-0.979	1.00	0.00	C
ATOM	324	CG	LEU	A	34	3.445	-1.283	-1.384	1.00	0.00	C
ATOM	325	CD1	LEU	A	34	2.331	-0.275	-1.630	1.00	0.00	C
ATOM	326	CD2	LEU	A	34	4.472	-0.767	-0.378	1.00	0.00	C
ATOM	327	C	LEU	A	34	3.503	-4.960	-0.300	1.00	0.00	C
ATOM	328	O	LEU	A	34	3.745	-5.167	0.884	1.00	0.00	O
ATOM	329	N	LEU	A	35	2.860	-5.864	-1.081	1.00	0.00	N
ATOM	330	H	LEU	A	35	2.695	-5.625	-2.039	1.00	0.00	H
ATOM	331	CA	LEU	A	35	2.367	-7.119	-0.567	1.00	0.00	C
ATOM	332	CB	LEU	A	35	1.617	-7.840	-1.687	1.00	0.00	C
ATOM	333	CG	LEU	A	35	0.288	-8.501	-1.323	1.00	0.00	C
ATOM	334	CD1	LEU	A	35	-0.277	-9.271	-2.514	1.00	0.00	C
ATOM	335	CD2	LEU	A	35	0.355	-9.373	-0.073	1.00	0.00	C
ATOM	336	C	LEU	A	35	3.504	-7.977	-0.115	1.00	0.00	C
ATOM	337	O	LEU	A	35	3.369	-8.667	0.894	1.00	0.00	O
ATOM	338	N	GLN	A	36	4.660	-7.903	-0.822	1.00	0.00	N
ATOM	339	H	GLN	A	36	4.688	-7.318	-1.637	1.00	0.00	H
ATOM	340	CA	GLN	A	36	5.833	-8.661	-0.472	1.00	0.00	C
ATOM	341	CB	GLN	A	36	6.916	-8.512	-1.536	1.00	0.00	C
ATOM	342	CG	GLN	A	36	6.503	-9.169	-2.855	1.00	0.00	C
ATOM	343	CD	GLN	A	36	7.588	-8.945	-3.883	1.00	0.00	C
ATOM	344	OE1	GLN	A	36	8.256	-7.916	-3.894	1.00	0.00	O
ATOM	345	NE2	GLN	A	36	7.734	-9.968	-4.743	1.00	0.00	N
ATOM	346	HE21	GLN	A	36	8.378	-9.946	-5.506	1.00	0.00	H
ATOM	347	HE22	GLN	A	36	7.189	-10.811	-4.664	1.00	0.00	H
ATOM	348	C	GLN	A	36	6.302	-8.199	0.884	1.00	0.00	C
ATOM	349	O	GLN	A	36	6.528	-9.031	1.765	1.00	0.00	O
ATOM	350	N	PHE	A	37	6.358	-6.859	1.106	1.00	0.00	N
ATOM	351	H	PHE	A	37	6.121	-6.242	0.351	1.00	0.00	H
ATOM	352	CA	PHE	A	37	6.767	-6.304	2.378	1.00	0.00	C
ATOM	353	CB	PHE	A	37	6.996	-4.794	2.298	1.00	0.00	C
ATOM	354	CG	PHE	A	37	7.934	-4.428	1.174	1.00	0.00	C

```
ATOM    355  CD1 PHE A  37       7.704  -3.263   0.452  1.00  0.00           C
ATOM    356  CD2 PHE A  37       9.020  -5.237   0.861  1.00  0.00           C
ATOM    357  CE1 PHE A  37       8.548  -2.916  -0.596  1.00  0.00           C
ATOM    358  CE2 PHE A  37       9.863  -4.891  -0.188  1.00  0.00           C
ATOM    359  CZ  PHE A  37       9.623  -3.734  -0.921  1.00  0.00           C
ATOM    360  C   PHE A  37       5.775  -6.603   3.473  1.00  0.00           C
ATOM    361  O   PHE A  37       6.175  -6.852   4.609  1.00  0.00           O
ATOM    362  N   ALA A  38       4.466  -6.658   3.121  1.00  0.00           N
ATOM    363  H   ALA A  38       4.264  -6.462   2.160  1.00  0.00           H
ATOM    364  CA  ALA A  38       3.351  -6.906   4.004  1.00  0.00           C
ATOM    365  CB  ALA A  38       2.015  -6.822   3.267  1.00  0.00           C
ATOM    366  C   ALA A  38       3.433  -8.230   4.698  1.00  0.00           C
ATOM    367  O   ALA A  38       2.817  -8.392   5.753  1.00  0.00           O
ATOM    368  N   TYR A  39       4.201  -9.205   4.148  1.00  0.00           N
ATOM    369  H   TYR A  39       4.651  -9.024   3.273  1.00  0.00           H
ATOM    370  CA  TYR A  39       4.345 -10.500   4.769  1.00  0.00           C
ATOM    371  CB  TYR A  39       5.214 -11.432   3.910  1.00  0.00           C
ATOM    372  CG  TYR A  39       4.454 -12.023   2.743  1.00  0.00           C
ATOM    373  CD1 TYR A  39       3.432 -11.318   2.122  1.00  0.00           C
ATOM    374  CE1 TYR A  39       2.759 -11.867   1.041  1.00  0.00           C
ATOM    375  CD2 TYR A  39       4.785 -13.292   2.278  1.00  0.00           C
ATOM    376  CE2 TYR A  39       4.103 -13.850   1.201  1.00  0.00           C
ATOM    377  CZ  TYR A  39       3.088 -13.134   0.580  1.00  0.00           C
ATOM    378  OH  TYR A  39       2.398 -13.667  -0.491  1.00  0.00           O
ATOM    379  HH  TYR A  39       1.725 -13.048  -0.744  1.00  0.00           H
ATOM    380  C   TYR A  39       4.987 -10.372   6.133  1.00  0.00           C
ATOM    381  O   TYR A  39       4.684 -11.155   7.034  1.00  0.00           O
ATOM    382  N   ALA A  40       5.855  -9.345   6.329  1.00  0.00           N
ATOM    383  H   ALA A  40       6.009  -8.698   5.578  1.00  0.00           H
ATOM    384  CA  ALA A  40       6.508  -9.074   7.587  1.00  0.00           C
ATOM    385  CB  ALA A  40       7.541  -7.957   7.444  1.00  0.00           C
ATOM    386  C   ALA A  40       5.491  -8.637   8.612  1.00  0.00           C
ATOM    387  O   ALA A  40       5.634  -8.954   9.799  1.00  0.00           O
ATOM    388  N   ASN A  41       4.446  -7.895   8.149  1.00  0.00           N
ATOM    389  H   ASN A  41       4.377  -7.754   7.161  1.00  0.00           H
ATOM    390  CA  ASN A  41       3.377  -7.382   8.964  1.00  0.00           C
ATOM    391  CB  ASN A  41       2.437  -6.430   8.226  1.00  0.00           C
ATOM    392  CG  ASN A  41       3.086  -5.102   7.940  1.00  0.00           C
ATOM    393  OD1 ASN A  41       4.027  -4.680   8.595  1.00  0.00           O
ATOM    394  ND2 ASN A  41       2.528  -4.465   6.911  1.00  0.00           N
ATOM    395 HD21 ASN A  41       2.912  -3.604   6.584  1.00  0.00           H
ATOM    396 HD22 ASN A  41       1.712  -4.846   6.466  1.00  0.00           H
ATOM    397  C   ASN A  41       2.526  -8.521   9.424  1.00  0.00           C
ATOM    398  O   ASN A  41       2.201  -8.584  10.610  1.00  0.00           O
ATOM    399  N   ARG A  42       2.198  -9.480   8.516  1.00  0.00           N
ATOM    400  H   ARG A  42       2.498  -9.336   7.570  1.00  0.00           H
ATOM    401  CA  ARG A  42       1.376 -10.610   8.880  1.00  0.00           C
ATOM    402  CB  ARG A  42       1.021 -11.388   7.630  1.00  0.00           C
ATOM    403  CG  ARG A  42       0.425 -10.534   6.522  1.00  0.00           C
ATOM    404  CD  ARG A  42      -0.147 -11.425   5.436  1.00  0.00           C
ATOM    405  NE  ARG A  42      -1.178 -12.292   6.002  1.00  0.00           N
ATOM    406  HE  ARG A  42      -1.034 -12.818   6.844  1.00  0.00           H
ATOM    407  CZ  ARG A  42      -2.358 -12.420   5.384  1.00  0.00           C
ATOM    408  NH1 ARG A  42      -2.660 -11.657   4.343  1.00  0.00           N
ATOM    409 HH11 ARG A  42      -2.139 -10.827   4.134  1.00  0.00           H
ATOM    410 HH12 ARG A  42      -3.439 -11.899   3.741  1.00  0.00           H
ATOM    411  NH2 ARG A  42      -3.224 -13.308   5.831  1.00  0.00           N
ATOM    412 HH21 ARG A  42      -3.046 -13.933   6.599  1.00  0.00           H
ATOM    413 HH22 ARG A  42      -4.123 -13.377   5.387  1.00  0.00           H
```

```
ATOM    414  C   ARG A  42       2.085 -11.528   9.838  1.00  0.00           C
ATOM    415  O   ARG A  42       1.448 -12.081  10.735  1.00  0.00           O
ATOM    416  N   ASN A  43       3.423 -11.688   9.681  1.00  0.00           N
ATOM    417  H   ASN A  43       3.862 -11.231   8.908  1.00  0.00           H
ATOM    418  CA  ASN A  43       4.246 -12.502  10.550  1.00  0.00           C
ATOM    419  CB  ASN A  43       5.623 -12.722   9.929  1.00  0.00           C
ATOM    420  CG  ASN A  43       5.767 -14.120   9.372  1.00  0.00           C
ATOM    421  OD1 ASN A  43       5.937 -15.109  10.068  1.00  0.00           O
ATOM    422  ND2 ASN A  43       5.731 -14.158   8.039  1.00  0.00           N
ATOM    423 HD21 ASN A  43       5.831 -15.038   7.564  1.00  0.00           H
ATOM    424 HD22 ASN A  43       5.587 -13.323   7.510  1.00  0.00           H
ATOM    425  C   ASN A  43       4.472 -11.883  11.914  1.00  0.00           C
ATOM    426  O   ASN A  43       4.934 -12.578  12.823  1.00  0.00           O
ATOM    427  N   ARG A  44       4.150 -10.570  12.082  1.00  0.00           N
ATOM    428  H   ARG A  44       3.885 -10.079  11.257  1.00  0.00           H
ATOM    429  CA  ARG A  44       4.272  -9.809  13.308  1.00  0.00           C
ATOM    430  CB  ARG A  44       3.342 -10.333  14.416  1.00  0.00           C
ATOM    431  CG  ARG A  44       1.924 -10.757  14.009  1.00  0.00           C
ATOM    432  CD  ARG A  44       1.066  -9.652  13.399  1.00  0.00           C
ATOM    433  NE  ARG A  44       0.868  -8.542  14.330  1.00  0.00           N
ATOM    434  HE  ARG A  44       0.574  -8.782  15.257  1.00  0.00           H
ATOM    435  CZ  ARG A  44       1.048  -7.274  13.907  1.00  0.00           C
ATOM    436  NH1 ARG A  44       1.471  -7.026  12.668  1.00  0.00           N
ATOM    437 HH11 ARG A  44       1.660  -7.789  12.036  1.00  0.00           H
ATOM    438 HH12 ARG A  44       1.618  -6.104  12.318  1.00  0.00           H
ATOM    439  NH2 ARG A  44       0.792  -6.265  14.735  1.00  0.00           N
ATOM    440 HH21 ARG A  44       0.428  -6.422  15.657  1.00  0.00           H
ATOM    441 HH22 ARG A  44       0.924  -5.311  14.470  1.00  0.00           H
ATOM    442  C   ARG A  44       5.663  -9.687  13.889  1.00  0.00           C
ATOM    443  O   ARG A  44       5.888  -9.901  15.084  1.00  0.00           O
ATOM    444  N   PHE A  45       6.646  -9.346  13.019  1.00  0.00           N
ATOM    445  H   PHE A  45       6.386  -9.146  12.073  1.00  0.00           H
ATOM    446  CA  PHE A  45       8.019  -9.143  13.439  1.00  0.00           C
ATOM    447  CB  PHE A  45       8.960  -9.068  12.234  1.00  0.00           C
ATOM    448  CG  PHE A  45       9.056 -10.389  11.506  1.00  0.00           C
ATOM    449  CD1 PHE A  45       9.482 -10.412  10.184  1.00  0.00           C
ATOM    450  CD2 PHE A  45       8.747 -11.581  12.151  1.00  0.00           C
ATOM    451  CE1 PHE A  45       9.619 -11.625   9.519  1.00  0.00           C
ATOM    452  CE2 PHE A  45       8.887 -12.794  11.488  1.00  0.00           C
ATOM    453  CZ  PHE A  45       9.335 -12.817  10.173  1.00  0.00           C
ATOM    454  C   PHE A  45       8.106  -7.848  14.212  1.00  0.00           C
ATOM    455  O   PHE A  45       7.470  -6.864  13.847  1.00  0.00           O
ATOM    456  N   LEU A  46       8.899  -7.774  15.305  1.00  0.00           N
ATOM    457  H   LEU A  46       9.364  -8.600  15.624  1.00  0.00           H
ATOM    458  CA  LEU A  46       9.024  -6.513  16.021  1.00  0.00           C
ATOM    459  CB  LEU A  46       9.773  -6.680  17.340  1.00  0.00           C
ATOM    460  CG  LEU A  46       8.899  -7.275  18.442  1.00  0.00           C
ATOM    461  CD1 LEU A  46       9.687  -7.483  19.737  1.00  0.00           C
ATOM    462  CD2 LEU A  46       7.637  -6.441  18.666  1.00  0.00           C
ATOM    463  C   LEU A  46       9.704  -5.465  15.173  1.00  0.00           C
ATOM    464  O   LEU A  46       9.417  -4.270  15.295  1.00  0.00           O
ATOM    465  N   TYR A  47      10.564  -5.930  14.235  1.00  0.00           N
ATOM    466  H   TYR A  47      10.710  -6.916  14.194  1.00  0.00           H
ATOM    467  CA  TYR A  47      11.293  -5.109  13.311  1.00  0.00           C
ATOM    468  CB  TYR A  47      12.512  -5.867  12.800  1.00  0.00           C
ATOM    469  CG  TYR A  47      13.591  -5.664  13.839  1.00  0.00           C
ATOM    470  CD1 TYR A  47      14.315  -6.733  14.359  1.00  0.00           C
ATOM    471  CE1 TYR A  47      15.321  -6.502  15.294  1.00  0.00           C
ATOM    472  CD2 TYR A  47      13.853  -4.372  14.276  1.00  0.00           C
```

ATOM	473	CE2	TYR	A	47	14.861	-4.140	15.201	1.00	0.00	C
ATOM	474	CZ	TYR	A	47	15.608	-5.201	15.699	1.00	0.00	C
ATOM	475	OH	TYR	A	47	16.638	-4.928	16.577	1.00	0.00	O
ATOM	476	HH	TYR	A	47	16.810	-5.689	17.136	1.00	0.00	H
ATOM	477	C	TYR	A	47	10.447	-4.455	12.248	1.00	0.00	C
ATOM	478	O	TYR	A	47	10.982	-3.659	11.470	1.00	0.00	O
ATOM	479	N	ILE	A	48	9.110	-4.746	12.214	1.00	0.00	N
ATOM	480	H	ILE	A	48	8.745	-5.425	12.851	1.00	0.00	H
ATOM	481	CA	ILE	A	48	8.151	-4.172	11.287	1.00	0.00	C
ATOM	482	CB	ILE	A	48	6.754	-4.592	11.761	1.00	0.00	C
ATOM	483	CG2	ILE	A	48	5.682	-3.520	11.594	1.00	0.00	C
ATOM	484	CG1	ILE	A	48	6.310	-5.903	11.136	1.00	0.00	C
ATOM	485	CD1	ILE	A	48	5.019	-6.347	11.824	1.00	0.00	C
ATOM	486	C	ILE	A	48	8.243	-2.680	11.264	1.00	0.00	C
ATOM	487	O	ILE	A	48	8.154	-2.124	10.173	1.00	0.00	O
ATOM	488	N	ILE	A	49	8.534	-2.036	12.420	1.00	0.00	N
ATOM	489	H	ILE	A	49	8.685	-2.593	13.233	1.00	0.00	H
ATOM	490	CA	ILE	A	49	8.596	-0.598	12.520	1.00	0.00	C
ATOM	491	CB	ILE	A	49	8.944	-0.158	13.945	1.00	0.00	C
ATOM	492	CG2	ILE	A	49	10.327	-0.645	14.381	1.00	0.00	C
ATOM	493	CG1	ILE	A	49	8.757	1.354	14.086	1.00	0.00	C
ATOM	494	CD1	ILE	A	49	8.893	1.855	15.521	1.00	0.00	C
ATOM	495	C	ILE	A	49	9.487	0.047	11.483	1.00	0.00	C
ATOM	496	O	ILE	A	49	9.087	1.055	10.905	1.00	0.00	O
ATOM	497	N	LYS	A	50	10.658	-0.550	11.176	1.00	0.00	N
ATOM	498	H	LYS	A	50	10.842	-1.472	11.519	1.00	0.00	H
ATOM	499	CA	LYS	A	50	11.547	0.019	10.204	1.00	0.00	C
ATOM	500	CB	LYS	A	50	12.984	-0.405	10.500	1.00	0.00	C
ATOM	501	CG	LYS	A	50	13.379	-0.008	11.926	1.00	0.00	C
ATOM	502	CD	LYS	A	50	14.825	-0.357	12.283	1.00	0.00	C
ATOM	503	CE	LYS	A	50	15.186	-0.002	13.728	1.00	0.00	C
ATOM	504	NZ	LYS	A	50	16.574	-0.404	14.005	1.00	0.00	N
ATOM	505	HZ1	LYS	A	50	17.212	0.065	13.331	1.00	0.00	H
ATOM	506	HZ2	LYS	A	50	16.830	-0.124	14.974	1.00	0.00	H
ATOM	507	HZ3	LYS	A	50	16.669	-1.436	13.913	1.00	0.00	H
ATOM	508	C	LYS	A	50	11.143	-0.212	8.777	1.00	0.00	C
ATOM	509	O	LYS	A	50	11.573	0.533	7.899	1.00	0.00	O
ATOM	510	N	LEU	A	51	10.350	-1.273	8.508	1.00	0.00	N
ATOM	511	H	LEU	A	51	9.947	-1.772	9.275	1.00	0.00	H
ATOM	512	CA	LEU	A	51	9.877	-1.597	7.183	1.00	0.00	C
ATOM	513	CB	LEU	A	51	9.584	-3.095	7.124	1.00	0.00	C
ATOM	514	CG	LEU	A	51	10.801	-3.925	7.538	1.00	0.00	C
ATOM	515	CD1	LEU	A	51	10.420	-5.345	7.955	1.00	0.00	C
ATOM	516	CD2	LEU	A	51	11.889	-3.908	6.463	1.00	0.00	C
ATOM	517	C	LEU	A	51	8.659	-0.799	6.799	1.00	0.00	C
ATOM	518	O	LEU	A	51	8.540	-0.329	5.662	1.00	0.00	O
ATOM	519	N	ILE	A	52	7.742	-0.588	7.778	1.00	0.00	N
ATOM	520	H	ILE	A	52	7.930	-0.959	8.687	1.00	0.00	H
ATOM	521	CA	ILE	A	52	6.507	0.122	7.574	1.00	0.00	C
ATOM	522	CB	ILE	A	52	5.486	-0.039	8.695	1.00	0.00	C
ATOM	523	CG2	ILE	A	52	4.989	-1.480	8.733	1.00	0.00	C
ATOM	524	CG1	ILE	A	52	5.992	0.499	10.030	1.00	0.00	C
ATOM	525	CD1	ILE	A	52	4.942	0.396	11.133	1.00	0.00	C
ATOM	526	C	ILE	A	52	6.650	1.549	7.207	1.00	0.00	C
ATOM	527	O	ILE	A	52	5.687	2.116	6.709	1.00	0.00	O
ATOM	528	N	PHE	A	53	7.835	2.164	7.402	1.00	0.00	N
ATOM	529	H	PHE	A	53	8.585	1.659	7.826	1.00	0.00	H
ATOM	530	CA	PHE	A	53	8.028	3.544	7.049	1.00	0.00	C
ATOM	531	CB	PHE	A	53	9.433	3.982	7.457	1.00	0.00	C

ATOM	532	CG	PHE	A	53	9.580	3.960	8.961	1.00	0.00	C
ATOM	533	CD1	PHE	A	53	10.845	3.850	9.524	1.00	0.00	C
ATOM	534	CD2	PHE	A	53	8.462	4.059	9.782	1.00	0.00	C
ATOM	535	CE1	PHE	A	53	10.996	3.851	10.905	1.00	0.00	C
ATOM	536	CE2	PHE	A	53	8.612	4.055	11.164	1.00	0.00	C
ATOM	537	CZ	PHE	A	53	9.880	3.961	11.725	1.00	0.00	C
ATOM	538	C	PHE	A	53	7.796	3.692	5.550	1.00	0.00	C
ATOM	539	O	PHE	A	53	6.920	4.451	5.131	1.00	0.00	O
ATOM	540	N	LEU	A	54	8.490	2.870	4.726	1.00	0.00	N
ATOM	541	H	LEU	A	54	9.084	2.182	5.144	1.00	0.00	H
ATOM	542	CA	LEU	A	54	8.350	2.884	3.289	1.00	0.00	C
ATOM	543	CB	LEU	A	54	9.539	2.206	2.612	1.00	0.00	C
ATOM	544	CG	LEU	A	54	10.781	3.096	2.613	1.00	0.00	C
ATOM	545	CD1	LEU	A	54	12.008	2.364	2.068	1.00	0.00	C
ATOM	546	CD2	LEU	A	54	10.526	4.409	1.869	1.00	0.00	C
ATOM	547	C	LEU	A	54	7.058	2.259	2.845	1.00	0.00	C
ATOM	548	O	LEU	A	54	6.421	2.756	1.909	1.00	0.00	O
ATOM	549	N	TRP	A	55	6.640	1.176	3.554	1.00	0.00	N
ATOM	550	H	TRP	A	55	7.222	0.853	4.302	1.00	0.00	H
ATOM	551	CA	TRP	A	55	5.452	0.419	3.243	1.00	0.00	C
ATOM	552	CB	TRP	A	55	5.399	-0.776	4.192	1.00	0.00	C
ATOM	553	CG	TRP	A	55	4.269	-1.695	3.823	1.00	0.00	C
ATOM	554	CD2	TRP	A	55	2.974	-1.808	4.442	1.00	0.00	C
ATOM	555	CE2	TRP	A	55	2.245	-2.810	3.722	1.00	0.00	C
ATOM	556	CE3	TRP	A	55	2.376	-1.154	5.535	1.00	0.00	C
ATOM	557	CD1	TRP	A	55	4.259	-2.625	2.780	1.00	0.00	C
ATOM	558	NE1	TRP	A	55	3.074	-3.284	2.718	1.00	0.00	N
ATOM	559	HE1	TRP	A	55	2.849	-3.990	2.076	1.00	0.00	H
ATOM	560	CZ2	TRP	A	55	0.927	-3.130	4.101	1.00	0.00	C
ATOM	561	CZ3	TRP	A	55	1.057	-1.486	5.908	1.00	0.00	C
ATOM	562	CH2	TRP	A	55	0.336	-2.462	5.191	1.00	0.00	C
ATOM	563	C	TRP	A	55	4.217	1.269	3.344	1.00	0.00	C
ATOM	564	O	TRP	A	55	3.322	1.120	2.520	1.00	0.00	O
ATOM	565	N	LEU	A	56	4.138	2.154	4.357	1.00	0.00	N
ATOM	566	H	LEU	A	56	4.920	2.255	4.972	1.00	0.00	H
ATOM	567	CA	LEU	A	56	3.033	3.048	4.572	1.00	0.00	C
ATOM	568	CB	LEU	A	56	2.932	3.514	6.025	1.00	0.00	C
ATOM	569	CG	LEU	A	56	2.629	2.408	7.040	1.00	0.00	C
ATOM	570	CD1	LEU	A	56	2.807	2.893	8.479	1.00	0.00	C
ATOM	571	CD2	LEU	A	56	1.249	1.796	6.834	1.00	0.00	C
ATOM	572	C	LEU	A	56	3.126	4.230	3.646	1.00	0.00	C
ATOM	573	O	LEU	A	56	2.082	4.739	3.235	1.00	0.00	O
ATOM	574	N	LEU	A	57	4.368	4.688	3.298	1.00	0.00	N
ATOM	575	H	LEU	A	57	5.180	4.266	3.703	1.00	0.00	H
ATOM	576	CA	LEU	A	57	4.550	5.817	2.408	1.00	0.00	C
ATOM	577	CB	LEU	A	57	6.043	6.092	2.254	1.00	0.00	C
ATOM	578	CG	LEU	A	57	6.523	7.423	2.808	1.00	0.00	C
ATOM	579	CD1	LEU	A	57	7.946	7.723	2.342	1.00	0.00	C
ATOM	580	CD2	LEU	A	57	5.569	8.557	2.468	1.00	0.00	C
ATOM	581	C	LEU	A	57	4.026	5.654	1.020	1.00	0.00	C
ATOM	582	O	LEU	A	57	3.289	6.528	0.567	1.00	0.00	O
ATOM	583	N	TRP	A	58	4.363	4.531	0.334	1.00	0.00	N
ATOM	584	H	TRP	A	58	4.932	3.849	0.801	1.00	0.00	H
ATOM	585	CA	TRP	A	58	3.952	4.322	-1.043	1.00	0.00	C
ATOM	586	CB	TRP	A	58	4.614	3.081	-1.660	1.00	0.00	C
ATOM	587	CG	TRP	A	58	6.125	3.221	-1.666	1.00	0.00	C
ATOM	588	CD2	TRP	A	58	7.101	2.199	-1.958	1.00	0.00	C
ATOM	589	CE2	TRP	A	58	8.386	2.778	-1.830	1.00	0.00	C
ATOM	590	CE3	TRP	A	58	6.983	0.862	-2.314	1.00	0.00	C

```
ATOM    591  CD1 TRP A  58       6.887   4.367  -1.384  1.00  0.00           C
ATOM    592  NE1 TRP A  58       8.219   4.112  -1.476  1.00  0.00           N
ATOM    593  HE1 TRP A  58       8.940   4.757  -1.302  1.00  0.00           H
ATOM    594  CZ2 TRP A  58       9.515   2.000  -2.057  1.00  0.00           C
ATOM    595  CZ3 TRP A  58       8.120   0.096  -2.540  1.00  0.00           C
ATOM    596  CH2 TRP A  58       9.382   0.663  -2.410  1.00  0.00           C
ATOM    597  C   TRP A  58       2.444   4.397  -1.269  1.00  0.00           C
ATOM    598  O   TRP A  58       2.041   5.063  -2.234  1.00  0.00           O
ATOM    599  N   PRO A  59       1.569   3.827  -0.417  1.00  0.00           N
ATOM    600  CD  PRO A  59       1.874   2.909   0.674  1.00  0.00           C
ATOM    601  CA  PRO A  59       0.137   3.894  -0.571  1.00  0.00           C
ATOM    602  CB  PRO A  59      -0.454   3.044   0.555  1.00  0.00           C
ATOM    603  CG  PRO A  59       0.638   2.046   0.868  1.00  0.00           C
ATOM    604  C   PRO A  59      -0.411   5.282  -0.524  1.00  0.00           C
ATOM    605  O   PRO A  59      -1.551   5.422  -0.952  1.00  0.00           O
ATOM    606  N   VAL A  60       0.336   6.304  -0.025  1.00  0.00           N
ATOM    607  H   VAL A  60       1.274   6.150   0.294  1.00  0.00           H
ATOM    608  CA  VAL A  60      -0.181   7.654   0.036  1.00  0.00           C
ATOM    609  CB  VAL A  60       0.768   8.572   0.802  1.00  0.00           C
ATOM    610  CG1 VAL A  60       0.268  10.017   0.780  1.00  0.00           C
ATOM    611  CG2 VAL A  60       0.971   8.052   2.226  1.00  0.00           C
ATOM    612  C   VAL A  60      -0.384   8.126  -1.387  1.00  0.00           C
ATOM    613  O   VAL A  60      -1.468   8.598  -1.746  1.00  0.00           O
ATOM    614  N   THR A  61       0.642   7.914  -2.239  1.00  0.00           N
ATOM    615  H   THR A  61       1.491   7.554  -1.853  1.00  0.00           H
ATOM    616  CA  THR A  61       0.583   8.297  -3.618  1.00  0.00           C
ATOM    617  CB  THR A  61       2.028   8.324  -4.055  1.00  0.00           C
ATOM    618  OG1 THR A  61       2.828   7.609  -3.097  1.00  0.00           O
ATOM    619  HG1 THR A  61       2.627   6.679  -3.170  1.00  0.00           H
ATOM    620  CG2 THR A  61       2.489   9.774  -4.108  1.00  0.00           C
ATOM    621  C   THR A  61      -0.275   7.371  -4.393  1.00  0.00           C
ATOM    622  O   THR A  61      -1.001   7.836  -5.272  1.00  0.00           O
ATOM    623  N   LEU A  62      -0.246   6.052  -4.051  1.00  0.00           N
ATOM    624  H   LEU A  62       0.341   5.748  -3.299  1.00  0.00           H
ATOM    625  CA  LEU A  62      -1.056   5.098  -4.768  1.00  0.00           C
ATOM    626  CB  LEU A  62      -0.824   3.666  -4.301  1.00  0.00           C
ATOM    627  CG  LEU A  62       0.567   3.098  -4.553  1.00  0.00           C
ATOM    628  CD1 LEU A  62       0.567   1.601  -4.257  1.00  0.00           C
ATOM    629  CD2 LEU A  62       1.086   3.393  -5.959  1.00  0.00           C
ATOM    630  C   LEU A  62      -2.509   5.438  -4.615  1.00  0.00           C
ATOM    631  O   LEU A  62      -3.235   5.394  -5.602  1.00  0.00           O
ATOM    632  N   ALA A  63      -2.939   5.854  -3.400  1.00  0.00           N
ATOM    633  H   ALA A  63      -2.283   5.912  -2.647  1.00  0.00           H
ATOM    634  CA  ALA A  63      -4.300   6.208  -3.096  1.00  0.00           C
ATOM    635  CB  ALA A  63      -4.516   6.456  -1.616  1.00  0.00           C
ATOM    636  C   ALA A  63      -4.711   7.438  -3.812  1.00  0.00           C
ATOM    637  O   ALA A  63      -5.806   7.460  -4.367  1.00  0.00           O
ATOM    638  N   CYS A  64      -3.825   8.463  -3.864  1.00  0.00           N
ATOM    639  H   CYS A  64      -2.969   8.408  -3.345  1.00  0.00           H
ATOM    640  CA  CYS A  64      -4.132   9.701  -4.532  1.00  0.00           C
ATOM    641  CB  CYS A  64      -3.027  10.720  -4.267  1.00  0.00           C
ATOM    642  SG  CYS A  64      -2.885  11.138  -2.513  1.00  0.00           S
ATOM    643  C   CYS A  64      -4.343   9.463  -6.002  1.00  0.00           C
ATOM    644  O   CYS A  64      -5.254  10.036  -6.600  1.00  0.00           O
ATOM    645  N   PHE A  65      -3.529   8.555  -6.585  1.00  0.00           N
ATOM    646  H   PHE A  65      -2.852   8.080  -6.019  1.00  0.00           H
ATOM    647  CA  PHE A  65      -3.569   8.233  -7.977  1.00  0.00           C
ATOM    648  CB  PHE A  65      -2.293   7.473  -8.333  1.00  0.00           C
ATOM    649  CG  PHE A  65      -2.050   7.473  -9.821  1.00  0.00           C
```

ATOM	650	CD1	PHE	A	65	-1.543	8.612	-10.436	1.00	0.00	C
ATOM	651	CD2	PHE	A	65	-2.309	6.333	-10.570	1.00	0.00	C
ATOM	652	CE1	PHE	A	65	-1.271	8.603	-11.799	1.00	0.00	C
ATOM	653	CE2	PHE	A	65	-2.033	6.326	-11.931	1.00	0.00	C
ATOM	654	CZ	PHE	A	65	-1.506	7.455	-12.545	1.00	0.00	C
ATOM	655	C	PHE	A	65	-4.805	7.422	-8.312	1.00	0.00	C
ATOM	656	O	PHE	A	65	-5.454	7.747	-9.301	1.00	0.00	O
ATOM	657	N	VAL	A	66	-5.202	6.416	-7.474	1.00	0.00	N
ATOM	658	H	VAL	A	66	-4.641	6.233	-6.665	1.00	0.00	H
ATOM	659	CA	VAL	A	66	-6.349	5.555	-7.730	1.00	0.00	C
ATOM	660	CB	VAL	A	66	-6.626	4.424	-6.729	1.00	0.00	C
ATOM	661	CG1	VAL	A	66	-7.687	3.529	-7.367	1.00	0.00	C
ATOM	662	CG2	VAL	A	66	-5.448	3.587	-6.261	1.00	0.00	C
ATOM	663	C	VAL	A	66	-7.590	6.412	-7.644	1.00	0.00	C
ATOM	664	O	VAL	A	66	-8.422	6.360	-8.548	1.00	0.00	O
ATOM	665	N	LEU	A	67	-7.708	7.245	-6.579	1.00	0.00	N
ATOM	666	H	LEU	A	67	-6.966	7.276	-5.908	1.00	0.00	H
ATOM	667	CA	LEU	A	67	-8.875	8.057	-6.349	1.00	0.00	C
ATOM	668	CB	LEU	A	67	-8.811	8.765	-4.998	1.00	0.00	C
ATOM	669	CG	LEU	A	67	-8.861	7.782	-3.827	1.00	0.00	C
ATOM	670	CD1	LEU	A	67	-8.710	8.496	-2.484	1.00	0.00	C
ATOM	671	CD2	LEU	A	67	-10.101	6.886	-3.879	1.00	0.00	C
ATOM	672	C	LEU	A	67	-9.106	9.031	-7.455	1.00	0.00	C
ATOM	673	O	LEU	A	67	-10.239	9.138	-7.927	1.00	0.00	O
ATOM	674	N	ALA	A	68	-8.030	9.690	-7.944	1.00	0.00	N
ATOM	675	H	ALA	A	68	-7.132	9.538	-7.525	1.00	0.00	H
ATOM	676	CA	ALA	A	68	-8.124	10.633	-9.029	1.00	0.00	C
ATOM	677	CB	ALA	A	68	-6.794	11.359	-9.234	1.00	0.00	C
ATOM	678	C	ALA	A	68	-8.490	9.912	-10.308	1.00	0.00	C
ATOM	679	O	ALA	A	68	-9.282	10.414	-11.108	1.00	0.00	O
ATOM	680	N	ALA	A	69	-7.945	8.687	-10.483	1.00	0.00	N
ATOM	681	H	ALA	A	69	-7.365	8.310	-9.758	1.00	0.00	H
ATOM	682	CA	ALA	A	69	-8.136	7.854	-11.633	1.00	0.00	C
ATOM	683	CB	ALA	A	69	-6.866	7.080	-11.973	1.00	0.00	C
ATOM	684	C	ALA	A	69	-9.294	6.892	-11.589	1.00	0.00	C
ATOM	685	O	ALA	A	69	-9.428	6.124	-12.540	1.00	0.00	O
ATOM	686	N	VAL	A	70	-10.170	6.903	-10.540	1.00	0.00	N
ATOM	687	H	VAL	A	70	-9.937	7.472	-9.749	1.00	0.00	H
ATOM	688	CA	VAL	A	70	-11.317	5.995	-10.438	1.00	0.00	C
ATOM	689	CB	VAL	A	70	-12.170	6.315	-9.199	1.00	0.00	C
ATOM	690	CG1	VAL	A	70	-13.563	5.683	-9.260	1.00	0.00	C
ATOM	691	CG2	VAL	A	70	-11.456	5.878	-7.921	1.00	0.00	C
ATOM	692	C	VAL	A	70	-12.166	6.054	-11.701	1.00	0.00	C
ATOM	693	O	VAL	A	70	-12.690	5.028	-12.130	1.00	0.00	O
ATOM	694	N	TYR	A	71	-12.264	7.234	-12.358	1.00	0.00	N
ATOM	695	H	TYR	A	71	-11.782	8.016	-11.965	1.00	0.00	H
ATOM	696	CA	TYR	A	71	-13.026	7.398	-13.577	1.00	0.00	C
ATOM	697	CB	TYR	A	71	-13.072	8.874	-13.964	1.00	0.00	C
ATOM	698	CG	TYR	A	71	-13.697	9.652	-12.831	1.00	0.00	C
ATOM	699	CD1	TYR	A	71	-12.938	10.566	-12.108	1.00	0.00	C
ATOM	700	CE1	TYR	A	71	-13.519	11.276	-11.063	1.00	0.00	C
ATOM	701	CD2	TYR	A	71	-15.034	9.447	-12.510	1.00	0.00	C
ATOM	702	CE2	TYR	A	71	-15.614	10.158	-11.466	1.00	0.00	C
ATOM	703	CZ	TYR	A	71	-14.857	11.073	-10.744	1.00	0.00	C
ATOM	704	OH	TYR	A	71	-15.439	11.782	-9.712	1.00	0.00	O
ATOM	705	HH	TYR	A	71	-16.343	11.516	-9.616	1.00	0.00	H
ATOM	706	C	TYR	A	71	-12.444	6.557	-14.694	1.00	0.00	C
ATOM	707	O	TYR	A	71	-13.190	6.004	-15.504	1.00	0.00	O
ATOM	708	N	ARG	A	72	-11.093	6.461	-14.752	1.00	0.00	N

ATOM	709	H	ARG	A	72	-10.573	6.810	-13.974	1.00	0.00	H
ATOM	710	CA	ARG	A	72	-10.366	5.697	-15.731	1.00	0.00	C
ATOM	711	CB	ARG	A	72	-8.931	6.191	-15.886	1.00	0.00	C
ATOM	712	CG	ARG	A	72	-8.713	7.425	-16.754	1.00	0.00	C
ATOM	713	CD	ARG	A	72	-7.209	7.660	-16.912	1.00	0.00	C
ATOM	714	NE	ARG	A	72	-6.919	8.710	-17.885	1.00	0.00	N
ATOM	715	HE	ARG	A	72	-7.196	9.636	-17.607	1.00	0.00	H
ATOM	716	CZ	ARG	A	72	-6.323	8.370	-19.072	1.00	0.00	C
ATOM	717	NH1	ARG	A	72	-6.010	7.073	-19.320	1.00	0.00	N
ATOM	718	HH11	ARG	A	72	-6.175	6.353	-18.640	1.00	0.00	H
ATOM	719	HH12	ARG	A	72	-5.601	6.749	-20.183	1.00	0.00	H
ATOM	720	NH2	ARG	A	72	-6.058	9.342	-19.974	1.00	0.00	N
ATOM	721	HH21	ARG	A	72	-6.287	10.303	-19.791	1.00	0.00	H
ATOM	722	HH22	ARG	A	72	-5.622	9.153	-20.860	1.00	0.00	H
ATOM	723	C	ARG	A	72	-10.257	4.225	-15.444	1.00	0.00	C
ATOM	724	O	ARG	A	72	-10.240	3.449	-16.392	1.00	0.00	O
ATOM	725	N	ILE	A	73	-10.113	3.804	-14.162	1.00	0.00	N
ATOM	726	H	ILE	A	73	-10.167	4.488	-13.433	1.00	0.00	H
ATOM	727	CA	ILE	A	73	-9.976	2.398	-13.825	1.00	0.00	C
ATOM	728	CB	ILE	A	73	-8.756	2.131	-12.942	1.00	0.00	C
ATOM	729	CG2	ILE	A	73	-7.891	1.004	-13.506	1.00	0.00	C
ATOM	730	CG1	ILE	A	73	-7.982	3.394	-12.614	1.00	0.00	C
ATOM	731	CD1	ILE	A	73	-7.077	3.194	-11.405	1.00	0.00	C
ATOM	732	C	ILE	A	73	-11.091	1.637	-13.196	1.00	0.00	C
ATOM	733	O	ILE	A	73	-11.133	0.416	-13.362	1.00	0.00	O
ATOM	734	N	ASN	A	74	-12.036	2.328	-12.513	1.00	0.00	N
ATOM	735	H	ASN	A	74	-11.913	3.313	-12.407	1.00	0.00	H
ATOM	736	CA	ASN	A	74	-13.098	1.715	-11.743	1.00	0.00	C
ATOM	737	CB	ASN	A	74	-14.051	0.839	-12.558	1.00	0.00	C
ATOM	738	CG	ASN	A	74	-15.205	0.452	-11.657	1.00	0.00	C
ATOM	739	OD1	ASN	A	74	-15.099	-0.420	-10.800	1.00	0.00	O
ATOM	740	ND2	ASN	A	74	-16.318	1.171	-11.894	1.00	0.00	N
ATOM	741	HD21	ASN	A	74	-17.152	1.048	-11.356	1.00	0.00	H
ATOM	742	HD22	ASN	A	74	-16.334	1.863	-12.619	1.00	0.00	H
ATOM	743	C	ASN	A	74	-12.326	0.984	-10.665	1.00	0.00	C
ATOM	744	O	ASN	A	74	-11.651	1.687	-9.907	1.00	0.00	O
ATOM	745	N	TRP	A	75	-12.381	-0.382	-10.553	1.00	0.00	N
ATOM	746	H	TRP	A	75	-12.995	-0.892	-11.152	1.00	0.00	H
ATOM	747	CA	TRP	A	75	-11.649	-1.157	-9.557	1.00	0.00	C
ATOM	748	CB	TRP	A	75	-10.145	-1.191	-9.854	1.00	0.00	C
ATOM	749	CG	TRP	A	75	-9.810	-2.018	-11.076	1.00	0.00	C
ATOM	750	CD2	TRP	A	75	-8.495	-2.430	-11.504	1.00	0.00	C
ATOM	751	CE2	TRP	A	75	-8.645	-3.184	-12.691	1.00	0.00	C
ATOM	752	CE3	TRP	A	75	-7.227	-2.221	-10.976	1.00	0.00	C
ATOM	753	CD1	TRP	A	75	-10.684	-2.550	-12.037	1.00	0.00	C
ATOM	754	NE1	TRP	A	75	-10.002	-3.237	-12.992	1.00	0.00	N
ATOM	755	HE1	TRP	A	75	-10.399	-3.674	-13.774	1.00	0.00	H
ATOM	756	CZ2	TRP	A	75	-7.521	-3.708	-13.316	1.00	0.00	C
ATOM	757	CZ3	TRP	A	75	-6.111	-2.751	-11.612	1.00	0.00	C
ATOM	758	CH2	TRP	A	75	-6.259	-3.493	-12.777	1.00	0.00	C
ATOM	759	C	TRP	A	75	-11.871	-0.550	-8.196	1.00	0.00	C
ATOM	760	O	TRP	A	75	-10.936	-0.270	-7.447	1.00	0.00	O
ATOM	761	N	ILE	A	76	-13.163	-0.330	-7.881	1.00	0.00	N
ATOM	762	H	ILE	A	76	-13.825	-0.582	-8.588	1.00	0.00	H
ATOM	763	CA	ILE	A	76	-13.638	0.314	-6.685	1.00	0.00	C
ATOM	764	CB	ILE	A	76	-15.164	0.429	-6.731	1.00	0.00	C
ATOM	765	CG2	ILE	A	76	-15.738	1.019	-5.442	1.00	0.00	C
ATOM	766	CG1	ILE	A	76	-15.590	1.247	-7.951	1.00	0.00	C
ATOM	767	CD1	ILE	A	76	-17.107	1.274	-8.140	1.00	0.00	C

ATOM	768	C	ILE	A	76	-13.170	-0.377	-5.430	1.00	0.00	C
ATOM	769	O	ILE	A	76	-12.749	0.316	-4.505	1.00	0.00	O
ATOM	770	N	THR	A	77	-13.145	-1.731	-5.396	1.00	0.00	N
ATOM	771	H	THR	A	77	-13.526	-2.254	-6.156	1.00	0.00	H
ATOM	772	CA	THR	A	77	-12.719	-2.464	-4.224	1.00	0.00	C
ATOM	773	CB	THR	A	77	-13.085	-3.901	-4.530	1.00	0.00	C
ATOM	774	OG1	THR	A	77	-14.127	-3.872	-5.518	1.00	0.00	O
ATOM	775	HG1	THR	A	77	-14.482	-4.754	-5.559	1.00	0.00	H
ATOM	776	CG2	THR	A	77	-13.515	-4.675	-3.282	1.00	0.00	C
ATOM	777	C	THR	A	77	-11.238	-2.239	-3.981	1.00	0.00	C
ATOM	778	O	THR	A	77	-10.839	-1.992	-2.838	1.00	0.00	O
ATOM	779	N	GLY	A	78	-10.416	-2.236	-5.062	1.00	0.00	N
ATOM	780	H	GLY	A	78	-10.818	-2.253	-5.976	1.00	0.00	H
ATOM	781	CA	GLY	A	78	-8.993	-2.035	-4.947	1.00	0.00	C
ATOM	782	C	GLY	A	78	-8.683	-0.617	-4.565	1.00	0.00	C
ATOM	783	O	GLY	A	78	-7.765	-0.381	-3.780	1.00	0.00	O
ATOM	784	N	GLY	A	79	-9.488	0.346	-5.074	1.00	0.00	N
ATOM	785	H	GLY	A	79	-10.213	0.082	-5.710	1.00	0.00	H
ATOM	786	CA	GLY	A	79	-9.323	1.744	-4.809	1.00	0.00	C
ATOM	787	C	GLY	A	79	-9.594	2.102	-3.394	1.00	0.00	C
ATOM	788	O	GLY	A	79	-8.839	2.877	-2.807	1.00	0.00	O
ATOM	789	N	ILE	A	80	-10.662	1.508	-2.816	1.00	0.00	N
ATOM	790	H	ILE	A	80	-11.234	0.912	-3.386	1.00	0.00	H
ATOM	791	CA	ILE	A	80	-11.038	1.756	-1.448	1.00	0.00	C
ATOM	792	CB	ILE	A	80	-12.447	1.211	-1.151	1.00	0.00	C
ATOM	793	CG2	ILE	A	80	-12.780	1.189	0.346	1.00	0.00	C
ATOM	794	CG1	ILE	A	80	-13.492	2.027	-1.917	1.00	0.00	C
ATOM	795	CD1	ILE	A	80	-14.918	1.509	-1.713	1.00	0.00	C
ATOM	796	C	ILE	A	80	-9.992	1.145	-0.552	1.00	0.00	C
ATOM	797	O	ILE	A	80	-9.601	1.788	0.419	1.00	0.00	O
ATOM	798	N	ALA	A	81	-9.456	-0.053	-0.904	1.00	0.00	N
ATOM	799	H	ALA	A	81	-9.842	-0.528	-1.698	1.00	0.00	H
ATOM	800	CA	ALA	A	81	-8.461	-0.725	-0.101	1.00	0.00	C
ATOM	801	CB	ALA	A	81	-8.142	-2.107	-0.672	1.00	0.00	C
ATOM	802	C	ALA	A	81	-7.191	0.074	-0.007	1.00	0.00	C
ATOM	803	O	ALA	A	81	-6.637	0.219	1.084	1.00	0.00	O
ATOM	804	N	ILE	A	82	-6.743	0.656	-1.147	1.00	0.00	N
ATOM	805	H	ILE	A	82	-7.254	0.500	-1.993	1.00	0.00	H
ATOM	806	CA	ILE	A	82	-5.534	1.439	-1.187	1.00	0.00	C
ATOM	807	CB	ILE	A	82	-5.028	1.636	-2.612	1.00	0.00	C
ATOM	808	CG2	ILE	A	82	-3.780	2.509	-2.605	1.00	0.00	C
ATOM	809	CG1	ILE	A	82	-4.718	0.281	-3.251	1.00	0.00	C
ATOM	810	CD1	ILE	A	82	-4.093	0.396	-4.642	1.00	0.00	C
ATOM	811	C	ILE	A	82	-5.759	2.746	-0.443	1.00	0.00	C
ATOM	812	O	ILE	A	82	-4.886	3.171	0.316	1.00	0.00	O
ATOM	813	N	ALA	A	83	-6.957	3.371	-0.598	1.00	0.00	N
ATOM	814	H	ALA	A	83	-7.632	2.973	-1.223	1.00	0.00	H
ATOM	815	CA	ALA	A	83	-7.292	4.618	0.056	1.00	0.00	C
ATOM	816	CB	ALA	A	83	-8.664	5.121	-0.392	1.00	0.00	C
ATOM	817	C	ALA	A	83	-7.304	4.460	1.552	1.00	0.00	C
ATOM	818	O	ALA	A	83	-6.815	5.338	2.267	1.00	0.00	O
ATOM	819	N	MET	A	84	-7.828	3.311	2.047	1.00	0.00	N
ATOM	820	H	MET	A	84	-8.216	2.668	1.386	1.00	0.00	H
ATOM	821	CA	MET	A	84	-7.892	3.010	3.454	1.00	0.00	C
ATOM	822	CB	MET	A	84	-8.845	1.868	3.789	1.00	0.00	C
ATOM	823	CG	MET	A	84	-10.299	2.327	3.682	1.00	0.00	C
ATOM	824	SD	MET	A	84	-10.625	3.916	4.478	1.00	0.00	S
ATOM	825	CE	MET	A	84	-9.929	3.599	6.109	1.00	0.00	C
ATOM	826	C	MET	A	84	-6.522	2.780	4.003	1.00	0.00	C

ATOM	827	O	MET	A	84	-6.190	3.386	5.021	1.00	0.00	O
ATOM	828	N	ALA	A	85	-5.632	2.142	3.197	1.00	0.00	N
ATOM	829	H	ALA	A	85	-5.982	1.741	2.347	1.00	0.00	H
ATOM	830	CA	ALA	A	85	-4.267	1.869	3.577	1.00	0.00	C
ATOM	831	CB	ALA	A	85	-3.560	1.013	2.525	1.00	0.00	C
ATOM	832	C	ALA	A	85	-3.533	3.172	3.747	1.00	0.00	C
ATOM	833	O	ALA	A	85	-2.726	3.292	4.663	1.00	0.00	O
ATOM	834	N	CYS	A	86	-3.827	4.180	2.888	1.00	0.00	N
ATOM	835	H	CYS	A	86	-4.458	3.976	2.137	1.00	0.00	H
ATOM	836	CA	CYS	A	86	-3.220	5.486	2.954	1.00	0.00	C
ATOM	837	CB	CYS	A	86	-3.576	6.262	1.699	1.00	0.00	C
ATOM	838	SG	CYS	A	86	-3.007	7.981	1.686	1.00	0.00	S
ATOM	839	C	CYS	A	86	-3.637	6.255	4.173	1.00	0.00	C
ATOM	840	O	CYS	A	86	-2.779	6.806	4.861	1.00	0.00	O
ATOM	841	N	LEU	A	87	-4.951	6.268	4.501	1.00	0.00	N
ATOM	842	H	LEU	A	87	-5.586	5.774	3.902	1.00	0.00	H
ATOM	843	CA	LEU	A	87	-5.459	7.011	5.632	1.00	0.00	C
ATOM	844	CB	LEU	A	87	-6.986	7.070	5.535	1.00	0.00	C
ATOM	845	CG	LEU	A	87	-7.403	7.779	4.244	1.00	0.00	C
ATOM	846	CD1	LEU	A	87	-8.822	7.452	3.804	1.00	0.00	C
ATOM	847	CD2	LEU	A	87	-7.120	9.274	4.295	1.00	0.00	C
ATOM	848	C	LEU	A	87	-4.956	6.470	6.937	1.00	0.00	C
ATOM	849	O	LEU	A	87	-4.490	7.228	7.790	1.00	0.00	O
ATOM	850	N	VAL	A	88	-4.983	5.127	7.070	1.00	0.00	N
ATOM	851	H	VAL	A	88	-5.365	4.611	6.301	1.00	0.00	H
ATOM	852	CA	VAL	A	88	-4.556	4.425	8.252	1.00	0.00	C
ATOM	853	CB	VAL	A	88	-5.130	2.988	8.309	1.00	0.00	C
ATOM	854	CG1	VAL	A	88	-4.440	1.974	7.392	1.00	0.00	C
ATOM	855	CG2	VAL	A	88	-5.184	2.484	9.749	1.00	0.00	C
ATOM	856	C	VAL	A	88	-3.051	4.510	8.332	1.00	0.00	C
ATOM	857	O	VAL	A	88	-2.499	4.701	9.415	1.00	0.00	O
ATOM	858	N	GLY	A	89	-2.375	4.454	7.161	1.00	0.00	N
ATOM	859	H	GLY	A	89	-2.896	4.297	6.322	1.00	0.00	H
ATOM	860	CA	GLY	A	89	-0.947	4.506	7.038	1.00	0.00	C
ATOM	861	C	GLY	A	89	-0.368	5.802	7.473	1.00	0.00	C
ATOM	862	O	GLY	A	89	0.678	5.799	8.116	1.00	0.00	O
ATOM	863	N	LEU	A	90	-1.042	6.931	7.158	1.00	0.00	N
ATOM	864	H	LEU	A	90	-1.864	6.855	6.593	1.00	0.00	H
ATOM	865	CA	LEU	A	90	-0.582	8.238	7.550	1.00	0.00	C
ATOM	866	CB	LEU	A	90	-1.478	9.314	6.937	1.00	0.00	C
ATOM	867	CG	LEU	A	90	-1.260	9.539	5.445	1.00	0.00	C
ATOM	868	CD1	LEU	A	90	-2.419	10.303	4.803	1.00	0.00	C
ATOM	869	CD2	LEU	A	90	0.080	10.219	5.191	1.00	0.00	C
ATOM	870	C	LEU	A	90	-0.639	8.387	9.035	1.00	0.00	C
ATOM	871	O	LEU	A	90	0.301	8.925	9.626	1.00	0.00	O
ATOM	872	N	MET	A	91	-1.715	7.845	9.663	1.00	0.00	N
ATOM	873	H	MET	A	91	-2.436	7.446	9.092	1.00	0.00	H
ATOM	874	CA	MET	A	91	-1.880	7.916	11.092	1.00	0.00	C
ATOM	875	CB	MET	A	91	-3.282	7.402	11.398	1.00	0.00	C
ATOM	876	CG	MET	A	91	-3.748	7.523	12.843	1.00	0.00	C
ATOM	877	SD	MET	A	91	-5.440	6.936	12.988	1.00	0.00	S
ATOM	878	CE	MET	A	91	-5.166	5.225	12.506	1.00	0.00	C
ATOM	879	C	MET	A	91	-0.808	7.110	11.760	1.00	0.00	C
ATOM	880	O	MET	A	91	-0.097	7.652	12.608	1.00	0.00	O
ATOM	881	N	TRP	A	92	-0.488	5.919	11.192	1.00	0.00	N
ATOM	882	H	TRP	A	92	-1.058	5.588	10.437	1.00	0.00	H
ATOM	883	CA	TRP	A	92	0.524	5.075	11.755	1.00	0.00	C
ATOM	884	CB	TRP	A	92	0.333	3.609	11.365	1.00	0.00	C
ATOM	885	CG	TRP	A	92	-0.840	3.085	12.160	1.00	0.00	C

ATOM	886	CD2	TRP	A	92	-0.935	2.921	13.593	1.00	0.00	C
ATOM	887	CE2	TRP	A	92	-2.255	2.437	13.875	1.00	0.00	C
ATOM	888	CE3	TRP	A	92	-0.026	3.147	14.651	1.00	0.00	C
ATOM	889	CD1	TRP	A	92	-2.088	2.692	11.658	1.00	0.00	C
ATOM	890	NE1	TRP	A	92	-2.925	2.313	12.660	1.00	0.00	N
ATOM	891	HE1	TRP	A	92	-3.868	2.074	12.538	1.00	0.00	H
ATOM	892	CZ2	TRP	A	92	-2.642	2.193	15.210	1.00	0.00	C
ATOM	893	CZ3	TRP	A	92	-0.423	2.897	15.981	1.00	0.00	C
ATOM	894	CH2	TRP	A	92	-1.724	2.425	16.256	1.00	0.00	C
ATOM	895	C	TRP	A	92	1.904	5.610	11.580	1.00	0.00	C
ATOM	896	O	TRP	A	92	2.726	5.385	12.462	1.00	0.00	O
ATOM	897	N	LEU	A	93	2.197	6.340	10.471	1.00	0.00	N
ATOM	898	H	LEU	A	93	1.503	6.464	9.760	1.00	0.00	H
ATOM	899	CA	LEU	A	93	3.503	6.927	10.270	1.00	0.00	C
ATOM	900	CB	LEU	A	93	3.605	7.616	8.911	1.00	0.00	C
ATOM	901	CG	LEU	A	93	3.886	6.645	7.770	1.00	0.00	C
ATOM	902	CD1	LEU	A	93	3.861	7.337	6.407	1.00	0.00	C
ATOM	903	CD2	LEU	A	93	5.188	5.882	8.008	1.00	0.00	C
ATOM	904	C	LEU	A	93	3.745	7.944	11.336	1.00	0.00	C
ATOM	905	O	LEU	A	93	4.815	7.945	11.941	1.00	0.00	O
ATOM	906	N	SER	A	94	2.718	8.769	11.644	1.00	0.00	N
ATOM	907	H	SER	A	94	1.867	8.725	11.121	1.00	0.00	H
ATOM	908	CA	SER	A	94	2.828	9.792	12.645	1.00	0.00	C
ATOM	909	CB	SER	A	94	1.593	10.661	12.475	1.00	0.00	C
ATOM	910	OG	SER	A	94	1.474	10.937	11.073	1.00	0.00	O
ATOM	911	HG	SER	A	94	0.877	10.296	10.697	1.00	0.00	H
ATOM	912	C	SER	A	94	3.046	9.199	14.012	1.00	0.00	C
ATOM	913	O	SER	A	94	3.920	9.674	14.734	1.00	0.00	O
ATOM	914	N	TYR	A	95	2.307	8.119	14.371	1.00	0.00	N
ATOM	915	H	TYR	A	95	1.648	7.751	13.711	1.00	0.00	H
ATOM	916	CA	TYR	A	95	2.444	7.508	15.672	1.00	0.00	C
ATOM	917	CB	TYR	A	95	1.238	6.610	15.969	1.00	0.00	C
ATOM	918	CG	TYR	A	95	0.047	7.430	16.419	1.00	0.00	C
ATOM	919	CD1	TYR	A	95	-0.666	8.204	15.481	1.00	0.00	C
ATOM	920	CE1	TYR	A	95	-1.767	8.962	15.910	1.00	0.00	C
ATOM	921	CD2	TYR	A	95	-0.322	7.391	17.779	1.00	0.00	C
ATOM	922	CE2	TYR	A	95	-1.429	8.143	18.209	1.00	0.00	C
ATOM	923	CZ	TYR	A	95	-2.137	8.919	17.268	1.00	0.00	C
ATOM	924	OH	TYR	A	95	-3.225	9.660	17.681	1.00	0.00	O
ATOM	925	HH	TYR	A	95	-3.538	9.350	18.520	1.00	0.00	H
ATOM	926	C	TYR	A	95	3.721	6.759	15.882	1.00	0.00	C
ATOM	927	O	TYR	A	95	4.383	6.986	16.888	1.00	0.00	O
ATOM	928	N	PHE	A	96	4.121	5.894	14.926	1.00	0.00	N
ATOM	929	H	PHE	A	96	3.593	5.830	14.079	1.00	0.00	H
ATOM	930	CA	PHE	A	96	5.322	5.102	15.035	1.00	0.00	C
ATOM	931	CB	PHE	A	96	5.385	4.041	13.937	1.00	0.00	C
ATOM	932	CG	PHE	A	96	4.643	2.804	14.378	1.00	0.00	C
ATOM	933	CD1	PHE	A	96	3.424	2.451	13.778	1.00	0.00	C
ATOM	934	CD2	PHE	A	96	5.180	1.997	15.386	1.00	0.00	C
ATOM	935	CE1	PHE	A	96	2.715	1.296	14.138	1.00	0.00	C
ATOM	936	CE2	PHE	A	96	4.471	0.844	15.748	1.00	0.00	C
ATOM	937	CZ	PHE	A	96	3.259	0.483	15.138	1.00	0.00	C
ATOM	938	C	PHE	A	96	6.579	5.907	15.025	1.00	0.00	C
ATOM	939	O	PHE	A	96	7.538	5.543	15.705	1.00	0.00	O
ATOM	940	N	ILE	A	97	6.614	6.996	14.229	1.00	0.00	N
ATOM	941	H	ILE	A	97	5.813	7.260	13.688	1.00	0.00	H
ATOM	942	CA	ILE	A	97	7.788	7.822	14.146	1.00	0.00	C
ATOM	943	CB	ILE	A	97	7.845	8.504	12.784	1.00	0.00	C
ATOM	944	CG2	ILE	A	97	8.954	9.553	12.677	1.00	0.00	C

ATOM	945	CG1	ILE	A	97	7.994	7.391	11.747	1.00	0.00	C
ATOM	946	CD1	ILE	A	97	7.700	7.795	10.307	1.00	0.00	C
ATOM	947	C	ILE	A	97	7.867	8.783	15.309	1.00	0.00	C
ATOM	948	O	ILE	A	97	8.924	8.883	15.937	1.00	0.00	O
ATOM	949	N	ALA	A	98	6.752	9.481	15.641	1.00	0.00	N
ATOM	950	H	ALA	A	98	5.895	9.320	15.149	1.00	0.00	H
ATOM	951	CA	ALA	A	98	6.759	10.442	16.717	1.00	0.00	C
ATOM	952	CB	ALA	A	98	5.642	11.476	16.566	1.00	0.00	C
ATOM	953	C	ALA	A	98	6.698	9.864	18.097	1.00	0.00	C
ATOM	954	O	ALA	A	98	7.518	10.241	18.936	1.00	0.00	O
ATOM	955	N	SER	A	99	5.750	8.928	18.364	1.00	0.00	N
ATOM	956	H	SER	A	99	5.259	8.509	17.605	1.00	0.00	H
ATOM	957	CA	SER	A	99	5.635	8.345	19.674	1.00	0.00	C
ATOM	958	CB	SER	A	99	4.211	7.844	19.842	1.00	0.00	C
ATOM	959	OG	SER	A	99	3.378	8.627	18.984	1.00	0.00	O
ATOM	960	HG	SER	A	99	3.538	8.274	18.114	1.00	0.00	H
ATOM	961	C	SER	A	99	6.675	7.277	19.785	1.00	0.00	C
ATOM	962	O	SER	A	99	6.581	6.188	19.215	1.00	0.00	O
ATOM	963	N	PHE	A	100	7.688	7.599	20.604	1.00	0.00	N
ATOM	964	H	PHE	A	100	7.723	8.562	20.874	1.00	0.00	H
ATOM	965	CA	PHE	A	100	8.825	6.761	20.858	1.00	0.00	C
ATOM	966	CB	PHE	A	100	9.928	7.566	21.540	1.00	0.00	C
ATOM	967	CG	PHE	A	100	10.272	8.739	20.653	1.00	0.00	C
ATOM	968	CD1	PHE	A	100	10.200	10.034	21.151	1.00	0.00	C
ATOM	969	CD2	PHE	A	100	10.650	8.520	19.333	1.00	0.00	C
ATOM	970	CE1	PHE	A	100	10.497	11.112	20.325	1.00	0.00	C
ATOM	971	CE2	PHE	A	100	10.947	9.598	18.508	1.00	0.00	C
ATOM	972	CZ	PHE	A	100	10.865	10.894	19.002	1.00	0.00	C
ATOM	973	C	PHE	A	100	8.483	5.524	21.623	1.00	0.00	C
ATOM	974	O	PHE	A	100	9.142	4.503	21.438	1.00	0.00	O
ATOM	975	N	ARG	A	101	7.409	5.584	22.450	1.00	0.00	N
ATOM	976	H	ARG	A	101	6.934	6.460	22.466	1.00	0.00	H
ATOM	977	CA	ARG	A	101	6.943	4.501	23.282	1.00	0.00	C
ATOM	978	CB	ARG	A	101	5.731	4.939	24.108	1.00	0.00	C
ATOM	979	CG	ARG	A	101	5.981	6.196	24.944	1.00	0.00	C
ATOM	980	CD	ARG	A	101	4.788	6.572	25.830	1.00	0.00	C
ATOM	981	NE	ARG	A	101	3.584	6.845	25.043	1.00	0.00	N
ATOM	982	HE	ARG	A	101	3.258	6.091	24.463	1.00	0.00	H
ATOM	983	CZ	ARG	A	101	2.953	8.052	25.185	1.00	0.00	C
ATOM	984	NH1	ARG	A	101	3.466	8.970	26.039	1.00	0.00	N
ATOM	985	HH11	ARG	A	101	4.293	8.783	26.578	1.00	0.00	H
ATOM	986	HH12	ARG	A	101	3.050	9.874	26.181	1.00	0.00	H
ATOM	987	NH2	ARG	A	101	1.830	8.308	24.472	1.00	0.00	N
ATOM	988	HH21	ARG	A	101	1.440	7.630	23.841	1.00	0.00	H
ATOM	989	HH22	ARG	A	101	1.332	9.178	24.537	1.00	0.00	H
ATOM	990	C	ARG	A	101	6.597	3.263	22.503	1.00	0.00	C
ATOM	991	O	ARG	A	101	6.748	2.161	23.032	1.00	0.00	O
ATOM	992	N	LEU	A	102	6.148	3.411	21.230	1.00	0.00	N
ATOM	993	H	LEU	A	102	6.162	4.313	20.797	1.00	0.00	H
ATOM	994	CA	LEU	A	102	5.797	2.284	20.401	1.00	0.00	C
ATOM	995	CB	LEU	A	102	5.097	2.747	19.124	1.00	0.00	C
ATOM	996	CG	LEU	A	102	3.759	3.439	19.400	1.00	0.00	C
ATOM	997	CD1	LEU	A	102	3.113	3.941	18.111	1.00	0.00	C
ATOM	998	CD2	LEU	A	102	2.794	2.551	20.190	1.00	0.00	C
ATOM	999	C	LEU	A	102	6.977	1.408	20.088	1.00	0.00	C
ATOM	1000	O	LEU	A	102	6.818	0.192	19.989	1.00	0.00	O
ATOM	1001	N	PHE	A	103	8.171	2.014	19.896	1.00	0.00	N
ATOM	1002	H	PHE	A	103	8.239	2.997	20.071	1.00	0.00	H
ATOM	1003	CA	PHE	A	103	9.391	1.297	19.620	1.00	0.00	C

```
ATOM   1004  CB  PHE A 103      10.406   2.242  18.970  1.00  0.00           C
ATOM   1005  CG  PHE A 103      11.693   1.519  18.650  1.00  0.00           C
ATOM   1006  CD1 PHE A 103      11.784   0.730  17.510  1.00  0.00           C
ATOM   1007  CD2 PHE A 103      12.795   1.647  19.487  1.00  0.00           C
ATOM   1008  CE1 PHE A 103      12.969   0.070  17.208  1.00  0.00           C
ATOM   1009  CE2 PHE A 103      13.980   0.985  19.190  1.00  0.00           C
ATOM   1010  CZ  PHE A 103      14.067   0.196  18.049  1.00  0.00           C
ATOM   1011  C   PHE A 103       9.916   0.777  20.934  1.00  0.00           C
ATOM   1012  O   PHE A 103      10.285  -0.400  21.057  1.00  0.00           O
ATOM   1013  N   ALA A 104       9.896   1.694  21.946  1.00  0.00           N
ATOM   1014  H   ALA A 104       9.606   2.615  21.687  1.00  0.00           H
ATOM   1015  CA  ALA A 104      10.357   1.550  23.297  1.00  0.00           C
ATOM   1016  CB  ALA A 104       9.459   0.579  24.072  1.00  0.00           C
ATOM   1017  C   ALA A 104      11.773   1.067  23.143  1.00  0.00           C
ATOM   1018  O   ALA A 104      12.592   1.745  22.515  1.00  0.00           O
ATOM   1019  N   ARG A 105      12.112  -0.069  23.772  1.00  0.00           N
ATOM   1020  H   ARG A 105      11.479  -0.569  24.361  1.00  0.00           H
ATOM   1021  CA  ARG A 105      13.392  -0.694  23.641  1.00  0.00           C
ATOM   1022  CB  ARG A 105      14.197  -0.613  24.938  1.00  0.00           C
ATOM   1023  CG  ARG A 105      14.696   0.807  25.210  1.00  0.00           C
ATOM   1024  CD  ARG A 105      15.512   1.347  24.032  1.00  0.00           C
ATOM   1025  NE  ARG A 105      16.010   2.697  24.294  1.00  0.00           N
ATOM   1026  HE  ARG A 105      16.727   2.754  24.997  1.00  0.00           H
ATOM   1027  CZ  ARG A 105      15.513   3.753  23.581  1.00  0.00           C
ATOM   1028  NH1 ARG A 105      14.515   3.555  22.691  1.00  0.00           N
ATOM   1029 HH11 ARG A 105      14.068   2.653  22.574  1.00  0.00           H
ATOM   1030 HH12 ARG A 105      14.141   4.278  22.105  1.00  0.00           H
ATOM   1031  NH2 ARG A 105      16.031   4.988  23.778  1.00  0.00           N
ATOM   1032 HH21 ARG A 105      16.774   5.145  24.436  1.00  0.00           H
ATOM   1033 HH22 ARG A 105      15.701   5.798  23.284  1.00  0.00           H
ATOM   1034  C   ARG A 105      13.136  -2.124  23.228  1.00  0.00           C
ATOM   1035  O   ARG A 105      14.040  -2.804  22.746  1.00  0.00           O
ATOM   1036  N   THR A 106      11.860  -2.567  23.341  1.00  0.00           N
ATOM   1037  H   THR A 106      11.120  -1.953  23.598  1.00  0.00           H
ATOM   1038  CA  THR A 106      11.378  -3.889  23.079  1.00  0.00           C
ATOM   1039  CB  THR A 106       9.931  -3.834  23.526  1.00  0.00           C
ATOM   1040  OG1 THR A 106       9.832  -2.821  24.543  1.00  0.00           O
ATOM   1041  HG1 THR A 106       8.978  -2.936  24.947  1.00  0.00           H
ATOM   1042  CG2 THR A 106       9.412  -5.187  24.016  1.00  0.00           C
ATOM   1043  C   THR A 106      11.577  -4.334  21.658  1.00  0.00           C
ATOM   1044  O   THR A 106      11.992  -5.475  21.454  1.00  0.00           O
ATOM   1045  N   ARG A 107      11.367  -3.446  20.659  1.00  0.00           N
ATOM   1046  H   ARG A 107      11.106  -2.504  20.886  1.00  0.00           H
ATOM   1047  CA  ARG A 107      11.494  -3.839  19.275  1.00  0.00           C
ATOM   1048  CB  ARG A 107      10.952  -2.777  18.340  1.00  0.00           C
ATOM   1049  CG  ARG A 107       9.464  -2.607  18.594  1.00  0.00           C
ATOM   1050  CD  ARG A 107       8.827  -1.701  17.555  1.00  0.00           C
ATOM   1051  NE  ARG A 107       7.403  -1.574  17.816  1.00  0.00           N
ATOM   1052  HE  ARG A 107       7.155  -0.913  18.537  1.00  0.00           H
ATOM   1053  CZ  ARG A 107       6.539  -2.410  17.189  1.00  0.00           C
ATOM   1054  NH1 ARG A 107       6.994  -3.302  16.276  1.00  0.00           N
ATOM   1055 HH11 ARG A 107       7.979  -3.379  16.055  1.00  0.00           H
ATOM   1056 HH12 ARG A 107       6.397  -3.936  15.780  1.00  0.00           H
ATOM   1057  NH2 ARG A 107       5.233  -2.332  17.499  1.00  0.00           N
ATOM   1058 HH21 ARG A 107       4.904  -1.673  18.186  1.00  0.00           H
ATOM   1059 HH22 ARG A 107       4.557  -2.924  17.066  1.00  0.00           H
ATOM   1060  C   ARG A 107      12.883  -4.258  18.866  1.00  0.00           C
ATOM   1061  O   ARG A 107      13.033  -5.181  18.064  1.00  0.00           O
ATOM   1062  N   SER A 108      13.922  -3.620  19.450  1.00  0.00           N
```

```
ATOM   1063  H   SER A 108      13.700  -2.944  20.147  1.00  0.00           H
ATOM   1064  CA  SER A 108      15.308  -3.909  19.169  1.00  0.00           C
ATOM   1065  CB  SER A 108      16.076  -2.703  19.690  1.00  0.00           C
ATOM   1066  OG  SER A 108      15.140  -1.867  20.392  1.00  0.00           O
ATOM   1067  HG  SER A 108      15.114  -2.208  21.286  1.00  0.00           H
ATOM   1068  C   SER A 108      15.774  -5.229  19.744  1.00  0.00           C
ATOM   1069  O   SER A 108      16.759  -5.803  19.278  1.00  0.00           O
ATOM   1070  N   MET A 109      15.062  -5.724  20.782  1.00  0.00           N
ATOM   1071  H   MET A 109      14.217  -5.258  21.044  1.00  0.00           H
ATOM   1072  CA  MET A 109      15.350  -6.946  21.490  1.00  0.00           C
ATOM   1073  CB  MET A 109      14.558  -7.012  22.795  1.00  0.00           C
ATOM   1074  CG  MET A 109      14.899  -5.867  23.749  1.00  0.00           C
ATOM   1075  SD  MET A 109      13.881  -5.886  25.233  1.00  0.00           S
ATOM   1076  CE  MET A 109      14.300  -7.543  25.796  1.00  0.00           C
ATOM   1077  C   MET A 109      15.163  -8.217  20.709  1.00  0.00           C
ATOM   1078  O   MET A 109      15.778  -9.224  21.072  1.00  0.00           O
ATOM   1079  N   TRP A 110      14.346  -8.202  19.617  1.00  0.00           N
ATOM   1080  H   TRP A 110      13.995  -7.315  19.322  1.00  0.00           H
ATOM   1081  CA  TRP A 110      14.063  -9.363  18.793  1.00  0.00           C
ATOM   1082  CB  TRP A 110      13.139  -8.835  17.691  1.00  0.00           C
ATOM   1083  CG  TRP A 110      12.723  -9.848  16.652  1.00  0.00           C
ATOM   1084  CD2 TRP A 110      11.420 -10.442  16.498  1.00  0.00           C
ATOM   1085  CE2 TRP A 110      11.461 -11.272  15.358  1.00  0.00           C
ATOM   1086  CE3 TRP A 110      10.243 -10.330  17.228  1.00  0.00           C
ATOM   1087  CD1 TRP A 110      13.486 -10.361  15.591  1.00  0.00           C
ATOM   1088  NE1 TRP A 110      12.745 -11.204  14.823  1.00  0.00           N
ATOM   1089  HE1 TRP A 110      13.067 -11.680  14.024  1.00  0.00           H
ATOM   1090  CZ2 TRP A 110      10.318 -11.966  14.984  1.00  0.00           C
ATOM   1091  CZ3 TRP A 110       9.108 -11.031  16.842  1.00  0.00           C
ATOM   1092  CH2 TRP A 110       9.146 -11.848  15.720  1.00  0.00           C
ATOM   1093  C   TRP A 110      15.377  -9.869  18.242  1.00  0.00           C
ATOM   1094  O   TRP A 110      16.097  -9.139  17.555  1.00  0.00           O
ATOM   1095  N   SER A 111      15.724 -11.134  18.600  1.00  0.00           N
ATOM   1096  H   SER A 111      15.145 -11.647  19.233  1.00  0.00           H
ATOM   1097  CA  SER A 111      16.965 -11.716  18.155  1.00  0.00           C
ATOM   1098  CB  SER A 111      17.900 -11.785  19.360  1.00  0.00           C
ATOM   1099  OG  SER A 111      17.974 -10.500  19.985  1.00  0.00           O
ATOM   1100  HG  SER A 111      17.100 -10.251  20.273  1.00  0.00           H
ATOM   1101  C   SER A 111      16.787 -13.037  17.449  1.00  0.00           C
ATOM   1102  O   SER A 111      17.117 -13.164  16.271  1.00  0.00           O
ATOM   1103  N   PHE A 112      16.256 -14.052  18.175  1.00  0.00           N
ATOM   1104  H   PHE A 112      15.929 -13.855  19.102  1.00  0.00           H
ATOM   1105  CA  PHE A 112      15.988 -15.401  17.707  1.00  0.00           C
ATOM   1106  CB  PHE A 112      16.841 -16.410  18.473  1.00  0.00           C
ATOM   1107  CG  PHE A 112      18.297 -16.101  18.227  1.00  0.00           C
ATOM   1108  CD1 PHE A 112      19.114 -15.715  19.283  1.00  0.00           C
ATOM   1109  CD2 PHE A 112      18.816 -16.192  16.941  1.00  0.00           C
ATOM   1110  CE1 PHE A 112      20.449 -15.410  19.050  1.00  0.00           C
ATOM   1111  CE2 PHE A 112      20.152 -15.888  16.709  1.00  0.00           C
ATOM   1112  CZ  PHE A 112      20.967 -15.493  17.763  1.00  0.00           C
ATOM   1113  C   PHE A 112      14.520 -15.613  17.966  1.00  0.00           C
ATOM   1114  O   PHE A 112      14.057 -16.682  18.370  1.00  0.00           O
ATOM   1115  N   ASN A 113      13.755 -14.532  17.760  1.00  0.00           N
ATOM   1116  H   ASN A 113      14.177 -13.740  17.324  1.00  0.00           H
ATOM   1117  CA  ASN A 113      12.368 -14.494  18.029  1.00  0.00           C
ATOM   1118  CB  ASN A 113      11.936 -13.152  18.619  1.00  0.00           C
ATOM   1119  CG  ASN A 113      12.619 -12.932  19.944  1.00  0.00           C
ATOM   1120  OD1 ASN A 113      13.830 -12.732  20.003  1.00  0.00           O
ATOM   1121  ND2 ASN A 113      11.786 -12.963  20.998  1.00  0.00           N
```

```
ATOM   1122 HD21 ASN A 113      10.812 -13.153  20.867  1.00  0.00           H
ATOM   1123 HD22 ASN A 113      12.101 -12.812  21.934  1.00  0.00           H
ATOM   1124  C   ASN A 113      11.340 -14.956  17.066  1.00  0.00           C
ATOM   1125  O   ASN A 113      10.257 -15.129  17.599  1.00  0.00           O
ATOM   1126  N   PRO A 114      11.389 -15.099  15.749  1.00  0.00           N
ATOM   1127  CD  PRO A 114      12.512 -14.754  14.885  1.00  0.00           C
ATOM   1128  CA  PRO A 114      10.253 -15.653  15.047  1.00  0.00           C
ATOM   1129  CB  PRO A 114      10.617 -15.427  13.582  1.00  0.00           C
ATOM   1130  CG  PRO A 114      12.139 -15.295  13.524  1.00  0.00           C
ATOM   1131  C   PRO A 114      10.124 -17.100  15.500  1.00  0.00           C
ATOM   1132  O   PRO A 114      11.067 -17.881  15.350  1.00  0.00           O
ATOM   1133  N   GLU A 115       8.949 -17.444  16.079  1.00  0.00           N
ATOM   1134  H   GLU A 115       8.155 -16.875  15.841  1.00  0.00           H
ATOM   1135  CA  GLU A 115       8.755 -18.625  16.894  1.00  0.00           C
ATOM   1136  CB  GLU A 115       7.464 -18.585  17.734  1.00  0.00           C
ATOM   1137  CG  GLU A 115       6.373 -17.577  17.344  1.00  0.00           C
ATOM   1138  CD  GLU A 115       5.938 -17.827  15.914  1.00  0.00           C
ATOM   1139  OE1 GLU A 115       6.229 -17.003  15.057  1.00  0.00           O
ATOM   1140  OE2 GLU A 115       5.379 -18.876  15.625  1.00  0.00           O
ATOM   1141  C   GLU A 115       8.803 -19.807  15.972  1.00  0.00           C
ATOM   1142  O   GLU A 115       9.437 -20.826  16.254  1.00  0.00           O
ATOM   1143  N   THR A 116       8.163 -19.612  14.800  1.00  0.00           N
ATOM   1144  H   THR A 116       7.599 -18.787  14.722  1.00  0.00           H
ATOM   1145  CA  THR A 116       8.071 -20.550  13.729  1.00  0.00           C
ATOM   1146  CB  THR A 116       7.104 -19.940  12.727  1.00  0.00           C
ATOM   1147  OG1 THR A 116       7.069 -18.526  12.938  1.00  0.00           O
ATOM   1148  HG1 THR A 116       6.393 -18.341  13.601  1.00  0.00           H
ATOM   1149  CG2 THR A 116       5.702 -20.549  12.784  1.00  0.00           C
ATOM   1150  C   THR A 116       9.430 -20.784  13.126  1.00  0.00           C
ATOM   1151  O   THR A 116       9.725 -21.915  12.759  1.00  0.00           O
ATOM   1152  N   ASN A 117      10.298 -19.738  13.040  1.00  0.00           N
ATOM   1153  H   ASN A 117       9.981 -18.859  13.399  1.00  0.00           H
ATOM   1154  CA  ASN A 117      11.637 -19.858  12.491  1.00  0.00           C
ATOM   1155  CB  ASN A 117      12.388 -18.555  12.323  1.00  0.00           C
ATOM   1156  CG  ASN A 117      13.847 -18.895  12.090  1.00  0.00           C
ATOM   1157  OD1 ASN A 117      14.229 -19.490  11.088  1.00  0.00           O
ATOM   1158  ND2 ASN A 117      14.651 -18.461  13.080  1.00  0.00           N
ATOM   1159 HD21 ASN A 117      14.271 -17.981  13.873  1.00  0.00           H
ATOM   1160 HD22 ASN A 117      15.638 -18.615  13.046  1.00  0.00           H
ATOM   1161  C   ASN A 117      12.476 -20.765  13.332  1.00  0.00           C
ATOM   1162  O   ASN A 117      13.212 -21.583  12.786  1.00  0.00           O
ATOM   1163  N   ILE A 118      12.359 -20.647  14.673  1.00  0.00           N
ATOM   1164  H   ILE A 118      11.689 -19.998  15.042  1.00  0.00           H
ATOM   1165  CA  ILE A 118      13.130 -21.449  15.587  1.00  0.00           C
ATOM   1166  CB  ILE A 118      13.015 -20.888  17.001  1.00  0.00           C
ATOM   1167  CG2 ILE A 118      13.719 -21.770  18.034  1.00  0.00           C
ATOM   1168  CG1 ILE A 118      13.567 -19.467  17.005  1.00  0.00           C
ATOM   1169  CD1 ILE A 118      15.040 -19.426  16.592  1.00  0.00           C
ATOM   1170  C   ILE A 118      12.703 -22.894  15.531  1.00  0.00           C
ATOM   1171  O   ILE A 118      13.564 -23.773  15.478  1.00  0.00           O
ATOM   1172  N   LEU A 119      11.376 -23.161  15.490  1.00  0.00           N
ATOM   1173  H   LEU A 119      10.731 -22.394  15.538  1.00  0.00           H
ATOM   1174  CA  LEU A 119      10.863 -24.509  15.468  1.00  0.00           C
ATOM   1175  CB  LEU A 119       9.360 -24.475  15.743  1.00  0.00           C
ATOM   1176  CG  LEU A 119       8.722 -25.863  15.816  1.00  0.00           C
ATOM   1177  CD1 LEU A 119       9.293 -26.695  16.966  1.00  0.00           C
ATOM   1178  CD2 LEU A 119       7.196 -25.779  15.866  1.00  0.00           C
ATOM   1179  C   LEU A 119      11.142 -25.223  14.163  1.00  0.00           C
ATOM   1180  O   LEU A 119      11.663 -26.340  14.164  1.00  0.00           O
```

```
ATOM   1181  N   LEU A 120      10.825 -24.563  13.034  1.00  0.00           N
ATOM   1182  H   LEU A 120      10.474 -23.631  13.136  1.00  0.00           H
ATOM   1183  CA  LEU A 120      10.976 -25.082  11.701  1.00  0.00           C
ATOM   1184  CB  LEU A 120       9.942 -24.440  10.780  1.00  0.00           C
ATOM   1185  CG  LEU A 120       8.504 -24.614  11.271  1.00  0.00           C
ATOM   1186  CD1 LEU A 120       7.524 -23.788  10.437  1.00  0.00           C
ATOM   1187  CD2 LEU A 120       8.100 -26.087  11.352  1.00  0.00           C
ATOM   1188  C   LEU A 120      12.349 -25.037  11.092  1.00  0.00           C
ATOM   1189  O   LEU A 120      12.651 -25.878  10.242  1.00  0.00           O
ATOM   1190  N   ASN A 121      13.208 -24.071  11.522  1.00  0.00           N
ATOM   1191  H   ASN A 121      12.865 -23.405  12.188  1.00  0.00           H
ATOM   1192  CA  ASN A 121      14.545 -23.817  10.998  1.00  0.00           C
ATOM   1193  CB  ASN A 121      15.477 -25.013  11.185  1.00  0.00           C
ATOM   1194  CG  ASN A 121      16.487 -24.702  12.271  1.00  0.00           C
ATOM   1195  OD1 ASN A 121      17.689 -24.742  12.042  1.00  0.00           O
ATOM   1196  ND2 ASN A 121      15.944 -24.419  13.475  1.00  0.00           N
ATOM   1197 HD21 ASN A 121      16.526 -24.222  14.264  1.00  0.00           H
ATOM   1198 HD22 ASN A 121      14.961 -24.385  13.677  1.00  0.00           H
ATOM   1199  C   ASN A 121      14.395 -23.403   9.546  1.00  0.00           C
ATOM   1200  O   ASN A 121      15.141 -23.826   8.657  1.00  0.00           O
ATOM   1201  N   VAL A 122      13.371 -22.544   9.294  1.00  0.00           N
ATOM   1202  H   VAL A 122      12.942 -22.076  10.069  1.00  0.00           H
ATOM   1203  CA  VAL A 122      13.027 -22.061   7.980  1.00  0.00           C
ATOM   1204  CB  VAL A 122      11.578 -22.451   7.682  1.00  0.00           C
ATOM   1205  CG1 VAL A 122      11.036 -21.801   6.420  1.00  0.00           C
ATOM   1206  CG2 VAL A 122      11.486 -23.972   7.570  1.00  0.00           C
ATOM   1207  C   VAL A 122      13.294 -20.577   7.813  1.00  0.00           C
ATOM   1208  O   VAL A 122      12.619 -19.759   8.441  1.00  0.00           O
ATOM   1209  N   PRO A 123      14.219 -20.204   6.917  1.00  0.00           N
ATOM   1210  CD  PRO A 123      15.015 -21.173   6.173  1.00  0.00           C
ATOM   1211  CA  PRO A 123      14.613 -18.834   6.631  1.00  0.00           C
ATOM   1212  CB  PRO A 123      15.735 -18.994   5.601  1.00  0.00           C
ATOM   1213  CG  PRO A 123      16.277 -20.404   5.813  1.00  0.00           C
ATOM   1214  C   PRO A 123      13.499 -17.956   6.134  1.00  0.00           C
ATOM   1215  O   PRO A 123      13.632 -16.739   6.242  1.00  0.00           O
ATOM   1216  N   LEU A 124      12.387 -18.545   5.635  1.00  0.00           N
ATOM   1217  H   LEU A 124      12.388 -19.539   5.551  1.00  0.00           H
ATOM   1218  CA  LEU A 124      11.211 -17.841   5.183  1.00  0.00           C
ATOM   1219  CB  LEU A 124      10.187 -18.809   4.592  1.00  0.00           C
ATOM   1220  CG  LEU A 124      10.667 -19.573   3.355  1.00  0.00           C
ATOM   1221  CD1 LEU A 124       9.722 -20.718   2.991  1.00  0.00           C
ATOM   1222  CD2 LEU A 124      10.896 -18.652   2.161  1.00  0.00           C
ATOM   1223  C   LEU A 124      10.585 -17.050   6.310  1.00  0.00           C
ATOM   1224  O   LEU A 124       9.838 -16.099   6.067  1.00  0.00           O
ATOM   1225  N   HIS A 125      10.855 -17.466   7.570  1.00  0.00           N
ATOM   1226  H   HIS A 125      11.489 -18.223   7.734  1.00  0.00           H
ATOM   1227  CA  HIS A 125      10.343 -16.825   8.740  1.00  0.00           C
ATOM   1228  CB  HIS A 125       9.658 -17.807   9.694  1.00  0.00           C
ATOM   1229  CG  HIS A 125       8.341 -18.257   9.107  1.00  0.00           C
ATOM   1230  ND1 HIS A 125       7.168 -17.623   9.303  1.00  0.00           N
ATOM   1231  HD1 HIS A 125       6.995 -16.818   9.849  1.00  0.00           H
ATOM   1232  CD2 HIS A 125       8.120 -19.366   8.285  1.00  0.00           C
ATOM   1233  NE2 HIS A 125       6.800 -19.396   7.984  1.00  0.00           N
ATOM   1234  CE1 HIS A 125       6.214 -18.322   8.611  1.00  0.00           C
ATOM   1235  C   HIS A 125      11.411 -16.027   9.444  1.00  0.00           C
ATOM   1236  O   HIS A 125      11.179 -15.561  10.555  1.00  0.00           O
ATOM   1237  N   GLY A 126      12.608 -15.843   8.832  1.00  0.00           N
ATOM   1238  H   GLY A 126      12.679 -16.080   7.864  1.00  0.00           H
ATOM   1239  CA  GLY A 126      13.710 -15.095   9.398  1.00  0.00           C
```

```
ATOM   1240  C   GLY A 126      13.415 -13.619   9.363  1.00  0.00           C
ATOM   1241  O   GLY A 126      12.470 -13.178   8.706  1.00  0.00           O
ATOM   1242  N   THR A 127      14.245 -12.813  10.072  1.00  0.00           N
ATOM   1243  H   THR A 127      14.988 -13.176  10.630  1.00  0.00           H
ATOM   1244  CA  THR A 127      14.052 -11.385  10.145  1.00  0.00           C
ATOM   1245  CB  THR A 127      14.949 -10.936  11.276  1.00  0.00           C
ATOM   1246  OG1 THR A 127      14.988 -12.001  12.242  1.00  0.00           O
ATOM   1247  HG1 THR A 127      15.708 -11.797  12.831  1.00  0.00           H
ATOM   1248  CG2 THR A 127      14.452  -9.634  11.911  1.00  0.00           C
ATOM   1249  C   THR A 127      14.290 -10.762   8.796  1.00  0.00           C
ATOM   1250  O   THR A 127      15.367 -10.849   8.195  1.00  0.00           O
ATOM   1251  N   ILE A 128      13.219 -10.085   8.333  1.00  0.00           N
ATOM   1252  H   ILE A 128      12.387 -10.151   8.882  1.00  0.00           H
ATOM   1253  CA  ILE A 128      13.171  -9.428   7.052  1.00  0.00           C
ATOM   1254  CB  ILE A 128      11.721  -9.195   6.620  1.00  0.00           C
ATOM   1255  CG2 ILE A 128      11.598  -8.269   5.408  1.00  0.00           C
ATOM   1256  CG1 ILE A 128      11.092 -10.559   6.331  1.00  0.00           C
ATOM   1257  CD1 ILE A 128       9.704 -10.454   5.702  1.00  0.00           C
ATOM   1258  C   ILE A 128      14.010  -8.179   7.042  1.00  0.00           C
ATOM   1259  O   ILE A 128      14.657  -7.900   6.032  1.00  0.00           O
ATOM   1260  N   LEU A 129      14.069  -7.428   8.172  1.00  0.00           N
ATOM   1261  H   LEU A 129      13.566  -7.724   8.980  1.00  0.00           H
ATOM   1262  CA  LEU A 129      14.860  -6.217   8.227  1.00  0.00           C
ATOM   1263  CB  LEU A 129      14.620  -5.502   9.554  1.00  0.00           C
ATOM   1264  CG  LEU A 129      15.480  -4.247   9.701  1.00  0.00           C
ATOM   1265  CD1 LEU A 129      15.034  -3.130   8.757  1.00  0.00           C
ATOM   1266  CD2 LEU A 129      15.583  -3.795  11.153  1.00  0.00           C
ATOM   1267  C   LEU A 129      16.340  -6.525   8.075  1.00  0.00           C
ATOM   1268  O   LEU A 129      17.075  -5.728   7.493  1.00  0.00           O
ATOM   1269  N   THR A 130      16.792  -7.692   8.587  1.00  0.00           N
ATOM   1270  H   THR A 130      16.172  -8.331   9.039  1.00  0.00           H
ATOM   1271  CA  THR A 130      18.164  -8.146   8.540  1.00  0.00           C
ATOM   1272  CB  THR A 130      18.165  -9.255   9.556  1.00  0.00           C
ATOM   1273  OG1 THR A 130      17.218  -8.857  10.559  1.00  0.00           O
ATOM   1274  HG1 THR A 130      17.667  -8.193  11.072  1.00  0.00           H
ATOM   1275  CG2 THR A 130      19.541  -9.533  10.159  1.00  0.00           C
ATOM   1276  C   THR A 130      18.571  -8.550   7.132  1.00  0.00           C
ATOM   1277  O   THR A 130      19.719  -8.324   6.741  1.00  0.00           O
ATOM   1278  N   ARG A 131      17.621  -9.096   6.325  1.00  0.00           N
ATOM   1279  H   ARG A 131      16.709  -9.223   6.714  1.00  0.00           H
ATOM   1280  CA  ARG A 131      17.841  -9.570   4.972  1.00  0.00           C
ATOM   1281  CB  ARG A 131      16.575 -10.055   4.250  1.00  0.00           C
ATOM   1282  CG  ARG A 131      16.964 -10.515   2.834  1.00  0.00           C
ATOM   1283  CD  ARG A 131      15.849 -10.640   1.799  1.00  0.00           C
ATOM   1284  NE  ARG A 131      15.080  -9.405   1.680  1.00  0.00           N
ATOM   1285  HE  ARG A 131      15.544  -8.656   1.194  1.00  0.00           H
ATOM   1286  CZ  ARG A 131      13.831  -9.382   2.231  1.00  0.00           C
ATOM   1287  NH1 ARG A 131      13.360 -10.497   2.839  1.00  0.00           N
ATOM   1288 HH11 ARG A 131      13.900 -11.356   2.854  1.00  0.00           H
ATOM   1289 HH12 ARG A 131      12.474 -10.537   3.303  1.00  0.00           H
ATOM   1290  NH2 ARG A 131      13.097  -8.247   2.163  1.00  0.00           N
ATOM   1291 HH21 ARG A 131      13.456  -7.425   1.709  1.00  0.00           H
ATOM   1292 HH22 ARG A 131      12.176  -8.167   2.554  1.00  0.00           H
ATOM   1293  C   ARG A 131      18.615  -8.655   4.037  1.00  0.00           C
ATOM   1294  O   ARG A 131      19.546  -9.214   3.454  1.00  0.00           O
ATOM   1295  N   PRO A 132      18.387  -7.346   3.830  1.00  0.00           N
ATOM   1296  CD  PRO A 132      17.319  -6.555   4.441  1.00  0.00           C
ATOM   1297  CA  PRO A 132      19.169  -6.539   2.912  1.00  0.00           C
ATOM   1298  CB  PRO A 132      18.394  -5.220   2.827  1.00  0.00           C
```

ATOM	1299	CG	PRO	A	132	17.688	-5.106	4.170	1.00	0.00	C
ATOM	1300	C	PRO	A	132	20.616	-6.352	3.277	1.00	0.00	C
ATOM	1301	O	PRO	A	132	21.401	-6.079	2.368	1.00	0.00	O
ATOM	1302	N	LEU	A	133	20.984	-6.480	4.576	1.00	0.00	N
ATOM	1303	H	LEU	A	133	20.311	-6.792	5.246	1.00	0.00	H
ATOM	1304	CA	LEU	A	133	22.360	-6.387	5.003	1.00	0.00	C
ATOM	1305	CB	LEU	A	133	22.415	-6.255	6.524	1.00	0.00	C
ATOM	1306	CG	LEU	A	133	21.515	-5.134	7.046	1.00	0.00	C
ATOM	1307	CD1	LEU	A	133	21.349	-5.201	8.565	1.00	0.00	C
ATOM	1308	CD2	LEU	A	133	21.977	-3.755	6.571	1.00	0.00	C
ATOM	1309	C	LEU	A	133	23.104	-7.614	4.552	1.00	0.00	C
ATOM	1310	O	LEU	A	133	24.224	-7.520	4.048	1.00	0.00	O
ATOM	1311	N	LEU	A	134	22.454	-8.787	4.716	1.00	0.00	N
ATOM	1312	H	LEU	A	134	21.510	-8.741	5.042	1.00	0.00	H
ATOM	1313	CA	LEU	A	134	22.970	-10.082	4.366	1.00	0.00	C
ATOM	1314	CB	LEU	A	134	22.330	-11.146	5.254	1.00	0.00	C
ATOM	1315	CG	LEU	A	134	22.364	-10.806	6.743	1.00	0.00	C
ATOM	1316	CD1	LEU	A	134	21.501	-11.773	7.553	1.00	0.00	C
ATOM	1317	CD2	LEU	A	134	23.795	-10.719	7.274	1.00	0.00	C
ATOM	1318	C	LEU	A	134	22.931	-10.359	2.890	1.00	0.00	C
ATOM	1319	O	LEU	A	134	23.780	-11.095	2.387	1.00	0.00	O
ATOM	1320	N	GLU	A	135	21.940	-9.756	2.176	1.00	0.00	N
ATOM	1321	H	GLU	A	135	21.314	-9.174	2.688	1.00	0.00	H
ATOM	1322	CA	GLU	A	135	21.648	-9.925	0.764	1.00	0.00	C
ATOM	1323	CB	GLU	A	135	22.773	-9.442	-0.159	1.00	0.00	C
ATOM	1324	CG	GLU	A	135	22.979	-7.925	-0.102	1.00	0.00	C
ATOM	1325	CD	GLU	A	135	23.862	-7.475	-1.253	1.00	0.00	C
ATOM	1326	OE1	GLU	A	135	23.348	-6.832	-2.168	1.00	0.00	O
ATOM	1327	OE2	GLU	A	135	25.059	-7.762	-1.233	1.00	0.00	O
ATOM	1328	C	GLU	A	135	21.312	-11.388	0.543	1.00	0.00	C
ATOM	1329	O	GLU	A	135	21.689	-12.008	-0.455	1.00	0.00	O
ATOM	1330	N	SER	A	136	20.569	-11.958	1.524	1.00	0.00	N
ATOM	1331	H	SER	A	136	20.181	-11.378	2.242	1.00	0.00	H
ATOM	1332	CA	SER	A	136	20.160	-13.332	1.542	1.00	0.00	C
ATOM	1333	CB	SER	A	136	19.691	-13.604	2.942	1.00	0.00	C
ATOM	1334	OG	SER	A	136	20.381	-12.660	3.743	1.00	0.00	O
ATOM	1335	HG	SER	A	136	21.297	-12.760	3.503	1.00	0.00	H
ATOM	1336	C	SER	A	136	19.112	-13.649	0.520	1.00	0.00	C
ATOM	1337	O	SER	A	136	18.130	-12.926	0.338	1.00	0.00	O
ATOM	1338	N	GLU	A	137	19.368	-14.764	-0.190	1.00	0.00	N
ATOM	1339	H	GLU	A	137	20.266	-15.187	-0.090	1.00	0.00	H
ATOM	1340	CA	GLU	A	137	18.507	-15.315	-1.198	1.00	0.00	C
ATOM	1341	CB	GLU	A	137	19.313	-16.235	-2.114	1.00	0.00	C
ATOM	1342	CG	GLU	A	137	20.226	-15.494	-3.092	1.00	0.00	C
ATOM	1343	CD	GLU	A	137	19.405	-14.972	-4.254	1.00	0.00	C
ATOM	1344	OE1	GLU	A	137	19.434	-15.598	-5.312	1.00	0.00	O
ATOM	1345	OE2	GLU	A	137	18.745	-13.946	-4.104	1.00	0.00	O
ATOM	1346	C	GLU	A	137	17.411	-16.105	-0.529	1.00	0.00	C
ATOM	1347	O	GLU	A	137	16.309	-16.221	-1.061	1.00	0.00	O
ATOM	1348	N	LEU	A	138	17.733	-16.708	0.638	1.00	0.00	N
ATOM	1349	H	LEU	A	138	18.660	-16.568	0.971	1.00	0.00	H
ATOM	1350	CA	LEU	A	138	16.842	-17.536	1.401	1.00	0.00	C
ATOM	1351	CB	LEU	A	138	17.643	-18.564	2.199	1.00	0.00	C
ATOM	1352	CG	LEU	A	138	18.554	-19.425	1.321	1.00	0.00	C
ATOM	1353	CD1	LEU	A	138	19.359	-20.417	2.161	1.00	0.00	C
ATOM	1354	CD2	LEU	A	138	17.791	-20.123	0.193	1.00	0.00	C
ATOM	1355	C	LEU	A	138	15.835	-16.857	2.281	1.00	0.00	C
ATOM	1356	O	LEU	A	138	14.704	-17.339	2.388	1.00	0.00	O
ATOM	1357	N	VAL	A	139	16.205	-15.720	2.923	1.00	0.00	N

```
ATOM   1358  H   VAL A 139      17.042 -15.259   2.642  1.00  0.00           H
ATOM   1359  CA  VAL A 139      15.296 -15.063   3.831  1.00  0.00           C
ATOM   1360  CB  VAL A 139      16.055 -14.236   4.873  1.00  0.00           C
ATOM   1361  CG1 VAL A 139      15.108 -13.624   5.912  1.00  0.00           C
ATOM   1362  CG2 VAL A 139      17.133 -15.091   5.540  1.00  0.00           C
ATOM   1363  C   VAL A 139      14.302 -14.226   3.077  1.00  0.00           C
ATOM   1364  O   VAL A 139      14.454 -13.015   2.922  1.00  0.00           O
ATOM   1365  N   ILE A 140      13.248 -14.887   2.557  1.00  0.00           N
ATOM   1366  H   ILE A 140      13.130 -15.874   2.704  1.00  0.00           H
ATOM   1367  CA  ILE A 140      12.215 -14.183   1.849  1.00  0.00           C
ATOM   1368  CB  ILE A 140      12.073 -14.660   0.418  1.00  0.00           C
ATOM   1369  CG2 ILE A 140      11.092 -13.721  -0.262  1.00  0.00           C
ATOM   1370  CG1 ILE A 140      13.401 -14.715  -0.333  1.00  0.00           C
ATOM   1371  CD1 ILE A 140      13.207 -15.193  -1.771  1.00  0.00           C
ATOM   1372  C   ILE A 140      10.986 -14.531   2.638  1.00  0.00           C
ATOM   1373  O   ILE A 140      10.719 -15.708   2.874  1.00  0.00           O
ATOM   1374  N   GLY A 141      10.185 -13.512   3.025  1.00  0.00           N
ATOM   1375  H   GLY A 141      10.352 -12.604   2.657  1.00  0.00           H
ATOM   1376  CA  GLY A 141       9.010 -13.728   3.829  1.00  0.00           C
ATOM   1377  C   GLY A 141       7.957 -14.585   3.200  1.00  0.00           C
ATOM   1378  O   GLY A 141       7.651 -14.462   2.010  1.00  0.00           O
ATOM   1379  N   ALA A 142       7.415 -15.495   4.039  1.00  0.00           N
ATOM   1380  H   ALA A 142       7.741 -15.582   4.984  1.00  0.00           H
ATOM   1381  CA  ALA A 142       6.360 -16.388   3.646  1.00  0.00           C
ATOM   1382  CB  ALA A 142       6.846 -17.831   3.531  1.00  0.00           C
ATOM   1383  C   ALA A 142       5.259 -16.318   4.659  1.00  0.00           C
ATOM   1384  O   ALA A 142       5.515 -16.271   5.866  1.00  0.00           O
ATOM   1385  N   VAL A 143       3.996 -16.283   4.161  1.00  0.00           N
ATOM   1386  H   VAL A 143       3.881 -16.362   3.165  1.00  0.00           H
ATOM   1387  CA  VAL A 143       2.811 -16.236   5.007  1.00  0.00           C
ATOM   1388  CB  VAL A 143       2.268 -14.816   5.244  1.00  0.00           C
ATOM   1389  CG1 VAL A 143       3.228 -13.939   6.029  1.00  0.00           C
ATOM   1390  CG2 VAL A 143       1.785 -14.157   3.951  1.00  0.00           C
ATOM   1391  C   VAL A 143       1.688 -17.028   4.399  1.00  0.00           C
ATOM   1392  O   VAL A 143       1.738 -17.400   3.225  1.00  0.00           O
ATOM   1393  N   ILE A 144       0.658 -17.334   5.227  1.00  0.00           N
ATOM   1394  H   ILE A 144       0.688 -17.007   6.170  1.00  0.00           H
ATOM   1395  CA  ILE A 144      -0.510 -18.027   4.761  1.00  0.00           C
ATOM   1396  CB  ILE A 144      -1.166 -18.882   5.851  1.00  0.00           C
ATOM   1397  CG2 ILE A 144      -2.533 -19.423   5.417  1.00  0.00           C
ATOM   1398  CG1 ILE A 144      -0.212 -20.016   6.244  1.00  0.00           C
ATOM   1399  CD1 ILE A 144      -0.742 -20.912   7.364  1.00  0.00           C
ATOM   1400  C   ILE A 144      -1.393 -16.892   4.287  1.00  0.00           C
ATOM   1401  O   ILE A 144      -1.823 -16.042   5.074  1.00  0.00           O
ATOM   1402  N   LEU A 145      -1.644 -16.862   2.962  1.00  0.00           N
ATOM   1403  H   LEU A 145      -1.221 -17.566   2.390  1.00  0.00           H
ATOM   1404  CA  LEU A 145      -2.444 -15.859   2.312  1.00  0.00           C
ATOM   1405  CB  LEU A 145      -1.481 -15.001   1.492  1.00  0.00           C
ATOM   1406  CG  LEU A 145      -1.932 -13.574   1.189  1.00  0.00           C
ATOM   1407  CD1 LEU A 145      -0.741 -12.626   1.146  1.00  0.00           C
ATOM   1408  CD2 LEU A 145      -2.792 -13.462  -0.068  1.00  0.00           C
ATOM   1409  C   LEU A 145      -3.447 -16.611   1.482  1.00  0.00           C
ATOM   1410  O   LEU A 145      -3.083 -17.509   0.722  1.00  0.00           O
ATOM   1411  N   ARG A 146      -4.748 -16.246   1.616  1.00  0.00           N
ATOM   1412  H   ARG A 146      -4.886 -15.423   2.177  1.00  0.00           H
ATOM   1413  CA  ARG A 146      -5.876 -16.846   0.926  1.00  0.00           C
ATOM   1414  CB  ARG A 146      -6.040 -16.478  -0.539  1.00  0.00           C
ATOM   1415  CG  ARG A 146      -7.399 -15.814  -0.749  1.00  0.00           C
ATOM   1416  CD  ARG A 146      -7.781 -15.769  -2.224  1.00  0.00           C
```

```
ATOM   1417  NE  ARG A 146      -8.931 -14.897  -2.450  1.00  0.00           N
ATOM   1418  HE  ARG A 146      -9.783 -15.070  -1.957  1.00  0.00           H
ATOM   1419  CZ  ARG A 146      -8.800 -13.883  -3.332  1.00  0.00           C
ATOM   1420  NH1 ARG A 146      -7.658 -13.670  -3.964  1.00  0.00           N
ATOM   1421 HH11 ARG A 146      -6.792 -14.137  -3.763  1.00  0.00           H
ATOM   1422 HH12 ARG A 146      -7.652 -12.952  -4.672  1.00  0.00           H
ATOM   1423  NH2 ARG A 146      -9.811 -13.064  -3.588  1.00  0.00           N
ATOM   1424 HH21 ARG A 146     -10.711 -13.128  -3.171  1.00  0.00           H
ATOM   1425 HH22 ARG A 146      -9.616 -12.321  -4.256  1.00  0.00           H
ATOM   1426  C   ARG A 146      -5.959 -18.341   1.190  1.00  0.00           C
ATOM   1427  O   ARG A 146      -6.397 -19.124   0.344  1.00  0.00           O
ATOM   1428  N   GLY A 147      -5.531 -18.755   2.408  1.00  0.00           N
ATOM   1429  H   GLY A 147      -5.483 -18.036   3.102  1.00  0.00           H
ATOM   1430  CA  GLY A 147      -5.541 -20.122   2.855  1.00  0.00           C
ATOM   1431  C   GLY A 147      -4.371 -20.943   2.386  1.00  0.00           C
ATOM   1432  O   GLY A 147      -4.324 -22.138   2.683  1.00  0.00           O
ATOM   1433  N   HIS A 148      -3.398 -20.340   1.656  1.00  0.00           N
ATOM   1434  H   HIS A 148      -3.418 -19.357   1.465  1.00  0.00           H
ATOM   1435  CA  HIS A 148      -2.260 -21.080   1.165  1.00  0.00           C
ATOM   1436  CB  HIS A 148      -2.286 -21.143  -0.367  1.00  0.00           C
ATOM   1437  CG  HIS A 148      -2.929 -22.411  -0.872  1.00  0.00           C
ATOM   1438  ND1 HIS A 148      -2.429 -23.144  -1.885  1.00  0.00           N
ATOM   1439  HD1 HIS A 148      -1.650 -22.925  -2.438  1.00  0.00           H
ATOM   1440  CD2 HIS A 148      -4.086 -23.031  -0.401  1.00  0.00           C
ATOM   1441  NE2 HIS A 148      -4.274 -24.156  -1.135  1.00  0.00           N
ATOM   1442  CE1 HIS A 148      -3.257 -24.224  -2.054  1.00  0.00           C
ATOM   1443  C   HIS A 148      -0.979 -20.391   1.529  1.00  0.00           C
ATOM   1444  O   HIS A 148      -0.886 -19.166   1.457  1.00  0.00           O
ATOM   1445  N   LEU A 149       0.049 -21.176   1.937  1.00  0.00           N
ATOM   1446  H   LEU A 149      -0.027 -22.161   1.818  1.00  0.00           H
ATOM   1447  CA  LEU A 149       1.350 -20.656   2.287  1.00  0.00           C
ATOM   1448  CB  LEU A 149       2.146 -21.743   3.011  1.00  0.00           C
ATOM   1449  CG  LEU A 149       3.532 -21.329   3.508  1.00  0.00           C
ATOM   1450  CD1 LEU A 149       3.489 -20.104   4.419  1.00  0.00           C
ATOM   1451  CD2 LEU A 149       4.233 -22.499   4.198  1.00  0.00           C
ATOM   1452  C   LEU A 149       1.998 -20.219   0.997  1.00  0.00           C
ATOM   1453  O   LEU A 149       2.025 -20.972   0.024  1.00  0.00           O
ATOM   1454  N   ARG A 150       2.492 -18.960   0.967  1.00  0.00           N
ATOM   1455  H   ARG A 150       2.466 -18.372   1.777  1.00  0.00           H
ATOM   1456  CA  ARG A 150       3.114 -18.383  -0.201  1.00  0.00           C
ATOM   1457  CB  ARG A 150       2.223 -17.338  -0.869  1.00  0.00           C
ATOM   1458  CG  ARG A 150       0.764 -17.714  -1.091  1.00  0.00           C
ATOM   1459  CD  ARG A 150       0.011 -16.525  -1.682  1.00  0.00           C
ATOM   1460  NE  ARG A 150      -1.425 -16.777  -1.704  1.00  0.00           N
ATOM   1461  HE  ARG A 150      -1.786 -17.489  -1.093  1.00  0.00           H
ATOM   1462  CZ  ARG A 150      -2.260 -15.973  -2.397  1.00  0.00           C
ATOM   1463  NH1 ARG A 150      -1.816 -14.904  -3.051  1.00  0.00           N
ATOM   1464 HH11 ARG A 150      -0.846 -14.682  -3.106  1.00  0.00           H
ATOM   1465 HH12 ARG A 150      -2.478 -14.278  -3.495  1.00  0.00           H
ATOM   1466  NH2 ARG A 150      -3.556 -16.234  -2.417  1.00  0.00           N
ATOM   1467 HH21 ARG A 150      -3.965 -17.028  -1.965  1.00  0.00           H
ATOM   1468 HH22 ARG A 150      -4.169 -15.588  -2.891  1.00  0.00           H
ATOM   1469  C   ARG A 150       4.397 -17.663   0.115  1.00  0.00           C
ATOM   1470  O   ARG A 150       4.499 -17.025   1.165  1.00  0.00           O
ATOM   1471  N   ILE A 151       5.403 -17.797  -0.803  1.00  0.00           N
ATOM   1472  H   ILE A 151       5.175 -18.295  -1.644  1.00  0.00           H
ATOM   1473  CA  ILE A 151       6.699 -17.135  -0.711  1.00  0.00           C
ATOM   1474  CB  ILE A 151       7.836 -17.949  -1.345  1.00  0.00           C
ATOM   1475  CG2 ILE A 151       9.114 -17.115  -1.451  1.00  0.00           C
```

ATOM	1476	CG1	ILE	A	151	8.126	-19.235	-0.585	1.00	0.00	C
ATOM	1477	CD1	ILE	A	151	9.194	-20.077	-1.288	1.00	0.00	C
ATOM	1478	C	ILE	A	151	6.526	-15.863	-1.504	1.00	0.00	C
ATOM	1479	O	ILE	A	151	6.173	-15.908	-2.684	1.00	0.00	O
ATOM	1480	N	ALA	A	152	6.850	-14.709	-0.880	1.00	0.00	N
ATOM	1481	H	ALA	A	152	7.124	-14.759	0.085	1.00	0.00	H
ATOM	1482	CA	ALA	A	152	6.698	-13.412	-1.494	1.00	0.00	C
ATOM	1483	CB	ALA	A	152	6.949	-12.311	-0.465	1.00	0.00	C
ATOM	1484	C	ALA	A	152	7.582	-13.136	-2.673	1.00	0.00	C
ATOM	1485	O	ALA	A	152	7.123	-12.576	-3.669	1.00	0.00	O
ATOM	1486	N	GLY	A	153	8.860	-13.553	-2.594	1.00	0.00	N
ATOM	1487	H	GLY	A	153	9.106	-14.107	-1.802	1.00	0.00	H
ATOM	1488	CA	GLY	A	153	9.836	-13.312	-3.623	1.00	0.00	C
ATOM	1489	C	GLY	A	153	9.712	-14.173	-4.830	1.00	0.00	C
ATOM	1490	O	GLY	A	153	10.143	-13.758	-5.907	1.00	0.00	O
ATOM	1491	N	HIS	A	154	9.153	-15.393	-4.673	1.00	0.00	N
ATOM	1492	H	HIS	A	154	8.766	-15.666	-3.796	1.00	0.00	H
ATOM	1493	CA	HIS	A	154	9.029	-16.285	-5.795	1.00	0.00	C
ATOM	1494	CB	HIS	A	154	9.667	-17.635	-5.479	1.00	0.00	C
ATOM	1495	CG	HIS	A	154	11.145	-17.464	-5.231	1.00	0.00	C
ATOM	1496	ND1	HIS	A	154	11.889	-16.456	-5.726	1.00	0.00	N
ATOM	1497	HD1	HIS	A	154	11.577	-15.708	-6.282	1.00	0.00	H
ATOM	1498	CD2	HIS	A	154	11.965	-18.304	-4.476	1.00	0.00	C
ATOM	1499	NE2	HIS	A	154	13.215	-17.789	-4.523	1.00	0.00	N
ATOM	1500	CE1	HIS	A	154	13.174	-16.654	-5.289	1.00	0.00	C
ATOM	1501	C	HIS	A	154	7.624	-16.467	-6.272	1.00	0.00	C
ATOM	1502	O	HIS	A	154	7.419	-16.993	-7.367	1.00	0.00	O
ATOM	1503	N	HIS	A	155	6.627	-15.990	-5.480	1.00	0.00	N
ATOM	1504	H	HIS	A	155	6.852	-15.594	-4.592	1.00	0.00	H
ATOM	1505	CA	HIS	A	155	5.207	-16.087	-5.755	1.00	0.00	C
ATOM	1506	CB	HIS	A	155	4.805	-15.245	-6.971	1.00	0.00	C
ATOM	1507	CG	HIS	A	155	5.043	-13.785	-6.670	1.00	0.00	C
ATOM	1508	ND1	HIS	A	155	4.254	-13.053	-5.863	1.00	0.00	N
ATOM	1509	HD1	HIS	A	155	3.460	-13.364	-5.383	1.00	0.00	H
ATOM	1510	CD2	HIS	A	155	6.077	-12.973	-7.145	1.00	0.00	C
ATOM	1511	NE2	HIS	A	155	5.900	-11.738	-6.613	1.00	0.00	N
ATOM	1512	CE1	HIS	A	155	4.779	-11.788	-5.825	1.00	0.00	C
ATOM	1513	C	HIS	A	155	4.783	-17.537	-5.900	1.00	0.00	C
ATOM	1514	O	HIS	A	155	3.928	-17.880	-6.721	1.00	0.00	O
ATOM	1515	N	LEU	A	156	5.399	-18.425	-5.079	1.00	0.00	N
ATOM	1516	H	LEU	A	156	5.966	-18.077	-4.336	1.00	0.00	H
ATOM	1517	CA	LEU	A	156	5.117	-19.838	-5.082	1.00	0.00	C
ATOM	1518	CB	LEU	A	156	6.415	-20.620	-4.957	1.00	0.00	C
ATOM	1519	CG	LEU	A	156	7.325	-20.481	-6.170	1.00	0.00	C
ATOM	1520	CD1	LEU	A	156	8.596	-21.306	-5.983	1.00	0.00	C
ATOM	1521	CD2	LEU	A	156	6.595	-20.814	-7.472	1.00	0.00	C
ATOM	1522	C	LEU	A	156	4.214	-20.215	-3.965	1.00	0.00	C
ATOM	1523	O	LEU	A	156	4.233	-19.579	-2.910	1.00	0.00	O
ATOM	1524	N	GLY	A	157	3.400	-21.265	-4.221	1.00	0.00	N
ATOM	1525	H	GLY	A	157	3.721	-21.876	-4.950	1.00	0.00	H
ATOM	1526	CA	GLY	A	157	2.443	-21.798	-3.299	1.00	0.00	C
ATOM	1527	C	GLY	A	157	2.925	-22.921	-2.431	1.00	0.00	C
ATOM	1528	O	GLY	A	157	4.007	-23.494	-2.590	1.00	0.00	O
ATOM	1529	N	ARG	A	158	1.982	-23.306	-1.541	1.00	0.00	N
ATOM	1530	H	ARG	A	158	1.264	-22.618	-1.459	1.00	0.00	H
ATOM	1531	CA	ARG	A	158	2.069	-24.311	-0.516	1.00	0.00	C
ATOM	1532	CB	ARG	A	158	0.751	-24.399	0.269	1.00	0.00	C
ATOM	1533	CG	ARG	A	158	0.829	-25.359	1.462	1.00	0.00	C
ATOM	1534	CD	ARG	A	158	-0.370	-25.309	2.417	1.00	0.00	C

ATOM	1535	NE	ARG	A	158	-1.625	-25.694	1.771	1.00	0.00	N
ATOM	1536	HE	ARG	A	158	-1.578	-26.420	1.077	1.00	0.00	H
ATOM	1537	CZ	ARG	A	158	-2.779	-25.082	2.183	1.00	0.00	C
ATOM	1538	NH1	ARG	A	158	-2.720	-24.161	3.167	1.00	0.00	N
ATOM	1539	HH11	ARG	A	158	-1.904	-24.011	3.729	1.00	0.00	H
ATOM	1540	HH12	ARG	A	158	-3.513	-23.562	3.363	1.00	0.00	H
ATOM	1541	NH2	ARG	A	158	-3.959	-25.387	1.598	1.00	0.00	N
ATOM	1542	HH21	ARG	A	158	-4.038	-26.048	0.847	1.00	0.00	H
ATOM	1543	HH22	ARG	A	158	-4.817	-24.939	1.872	1.00	0.00	H
ATOM	1544	C	ARG	A	158	2.469	-25.649	-1.057	1.00	0.00	C
ATOM	1545	O	ARG	A	158	3.208	-26.368	-0.386	1.00	0.00	O
ATOM	1546	N	CYS	A	159	2.053	-25.975	-2.304	1.00	0.00	N
ATOM	1547	H	CYS	A	159	1.584	-25.285	-2.851	1.00	0.00	H
ATOM	1548	CA	CYS	A	159	2.337	-27.237	-2.942	1.00	0.00	C
ATOM	1549	CB	CYS	A	159	1.612	-27.234	-4.281	1.00	0.00	C
ATOM	1550	SG	CYS	A	159	-0.036	-26.513	-4.057	1.00	0.00	S
ATOM	1551	C	CYS	A	159	3.811	-27.500	-3.075	1.00	0.00	C
ATOM	1552	O	CYS	A	159	4.231	-28.650	-2.939	1.00	0.00	O
ATOM	1553	N	ASP	A	160	4.611	-26.444	-3.358	1.00	0.00	N
ATOM	1554	H	ASP	A	160	4.296	-25.492	-3.439	1.00	0.00	H
ATOM	1555	CA	ASP	A	160	6.039	-26.576	-3.491	1.00	0.00	C
ATOM	1556	CB	ASP	A	160	6.534	-25.765	-4.690	1.00	0.00	C
ATOM	1557	CG	ASP	A	160	6.189	-24.307	-4.501	1.00	0.00	C
ATOM	1558	OD1	ASP	A	160	5.193	-23.855	-5.070	1.00	0.00	O
ATOM	1559	OD2	ASP	A	160	6.916	-23.635	-3.777	1.00	0.00	O
ATOM	1560	C	ASP	A	160	6.770	-26.265	-2.216	1.00	0.00	C
ATOM	1561	O	ASP	A	160	7.770	-26.913	-1.898	1.00	0.00	O
ATOM	1562	N	ILE	A	161	6.248	-25.274	-1.447	1.00	0.00	N
ATOM	1563	H	ILE	A	161	5.592	-24.690	-1.933	1.00	0.00	H
ATOM	1564	CA	ILE	A	161	6.877	-24.796	-0.233	1.00	0.00	C
ATOM	1565	CB	ILE	A	161	6.187	-23.555	0.323	1.00	0.00	C
ATOM	1566	CG2	ILE	A	161	6.863	-23.079	1.609	1.00	0.00	C
ATOM	1567	CG1	ILE	A	161	6.150	-22.428	-0.691	1.00	0.00	C
ATOM	1568	CD1	ILE	A	161	5.258	-21.315	-0.164	1.00	0.00	C
ATOM	1569	C	ILE	A	161	6.905	-25.845	0.847	1.00	0.00	C
ATOM	1570	O	ILE	A	161	7.948	-26.010	1.478	1.00	0.00	O
ATOM	1571	N	LYS	A	162	5.799	-26.609	1.034	1.00	0.00	N
ATOM	1572	H	LYS	A	162	5.021	-26.444	0.427	1.00	0.00	H
ATOM	1573	CA	LYS	A	162	5.696	-27.606	2.076	1.00	0.00	C
ATOM	1574	CB	LYS	A	162	4.329	-28.313	2.043	1.00	0.00	C
ATOM	1575	CG	LYS	A	162	4.076	-29.021	0.702	1.00	0.00	C
ATOM	1576	CD	LYS	A	162	2.924	-30.020	0.643	1.00	0.00	C
ATOM	1577	CE	LYS	A	162	2.691	-30.512	-0.791	1.00	0.00	C
ATOM	1578	NZ	LYS	A	162	3.967	-30.835	-1.440	1.00	0.00	N
ATOM	1579	HZ1	LYS	A	162	4.668	-31.236	-0.783	1.00	0.00	H
ATOM	1580	HZ2	LYS	A	162	3.918	-31.521	-2.211	1.00	0.00	H
ATOM	1581	HZ3	LYS	A	162	4.476	-30.016	-1.852	1.00	0.00	H
ATOM	1582	C	LYS	A	162	6.751	-28.674	1.998	1.00	0.00	C
ATOM	1583	O	LYS	A	162	7.183	-29.177	3.038	1.00	0.00	O
ATOM	1584	N	ASP	A	163	7.178	-29.030	0.765	1.00	0.00	N
ATOM	1585	H	ASP	A	163	6.895	-28.517	-0.043	1.00	0.00	H
ATOM	1586	CA	ASP	A	163	8.173	-30.036	0.550	1.00	0.00	C
ATOM	1587	CB	ASP	A	163	8.168	-30.524	-0.901	1.00	0.00	C
ATOM	1588	CG	ASP	A	163	6.807	-31.089	-1.249	1.00	0.00	C
ATOM	1589	OD1	ASP	A	163	6.228	-31.819	-0.448	1.00	0.00	O
ATOM	1590	OD2	ASP	A	163	6.276	-30.763	-2.305	1.00	0.00	O
ATOM	1591	C	ASP	A	163	9.538	-29.556	0.926	1.00	0.00	C
ATOM	1592	O	ASP	A	163	10.246	-30.283	1.625	1.00	0.00	O
ATOM	1593	N	LEU	A	164	9.936	-28.319	0.518	1.00	0.00	N

```
ATOM   1594  H   LEU A 164       9.325 -27.714   0.004  1.00  0.00           H
ATOM   1595  CA  LEU A 164      11.258 -27.840   0.855  1.00  0.00           C
ATOM   1596  CB  LEU A 164      12.025 -27.756  -0.463  1.00  0.00           C
ATOM   1597  CG  LEU A 164      11.986 -28.999  -1.361  1.00  0.00           C
ATOM   1598  CD1 LEU A 164      12.541 -28.689  -2.750  1.00  0.00           C
ATOM   1599  CD2 LEU A 164      12.674 -30.213  -0.733  1.00  0.00           C
ATOM   1600  C   LEU A 164      11.251 -26.441   1.453  1.00  0.00           C
ATOM   1601  O   LEU A 164      11.786 -25.519   0.831  1.00  0.00           O
ATOM   1602  N   PRO A 165      10.703 -26.197   2.649  1.00  0.00           N
ATOM   1603  CD  PRO A 165      10.081 -27.209   3.504  1.00  0.00           C
ATOM   1604  CA  PRO A 165      10.693 -24.879   3.254  1.00  0.00           C
ATOM   1605  CB  PRO A 165       9.682 -25.044   4.393  1.00  0.00           C
ATOM   1606  CG  PRO A 165       9.805 -26.502   4.823  1.00  0.00           C
ATOM   1607  C   PRO A 165      12.080 -24.472   3.694  1.00  0.00           C
ATOM   1608  O   PRO A 165      12.371 -23.277   3.694  1.00  0.00           O
ATOM   1609  N   LYS A 166      12.925 -25.463   4.071  1.00  0.00           N
ATOM   1610  H   LYS A 166      12.701 -26.404   3.811  1.00  0.00           H
ATOM   1611  CA  LYS A 166      14.257 -25.270   4.585  1.00  0.00           C
ATOM   1612  CB  LYS A 166      14.815 -26.550   5.185  1.00  0.00           C
ATOM   1613  CG  LYS A 166      13.862 -27.094   6.230  1.00  0.00           C
ATOM   1614  CD  LYS A 166      14.298 -28.441   6.783  1.00  0.00           C
ATOM   1615  CE  LYS A 166      13.621 -28.671   8.121  1.00  0.00           C
ATOM   1616  NZ  LYS A 166      13.906 -27.478   8.922  1.00  0.00           N
ATOM   1617  HZ1 LYS A 166      13.701 -27.643   9.926  1.00  0.00           H
ATOM   1618  HZ2 LYS A 166      14.879 -27.138   8.800  1.00  0.00           H
ATOM   1619  HZ3 LYS A 166      13.267 -26.698   8.665  1.00  0.00           H
ATOM   1620  C   LYS A 166      15.169 -24.842   3.470  1.00  0.00           C
ATOM   1621  O   LYS A 166      16.019 -23.973   3.659  1.00  0.00           O
ATOM   1622  N   GLU A 167      15.011 -25.481   2.288  1.00  0.00           N
ATOM   1623  H   GLU A 167      14.311 -26.192   2.250  1.00  0.00           H
ATOM   1624  CA  GLU A 167      15.784 -25.221   1.106  1.00  0.00           C
ATOM   1625  CB  GLU A 167      16.119 -26.500   0.309  1.00  0.00           C
ATOM   1626  CG  GLU A 167      15.291 -27.787   0.502  1.00  0.00           C
ATOM   1627  CD  GLU A 167      15.119 -28.236   1.951  1.00  0.00           C
ATOM   1628  OE1 GLU A 167      16.090 -28.662   2.565  1.00  0.00           O
ATOM   1629  OE2 GLU A 167      14.011 -28.111   2.483  1.00  0.00           O
ATOM   1630  C   GLU A 167      15.305 -24.017   0.361  1.00  0.00           C
ATOM   1631  O   GLU A 167      16.122 -23.296  -0.212  1.00  0.00           O
ATOM   1632  N   ILE A 168      13.964 -23.787   0.380  1.00  0.00           N
ATOM   1633  H   ILE A 168      13.407 -24.519   0.768  1.00  0.00           H
ATOM   1634  CA  ILE A 168      13.247 -22.729  -0.291  1.00  0.00           C
ATOM   1635  CB  ILE A 168      13.827 -21.318  -0.072  1.00  0.00           C
ATOM   1636  CG2 ILE A 168      13.167 -20.324  -1.033  1.00  0.00           C
ATOM   1637  CG1 ILE A 168      13.663 -20.825   1.368  1.00  0.00           C
ATOM   1638  CD1 ILE A 168      14.751 -21.242   2.351  1.00  0.00           C
ATOM   1639  C   ILE A 168      13.239 -23.075  -1.754  1.00  0.00           C
ATOM   1640  O   ILE A 168      14.262 -22.992  -2.438  1.00  0.00           O
ATOM   1641  N   THR A 169      12.062 -23.538  -2.238  1.00  0.00           N
ATOM   1642  H   THR A 169      11.266 -23.665  -1.648  1.00  0.00           H
ATOM   1643  CA  THR A 169      11.862 -23.904  -3.618  1.00  0.00           C
ATOM   1644  CB  THR A 169      10.494 -24.561  -3.614  1.00  0.00           C
ATOM   1645  OG1 THR A 169      10.322 -25.215  -2.346  1.00  0.00           O
ATOM   1646  HG1 THR A 169       9.460 -25.626  -2.367  1.00  0.00           H
ATOM   1647  CG2 THR A 169      10.323 -25.538  -4.777  1.00  0.00           C
ATOM   1648  C   THR A 169      11.929 -22.609  -4.402  1.00  0.00           C
ATOM   1649  O   THR A 169      11.307 -21.614  -4.024  1.00  0.00           O
ATOM   1650  N   VAL A 170      12.752 -22.598  -5.478  1.00  0.00           N
ATOM   1651  H   VAL A 170      13.199 -23.441  -5.771  1.00  0.00           H
ATOM   1652  CA  VAL A 170      12.946 -21.437  -6.310  1.00  0.00           C
```

```
ATOM   1653  CB  VAL A 170      14.435 -21.086  -6.395  1.00  0.00           C
ATOM   1654  CG1 VAL A 170      14.673 -19.857  -7.275  1.00  0.00           C
ATOM   1655  CG2 VAL A 170      15.038 -20.912  -5.000  1.00  0.00           C
ATOM   1656  C   VAL A 170      12.372 -21.648  -7.688  1.00  0.00           C
ATOM   1657  O   VAL A 170      12.684 -22.635  -8.359  1.00  0.00           O
ATOM   1658  N   ALA A 171      11.492 -20.706  -8.106  1.00  0.00           N
ATOM   1659  H   ALA A 171      11.200 -19.949  -7.516  1.00  0.00           H
ATOM   1660  CA  ALA A 171      10.872 -20.696  -9.409  1.00  0.00           C
ATOM   1661  CB  ALA A 171       9.724 -21.702  -9.514  1.00  0.00           C
ATOM   1662  C   ALA A 171      10.350 -19.302  -9.623  1.00  0.00           C
ATOM   1663  O   ALA A 171       9.795 -18.697  -8.704  1.00  0.00           O
ATOM   1664  N   THR A 172      10.550 -18.756 -10.844  1.00  0.00           N
ATOM   1665  H   THR A 172      10.987 -19.295 -11.559  1.00  0.00           H
ATOM   1666  CA  THR A 172      10.109 -17.428 -11.196  1.00  0.00           C
ATOM   1667  CB  THR A 172      11.294 -16.832 -11.920  1.00  0.00           C
ATOM   1668  OG1 THR A 172      12.045 -17.911 -12.494  1.00  0.00           O
ATOM   1669  HG1 THR A 172      11.477 -18.308 -13.147  1.00  0.00           H
ATOM   1670  CG2 THR A 172      12.184 -16.023 -10.975  1.00  0.00           C
ATOM   1671  C   THR A 172       8.845 -17.437 -12.012  1.00  0.00           C
ATOM   1672  O   THR A 172       8.656 -18.299 -12.874  1.00  0.00           O
ATOM   1673  N   SER A 173       7.946 -16.467 -11.719  1.00  0.00           N
ATOM   1674  H   SER A 173       8.018 -15.919 -10.887  1.00  0.00           H
ATOM   1675  CA  SER A 173       6.682 -16.292 -12.391  1.00  0.00           C
ATOM   1676  CB  SER A 173       5.797 -16.943 -11.347  1.00  0.00           C
ATOM   1677  OG  SER A 173       6.725 -17.327 -10.316  1.00  0.00           O
ATOM   1678  HG  SER A 173       6.258 -17.432  -9.491  1.00  0.00           H
ATOM   1679  C   SER A 173       6.485 -14.811 -12.563  1.00  0.00           C
ATOM   1680  O   SER A 173       6.732 -14.040 -11.633  1.00  0.00           O
ATOM   1681  N   ARG A 174       6.051 -14.375 -13.772  1.00  0.00           N
ATOM   1682  H   ARG A 174       5.747 -15.003 -14.490  1.00  0.00           H
ATOM   1683  CA  ARG A 174       5.838 -12.976 -14.053  1.00  0.00           C
ATOM   1684  CB  ARG A 174       7.023 -12.358 -14.804  1.00  0.00           C
ATOM   1685  CG  ARG A 174       8.208 -12.100 -13.868  1.00  0.00           C
ATOM   1686  CD  ARG A 174       9.434 -11.482 -14.540  1.00  0.00           C
ATOM   1687  NE  ARG A 174      10.473 -11.226 -13.542  1.00  0.00           N
ATOM   1688  HE  ARG A 174      10.282 -10.460 -12.918  1.00  0.00           H
ATOM   1689  CZ  ARG A 174      11.561 -12.051 -13.462  1.00  0.00           C
ATOM   1690  NH1 ARG A 174      11.704 -13.049 -14.366  1.00  0.00           N
ATOM   1691 HH11 ARG A 174      11.029 -13.190 -15.096  1.00  0.00           H
ATOM   1692 HH12 ARG A 174      12.480 -13.687 -14.357  1.00  0.00           H
ATOM   1693  NH2 ARG A 174      12.473 -11.855 -12.481  1.00  0.00           N
ATOM   1694 HH21 ARG A 174      12.364 -11.117 -11.807  1.00  0.00           H
ATOM   1695 HH22 ARG A 174      13.294 -12.426 -12.374  1.00  0.00           H
ATOM   1696  C   ARG A 174       4.546 -12.754 -14.794  1.00  0.00           C
ATOM   1697  O   ARG A 174       4.018 -13.656 -15.447  1.00  0.00           O
ATOM   1698  N   THR A 175       4.001 -11.516 -14.681  1.00  0.00           N
ATOM   1699  H   THR A 175       4.453 -10.801 -14.148  1.00  0.00           H
ATOM   1700  CA  THR A 175       2.775 -11.088 -15.317  1.00  0.00           C
ATOM   1701  CB  THR A 175       2.513  -9.739 -14.696  1.00  0.00           C
ATOM   1702  OG1 THR A 175       2.960  -9.817 -13.337  1.00  0.00           O
ATOM   1703  HG1 THR A 175       2.334 -10.378 -12.891  1.00  0.00           H
ATOM   1704  CG2 THR A 175       1.053  -9.299 -14.804  1.00  0.00           C
ATOM   1705  C   THR A 175       3.048 -11.054 -16.807  1.00  0.00           C
ATOM   1706  O   THR A 175       4.115 -10.613 -17.238  1.00  0.00           O
ATOM   1707  N   LEU A 176       2.075 -11.504 -17.631  1.00  0.00           N
ATOM   1708  H   LEU A 176       1.258 -11.883 -17.201  1.00  0.00           H
ATOM   1709  CA  LEU A 176       2.214 -11.545 -19.070  1.00  0.00           C
ATOM   1710  CB  LEU A 176       1.010 -12.225 -19.722  1.00  0.00           C
ATOM   1711  CG  LEU A 176       0.872 -13.699 -19.341  1.00  0.00           C
```

```
ATOM   1712  CD1 LEU A 176      -0.358 -14.332 -19.995  1.00  0.00           C
ATOM   1713  CD2 LEU A 176       2.148 -14.488 -19.637  1.00  0.00           C
ATOM   1714  C   LEU A 176       2.419 -10.190 -19.688  1.00  0.00           C
ATOM   1715  O   LEU A 176       3.189 -10.058 -20.640  1.00  0.00           O
ATOM   1716  N   SER A 177       1.761  -9.154 -19.131  1.00  0.00           N
ATOM   1717  H   SER A 177       1.119  -9.303 -18.383  1.00  0.00           H
ATOM   1718  CA  SER A 177       1.855  -7.807 -19.620  1.00  0.00           C
ATOM   1719  CB  SER A 177       0.396  -7.386 -19.587  1.00  0.00           C
ATOM   1720  OG  SER A 177      -0.366  -8.585 -19.325  1.00  0.00           O
ATOM   1721  HG  SER A 177      -1.228  -8.286 -19.034  1.00  0.00           H
ATOM   1722  C   SER A 177       2.785  -6.948 -18.785  1.00  0.00           C
ATOM   1723  O   SER A 177       2.713  -5.717 -18.839  1.00  0.00           O
ATOM   1724  N   TYR A 178       3.723  -7.594 -18.042  1.00  0.00           N
ATOM   1725  H   TYR A 178       3.784  -8.592 -18.121  1.00  0.00           H
ATOM   1726  CA  TYR A 178       4.667  -6.973 -17.140  1.00  0.00           C
ATOM   1727  CB  TYR A 178       5.586  -8.043 -16.550  1.00  0.00           C
ATOM   1728  CG  TYR A 178       6.517  -7.478 -15.506  1.00  0.00           C
ATOM   1729  CD1 TYR A 178       6.040  -7.177 -14.237  1.00  0.00           C
ATOM   1730  CE1 TYR A 178       6.911  -6.693 -13.268  1.00  0.00           C
ATOM   1731  CD2 TYR A 178       7.858  -7.277 -15.815  1.00  0.00           C
ATOM   1732  CE2 TYR A 178       8.726  -6.788 -14.847  1.00  0.00           C
ATOM   1733  CZ  TYR A 178       8.254  -6.504 -13.572  1.00  0.00           C
ATOM   1734  OH  TYR A 178       9.124  -6.039 -12.607  1.00  0.00           O
ATOM   1735  HH  TYR A 178       8.641  -5.852 -11.811  1.00  0.00           H
ATOM   1736  C   TYR A 178       5.500  -5.870 -17.735  1.00  0.00           C
ATOM   1737  O   TYR A 178       5.694  -4.871 -17.042  1.00  0.00           O
ATOM   1738  N   TYR A 179       5.977  -5.986 -19.003  1.00  0.00           N
ATOM   1739  H   TYR A 179       5.758  -6.805 -19.532  1.00  0.00           H
ATOM   1740  CA  TYR A 179       6.808  -4.942 -19.565  1.00  0.00           C
ATOM   1741  CB  TYR A 179       7.606  -5.309 -20.812  1.00  0.00           C
ATOM   1742  CG  TYR A 179       8.737  -4.310 -20.781  1.00  0.00           C
ATOM   1743  CD1 TYR A 179       9.471  -4.190 -19.607  1.00  0.00           C
ATOM   1744  CE1 TYR A 179      10.413  -3.182 -19.473  1.00  0.00           C
ATOM   1745  CD2 TYR A 179       9.014  -3.479 -21.861  1.00  0.00           C
ATOM   1746  CE2 TYR A 179       9.964  -2.470 -21.727  1.00  0.00           C
ATOM   1747  CZ  TYR A 179      10.637  -2.299 -20.520  1.00  0.00           C
ATOM   1748  OH  TYR A 179      11.525  -1.250 -20.342  1.00  0.00           O
ATOM   1749  HH  TYR A 179      11.998  -1.145 -21.167  1.00  0.00           H
ATOM   1750  C   TYR A 179       6.031  -3.661 -19.745  1.00  0.00           C
ATOM   1751  O   TYR A 179       6.535  -2.595 -19.381  1.00  0.00           O
ATOM   1752  N   LYS A 180       4.779  -3.749 -20.256  1.00  0.00           N
ATOM   1753  H   LYS A 180       4.370  -4.652 -20.383  1.00  0.00           H
ATOM   1754  CA  LYS A 180       3.963  -2.577 -20.460  1.00  0.00           C
ATOM   1755  CB  LYS A 180       2.686  -2.887 -21.239  1.00  0.00           C
ATOM   1756  CG  LYS A 180       2.900  -3.078 -22.740  1.00  0.00           C
ATOM   1757  CD  LYS A 180       1.559  -3.083 -23.475  1.00  0.00           C
ATOM   1758  CE  LYS A 180       1.691  -3.140 -24.998  1.00  0.00           C
ATOM   1759  NZ  LYS A 180       0.361  -2.994 -25.593  1.00  0.00           N
ATOM   1760  HZ1 LYS A 180      -0.337  -3.677 -25.226  1.00  0.00           H
ATOM   1761  HZ2 LYS A 180      -0.103  -2.105 -25.297  1.00  0.00           H
ATOM   1762  HZ3 LYS A 180       0.322  -3.000 -26.627  1.00  0.00           H
ATOM   1763  C   LYS A 180       3.593  -1.959 -19.147  1.00  0.00           C
ATOM   1764  O   LYS A 180       3.611  -0.735 -19.025  1.00  0.00           O
ATOM   1765  N   LEU A 181       3.321  -2.803 -18.122  1.00  0.00           N
ATOM   1766  H   LEU A 181       3.329  -3.790 -18.294  1.00  0.00           H
ATOM   1767  CA  LEU A 181       2.956  -2.339 -16.808  1.00  0.00           C
ATOM   1768  CB  LEU A 181       2.575  -3.501 -15.896  1.00  0.00           C
ATOM   1769  CG  LEU A 181       1.356  -4.305 -16.328  1.00  0.00           C
ATOM   1770  CD1 LEU A 181       1.159  -5.506 -15.408  1.00  0.00           C
```

```
ATOM   1771  CD2 LEU A 181       0.098  -3.447 -16.431  1.00  0.00           C
ATOM   1772  C   LEU A 181       4.123  -1.626 -16.171  1.00  0.00           C
ATOM   1773  O   LEU A 181       3.927  -0.593 -15.532  1.00  0.00           O
ATOM   1774  N   GLY A 182       5.366  -2.129 -16.389  1.00  0.00           N
ATOM   1775  H   GLY A 182       5.448  -2.973 -16.920  1.00  0.00           H
ATOM   1776  CA  GLY A 182       6.571  -1.559 -15.836  1.00  0.00           C
ATOM   1777  C   GLY A 182       6.849  -0.208 -16.403  1.00  0.00           C
ATOM   1778  O   GLY A 182       7.169   0.718 -15.654  1.00  0.00           O
ATOM   1779  N   ALA A 183       6.668  -0.064 -17.738  1.00  0.00           N
ATOM   1780  H   ALA A 183       6.376  -0.869 -18.260  1.00  0.00           H
ATOM   1781  CA  ALA A 183       6.893   1.177 -18.435  1.00  0.00           C
ATOM   1782  CB  ALA A 183       6.767   0.975 -19.945  1.00  0.00           C
ATOM   1783  C   ALA A 183       5.882   2.194 -17.981  1.00  0.00           C
ATOM   1784  O   ALA A 183       6.226   3.356 -17.759  1.00  0.00           O
ATOM   1785  N   SER A 184       4.624   1.741 -17.768  1.00  0.00           N
ATOM   1786  H   SER A 184       4.428   0.767 -17.884  1.00  0.00           H
ATOM   1787  CA  SER A 184       3.537   2.570 -17.326  1.00  0.00           C
ATOM   1788  CB  SER A 184       2.239   1.779 -17.410  1.00  0.00           C
ATOM   1789  OG  SER A 184       2.075   1.304 -18.745  1.00  0.00           O
ATOM   1790  HG  SER A 184       2.893   0.888 -18.979  1.00  0.00           H
ATOM   1791  C   SER A 184       3.782   3.078 -15.931  1.00  0.00           C
ATOM   1792  O   SER A 184       3.509   4.244 -15.661  1.00  0.00           O
ATOM   1793  N   GLN A 185       4.350   2.231 -15.035  1.00  0.00           N
ATOM   1794  H   GLN A 185       4.555   1.296 -15.328  1.00  0.00           H
ATOM   1795  CA  GLN A 185       4.645   2.582 -13.665  1.00  0.00           C
ATOM   1796  CB  GLN A 185       5.209   1.430 -12.808  1.00  0.00           C
ATOM   1797  CG  GLN A 185       5.602   1.991 -11.423  1.00  0.00           C
ATOM   1798  CD  GLN A 185       6.358   1.020 -10.536  1.00  0.00           C
ATOM   1799  OE1 GLN A 185       7.529   1.208 -10.194  1.00  0.00           O
ATOM   1800  NE2 GLN A 185       5.596  -0.002 -10.123  1.00  0.00           N
ATOM   1801 HE21 GLN A 185       5.923  -0.681  -9.466  1.00  0.00           H
ATOM   1802 HE22 GLN A 185       4.658  -0.096 -10.462  1.00  0.00           H
ATOM   1803  C   GLN A 185       5.673   3.679 -13.618  1.00  0.00           C
ATOM   1804  O   GLN A 185       5.498   4.650 -12.881  1.00  0.00           O
ATOM   1805  N   ARG A 186       6.726   3.559 -14.461  1.00  0.00           N
ATOM   1806  H   ARG A 186       6.759   2.750 -15.050  1.00  0.00           H
ATOM   1807  CA  ARG A 186       7.814   4.502 -14.518  1.00  0.00           C
ATOM   1808  CB  ARG A 186       8.879   4.066 -15.525  1.00  0.00           C
ATOM   1809  CG  ARG A 186       9.552   2.712 -15.312  1.00  0.00           C
ATOM   1810  CD  ARG A 186      10.424   2.384 -16.528  1.00  0.00           C
ATOM   1811  NE  ARG A 186      11.016   1.049 -16.452  1.00  0.00           N
ATOM   1812  HE  ARG A 186      11.306   0.748 -15.537  1.00  0.00           H
ATOM   1813  CZ  ARG A 186      11.188   0.355 -17.619  1.00  0.00           C
ATOM   1814  NH1 ARG A 186      10.742   0.874 -18.783  1.00  0.00           N
ATOM   1815 HH11 ARG A 186      10.293   1.769 -18.841  1.00  0.00           H
ATOM   1816 HH12 ARG A 186      10.853   0.356 -19.644  1.00  0.00           H
ATOM   1817  NH2 ARG A 186      11.801  -0.846 -17.617  1.00  0.00           N
ATOM   1818 HH21 ARG A 186      12.150  -1.281 -16.782  1.00  0.00           H
ATOM   1819 HH22 ARG A 186      11.928  -1.345 -18.487  1.00  0.00           H
ATOM   1820  C   ARG A 186       7.353   5.859 -14.964  1.00  0.00           C
ATOM   1821  O   ARG A 186       7.716   6.860 -14.342  1.00  0.00           O
ATOM   1822  N   VAL A 187       6.500   5.907 -16.017  1.00  0.00           N
ATOM   1823  H   VAL A 187       6.207   5.048 -16.441  1.00  0.00           H
ATOM   1824  CA  VAL A 187       6.014   7.154 -16.556  1.00  0.00           C
ATOM   1825  CB  VAL A 187       5.408   6.974 -17.949  1.00  0.00           C
ATOM   1826  CG1 VAL A 187       4.902   8.306 -18.510  1.00  0.00           C
ATOM   1827  CG2 VAL A 187       6.426   6.335 -18.895  1.00  0.00           C
ATOM   1828  C   VAL A 187       5.049   7.799 -15.588  1.00  0.00           C
ATOM   1829  O   VAL A 187       5.128   9.009 -15.373  1.00  0.00           O
```

```
ATOM   1830  N   ALA A 188       4.172   6.996 -14.940  1.00  0.00           N
ATOM   1831  H   ALA A 188       4.197   6.012 -15.123  1.00  0.00           H
ATOM   1832  CA  ALA A 188       3.182   7.487 -14.013  1.00  0.00           C
ATOM   1833  CB  ALA A 188       2.208   6.385 -13.607  1.00  0.00           C
ATOM   1834  C   ALA A 188       3.773   8.054 -12.765  1.00  0.00           C
ATOM   1835  O   ALA A 188       3.237   9.037 -12.253  1.00  0.00           O
ATOM   1836  N   GLY A 189       4.905   7.486 -12.274  1.00  0.00           N
ATOM   1837  H   GLY A 189       5.314   6.702 -12.744  1.00  0.00           H
ATOM   1838  CA  GLY A 189       5.537   7.963 -11.066  1.00  0.00           C
ATOM   1839  C   GLY A 189       5.998   9.383 -11.203  1.00  0.00           C
ATOM   1840  O   GLY A 189       5.774  10.191 -10.303  1.00  0.00           O
ATOM   1841  N   ASP A 190       6.621   9.712 -12.355  1.00  0.00           N
ATOM   1842  H   ASP A 190       6.855   9.002 -13.019  1.00  0.00           H
ATOM   1843  CA  ASP A 190       7.115  11.038 -12.610  1.00  0.00           C
ATOM   1844  CB  ASP A 190       8.360  10.989 -13.497  1.00  0.00           C
ATOM   1845  CG  ASP A 190       9.494  10.356 -12.704  1.00  0.00           C
ATOM   1846  OD1 ASP A 190       9.362  10.212 -11.487  1.00  0.00           O
ATOM   1847  OD2 ASP A 190      10.509  10.006 -13.303  1.00  0.00           O
ATOM   1848  C   ASP A 190       6.064  12.005 -13.090  1.00  0.00           C
ATOM   1849  O   ASP A 190       6.102  13.178 -12.716  1.00  0.00           O
ATOM   1850  N   SER A 191       5.090  11.532 -13.906  1.00  0.00           N
ATOM   1851  H   SER A 191       5.080  10.556 -14.117  1.00  0.00           H
ATOM   1852  CA  SER A 191       4.062  12.378 -14.471  1.00  0.00           C
ATOM   1853  CB  SER A 191       3.692  11.808 -15.834  1.00  0.00           C
ATOM   1854  OG  SER A 191       4.903  11.458 -16.518  1.00  0.00           O
ATOM   1855  HG  SER A 191       5.260  10.699 -16.067  1.00  0.00           H
ATOM   1856  C   SER A 191       2.886  12.665 -13.578  1.00  0.00           C
ATOM   1857  O   SER A 191       2.229  13.695 -13.749  1.00  0.00           O
ATOM   1858  N   GLY A 192       2.621  11.789 -12.585  1.00  0.00           N
ATOM   1859  H   GLY A 192       3.265  11.046 -12.398  1.00  0.00           H
ATOM   1860  CA  GLY A 192       1.504  11.935 -11.692  1.00  0.00           C
ATOM   1861  C   GLY A 192       1.952  12.554 -10.418  1.00  0.00           C
ATOM   1862  O   GLY A 192       2.583  13.615 -10.419  1.00  0.00           O
ATOM   1863  N   PHE A 193       1.608  11.890  -9.290  1.00  0.00           N
ATOM   1864  H   PHE A 193       1.272  10.955  -9.385  1.00  0.00           H
ATOM   1865  CA  PHE A 193       1.962  12.378  -7.987  1.00  0.00           C
ATOM   1866  CB  PHE A 193       1.070  11.737  -6.926  1.00  0.00           C
ATOM   1867  CG  PHE A 193      -0.365  12.136  -7.173  1.00  0.00           C
ATOM   1868  CD1 PHE A 193      -1.213  11.294  -7.883  1.00  0.00           C
ATOM   1869  CD2 PHE A 193      -0.836  13.353  -6.697  1.00  0.00           C
ATOM   1870  CE1 PHE A 193      -2.527  11.675  -8.127  1.00  0.00           C
ATOM   1871  CE2 PHE A 193      -2.151  13.732  -6.937  1.00  0.00           C
ATOM   1872  CZ  PHE A 193      -2.996  12.895  -7.655  1.00  0.00           C
ATOM   1873  C   PHE A 193       3.406  12.011  -7.817  1.00  0.00           C
ATOM   1874  O   PHE A 193       3.764  10.890  -7.436  1.00  0.00           O
ATOM   1875  N   ALA A 194       4.243  13.054  -8.027  1.00  0.00           N
ATOM   1876  H   ALA A 194       3.796  13.829  -8.478  1.00  0.00           H
ATOM   1877  CA  ALA A 194       5.686  13.066  -8.006  1.00  0.00           C
ATOM   1878  CB  ALA A 194       6.217  14.458  -8.347  1.00  0.00           C
ATOM   1879  C   ALA A 194       6.272  12.635  -6.705  1.00  0.00           C
ATOM   1880  O   ALA A 194       7.426  12.204  -6.660  1.00  0.00           O
ATOM   1881  N   ALA A 195       5.469  12.725  -5.620  1.00  0.00           N
ATOM   1882  H   ALA A 195       4.527  13.007  -5.778  1.00  0.00           H
ATOM   1883  CA  ALA A 195       5.854  12.337  -4.295  1.00  0.00           C
ATOM   1884  CB  ALA A 195       4.708  12.590  -3.320  1.00  0.00           C
ATOM   1885  C   ALA A 195       6.263  10.890  -4.285  1.00  0.00           C
ATOM   1886  O   ALA A 195       7.157  10.552  -3.517  1.00  0.00           O
ATOM   1887  N   TYR A 196       5.684  10.027  -5.170  1.00  0.00           N
ATOM   1888  H   TYR A 196       5.033  10.379  -5.845  1.00  0.00           H
```

```
ATOM   1889  CA  TYR A 196       6.019   8.621  -5.262  1.00  0.00           C
ATOM   1890  CB  TYR A 196       5.142   8.002  -6.341  1.00  0.00           C
ATOM   1891  CG  TYR A 196       5.509   6.559  -6.547  1.00  0.00           C
ATOM   1892  CD1 TYR A 196       5.007   5.588  -5.690  1.00  0.00           C
ATOM   1893  CE1 TYR A 196       5.316   4.254  -5.913  1.00  0.00           C
ATOM   1894  CD2 TYR A 196       6.338   6.210  -7.607  1.00  0.00           C
ATOM   1895  CE2 TYR A 196       6.649   4.877  -7.825  1.00  0.00           C
ATOM   1896  CZ  TYR A 196       6.131   3.903  -6.983  1.00  0.00           C
ATOM   1897  OH  TYR A 196       6.444   2.580  -7.216  1.00  0.00           O
ATOM   1898  HH  TYR A 196       5.631   2.072  -7.242  1.00  0.00           H
ATOM   1899  C   TYR A 196       7.479   8.465  -5.604  1.00  0.00           C
ATOM   1900  O   TYR A 196       8.163   7.637  -4.998  1.00  0.00           O
ATOM   1901  N   SER A 197       7.975   9.290  -6.555  1.00  0.00           N
ATOM   1902  H   SER A 197       7.415   9.937  -7.077  1.00  0.00           H
ATOM   1903  CA  SER A 197       9.342   9.249  -6.989  1.00  0.00           C
ATOM   1904  CB  SER A 197       9.374  10.075  -8.254  1.00  0.00           C
ATOM   1905  OG  SER A 197       8.123   9.815  -8.890  1.00  0.00           O
ATOM   1906  HG  SER A 197       8.273  10.042  -9.815  1.00  0.00           H
ATOM   1907  C   SER A 197      10.231   9.707  -5.875  1.00  0.00           C
ATOM   1908  O   SER A 197      11.249   9.067  -5.630  1.00  0.00           O
ATOM   1909  N   ARG A 198       9.821  10.760  -5.123  1.00  0.00           N
ATOM   1910  H   ARG A 198       8.951  11.188  -5.377  1.00  0.00           H
ATOM   1911  CA  ARG A 198      10.596  11.296  -4.022  1.00  0.00           C
ATOM   1912  CB  ARG A 198       9.944  12.541  -3.419  1.00  0.00           C
ATOM   1913  CG  ARG A 198       9.580  13.620  -4.433  1.00  0.00           C
ATOM   1914  CD  ARG A 198       9.210  14.946  -3.763  1.00  0.00           C
ATOM   1915  NE  ARG A 198       7.984  14.881  -2.966  1.00  0.00           N
ATOM   1916  HE  ARG A 198       7.904  14.185  -2.235  1.00  0.00           H
ATOM   1917  CZ  ARG A 198       7.061  15.868  -3.148  1.00  0.00           C
ATOM   1918  NH1 ARG A 198       7.268  16.790  -4.116  1.00  0.00           N
ATOM   1919 HH11 ARG A 198       8.088  16.749  -4.695  1.00  0.00           H
ATOM   1920 HH12 ARG A 198       6.630  17.543  -4.289  1.00  0.00           H
ATOM   1921  NH2 ARG A 198       5.962  15.912  -2.366  1.00  0.00           N
ATOM   1922 HH21 ARG A 198       5.856  15.210  -1.641  1.00  0.00           H
ATOM   1923 HH22 ARG A 198       5.236  16.599  -2.427  1.00  0.00           H
ATOM   1924  C   ARG A 198      10.750  10.297  -2.908  1.00  0.00           C
ATOM   1925  O   ARG A 198      11.821  10.203  -2.307  1.00  0.00           O
ATOM   1926  N   TYR A 199       9.687   9.493  -2.664  1.00  0.00           N
ATOM   1927  H   TYR A 199       8.859   9.656  -3.202  1.00  0.00           H
ATOM   1928  CA  TYR A 199       9.626   8.482  -1.639  1.00  0.00           C
ATOM   1929  CB  TYR A 199       8.233   7.843  -1.569  1.00  0.00           C
ATOM   1930  CG  TYR A 199       7.217   8.862  -1.110  1.00  0.00           C
ATOM   1931  CD1 TYR A 199       5.858   8.677  -1.347  1.00  0.00           C
ATOM   1932  CE1 TYR A 199       4.943   9.652  -0.955  1.00  0.00           C
ATOM   1933  CD2 TYR A 199       7.653  10.002  -0.451  1.00  0.00           C
ATOM   1934  CE2 TYR A 199       6.742  10.969  -0.065  1.00  0.00           C
ATOM   1935  CZ  TYR A 199       5.391  10.809  -0.324  1.00  0.00           C
ATOM   1936  OH  TYR A 199       4.517  11.811   0.043  1.00  0.00           O
ATOM   1937  HH  TYR A 199       5.045  12.582   0.280  1.00  0.00           H
ATOM   1938  C   TYR A 199      10.665   7.418  -1.847  1.00  0.00           C
ATOM   1939  O   TYR A 199      11.085   6.793  -0.872  1.00  0.00           O
ATOM   1940  N   ARG A 200      11.039   7.153  -3.123  1.00  0.00           N
ATOM   1941  H   ARG A 200      10.662   7.729  -3.851  1.00  0.00           H
ATOM   1942  CA  ARG A 200      12.039   6.177  -3.457  1.00  0.00           C
ATOM   1943  CB  ARG A 200      11.637   5.457  -4.757  1.00  0.00           C
ATOM   1944  CG  ARG A 200      10.319   4.671  -4.652  1.00  0.00           C
ATOM   1945  CD  ARG A 200       9.757   4.115  -5.976  1.00  0.00           C
ATOM   1946  NE  ARG A 200      10.660   3.166  -6.637  1.00  0.00           N
ATOM   1947  HE  ARG A 200      11.615   3.203  -6.329  1.00  0.00           H
```

```
ATOM   1948  CZ  ARG A 200      10.207   2.373  -7.668  1.00  0.00           C
ATOM   1949  NH1 ARG A 200       8.903   2.360  -8.016  1.00  0.00           N
ATOM   1950 HH11 ARG A 200       8.184   2.827  -7.481  1.00  0.00           H
ATOM   1951 HH12 ARG A 200       8.530   1.871  -8.820  1.00  0.00           H
ATOM   1952  NH2 ARG A 200      11.081   1.592  -8.340  1.00  0.00           N
ATOM   1953 HH21 ARG A 200      12.058   1.564  -8.112  1.00  0.00           H
ATOM   1954 HH22 ARG A 200      10.776   1.005  -9.096  1.00  0.00           H
ATOM   1955  C   ARG A 200      13.420   6.786  -3.582  1.00  0.00           C
ATOM   1956  O   ARG A 200      14.378   6.282  -2.995  1.00  0.00           O
ATOM   1957  N   ILE A 201      13.522   7.946  -4.282  1.00  0.00           N
ATOM   1958  H   ILE A 201      12.673   8.358  -4.617  1.00  0.00           H
ATOM   1959  CA  ILE A 201      14.757   8.652  -4.582  1.00  0.00           C
ATOM   1960  CB  ILE A 201      14.492   9.768  -5.603  1.00  0.00           C
ATOM   1961  CG2 ILE A 201      15.673  10.725  -5.777  1.00  0.00           C
ATOM   1962  CG1 ILE A 201      14.099   9.149  -6.945  1.00  0.00           C
ATOM   1963  CD1 ILE A 201      13.713  10.208  -7.979  1.00  0.00           C
ATOM   1964  C   ILE A 201      15.467   9.167  -3.357  1.00  0.00           C
ATOM   1965  O   ILE A 201      16.699   9.179  -3.345  1.00  0.00           O
ATOM   1966  N   GLY A 202      14.724   9.518  -2.280  1.00  0.00           N
ATOM   1967  H   GLY A 202      13.724   9.553  -2.357  1.00  0.00           H
ATOM   1968  CA  GLY A 202      15.302  10.033  -1.062  1.00  0.00           C
ATOM   1969  C   GLY A 202      16.262   9.079  -0.411  1.00  0.00           C
ATOM   1970  O   GLY A 202      17.190   9.517   0.268  1.00  0.00           O
ATOM   1971  N   ASN A 203      16.048   7.757  -0.605  1.00  0.00           N
ATOM   1972  H   ASN A 203      15.289   7.476  -1.192  1.00  0.00           H
ATOM   1973  CA  ASN A 203      16.898   6.744  -0.047  1.00  0.00           C
ATOM   1974  CB  ASN A 203      16.171   5.408   0.103  1.00  0.00           C
ATOM   1975  CG  ASN A 203      15.523   5.332   1.467  1.00  0.00           C
ATOM   1976  OD1 ASN A 203      15.087   6.330   2.028  1.00  0.00           O
ATOM   1977  ND2 ASN A 203      15.477   4.087   1.974  1.00  0.00           N
ATOM   1978 HD21 ASN A 203      15.063   3.923   2.870  1.00  0.00           H
ATOM   1979 HD22 ASN A 203      15.854   3.308   1.472  1.00  0.00           H
ATOM   1980  C   ASN A 203      18.143   6.538  -0.862  1.00  0.00           C
ATOM   1981  O   ASN A 203      19.188   6.198  -0.302  1.00  0.00           O
ATOM   1982  N   TYR A 204      18.051   6.720  -2.205  1.00  0.00           N
ATOM   1983  H   TYR A 204      17.222   7.140  -2.571  1.00  0.00           H
ATOM   1984  CA  TYR A 204      19.173   6.487  -3.071  1.00  0.00           C
ATOM   1985  CB  TYR A 204      18.699   5.778  -4.340  1.00  0.00           C
ATOM   1986  CG  TYR A 204      17.822   4.618  -3.931  1.00  0.00           C
ATOM   1987  CD1 TYR A 204      16.499   4.566  -4.356  1.00  0.00           C
ATOM   1988  CE1 TYR A 204      15.669   3.541  -3.916  1.00  0.00           C
ATOM   1989  CD2 TYR A 204      18.329   3.615  -3.112  1.00  0.00           C
ATOM   1990  CE2 TYR A 204      17.499   2.589  -2.674  1.00  0.00           C
ATOM   1991  CZ  TYR A 204      16.164   2.563  -3.061  1.00  0.00           C
ATOM   1992  OH  TYR A 204      15.330   1.568  -2.591  1.00  0.00           O
ATOM   1993  HH  TYR A 204      14.429   1.818  -2.738  1.00  0.00           H
ATOM   1994  C   TYR A 204      20.041   7.668  -3.400  1.00  0.00           C
ATOM   1995  O   TYR A 204      21.241   7.630  -3.116  1.00  0.00           O
ATOM   1996  N   LYS A 205      19.456   8.745  -3.988  1.00  0.00           N
ATOM   1997  H   LYS A 205      18.458   8.794  -4.024  1.00  0.00           H
ATOM   1998  CA  LYS A 205      20.241   9.878  -4.399  1.00  0.00           C
ATOM   1999  CB  LYS A 205      20.081  10.108  -5.904  1.00  0.00           C
ATOM   2000  CG  LYS A 205      20.417   8.880  -6.752  1.00  0.00           C
ATOM   2001  CD  LYS A 205      20.255   9.150  -8.248  1.00  0.00           C
ATOM   2002  CE  LYS A 205      20.606   7.933  -9.107  1.00  0.00           C
ATOM   2003  NZ  LYS A 205      20.449   8.272 -10.529  1.00  0.00           N
ATOM   2004  HZ1 LYS A 205      21.087   9.057 -10.770  1.00  0.00           H
ATOM   2005  HZ2 LYS A 205      20.686   7.444 -11.111  1.00  0.00           H
ATOM   2006  HZ3 LYS A 205      19.466   8.556 -10.715  1.00  0.00           H
```

```
ATOM   2007  C   LYS A 205      20.027  11.179  -3.687  1.00  0.00           C
ATOM   2008  O   LYS A 205      20.973  11.702  -3.094  1.00  0.00           O
ATOM   2009  N   LEU A 206      18.780  11.720  -3.716  1.00  0.00           N
ATOM   2010  H   LEU A 206      17.995  11.172  -4.004  1.00  0.00           H
ATOM   2011  CA  LEU A 206      18.512  13.006  -3.125  1.00  0.00           C
ATOM   2012  CB  LEU A 206      17.618  13.824  -4.050  1.00  0.00           C
ATOM   2013  CG  LEU A 206      18.233  14.030  -5.435  1.00  0.00           C
ATOM   2014  CD1 LEU A 206      17.246  14.687  -6.400  1.00  0.00           C
ATOM   2015  CD2 LEU A 206      19.560  14.789  -5.362  1.00  0.00           C
ATOM   2016  C   LEU A 206      17.927  12.943  -1.752  1.00  0.00           C
ATOM   2017  O   LEU A 206      16.716  12.816  -1.563  1.00  0.00           O
ATOM   2018  N   ASN A 207      18.813  13.125  -0.757  1.00  0.00           N
ATOM   2019  H   ASN A 207      19.766  13.259  -1.031  1.00  0.00           H
ATOM   2020  CA  ASN A 207      18.485  13.104   0.644  1.00  0.00           C
ATOM   2021  CB  ASN A 207      19.748  12.966   1.488  1.00  0.00           C
ATOM   2022  CG  ASN A 207      19.761  11.628   2.200  1.00  0.00           C
ATOM   2023  OD1 ASN A 207      20.016  11.539   3.395  1.00  0.00           O
ATOM   2024  ND2 ASN A 207      19.526  10.571   1.398  1.00  0.00           N
ATOM   2025 HD21 ASN A 207      19.621   9.625   1.709  1.00  0.00           H
ATOM   2026 HD22 ASN A 207      19.204  10.643   0.452  1.00  0.00           H
ATOM   2027  C   ASN A 207      17.694  14.299   1.091  1.00  0.00           C
ATOM   2028  O   ASN A 207      16.951  14.210   2.069  1.00  0.00           O
ATOM   2029  N   THR A 208      17.886  15.453   0.416  1.00  0.00           N
ATOM   2030  H   THR A 208      18.572  15.506  -0.305  1.00  0.00           H
ATOM   2031  CA  THR A 208      17.227  16.687   0.753  1.00  0.00           C
ATOM   2032  CB  THR A 208      18.411  17.613   0.864  1.00  0.00           C
ATOM   2033  OG1 THR A 208      19.576  16.789   0.674  1.00  0.00           O
ATOM   2034  HG1 THR A 208      20.307  17.282   1.027  1.00  0.00           H
ATOM   2035  CG2 THR A 208      18.466  18.345   2.206  1.00  0.00           C
ATOM   2036  C   THR A 208      16.141  17.159  -0.186  1.00  0.00           C
ATOM   2037  O   THR A 208      15.591  18.240   0.042  1.00  0.00           O
ATOM   2038  N   ASP A 209      15.758  16.372  -1.229  1.00  0.00           N
ATOM   2039  H   ASP A 209      16.035  15.410  -1.284  1.00  0.00           H
ATOM   2040  CA  ASP A 209      14.759  16.826  -2.183  1.00  0.00           C
ATOM   2041  CB  ASP A 209      14.648  15.912  -3.400  1.00  0.00           C
ATOM   2042  CG  ASP A 209      13.559  14.897  -3.160  1.00  0.00           C
ATOM   2043  OD1 ASP A 209      12.469  15.087  -3.685  1.00  0.00           O
ATOM   2044  OD2 ASP A 209      13.785  13.958  -2.401  1.00  0.00           O
ATOM   2045  C   ASP A 209      13.420  17.061  -1.525  1.00  0.00           C
ATOM   2046  O   ASP A 209      12.775  18.089  -1.750  1.00  0.00           O
ATOM   2047  N   HIS A 210      13.025  16.127  -0.641  1.00  0.00           N
ATOM   2048  H   HIS A 210      13.552  15.276  -0.681  1.00  0.00           H
ATOM   2049  CA  HIS A 210      11.799  16.179   0.098  1.00  0.00           C
ATOM   2050  CB  HIS A 210      11.381  14.811   0.648  1.00  0.00           C
ATOM   2051  CG  HIS A 210      12.545  14.018   1.185  1.00  0.00           C
ATOM   2052  ND1 HIS A 210      13.415  13.343   0.409  1.00  0.00           N
ATOM   2053  HD1 HIS A 210      13.414  13.280  -0.579  1.00  0.00           H
ATOM   2054  CD2 HIS A 210      12.889  13.823   2.523  1.00  0.00           C
ATOM   2055  NE2 HIS A 210      13.977  13.020   2.548  1.00  0.00           N
ATOM   2056  CE1 HIS A 210      14.303  12.726   1.249  1.00  0.00           C
ATOM   2057  C   HIS A 210      11.726  17.303   1.094  1.00  0.00           C
ATOM   2058  O   HIS A 210      10.629  17.794   1.352  1.00  0.00           O
ATOM   2059  N   SER A 211      12.875  17.758   1.656  1.00  0.00           N
ATOM   2060  H   SER A 211      13.733  17.298   1.437  1.00  0.00           H
ATOM   2061  CA  SER A 211      12.883  18.857   2.594  1.00  0.00           C
ATOM   2062  CB  SER A 211      14.209  18.808   3.326  1.00  0.00           C
ATOM   2063  OG  SER A 211      15.034  17.891   2.607  1.00  0.00           O
ATOM   2064  HG  SER A 211      15.410  18.403   1.897  1.00  0.00           H
ATOM   2065  C   SER A 211      12.593  20.162   1.889  1.00  0.00           C
```

ATOM	2066	O	SER	A	211	11.927	21.033	2.453	1.00	0.00	O
ATOM	2067	N	SER	A	212	13.093	20.314	0.637	1.00	0.00	N
ATOM	2068	H	SER	A	212	13.647	19.588	0.233	1.00	0.00	H
ATOM	2069	CA	SER	A	212	12.895	21.492	-0.175	1.00	0.00	C
ATOM	2070	CB	SER	A	212	13.968	21.361	-1.237	1.00	0.00	C
ATOM	2071	OG	SER	A	212	15.095	20.779	-0.565	1.00	0.00	O
ATOM	2072	HG	SER	A	212	15.190	19.881	-0.868	1.00	0.00	H
ATOM	2073	C	SER	A	212	11.466	21.581	-0.650	1.00	0.00	C
ATOM	2074	O	SER	A	212	10.864	22.656	-0.614	1.00	0.00	O
ATOM	2075	N	SER	A	213	10.896	20.428	-1.074	1.00	0.00	N
ATOM	2076	H	SER	A	213	11.403	19.564	-1.028	1.00	0.00	H
ATOM	2077	CA	SER	A	213	9.546	20.283	-1.566	1.00	0.00	C
ATOM	2078	CB	SER	A	213	9.505	19.039	-2.441	1.00	0.00	C
ATOM	2079	OG	SER	A	213	10.191	17.982	-1.780	1.00	0.00	O
ATOM	2080	HG	SER	A	213	11.012	17.847	-2.251	1.00	0.00	H
ATOM	2081	C	SER	A	213	8.510	20.336	-0.469	1.00	0.00	C
ATOM	2082	O	SER	A	213	7.321	20.499	-0.751	1.00	0.00	O
ATOM	2083	N	SER	A	214	8.961	20.197	0.804	1.00	0.00	N
ATOM	2084	H	SER	A	214	9.923	19.967	0.939	1.00	0.00	H
ATOM	2085	CA	SER	A	214	8.162	20.188	2.006	1.00	0.00	C
ATOM	2086	CB	SER	A	214	7.513	21.564	2.120	1.00	0.00	C
ATOM	2087	OG	SER	A	214	8.543	22.552	1.970	1.00	0.00	O
ATOM	2088	HG	SER	A	214	8.994	22.388	1.150	1.00	0.00	H
ATOM	2089	C	SER	A	214	7.220	19.009	2.042	1.00	0.00	C
ATOM	2090	O	SER	A	214	6.057	19.105	2.444	1.00	0.00	O
ATOM	2091	N	ASP	A	215	7.755	17.836	1.617	1.00	0.00	N
ATOM	2092	H	ASP	A	215	8.740	17.796	1.440	1.00	0.00	H
ATOM	2093	CA	ASP	A	215	7.048	16.586	1.599	1.00	0.00	C
ATOM	2094	CB	ASP	A	215	7.587	15.581	0.594	1.00	0.00	C
ATOM	2095	CG	ASP	A	215	6.621	14.410	0.503	1.00	0.00	C
ATOM	2096	OD1	ASP	A	215	6.038	14.006	1.509	1.00	0.00	O
ATOM	2097	OD2	ASP	A	215	6.422	13.916	-0.601	1.00	0.00	O
ATOM	2098	C	ASP	A	215	7.309	16.067	2.986	1.00	0.00	C
ATOM	2099	O	ASP	A	215	8.271	15.350	3.274	1.00	0.00	O
ATOM	2100	N	ASN	A	216	6.374	16.469	3.866	1.00	0.00	N
ATOM	2101	H	ASN	A	216	5.640	16.996	3.437	1.00	0.00	H
ATOM	2102	CA	ASN	A	216	6.325	16.218	5.280	1.00	0.00	C
ATOM	2103	CB	ASN	A	216	5.246	17.067	5.952	1.00	0.00	C
ATOM	2104	CG	ASN	A	216	5.830	18.392	6.396	1.00	0.00	C
ATOM	2105	OD1	ASN	A	216	6.156	18.593	7.559	1.00	0.00	O
ATOM	2106	ND2	ASN	A	216	5.932	19.306	5.410	1.00	0.00	N
ATOM	2107	HD21	ASN	A	216	6.283	20.222	5.601	1.00	0.00	H
ATOM	2108	HD22	ASN	A	216	5.666	19.125	4.459	1.00	0.00	H
ATOM	2109	C	ASN	A	216	6.141	14.781	5.650	1.00	0.00	C
ATOM	2110	O	ASN	A	216	6.549	14.367	6.737	1.00	0.00	O
ATOM	2111	N	ILE	A	217	5.489	14.001	4.760	1.00	0.00	N
ATOM	2112	H	ILE	A	217	5.369	14.354	3.830	1.00	0.00	H
ATOM	2113	CA	ILE	A	217	5.224	12.600	4.966	1.00	0.00	C
ATOM	2114	CB	ILE	A	217	4.182	12.110	3.970	1.00	0.00	C
ATOM	2115	CG2	ILE	A	217	3.625	10.764	4.421	1.00	0.00	C
ATOM	2116	CG1	ILE	A	217	3.076	13.150	3.787	1.00	0.00	C
ATOM	2117	CD1	ILE	A	217	2.121	12.792	2.650	1.00	0.00	C
ATOM	2118	C	ILE	A	217	6.551	11.873	4.809	1.00	0.00	C
ATOM	2119	O	ILE	A	217	6.953	11.116	5.696	1.00	0.00	O
ATOM	2120	N	ALA	A	218	7.303	12.182	3.718	1.00	0.00	N
ATOM	2121	H	ALA	A	218	6.935	12.832	3.046	1.00	0.00	H
ATOM	2122	CA	ALA	A	218	8.585	11.581	3.420	1.00	0.00	C
ATOM	2123	CB	ALA	A	218	9.139	12.081	2.090	1.00	0.00	C
ATOM	2124	C	ALA	A	218	9.598	11.924	4.473	1.00	0.00	C

```
ATOM   2125  O   ALA A 218      10.427  11.078   4.811  1.00  0.00           O
ATOM   2126  N   LEU A 219       9.526  13.163   5.027  1.00  0.00           N
ATOM   2127  H   LEU A 219       8.869  13.804   4.624  1.00  0.00           H
ATOM   2128  CA  LEU A 219      10.416  13.637   6.064  1.00  0.00           C
ATOM   2129  CB  LEU A 219      10.122  15.103   6.381  1.00  0.00           C
ATOM   2130  CG  LEU A 219      10.654  16.055   5.311  1.00  0.00           C
ATOM   2131  CD1 LEU A 219      10.093  17.470   5.458  1.00  0.00           C
ATOM   2132  CD2 LEU A 219      12.181  16.047   5.282  1.00  0.00           C
ATOM   2133  C   LEU A 219      10.329  12.816   7.313  1.00  0.00           C
ATOM   2134  O   LEU A 219      11.342  12.659   7.996  1.00  0.00           O
ATOM   2135  N   LEU A 220       9.128  12.275   7.637  1.00  0.00           N
ATOM   2136  H   LEU A 220       8.347  12.407   7.024  1.00  0.00           H
ATOM   2137  CA  LEU A 220       8.946  11.462   8.811  1.00  0.00           C
ATOM   2138  CB  LEU A 220       7.460  11.196   9.044  1.00  0.00           C
ATOM   2139  CG  LEU A 220       6.642  12.444   9.364  1.00  0.00           C
ATOM   2140  CD1 LEU A 220       5.146  12.127   9.433  1.00  0.00           C
ATOM   2141  CD2 LEU A 220       7.143  13.145  10.627  1.00  0.00           C
ATOM   2142  C   LEU A 220       9.684  10.162   8.649  1.00  0.00           C
ATOM   2143  O   LEU A 220      10.371   9.716   9.568  1.00  0.00           O
ATOM   2144  N   VAL A 221       9.574   9.561   7.445  1.00  0.00           N
ATOM   2145  H   VAL A 221       9.077  10.059   6.733  1.00  0.00           H
ATOM   2146  CA  VAL A 221      10.173   8.298   7.098  1.00  0.00           C
ATOM   2147  CB  VAL A 221       9.534   7.832   5.798  1.00  0.00           C
ATOM   2148  CG1 VAL A 221      10.253   6.644   5.156  1.00  0.00           C
ATOM   2149  CG2 VAL A 221       8.054   7.579   6.074  1.00  0.00           C
ATOM   2150  C   VAL A 221      11.669   8.295   7.017  1.00  0.00           C
ATOM   2151  O   VAL A 221      12.285   7.361   7.538  1.00  0.00           O
ATOM   2152  N   GLN A 222      12.271   9.330   6.391  1.00  0.00           N
ATOM   2153  H   GLN A 222      11.707  10.082   6.044  1.00  0.00           H
ATOM   2154  CA  GLN A 222      13.699   9.391   6.246  1.00  0.00           C
ATOM   2155  CB  GLN A 222      13.973  10.383   5.112  1.00  0.00           C
ATOM   2156  CG  GLN A 222      15.069   9.975   4.127  1.00  0.00           C
ATOM   2157  CD  GLN A 222      16.419  10.379   4.668  1.00  0.00           C
ATOM   2158  OE1 GLN A 222      16.558  10.807   5.809  1.00  0.00           O
ATOM   2159  NE2 GLN A 222      17.402  10.250   3.762  1.00  0.00           N
ATOM   2160 HE21 GLN A 222      18.341  10.543   3.967  1.00  0.00           H
ATOM   2161 HE22 GLN A 222      17.215   9.885   2.848  1.00  0.00           H
ATOM   2162  C   GLN A 222      14.348   9.772   7.574  1.00  0.00           C
ATOM   2163  O   GLN A 222      15.216   8.979   8.025  1.00  0.00           O
ATOM      1  N   MET B   1     -13.101  12.359  47.015  1.00  0.00           N
ATOM      2  H   MET B   1     -12.820  12.578  47.952  1.00  0.00           H
ATOM      3  CA  MET B   1     -14.515  12.437  46.648  1.00  0.00           C
ATOM      4  CB  MET B   1     -14.668  12.215  45.136  1.00  0.00           C
ATOM      5  CG  MET B   1     -16.094  12.126  44.578  1.00  0.00           C
ATOM      6  SD  MET B   1     -16.866  10.525  44.859  1.00  0.00           S
ATOM      7  CE  MET B   1     -18.485  10.911  44.177  1.00  0.00           C
ATOM      8  C   MET B   1     -15.391  11.521  47.485  1.00  0.00           C
ATOM      9  O   MET B   1     -16.530  11.835  47.803  1.00  0.00           O
ATOM     10  N   SER B   2     -14.781  10.392  47.876  1.00  0.00           N
ATOM     11  H   SER B   2     -13.865  10.153  47.555  1.00  0.00           H
ATOM     12  CA  SER B   2     -15.476   9.424  48.723  1.00  0.00           C
ATOM     13  CB  SER B   2     -14.467   8.365  49.154  1.00  0.00           C
ATOM     14  OG  SER B   2     -13.552   8.094  48.079  1.00  0.00           O
ATOM     15  HG  SER B   2     -12.804   7.658  48.492  1.00  0.00           H
ATOM     16  C   SER B   2     -16.195  10.023  49.929  1.00  0.00           C
ATOM     17  O   SER B   2     -17.380   9.815  50.156  1.00  0.00           O
ATOM     18  N   ASP B   3     -15.411  10.841  50.648  1.00  0.00           N
ATOM     19  H   ASP B   3     -14.418  10.847  50.509  1.00  0.00           H
ATOM     20  CA  ASP B   3     -15.885  11.625  51.793  1.00  0.00           C
```

ATOM	21	CB	ASP	B	3	-14.781	12.590	52.252	1.00	0.00	C
ATOM	22	CG	ASP	B	3	-13.521	11.883	52.723	1.00	0.00	C
ATOM	23	OD1	ASP	B	3	-13.037	12.213	53.799	1.00	0.00	O
ATOM	24	OD2	ASP	B	3	-13.020	10.994	52.039	1.00	0.00	O
ATOM	25	C	ASP	B	3	-17.129	12.457	51.528	1.00	0.00	C
ATOM	26	O	ASP	B	3	-17.998	12.661	52.368	1.00	0.00	O
ATOM	27	N	ASN	B	4	-17.157	12.992	50.302	1.00	0.00	N
ATOM	28	H	ASN	B	4	-16.561	12.629	49.588	1.00	0.00	H
ATOM	29	CA	ASN	B	4	-18.180	13.987	50.021	1.00	0.00	C
ATOM	30	CB	ASN	B	4	-17.688	14.978	48.965	1.00	0.00	C
ATOM	31	CG	ASN	B	4	-18.594	16.194	48.940	1.00	0.00	C
ATOM	32	OD1	ASN	B	4	-19.443	16.358	48.076	1.00	0.00	O
ATOM	33	ND2	ASN	B	4	-18.372	17.062	49.930	1.00	0.00	N
ATOM	34	HD21	ASN	B	4	-18.923	17.895	49.972	1.00	0.00	H
ATOM	35	HD22	ASN	B	4	-17.669	16.908	50.623	1.00	0.00	H
ATOM	36	C	ASN	B	4	-19.538	13.412	49.674	1.00	0.00	C
ATOM	37	O	ASN	B	4	-19.909	13.228	48.518	1.00	0.00	O
ATOM	38	N	GLY	B	5	-20.292	13.193	50.761	1.00	0.00	N
ATOM	39	H	GLY	B	5	-19.805	13.177	51.639	1.00	0.00	H
ATOM	40	CA	GLY	B	5	-21.725	12.882	50.677	1.00	0.00	C
ATOM	41	C	GLY	B	5	-22.490	13.476	49.495	1.00	0.00	C
ATOM	42	O	GLY	B	5	-22.936	12.754	48.612	1.00	0.00	O
ATOM	43	N	PRO	B	6	-22.616	14.832	49.489	1.00	0.00	N
ATOM	44	CD	PRO	B	6	-22.192	15.767	50.530	1.00	0.00	C
ATOM	45	CA	PRO	B	6	-23.256	15.522	48.360	1.00	0.00	C
ATOM	46	CB	PRO	B	6	-22.881	16.986	48.601	1.00	0.00	C
ATOM	47	CG	PRO	B	6	-22.802	17.102	50.120	1.00	0.00	C
ATOM	48	C	PRO	B	6	-22.910	15.016	46.965	1.00	0.00	C
ATOM	49	O	PRO	B	6	-23.792	14.667	46.190	1.00	0.00	O
ATOM	50	N	GLN	B	7	-21.597	14.971	46.678	1.00	0.00	N
ATOM	51	H	GLN	B	7	-20.914	15.253	47.355	1.00	0.00	H
ATOM	52	CA	GLN	B	7	-21.191	14.453	45.369	1.00	0.00	C
ATOM	53	CB	GLN	B	7	-19.681	14.558	45.169	1.00	0.00	C
ATOM	54	CG	GLN	B	7	-19.236	15.973	44.811	1.00	0.00	C
ATOM	55	CD	GLN	B	7	-17.725	16.017	44.755	1.00	0.00	C
ATOM	56	OE1	GLN	B	7	-17.078	15.498	43.849	1.00	0.00	O
ATOM	57	NE2	GLN	B	7	-17.186	16.683	45.777	1.00	0.00	N
ATOM	58	HE21	GLN	B	7	-16.199	16.818	45.833	1.00	0.00	H
ATOM	59	HE22	GLN	B	7	-17.796	17.048	46.483	1.00	0.00	H
ATOM	60	C	GLN	B	7	-21.642	13.034	45.093	1.00	0.00	C
ATOM	61	O	GLN	B	7	-22.164	12.727	44.032	1.00	0.00	O
ATOM	62	N	ASN	B	8	-21.455	12.180	46.113	1.00	0.00	N
ATOM	63	H	ASN	B	8	-21.065	12.524	46.970	1.00	0.00	H
ATOM	64	CA	ASN	B	8	-21.899	10.790	45.941	1.00	0.00	C
ATOM	65	CB	ASN	B	8	-21.636	9.948	47.194	1.00	0.00	C
ATOM	66	CG	ASN	B	8	-20.181	9.525	47.271	1.00	0.00	C
ATOM	67	OD1	ASN	B	8	-19.776	8.483	46.773	1.00	0.00	O
ATOM	68	ND2	ASN	B	8	-19.412	10.366	47.968	1.00	0.00	N
ATOM	69	HD21	ASN	B	8	-18.471	10.122	48.201	1.00	0.00	H
ATOM	70	HD22	ASN	B	8	-19.744	11.253	48.287	1.00	0.00	H
ATOM	71	C	ASN	B	8	-23.362	10.684	45.542	1.00	0.00	C
ATOM	72	O	ASN	B	8	-23.732	10.039	44.570	1.00	0.00	O
ATOM	73	N	GLN	B	9	-24.175	11.425	46.310	1.00	0.00	N
ATOM	74	H	GLN	B	9	-23.764	11.947	47.059	1.00	0.00	H
ATOM	75	CA	GLN	B	9	-25.607	11.503	46.009	1.00	0.00	C
ATOM	76	CB	GLN	B	9	-26.296	12.423	47.015	1.00	0.00	C
ATOM	77	CG	GLN	B	9	-26.090	12.027	48.476	1.00	0.00	C
ATOM	78	CD	GLN	B	9	-26.462	13.199	49.361	1.00	0.00	C
ATOM	79	OE1	GLN	B	9	-26.596	14.338	48.927	1.00	0.00	O

ATOM	80	NE2	GLN	B	9	-26.625	12.872	50.645	1.00	0.00	N
ATOM	81	HE21	GLN	B	9	-26.868	13.577	51.308	1.00	0.00	H
ATOM	82	HE22	GLN	B	9	-26.508	11.928	50.948	1.00	0.00	H
ATOM	83	C	GLN	B	9	-25.920	12.002	44.607	1.00	0.00	C
ATOM	84	O	GLN	B	9	-26.770	11.487	43.895	1.00	0.00	O
ATOM	85	N	ARG	B	10	-25.191	13.067	44.253	1.00	0.00	N
ATOM	86	H	ARG	B	10	-24.451	13.390	44.845	1.00	0.00	H
ATOM	87	CA	ARG	B	10	-25.464	13.711	42.971	1.00	0.00	C
ATOM	88	CB	ARG	B	10	-25.064	15.189	43.015	1.00	0.00	C
ATOM	89	CG	ARG	B	10	-26.151	16.153	43.504	1.00	0.00	C
ATOM	90	CD	ARG	B	10	-26.580	15.987	44.964	1.00	0.00	C
ATOM	91	NE	ARG	B	10	-27.605	16.973	45.307	1.00	0.00	N
ATOM	92	HE	ARG	B	10	-27.928	17.553	44.557	1.00	0.00	H
ATOM	93	CZ	ARG	B	10	-28.084	17.073	46.565	1.00	0.00	C
ATOM	94	NH1	ARG	B	10	-27.638	16.273	47.530	1.00	0.00	N
ATOM	95	HH11	ARG	B	10	-26.942	15.566	47.365	1.00	0.00	H
ATOM	96	HH12	ARG	B	10	-27.966	16.315	48.474	1.00	0.00	H
ATOM	97	NH2	ARG	B	10	-29.015	17.987	46.833	1.00	0.00	N
ATOM	98	HH21	ARG	B	10	-29.356	18.592	46.113	1.00	0.00	H
ATOM	99	HH22	ARG	B	10	-29.396	18.090	47.753	1.00	0.00	H
ATOM	100	C	ARG	B	10	-24.813	13.050	41.770	1.00	0.00	C
ATOM	101	O	ARG	B	10	-25.019	13.452	40.631	1.00	0.00	O
ATOM	102	N	ASN	B	11	-23.980	12.043	42.052	1.00	0.00	N
ATOM	103	H	ASN	B	11	-23.786	11.771	42.996	1.00	0.00	H
ATOM	104	CA	ASN	B	11	-23.287	11.463	40.905	1.00	0.00	C
ATOM	105	CB	ASN	B	11	-21.772	11.682	40.974	1.00	0.00	C
ATOM	106	CG	ASN	B	11	-21.372	13.152	40.897	1.00	0.00	C
ATOM	107	OD1	ASN	B	11	-20.325	13.549	41.395	1.00	0.00	O
ATOM	108	ND2	ASN	B	11	-22.194	13.962	40.215	1.00	0.00	N
ATOM	109	HD21	ASN	B	11	-21.924	14.910	40.071	1.00	0.00	H
ATOM	110	HD22	ASN	B	11	-23.088	13.679	39.857	1.00	0.00	H
ATOM	111	C	ASN	B	11	-23.602	10.006	40.694	1.00	0.00	C
ATOM	112	O	ASN	B	11	-23.806	9.540	39.581	1.00	0.00	O
ATOM	113	N	ALA	B	12	-23.663	9.296	41.825	1.00	0.00	N
ATOM	114	H	ALA	B	12	-23.471	9.699	42.720	1.00	0.00	H
ATOM	115	CA	ALA	B	12	-24.113	7.915	41.722	1.00	0.00	C
ATOM	116	CB	ALA	B	12	-22.920	6.956	41.792	1.00	0.00	C
ATOM	117	C	ALA	B	12	-25.116	7.550	42.804	1.00	0.00	C
ATOM	118	O	ALA	B	12	-24.806	6.840	43.749	1.00	0.00	O
ATOM	119	N	PRO	B	13	-26.372	8.032	42.632	1.00	0.00	N
ATOM	120	CD	PRO	B	13	-26.891	8.840	41.530	1.00	0.00	C
ATOM	121	CA	PRO	B	13	-27.377	7.752	43.665	1.00	0.00	C
ATOM	122	CB	PRO	B	13	-28.560	8.620	43.220	1.00	0.00	C
ATOM	123	CG	PRO	B	13	-28.402	8.793	41.710	1.00	0.00	C
ATOM	124	C	PRO	B	13	-27.715	6.271	43.824	1.00	0.00	C
ATOM	125	O	PRO	B	13	-28.041	5.788	44.903	1.00	0.00	O
ATOM	126	N	ARG	B	14	-27.625	5.546	42.695	1.00	0.00	N
ATOM	127	H	ARG	B	14	-27.283	5.965	41.857	1.00	0.00	H
ATOM	128	CA	ARG	B	14	-27.912	4.119	42.802	1.00	0.00	C
ATOM	129	CB	ARG	B	14	-28.456	3.564	41.481	1.00	0.00	C
ATOM	130	CG	ARG	B	14	-29.241	2.257	41.664	1.00	0.00	C
ATOM	131	CD	ARG	B	14	-28.870	1.154	40.663	1.00	0.00	C
ATOM	132	NE	ARG	B	14	-27.527	0.631	40.920	1.00	0.00	N
ATOM	133	HE	ARG	B	14	-27.348	0.066	41.735	1.00	0.00	H
ATOM	134	CZ	ARG	B	14	-26.464	0.953	40.157	1.00	0.00	C
ATOM	135	NH1	ARG	B	14	-26.617	1.686	39.054	1.00	0.00	N
ATOM	136	HH11	ARG	B	14	-27.532	1.988	38.785	1.00	0.00	H
ATOM	137	HH12	ARG	B	14	-25.839	1.948	38.487	1.00	0.00	H
ATOM	138	NH2	ARG	B	14	-25.262	0.531	40.522	1.00	0.00	N

ATOM	139	HH21	ARG	B	14	-25.176	-0.029	41.364	1.00	0.00	H
ATOM	140	HH22	ARG	B	14	-24.423	0.730	40.026	1.00	0.00	H
ATOM	141	C	ARG	B	14	-26.726	3.302	43.286	1.00	0.00	C
ATOM	142	O	ARG	B	14	-26.044	2.627	42.524	1.00	0.00	O
ATOM	143	N	ILE	B	15	-26.522	3.387	44.600	1.00	0.00	N
ATOM	144	H	ILE	B	15	-27.066	4.030	45.143	1.00	0.00	H
ATOM	145	CA	ILE	B	15	-25.606	2.443	45.228	1.00	0.00	C
ATOM	146	CB	ILE	B	15	-24.573	3.198	46.079	1.00	0.00	C
ATOM	147	CG2	ILE	B	15	-23.613	2.254	46.805	1.00	0.00	C
ATOM	148	CG1	ILE	B	15	-23.800	4.206	45.227	1.00	0.00	C
ATOM	149	CD1	ILE	B	15	-22.969	5.182	46.062	1.00	0.00	C
ATOM	150	C	ILE	B	15	-26.433	1.490	46.066	1.00	0.00	C
ATOM	151	O	ILE	B	15	-26.863	1.817	47.167	1.00	0.00	O
ATOM	152	N	THR	B	16	-26.694	0.311	45.488	1.00	0.00	N
ATOM	153	H	THR	B	16	-26.250	-0.007	44.648	1.00	0.00	H
ATOM	154	CA	THR	B	16	-27.586	-0.523	46.284	1.00	0.00	C
ATOM	155	CB	THR	B	16	-28.499	-1.393	45.408	1.00	0.00	C
ATOM	156	OG1	THR	B	16	-27.760	-2.341	44.637	1.00	0.00	O
ATOM	157	HG1	THR	B	16	-27.203	-1.856	44.030	1.00	0.00	H
ATOM	158	CG2	THR	B	16	-29.373	-0.530	44.497	1.00	0.00	C
ATOM	159	C	THR	B	16	-26.874	-1.326	47.352	1.00	0.00	C
ATOM	160	O	THR	B	16	-25.747	-1.785	47.192	1.00	0.00	O
ATOM	161	N	PHE	B	17	-27.588	-1.446	48.472	1.00	0.00	N
ATOM	162	H	PHE	B	17	-28.501	-1.051	48.559	1.00	0.00	H
ATOM	163	CA	PHE	B	17	-27.111	-2.353	49.504	1.00	0.00	C
ATOM	164	CB	PHE	B	17	-26.804	-1.609	50.808	1.00	0.00	C
ATOM	165	CG	PHE	B	17	-25.777	-0.520	50.601	1.00	0.00	C
ATOM	166	CD1	PHE	B	17	-26.186	0.830	50.659	1.00	0.00	C
ATOM	167	CD2	PHE	B	17	-24.429	-0.866	50.366	1.00	0.00	C
ATOM	168	CE1	PHE	B	17	-25.229	1.850	50.497	1.00	0.00	C
ATOM	169	CE2	PHE	B	17	-23.470	0.152	50.203	1.00	0.00	C
ATOM	170	CZ	PHE	B	17	-23.879	1.500	50.282	1.00	0.00	C
ATOM	171	C	PHE	B	17	-28.215	-3.347	49.743	1.00	0.00	C
ATOM	172	O	PHE	B	17	-29.385	-2.980	49.732	1.00	0.00	O
ATOM	173	N	GLY	B	18	-27.813	-4.602	49.944	1.00	0.00	N
ATOM	174	H	GLY	B	18	-26.830	-4.801	49.916	1.00	0.00	H
ATOM	175	CA	GLY	B	18	-28.809	-5.607	50.303	1.00	0.00	C
ATOM	176	C	GLY	B	18	-29.710	-6.064	49.171	1.00	0.00	C
ATOM	177	O	GLY	B	18	-29.541	-7.134	48.603	1.00	0.00	O
ATOM	178	N	GLY	B	19	-30.711	-5.226	48.880	1.00	0.00	N
ATOM	179	H	GLY	B	19	-30.748	-4.345	49.357	1.00	0.00	H
ATOM	180	CA	GLY	B	19	-31.762	-5.704	47.985	1.00	0.00	C
ATOM	181	C	GLY	B	19	-32.683	-6.670	48.714	1.00	0.00	C
ATOM	182	O	GLY	B	19	-32.503	-7.885	48.681	1.00	0.00	O
ATOM	183	N	PRO	B	20	-33.684	-6.055	49.390	1.00	0.00	N
ATOM	184	CD	PRO	B	20	-34.127	-4.683	49.162	1.00	0.00	C
ATOM	185	CA	PRO	B	20	-34.423	-6.698	50.487	1.00	0.00	C
ATOM	186	CB	PRO	B	20	-35.475	-5.640	50.860	1.00	0.00	C
ATOM	187	CG	PRO	B	20	-35.555	-4.675	49.682	1.00	0.00	C
ATOM	188	C	PRO	B	20	-35.007	-8.083	50.255	1.00	0.00	C
ATOM	189	O	PRO	B	20	-34.900	-8.702	49.201	1.00	0.00	O
ATOM	190	N	SER	B	21	-35.641	-8.552	51.331	1.00	0.00	N
ATOM	191	H	SER	B	21	-35.732	-8.019	52.170	1.00	0.00	H
ATOM	192	CA	SER	B	21	-36.274	-9.861	51.259	1.00	0.00	C
ATOM	193	CB	SER	B	21	-36.187	-10.520	52.637	1.00	0.00	C
ATOM	194	OG	SER	B	21	-34.805	-10.538	53.039	1.00	0.00	O
ATOM	195	HG	SER	B	21	-34.387	-11.101	52.387	1.00	0.00	H
ATOM	196	C	SER	B	21	-37.681	-9.836	50.687	1.00	0.00	C
ATOM	197	O	SER	B	21	-38.671	-10.066	51.367	1.00	0.00	O

```
ATOM    198  N   ASP B  22     -37.705  -9.553  49.373  1.00  0.00           N
ATOM    199  H   ASP B  22     -36.857  -9.284  48.916  1.00  0.00           H
ATOM    200  CA  ASP B  22     -38.949  -9.579  48.589  1.00  0.00           C
ATOM    201  CB  ASP B  22     -38.722  -9.237  47.096  1.00  0.00           C
ATOM    202  CG  ASP B  22     -37.252  -9.098  46.713  1.00  0.00           C
ATOM    203  OD1 ASP B  22     -36.641 -10.074  46.289  1.00  0.00           O
ATOM    204  OD2 ASP B  22     -36.685  -8.021  46.870  1.00  0.00           O
ATOM    205  C   ASP B  22     -39.731 -10.879  48.717  1.00  0.00           C
ATOM    206  O   ASP B  22     -40.949 -10.917  48.830  1.00  0.00           O
ATOM    207  N   SER B  23     -38.948 -11.959  48.755  1.00  0.00           N
ATOM    208  H   SER B  23     -37.965 -11.864  48.609  1.00  0.00           H
ATOM    209  CA  SER B  23     -39.469 -13.150  49.412  1.00  0.00           C
ATOM    210  CB  SER B  23     -39.435 -14.339  48.450  1.00  0.00           C
ATOM    211  OG  SER B  23     -38.099 -14.561  47.969  1.00  0.00           O
ATOM    212  HG  SER B  23     -37.805 -13.728  47.606  1.00  0.00           H
ATOM    213  C   SER B  23     -38.581 -13.395  50.611  1.00  0.00           C
ATOM    214  O   SER B  23     -37.488 -12.839  50.677  1.00  0.00           O
ATOM    215  N   THR B  24     -39.012 -14.280  51.516  1.00  0.00           N
ATOM    216  H   THR B  24     -39.927 -14.690  51.526  1.00  0.00           H
ATOM    217  CA  THR B  24     -38.035 -14.709  52.519  1.00  0.00           C
ATOM    218  CB  THR B  24     -38.744 -15.584  53.544  1.00  0.00           C
ATOM    219  OG1 THR B  24     -39.825 -16.286  52.913  1.00  0.00           O
ATOM    220  HG1 THR B  24     -40.354 -16.657  53.622  1.00  0.00           H
ATOM    221  CG2 THR B  24     -39.264 -14.724  54.697  1.00  0.00           C
ATOM    222  C   THR B  24     -36.797 -15.390  51.936  1.00  0.00           C
ATOM    223  O   THR B  24     -35.681 -15.264  52.426  1.00  0.00           O
ATOM    224  N   GLY B  25     -37.042 -16.068  50.800  1.00  0.00           N
ATOM    225  H   GLY B  25     -37.990 -16.181  50.503  1.00  0.00           H
ATOM    226  CA  GLY B  25     -35.926 -16.625  50.032  1.00  0.00           C
ATOM    227  C   GLY B  25     -34.919 -15.603  49.515  1.00  0.00           C
ATOM    228  O   GLY B  25     -33.742 -15.894  49.309  1.00  0.00           O
ATOM    229  N   SER B  26     -35.423 -14.374  49.317  1.00  0.00           N
ATOM    230  H   SER B  26     -36.391 -14.180  49.482  1.00  0.00           H
ATOM    231  CA  SER B  26     -34.528 -13.287  48.935  1.00  0.00           C
ATOM    232  CB  SER B  26     -35.322 -12.090  48.429  1.00  0.00           C
ATOM    233  OG  SER B  26     -36.375 -12.538  47.560  1.00  0.00           O
ATOM    234  HG  SER B  26     -36.507 -11.824  46.931  1.00  0.00           H
ATOM    235  C   SER B  26     -33.550 -12.867  50.017  1.00  0.00           C
ATOM    236  O   SER B  26     -33.744 -11.947  50.805  1.00  0.00           O
ATOM    237  N   ASN B  27     -32.440 -13.600  49.975  1.00  0.00           N
ATOM    238  H   ASN B  27     -32.459 -14.348  49.310  1.00  0.00           H
ATOM    239  CA  ASN B  27     -31.332 -13.443  50.917  1.00  0.00           C
ATOM    240  CB  ASN B  27     -30.527 -14.751  50.977  1.00  0.00           C
ATOM    241  CG  ASN B  27     -30.048 -15.160  49.590  1.00  0.00           C
ATOM    242  OD1 ASN B  27     -29.068 -14.659  49.050  1.00  0.00           O
ATOM    243  ND2 ASN B  27     -30.809 -16.100  49.020  1.00  0.00           N
ATOM    244 HD21 ASN B  27     -30.545 -16.509  48.149  1.00  0.00           H
ATOM    245 HD22 ASN B  27     -31.662 -16.419  49.445  1.00  0.00           H
ATOM    246  C   ASN B  27     -30.419 -12.254  50.656  1.00  0.00           C
ATOM    247  O   ASN B  27     -29.199 -12.380  50.646  1.00  0.00           O
ATOM    248  N   GLN B  28     -31.068 -11.095  50.436  1.00  0.00           N
ATOM    249  H   GLN B  28     -32.059 -11.088  50.559  1.00  0.00           H
ATOM    250  CA  GLN B  28     -30.381  -9.823  50.198  1.00  0.00           C
ATOM    251  CB  GLN B  28     -30.063  -9.130  51.522  1.00  0.00           C
ATOM    252  CG  GLN B  28     -31.361  -8.802  52.264  1.00  0.00           C
ATOM    253  CD  GLN B  28     -31.062  -8.023  53.524  1.00  0.00           C
ATOM    254  OE1 GLN B  28     -30.014  -7.415  53.689  1.00  0.00           O
ATOM    255  NE2 GLN B  28     -32.056  -8.057  54.412  1.00  0.00           N
ATOM    256 HE21 GLN B  28     -31.948  -7.509  55.244  1.00  0.00           H
```

```
ATOM    257 HE22 GLN B  28      -32.896  -8.583  54.282  1.00  0.00           H
ATOM    258  C   GLN B  28      -29.201  -9.864  49.243  1.00  0.00           C
ATOM    259  O   GLN B  28      -28.027  -9.858  49.600  1.00  0.00           O
ATOM    260  N   ASN B  29      -29.593  -9.952  47.968  1.00  0.00           N
ATOM    261  H   ASN B  29      -30.530  -9.677  47.765  1.00  0.00           H
ATOM    262  CA  ASN B  29      -28.569 -10.131  46.943  1.00  0.00           C
ATOM    263  CB  ASN B  29      -29.096 -11.010  45.810  1.00  0.00           C
ATOM    264  CG  ASN B  29      -28.483 -12.385  45.960  1.00  0.00           C
ATOM    265  OD1 ASN B  29      -27.269 -12.563  46.057  1.00  0.00           O
ATOM    266  ND2 ASN B  29      -29.385 -13.366  46.040  1.00  0.00           N
ATOM    267 HD21 ASN B  29      -29.108 -14.303  46.251  1.00  0.00           H
ATOM    268 HD22 ASN B  29      -30.360 -13.189  45.898  1.00  0.00           H
ATOM    269  C   ASN B  29      -27.962  -8.846  46.416  1.00  0.00           C
ATOM    270  O   ASN B  29      -28.179  -8.443  45.281  1.00  0.00           O
ATOM    271  N   GLY B  30      -27.175  -8.240  47.310  1.00  0.00           N
ATOM    272  H   GLY B  30      -27.154  -8.620  48.235  1.00  0.00           H
ATOM    273  CA  GLY B  30      -26.610  -6.924  47.021  1.00  0.00           C
ATOM    274  C   GLY B  30      -25.606  -6.827  45.886  1.00  0.00           C
ATOM    275  O   GLY B  30      -24.973  -7.788  45.452  1.00  0.00           O
ATOM    276  N   GLU B  31      -25.515  -5.571  45.436  1.00  0.00           N
ATOM    277  H   GLU B  31      -26.021  -4.868  45.935  1.00  0.00           H
ATOM    278  CA  GLU B  31      -24.749  -5.190  44.253  1.00  0.00           C
ATOM    279  CB  GLU B  31      -24.998  -3.705  44.029  1.00  0.00           C
ATOM    280  CG  GLU B  31      -25.231  -3.261  42.590  1.00  0.00           C
ATOM    281  CD  GLU B  31      -25.050  -1.761  42.508  1.00  0.00           C
ATOM    282  OE1 GLU B  31      -25.973  -1.008  42.808  1.00  0.00           O
ATOM    283  OE2 GLU B  31      -23.976  -1.324  42.116  1.00  0.00           O
ATOM    284  C   GLU B  31      -23.249  -5.441  44.342  1.00  0.00           C
ATOM    285  O   GLU B  31      -22.657  -5.477  45.416  1.00  0.00           O
ATOM    286  N   ARG B  32      -22.636  -5.564  43.149  1.00  0.00           N
ATOM    287  H   ARG B  32      -23.182  -5.502  42.311  1.00  0.00           H
ATOM    288  CA  ARG B  32      -21.170  -5.560  43.120  1.00  0.00           C
ATOM    289  CB  ARG B  32      -20.575  -5.930  41.755  1.00  0.00           C
ATOM    290  CG  ARG B  32      -21.073  -7.179  41.026  1.00  0.00           C
ATOM    291  CD  ARG B  32      -20.239  -7.383  39.755  1.00  0.00           C
ATOM    292  NE  ARG B  32      -20.881  -8.296  38.811  1.00  0.00           N
ATOM    293  HE  ARG B  32      -21.885  -8.325  38.783  1.00  0.00           H
ATOM    294  CZ  ARG B  32      -20.184  -8.971  37.871  1.00  0.00           C
ATOM    295  NH1 ARG B  32      -18.856  -8.896  37.798  1.00  0.00           N
ATOM    296 HH11 ARG B  32      -18.334  -8.384  38.478  1.00  0.00           H
ATOM    297 HH12 ARG B  32      -18.350  -9.346  37.049  1.00  0.00           H
ATOM    298  NH2 ARG B  32      -20.853  -9.711  36.998  1.00  0.00           N
ATOM    299 HH21 ARG B  32      -21.860  -9.686  37.014  1.00  0.00           H
ATOM    300 HH22 ARG B  32      -20.389 -10.280  36.318  1.00  0.00           H
ATOM    301  C   ARG B  32      -20.594  -4.199  43.473  1.00  0.00           C
ATOM    302  O   ARG B  32      -19.641  -4.062  44.237  1.00  0.00           O
ATOM    303  N   SER B  33      -21.209  -3.186  42.854  1.00  0.00           N
ATOM    304  H   SER B  33      -21.982  -3.338  42.237  1.00  0.00           H
ATOM    305  CA  SER B  33      -20.675  -1.838  43.038  1.00  0.00           C
ATOM    306  CB  SER B  33      -20.831  -1.041  41.738  1.00  0.00           C
ATOM    307  OG  SER B  33      -21.554  -1.823  40.773  1.00  0.00           O
ATOM    308  HG  SER B  33      -22.479  -1.705  41.001  1.00  0.00           H
ATOM    309  C   SER B  33      -21.222  -1.106  44.256  1.00  0.00           C
ATOM    310  O   SER B  33      -20.570  -0.260  44.867  1.00  0.00           O
ATOM    311  N   GLY B  34      -22.441  -1.512  44.622  1.00  0.00           N
ATOM    312  H   GLY B  34      -23.012  -1.999  43.959  1.00  0.00           H
ATOM    313  CA  GLY B  34      -22.913  -1.251  45.978  1.00  0.00           C
ATOM    314  C   GLY B  34      -22.290  -2.214  46.969  1.00  0.00           C
ATOM    315  O   GLY B  34      -21.069  -2.258  47.110  1.00  0.00           O
```

```
ATOM    316  N   ALA B  35      -23.165  -2.988  47.623  1.00  0.00           N
ATOM    317  H   ALA B  35      -24.153  -2.860  47.492  1.00  0.00           H
ATOM    318  CA  ALA B  35      -22.703  -4.056  48.506  1.00  0.00           C
ATOM    319  CB  ALA B  35      -22.074  -3.503  49.794  1.00  0.00           C
ATOM    320  C   ALA B  35      -23.850  -4.959  48.905  1.00  0.00           C
ATOM    321  O   ALA B  35      -25.014  -4.677  48.646  1.00  0.00           O
ATOM    322  N   ARG B  36      -23.466  -6.056  49.579  1.00  0.00           N
ATOM    323  H   ARG B  36      -22.495  -6.224  49.727  1.00  0.00           H
ATOM    324  CA  ARG B  36      -24.500  -6.780  50.313  1.00  0.00           C
ATOM    325  CB  ARG B  36      -24.099  -8.245  50.530  1.00  0.00           C
ATOM    326  CG  ARG B  36      -24.662  -9.195  49.475  1.00  0.00           C
ATOM    327  CD  ARG B  36      -24.375 -10.664  49.794  1.00  0.00           C
ATOM    328  NE  ARG B  36      -25.216 -11.549  48.988  1.00  0.00           N
ATOM    329  HE  ARG B  36      -25.039 -11.633  48.010  1.00  0.00           H
ATOM    330  CZ  ARG B  36      -26.300 -12.142  49.537  1.00  0.00           C
ATOM    331  NH1 ARG B  36      -26.590 -11.985  50.823  1.00  0.00           N
ATOM    332 HH11 ARG B  36      -25.979 -11.509  51.450  1.00  0.00           H
ATOM    333 HH12 ARG B  36      -27.475 -12.324  51.161  1.00  0.00           H
ATOM    334  NH2 ARG B  36      -27.099 -12.880  48.780  1.00  0.00           N
ATOM    335 HH21 ARG B  36      -26.943 -13.000  47.797  1.00  0.00           H
ATOM    336 HH22 ARG B  36      -27.907 -13.338  49.179  1.00  0.00           H
ATOM    337  C   ARG B  36      -24.841  -6.073  51.617  1.00  0.00           C
ATOM    338  O   ARG B  36      -25.674  -5.179  51.655  1.00  0.00           O
ATOM    339  N   SER B  37      -24.158  -6.495  52.690  1.00  0.00           N
ATOM    340  H   SER B  37      -23.368  -7.098  52.630  1.00  0.00           H
ATOM    341  CA  SER B  37      -24.473  -5.821  53.942  1.00  0.00           C
ATOM    342  CB  SER B  37      -24.231  -6.763  55.123  1.00  0.00           C
ATOM    343  OG  SER B  37      -24.842  -6.236  56.304  1.00  0.00           O
ATOM    344  HG  SER B  37      -24.684  -6.874  56.996  1.00  0.00           H
ATOM    345  C   SER B  37      -23.691  -4.533  54.089  1.00  0.00           C
ATOM    346  O   SER B  37      -22.634  -4.351  53.491  1.00  0.00           O
ATOM    347  N   LYS B  38      -24.274  -3.660  54.919  1.00  0.00           N
ATOM    348  H   LYS B  38      -25.107  -3.966  55.381  1.00  0.00           H
ATOM    349  CA  LYS B  38      -23.620  -2.406  55.266  1.00  0.00           C
ATOM    350  CB  LYS B  38      -23.700  -1.403  54.095  1.00  0.00           C
ATOM    351  CG  LYS B  38      -22.829  -0.151  54.257  1.00  0.00           C
ATOM    352  CD  LYS B  38      -21.331  -0.448  54.285  1.00  0.00           C
ATOM    353  CE  LYS B  38      -20.516   0.797  54.637  1.00  0.00           C
ATOM    354  NZ  LYS B  38      -19.080   0.500  54.573  1.00  0.00           N
ATOM    355  HZ1 LYS B  38      -18.783   0.370  53.590  1.00  0.00           H
ATOM    356  HZ2 LYS B  38      -18.538   1.326  54.910  1.00  0.00           H
ATOM    357  HZ3 LYS B  38      -18.835  -0.349  55.124  1.00  0.00           H
ATOM    358  C   LYS B  38      -24.170  -1.877  56.586  1.00  0.00           C
ATOM    359  O   LYS B  38      -23.752  -2.311  57.653  1.00  0.00           O
ATOM    360  N   GLN B  39      -25.130  -0.944  56.468  1.00  0.00           N
ATOM    361  H   GLN B  39      -25.473  -0.646  55.576  1.00  0.00           H
ATOM    362  CA  GLN B  39      -25.693  -0.219  57.603  1.00  0.00           C
ATOM    363  CB  GLN B  39      -24.600   0.665  58.241  1.00  0.00           C
ATOM    364  CG  GLN B  39      -24.980   2.012  58.875  1.00  0.00           C
ATOM    365  CD  GLN B  39      -25.964   1.860  60.018  1.00  0.00           C
ATOM    366  OE1 GLN B  39      -27.110   2.280  59.953  1.00  0.00           O
ATOM    367  NE2 GLN B  39      -25.458   1.276  61.091  1.00  0.00           N
ATOM    368 HE21 GLN B  39      -26.028   1.132  61.903  1.00  0.00           H
ATOM    369 HE22 GLN B  39      -24.522   0.926  61.122  1.00  0.00           H
ATOM    370  C   GLN B  39      -26.836   0.592  57.033  1.00  0.00           C
ATOM    371  O   GLN B  39      -26.660   1.273  56.030  1.00  0.00           O
ATOM    372  N   ARG B  40      -27.987   0.436  57.696  1.00  0.00           N
ATOM    373  H   ARG B  40      -28.004  -0.156  58.504  1.00  0.00           H
ATOM    374  CA  ARG B  40      -29.216   1.192  57.448  1.00  0.00           C
```

ATOM	375	CB	ARG	B	40	-29.860	0.910	56.083	1.00	0.00	C
ATOM	376	CG	ARG	B	40	-29.495	1.859	54.940	1.00	0.00	C
ATOM	377	CD	ARG	B	40	-30.221	1.487	53.647	1.00	0.00	C
ATOM	378	NE	ARG	B	40	-29.821	2.361	52.546	1.00	0.00	N
ATOM	379	HE	ARG	B	40	-28.840	2.417	52.356	1.00	0.00	H
ATOM	380	CZ	ARG	B	40	-30.743	3.056	51.844	1.00	0.00	C
ATOM	381	NH1	ARG	B	40	-32.041	2.947	52.120	1.00	0.00	N
ATOM	382	HH11	ARG	B	40	-32.375	2.289	52.806	1.00	0.00	H
ATOM	383	HH12	ARG	B	40	-32.740	3.503	51.676	1.00	0.00	H
ATOM	384	NH2	ARG	B	40	-30.337	3.859	50.862	1.00	0.00	N
ATOM	385	HH21	ARG	B	40	-29.365	3.940	50.640	1.00	0.00	H
ATOM	386	HH22	ARG	B	40	-30.989	4.393	50.323	1.00	0.00	H
ATOM	387	C	ARG	B	40	-30.185	0.756	58.523	1.00	0.00	C
ATOM	388	O	ARG	B	40	-29.977	-0.257	59.188	1.00	0.00	O
ATOM	389	N	ARG	B	41	-31.247	1.547	58.681	1.00	0.00	N
ATOM	390	H	ARG	B	41	-31.378	2.334	58.078	1.00	0.00	H
ATOM	391	CA	ARG	B	41	-32.203	1.133	59.701	1.00	0.00	C
ATOM	392	CB	ARG	B	41	-32.814	2.360	60.389	1.00	0.00	C
ATOM	393	CG	ARG	B	41	-31.702	3.272	60.909	1.00	0.00	C
ATOM	394	CD	ARG	B	41	-32.170	4.447	61.767	1.00	0.00	C
ATOM	395	NE	ARG	B	41	-31.137	5.482	61.770	1.00	0.00	N
ATOM	396	HE	ARG	B	41	-30.181	5.202	61.878	1.00	0.00	H
ATOM	397	CZ	ARG	B	41	-31.434	6.727	61.345	1.00	0.00	C
ATOM	398	NH1	ARG	B	41	-32.703	7.088	61.185	1.00	0.00	N
ATOM	399	HH11	ARG	B	41	-33.441	6.518	61.540	1.00	0.00	H
ATOM	400	HH12	ARG	B	41	-32.943	7.919	60.673	1.00	0.00	H
ATOM	401	NH2	ARG	B	41	-30.457	7.582	61.067	1.00	0.00	N
ATOM	402	HH21	ARG	B	41	-29.498	7.280	61.132	1.00	0.00	H
ATOM	403	HH22	ARG	B	41	-30.635	8.520	60.776	1.00	0.00	H
ATOM	404	C	ARG	B	41	-33.221	0.122	59.187	1.00	0.00	C
ATOM	405	O	ARG	B	41	-33.778	0.243	58.097	1.00	0.00	O
ATOM	406	N	PRO	B	42	-33.410	-0.932	60.021	1.00	0.00	N
ATOM	407	CD	PRO	B	42	-32.859	-1.079	61.367	1.00	0.00	C
ATOM	408	CA	PRO	B	42	-34.145	-2.127	59.589	1.00	0.00	C
ATOM	409	CB	PRO	B	42	-33.664	-3.168	60.605	1.00	0.00	C
ATOM	410	CG	PRO	B	42	-33.444	-2.381	61.892	1.00	0.00	C
ATOM	411	C	PRO	B	42	-35.662	-2.017	59.591	1.00	0.00	C
ATOM	412	O	PRO	B	42	-36.343	-2.973	59.937	1.00	0.00	O
ATOM	413	N	GLN	B	43	-36.187	-0.846	59.195	1.00	0.00	N
ATOM	414	H	GLN	B	43	-35.593	-0.124	58.835	1.00	0.00	H
ATOM	415	CA	GLN	B	43	-37.629	-0.640	59.381	1.00	0.00	C
ATOM	416	CB	GLN	B	43	-38.022	0.782	58.987	1.00	0.00	C
ATOM	417	CG	GLN	B	43	-37.401	1.828	59.912	1.00	0.00	C
ATOM	418	CD	GLN	B	43	-36.658	2.832	59.064	1.00	0.00	C
ATOM	419	OE1	GLN	B	43	-35.535	2.604	58.635	1.00	0.00	O
ATOM	420	NE2	GLN	B	43	-37.351	3.940	58.793	1.00	0.00	N
ATOM	421	HE21	GLN	B	43	-36.980	4.607	58.144	1.00	0.00	H
ATOM	422	HE22	GLN	B	43	-38.247	4.106	59.204	1.00	0.00	H
ATOM	423	C	GLN	B	43	-38.572	-1.653	58.737	1.00	0.00	C
ATOM	424	O	GLN	B	43	-39.706	-1.819	59.160	1.00	0.00	O
ATOM	425	N	GLY	B	44	-38.048	-2.332	57.707	1.00	0.00	N
ATOM	426	H	GLY	B	44	-37.137	-2.131	57.360	1.00	0.00	H
ATOM	427	CA	GLY	B	44	-38.850	-3.427	57.171	1.00	0.00	C
ATOM	428	C	GLY	B	44	-38.047	-4.687	56.912	1.00	0.00	C
ATOM	429	O	GLY	B	44	-38.341	-5.448	56.002	1.00	0.00	O
ATOM	430	N	LEU	B	45	-36.992	-4.860	57.727	1.00	0.00	N
ATOM	431	H	LEU	B	45	-36.850	-4.261	58.517	1.00	0.00	H
ATOM	432	CA	LEU	B	45	-36.108	-6.001	57.472	1.00	0.00	C
ATOM	433	CB	LEU	B	45	-34.924	-5.610	56.584	1.00	0.00	C

ATOM	434	CG	LEU	B	45	-35.183	-5.605	55.079	1.00	0.00	C
ATOM	435	CD1	LEU	B	45	-33.928	-5.189	54.316	1.00	0.00	C
ATOM	436	CD2	LEU	B	45	-35.719	-6.948	54.582	1.00	0.00	C
ATOM	437	C	LEU	B	45	-35.567	-6.658	58.730	1.00	0.00	C
ATOM	438	O	LEU	B	45	-34.510	-6.296	59.242	1.00	0.00	O
ATOM	439	N	PRO	B	46	-36.333	-7.656	59.226	1.00	0.00	N
ATOM	440	CD	PRO	B	46	-37.642	-8.079	58.748	1.00	0.00	C
ATOM	441	CA	PRO	B	46	-35.890	-8.426	60.393	1.00	0.00	C
ATOM	442	CB	PRO	B	46	-37.192	-9.087	60.876	1.00	0.00	C
ATOM	443	CG	PRO	B	46	-38.335	-8.524	60.026	1.00	0.00	C
ATOM	444	C	PRO	B	46	-34.832	-9.469	60.045	1.00	0.00	C
ATOM	445	O	PRO	B	46	-35.050	-10.669	60.155	1.00	0.00	O
ATOM	446	N	ASN	B	47	-33.668	-8.980	59.602	1.00	0.00	N
ATOM	447	H	ASN	B	47	-33.511	-7.993	59.556	1.00	0.00	H
ATOM	448	CA	ASN	B	47	-32.706	-9.968	59.117	1.00	0.00	C
ATOM	449	CB	ASN	B	47	-32.420	-9.820	57.612	1.00	0.00	C
ATOM	450	CG	ASN	B	47	-33.672	-9.777	56.753	1.00	0.00	C
ATOM	451	OD1	ASN	B	47	-34.545	-8.936	56.912	1.00	0.00	O
ATOM	452	ND2	ASN	B	47	-33.691	-10.680	55.767	1.00	0.00	N
ATOM	453	HD21	ASN	B	47	-34.425	-10.638	55.083	1.00	0.00	H
ATOM	454	HD22	ASN	B	47	-33.002	-11.395	55.683	1.00	0.00	H
ATOM	455	C	ASN	B	47	-31.412	-9.894	59.903	1.00	0.00	C
ATOM	456	O	ASN	B	47	-31.143	-8.939	60.628	1.00	0.00	O
ATOM	457	N	ASN	B	48	-30.600	-10.941	59.748	1.00	0.00	N
ATOM	458	H	ASN	B	48	-30.795	-11.664	59.089	1.00	0.00	H
ATOM	459	CA	ASN	B	48	-29.360	-10.909	60.522	1.00	0.00	C
ATOM	460	CB	ASN	B	48	-29.061	-12.304	61.076	1.00	0.00	C
ATOM	461	CG	ASN	B	48	-28.016	-12.192	62.162	1.00	0.00	C
ATOM	462	OD1	ASN	B	48	-27.763	-11.124	62.703	1.00	0.00	O
ATOM	463	ND2	ASN	B	48	-27.423	-13.348	62.470	1.00	0.00	N
ATOM	464	HD21	ASN	B	48	-26.730	-13.365	63.193	1.00	0.00	H
ATOM	465	HD22	ASN	B	48	-27.645	-14.198	61.997	1.00	0.00	H
ATOM	466	C	ASN	B	48	-28.182	-10.348	59.741	1.00	0.00	C
ATOM	467	O	ASN	B	48	-27.361	-11.090	59.216	1.00	0.00	O
ATOM	468	N	THR	B	49	-28.154	-9.010	59.653	1.00	0.00	N
ATOM	469	H	THR	B	49	-28.868	-8.466	60.088	1.00	0.00	H
ATOM	470	CA	THR	B	49	-27.326	-8.497	58.562	1.00	0.00	C
ATOM	471	CB	THR	B	49	-28.244	-8.012	57.434	1.00	0.00	C
ATOM	472	OG1	THR	B	49	-29.561	-8.563	57.581	1.00	0.00	O
ATOM	473	HG1	THR	B	49	-30.092	-8.090	56.939	1.00	0.00	H
ATOM	474	CG2	THR	B	49	-27.675	-8.321	56.050	1.00	0.00	C
ATOM	475	C	THR	B	49	-26.263	-7.447	58.870	1.00	0.00	C
ATOM	476	O	THR	B	49	-25.063	-7.640	58.666	1.00	0.00	O
ATOM	477	N	ALA	B	50	-26.759	-6.279	59.297	1.00	0.00	N
ATOM	478	H	ALA	B	50	-27.699	-6.181	59.625	1.00	0.00	H
ATOM	479	CA	ALA	B	50	-25.898	-5.110	59.150	1.00	0.00	C
ATOM	480	CB	ALA	B	50	-26.569	-4.067	58.253	1.00	0.00	C
ATOM	481	C	ALA	B	50	-25.468	-4.453	60.441	1.00	0.00	C
ATOM	482	O	ALA	B	50	-26.022	-4.683	61.512	1.00	0.00	O
ATOM	483	N	SER	B	51	-24.426	-3.625	60.253	1.00	0.00	N
ATOM	484	H	SER	B	51	-24.132	-3.435	59.315	1.00	0.00	H
ATOM	485	CA	SER	B	51	-23.768	-2.915	61.347	1.00	0.00	C
ATOM	486	CB	SER	B	51	-22.675	-2.027	60.744	1.00	0.00	C
ATOM	487	OG	SER	B	51	-21.828	-1.460	61.751	1.00	0.00	O
ATOM	488	HG	SER	B	51	-21.888	-0.510	61.639	1.00	0.00	H
ATOM	489	C	SER	B	51	-24.716	-2.064	62.163	1.00	0.00	C
ATOM	490	O	SER	B	51	-25.576	-1.390	61.616	1.00	0.00	O
ATOM	491	N	TRP	B	52	-24.488	-2.090	63.483	1.00	0.00	N
ATOM	492	H	TRP	B	52	-23.822	-2.755	63.822	1.00	0.00	H

ATOM	493	CA	TRP	B	52	-25.220	-1.194	64.377	1.00	0.00	C
ATOM	494	CB	TRP	B	52	-24.849	-1.474	65.832	1.00	0.00	C
ATOM	495	CG	TRP	B	52	-25.859	-2.383	66.490	1.00	0.00	C
ATOM	496	CD2	TRP	B	52	-26.409	-2.249	67.817	1.00	0.00	C
ATOM	497	CE2	TRP	B	52	-27.301	-3.352	68.020	1.00	0.00	C
ATOM	498	CE3	TRP	B	52	-26.220	-1.300	68.845	1.00	0.00	C
ATOM	499	CD1	TRP	B	52	-26.446	-3.544	65.961	1.00	0.00	C
ATOM	500	NE1	TRP	B	52	-27.295	-4.112	66.858	1.00	0.00	N
ATOM	501	HE1	TRP	B	52	-27.849	-4.912	66.713	1.00	0.00	H
ATOM	502	CZ2	TRP	B	52	-27.981	-3.492	69.248	1.00	0.00	C
ATOM	503	CZ3	TRP	B	52	-26.906	-1.449	70.068	1.00	0.00	C
ATOM	504	CH2	TRP	B	52	-27.778	-2.540	70.270	1.00	0.00	C
ATOM	505	C	TRP	B	52	-25.027	0.282	64.077	1.00	0.00	C
ATOM	506	O	TRP	B	52	-25.976	1.038	63.910	1.00	0.00	O
ATOM	507	N	PHE	B	53	-23.743	0.652	64.022	1.00	0.00	N
ATOM	508	H	PHE	B	53	-23.020	-0.035	64.076	1.00	0.00	H
ATOM	509	CA	PHE	B	53	-23.419	2.057	63.781	1.00	0.00	C
ATOM	510	CB	PHE	B	53	-22.528	2.594	64.910	1.00	0.00	C
ATOM	511	CG	PHE	B	53	-23.326	2.966	66.144	1.00	0.00	C
ATOM	512	CD1	PHE	B	53	-24.096	2.001	66.831	1.00	0.00	C
ATOM	513	CD2	PHE	B	53	-23.272	4.300	66.599	1.00	0.00	C
ATOM	514	CE1	PHE	B	53	-24.825	2.376	67.975	1.00	0.00	C
ATOM	515	CE2	PHE	B	53	-23.995	4.681	67.746	1.00	0.00	C
ATOM	516	CZ	PHE	B	53	-24.767	3.713	68.420	1.00	0.00	C
ATOM	517	C	PHE	B	53	-22.737	2.198	62.435	1.00	0.00	C
ATOM	518	O	PHE	B	53	-22.581	1.214	61.712	1.00	0.00	O
ATOM	519	N	THR	B	54	-22.315	3.434	62.125	1.00	0.00	N
ATOM	520	H	THR	B	54	-22.648	4.231	62.633	1.00	0.00	H
ATOM	521	CA	THR	B	54	-21.419	3.623	60.984	1.00	0.00	C
ATOM	522	CB	THR	B	54	-20.996	5.095	60.903	1.00	0.00	C
ATOM	523	OG1	THR	B	54	-22.079	5.954	61.293	1.00	0.00	O
ATOM	524	HG1	THR	B	54	-22.000	6.736	60.758	1.00	0.00	H
ATOM	525	CG2	THR	B	54	-20.474	5.474	59.514	1.00	0.00	C
ATOM	526	C	THR	B	54	-20.190	2.729	61.081	1.00	0.00	C
ATOM	527	O	THR	B	54	-19.635	2.548	62.154	1.00	0.00	O
ATOM	528	N	ALA	B	55	-19.819	2.149	59.929	1.00	0.00	N
ATOM	529	H	ALA	B	55	-20.277	2.396	59.080	1.00	0.00	H
ATOM	530	CA	ALA	B	55	-18.685	1.227	59.971	1.00	0.00	C
ATOM	531	CB	ALA	B	55	-18.497	0.553	58.613	1.00	0.00	C
ATOM	532	C	ALA	B	55	-17.382	1.900	60.354	1.00	0.00	C
ATOM	533	O	ALA	B	55	-17.166	3.075	60.078	1.00	0.00	O
ATOM	534	N	LEU	B	56	-16.530	1.098	60.992	1.00	0.00	N
ATOM	535	H	LEU	B	56	-16.776	0.137	61.122	1.00	0.00	H
ATOM	536	CA	LEU	B	56	-15.211	1.608	61.347	1.00	0.00	C
ATOM	537	CB	LEU	B	56	-14.878	1.082	62.745	1.00	0.00	C
ATOM	538	CG	LEU	B	56	-14.021	1.996	63.616	1.00	0.00	C
ATOM	539	CD1	LEU	B	56	-14.518	1.995	65.061	1.00	0.00	C
ATOM	540	CD2	LEU	B	56	-12.534	1.656	63.522	1.00	0.00	C
ATOM	541	C	LEU	B	56	-14.211	1.201	60.277	1.00	0.00	C
ATOM	542	O	LEU	B	56	-14.533	0.401	59.402	1.00	0.00	O
ATOM	543	N	THR	B	57	-13.005	1.773	60.340	1.00	0.00	N
ATOM	544	H	THR	B	57	-12.786	2.485	61.012	1.00	0.00	H
ATOM	545	CA	THR	B	57	-12.017	1.422	59.327	1.00	0.00	C
ATOM	546	CB	THR	B	57	-12.225	2.277	58.073	1.00	0.00	C
ATOM	547	OG1	THR	B	57	-13.610	2.309	57.703	1.00	0.00	O
ATOM	548	HG1	THR	B	57	-13.953	1.439	57.890	1.00	0.00	H
ATOM	549	CG2	THR	B	57	-11.366	1.782	56.913	1.00	0.00	C
ATOM	550	C	THR	B	57	-10.581	1.553	59.807	1.00	0.00	C
ATOM	551	O	THR	B	57	-10.010	2.634	59.846	1.00	0.00	O

ATOM	552	N	GLN	B	58	-9.979	0.403	60.128	1.00	0.00	N
ATOM	553	H	GLN	B	58	-10.442	-0.467	59.959	1.00	0.00	H
ATOM	554	CA	GLN	B	58	-8.541	0.526	60.346	1.00	0.00	C
ATOM	555	CB	GLN	B	58	-7.997	-0.553	61.288	1.00	0.00	C
ATOM	556	CG	GLN	B	58	-8.524	-0.444	62.724	1.00	0.00	C
ATOM	557	CD	GLN	B	58	-8.229	0.930	63.301	1.00	0.00	C
ATOM	558	OE1	GLN	B	58	-9.010	1.863	63.173	1.00	0.00	O
ATOM	559	NE2	GLN	B	58	-7.060	1.022	63.937	1.00	0.00	N
ATOM	560	HE21	GLN	B	58	-6.781	1.896	64.336	1.00	0.00	H
ATOM	561	HE22	GLN	B	58	-6.466	0.219	64.036	1.00	0.00	H
ATOM	562	C	GLN	B	58	-7.749	0.594	59.056	1.00	0.00	C
ATOM	563	O	GLN	B	58	-7.303	-0.403	58.486	1.00	0.00	O
ATOM	564	N	HIS	B	59	-7.566	1.851	58.620	1.00	0.00	N
ATOM	565	H	HIS	B	59	-7.991	2.595	59.145	1.00	0.00	H
ATOM	566	CA	HIS	B	59	-6.624	2.062	57.522	1.00	0.00	C
ATOM	567	CB	HIS	B	59	-6.716	3.487	56.956	1.00	0.00	C
ATOM	568	CG	HIS	B	59	-7.845	3.608	55.950	1.00	0.00	C
ATOM	569	ND1	HIS	B	59	-8.595	4.714	55.781	1.00	0.00	N
ATOM	570	HD1	HIS	B	59	-8.535	5.565	56.268	1.00	0.00	H
ATOM	571	CD2	HIS	B	59	-8.280	2.644	55.035	1.00	0.00	C
ATOM	572	NE2	HIS	B	59	-9.297	3.175	54.313	1.00	0.00	N
ATOM	573	CE1	HIS	B	59	-9.490	4.456	54.774	1.00	0.00	C
ATOM	574	C	HIS	B	59	-5.209	1.708	57.949	1.00	0.00	C
ATOM	575	O	HIS	B	59	-4.566	0.832	57.374	1.00	0.00	O
ATOM	576	N	GLY	B	60	-4.793	2.364	59.044	1.00	0.00	N
ATOM	577	H	GLY	B	60	-5.419	3.006	59.490	1.00	0.00	H
ATOM	578	CA	GLY	B	60	-3.550	1.955	59.701	1.00	0.00	C
ATOM	579	C	GLY	B	60	-3.661	0.567	60.302	1.00	0.00	C
ATOM	580	O	GLY	B	60	-4.679	-0.106	60.156	1.00	0.00	O
ATOM	581	N	LYS	B	61	-2.556	0.114	60.900	1.00	0.00	N
ATOM	582	H	LYS	B	61	-1.800	0.717	61.155	1.00	0.00	H
ATOM	583	CA	LYS	B	61	-2.526	-1.330	61.109	1.00	0.00	C
ATOM	584	CB	LYS	B	61	-1.552	-2.006	60.136	1.00	0.00	C
ATOM	585	CG	LYS	B	61	-2.226	-2.212	58.776	1.00	0.00	C
ATOM	586	CD	LYS	B	61	-3.411	-3.177	58.892	1.00	0.00	C
ATOM	587	CE	LYS	B	61	-4.530	-2.911	57.883	1.00	0.00	C
ATOM	588	NZ	LYS	B	61	-5.121	-1.599	58.167	1.00	0.00	N
ATOM	589	HZ1	LYS	B	61	-4.577	-0.825	57.733	1.00	0.00	H
ATOM	590	HZ2	LYS	B	61	-6.112	-1.504	57.864	1.00	0.00	H
ATOM	591	HZ3	LYS	B	61	-5.129	-1.408	59.191	1.00	0.00	H
ATOM	592	C	LYS	B	61	-2.372	-1.848	62.519	1.00	0.00	C
ATOM	593	O	LYS	B	61	-1.951	-2.976	62.731	1.00	0.00	O
ATOM	594	N	GLU	B	62	-2.778	-1.008	63.477	1.00	0.00	N
ATOM	595	H	GLU	B	62	-3.114	-0.083	63.289	1.00	0.00	H
ATOM	596	CA	GLU	B	62	-3.084	-1.683	64.732	1.00	0.00	C
ATOM	597	CB	GLU	B	62	-2.731	-0.787	65.917	1.00	0.00	C
ATOM	598	CG	GLU	B	62	-2.935	-1.409	67.301	1.00	0.00	C
ATOM	599	CD	GLU	B	62	-2.267	-0.533	68.342	1.00	0.00	C
ATOM	600	OE1	GLU	B	62	-1.299	-0.976	68.951	1.00	0.00	O
ATOM	601	OE2	GLU	B	62	-2.671	0.612	68.533	1.00	0.00	O
ATOM	602	C	GLU	B	62	-4.535	-2.117	64.708	1.00	0.00	C
ATOM	603	O	GLU	B	62	-5.411	-1.369	64.282	1.00	0.00	O
ATOM	604	N	ASP	B	63	-4.724	-3.381	65.108	1.00	0.00	N
ATOM	605	H	ASP	B	63	-3.967	-3.890	65.511	1.00	0.00	H
ATOM	606	CA	ASP	B	63	-6.072	-3.941	65.051	1.00	0.00	C
ATOM	607	CB	ASP	B	63	-5.992	-5.470	65.107	1.00	0.00	C
ATOM	608	CG	ASP	B	63	-7.244	-6.083	64.514	1.00	0.00	C
ATOM	609	OD1	ASP	B	63	-7.414	-6.052	63.301	1.00	0.00	O
ATOM	610	OD2	ASP	B	63	-8.059	-6.613	65.258	1.00	0.00	O

ATOM	611	C	ASP	B	63	-6.962	-3.351	66.131	1.00	0.00	C
ATOM	612	O	ASP	B	63	-6.492	-2.631	67.006	1.00	0.00	O
ATOM	613	N	LEU	B	64	-8.266	-3.627	66.024	1.00	0.00	N
ATOM	614	H	LEU	B	64	-8.547	-4.415	65.468	1.00	0.00	H
ATOM	615	CA	LEU	B	64	-9.202	-2.795	66.774	1.00	0.00	C
ATOM	616	CB	LEU	B	64	-10.500	-2.664	65.983	1.00	0.00	C
ATOM	617	CG	LEU	B	64	-11.307	-1.419	66.337	1.00	0.00	C
ATOM	618	CD1	LEU	B	64	-10.529	-0.136	66.052	1.00	0.00	C
ATOM	619	CD2	LEU	B	64	-12.662	-1.434	65.645	1.00	0.00	C
ATOM	620	C	LEU	B	64	-9.465	-3.202	68.217	1.00	0.00	C
ATOM	621	O	LEU	B	64	-10.591	-3.422	68.646	1.00	0.00	O
ATOM	622	N	LYS	B	65	-8.369	-3.296	68.973	1.00	0.00	N
ATOM	623	H	LYS	B	65	-7.488	-2.989	68.609	1.00	0.00	H
ATOM	624	CA	LYS	B	65	-8.536	-3.779	70.337	1.00	0.00	C
ATOM	625	CB	LYS	B	65	-7.297	-4.577	70.741	1.00	0.00	C
ATOM	626	CG	LYS	B	65	-7.560	-5.567	71.876	1.00	0.00	C
ATOM	627	CD	LYS	B	65	-6.324	-6.413	72.169	1.00	0.00	C
ATOM	628	CE	LYS	B	65	-6.569	-7.450	73.262	1.00	0.00	C
ATOM	629	NZ	LYS	B	65	-5.350	-8.251	73.434	1.00	0.00	N
ATOM	630	HZ1	LYS	B	65	-4.564	-7.627	73.705	1.00	0.00	H
ATOM	631	HZ2	LYS	B	65	-5.504	-8.966	74.173	1.00	0.00	H
ATOM	632	HZ3	LYS	B	65	-5.124	-8.724	72.535	1.00	0.00	H
ATOM	633	C	LYS	B	65	-8.901	-2.701	71.345	1.00	0.00	C
ATOM	634	O	LYS	B	65	-8.106	-2.285	72.179	1.00	0.00	O
ATOM	635	N	PHE	B	66	-10.162	-2.265	71.224	1.00	0.00	N
ATOM	636	H	PHE	B	66	-10.744	-2.676	70.520	1.00	0.00	H
ATOM	637	CA	PHE	B	66	-10.659	-1.254	72.158	1.00	0.00	C
ATOM	638	CB	PHE	B	66	-12.079	-0.825	71.786	1.00	0.00	C
ATOM	639	CG	PHE	B	66	-12.166	0.051	70.557	1.00	0.00	C
ATOM	640	CD1	PHE	B	66	-13.224	-0.178	69.651	1.00	0.00	C
ATOM	641	CD2	PHE	B	66	-11.248	1.106	70.353	1.00	0.00	C
ATOM	642	CE1	PHE	B	66	-13.405	0.693	68.560	1.00	0.00	C
ATOM	643	CE2	PHE	B	66	-11.425	1.974	69.258	1.00	0.00	C
ATOM	644	CZ	PHE	B	66	-12.516	1.774	68.388	1.00	0.00	C
ATOM	645	C	PHE	B	66	-10.666	-1.699	73.616	1.00	0.00	C
ATOM	646	O	PHE	B	66	-11.078	-2.805	73.951	1.00	0.00	O
ATOM	647	N	PRO	B	67	-10.188	-0.776	74.486	1.00	0.00	N
ATOM	648	CD	PRO	B	67	-9.495	0.464	74.157	1.00	0.00	C
ATOM	649	CA	PRO	B	67	-10.321	-0.983	75.933	1.00	0.00	C
ATOM	650	CB	PRO	B	67	-9.603	0.239	76.521	1.00	0.00	C
ATOM	651	CG	PRO	B	67	-8.684	0.748	75.413	1.00	0.00	C
ATOM	652	C	PRO	B	67	-11.770	-1.070	76.391	1.00	0.00	C
ATOM	653	O	PRO	B	67	-12.697	-0.696	75.686	1.00	0.00	O
ATOM	654	N	ARG	B	68	-11.919	-1.581	77.624	1.00	0.00	N
ATOM	655	H	ARG	B	68	-11.110	-1.757	78.180	1.00	0.00	H
ATOM	656	CA	ARG	B	68	-13.263	-1.675	78.194	1.00	0.00	C
ATOM	657	CB	ARG	B	68	-13.207	-2.564	79.445	1.00	0.00	C
ATOM	658	CG	ARG	B	68	-14.568	-2.789	80.108	1.00	0.00	C
ATOM	659	CD	ARG	B	68	-14.616	-3.879	81.181	1.00	0.00	C
ATOM	660	NE	ARG	B	68	-14.125	-5.168	80.694	1.00	0.00	N
ATOM	661	HE	ARG	B	68	-13.264	-5.453	81.117	1.00	0.00	H
ATOM	662	CZ	ARG	B	68	-14.767	-5.884	79.744	1.00	0.00	C
ATOM	663	NH1	ARG	B	68	-15.964	-5.535	79.279	1.00	0.00	N
ATOM	664	HH11	ARG	B	68	-16.484	-4.767	79.679	1.00	0.00	H
ATOM	665	HH12	ARG	B	68	-16.370	-6.036	78.513	1.00	0.00	H
ATOM	666	NH2	ARG	B	68	-14.180	-6.964	79.246	1.00	0.00	N
ATOM	667	HH21	ARG	B	68	-13.312	-7.304	79.604	1.00	0.00	H
ATOM	668	HH22	ARG	B	68	-14.622	-7.442	78.477	1.00	0.00	H
ATOM	669	C	ARG	B	68	-13.860	-0.307	78.495	1.00	0.00	C

ATOM	670	O	ARG	B	68	-13.150	0.622	78.863	1.00	0.00	O
ATOM	671	N	GLY	B	69	-15.190	-0.217	78.322	1.00	0.00	N
ATOM	672	H	GLY	B	69	-15.666	-0.978	77.878	1.00	0.00	H
ATOM	673	CA	GLY	B	69	-15.878	1.032	78.649	1.00	0.00	C
ATOM	674	C	GLY	B	69	-15.862	2.012	77.495	1.00	0.00	C
ATOM	675	O	GLY	B	69	-16.881	2.451	76.977	1.00	0.00	O
ATOM	676	N	GLN	B	70	-14.620	2.303	77.096	1.00	0.00	N
ATOM	677	H	GLN	B	70	-13.866	1.892	77.606	1.00	0.00	H
ATOM	678	CA	GLN	B	70	-14.389	2.922	75.799	1.00	0.00	C
ATOM	679	CB	GLN	B	70	-12.897	3.216	75.671	1.00	0.00	C
ATOM	680	CG	GLN	B	70	-12.331	4.040	76.832	1.00	0.00	C
ATOM	681	CD	GLN	B	70	-10.825	4.130	76.696	1.00	0.00	C
ATOM	682	OE1	GLN	B	70	-10.227	3.688	75.723	1.00	0.00	O
ATOM	683	NE2	GLN	B	70	-10.221	4.729	77.723	1.00	0.00	N
ATOM	684	HE21	GLN	B	70	-9.233	4.866	77.684	1.00	0.00	H
ATOM	685	HE22	GLN	B	70	-10.742	5.068	78.503	1.00	0.00	H
ATOM	686	C	GLN	B	70	-14.833	1.987	74.686	1.00	0.00	C
ATOM	687	O	GLN	B	70	-15.182	0.836	74.923	1.00	0.00	O
ATOM	688	N	GLY	B	71	-14.787	2.508	73.456	1.00	0.00	N
ATOM	689	H	GLY	B	71	-14.625	3.488	73.319	1.00	0.00	H
ATOM	690	CA	GLY	B	71	-14.915	1.565	72.348	1.00	0.00	C
ATOM	691	C	GLY	B	71	-16.319	1.196	71.918	1.00	0.00	C
ATOM	692	O	GLY	B	71	-16.686	1.341	70.760	1.00	0.00	O
ATOM	693	N	VAL	B	72	-17.103	0.707	72.883	1.00	0.00	N
ATOM	694	H	VAL	B	72	-16.731	0.632	73.809	1.00	0.00	H
ATOM	695	CA	VAL	B	72	-18.512	0.485	72.559	1.00	0.00	C
ATOM	696	CB	VAL	B	72	-19.201	-0.402	73.599	1.00	0.00	C
ATOM	697	CG1	VAL	B	72	-18.992	-1.864	73.226	1.00	0.00	C
ATOM	698	CG2	VAL	B	72	-18.786	-0.081	75.040	1.00	0.00	C
ATOM	699	C	VAL	B	72	-19.271	1.783	72.373	1.00	0.00	C
ATOM	700	O	VAL	B	72	-19.328	2.628	73.258	1.00	0.00	O
ATOM	701	N	PRO	B	73	-19.843	1.933	71.159	1.00	0.00	N
ATOM	702	CD	PRO	B	73	-19.905	0.961	70.073	1.00	0.00	C
ATOM	703	CA	PRO	B	73	-20.490	3.202	70.834	1.00	0.00	C
ATOM	704	CB	PRO	B	73	-20.896	3.006	69.369	1.00	0.00	C
ATOM	705	CG	PRO	B	73	-21.013	1.496	69.178	1.00	0.00	C
ATOM	706	C	PRO	B	73	-21.643	3.531	71.764	1.00	0.00	C
ATOM	707	O	PRO	B	73	-22.213	2.679	72.443	1.00	0.00	O
ATOM	708	N	ILE	B	74	-21.947	4.833	71.772	1.00	0.00	N
ATOM	709	H	ILE	B	74	-21.452	5.453	71.162	1.00	0.00	H
ATOM	710	CA	ILE	B	74	-23.053	5.306	72.598	1.00	0.00	C
ATOM	711	CB	ILE	B	74	-22.890	6.806	72.886	1.00	0.00	C
ATOM	712	CG2	ILE	B	74	-23.975	7.333	73.834	1.00	0.00	C
ATOM	713	CG1	ILE	B	74	-21.482	7.063	73.444	1.00	0.00	C
ATOM	714	CD1	ILE	B	74	-21.153	8.537	73.683	1.00	0.00	C
ATOM	715	C	ILE	B	74	-24.392	4.947	71.974	1.00	0.00	C
ATOM	716	O	ILE	B	74	-25.027	5.685	71.227	1.00	0.00	O
ATOM	717	N	ASN	B	75	-24.763	3.705	72.306	1.00	0.00	N
ATOM	718	H	ASN	B	75	-24.136	3.182	72.885	1.00	0.00	H
ATOM	719	CA	ASN	B	75	-25.924	3.095	71.664	1.00	0.00	C
ATOM	720	CB	ASN	B	75	-26.089	1.624	72.026	1.00	0.00	C
ATOM	721	CG	ASN	B	75	-24.857	0.828	71.678	1.00	0.00	C
ATOM	722	OD1	ASN	B	75	-24.298	0.885	70.590	1.00	0.00	O
ATOM	723	ND2	ASN	B	75	-24.434	0.081	72.692	1.00	0.00	N
ATOM	724	HD21	ASN	B	75	-23.565	-0.404	72.576	1.00	0.00	H
ATOM	725	HD22	ASN	B	75	-24.951	-0.003	73.541	1.00	0.00	H
ATOM	726	C	ASN	B	75	-27.209	3.806	71.985	1.00	0.00	C
ATOM	727	O	ASN	B	75	-27.776	3.694	73.063	1.00	0.00	O
ATOM	728	N	THR	B	76	-27.658	4.538	70.968	1.00	0.00	N

ATOM	729	H	THR	B	76	-27.119	4.601	70.127	1.00	0.00	H
ATOM	730	CA	THR	B	76	-28.744	5.493	71.172	1.00	0.00	C
ATOM	731	CB	THR	B	76	-28.791	6.392	69.933	1.00	0.00	C
ATOM	732	OG1	THR	B	76	-27.512	6.368	69.272	1.00	0.00	O
ATOM	733	HG1	THR	B	76	-26.862	6.685	69.899	1.00	0.00	H
ATOM	734	CG2	THR	B	76	-29.201	7.829	70.266	1.00	0.00	C
ATOM	735	C	THR	B	76	-30.118	4.926	71.546	1.00	0.00	C
ATOM	736	O	THR	B	76	-31.090	5.642	71.760	1.00	0.00	O
ATOM	737	N	ASN	B	77	-30.165	3.588	71.626	1.00	0.00	N
ATOM	738	H	ASN	B	77	-29.361	3.026	71.447	1.00	0.00	H
ATOM	739	CA	ASN	B	77	-31.374	2.976	72.163	1.00	0.00	C
ATOM	740	CB	ASN	B	77	-32.408	2.742	71.060	1.00	0.00	C
ATOM	741	CG	ASN	B	77	-33.767	3.159	71.581	1.00	0.00	C
ATOM	742	OD1	ASN	B	77	-34.713	2.390	71.648	1.00	0.00	O
ATOM	743	ND2	ASN	B	77	-33.834	4.447	71.937	1.00	0.00	N
ATOM	744	HD21	ASN	B	77	-34.708	4.792	72.273	1.00	0.00	H
ATOM	745	HD22	ASN	B	77	-33.040	5.058	71.874	1.00	0.00	H
ATOM	746	C	ASN	B	77	-31.082	1.706	72.930	1.00	0.00	C
ATOM	747	O	ASN	B	77	-31.624	0.640	72.666	1.00	0.00	O
ATOM	748	N	SER	B	78	-30.143	1.855	73.873	1.00	0.00	N
ATOM	749	H	SER	B	78	-29.656	2.717	74.030	1.00	0.00	H
ATOM	750	CA	SER	B	78	-29.749	0.683	74.652	1.00	0.00	C
ATOM	751	CB	SER	B	78	-28.676	-0.103	73.893	1.00	0.00	C
ATOM	752	OG	SER	B	78	-29.046	-0.231	72.511	1.00	0.00	O
ATOM	753	HG	SER	B	78	-29.983	-0.430	72.520	1.00	0.00	H
ATOM	754	C	SER	B	78	-29.256	1.093	76.026	1.00	0.00	C
ATOM	755	O	SER	B	78	-28.985	2.264	76.267	1.00	0.00	O
ATOM	756	N	SER	B	79	-29.161	0.106	76.923	1.00	0.00	N
ATOM	757	H	SER	B	79	-29.351	-0.851	76.689	1.00	0.00	H
ATOM	758	CA	SER	B	79	-28.599	0.440	78.228	1.00	0.00	C
ATOM	759	CB	SER	B	79	-29.163	-0.549	79.264	1.00	0.00	C
ATOM	760	OG	SER	B	79	-28.571	-1.841	79.098	1.00	0.00	O
ATOM	761	HG	SER	B	79	-29.122	-2.337	78.486	1.00	0.00	H
ATOM	762	C	SER	B	79	-27.072	0.424	78.177	1.00	0.00	C
ATOM	763	O	SER	B	79	-26.494	-0.116	77.237	1.00	0.00	O
ATOM	764	N	PRO	B	80	-26.410	0.990	79.223	1.00	0.00	N
ATOM	765	CD	PRO	B	80	-26.928	1.898	80.244	1.00	0.00	C
ATOM	766	CA	PRO	B	80	-24.981	0.692	79.411	1.00	0.00	C
ATOM	767	CB	PRO	B	80	-24.674	1.331	80.769	1.00	0.00	C
ATOM	768	CG	PRO	B	80	-25.677	2.475	80.895	1.00	0.00	C
ATOM	769	C	PRO	B	80	-24.648	-0.797	79.360	1.00	0.00	C
ATOM	770	O	PRO	B	80	-23.718	-1.237	78.698	1.00	0.00	O
ATOM	771	N	ASP	B	81	-25.494	-1.570	80.048	1.00	0.00	N
ATOM	772	H	ASP	B	81	-26.143	-1.192	80.711	1.00	0.00	H
ATOM	773	CA	ASP	B	81	-25.353	-3.028	80.030	1.00	0.00	C
ATOM	774	CB	ASP	B	81	-26.179	-3.618	81.167	1.00	0.00	C
ATOM	775	CG	ASP	B	81	-25.542	-3.199	82.475	1.00	0.00	C
ATOM	776	OD1	ASP	B	81	-25.767	-2.068	82.901	1.00	0.00	O
ATOM	777	OD2	ASP	B	81	-24.799	-3.994	83.044	1.00	0.00	O
ATOM	778	C	ASP	B	81	-25.663	-3.717	78.711	1.00	0.00	C
ATOM	779	O	ASP	B	81	-25.391	-4.897	78.484	1.00	0.00	O
ATOM	780	N	ASP	B	82	-26.219	-2.911	77.806	1.00	0.00	N
ATOM	781	H	ASP	B	82	-26.574	-2.012	78.061	1.00	0.00	H
ATOM	782	CA	ASP	B	82	-26.346	-3.417	76.450	1.00	0.00	C
ATOM	783	CB	ASP	B	82	-27.677	-3.029	75.810	1.00	0.00	C
ATOM	784	CG	ASP	B	82	-28.852	-3.530	76.627	1.00	0.00	C
ATOM	785	OD1	ASP	B	82	-28.790	-4.630	77.174	1.00	0.00	O
ATOM	786	OD2	ASP	B	82	-29.824	-2.792	76.744	1.00	0.00	O
ATOM	787	C	ASP	B	82	-25.203	-3.076	75.528	1.00	0.00	C

ATOM	788	O	ASP	B	82	-25.179	-3.487	74.375	1.00	0.00	O
ATOM	789	N	GLN	B	83	-24.228	-2.332	76.069	1.00	0.00	N
ATOM	790	H	GLN	B	83	-24.281	-2.038	77.024	1.00	0.00	H
ATOM	791	CA	GLN	B	83	-23.039	-2.042	75.271	1.00	0.00	C
ATOM	792	CB	GLN	B	83	-22.371	-0.752	75.754	1.00	0.00	C
ATOM	793	CG	GLN	B	83	-23.268	0.488	75.715	1.00	0.00	C
ATOM	794	CD	GLN	B	83	-22.488	1.694	76.194	1.00	0.00	C
ATOM	795	OE1	GLN	B	83	-22.564	2.099	77.345	1.00	0.00	O
ATOM	796	NE2	GLN	B	83	-21.712	2.253	75.260	1.00	0.00	N
ATOM	797	HE21	GLN	B	83	-21.077	2.976	75.526	1.00	0.00	H
ATOM	798	HE22	GLN	B	83	-21.718	1.987	74.294	1.00	0.00	H
ATOM	799	C	GLN	B	83	-22.034	-3.187	75.210	1.00	0.00	C
ATOM	800	O	GLN	B	83	-20.903	-3.091	75.666	1.00	0.00	O
ATOM	801	N	ILE	B	84	-22.508	-4.288	74.608	1.00	0.00	N
ATOM	802	H	ILE	B	84	-23.440	-4.279	74.246	1.00	0.00	H
ATOM	803	CA	ILE	B	84	-21.626	-5.419	74.337	1.00	0.00	C
ATOM	804	CB	ILE	B	84	-22.154	-6.700	74.994	1.00	0.00	C
ATOM	805	CG2	ILE	B	84	-21.214	-7.881	74.736	1.00	0.00	C
ATOM	806	CG1	ILE	B	84	-22.403	-6.501	76.490	1.00	0.00	C
ATOM	807	CD1	ILE	B	84	-22.929	-7.771	77.153	1.00	0.00	C
ATOM	808	C	ILE	B	84	-21.503	-5.599	72.838	1.00	0.00	C
ATOM	809	O	ILE	B	84	-22.297	-6.279	72.193	1.00	0.00	O
ATOM	810	N	GLY	B	85	-20.496	-4.907	72.309	1.00	0.00	N
ATOM	811	H	GLY	B	85	-19.771	-4.536	72.893	1.00	0.00	H
ATOM	812	CA	GLY	B	85	-20.385	-4.866	70.861	1.00	0.00	C
ATOM	813	C	GLY	B	85	-19.142	-5.562	70.374	1.00	0.00	C
ATOM	814	O	GLY	B	85	-18.019	-5.164	70.661	1.00	0.00	O
ATOM	815	N	TYR	B	86	-19.390	-6.631	69.612	1.00	0.00	N
ATOM	816	H	TYR	B	86	-20.324	-6.840	69.321	1.00	0.00	H
ATOM	817	CA	TYR	B	86	-18.223	-7.149	68.924	1.00	0.00	C
ATOM	818	CB	TYR	B	86	-18.215	-8.684	68.839	1.00	0.00	C
ATOM	819	CG	TYR	B	86	-19.259	-9.269	67.911	1.00	0.00	C
ATOM	820	CD1	TYR	B	86	-18.911	-9.504	66.565	1.00	0.00	C
ATOM	821	CE1	TYR	B	86	-19.844	-10.116	65.714	1.00	0.00	C
ATOM	822	CD2	TYR	B	86	-20.532	-9.596	68.419	1.00	0.00	C
ATOM	823	CE2	TYR	B	86	-21.465	-10.215	67.570	1.00	0.00	C
ATOM	824	CZ	TYR	B	86	-21.104	-10.475	66.232	1.00	0.00	C
ATOM	825	OH	TYR	B	86	-22.010	-11.098	65.396	1.00	0.00	O
ATOM	826	HH	TYR	B	86	-22.827	-11.192	65.887	1.00	0.00	H
ATOM	827	C	TYR	B	86	-17.991	-6.461	67.597	1.00	0.00	C
ATOM	828	O	TYR	B	86	-18.847	-6.374	66.721	1.00	0.00	O
ATOM	829	N	TYR	B	87	-16.766	-5.949	67.504	1.00	0.00	N
ATOM	830	H	TYR	B	87	-16.140	-6.055	68.279	1.00	0.00	H
ATOM	831	CA	TYR	B	87	-16.340	-5.417	66.220	1.00	0.00	C
ATOM	832	CB	TYR	B	87	-15.206	-4.420	66.441	1.00	0.00	C
ATOM	833	CG	TYR	B	87	-15.749	-3.095	66.918	1.00	0.00	C
ATOM	834	CD1	TYR	B	87	-15.881	-2.839	68.300	1.00	0.00	C
ATOM	835	CE1	TYR	B	87	-16.348	-1.581	68.720	1.00	0.00	C
ATOM	836	CD2	TYR	B	87	-16.095	-2.138	65.947	1.00	0.00	C
ATOM	837	CE2	TYR	B	87	-16.546	-0.881	66.368	1.00	0.00	C
ATOM	838	CZ	TYR	B	87	-16.666	-0.613	67.744	1.00	0.00	C
ATOM	839	OH	TYR	B	87	-17.110	0.637	68.122	1.00	0.00	O
ATOM	840	HH	TYR	B	87	-16.937	0.785	69.049	1.00	0.00	H
ATOM	841	C	TYR	B	87	-15.893	-6.544	65.316	1.00	0.00	C
ATOM	842	O	TYR	B	87	-14.987	-7.293	65.655	1.00	0.00	O
ATOM	843	N	ARG	B	88	-16.574	-6.636	64.168	1.00	0.00	N
ATOM	844	H	ARG	B	88	-17.331	-6.011	63.974	1.00	0.00	H
ATOM	845	CA	ARG	B	88	-16.230	-7.721	63.258	1.00	0.00	C
ATOM	846	CB	ARG	B	88	-17.467	-8.575	62.975	1.00	0.00	C

```
ATOM    847  CG  ARG B  88     -17.112  -9.994  62.527  1.00  0.00           C
ATOM    848  CD  ARG B  88     -17.762 -10.433  61.216  1.00  0.00           C
ATOM    849  NE  ARG B  88     -17.339  -9.544  60.140  1.00  0.00           N
ATOM    850  HE  ARG B  88     -16.354  -9.447  59.968  1.00  0.00           H
ATOM    851  CZ  ARG B  88     -18.214  -8.744  59.507  1.00  0.00           C
ATOM    852  NH1 ARG B  88     -19.526  -8.931  59.644  1.00  0.00           N
ATOM    853 HH11 ARG B  88     -19.851  -9.727  60.153  1.00  0.00           H
ATOM    854 HH12 ARG B  88     -20.228  -8.305  59.284  1.00  0.00           H
ATOM    855  NH2 ARG B  88     -17.727  -7.765  58.761  1.00  0.00           N
ATOM    856 HH21 ARG B  88     -16.725  -7.664  58.732  1.00  0.00           H
ATOM    857 HH22 ARG B  88     -18.305  -7.133  58.250  1.00  0.00           H
ATOM    858  C   ARG B  88     -15.574  -7.245  61.972  1.00  0.00           C
ATOM    859  O   ARG B  88     -16.161  -6.515  61.178  1.00  0.00           O
ATOM    860  N   ARG B  89     -14.323  -7.696  61.800  1.00  0.00           N
ATOM    861  H   ARG B  89     -13.970  -8.301  62.519  1.00  0.00           H
ATOM    862  CA  ARG B  89     -13.495  -7.249  60.670  1.00  0.00           C
ATOM    863  CB  ARG B  89     -12.101  -7.888  60.810  1.00  0.00           C
ATOM    864  CG  ARG B  89     -10.934  -7.121  60.169  1.00  0.00           C
ATOM    865  CD  ARG B  89      -9.569  -7.813  60.303  1.00  0.00           C
ATOM    866  NE  ARG B  89      -9.039  -7.793  61.670  1.00  0.00           N
ATOM    867  HE  ARG B  89      -8.882  -6.930  62.168  1.00  0.00           H
ATOM    868  CZ  ARG B  89      -8.583  -8.911  62.275  1.00  0.00           C
ATOM    869  NH1 ARG B  89      -8.655 -10.090  61.674  1.00  0.00           N
ATOM    870 HH11 ARG B  89      -9.025 -10.213  60.759  1.00  0.00           H
ATOM    871 HH12 ARG B  89      -8.319 -10.918  62.157  1.00  0.00           H
ATOM    872  NH2 ARG B  89      -8.056  -8.840  63.487  1.00  0.00           N
ATOM    873 HH21 ARG B  89      -7.958  -7.946  63.955  1.00  0.00           H
ATOM    874 HH22 ARG B  89      -7.740  -9.677  63.948  1.00  0.00           H
ATOM    875  C   ARG B  89     -14.119  -7.497  59.291  1.00  0.00           C
ATOM    876  O   ARG B  89     -15.040  -8.299  59.143  1.00  0.00           O
ATOM    877  N   ALA B  90     -13.593  -6.779  58.286  1.00  0.00           N
ATOM    878  H   ALA B  90     -12.978  -6.012  58.491  1.00  0.00           H
ATOM    879  CA  ALA B  90     -13.984  -7.004  56.895  1.00  0.00           C
ATOM    880  CB  ALA B  90     -15.373  -6.426  56.603  1.00  0.00           C
ATOM    881  C   ALA B  90     -13.019  -6.328  55.942  1.00  0.00           C
ATOM    882  O   ALA B  90     -12.277  -5.432  56.322  1.00  0.00           O
ATOM    883  N   THR B  91     -13.100  -6.772  54.686  1.00  0.00           N
ATOM    884  H   THR B  91     -13.668  -7.558  54.447  1.00  0.00           H
ATOM    885  CA  THR B  91     -12.378  -6.119  53.595  1.00  0.00           C
ATOM    886  CB  THR B  91     -11.103  -6.906  53.272  1.00  0.00           C
ATOM    887  OG1 THR B  91     -11.321  -8.308  53.502  1.00  0.00           O
ATOM    888  HG1 THR B  91     -11.992  -8.581  52.888  1.00  0.00           H
ATOM    889  CG2 THR B  91      -9.902  -6.415  54.079  1.00  0.00           C
ATOM    890  C   THR B  91     -13.306  -6.125  52.401  1.00  0.00           C
ATOM    891  O   THR B  91     -14.169  -6.992  52.311  1.00  0.00           O
ATOM    892  N   ARG B  92     -13.123  -5.152  51.505  1.00  0.00           N
ATOM    893  H   ARG B  92     -12.394  -4.470  51.596  1.00  0.00           H
ATOM    894  CA  ARG B  92     -13.966  -5.193  50.313  1.00  0.00           C
ATOM    895  CB  ARG B  92     -15.140  -4.222  50.431  1.00  0.00           C
ATOM    896  CG  ARG B  92     -16.474  -4.837  50.004  1.00  0.00           C
ATOM    897  CD  ARG B  92     -17.254  -3.964  49.018  1.00  0.00           C
ATOM    898  NE  ARG B  92     -16.766  -4.134  47.648  1.00  0.00           N
ATOM    899  HE  ARG B  92     -15.794  -4.303  47.467  1.00  0.00           H
ATOM    900  CZ  ARG B  92     -17.632  -4.085  46.615  1.00  0.00           C
ATOM    901  NH1 ARG B  92     -18.912  -3.785  46.800  1.00  0.00           N
ATOM    902 HH11 ARG B  92     -19.301  -3.539  47.687  1.00  0.00           H
ATOM    903 HH12 ARG B  92     -19.543  -3.781  46.017  1.00  0.00           H
ATOM    904  NH2 ARG B  92     -17.208  -4.340  45.386  1.00  0.00           N
ATOM    905 HH21 ARG B  92     -16.246  -4.579  45.213  1.00  0.00           H
```

```
ATOM    906 HH22 ARG B  92     -17.861  -4.299  44.623  1.00  0.00           H
ATOM    907  C   ARG B  92     -13.143  -4.822  49.109  1.00  0.00           C
ATOM    908  O   ARG B  92     -12.297  -3.952  49.196  1.00  0.00           O
ATOM    909  N   ARG B  93     -13.399  -5.494  47.982  1.00  0.00           N
ATOM    910  H   ARG B  93     -14.076  -6.226  47.933  1.00  0.00           H
ATOM    911  CA  ARG B  93     -12.577  -5.100  46.838  1.00  0.00           C
ATOM    912  CB  ARG B  93     -11.697  -6.278  46.397  1.00  0.00           C
ATOM    913  CG  ARG B  93     -10.751  -6.561  47.562  1.00  0.00           C
ATOM    914  CD  ARG B  93      -9.757  -7.716  47.516  1.00  0.00           C
ATOM    915  NE  ARG B  93      -9.004  -7.627  48.764  1.00  0.00           N
ATOM    916  HE  ARG B  93      -9.215  -6.842  49.359  1.00  0.00           H
ATOM    917  CZ  ARG B  93      -8.031  -8.457  49.169  1.00  0.00           C
ATOM    918  NH1 ARG B  93      -7.717  -9.531  48.448  1.00  0.00           N
ATOM    919 HH11 ARG B  93      -8.216  -9.713  47.600  1.00  0.00           H
ATOM    920 HH12 ARG B  93      -6.994 -10.161  48.728  1.00  0.00           H
ATOM    921  NH2 ARG B  93      -7.393  -8.174  50.301  1.00  0.00           N
ATOM    922 HH21 ARG B  93      -7.616  -7.309  50.782  1.00  0.00           H
ATOM    923 HH22 ARG B  93      -6.690  -8.751  50.709  1.00  0.00           H
ATOM    924  C   ARG B  93     -13.401  -4.504  45.722  1.00  0.00           C
ATOM    925  O   ARG B  93     -14.576  -4.832  45.561  1.00  0.00           O
ATOM    926  N   ILE B  94     -12.764  -3.587  44.983  1.00  0.00           N
ATOM    927  H   ILE B  94     -11.830  -3.307  45.229  1.00  0.00           H
ATOM    928  CA  ILE B  94     -13.416  -3.038  43.792  1.00  0.00           C
ATOM    929  CB  ILE B  94     -13.926  -1.595  44.007  1.00  0.00           C
ATOM    930  CG2 ILE B  94     -14.943  -1.215  42.924  1.00  0.00           C
ATOM    931  CG1 ILE B  94     -14.511  -1.342  45.401  1.00  0.00           C
ATOM    932  CD1 ILE B  94     -14.906   0.122  45.617  1.00  0.00           C
ATOM    933  C   ILE B  94     -12.424  -3.065  42.642  1.00  0.00           C
ATOM    934  O   ILE B  94     -11.232  -2.876  42.847  1.00  0.00           O
ATOM    935  N   ARG B  95     -12.943  -3.303  41.425  1.00  0.00           N
ATOM    936  H   ARG B  95     -13.922  -3.453  41.306  1.00  0.00           H
ATOM    937  CA  ARG B  95     -12.034  -3.281  40.277  1.00  0.00           C
ATOM    938  CB  ARG B  95     -12.770  -3.710  39.004  1.00  0.00           C
ATOM    939  CG  ARG B  95     -11.844  -4.401  38.003  1.00  0.00           C
ATOM    940  CD  ARG B  95     -12.479  -4.686  36.641  1.00  0.00           C
ATOM    941  NE  ARG B  95     -12.523  -3.491  35.797  1.00  0.00           N
ATOM    942  HE  ARG B  95     -11.845  -2.775  35.981  1.00  0.00           H
ATOM    943  CZ  ARG B  95     -13.336  -3.445  34.715  1.00  0.00           C
ATOM    944  NH1 ARG B  95     -14.177  -4.441  34.442  1.00  0.00           N
ATOM    945 HH11 ARG B  95     -14.175  -5.296  34.976  1.00  0.00           H
ATOM    946 HH12 ARG B  95     -14.838  -4.371  33.686  1.00  0.00           H
ATOM    947  NH2 ARG B  95     -13.285  -2.390  33.909  1.00  0.00           N
ATOM    948 HH21 ARG B  95     -12.696  -1.604  34.091  1.00  0.00           H
ATOM    949 HH22 ARG B  95     -13.834  -2.363  33.064  1.00  0.00           H
ATOM    950  C   ARG B  95     -11.400  -1.917  40.070  1.00  0.00           C
ATOM    951  O   ARG B  95     -12.099  -0.913  39.937  1.00  0.00           O
ATOM    952  N   GLY B  96     -10.065  -1.914  40.078  1.00  0.00           N
ATOM    953  H   GLY B  96      -9.550  -2.767  40.181  1.00  0.00           H
ATOM    954  CA  GLY B  96      -9.373  -0.690  39.694  1.00  0.00           C
ATOM    955  C   GLY B  96      -9.259  -0.618  38.187  1.00  0.00           C
ATOM    956  O   GLY B  96      -9.763  -1.483  37.476  1.00  0.00           O
ATOM    957  N   GLY B  97      -8.574   0.445  37.735  1.00  0.00           N
ATOM    958  H   GLY B  97      -8.220   1.101  38.404  1.00  0.00           H
ATOM    959  CA  GLY B  97      -8.465   0.693  36.293  1.00  0.00           C
ATOM    960  C   GLY B  97      -8.044  -0.498  35.452  1.00  0.00           C
ATOM    961  O   GLY B  97      -8.744  -0.959  34.559  1.00  0.00           O
ATOM    962  N   ASP B  98      -6.867  -1.000  35.845  1.00  0.00           N
ATOM    963  H   ASP B  98      -6.343  -0.474  36.517  1.00  0.00           H
ATOM    964  CA  ASP B  98      -6.287  -2.196  35.229  1.00  0.00           C
```

ATOM	965	CB	ASP	B	98	-5.121	-2.708	36.058	1.00	0.00	C
ATOM	966	CG	ASP	B	98	-3.831	-1.945	35.912	1.00	0.00	C
ATOM	967	OD1	ASP	B	98	-3.241	-1.952	34.847	1.00	0.00	O
ATOM	968	OD2	ASP	B	98	-3.291	-1.503	36.916	1.00	0.00	O
ATOM	969	C	ASP	B	98	-7.197	-3.405	35.101	1.00	0.00	C
ATOM	970	O	ASP	B	98	-7.079	-4.211	34.188	1.00	0.00	O
ATOM	971	N	GLY	B	99	-8.032	-3.558	36.137	1.00	0.00	N
ATOM	972	H	GLY	B	99	-8.265	-2.785	36.728	1.00	0.00	H
ATOM	973	CA	GLY	B	99	-8.514	-4.906	36.415	1.00	0.00	C
ATOM	974	C	GLY	B	99	-8.096	-5.355	37.803	1.00	0.00	C
ATOM	975	O	GLY	B	99	-8.849	-5.970	38.545	1.00	0.00	O
ATOM	976	N	LYS	B	100	-6.850	-4.970	38.141	1.00	0.00	N
ATOM	977	H	LYS	B	100	-6.293	-4.528	37.444	1.00	0.00	H
ATOM	978	CA	LYS	B	100	-6.349	-5.185	39.503	1.00	0.00	C
ATOM	979	CB	LYS	B	100	-4.974	-4.525	39.685	1.00	0.00	C
ATOM	980	CG	LYS	B	100	-3.890	-4.963	38.689	1.00	0.00	C
ATOM	981	CD	LYS	B	100	-2.585	-4.172	38.866	1.00	0.00	C
ATOM	982	CE	LYS	B	100	-1.514	-4.412	37.787	1.00	0.00	C
ATOM	983	NZ	LYS	B	100	-1.978	-3.946	36.474	1.00	0.00	N
ATOM	984	HZ1	LYS	B	100	-1.357	-4.219	35.684	1.00	0.00	H
ATOM	985	HZ2	LYS	B	100	-2.865	-4.432	36.226	1.00	0.00	H
ATOM	986	HZ3	LYS	B	100	-2.173	-2.918	36.438	1.00	0.00	H
ATOM	987	C	LYS	B	100	-7.313	-4.665	40.562	1.00	0.00	C
ATOM	988	O	LYS	B	100	-7.788	-3.536	40.500	1.00	0.00	O
ATOM	989	N	MET	B	101	-7.613	-5.553	41.514	1.00	0.00	N
ATOM	990	H	MET	B	101	-7.132	-6.425	41.558	1.00	0.00	H
ATOM	991	CA	MET	B	101	-8.632	-5.180	42.492	1.00	0.00	C
ATOM	992	CB	MET	B	101	-9.323	-6.431	43.037	1.00	0.00	C
ATOM	993	CG	MET	B	101	-9.973	-7.302	41.957	1.00	0.00	C
ATOM	994	SD	MET	B	101	-11.437	-6.563	41.217	1.00	0.00	S
ATOM	995	CE	MET	B	101	-12.576	-6.842	42.581	1.00	0.00	C
ATOM	996	C	MET	B	101	-8.069	-4.365	43.637	1.00	0.00	C
ATOM	997	O	MET	B	101	-7.224	-4.822	44.398	1.00	0.00	O
ATOM	998	N	LYS	B	102	-8.578	-3.134	43.750	1.00	0.00	N
ATOM	999	H	LYS	B	102	-9.322	-2.848	43.146	1.00	0.00	H
ATOM	1000	CA	LYS	B	102	-8.196	-2.388	44.943	1.00	0.00	C
ATOM	1001	CB	LYS	B	102	-8.390	-0.878	44.750	1.00	0.00	C
ATOM	1002	CG	LYS	B	102	-7.890	-0.019	45.926	1.00	0.00	C
ATOM	1003	CD	LYS	B	102	-6.384	-0.155	46.191	1.00	0.00	C
ATOM	1004	CE	LYS	B	102	-5.873	0.652	47.394	1.00	0.00	C
ATOM	1005	NZ	LYS	B	102	-6.360	0.097	48.663	1.00	0.00	N
ATOM	1006	HZ1	LYS	B	102	-6.211	0.781	49.437	1.00	0.00	H
ATOM	1007	HZ2	LYS	B	102	-7.382	-0.095	48.621	1.00	0.00	H
ATOM	1008	HZ3	LYS	B	102	-5.896	-0.796	48.911	1.00	0.00	H
ATOM	1009	C	LYS	B	102	-8.934	-2.882	46.171	1.00	0.00	C
ATOM	1010	O	LYS	B	102	-10.155	-2.818	46.269	1.00	0.00	O
ATOM	1011	N	ASP	B	103	-8.105	-3.357	47.108	1.00	0.00	N
ATOM	1012	H	ASP	B	103	-7.149	-3.522	46.869	1.00	0.00	H
ATOM	1013	CA	ASP	B	103	-8.631	-3.654	48.437	1.00	0.00	C
ATOM	1014	CB	ASP	B	103	-7.595	-4.442	49.245	1.00	0.00	C
ATOM	1015	CG	ASP	B	103	-8.209	-5.136	50.455	1.00	0.00	C
ATOM	1016	OD1	ASP	B	103	-7.457	-5.605	51.298	1.00	0.00	O
ATOM	1017	OD2	ASP	B	103	-9.427	-5.285	50.528	1.00	0.00	O
ATOM	1018	C	ASP	B	103	-9.025	-2.400	49.190	1.00	0.00	C
ATOM	1019	O	ASP	B	103	-8.360	-1.366	49.106	1.00	0.00	O
ATOM	1020	N	LEU	B	104	-10.126	-2.530	49.922	1.00	0.00	N
ATOM	1021	H	LEU	B	104	-10.567	-3.432	49.947	1.00	0.00	H
ATOM	1022	CA	LEU	B	104	-10.516	-1.462	50.824	1.00	0.00	C
ATOM	1023	CB	LEU	B	104	-11.995	-1.078	50.696	1.00	0.00	C

```
ATOM   1024  CG  LEU B 104     -12.553  -0.866  49.282  1.00  0.00           C
ATOM   1025  CD1 LEU B 104     -14.022  -0.450  49.356  1.00  0.00           C
ATOM   1026  CD2 LEU B 104     -11.731   0.085  48.411  1.00  0.00           C
ATOM   1027  C   LEU B 104     -10.217  -1.876  52.246  1.00  0.00           C
ATOM   1028  O   LEU B 104     -11.035  -2.474  52.935  1.00  0.00           O
ATOM   1029  N   SER B 105      -8.993  -1.494  52.638  1.00  0.00           N
ATOM   1030  H   SER B 105      -8.375  -1.078  51.972  1.00  0.00           H
ATOM   1031  CA  SER B 105      -8.414  -1.762  53.955  1.00  0.00           C
ATOM   1032  CB  SER B 105      -7.354  -0.685  54.195  1.00  0.00           C
ATOM   1033  OG  SER B 105      -6.712  -0.370  52.949  1.00  0.00           O
ATOM   1034  HG  SER B 105      -6.015   0.241  53.153  1.00  0.00           H
ATOM   1035  C   SER B 105      -9.382  -1.883  55.132  1.00  0.00           C
ATOM   1036  O   SER B 105     -10.316  -1.093  55.251  1.00  0.00           O
ATOM   1037  N   PRO B 106      -9.110  -2.912  55.984  1.00  0.00           N
ATOM   1038  CD  PRO B 106      -7.893  -3.722  55.987  1.00  0.00           C
ATOM   1039  CA  PRO B 106     -10.031  -3.374  57.029  1.00  0.00           C
ATOM   1040  CB  PRO B 106      -9.074  -3.964  58.063  1.00  0.00           C
ATOM   1041  CG  PRO B 106      -8.039  -4.662  57.184  1.00  0.00           C
ATOM   1042  C   PRO B 106     -11.087  -2.441  57.583  1.00  0.00           C
ATOM   1043  O   PRO B 106     -10.848  -1.554  58.397  1.00  0.00           O
ATOM   1044  N   ARG B 107     -12.298  -2.737  57.107  1.00  0.00           N
ATOM   1045  H   ARG B 107     -12.361  -3.525  56.495  1.00  0.00           H
ATOM   1046  CA  ARG B 107     -13.478  -2.166  57.737  1.00  0.00           C
ATOM   1047  CB  ARG B 107     -14.643  -2.096  56.749  1.00  0.00           C
ATOM   1048  CG  ARG B 107     -14.700  -0.833  55.889  1.00  0.00           C
ATOM   1049  CD  ARG B 107     -13.569  -0.673  54.874  1.00  0.00           C
ATOM   1050  NE  ARG B 107     -13.751   0.574  54.139  1.00  0.00           N
ATOM   1051  HE  ARG B 107     -14.677   0.947  54.025  1.00  0.00           H
ATOM   1052  CZ  ARG B 107     -12.730   1.251  53.585  1.00  0.00           C
ATOM   1053  NH1 ARG B 107     -11.469   0.857  53.732  1.00  0.00           N
ATOM   1054 HH11 ARG B 107     -11.250   0.019  54.250  1.00  0.00           H
ATOM   1055 HH12 ARG B 107     -10.717   1.401  53.357  1.00  0.00           H
ATOM   1056  NH2 ARG B 107     -13.012   2.338  52.883  1.00  0.00           N
ATOM   1057 HH21 ARG B 107     -13.964   2.668  52.878  1.00  0.00           H
ATOM   1058 HH22 ARG B 107     -12.299   2.818  52.362  1.00  0.00           H
ATOM   1059  C   ARG B 107     -13.893  -3.007  58.926  1.00  0.00           C
ATOM   1060  O   ARG B 107     -13.504  -4.160  59.070  1.00  0.00           O
ATOM   1061  N   TRP B 108     -14.720  -2.378  59.760  1.00  0.00           N
ATOM   1062  H   TRP B 108     -14.978  -1.426  59.592  1.00  0.00           H
ATOM   1063  CA  TRP B 108     -15.275  -3.089  60.900  1.00  0.00           C
ATOM   1064  CB  TRP B 108     -14.658  -2.596  62.210  1.00  0.00           C
ATOM   1065  CG  TRP B 108     -13.195  -2.962  62.279  1.00  0.00           C
ATOM   1066  CD2 TRP B 108     -12.613  -4.110  62.926  1.00  0.00           C
ATOM   1067  CE2 TRP B 108     -11.197  -4.042  62.719  1.00  0.00           C
ATOM   1068  CE3 TRP B 108     -13.167  -5.178  63.661  1.00  0.00           C
ATOM   1069  CD1 TRP B 108     -12.110  -2.262  61.724  1.00  0.00           C
ATOM   1070  NE1 TRP B 108     -10.934  -2.896  61.979  1.00  0.00           N
ATOM   1071  HE1 TRP B 108     -10.043  -2.603  61.697  1.00  0.00           H
ATOM   1072  CZ2 TRP B 108     -10.364  -5.046  63.253  1.00  0.00           C
ATOM   1073  CZ3 TRP B 108     -12.323  -6.176  64.192  1.00  0.00           C
ATOM   1074  CH2 TRP B 108     -10.929  -6.110  63.988  1.00  0.00           C
ATOM   1075  C   TRP B 108     -16.763  -2.856  60.917  1.00  0.00           C
ATOM   1076  O   TRP B 108     -17.234  -1.737  60.752  1.00  0.00           O
ATOM   1077  N   TYR B 109     -17.481  -3.964  61.087  1.00  0.00           N
ATOM   1078  H   TYR B 109     -17.033  -4.841  61.262  1.00  0.00           H
ATOM   1079  CA  TYR B 109     -18.926  -3.835  61.188  1.00  0.00           C
ATOM   1080  CB  TYR B 109     -19.621  -4.638  60.081  1.00  0.00           C
ATOM   1081  CG  TYR B 109     -19.345  -3.983  58.744  1.00  0.00           C
ATOM   1082  CD1 TYR B 109     -18.228  -4.395  57.990  1.00  0.00           C
```

ATOM	1083	CE1	TYR	B	109	-17.928	-3.725	56.793	1.00	0.00	C
ATOM	1084	CD2	TYR	B	109	-20.202	-2.956	58.298	1.00	0.00	C
ATOM	1085	CE2	TYR	B	109	-19.903	-2.288	57.099	1.00	0.00	C
ATOM	1086	CZ	TYR	B	109	-18.753	-2.661	56.375	1.00	0.00	C
ATOM	1087	OH	TYR	B	109	-18.415	-1.958	55.228	1.00	0.00	O
ATOM	1088	HH	TYR	B	109	-17.797	-2.499	54.741	1.00	0.00	H
ATOM	1089	C	TYR	B	109	-19.356	-4.252	62.570	1.00	0.00	C
ATOM	1090	O	TYR	B	109	-18.850	-5.211	63.138	1.00	0.00	O
ATOM	1091	N	PHE	B	110	-20.258	-3.437	63.104	1.00	0.00	N
ATOM	1092	H	PHE	B	110	-20.712	-2.749	62.538	1.00	0.00	H
ATOM	1093	CA	PHE	B	110	-20.572	-3.529	64.523	1.00	0.00	C
ATOM	1094	CB	PHE	B	110	-21.038	-2.172	65.073	1.00	0.00	C
ATOM	1095	CG	PHE	B	110	-20.071	-1.010	64.929	1.00	0.00	C
ATOM	1096	CD1	PHE	B	110	-19.236	-0.850	63.798	1.00	0.00	C
ATOM	1097	CD2	PHE	B	110	-20.065	-0.053	65.963	1.00	0.00	C
ATOM	1098	CE1	PHE	B	110	-18.405	0.278	63.690	1.00	0.00	C
ATOM	1099	CE2	PHE	B	110	-19.241	1.082	65.857	1.00	0.00	C
ATOM	1100	CZ	PHE	B	110	-18.426	1.241	64.718	1.00	0.00	C
ATOM	1101	C	PHE	B	110	-21.720	-4.486	64.709	1.00	0.00	C
ATOM	1102	O	PHE	B	110	-22.775	-4.299	64.114	1.00	0.00	O
ATOM	1103	N	TYR	B	111	-21.503	-5.490	65.555	1.00	0.00	N
ATOM	1104	H	TYR	B	111	-20.617	-5.671	65.993	1.00	0.00	H
ATOM	1105	CA	TYR	B	111	-22.687	-6.251	65.933	1.00	0.00	C
ATOM	1106	CB	TYR	B	111	-22.753	-7.622	65.260	1.00	0.00	C
ATOM	1107	CG	TYR	B	111	-22.551	-7.528	63.767	1.00	0.00	C
ATOM	1108	CD1	TYR	B	111	-21.247	-7.697	63.265	1.00	0.00	C
ATOM	1109	CE1	TYR	B	111	-21.048	-7.668	61.880	1.00	0.00	C
ATOM	1110	CD2	TYR	B	111	-23.654	-7.290	62.921	1.00	0.00	C
ATOM	1111	CE2	TYR	B	111	-23.453	-7.277	61.530	1.00	0.00	C
ATOM	1112	CZ	TYR	B	111	-22.153	-7.495	61.025	1.00	0.00	C
ATOM	1113	OH	TYR	B	111	-21.946	-7.564	59.656	1.00	0.00	O
ATOM	1114	HH	TYR	B	111	-22.808	-7.534	59.237	1.00	0.00	H
ATOM	1115	C	TYR	B	111	-22.699	-6.410	67.427	1.00	0.00	C
ATOM	1116	O	TYR	B	111	-21.685	-6.230	68.091	1.00	0.00	O
ATOM	1117	N	TYR	B	112	-23.884	-6.716	67.944	1.00	0.00	N
ATOM	1118	H	TYR	B	112	-24.680	-6.958	67.385	1.00	0.00	H
ATOM	1119	CA	TYR	B	112	-23.923	-6.812	69.395	1.00	0.00	C
ATOM	1120	CB	TYR	B	112	-24.972	-5.840	69.935	1.00	0.00	C
ATOM	1121	CG	TYR	B	112	-24.314	-4.505	70.202	1.00	0.00	C
ATOM	1122	CD1	TYR	B	112	-23.719	-3.768	69.153	1.00	0.00	C
ATOM	1123	CE1	TYR	B	112	-23.058	-2.564	69.449	1.00	0.00	C
ATOM	1124	CD2	TYR	B	112	-24.299	-4.045	71.529	1.00	0.00	C
ATOM	1125	CE2	TYR	B	112	-23.633	-2.849	71.826	1.00	0.00	C
ATOM	1126	CZ	TYR	B	112	-23.017	-2.128	70.788	1.00	0.00	C
ATOM	1127	OH	TYR	B	112	-22.363	-0.958	71.110	1.00	0.00	O
ATOM	1128	HH	TYR	B	112	-22.753	-0.244	70.604	1.00	0.00	H
ATOM	1129	C	TYR	B	112	-24.164	-8.235	69.820	1.00	0.00	C
ATOM	1130	O	TYR	B	112	-24.506	-9.077	68.999	1.00	0.00	O
ATOM	1131	N	LEU	B	113	-23.989	-8.481	71.124	1.00	0.00	N
ATOM	1132	H	LEU	B	113	-23.618	-7.748	71.697	1.00	0.00	H
ATOM	1133	CA	LEU	B	113	-24.271	-9.822	71.647	1.00	0.00	C
ATOM	1134	CB	LEU	B	113	-24.209	-9.769	73.173	1.00	0.00	C
ATOM	1135	CG	LEU	B	113	-24.237	-11.128	73.872	1.00	0.00	C
ATOM	1136	CD1	LEU	B	113	-23.056	-12.005	73.472	1.00	0.00	C
ATOM	1137	CD2	LEU	B	113	-24.304	-10.968	75.384	1.00	0.00	C
ATOM	1138	C	LEU	B	113	-25.609	-10.390	71.178	1.00	0.00	C
ATOM	1139	O	LEU	B	113	-26.637	-9.731	71.256	1.00	0.00	O
ATOM	1140	N	GLY	B	114	-25.561	-11.624	70.659	1.00	0.00	N
ATOM	1141	H	GLY	B	114	-24.705	-12.131	70.556	1.00	0.00	H

ATOM	1142	CA	GLY	B	114	-26.829	-12.189	70.196	1.00	0.00	C
ATOM	1143	C	GLY	B	114	-27.259	-11.801	68.786	1.00	0.00	C
ATOM	1144	O	GLY	B	114	-28.242	-12.301	68.258	1.00	0.00	O
ATOM	1145	N	THR	B	115	-26.492	-10.884	68.184	1.00	0.00	N
ATOM	1146	H	THR	B	115	-25.634	-10.568	68.585	1.00	0.00	H
ATOM	1147	CA	THR	B	115	-26.839	-10.493	66.821	1.00	0.00	C
ATOM	1148	CB	THR	B	115	-27.343	-9.039	66.779	1.00	0.00	C
ATOM	1149	OG1	THR	B	115	-26.363	-8.137	67.307	1.00	0.00	O
ATOM	1150	HG1	THR	B	115	-26.056	-8.550	68.112	1.00	0.00	H
ATOM	1151	CG2	THR	B	115	-28.668	-8.867	67.527	1.00	0.00	C
ATOM	1152	C	THR	B	115	-25.641	-10.695	65.915	1.00	0.00	C
ATOM	1153	O	THR	B	115	-24.547	-11.004	66.377	1.00	0.00	O
ATOM	1154	N	GLY	B	116	-25.873	-10.491	64.613	1.00	0.00	N
ATOM	1155	H	GLY	B	116	-26.806	-10.420	64.257	1.00	0.00	H
ATOM	1156	CA	GLY	B	116	-24.751	-10.625	63.691	1.00	0.00	C
ATOM	1157	C	GLY	B	116	-24.370	-12.070	63.437	1.00	0.00	C
ATOM	1158	O	GLY	B	116	-24.994	-13.001	63.935	1.00	0.00	O
ATOM	1159	N	PRO	B	117	-23.300	-12.233	62.628	1.00	0.00	N
ATOM	1160	CD	PRO	B	117	-22.510	-11.179	62.006	1.00	0.00	C
ATOM	1161	CA	PRO	B	117	-22.825	-13.584	62.305	1.00	0.00	C
ATOM	1162	CB	PRO	B	117	-21.645	-13.309	61.361	1.00	0.00	C
ATOM	1163	CG	PRO	B	117	-21.207	-11.871	61.638	1.00	0.00	C
ATOM	1164	C	PRO	B	117	-22.478	-14.417	63.531	1.00	0.00	C
ATOM	1165	O	PRO	B	117	-22.869	-15.570	63.662	1.00	0.00	O
ATOM	1166	N	GLU	B	118	-21.765	-13.765	64.457	1.00	0.00	N
ATOM	1167	H	GLU	B	118	-21.577	-12.785	64.388	1.00	0.00	H
ATOM	1168	CA	GLU	B	118	-21.342	-14.499	65.646	1.00	0.00	C
ATOM	1169	CB	GLU	B	118	-20.007	-13.952	66.151	1.00	0.00	C
ATOM	1170	CG	GLU	B	118	-18.977	-13.653	65.056	1.00	0.00	C
ATOM	1171	CD	GLU	B	118	-18.463	-14.901	64.357	1.00	0.00	C
ATOM	1172	OE1	GLU	B	118	-18.337	-15.953	64.979	1.00	0.00	O
ATOM	1173	OE2	GLU	B	118	-18.101	-14.816	63.185	1.00	0.00	O
ATOM	1174	C	GLU	B	118	-22.369	-14.466	66.765	1.00	0.00	C
ATOM	1175	O	GLU	B	118	-22.047	-14.364	67.942	1.00	0.00	O
ATOM	1176	N	ALA	B	119	-23.646	-14.545	66.354	1.00	0.00	N
ATOM	1177	H	ALA	B	119	-23.837	-14.669	65.380	1.00	0.00	H
ATOM	1178	CA	ALA	B	119	-24.739	-14.336	67.305	1.00	0.00	C
ATOM	1179	CB	ALA	B	119	-26.086	-14.564	66.619	1.00	0.00	C
ATOM	1180	C	ALA	B	119	-24.689	-15.196	68.558	1.00	0.00	C
ATOM	1181	O	ALA	B	119	-25.006	-14.765	69.658	1.00	0.00	O
ATOM	1182	N	GLY	B	120	-24.273	-16.450	68.329	1.00	0.00	N
ATOM	1183	H	GLY	B	120	-23.935	-16.699	67.424	1.00	0.00	H
ATOM	1184	CA	GLY	B	120	-24.267	-17.381	69.454	1.00	0.00	C
ATOM	1185	C	GLY	B	120	-23.046	-17.331	70.358	1.00	0.00	C
ATOM	1186	O	GLY	B	120	-22.952	-18.069	71.330	1.00	0.00	O
ATOM	1187	N	LEU	B	121	-22.094	-16.458	69.999	1.00	0.00	N
ATOM	1188	H	LEU	B	121	-22.243	-15.752	69.306	1.00	0.00	H
ATOM	1189	CA	LEU	B	121	-20.928	-16.390	70.874	1.00	0.00	C
ATOM	1190	CB	LEU	B	121	-19.695	-15.888	70.117	1.00	0.00	C
ATOM	1191	CG	LEU	B	121	-19.186	-16.809	69.006	1.00	0.00	C
ATOM	1192	CD1	LEU	B	121	-17.892	-16.262	68.400	1.00	0.00	C
ATOM	1193	CD2	LEU	B	121	-19.011	-18.257	69.470	1.00	0.00	C
ATOM	1194	C	LEU	B	121	-21.190	-15.519	72.087	1.00	0.00	C
ATOM	1195	O	LEU	B	121	-21.611	-14.376	71.972	1.00	0.00	O
ATOM	1196	N	PRO	B	122	-20.938	-16.103	73.280	1.00	0.00	N
ATOM	1197	CD	PRO	B	122	-20.501	-17.473	73.531	1.00	0.00	C
ATOM	1198	CA	PRO	B	122	-21.099	-15.312	74.502	1.00	0.00	C
ATOM	1199	CB	PRO	B	122	-21.031	-16.390	75.588	1.00	0.00	C
ATOM	1200	CG	PRO	B	122	-20.125	-17.477	75.008	1.00	0.00	C

```
ATOM   1201  C   PRO B 122     -20.004 -14.265  74.621  1.00  0.00           C
ATOM   1202  O   PRO B 122     -18.976 -14.318  73.947  1.00  0.00           O
ATOM   1203  N   TYR B 123     -20.253 -13.306  75.527  1.00  0.00           N
ATOM   1204  H   TYR B 123     -21.086 -13.297  76.087  1.00  0.00           H
ATOM   1205  CA  TYR B 123     -19.220 -12.292  75.718  1.00  0.00           C
ATOM   1206  CB  TYR B 123     -19.691 -11.207  76.699  1.00  0.00           C
ATOM   1207  CG  TYR B 123     -18.650 -10.114  76.811  1.00  0.00           C
ATOM   1208  CD1 TYR B 123     -18.297  -9.391  75.656  1.00  0.00           C
ATOM   1209  CE1 TYR B 123     -17.292  -8.421  75.740  1.00  0.00           C
ATOM   1210  CD2 TYR B 123     -18.046  -9.865  78.060  1.00  0.00           C
ATOM   1211  CE2 TYR B 123     -17.034  -8.894  78.144  1.00  0.00           C
ATOM   1212  CZ  TYR B 123     -16.661  -8.196  76.977  1.00  0.00           C
ATOM   1213  OH  TYR B 123     -15.644  -7.261  77.045  1.00  0.00           O
ATOM   1214  HH  TYR B 123     -15.356  -7.093  76.139  1.00  0.00           H
ATOM   1215  C   TYR B 123     -17.875 -12.866  76.134  1.00  0.00           C
ATOM   1216  O   TYR B 123     -17.702 -13.431  77.204  1.00  0.00           O
ATOM   1217  N   GLY B 124     -16.920 -12.670  75.219  1.00  0.00           N
ATOM   1218  H   GLY B 124     -17.165 -12.321  74.315  1.00  0.00           H
ATOM   1219  CA  GLY B 124     -15.581 -13.127  75.566  1.00  0.00           C
ATOM   1220  C   GLY B 124     -15.097 -14.331  74.783  1.00  0.00           C
ATOM   1221  O   GLY B 124     -13.955 -14.754  74.924  1.00  0.00           O
ATOM   1222  N   ALA B 125     -15.986 -14.854  73.918  1.00  0.00           N
ATOM   1223  H   ALA B 125     -16.922 -14.504  73.843  1.00  0.00           H
ATOM   1224  CA  ALA B 125     -15.518 -15.914  73.024  1.00  0.00           C
ATOM   1225  CB  ALA B 125     -16.700 -16.630  72.375  1.00  0.00           C
ATOM   1226  C   ALA B 125     -14.574 -15.398  71.948  1.00  0.00           C
ATOM   1227  O   ALA B 125     -14.961 -15.029  70.846  1.00  0.00           O
ATOM   1228  N   ASN B 126     -13.301 -15.350  72.358  1.00  0.00           N
ATOM   1229  H   ASN B 126     -13.101 -15.719  73.266  1.00  0.00           H
ATOM   1230  CA  ASN B 126     -12.283 -14.716  71.522  1.00  0.00           C
ATOM   1231  CB  ASN B 126     -10.971 -14.548  72.291  1.00  0.00           C
ATOM   1232  CG  ASN B 126     -10.947 -13.224  73.029  1.00  0.00           C
ATOM   1233  OD1 ASN B 126     -10.295 -12.268  72.632  1.00  0.00           O
ATOM   1234  ND2 ASN B 126     -11.664 -13.202  74.156  1.00  0.00           N
ATOM   1235 HD21 ASN B 126     -11.644 -12.371  74.705  1.00  0.00           H
ATOM   1236 HD22 ASN B 126     -12.216 -13.979  74.466  1.00  0.00           H
ATOM   1237  C   ASN B 126     -12.020 -15.456  70.232  1.00  0.00           C
ATOM   1238  O   ASN B 126     -11.815 -16.663  70.207  1.00  0.00           O
ATOM   1239  N   LYS B 127     -12.036 -14.658  69.163  1.00  0.00           N
ATOM   1240  H   LYS B 127     -12.236 -13.680  69.250  1.00  0.00           H
ATOM   1241  CA  LYS B 127     -11.826 -15.188  67.822  1.00  0.00           C
ATOM   1242  CB  LYS B 127     -13.177 -15.700  67.299  1.00  0.00           C
ATOM   1243  CG  LYS B 127     -13.240 -16.278  65.882  1.00  0.00           C
ATOM   1244  CD  LYS B 127     -14.703 -16.444  65.459  1.00  0.00           C
ATOM   1245  CE  LYS B 127     -14.900 -16.797  63.984  1.00  0.00           C
ATOM   1246  NZ  LYS B 127     -16.311 -16.602  63.636  1.00  0.00           N
ATOM   1247  HZ1 LYS B 127     -16.601 -16.980  62.719  1.00  0.00           H
ATOM   1248  HZ2 LYS B 127     -16.975 -16.945  64.365  1.00  0.00           H
ATOM   1249  HZ3 LYS B 127     -16.573 -15.590  63.620  1.00  0.00           H
ATOM   1250  C   LYS B 127     -11.270 -14.034  67.010  1.00  0.00           C
ATOM   1251  O   LYS B 127     -11.550 -12.877  67.312  1.00  0.00           O
ATOM   1252  N   ASP B 128     -10.448 -14.368  66.001  1.00  0.00           N
ATOM   1253  H   ASP B 128     -10.283 -15.317  65.748  1.00  0.00           H
ATOM   1254  CA  ASP B 128      -9.881 -13.259  65.232  1.00  0.00           C
ATOM   1255  CB  ASP B 128      -8.781 -13.744  64.271  1.00  0.00           C
ATOM   1256  CG  ASP B 128      -8.182 -12.582  63.485  1.00  0.00           C
ATOM   1257  OD1 ASP B 128      -7.883 -11.537  64.064  1.00  0.00           O
ATOM   1258  OD2 ASP B 128      -8.083 -12.685  62.270  1.00  0.00           O
ATOM   1259  C   ASP B 128     -10.942 -12.434  64.522  1.00  0.00           C
```

```
ATOM   1260  O   ASP B 128     -11.953 -12.933  64.039  1.00  0.00           O
ATOM   1261  N   GLY B 129     -10.672 -11.124  64.558  1.00  0.00           N
ATOM   1262  H   GLY B 129      -9.753 -10.884  64.875  1.00  0.00           H
ATOM   1263  CA  GLY B 129     -11.600 -10.156  63.990  1.00  0.00           C
ATOM   1264  C   GLY B 129     -12.941 -10.044  64.687  1.00  0.00           C
ATOM   1265  O   GLY B 129     -13.879  -9.490  64.127  1.00  0.00           O
ATOM   1266  N   ILE B 130     -12.986 -10.586  65.918  1.00  0.00           N
ATOM   1267  H   ILE B 130     -12.205 -11.086  66.295  1.00  0.00           H
ATOM   1268  CA  ILE B 130     -14.202 -10.481  66.723  1.00  0.00           C
ATOM   1269  CB  ILE B 130     -14.818 -11.864  67.006  1.00  0.00           C
ATOM   1270  CG2 ILE B 130     -16.237 -11.751  67.569  1.00  0.00           C
ATOM   1271  CG1 ILE B 130     -14.759 -12.802  65.802  1.00  0.00           C
ATOM   1272  CD1 ILE B 130     -15.601 -12.411  64.596  1.00  0.00           C
ATOM   1273  C   ILE B 130     -13.860  -9.794  68.030  1.00  0.00           C
ATOM   1274  O   ILE B 130     -13.735 -10.398  69.091  1.00  0.00           O
ATOM   1275  N   ILE B 131     -13.661  -8.479  67.914  1.00  0.00           N
ATOM   1276  H   ILE B 131     -13.883  -8.005  67.059  1.00  0.00           H
ATOM   1277  CA  ILE B 131     -13.237  -7.808  69.139  1.00  0.00           C
ATOM   1278  CB  ILE B 131     -12.477  -6.505  68.854  1.00  0.00           C
ATOM   1279  CG2 ILE B 131     -11.827  -5.981  70.140  1.00  0.00           C
ATOM   1280  CG1 ILE B 131     -11.447  -6.661  67.727  1.00  0.00           C
ATOM   1281  CD1 ILE B 131     -10.282  -7.600  68.058  1.00  0.00           C
ATOM   1282  C   ILE B 131     -14.397  -7.576  70.090  1.00  0.00           C
ATOM   1283  O   ILE B 131     -15.250  -6.724  69.876  1.00  0.00           O
ATOM   1284  N   TRP B 132     -14.385  -8.396  71.148  1.00  0.00           N
ATOM   1285  H   TRP B 132     -13.698  -9.124  71.146  1.00  0.00           H
ATOM   1286  CA  TRP B 132     -15.416  -8.308  72.182  1.00  0.00           C
ATOM   1287  CB  TRP B 132     -15.419  -9.587  73.020  1.00  0.00           C
ATOM   1288  CG  TRP B 132     -16.077 -10.710  72.262  1.00  0.00           C
ATOM   1289  CD2 TRP B 132     -17.484 -10.890  71.995  1.00  0.00           C
ATOM   1290  CE2 TRP B 132     -17.619 -12.109  71.252  1.00  0.00           C
ATOM   1291  CE3 TRP B 132     -18.629 -10.130  72.319  1.00  0.00           C
ATOM   1292  CD1 TRP B 132     -15.442 -11.813  71.675  1.00  0.00           C
ATOM   1293  NE1 TRP B 132     -16.344 -12.639  71.081  1.00  0.00           N
ATOM   1294  HE1 TRP B 132     -16.119 -13.471  70.603  1.00  0.00           H
ATOM   1295  CZ2 TRP B 132     -18.898 -12.541  70.846  1.00  0.00           C
ATOM   1296  CZ3 TRP B 132     -19.903 -10.575  71.908  1.00  0.00           C
ATOM   1297  CH2 TRP B 132     -20.036 -11.774  71.174  1.00  0.00           C
ATOM   1298  C   TRP B 132     -15.328  -7.095  73.097  1.00  0.00           C
ATOM   1299  O   TRP B 132     -14.954  -7.182  74.267  1.00  0.00           O
ATOM   1300  N   VAL B 133     -15.705  -5.949  72.526  1.00  0.00           N
ATOM   1301  H   VAL B 133     -16.107  -5.929  71.605  1.00  0.00           H
ATOM   1302  CA  VAL B 133     -15.716  -4.773  73.386  1.00  0.00           C
ATOM   1303  CB  VAL B 133     -15.522  -3.489  72.564  1.00  0.00           C
ATOM   1304  CG1 VAL B 133     -15.171  -2.292  73.452  1.00  0.00           C
ATOM   1305  CG2 VAL B 133     -14.458  -3.681  71.485  1.00  0.00           C
ATOM   1306  C   VAL B 133     -17.000  -4.733  74.195  1.00  0.00           C
ATOM   1307  O   VAL B 133     -18.061  -5.149  73.742  1.00  0.00           O
ATOM   1308  N   ALA B 134     -16.853  -4.237  75.426  1.00  0.00           N
ATOM   1309  H   ALA B 134     -15.972  -3.900  75.760  1.00  0.00           H
ATOM   1310  CA  ALA B 134     -18.049  -3.997  76.217  1.00  0.00           C
ATOM   1311  CB  ALA B 134     -18.556  -5.268  76.902  1.00  0.00           C
ATOM   1312  C   ALA B 134     -17.745  -2.981  77.281  1.00  0.00           C
ATOM   1313  O   ALA B 134     -16.587  -2.696  77.573  1.00  0.00           O
ATOM   1314  N   THR B 135     -18.831  -2.483  77.863  1.00  0.00           N
ATOM   1315  H   THR B 135     -19.749  -2.715  77.524  1.00  0.00           H
ATOM   1316  CA  THR B 135     -18.732  -1.722  79.103  1.00  0.00           C
ATOM   1317  CB  THR B 135     -20.126  -1.169  79.399  1.00  0.00           C
ATOM   1318  OG1 THR B 135     -21.110  -2.000  78.766  1.00  0.00           O
```

ATOM	1319	HG1	THR	B	135	-21.958	-1.591	78.923	1.00	0.00	H
ATOM	1320	CG2	THR	B	135	-20.258	0.277	78.922	1.00	0.00	C
ATOM	1321	C	THR	B	135	-18.206	-2.564	80.260	1.00	0.00	C
ATOM	1322	O	THR	B	135	-17.858	-3.735	80.105	1.00	0.00	O
ATOM	1323	N	GLU	B	136	-18.160	-1.915	81.429	1.00	0.00	N
ATOM	1324	H	GLU	B	136	-18.448	-0.964	81.519	1.00	0.00	H
ATOM	1325	CA	GLU	B	136	-18.103	-2.728	82.637	1.00	0.00	C
ATOM	1326	CB	GLU	B	136	-17.070	-2.152	83.605	1.00	0.00	C
ATOM	1327	CG	GLU	B	136	-16.575	-3.180	84.626	1.00	0.00	C
ATOM	1328	CD	GLU	B	136	-15.081	-3.023	84.814	1.00	0.00	C
ATOM	1329	OE1	GLU	B	136	-14.658	-2.003	85.349	1.00	0.00	O
ATOM	1330	OE2	GLU	B	136	-14.347	-3.924	84.410	1.00	0.00	O
ATOM	1331	C	GLU	B	136	-19.513	-2.766	83.190	1.00	0.00	C
ATOM	1332	O	GLU	B	136	-20.338	-1.956	82.785	1.00	0.00	O
ATOM	1333	N	GLY	B	137	-19.784	-3.755	84.052	1.00	0.00	N
ATOM	1334	H	GLY	B	137	-19.061	-4.377	84.347	1.00	0.00	H
ATOM	1335	CA	GLY	B	137	-21.194	-4.103	84.210	1.00	0.00	C
ATOM	1336	C	GLY	B	137	-21.552	-5.035	83.073	1.00	0.00	C
ATOM	1337	O	GLY	B	137	-21.178	-6.201	83.075	1.00	0.00	O
ATOM	1338	N	ALA	B	138	-22.197	-4.431	82.064	1.00	0.00	N
ATOM	1339	H	ALA	B	138	-22.688	-3.583	82.284	1.00	0.00	H
ATOM	1340	CA	ALA	B	138	-22.341	-5.073	80.757	1.00	0.00	C
ATOM	1341	CB	ALA	B	138	-21.000	-5.144	80.017	1.00	0.00	C
ATOM	1342	C	ALA	B	138	-22.978	-6.449	80.799	1.00	0.00	C
ATOM	1343	O	ALA	B	138	-22.439	-7.446	80.331	1.00	0.00	O
ATOM	1344	N	LEU	B	139	-24.186	-6.452	81.382	1.00	0.00	N
ATOM	1345	H	LEU	B	139	-24.494	-5.610	81.834	1.00	0.00	H
ATOM	1346	CA	LEU	B	139	-24.948	-7.698	81.451	1.00	0.00	C
ATOM	1347	CB	LEU	B	139	-26.358	-7.454	81.992	1.00	0.00	C
ATOM	1348	CG	LEU	B	139	-26.382	-6.943	83.435	1.00	0.00	C
ATOM	1349	CD1	LEU	B	139	-27.795	-6.551	83.868	1.00	0.00	C
ATOM	1350	CD2	LEU	B	139	-25.731	-7.925	84.411	1.00	0.00	C
ATOM	1351	C	LEU	B	139	-25.033	-8.477	80.150	1.00	0.00	C
ATOM	1352	O	LEU	B	139	-25.551	-8.016	79.132	1.00	0.00	O
ATOM	1353	N	ASN	B	140	-24.503	-9.709	80.272	1.00	0.00	N
ATOM	1354	H	ASN	B	140	-24.043	-9.902	81.137	1.00	0.00	H
ATOM	1355	CA	ASN	B	140	-24.547	-10.711	79.203	1.00	0.00	C
ATOM	1356	CB	ASN	B	140	-23.537	-11.839	79.497	1.00	0.00	C
ATOM	1357	CG	ASN	B	140	-23.357	-12.813	78.332	1.00	0.00	C
ATOM	1358	OD1	ASN	B	140	-22.476	-12.685	77.492	1.00	0.00	O
ATOM	1359	ND2	ASN	B	140	-24.195	-13.851	78.342	1.00	0.00	N
ATOM	1360	HD21	ASN	B	140	-24.073	-14.595	77.690	1.00	0.00	H
ATOM	1361	HD22	ASN	B	140	-24.959	-13.868	78.992	1.00	0.00	H
ATOM	1362	C	ASN	B	140	-25.950	-11.254	78.977	1.00	0.00	C
ATOM	1363	O	ASN	B	140	-26.280	-12.397	79.267	1.00	0.00	O
ATOM	1364	N	THR	B	141	-26.767	-10.358	78.438	1.00	0.00	N
ATOM	1365	H	THR	B	141	-26.448	-9.433	78.243	1.00	0.00	H
ATOM	1366	CA	THR	B	141	-28.062	-10.749	77.913	1.00	0.00	C
ATOM	1367	CB	THR	B	141	-29.113	-9.754	78.419	1.00	0.00	C
ATOM	1368	OG1	THR	B	141	-28.634	-8.397	78.329	1.00	0.00	O
ATOM	1369	HG1	THR	B	141	-29.390	-7.857	78.555	1.00	0.00	H
ATOM	1370	CG2	THR	B	141	-29.505	-10.067	79.864	1.00	0.00	C
ATOM	1371	C	THR	B	141	-27.926	-10.674	76.412	1.00	0.00	C
ATOM	1372	O	THR	B	141	-27.201	-9.820	75.915	1.00	0.00	O
ATOM	1373	N	PRO	B	142	-28.611	-11.579	75.685	1.00	0.00	N
ATOM	1374	CD	PRO	B	142	-29.315	-12.769	76.142	1.00	0.00	C
ATOM	1375	CA	PRO	B	142	-28.711	-11.358	74.240	1.00	0.00	C
ATOM	1376	CB	PRO	B	142	-29.551	-12.552	73.776	1.00	0.00	C
ATOM	1377	CG	PRO	B	142	-29.428	-13.608	74.876	1.00	0.00	C

```
ATOM   1378  C    PRO B 142     -29.353 -10.005  73.966  1.00  0.00           C
ATOM   1379  O    PRO B 142     -30.147  -9.507  74.754  1.00  0.00           O
ATOM   1380  N    LYS B 143     -28.923  -9.395  72.859  1.00  0.00           N
ATOM   1381  H    LYS B 143     -28.278  -9.827  72.230  1.00  0.00           H
ATOM   1382  CA   LYS B 143     -29.482  -8.075  72.581  1.00  0.00           C
ATOM   1383  CB   LYS B 143     -28.366  -7.038  72.367  1.00  0.00           C
ATOM   1384  CG   LYS B 143     -27.233  -7.051  73.411  1.00  0.00           C
ATOM   1385  CD   LYS B 143     -27.661  -6.803  74.864  1.00  0.00           C
ATOM   1386  CE   LYS B 143     -26.479  -6.858  75.845  1.00  0.00           C
ATOM   1387  NZ   LYS B 143     -26.930  -6.644  77.229  1.00  0.00           N
ATOM   1388  HZ1  LYS B 143     -27.570  -5.823  77.272  1.00  0.00           H
ATOM   1389  HZ2  LYS B 143     -27.445  -7.477  77.578  1.00  0.00           H
ATOM   1390  HZ3  LYS B 143     -26.132  -6.458  77.878  1.00  0.00           H
ATOM   1391  C    LYS B 143     -30.453  -8.122  71.412  1.00  0.00           C
ATOM   1392  O    LYS B 143     -30.610  -7.189  70.630  1.00  0.00           O
ATOM   1393  N    ASP B 144     -31.095  -9.295  71.325  1.00  0.00           N
ATOM   1394  H    ASP B 144     -31.068  -9.946  72.087  1.00  0.00           H
ATOM   1395  CA   ASP B 144     -32.016  -9.627  70.236  1.00  0.00           C
ATOM   1396  CB   ASP B 144     -32.558 -11.050  70.443  1.00  0.00           C
ATOM   1397  CG   ASP B 144     -33.150 -11.178  71.836  1.00  0.00           C
ATOM   1398  OD1  ASP B 144     -32.394 -11.413  72.774  1.00  0.00           O
ATOM   1399  OD2  ASP B 144     -34.348 -10.964  71.988  1.00  0.00           O
ATOM   1400  C    ASP B 144     -33.138  -8.618  70.055  1.00  0.00           C
ATOM   1401  O    ASP B 144     -33.393  -8.129  68.962  1.00  0.00           O
ATOM   1402  N    HIS B 145     -33.731  -8.250  71.202  1.00  0.00           N
ATOM   1403  H    HIS B 145     -33.627  -8.903  71.958  1.00  0.00           H
ATOM   1404  CA   HIS B 145     -34.744  -7.193  71.220  1.00  0.00           C
ATOM   1405  CB   HIS B 145     -35.178  -6.943  72.669  1.00  0.00           C
ATOM   1406  CG   HIS B 145     -36.560  -6.327  72.764  1.00  0.00           C
ATOM   1407  ND1  HIS B 145     -37.036  -5.355  71.960  1.00  0.00           N
ATOM   1408  HD1  HIS B 145     -36.573  -4.936  71.201  1.00  0.00           H
ATOM   1409  CD2  HIS B 145     -37.549  -6.649  73.697  1.00  0.00           C
ATOM   1410  NE2  HIS B 145     -38.623  -5.860  73.444  1.00  0.00           N
ATOM   1411  CE1  HIS B 145     -38.307  -5.064  72.375  1.00  0.00           C
ATOM   1412  C    HIS B 145     -34.323  -5.887  70.550  1.00  0.00           C
ATOM   1413  O    HIS B 145     -35.123  -5.154  69.979  1.00  0.00           O
ATOM   1414  N    ILE B 146     -33.016  -5.610  70.661  1.00  0.00           N
ATOM   1415  H    ILE B 146     -32.389  -6.284  71.053  1.00  0.00           H
ATOM   1416  CA   ILE B 146     -32.535  -4.387  70.025  1.00  0.00           C
ATOM   1417  CB   ILE B 146     -31.413  -3.739  70.850  1.00  0.00           C
ATOM   1418  CG2  ILE B 146     -31.309  -2.242  70.535  1.00  0.00           C
ATOM   1419  CG1  ILE B 146     -31.604  -3.969  72.352  1.00  0.00           C
ATOM   1420  CD1  ILE B 146     -30.389  -3.545  73.174  1.00  0.00           C
ATOM   1421  C    ILE B 146     -32.121  -4.591  68.570  1.00  0.00           C
ATOM   1422  O    ILE B 146     -32.111  -3.667  67.757  1.00  0.00           O
ATOM   1423  N    GLY B 147     -31.812  -5.861  68.269  1.00  0.00           N
ATOM   1424  H    GLY B 147     -31.724  -6.533  69.007  1.00  0.00           H
ATOM   1425  CA   GLY B 147     -31.736  -6.309  66.880  1.00  0.00           C
ATOM   1426  C    GLY B 147     -30.540  -5.828  66.085  1.00  0.00           C
ATOM   1427  O    GLY B 147     -29.846  -4.883  66.439  1.00  0.00           O
ATOM   1428  N    THR B 148     -30.349  -6.523  64.961  1.00  0.00           N
ATOM   1429  H    THR B 148     -30.910  -7.319  64.731  1.00  0.00           H
ATOM   1430  CA   THR B 148     -29.380  -6.078  63.962  1.00  0.00           C
ATOM   1431  CB   THR B 148     -29.183  -7.202  62.942  1.00  0.00           C
ATOM   1432  OG1  THR B 148     -30.122  -8.263  63.183  1.00  0.00           O
ATOM   1433  HG1  THR B 148     -30.380  -8.611  62.331  1.00  0.00           H
ATOM   1434  CG2  THR B 148     -27.745  -7.720  62.948  1.00  0.00           C
ATOM   1435  C    THR B 148     -29.837  -4.814  63.253  1.00  0.00           C
ATOM   1436  O    THR B 148     -30.979  -4.377  63.385  1.00  0.00           O
```

ATOM	1437	N	ARG	B	149	-28.918	-4.228	62.477	1.00	0.00	N
ATOM	1438	H	ARG	B	149	-27.977	-4.560	62.377	1.00	0.00	H
ATOM	1439	CA	ARG	B	149	-29.470	-3.263	61.532	1.00	0.00	C
ATOM	1440	CB	ARG	B	149	-28.654	-1.973	61.522	1.00	0.00	C
ATOM	1441	CG	ARG	B	149	-28.507	-1.402	62.935	1.00	0.00	C
ATOM	1442	CD	ARG	B	149	-29.834	-1.105	63.628	1.00	0.00	C
ATOM	1443	NE	ARG	B	149	-29.648	-1.030	65.075	1.00	0.00	N
ATOM	1444	HE	ARG	B	149	-28.960	-0.390	65.419	1.00	0.00	H
ATOM	1445	CZ	ARG	B	149	-30.373	-1.829	65.890	1.00	0.00	C
ATOM	1446	NH1	ARG	B	149	-31.266	-2.684	65.410	1.00	0.00	N
ATOM	1447	HH11	ARG	B	149	-31.385	-2.868	64.430	1.00	0.00	H
ATOM	1448	HH12	ARG	B	149	-31.824	-3.214	66.058	1.00	0.00	H
ATOM	1449	NH2	ARG	B	149	-30.200	-1.780	67.204	1.00	0.00	N
ATOM	1450	HH21	ARG	B	149	-29.547	-1.167	67.644	1.00	0.00	H
ATOM	1451	HH22	ARG	B	149	-30.746	-2.406	67.772	1.00	0.00	H
ATOM	1452	C	ARG	B	149	-29.587	-3.926	60.180	1.00	0.00	C
ATOM	1453	O	ARG	B	149	-29.222	-5.089	60.044	1.00	0.00	O
ATOM	1454	N	ASN	B	150	-30.130	-3.192	59.203	1.00	0.00	N
ATOM	1455	H	ASN	B	150	-30.347	-2.217	59.312	1.00	0.00	H
ATOM	1456	CA	ASN	B	150	-30.429	-3.863	57.936	1.00	0.00	C
ATOM	1457	CB	ASN	B	150	-31.743	-4.630	58.010	1.00	0.00	C
ATOM	1458	CG	ASN	B	150	-31.463	-6.099	57.884	1.00	0.00	C
ATOM	1459	OD1	ASN	B	150	-31.300	-6.653	56.806	1.00	0.00	O
ATOM	1460	ND2	ASN	B	150	-31.351	-6.708	59.056	1.00	0.00	N
ATOM	1461	HD21	ASN	B	150	-31.011	-7.646	59.097	1.00	0.00	H
ATOM	1462	HD22	ASN	B	150	-31.571	-6.249	59.915	1.00	0.00	H
ATOM	1463	C	ASN	B	150	-30.576	-2.858	56.833	1.00	0.00	C
ATOM	1464	O	ASN	B	150	-31.136	-1.795	57.069	1.00	0.00	O
ATOM	1465	N	PRO	B	151	-30.063	-3.210	55.628	1.00	0.00	N
ATOM	1466	CD	PRO	B	151	-29.247	-4.386	55.340	1.00	0.00	C
ATOM	1467	CA	PRO	B	151	-30.252	-2.362	54.443	1.00	0.00	C
ATOM	1468	CB	PRO	B	151	-29.418	-3.078	53.372	1.00	0.00	C
ATOM	1469	CG	PRO	B	151	-28.433	-3.967	54.126	1.00	0.00	C
ATOM	1470	C	PRO	B	151	-31.703	-2.156	54.009	1.00	0.00	C
ATOM	1471	O	PRO	B	151	-32.164	-2.698	53.014	1.00	0.00	O
ATOM	1472	N	ALA	B	152	-32.401	-1.327	54.794	1.00	0.00	N
ATOM	1473	H	ALA	B	152	-31.979	-0.943	55.615	1.00	0.00	H
ATOM	1474	CA	ALA	B	152	-33.779	-1.021	54.455	1.00	0.00	C
ATOM	1475	CB	ALA	B	152	-34.736	-1.618	55.499	1.00	0.00	C
ATOM	1476	C	ALA	B	152	-33.967	0.476	54.281	1.00	0.00	C
ATOM	1477	O	ALA	B	152	-33.540	1.051	53.286	1.00	0.00	O
ATOM	1478	N	ASN	B	153	-34.626	1.114	55.261	1.00	0.00	N
ATOM	1479	H	ASN	B	153	-34.694	0.747	56.191	1.00	0.00	H
ATOM	1480	CA	ASN	B	153	-35.029	2.480	54.939	1.00	0.00	C
ATOM	1481	CB	ASN	B	153	-36.416	2.783	55.536	1.00	0.00	C
ATOM	1482	CG	ASN	B	153	-36.822	4.243	55.368	1.00	0.00	C
ATOM	1483	OD1	ASN	B	153	-36.551	5.094	56.209	1.00	0.00	O
ATOM	1484	ND2	ASN	B	153	-37.500	4.497	54.247	1.00	0.00	N
ATOM	1485	HD21	ASN	B	153	-37.808	5.432	54.073	1.00	0.00	H
ATOM	1486	HD22	ASN	B	153	-37.717	3.784	53.582	1.00	0.00	H
ATOM	1487	C	ASN	B	153	-33.982	3.520	55.284	1.00	0.00	C
ATOM	1488	O	ASN	B	153	-33.373	4.125	54.409	1.00	0.00	O
ATOM	1489	N	ASN	B	154	-33.841	3.733	56.594	1.00	0.00	N
ATOM	1490	H	ASN	B	154	-34.221	3.108	57.279	1.00	0.00	H
ATOM	1491	CA	ASN	B	154	-33.225	5.002	56.958	1.00	0.00	C
ATOM	1492	CB	ASN	B	154	-33.877	5.537	58.229	1.00	0.00	C
ATOM	1493	CG	ASN	B	154	-33.998	7.039	58.118	1.00	0.00	C
ATOM	1494	OD1	ASN	B	154	-33.209	7.794	58.675	1.00	0.00	O
ATOM	1495	ND2	ASN	B	154	-35.028	7.440	57.371	1.00	0.00	N

ATOM	1496	HD21	ASN	B	154	-35.208	8.413	57.240	1.00	0.00	H
ATOM	1497	HD22	ASN	B	154	-35.631	6.770	56.925	1.00	0.00	H
ATOM	1498	C	ASN	B	154	-31.716	4.968	57.036	1.00	0.00	C
ATOM	1499	O	ASN	B	154	-31.108	3.906	57.008	1.00	0.00	O
ATOM	1500	N	ALA	B	155	-31.139	6.177	57.109	1.00	0.00	N
ATOM	1501	H	ALA	B	155	-31.693	6.995	57.254	1.00	0.00	H
ATOM	1502	CA	ALA	B	155	-29.682	6.261	57.091	1.00	0.00	C
ATOM	1503	CB	ALA	B	155	-29.251	7.704	56.818	1.00	0.00	C
ATOM	1504	C	ALA	B	155	-29.040	5.773	58.378	1.00	0.00	C
ATOM	1505	O	ALA	B	155	-29.715	5.418	59.336	1.00	0.00	O
ATOM	1506	N	ALA	B	156	-27.699	5.800	58.353	1.00	0.00	N
ATOM	1507	H	ALA	B	156	-27.245	6.084	57.512	1.00	0.00	H
ATOM	1508	CA	ALA	B	156	-26.901	5.290	59.468	1.00	0.00	C
ATOM	1509	CB	ALA	B	156	-25.440	5.717	59.301	1.00	0.00	C
ATOM	1510	C	ALA	B	156	-27.335	5.677	60.873	1.00	0.00	C
ATOM	1511	O	ALA	B	156	-27.867	6.756	61.126	1.00	0.00	O
ATOM	1512	N	ILE	B	157	-27.039	4.754	61.796	1.00	0.00	N
ATOM	1513	H	ILE	B	157	-26.723	3.849	61.507	1.00	0.00	H
ATOM	1514	CA	ILE	B	157	-26.863	5.235	63.164	1.00	0.00	C
ATOM	1515	CB	ILE	B	157	-27.216	4.151	64.189	1.00	0.00	C
ATOM	1516	CG2	ILE	B	157	-27.115	4.686	65.623	1.00	0.00	C
ATOM	1517	CG1	ILE	B	157	-28.604	3.572	63.903	1.00	0.00	C
ATOM	1518	CD1	ILE	B	157	-29.015	2.482	64.892	1.00	0.00	C
ATOM	1519	C	ILE	B	157	-25.426	5.703	63.314	1.00	0.00	C
ATOM	1520	O	ILE	B	157	-24.485	4.966	63.054	1.00	0.00	O
ATOM	1521	N	VAL	B	158	-25.305	6.988	63.661	1.00	0.00	N
ATOM	1522	H	VAL	B	158	-26.081	7.493	64.032	1.00	0.00	H
ATOM	1523	CA	VAL	B	158	-23.980	7.593	63.544	1.00	0.00	C
ATOM	1524	CB	VAL	B	158	-24.102	9.124	63.467	1.00	0.00	C
ATOM	1525	CG1	VAL	B	158	-22.750	9.811	63.241	1.00	0.00	C
ATOM	1526	CG2	VAL	B	158	-25.110	9.527	62.386	1.00	0.00	C
ATOM	1527	C	VAL	B	158	-23.027	7.162	64.645	1.00	0.00	C
ATOM	1528	O	VAL	B	158	-23.313	7.281	65.829	1.00	0.00	O
ATOM	1529	N	LEU	B	159	-21.873	6.659	64.184	1.00	0.00	N
ATOM	1530	H	LEU	B	159	-21.789	6.522	63.197	1.00	0.00	H
ATOM	1531	CA	LEU	B	159	-20.798	6.289	65.105	1.00	0.00	C
ATOM	1532	CB	LEU	B	159	-19.597	5.776	64.297	1.00	0.00	C
ATOM	1533	CG	LEU	B	159	-18.351	5.382	65.101	1.00	0.00	C
ATOM	1534	CD1	LEU	B	159	-18.666	4.431	66.254	1.00	0.00	C
ATOM	1535	CD2	LEU	B	159	-17.252	4.838	64.189	1.00	0.00	C
ATOM	1536	C	LEU	B	159	-20.394	7.376	66.092	1.00	0.00	C
ATOM	1537	O	LEU	B	159	-19.715	8.342	65.767	1.00	0.00	O
ATOM	1538	N	GLN	B	160	-20.819	7.136	67.336	1.00	0.00	N
ATOM	1539	H	GLN	B	160	-21.476	6.404	67.509	1.00	0.00	H
ATOM	1540	CA	GLN	B	160	-20.315	7.986	68.404	1.00	0.00	C
ATOM	1541	CB	GLN	B	160	-21.470	8.768	69.029	1.00	0.00	C
ATOM	1542	CG	GLN	B	160	-21.006	9.905	69.941	1.00	0.00	C
ATOM	1543	CD	GLN	B	160	-22.216	10.676	70.422	1.00	0.00	C
ATOM	1544	OE1	GLN	B	160	-22.857	10.348	71.408	1.00	0.00	O
ATOM	1545	NE2	GLN	B	160	-22.508	11.741	69.671	1.00	0.00	N
ATOM	1546	HE21	GLN	B	160	-23.294	12.289	69.947	1.00	0.00	H
ATOM	1547	HE22	GLN	B	160	-21.969	11.979	68.866	1.00	0.00	H
ATOM	1548	C	GLN	B	160	-19.555	7.169	69.428	1.00	0.00	C
ATOM	1549	O	GLN	B	160	-20.117	6.353	70.149	1.00	0.00	O
ATOM	1550	N	LEU	B	161	-18.240	7.406	69.443	1.00	0.00	N
ATOM	1551	H	LEU	B	161	-17.860	8.139	68.883	1.00	0.00	H
ATOM	1552	CA	LEU	B	161	-17.436	6.726	70.458	1.00	0.00	C
ATOM	1553	CB	LEU	B	161	-16.004	6.530	69.951	1.00	0.00	C
ATOM	1554	CG	LEU	B	161	-15.886	5.501	68.828	1.00	0.00	C

ATOM	1555	CD1	LEU	B	161	-14.459	5.429	68.292	1.00	0.00	C
ATOM	1556	CD2	LEU	B	161	-16.386	4.122	69.255	1.00	0.00	C
ATOM	1557	C	LEU	B	161	-17.421	7.507	71.758	1.00	0.00	C
ATOM	1558	O	LEU	B	161	-17.450	8.732	71.748	1.00	0.00	O
ATOM	1559	N	PRO	B	162	-17.369	6.766	72.893	1.00	0.00	N
ATOM	1560	CD	PRO	B	162	-17.481	5.316	73.020	1.00	0.00	C
ATOM	1561	CA	PRO	B	162	-17.180	7.432	74.187	1.00	0.00	C
ATOM	1562	CB	PRO	B	162	-17.029	6.260	75.161	1.00	0.00	C
ATOM	1563	CG	PRO	B	162	-17.761	5.098	74.501	1.00	0.00	C
ATOM	1564	C	PRO	B	162	-15.967	8.345	74.209	1.00	0.00	C
ATOM	1565	O	PRO	B	162	-14.907	8.028	73.672	1.00	0.00	O
ATOM	1566	N	GLN	B	163	-16.163	9.497	74.865	1.00	0.00	N
ATOM	1567	H	GLN	B	163	-17.044	9.684	75.297	1.00	0.00	H
ATOM	1568	CA	GLN	B	163	-15.035	10.415	75.001	1.00	0.00	C
ATOM	1569	CB	GLN	B	163	-15.493	11.683	75.727	1.00	0.00	C
ATOM	1570	CG	GLN	B	163	-14.467	12.821	75.734	1.00	0.00	C
ATOM	1571	CD	GLN	B	163	-15.040	14.021	76.462	1.00	0.00	C
ATOM	1572	OE1	GLN	B	163	-16.094	13.976	77.078	1.00	0.00	O
ATOM	1573	NE2	GLN	B	163	-14.293	15.122	76.360	1.00	0.00	N
ATOM	1574	HE21	GLN	B	163	-14.635	15.939	76.821	1.00	0.00	H
ATOM	1575	HE22	GLN	B	163	-13.434	15.142	75.853	1.00	0.00	H
ATOM	1576	C	GLN	B	163	-13.851	9.761	75.695	1.00	0.00	C
ATOM	1577	O	GLN	B	163	-13.971	9.182	76.765	1.00	0.00	O
ATOM	1578	N	GLY	B	164	-12.707	9.853	75.007	1.00	0.00	N
ATOM	1579	H	GLY	B	164	-12.694	10.275	74.103	1.00	0.00	H
ATOM	1580	CA	GLY	B	164	-11.539	9.195	75.584	1.00	0.00	C
ATOM	1581	C	GLY	B	164	-11.355	7.742	75.180	1.00	0.00	C
ATOM	1582	O	GLY	B	164	-10.575	7.012	75.774	1.00	0.00	O
ATOM	1583	N	THR	B	165	-12.076	7.345	74.117	1.00	0.00	N
ATOM	1584	H	THR	B	165	-12.760	7.936	73.694	1.00	0.00	H
ATOM	1585	CA	THR	B	165	-11.704	6.058	73.528	1.00	0.00	C
ATOM	1586	CB	THR	B	165	-12.761	5.630	72.499	1.00	0.00	C
ATOM	1587	OG1	THR	B	165	-14.026	5.454	73.153	1.00	0.00	O
ATOM	1588	HG1	THR	B	165	-14.276	6.323	73.459	1.00	0.00	H
ATOM	1589	CG2	THR	B	165	-12.394	4.355	71.739	1.00	0.00	C
ATOM	1590	C	THR	B	165	-10.297	6.069	72.940	1.00	0.00	C
ATOM	1591	O	THR	B	165	-9.931	6.932	72.152	1.00	0.00	O
ATOM	1592	N	THR	B	166	-9.525	5.071	73.382	1.00	0.00	N
ATOM	1593	H	THR	B	166	-9.914	4.387	73.996	1.00	0.00	H
ATOM	1594	CA	THR	B	166	-8.181	4.917	72.840	1.00	0.00	C
ATOM	1595	CB	THR	B	166	-7.382	3.970	73.739	1.00	0.00	C
ATOM	1596	OG1	THR	B	166	-7.555	4.333	75.116	1.00	0.00	O
ATOM	1597	HG1	THR	B	166	-8.471	4.169	75.326	1.00	0.00	H
ATOM	1598	CG2	THR	B	166	-5.894	3.931	73.377	1.00	0.00	C
ATOM	1599	C	THR	B	166	-8.247	4.402	71.414	1.00	0.00	C
ATOM	1600	O	THR	B	166	-8.899	3.406	71.129	1.00	0.00	O
ATOM	1601	N	LEU	B	167	-7.583	5.151	70.527	1.00	0.00	N
ATOM	1602	H	LEU	B	167	-7.025	5.926	70.817	1.00	0.00	H
ATOM	1603	CA	LEU	B	167	-7.717	4.792	69.119	1.00	0.00	C
ATOM	1604	CB	LEU	B	167	-7.986	6.045	68.283	1.00	0.00	C
ATOM	1605	CG	LEU	B	167	-9.209	6.854	68.732	1.00	0.00	C
ATOM	1606	CD1	LEU	B	167	-9.365	8.131	67.908	1.00	0.00	C
ATOM	1607	CD2	LEU	B	167	-10.496	6.027	68.741	1.00	0.00	C
ATOM	1608	C	LEU	B	167	-6.510	4.040	68.587	1.00	0.00	C
ATOM	1609	O	LEU	B	167	-5.404	4.566	68.535	1.00	0.00	O
ATOM	1610	N	PRO	B	168	-6.757	2.767	68.199	1.00	0.00	N
ATOM	1611	CD	PRO	B	168	-8.011	2.037	68.315	1.00	0.00	C
ATOM	1612	CA	PRO	B	168	-5.686	1.949	67.619	1.00	0.00	C
ATOM	1613	CB	PRO	B	168	-6.398	0.631	67.305	1.00	0.00	C

ATOM	1614	CG	PRO	B	168	-7.577	0.579	68.272	1.00	0.00	C
ATOM	1615	C	PRO	B	168	-5.030	2.586	66.405	1.00	0.00	C
ATOM	1616	O	PRO	B	168	-5.664	3.225	65.570	1.00	0.00	O
ATOM	1617	N	LYS	B	169	-3.706	2.405	66.365	1.00	0.00	N
ATOM	1618	H	LYS	B	169	-3.326	1.771	67.042	1.00	0.00	H
ATOM	1619	CA	LYS	B	169	-2.873	3.146	65.417	1.00	0.00	C
ATOM	1620	CB	LYS	B	169	-1.402	2.760	65.596	1.00	0.00	C
ATOM	1621	CG	LYS	B	169	-0.966	2.953	67.050	1.00	0.00	C
ATOM	1622	CD	LYS	B	169	0.385	2.324	67.394	1.00	0.00	C
ATOM	1623	CE	LYS	B	169	0.610	2.248	68.909	1.00	0.00	C
ATOM	1624	NZ	LYS	B	169	-0.343	1.306	69.510	1.00	0.00	N
ATOM	1625	HZ1	LYS	B	169	-0.303	1.249	70.541	1.00	0.00	H
ATOM	1626	HZ2	LYS	B	169	-1.331	1.488	69.228	1.00	0.00	H
ATOM	1627	HZ3	LYS	B	169	-0.205	0.341	69.137	1.00	0.00	H
ATOM	1628	C	LYS	B	169	-3.302	3.079	63.960	1.00	0.00	C
ATOM	1629	O	LYS	B	169	-3.231	2.061	63.276	1.00	0.00	O
ATOM	1630	N	GLY	B	170	-3.751	4.266	63.529	1.00	0.00	N
ATOM	1631	H	GLY	B	170	-3.830	5.008	64.193	1.00	0.00	H
ATOM	1632	CA	GLY	B	170	-4.258	4.397	62.168	1.00	0.00	C
ATOM	1633	C	GLY	B	170	-5.763	4.237	62.040	1.00	0.00	C
ATOM	1634	O	GLY	B	170	-6.289	3.842	61.003	1.00	0.00	O
ATOM	1635	N	PHE	B	171	-6.416	4.592	63.154	1.00	0.00	N
ATOM	1636	H	PHE	B	171	-5.912	4.713	64.008	1.00	0.00	H
ATOM	1637	CA	PHE	B	171	-7.870	4.703	63.205	1.00	0.00	C
ATOM	1638	CB	PHE	B	171	-8.233	5.234	64.595	1.00	0.00	C
ATOM	1639	CG	PHE	B	171	-9.717	5.186	64.875	1.00	0.00	C
ATOM	1640	CD1	PHE	B	171	-10.246	4.075	65.563	1.00	0.00	C
ATOM	1641	CD2	PHE	B	171	-10.541	6.256	64.462	1.00	0.00	C
ATOM	1642	CE1	PHE	B	171	-11.624	4.036	65.845	1.00	0.00	C
ATOM	1643	CE2	PHE	B	171	-11.920	6.213	64.735	1.00	0.00	C
ATOM	1644	CZ	PHE	B	171	-12.447	5.101	65.422	1.00	0.00	C
ATOM	1645	C	PHE	B	171	-8.470	5.570	62.111	1.00	0.00	C
ATOM	1646	O	PHE	B	171	-8.078	6.714	61.915	1.00	0.00	O
ATOM	1647	N	TYR	B	172	-9.460	4.974	61.442	1.00	0.00	N
ATOM	1648	H	TYR	B	172	-9.663	4.003	61.598	1.00	0.00	H
ATOM	1649	CA	TYR	B	172	-10.348	5.732	60.566	1.00	0.00	C
ATOM	1650	CB	TYR	B	172	-9.874	5.700	59.104	1.00	0.00	C
ATOM	1651	CG	TYR	B	172	-8.844	6.773	58.842	1.00	0.00	C
ATOM	1652	CD1	TYR	B	172	-7.474	6.445	58.887	1.00	0.00	C
ATOM	1653	CE1	TYR	B	172	-6.525	7.450	58.638	1.00	0.00	C
ATOM	1654	CD2	TYR	B	172	-9.290	8.079	58.554	1.00	0.00	C
ATOM	1655	CE2	TYR	B	172	-8.342	9.082	58.300	1.00	0.00	C
ATOM	1656	CZ	TYR	B	172	-6.973	8.754	58.341	1.00	0.00	C
ATOM	1657	OH	TYR	B	172	-6.044	9.741	58.072	1.00	0.00	O
ATOM	1658	HH	TYR	B	172	-5.224	9.522	58.495	1.00	0.00	H
ATOM	1659	C	TYR	B	172	-11.735	5.132	60.685	1.00	0.00	C
ATOM	1660	O	TYR	B	172	-11.956	4.186	61.433	1.00	0.00	O
ATOM	1661	N	ALA	B	173	-12.666	5.705	59.916	1.00	0.00	N
ATOM	1662	H	ALA	B	173	-12.471	6.474	59.305	1.00	0.00	H
ATOM	1663	CA	ALA	B	173	-14.004	5.126	59.898	1.00	0.00	C
ATOM	1664	CB	ALA	B	173	-14.880	5.727	61.002	1.00	0.00	C
ATOM	1665	C	ALA	B	173	-14.632	5.423	58.561	1.00	0.00	C
ATOM	1666	O	ALA	B	173	-14.170	6.298	57.844	1.00	0.00	O
ATOM	1667	N	GLU	B	174	-15.716	4.699	58.270	1.00	0.00	N
ATOM	1668	H	GLU	B	174	-16.042	4.034	58.939	1.00	0.00	H
ATOM	1669	CA	GLU	B	174	-16.457	4.954	57.036	1.00	0.00	C
ATOM	1670	CB	GLU	B	174	-17.188	3.680	56.594	1.00	0.00	C
ATOM	1671	CG	GLU	B	174	-16.330	2.649	55.845	1.00	0.00	C
ATOM	1672	CD	GLU	B	174	-16.656	2.625	54.355	1.00	0.00	C

ATOM	1673	OE1	GLU	B	174	-17.828	2.660	53.989	1.00	0.00	O
ATOM	1674	OE2	GLU	B	174	-15.745	2.564	53.537	1.00	0.00	O
ATOM	1675	C	GLU	B	174	-17.437	6.124	57.110	1.00	0.00	C
ATOM	1676	O	GLU	B	174	-18.522	6.079	56.538	1.00	0.00	O
ATOM	1677	N	GLY	B	175	-17.018	7.171	57.838	1.00	0.00	N
ATOM	1678	H	GLY	B	175	-16.042	7.209	58.055	1.00	0.00	H
ATOM	1679	CA	GLY	B	175	-17.848	8.375	57.882	1.00	0.00	C
ATOM	1680	C	GLY	B	175	-17.430	9.292	56.753	1.00	0.00	C
ATOM	1681	O	GLY	B	175	-17.949	9.237	55.643	1.00	0.00	O
ATOM	1682	N	SER	B	176	-16.379	10.058	57.073	1.00	0.00	N
ATOM	1683	H	SER	B	176	-16.037	10.136	58.009	1.00	0.00	H
ATOM	1684	CA	SER	B	176	-15.461	10.500	56.024	1.00	0.00	C
ATOM	1685	CB	SER	B	176	-14.586	11.605	56.619	1.00	0.00	C
ATOM	1686	OG	SER	B	176	-14.443	11.394	58.035	1.00	0.00	O
ATOM	1687	HG	SER	B	176	-13.803	12.034	58.324	1.00	0.00	H
ATOM	1688	C	SER	B	176	-14.656	9.292	55.552	1.00	0.00	C
ATOM	1689	O	SER	B	176	-15.010	8.167	55.884	1.00	0.00	O
ATOM	1690	N	ARG	B	177	-13.601	9.514	54.770	1.00	0.00	N
ATOM	1691	H	ARG	B	177	-13.403	10.421	54.395	1.00	0.00	H
ATOM	1692	CA	ARG	B	177	-12.671	8.414	54.524	1.00	0.00	C
ATOM	1693	CB	ARG	B	177	-12.765	7.871	53.093	1.00	0.00	C
ATOM	1694	CG	ARG	B	177	-14.109	7.228	52.746	1.00	0.00	C
ATOM	1695	CD	ARG	B	177	-14.436	6.032	53.646	1.00	0.00	C
ATOM	1696	NE	ARG	B	177	-15.764	5.463	53.397	1.00	0.00	N
ATOM	1697	HE	ARG	B	177	-15.820	4.507	53.092	1.00	0.00	H
ATOM	1698	CZ	ARG	B	177	-16.915	6.074	53.755	1.00	0.00	C
ATOM	1699	NH1	ARG	B	177	-16.912	7.308	54.233	1.00	0.00	N
ATOM	1700	HH11	ARG	B	177	-16.036	7.760	54.417	1.00	0.00	H
ATOM	1701	HH12	ARG	B	177	-17.737	7.829	54.481	1.00	0.00	H
ATOM	1702	NH2	ARG	B	177	-18.056	5.407	53.673	1.00	0.00	N
ATOM	1703	HH21	ARG	B	177	-18.041	4.434	53.413	1.00	0.00	H
ATOM	1704	HH22	ARG	B	177	-18.926	5.834	53.917	1.00	0.00	H
ATOM	1705	C	ARG	B	177	-11.251	8.840	54.819	1.00	0.00	C
ATOM	1706	O	ARG	B	177	-10.513	8.171	55.536	1.00	0.00	O
ATOM	1707	N	GLY	B	178	-10.913	10.009	54.246	1.00	0.00	N
ATOM	1708	H	GLY	B	178	-11.568	10.503	53.661	1.00	0.00	H
ATOM	1709	CA	GLY	B	178	-9.588	10.570	54.503	1.00	0.00	C
ATOM	1710	C	GLY	B	178	-8.437	9.635	54.176	1.00	0.00	C
ATOM	1711	O	GLY	B	178	-8.458	8.879	53.209	1.00	0.00	O
ATOM	1712	N	GLY	B	179	-7.427	9.712	55.051	1.00	0.00	N
ATOM	1713	H	GLY	B	179	-7.444	10.379	55.794	1.00	0.00	H
ATOM	1714	CA	GLY	B	179	-6.212	8.971	54.739	1.00	0.00	C
ATOM	1715	C	GLY	B	179	-5.288	9.794	53.868	1.00	0.00	C
ATOM	1716	O	GLY	B	179	-5.573	10.930	53.511	1.00	0.00	O
ATOM	1717	N	SER	B	180	-4.158	9.163	53.548	1.00	0.00	N
ATOM	1718	H	SER	B	180	-3.983	8.245	53.899	1.00	0.00	H
ATOM	1719	CA	SER	B	180	-3.255	9.802	52.604	1.00	0.00	C
ATOM	1720	CB	SER	B	180	-2.118	10.517	53.339	1.00	0.00	C
ATOM	1721	OG	SER	B	180	-1.404	11.368	52.434	1.00	0.00	O
ATOM	1722	HG	SER	B	180	-1.149	12.132	52.940	1.00	0.00	H
ATOM	1723	C	SER	B	180	-2.739	8.799	51.595	1.00	0.00	C
ATOM	1724	O	SER	B	180	-2.563	7.613	51.877	1.00	0.00	O
ATOM	1725	N	GLN	B	181	-2.535	9.335	50.380	1.00	0.00	N
ATOM	1726	H	GLN	B	181	-2.685	10.322	50.318	1.00	0.00	H
ATOM	1727	CA	GLN	B	181	-2.116	8.542	49.224	1.00	0.00	C
ATOM	1728	CB	GLN	B	181	-0.587	8.536	49.129	1.00	0.00	C
ATOM	1729	CG	GLN	B	181	0.029	9.936	49.113	1.00	0.00	C
ATOM	1730	CD	GLN	B	181	1.536	9.806	49.057	1.00	0.00	C
ATOM	1731	OE1	GLN	B	181	2.086	8.757	48.758	1.00	0.00	O

ATOM	1732	NE2	GLN	B	181	2.191	10.925	49.368	1.00	0.00	N
ATOM	1733	HE21	GLN	B	181	3.189	10.890	49.345	1.00	0.00	H
ATOM	1734	HE22	GLN	B	181	1.711	11.764	49.615	1.00	0.00	H
ATOM	1735	C	GLN	B	181	-2.752	7.150	49.121	1.00	0.00	C
ATOM	1736	O	GLN	B	181	-3.939	7.018	48.830	1.00	0.00	O
ATOM	1737	N	ALA	B	182	-1.937	6.109	49.380	1.00	0.00	N
ATOM	1738	H	ALA	B	182	-1.012	6.243	49.733	1.00	0.00	H
ATOM	1739	CA	ALA	B	182	-2.446	4.750	49.193	1.00	0.00	C
ATOM	1740	CB	ALA	B	182	-1.319	3.732	49.369	1.00	0.00	C
ATOM	1741	C	ALA	B	182	-3.603	4.363	50.101	1.00	0.00	C
ATOM	1742	O	ALA	B	182	-4.490	3.595	49.737	1.00	0.00	O
ATOM	1743	N	SER	B	183	-3.566	4.943	51.312	1.00	0.00	N
ATOM	1744	H	SER	B	183	-2.914	5.679	51.495	1.00	0.00	H
ATOM	1745	CA	SER	B	183	-4.668	4.623	52.213	1.00	0.00	C
ATOM	1746	CB	SER	B	183	-4.341	5.012	53.659	1.00	0.00	C
ATOM	1747	OG	SER	B	183	-3.896	6.369	53.735	1.00	0.00	O
ATOM	1748	HG	SER	B	183	-3.064	6.428	53.269	1.00	0.00	H
ATOM	1749	C	SER	B	183	-5.990	5.181	51.718	1.00	0.00	C
ATOM	1750	O	SER	B	183	-6.954	4.446	51.544	1.00	0.00	O
ATOM	1751	N	SER	B	184	-5.953	6.484	51.400	1.00	0.00	N
ATOM	1752	H	SER	B	184	-5.141	7.040	51.588	1.00	0.00	H
ATOM	1753	CA	SER	B	184	-7.119	7.151	50.813	1.00	0.00	C
ATOM	1754	CB	SER	B	184	-6.757	8.606	50.514	1.00	0.00	C
ATOM	1755	OG	SER	B	184	-5.336	8.707	50.344	1.00	0.00	O
ATOM	1756	HG	SER	B	184	-5.135	8.361	49.479	1.00	0.00	H
ATOM	1757	C	SER	B	184	-7.716	6.465	49.592	1.00	0.00	C
ATOM	1758	O	SER	B	184	-8.928	6.363	49.425	1.00	0.00	O
ATOM	1759	N	ARG	B	185	-6.801	5.924	48.755	1.00	0.00	N
ATOM	1760	H	ARG	B	185	-5.825	6.086	48.921	1.00	0.00	H
ATOM	1761	CA	ARG	B	185	-7.280	5.173	47.587	1.00	0.00	C
ATOM	1762	CB	ARG	B	185	-6.133	4.609	46.731	1.00	0.00	C
ATOM	1763	CG	ARG	B	185	-5.083	5.623	46.260	1.00	0.00	C
ATOM	1764	CD	ARG	B	185	-4.066	5.047	45.262	1.00	0.00	C
ATOM	1765	NE	ARG	B	185	-4.705	4.826	43.966	1.00	0.00	N
ATOM	1766	HE	ARG	B	185	-5.703	4.917	43.917	1.00	0.00	H
ATOM	1767	CZ	ARG	B	185	-4.046	4.584	42.812	1.00	0.00	C
ATOM	1768	NH1	ARG	B	185	-2.725	4.426	42.799	1.00	0.00	N
ATOM	1769	HH11	ARG	B	185	-2.204	4.483	43.651	1.00	0.00	H
ATOM	1770	HH12	ARG	B	185	-2.231	4.251	41.947	1.00	0.00	H
ATOM	1771	NH2	ARG	B	185	-4.755	4.518	41.690	1.00	0.00	N
ATOM	1772	HH21	ARG	B	185	-5.744	4.692	41.741	1.00	0.00	H
ATOM	1773	HH22	ARG	B	185	-4.403	4.326	40.768	1.00	0.00	H
ATOM	1774	C	ARG	B	185	-8.270	4.048	47.889	1.00	0.00	C
ATOM	1775	O	ARG	B	185	-9.078	3.659	47.052	1.00	0.00	O
ATOM	1776	N	SER	B	186	-8.178	3.549	49.131	1.00	0.00	N
ATOM	1777	H	SER	B	186	-7.553	3.987	49.774	1.00	0.00	H
ATOM	1778	CA	SER	B	186	-9.071	2.500	49.624	1.00	0.00	C
ATOM	1779	CB	SER	B	186	-8.386	1.775	50.782	1.00	0.00	C
ATOM	1780	OG	SER	B	186	-6.967	1.755	50.539	1.00	0.00	O
ATOM	1781	HG	SER	B	186	-6.665	2.594	50.896	1.00	0.00	H
ATOM	1782	C	SER	B	186	-10.474	2.957	50.017	1.00	0.00	C
ATOM	1783	O	SER	B	186	-10.972	2.710	51.115	1.00	0.00	O
ATOM	1784	N	SER	B	187	-11.096	3.630	49.043	1.00	0.00	N
ATOM	1785	H	SER	B	187	-10.626	3.772	48.174	1.00	0.00	H
ATOM	1786	CA	SER	B	187	-12.451	4.134	49.225	1.00	0.00	C
ATOM	1787	CB	SER	B	187	-12.416	5.396	50.093	1.00	0.00	C
ATOM	1788	OG	SER	B	187	-11.673	6.440	49.444	1.00	0.00	O
ATOM	1789	HG	SER	B	187	-10.778	6.410	49.787	1.00	0.00	H
ATOM	1790	C	SER	B	187	-13.103	4.404	47.881	1.00	0.00	C

ATOM	1791	O	SER	B	187	-14.197	3.956	47.564	1.00	0.00	O
ATOM	1792	N	SER	B	188	-12.308	5.152	47.099	1.00	0.00	N
ATOM	1793	H	SER	B	188	-11.495	5.551	47.525	1.00	0.00	H
ATOM	1794	CA	SER	B	188	-12.634	5.642	45.766	1.00	0.00	C
ATOM	1795	CB	SER	B	188	-11.354	5.687	44.942	1.00	0.00	C
ATOM	1796	OG	SER	B	188	-10.215	5.900	45.796	1.00	0.00	O
ATOM	1797	HG	SER	B	188	-9.931	5.025	46.055	1.00	0.00	H
ATOM	1798	C	SER	B	188	-13.738	4.973	44.970	1.00	0.00	C
ATOM	1799	O	SER	B	188	-13.521	4.049	44.187	1.00	0.00	O
ATOM	1800	N	ARG	B	189	-14.944	5.528	45.145	1.00	0.00	N
ATOM	1801	H	ARG	B	189	-15.064	6.259	45.815	1.00	0.00	H
ATOM	1802	CA	ARG	B	189	-15.943	5.186	44.139	1.00	0.00	C
ATOM	1803	CB	ARG	B	189	-17.344	5.549	44.627	1.00	0.00	C
ATOM	1804	CG	ARG	B	189	-17.717	4.582	45.750	1.00	0.00	C
ATOM	1805	CD	ARG	B	189	-19.166	4.674	46.227	1.00	0.00	C
ATOM	1806	NE	ARG	B	189	-19.488	3.490	47.027	1.00	0.00	N
ATOM	1807	HE	ARG	B	189	-19.224	3.518	47.992	1.00	0.00	H
ATOM	1808	CZ	ARG	B	189	-19.953	2.374	46.419	1.00	0.00	C
ATOM	1809	NH1	ARG	B	189	-20.339	2.405	45.147	1.00	0.00	N
ATOM	1810	HH11	ARG	B	189	-20.366	3.263	44.633	1.00	0.00	H
ATOM	1811	HH12	ARG	B	189	-20.614	1.548	44.698	1.00	0.00	H
ATOM	1812	NH2	ARG	B	189	-20.024	1.223	47.080	1.00	0.00	N
ATOM	1813	HH21	ARG	B	189	-19.784	1.152	48.047	1.00	0.00	H
ATOM	1814	HH22	ARG	B	189	-20.322	0.394	46.590	1.00	0.00	H
ATOM	1815	C	ARG	B	189	-15.583	5.811	42.806	1.00	0.00	C
ATOM	1816	O	ARG	B	189	-14.765	6.721	42.746	1.00	0.00	O
ATOM	1817	N	SER	B	190	-16.167	5.238	41.742	1.00	0.00	N
ATOM	1818	H	SER	B	190	-16.926	4.604	41.872	1.00	0.00	H
ATOM	1819	CA	SER	B	190	-15.748	5.619	40.392	1.00	0.00	C
ATOM	1820	CB	SER	B	190	-16.148	4.521	39.410	1.00	0.00	C
ATOM	1821	OG	SER	B	190	-15.228	3.422	39.531	1.00	0.00	O
ATOM	1822	HG	SER	B	190	-14.758	3.479	38.697	1.00	0.00	H
ATOM	1823	C	SER	B	190	-16.198	6.990	39.918	1.00	0.00	C
ATOM	1824	O	SER	B	190	-17.063	7.145	39.067	1.00	0.00	O
ATOM	1825	N	ARG	B	191	-15.521	7.969	40.524	1.00	0.00	N
ATOM	1826	H	ARG	B	191	-14.774	7.658	41.110	1.00	0.00	H
ATOM	1827	CA	ARG	B	191	-15.752	9.398	40.338	1.00	0.00	C
ATOM	1828	CB	ARG	B	191	-17.160	9.807	40.790	1.00	0.00	C
ATOM	1829	CG	ARG	B	191	-17.931	10.491	39.653	1.00	0.00	C
ATOM	1830	CD	ARG	B	191	-18.028	12.017	39.763	1.00	0.00	C
ATOM	1831	NE	ARG	B	191	-16.742	12.616	40.103	1.00	0.00	N
ATOM	1832	HE	ARG	B	191	-15.918	12.276	39.642	1.00	0.00	H
ATOM	1833	CZ	ARG	B	191	-16.635	13.515	41.101	1.00	0.00	C
ATOM	1834	NH1	ARG	B	191	-17.710	14.024	41.689	1.00	0.00	N
ATOM	1835	HH11	ARG	B	191	-18.642	13.770	41.403	1.00	0.00	H
ATOM	1836	HH12	ARG	B	191	-17.623	14.677	42.446	1.00	0.00	H
ATOM	1837	NH2	ARG	B	191	-15.425	13.876	41.509	1.00	0.00	N
ATOM	1838	HH21	ARG	B	191	-14.621	13.420	41.115	1.00	0.00	H
ATOM	1839	HH22	ARG	B	191	-15.307	14.580	42.212	1.00	0.00	H
ATOM	1840	C	ARG	B	191	-14.663	10.221	41.011	1.00	0.00	C
ATOM	1841	O	ARG	B	191	-14.394	11.368	40.661	1.00	0.00	O
ATOM	1842	N	ASN	B	192	-13.999	9.560	41.974	1.00	0.00	N
ATOM	1843	H	ASN	B	192	-14.296	8.673	42.330	1.00	0.00	H
ATOM	1844	CA	ASN	B	192	-12.635	10.011	42.227	1.00	0.00	C
ATOM	1845	CB	ASN	B	192	-12.261	9.869	43.707	1.00	0.00	C
ATOM	1846	CG	ASN	B	192	-10.990	10.652	44.005	1.00	0.00	C
ATOM	1847	OD1	ASN	B	192	-10.156	10.904	43.146	1.00	0.00	O
ATOM	1848	ND2	ASN	B	192	-10.879	11.054	45.272	1.00	0.00	N
ATOM	1849	HD21	ASN	B	192	-10.056	11.557	45.528	1.00	0.00	H

ATOM	1850	HD22	ASN	B	192	-11.581	10.874	45.958	1.00	0.00	H
ATOM	1851	C	ASN	B	192	-11.680	9.219	41.354	1.00	0.00	C
ATOM	1852	O	ASN	B	192	-11.732	7.995	41.287	1.00	0.00	O
ATOM	1853	N	SER	B	193	-10.808	9.978	40.687	1.00	0.00	N
ATOM	1854	H	SER	B	193	-10.821	10.974	40.777	1.00	0.00	H
ATOM	1855	CA	SER	B	193	-9.766	9.383	39.858	1.00	0.00	C
ATOM	1856	CB	SER	B	193	-9.005	10.511	39.166	1.00	0.00	C
ATOM	1857	OG	SER	B	193	-9.934	11.548	38.817	1.00	0.00	O
ATOM	1858	HG	SER	B	193	-9.429	12.222	38.378	1.00	0.00	H
ATOM	1859	C	SER	B	193	-8.807	8.441	40.576	1.00	0.00	C
ATOM	1860	O	SER	B	193	-8.112	7.642	39.962	1.00	0.00	O
ATOM	1861	N	SER	B	194	-8.804	8.536	41.915	1.00	0.00	N
ATOM	1862	H	SER	B	194	-9.367	9.209	42.401	1.00	0.00	H
ATOM	1863	CA	SER	B	194	-7.912	7.692	42.703	1.00	0.00	C
ATOM	1864	CB	SER	B	194	-8.074	8.044	44.181	1.00	0.00	C
ATOM	1865	OG	SER	B	194	-9.466	8.193	44.474	1.00	0.00	O
ATOM	1866	HG	SER	B	194	-9.651	7.606	45.207	1.00	0.00	H
ATOM	1867	C	SER	B	194	-7.993	6.186	42.476	1.00	0.00	C
ATOM	1868	O	SER	B	194	-7.021	5.468	42.687	1.00	0.00	O
ATOM	1869	N	ARG	B	195	-9.158	5.698	42.025	1.00	0.00	N
ATOM	1870	H	ARG	B	195	-9.929	6.304	41.819	1.00	0.00	H
ATOM	1871	CA	ARG	B	195	-9.144	4.251	41.784	1.00	0.00	C
ATOM	1872	CB	ARG	B	195	-10.493	3.617	42.104	1.00	0.00	C
ATOM	1873	CG	ARG	B	195	-10.334	2.314	42.897	1.00	0.00	C
ATOM	1874	CD	ARG	B	195	-11.659	1.601	43.174	1.00	0.00	C
ATOM	1875	NE	ARG	B	195	-12.251	1.080	41.943	1.00	0.00	N
ATOM	1876	HE	ARG	B	195	-11.826	0.280	41.511	1.00	0.00	H
ATOM	1877	CZ	ARG	B	195	-13.294	1.677	41.332	1.00	0.00	C
ATOM	1878	NH1	ARG	B	195	-13.860	2.769	41.828	1.00	0.00	N
ATOM	1879	HH11	ARG	B	195	-13.577	3.149	42.718	1.00	0.00	H
ATOM	1880	HH12	ARG	B	195	-14.576	3.234	41.299	1.00	0.00	H
ATOM	1881	NH2	ARG	B	195	-13.759	1.167	40.201	1.00	0.00	N
ATOM	1882	HH21	ARG	B	195	-13.327	0.342	39.818	1.00	0.00	H
ATOM	1883	HH22	ARG	B	195	-14.526	1.603	39.723	1.00	0.00	H
ATOM	1884	C	ARG	B	195	-8.647	3.796	40.419	1.00	0.00	C
ATOM	1885	O	ARG	B	195	-8.601	2.613	40.096	1.00	0.00	O
ATOM	1886	N	ASN	B	196	-8.288	4.799	39.611	1.00	0.00	N
ATOM	1887	H	ASN	B	196	-8.236	5.747	39.919	1.00	0.00	H
ATOM	1888	CA	ASN	B	196	-7.815	4.440	38.282	1.00	0.00	C
ATOM	1889	CB	ASN	B	196	-8.079	5.589	37.300	1.00	0.00	C
ATOM	1890	CG	ASN	B	196	-8.353	5.019	35.925	1.00	0.00	C
ATOM	1891	OD1	ASN	B	196	-8.295	3.818	35.708	1.00	0.00	O
ATOM	1892	ND2	ASN	B	196	-8.736	5.922	35.017	1.00	0.00	N
ATOM	1893	HD21	ASN	B	196	-9.054	5.601	34.125	1.00	0.00	H
ATOM	1894	HD22	ASN	B	196	-8.707	6.900	35.210	1.00	0.00	H
ATOM	1895	C	ASN	B	196	-6.355	4.029	38.283	1.00	0.00	C
ATOM	1896	O	ASN	B	196	-5.584	4.376	39.178	1.00	0.00	O
ATOM	1897	N	SER	B	197	-6.010	3.269	37.246	1.00	0.00	N
ATOM	1898	H	SER	B	197	-6.689	3.035	36.549	1.00	0.00	H
ATOM	1899	CA	SER	B	197	-4.591	3.150	36.954	1.00	0.00	C
ATOM	1900	CB	SER	B	197	-4.308	1.764	36.388	1.00	0.00	C
ATOM	1901	OG	SER	B	197	-4.965	0.775	37.192	1.00	0.00	O
ATOM	1902	HG	SER	B	197	-4.358	0.032	37.205	1.00	0.00	H
ATOM	1903	C	SER	B	197	-4.209	4.286	36.019	1.00	0.00	C
ATOM	1904	O	SER	B	197	-5.034	5.163	35.793	1.00	0.00	O
ATOM	1905	N	THR	B	198	-2.953	4.262	35.534	1.00	0.00	N
ATOM	1906	H	THR	B	198	-2.365	3.462	35.644	1.00	0.00	H
ATOM	1907	CA	THR	B	198	-2.355	5.357	34.753	1.00	0.00	C
ATOM	1908	CB	THR	B	198	-1.479	4.754	33.655	1.00	0.00	C

ATOM	1909	OG1	THR	B	198	-1.442	3.324	33.787	1.00	0.00	O
ATOM	1910	HG1	THR	B	198	-2.195	3.031	33.273	1.00	0.00	H
ATOM	1911	CG2	THR	B	198	-0.070	5.349	33.669	1.00	0.00	C
ATOM	1912	C	THR	B	198	-3.266	6.438	34.176	1.00	0.00	C
ATOM	1913	O	THR	B	198	-3.741	6.354	33.052	1.00	0.00	O
ATOM	1914	N	PRO	B	199	-3.516	7.468	35.016	1.00	0.00	N
ATOM	1915	CD	PRO	B	199	-2.906	7.731	36.317	1.00	0.00	C
ATOM	1916	CA	PRO	B	199	-4.584	8.407	34.677	1.00	0.00	C
ATOM	1917	CB	PRO	B	199	-4.883	9.046	36.037	1.00	0.00	C
ATOM	1918	CG	PRO	B	199	-3.548	9.033	36.783	1.00	0.00	C
ATOM	1919	C	PRO	B	199	-4.228	9.400	33.583	1.00	0.00	C
ATOM	1920	O	PRO	B	199	-3.790	10.519	33.827	1.00	0.00	O
ATOM	1921	N	GLY	B	200	-4.506	8.965	32.347	1.00	0.00	N
ATOM	1922	H	GLY	B	200	-4.763	8.006	32.195	1.00	0.00	H
ATOM	1923	CA	GLY	B	200	-4.671	9.974	31.307	1.00	0.00	C
ATOM	1924	C	GLY	B	200	-5.931	10.777	31.575	1.00	0.00	C
ATOM	1925	O	GLY	B	200	-6.741	10.430	32.430	1.00	0.00	O
ATOM	1926	N	SER	B	201	-6.075	11.883	30.833	1.00	0.00	N
ATOM	1927	H	SER	B	201	-5.437	12.071	30.088	1.00	0.00	H
ATOM	1928	CA	SER	B	201	-7.183	12.769	31.186	1.00	0.00	C
ATOM	1929	CB	SER	B	201	-6.853	14.224	30.817	1.00	0.00	C
ATOM	1930	OG	SER	B	201	-7.691	15.143	31.546	1.00	0.00	O
ATOM	1931	HG	SER	B	201	-7.121	15.489	32.232	1.00	0.00	H
ATOM	1932	C	SER	B	201	-8.554	12.359	30.661	1.00	0.00	C
ATOM	1933	O	SER	B	201	-9.171	13.047	29.849	1.00	0.00	O
ATOM	1934	N	SER	B	202	-9.049	11.240	31.212	1.00	0.00	N
ATOM	1935	H	SER	B	202	-8.506	10.751	31.895	1.00	0.00	H
ATOM	1936	CA	SER	B	202	-10.443	10.890	30.959	1.00	0.00	C
ATOM	1937	CB	SER	B	202	-10.791	9.542	31.609	1.00	0.00	C
ATOM	1938	OG	SER	B	202	-12.118	9.143	31.233	1.00	0.00	O
ATOM	1939	HG	SER	B	202	-12.365	8.441	31.840	1.00	0.00	H
ATOM	1940	C	SER	B	202	-11.378	11.986	31.442	1.00	0.00	C
ATOM	1941	O	SER	B	202	-10.986	12.925	32.133	1.00	0.00	O
ATOM	1942	N	LYS	B	203	-12.628	11.845	31.007	1.00	0.00	N
ATOM	1943	H	LYS	B	203	-12.835	11.060	30.422	1.00	0.00	H
ATOM	1944	CA	LYS	B	203	-13.666	12.699	31.573	1.00	0.00	C
ATOM	1945	CB	LYS	B	203	-14.222	13.638	30.495	1.00	0.00	C
ATOM	1946	CG	LYS	B	203	-13.138	14.581	29.952	1.00	0.00	C
ATOM	1947	CD	LYS	B	203	-12.647	15.585	31.003	1.00	0.00	C
ATOM	1948	CE	LYS	B	203	-11.248	16.152	30.730	1.00	0.00	C
ATOM	1949	NZ	LYS	B	203	-10.241	15.102	30.938	1.00	0.00	N
ATOM	1950	HZ1	LYS	B	203	-9.289	15.491	31.105	1.00	0.00	H
ATOM	1951	HZ2	LYS	B	203	-10.494	14.513	31.759	1.00	0.00	H
ATOM	1952	HZ3	LYS	B	203	-10.163	14.456	30.125	1.00	0.00	H
ATOM	1953	C	LYS	B	203	-14.750	11.891	32.268	1.00	0.00	C
ATOM	1954	O	LYS	B	203	-15.659	12.426	32.884	1.00	0.00	O
ATOM	1955	N	ARG	B	204	-14.607	10.559	32.142	1.00	0.00	N
ATOM	1956	H	ARG	B	204	-13.826	10.134	31.685	1.00	0.00	H
ATOM	1957	CA	ARG	B	204	-15.541	9.682	32.836	1.00	0.00	C
ATOM	1958	CB	ARG	B	204	-16.509	9.004	31.863	1.00	0.00	C
ATOM	1959	CG	ARG	B	204	-17.194	9.928	30.850	1.00	0.00	C
ATOM	1960	CD	ARG	B	204	-18.135	9.171	29.909	1.00	0.00	C
ATOM	1961	NE	ARG	B	204	-17.449	8.033	29.296	1.00	0.00	N
ATOM	1962	HE	ARG	B	204	-16.558	8.188	28.871	1.00	0.00	H
ATOM	1963	CZ	ARG	B	204	-17.968	6.792	29.410	1.00	0.00	C
ATOM	1964	NH1	ARG	B	204	-19.163	6.603	29.959	1.00	0.00	N
ATOM	1965	HH11	ARG	B	204	-19.736	7.350	30.285	1.00	0.00	H
ATOM	1966	HH12	ARG	B	204	-19.484	5.651	30.067	1.00	0.00	H
ATOM	1967	NH2	ARG	B	204	-17.277	5.744	28.982	1.00	0.00	N

ATOM	1968	HH21	ARG	B	204	-16.375	5.804	28.568	1.00	0.00	H
ATOM	1969	HH22	ARG	B	204	-17.681	4.818	29.092	1.00	0.00	H
ATOM	1970	C	ARG	B	204	-14.758	8.607	33.553	1.00	0.00	C
ATOM	1971	O	ARG	B	204	-13.803	8.054	33.017	1.00	0.00	O
ATOM	1972	N	THR	B	205	-15.201	8.322	34.776	1.00	0.00	N
ATOM	1973	H	THR	B	205	-15.937	8.839	35.215	1.00	0.00	H
ATOM	1974	CA	THR	B	205	-14.534	7.276	35.548	1.00	0.00	C
ATOM	1975	CB	THR	B	205	-14.430	7.776	36.984	1.00	0.00	C
ATOM	1976	OG1	THR	B	205	-15.556	8.621	37.282	1.00	0.00	O
ATOM	1977	HG1	THR	B	205	-16.249	8.061	37.631	1.00	0.00	H
ATOM	1978	CG2	THR	B	205	-13.128	8.547	37.213	1.00	0.00	C
ATOM	1979	C	THR	B	205	-15.225	5.921	35.469	1.00	0.00	C
ATOM	1980	O	THR	B	205	-15.255	5.138	36.413	1.00	0.00	O
ATOM	1981	N	SER	B	206	-15.822	5.703	34.295	1.00	0.00	N
ATOM	1982	H	SER	B	206	-15.664	6.283	33.497	1.00	0.00	H
ATOM	1983	CA	SER	B	206	-16.646	4.519	34.103	1.00	0.00	C
ATOM	1984	CB	SER	B	206	-17.746	4.967	33.139	1.00	0.00	C
ATOM	1985	OG	SER	B	206	-17.153	5.532	31.966	1.00	0.00	O
ATOM	1986	HG	SER	B	206	-17.308	4.853	31.300	1.00	0.00	H
ATOM	1987	C	SER	B	206	-15.788	3.346	33.625	1.00	0.00	C
ATOM	1988	O	SER	B	206	-14.653	3.556	33.202	1.00	0.00	O
ATOM	1989	N	PRO	B	207	-16.325	2.094	33.722	1.00	0.00	N
ATOM	1990	CD	PRO	B	207	-17.669	1.727	34.171	1.00	0.00	C
ATOM	1991	CA	PRO	B	207	-15.537	0.896	33.395	1.00	0.00	C
ATOM	1992	CB	PRO	B	207	-16.618	-0.157	33.143	1.00	0.00	C
ATOM	1993	CG	PRO	B	207	-17.690	0.201	34.168	1.00	0.00	C
ATOM	1994	C	PRO	B	207	-14.464	0.981	32.318	1.00	0.00	C
ATOM	1995	O	PRO	B	207	-13.306	0.680	32.580	1.00	0.00	O
ATOM	1996	N	ALA	B	208	-14.870	1.404	31.111	1.00	0.00	N
ATOM	1997	H	ALA	B	208	-15.795	1.765	30.975	1.00	0.00	H
ATOM	1998	CA	ALA	B	208	-13.873	1.439	30.041	1.00	0.00	C
ATOM	1999	CB	ALA	B	208	-14.492	1.463	28.655	1.00	0.00	C
ATOM	2000	C	ALA	B	208	-12.911	2.595	30.046	1.00	0.00	C
ATOM	2001	O	ALA	B	208	-11.868	2.534	29.406	1.00	0.00	O
ATOM	2002	N	ARG	B	209	-13.274	3.670	30.747	1.00	0.00	N
ATOM	2003	H	ARG	B	209	-14.129	3.715	31.270	1.00	0.00	H
ATOM	2004	CA	ARG	B	209	-12.222	4.677	30.834	1.00	0.00	C
ATOM	2005	CB	ARG	B	209	-12.698	6.036	30.319	1.00	0.00	C
ATOM	2006	CG	ARG	B	209	-12.842	5.996	28.788	1.00	0.00	C
ATOM	2007	CD	ARG	B	209	-11.539	5.569	28.086	1.00	0.00	C
ATOM	2008	NE	ARG	B	209	-11.709	5.377	26.641	1.00	0.00	N
ATOM	2009	HE	ARG	B	209	-11.849	6.205	26.097	1.00	0.00	H
ATOM	2010	CZ	ARG	B	209	-11.623	4.143	26.077	1.00	0.00	C
ATOM	2011	NH1	ARG	B	209	-11.418	3.064	26.827	1.00	0.00	N
ATOM	2012	HH11	ARG	B	209	-11.352	3.135	27.829	1.00	0.00	H
ATOM	2013	HH12	ARG	B	209	-11.329	2.152	26.426	1.00	0.00	H
ATOM	2014	NH2	ARG	B	209	-11.749	4.000	24.756	1.00	0.00	N
ATOM	2015	HH21	ARG	B	209	-11.887	4.795	24.166	1.00	0.00	H
ATOM	2016	HH22	ARG	B	209	-11.712	3.095	24.315	1.00	0.00	H
ATOM	2017	C	ARG	B	209	-11.474	4.701	32.154	1.00	0.00	C
ATOM	2018	O	ARG	B	209	-10.595	5.515	32.411	1.00	0.00	O
ATOM	2019	N	MET	B	210	-11.807	3.676	32.946	1.00	0.00	N
ATOM	2020	H	MET	B	210	-12.674	3.193	32.824	1.00	0.00	H
ATOM	2021	CA	MET	B	210	-10.776	3.164	33.838	1.00	0.00	C
ATOM	2022	CB	MET	B	210	-11.423	2.497	35.051	1.00	0.00	C
ATOM	2023	CG	MET	B	210	-12.345	3.447	35.816	1.00	0.00	C
ATOM	2024	SD	MET	B	210	-12.977	2.723	37.336	1.00	0.00	S
ATOM	2025	CE	MET	B	210	-11.413	2.676	38.219	1.00	0.00	C
ATOM	2026	C	MET	B	210	-9.849	2.212	33.094	1.00	0.00	C

```
ATOM   2027  O   MET B 210      -8.634   2.344  33.063  1.00  0.00           O
ATOM   2028  N   ALA B 211     -10.517   1.259  32.423  1.00  0.00           N
ATOM   2029  H   ALA B 211     -11.503   1.186  32.555  1.00  0.00           H
ATOM   2030  CA  ALA B 211      -9.800   0.234  31.666  1.00  0.00           C
ATOM   2031  CB  ALA B 211     -10.792  -0.660  30.914  1.00  0.00           C
ATOM   2032  C   ALA B 211      -8.763   0.764  30.686  1.00  0.00           C
ATOM   2033  O   ALA B 211      -7.582   0.454  30.732  1.00  0.00           O
ATOM   2034  N   GLY B 212      -9.258   1.620  29.790  1.00  0.00           N
ATOM   2035  H   GLY B 212     -10.207   1.900  29.890  1.00  0.00           H
ATOM   2036  CA  GLY B 212      -8.320   2.301  28.905  1.00  0.00           C
ATOM   2037  C   GLY B 212      -7.675   3.469  29.618  1.00  0.00           C
ATOM   2038  O   GLY B 212      -8.103   4.606  29.470  1.00  0.00           O
ATOM   2039  N   ASN B 213      -6.668   3.077  30.413  1.00  0.00           N
ATOM   2040  H   ASN B 213      -6.462   2.095  30.372  1.00  0.00           H
ATOM   2041  CA  ASN B 213      -5.856   3.890  31.331  1.00  0.00           C
ATOM   2042  CB  ASN B 213      -6.666   4.827  32.239  1.00  0.00           C
ATOM   2043  CG  ASN B 213      -6.833   6.193  31.598  1.00  0.00           C
ATOM   2044  OD1 ASN B 213      -5.909   6.824  31.106  1.00  0.00           O
ATOM   2045  ND2 ASN B 213      -8.079   6.665  31.645  1.00  0.00           N
ATOM   2046 HD21 ASN B 213      -8.234   7.581  31.291  1.00  0.00           H
ATOM   2047 HD22 ASN B 213      -8.837   6.105  31.980  1.00  0.00           H
ATOM   2048  C   ASN B 213      -5.074   2.933  32.213  1.00  0.00           C
ATOM   2049  O   ASN B 213      -3.895   3.091  32.509  1.00  0.00           O
ATOM   2050  N   GLY B 214      -5.784   1.854  32.582  1.00  0.00           N
ATOM   2051  H   GLY B 214      -6.763   1.792  32.377  1.00  0.00           H
ATOM   2052  CA  GLY B 214      -5.062   0.706  33.119  1.00  0.00           C
ATOM   2053  C   GLY B 214      -4.187   0.032  32.082  1.00  0.00           C
ATOM   2054  O   GLY B 214      -4.559  -0.109  30.925  1.00  0.00           O
ATOM   2055  N   GLY B 215      -2.995  -0.369  32.541  1.00  0.00           N
ATOM   2056  H   GLY B 215      -2.843  -0.386  33.531  1.00  0.00           H
ATOM   2057  CA  GLY B 215      -2.071  -1.015  31.610  1.00  0.00           C
ATOM   2058  C   GLY B 215      -2.555  -2.386  31.177  1.00  0.00           C
ATOM   2059  O   GLY B 215      -2.687  -2.695  29.998  1.00  0.00           O
ATOM   2060  N   ASP B 216      -2.864  -3.190  32.209  1.00  0.00           N
ATOM   2061  H   ASP B 216      -2.709  -2.863  33.143  1.00  0.00           H
ATOM   2062  CA  ASP B 216      -3.434  -4.524  31.991  1.00  0.00           C
ATOM   2063  CB  ASP B 216      -3.954  -5.131  33.302  1.00  0.00           C
ATOM   2064  CG  ASP B 216      -2.884  -5.479  34.326  1.00  0.00           C
ATOM   2065  OD1 ASP B 216      -1.696  -5.264  34.112  1.00  0.00           O
ATOM   2066  OD2 ASP B 216      -3.231  -6.003  35.377  1.00  0.00           O
ATOM   2067  C   ASP B 216      -4.608  -4.494  31.035  1.00  0.00           C
ATOM   2068  O   ASP B 216      -4.705  -5.217  30.050  1.00  0.00           O
ATOM   2069  N   ALA B 217      -5.502  -3.570  31.394  1.00  0.00           N
ATOM   2070  H   ALA B 217      -5.343  -3.034  32.221  1.00  0.00           H
ATOM   2071  CA  ALA B 217      -6.733  -3.433  30.638  1.00  0.00           C
ATOM   2072  CB  ALA B 217      -7.679  -2.539  31.410  1.00  0.00           C
ATOM   2073  C   ALA B 217      -6.565  -2.912  29.225  1.00  0.00           C
ATOM   2074  O   ALA B 217      -7.264  -3.324  28.307  1.00  0.00           O
ATOM   2075  N   ALA B 218      -5.575  -2.021  29.062  1.00  0.00           N
ATOM   2076  H   ALA B 218      -5.082  -1.645  29.848  1.00  0.00           H
ATOM   2077  CA  ALA B 218      -5.249  -1.604  27.700  1.00  0.00           C
ATOM   2078  CB  ALA B 218      -4.149  -0.540  27.693  1.00  0.00           C
ATOM   2079  C   ALA B 218      -4.818  -2.769  26.828  1.00  0.00           C
ATOM   2080  O   ALA B 218      -5.319  -2.967  25.728  1.00  0.00           O
ATOM   2081  N   LEU B 219      -3.904  -3.573  27.401  1.00  0.00           N
ATOM   2082  H   LEU B 219      -3.548  -3.334  28.308  1.00  0.00           H
ATOM   2083  CA  LEU B 219      -3.469  -4.787  26.700  1.00  0.00           C
ATOM   2084  CB  LEU B 219      -2.450  -5.563  27.536  1.00  0.00           C
ATOM   2085  CG  LEU B 219      -1.214  -4.753  27.933  1.00  0.00           C
```

ATOM	2086	CD1	LEU	B	219	-0.366	-5.501	28.961	1.00	0.00	C
ATOM	2087	CD2	LEU	B	219	-0.398	-4.302	26.720	1.00	0.00	C
ATOM	2088	C	LEU	B	219	-4.621	-5.701	26.317	1.00	0.00	C
ATOM	2089	O	LEU	B	219	-4.760	-6.149	25.184	1.00	0.00	O
ATOM	2090	N	ALA	B	220	-5.492	-5.892	27.322	1.00	0.00	N
ATOM	2091	H	ALA	B	220	-5.257	-5.532	28.226	1.00	0.00	H
ATOM	2092	CA	ALA	B	220	-6.736	-6.625	27.100	1.00	0.00	C
ATOM	2093	CB	ALA	B	220	-7.636	-6.519	28.331	1.00	0.00	C
ATOM	2094	C	ALA	B	220	-7.518	-6.152	25.889	1.00	0.00	C
ATOM	2095	O	ALA	B	220	-7.862	-6.913	24.995	1.00	0.00	O
ATOM	2096	N	LEU	B	221	-7.755	-4.835	25.882	1.00	0.00	N
ATOM	2097	H	LEU	B	221	-7.398	-4.257	26.618	1.00	0.00	H
ATOM	2098	CA	LEU	B	221	-8.498	-4.271	24.758	1.00	0.00	C
ATOM	2099	CB	LEU	B	221	-8.946	-2.848	25.090	1.00	0.00	C
ATOM	2100	CG	LEU	B	221	-9.873	-2.752	26.307	1.00	0.00	C
ATOM	2101	CD1	LEU	B	221	-10.106	-1.298	26.724	1.00	0.00	C
ATOM	2102	CD2	LEU	B	221	-11.188	-3.506	26.097	1.00	0.00	C
ATOM	2103	C	LEU	B	221	-7.770	-4.332	23.423	1.00	0.00	C
ATOM	2104	O	LEU	B	221	-8.373	-4.381	22.362	1.00	0.00	O
ATOM	2105	N	LEU	B	222	-6.432	-4.372	23.509	1.00	0.00	N
ATOM	2106	H	LEU	B	222	-5.982	-4.311	24.402	1.00	0.00	H
ATOM	2107	CA	LEU	B	222	-5.656	-4.598	22.288	1.00	0.00	C
ATOM	2108	CB	LEU	B	222	-4.198	-4.179	22.493	1.00	0.00	C
ATOM	2109	CG	LEU	B	222	-4.002	-2.711	22.874	1.00	0.00	C
ATOM	2110	CD1	LEU	B	222	-2.572	-2.435	23.337	1.00	0.00	C
ATOM	2111	CD2	LEU	B	222	-4.436	-1.764	21.759	1.00	0.00	C
ATOM	2112	C	LEU	B	222	-5.681	-6.026	21.754	1.00	0.00	C
ATOM	2113	O	LEU	B	222	-5.041	-6.331	20.753	1.00	0.00	O
ATOM	2114	N	LEU	B	223	-6.417	-6.885	22.489	1.00	0.00	N
ATOM	2115	H	LEU	B	223	-6.922	-6.546	23.281	1.00	0.00	H
ATOM	2116	CA	LEU	B	223	-6.444	-8.334	22.276	1.00	0.00	C
ATOM	2117	CB	LEU	B	223	-7.042	-8.747	20.922	1.00	0.00	C
ATOM	2118	CG	LEU	B	223	-8.543	-8.452	20.822	1.00	0.00	C
ATOM	2119	CD1	LEU	B	223	-8.843	-7.114	20.140	1.00	0.00	C
ATOM	2120	CD2	LEU	B	223	-9.301	-9.613	20.178	1.00	0.00	C
ATOM	2121	C	LEU	B	223	-5.138	-9.046	22.578	1.00	0.00	C
ATOM	2122	O	LEU	B	223	-4.900	-10.191	22.213	1.00	0.00	O
ATOM	2123	N	LEU	B	224	-4.301	-8.323	23.335	1.00	0.00	N
ATOM	2124	H	LEU	B	224	-4.568	-7.419	23.669	1.00	0.00	H
ATOM	2125	CA	LEU	B	224	-3.186	-9.019	23.963	1.00	0.00	C
ATOM	2126	CB	LEU	B	224	-2.023	-8.053	24.197	1.00	0.00	C
ATOM	2127	CG	LEU	B	224	-1.543	-7.364	22.916	1.00	0.00	C
ATOM	2128	CD1	LEU	B	224	-0.534	-6.257	23.217	1.00	0.00	C
ATOM	2129	CD2	LEU	B	224	-1.013	-8.359	21.882	1.00	0.00	C
ATOM	2130	C	LEU	B	224	-3.698	-9.596	25.264	1.00	0.00	C
ATOM	2131	O	LEU	B	224	-3.620	-8.974	26.316	1.00	0.00	O
ATOM	2132	N	ASP	B	225	-4.308	-10.784	25.100	1.00	0.00	N
ATOM	2133	H	ASP	B	225	-4.285	-11.177	24.180	1.00	0.00	H
ATOM	2134	CA	ASP	B	225	-5.263	-11.302	26.085	1.00	0.00	C
ATOM	2135	CB	ASP	B	225	-5.711	-12.729	25.737	1.00	0.00	C
ATOM	2136	CG	ASP	B	225	-7.136	-13.001	26.214	1.00	0.00	C
ATOM	2137	OD1	ASP	B	225	-7.852	-13.726	25.529	1.00	0.00	O
ATOM	2138	OD2	ASP	B	225	-7.538	-12.498	27.265	1.00	0.00	O
ATOM	2139	C	ASP	B	225	-4.947	-11.172	27.569	1.00	0.00	C
ATOM	2140	O	ASP	B	225	-4.414	-12.042	28.247	1.00	0.00	O
ATOM	2141	N	ARG	B	226	-5.387	-10.008	28.053	1.00	0.00	N
ATOM	2142	H	ARG	B	226	-5.551	-9.274	27.395	1.00	0.00	H
ATOM	2143	CA	ARG	B	226	-5.673	-9.887	29.474	1.00	0.00	C
ATOM	2144	CB	ARG	B	226	-5.006	-8.637	30.047	1.00	0.00	C

```
ATOM   2145  CG  ARG B 226      -3.485  -8.572  29.870  1.00  0.00           C
ATOM   2146  CD  ARG B 226      -2.705  -9.610  30.691  1.00  0.00           C
ATOM   2147  NE  ARG B 226      -3.072  -9.557  32.108  1.00  0.00           N
ATOM   2148  HE  ARG B 226      -3.738 -10.226  32.454  1.00  0.00           H
ATOM   2149  CZ  ARG B 226      -2.683  -8.564  32.936  1.00  0.00           C
ATOM   2150  NH1 ARG B 226      -1.785  -7.655  32.560  1.00  0.00           N
ATOM   2151 HH11 ARG B 226      -1.330  -7.716  31.674  1.00  0.00           H
ATOM   2152 HH12 ARG B 226      -1.567  -6.875  33.164  1.00  0.00           H
ATOM   2153  NH2 ARG B 226      -3.234  -8.498  34.139  1.00  0.00           N
ATOM   2154 HH21 ARG B 226      -3.864  -9.223  34.438  1.00  0.00           H
ATOM   2155 HH22 ARG B 226      -3.058  -7.717  34.754  1.00  0.00           H
ATOM   2156  C   ARG B 226      -7.173  -9.839  29.715  1.00  0.00           C
ATOM   2157  O   ARG B 226      -7.647  -9.618  30.825  1.00  0.00           O
ATOM   2158  N   LEU B 227      -7.921 -10.038  28.609  1.00  0.00           N
ATOM   2159  H   LEU B 227      -7.509 -10.472  27.805  1.00  0.00           H
ATOM   2160  CA  LEU B 227      -9.379  -9.950  28.674  1.00  0.00           C
ATOM   2161  CB  LEU B 227     -10.006 -10.207  27.304  1.00  0.00           C
ATOM   2162  CG  LEU B 227      -9.970  -9.013  26.355  1.00  0.00           C
ATOM   2163  CD1 LEU B 227     -10.232  -9.432  24.907  1.00  0.00           C
ATOM   2164  CD2 LEU B 227     -10.922  -7.903  26.808  1.00  0.00           C
ATOM   2165  C   LEU B 227      -9.960 -10.918  29.673  1.00  0.00           C
ATOM   2166  O   LEU B 227     -10.864 -10.603  30.429  1.00  0.00           O
ATOM   2167  N   ASN B 228      -9.352 -12.105  29.697  1.00  0.00           N
ATOM   2168  H   ASN B 228      -8.643 -12.311  29.014  1.00  0.00           H
ATOM   2169  CA  ASN B 228      -9.890 -13.108  30.619  1.00  0.00           C
ATOM   2170  CB  ASN B 228      -9.466 -14.513  30.191  1.00  0.00           C
ATOM   2171  CG  ASN B 228      -9.902 -14.710  28.758  1.00  0.00           C
ATOM   2172  OD1 ASN B 228     -11.051 -14.486  28.385  1.00  0.00           O
ATOM   2173  ND2 ASN B 228      -8.884 -14.986  27.947  1.00  0.00           N
ATOM   2174 HD21 ASN B 228      -8.913 -14.761  26.968  1.00  0.00           H
ATOM   2175 HD22 ASN B 228      -7.976 -15.274  28.243  1.00  0.00           H
ATOM   2176  C   ASN B 228      -9.634 -12.872  32.102  1.00  0.00           C
ATOM   2177  O   ASN B 228      -9.977 -13.687  32.947  1.00  0.00           O
ATOM   2178  N   GLN B 229      -9.014 -11.712  32.384  1.00  0.00           N
ATOM   2179  H   GLN B 229      -8.684 -11.130  31.643  1.00  0.00           H
ATOM   2180  CA  GLN B 229      -8.996 -11.217  33.757  1.00  0.00           C
ATOM   2181  CB  GLN B 229      -7.557 -10.944  34.204  1.00  0.00           C
ATOM   2182  CG  GLN B 229      -6.633 -12.152  34.038  1.00  0.00           C
ATOM   2183  CD  GLN B 229      -5.235 -11.795  34.494  1.00  0.00           C
ATOM   2184  OE1 GLN B 229      -4.662 -10.769  34.140  1.00  0.00           O
ATOM   2185  NE2 GLN B 229      -4.692 -12.699  35.311  1.00  0.00           N
ATOM   2186 HE21 GLN B 229      -3.767 -12.566  35.662  1.00  0.00           H
ATOM   2187 HE22 GLN B 229      -5.204 -13.516  35.574  1.00  0.00           H
ATOM   2188  C   GLN B 229      -9.838  -9.956  33.899  1.00  0.00           C
ATOM   2189  O   GLN B 229     -10.725  -9.837  34.733  1.00  0.00           O
ATOM   2190  N   LEU B 230      -9.506  -8.996  33.020  1.00  0.00           N
ATOM   2191  H   LEU B 230      -8.792  -9.181  32.343  1.00  0.00           H
ATOM   2192  CA  LEU B 230     -10.148  -7.681  33.070  1.00  0.00           C
ATOM   2193  CB  LEU B 230      -9.423  -6.780  32.052  1.00  0.00           C
ATOM   2194  CG  LEU B 230      -9.913  -5.344  31.789  1.00  0.00           C
ATOM   2195  CD1 LEU B 230     -10.956  -5.265  30.673  1.00  0.00           C
ATOM   2196  CD2 LEU B 230     -10.317  -4.587  33.055  1.00  0.00           C
ATOM   2197  C   LEU B 230     -11.660  -7.714  32.881  1.00  0.00           C
ATOM   2198  O   LEU B 230     -12.434  -7.069  33.585  1.00  0.00           O
ATOM   2199  N   GLU B 231     -12.048  -8.488  31.863  1.00  0.00           N
ATOM   2200  H   GLU B 231     -11.389  -9.102  31.431  1.00  0.00           H
ATOM   2201  CA  GLU B 231     -13.455  -8.541  31.492  1.00  0.00           C
ATOM   2202  CB  GLU B 231     -13.594  -9.133  30.091  1.00  0.00           C
ATOM   2203  CG  GLU B 231     -14.984  -8.965  29.488  1.00  0.00           C
```

ATOM	2204	CD	GLU	B	231	-15.110	-9.825	28.247	1.00	0.00	C
ATOM	2205	OE1	GLU	B	231	-14.873	-11.019	28.315	1.00	0.00	O
ATOM	2206	OE2	GLU	B	231	-15.502	-9.326	27.199	1.00	0.00	O
ATOM	2207	C	GLU	B	231	-14.294	-9.293	32.507	1.00	0.00	C
ATOM	2208	O	GLU	B	231	-14.502	-10.499	32.455	1.00	0.00	O
ATOM	2209	N	SER	B	232	-14.802	-8.496	33.450	1.00	0.00	N
ATOM	2210	H	SER	B	232	-14.572	-7.522	33.425	1.00	0.00	H
ATOM	2211	CA	SER	B	232	-15.408	-9.066	34.652	1.00	0.00	C
ATOM	2212	CB	SER	B	232	-15.314	-8.022	35.768	1.00	0.00	C
ATOM	2213	OG	SER	B	232	-14.362	-6.997	35.417	1.00	0.00	O
ATOM	2214	HG	SER	B	232	-13.549	-7.451	35.176	1.00	0.00	H
ATOM	2215	C	SER	B	232	-16.816	-9.658	34.529	1.00	0.00	C
ATOM	2216	O	SER	B	232	-17.614	-9.673	35.470	1.00	0.00	O
ATOM	2217	N	LYS	B	233	-17.081	-10.146	33.308	1.00	0.00	N
ATOM	2218	H	LYS	B	233	-16.380	-10.092	32.600	1.00	0.00	H
ATOM	2219	CA	LYS	B	233	-18.290	-10.902	33.021	1.00	0.00	C
ATOM	2220	CB	LYS	B	233	-19.155	-10.145	32.002	1.00	0.00	C
ATOM	2221	CG	LYS	B	233	-18.596	-10.059	30.572	1.00	0.00	C
ATOM	2222	CD	LYS	B	233	-19.546	-9.315	29.627	1.00	0.00	C
ATOM	2223	CE	LYS	B	233	-19.195	-9.420	28.136	1.00	0.00	C
ATOM	2224	NZ	LYS	B	233	-17.897	-8.802	27.850	1.00	0.00	N
ATOM	2225	HZ1	LYS	B	233	-17.097	-9.403	28.141	1.00	0.00	H
ATOM	2226	HZ2	LYS	B	233	-17.740	-8.650	26.836	1.00	0.00	H
ATOM	2227	HZ3	LYS	B	233	-17.796	-7.877	28.313	1.00	0.00	H
ATOM	2228	C	LYS	B	233	-18.002	-12.318	32.534	1.00	0.00	C
ATOM	2229	O	LYS	B	233	-18.899	-13.137	32.397	1.00	0.00	O
ATOM	2230	N	MET	B	234	-16.717	-12.556	32.228	1.00	0.00	N
ATOM	2231	H	MET	B	234	-15.953	-11.966	32.491	1.00	0.00	H
ATOM	2232	CA	MET	B	234	-16.424	-13.794	31.516	1.00	0.00	C
ATOM	2233	CB	MET	B	234	-15.517	-13.468	30.324	1.00	0.00	C
ATOM	2234	CG	MET	B	234	-15.349	-14.569	29.271	1.00	0.00	C
ATOM	2235	SD	MET	B	234	-16.904	-15.038	28.491	1.00	0.00	S
ATOM	2236	CE	MET	B	234	-17.249	-13.482	27.651	1.00	0.00	C
ATOM	2237	C	MET	B	234	-15.792	-14.834	32.416	1.00	0.00	C
ATOM	2238	O	MET	B	234	-15.054	-14.522	33.342	1.00	0.00	O
ATOM	2239	N	SER	B	235	-16.077	-16.101	32.088	1.00	0.00	N
ATOM	2240	H	SER	B	235	-16.677	-16.297	31.314	1.00	0.00	H
ATOM	2241	CA	SER	B	235	-15.181	-17.100	32.656	1.00	0.00	C
ATOM	2242	CB	SER	B	235	-15.783	-18.510	32.585	1.00	0.00	C
ATOM	2243	OG	SER	B	235	-14.873	-19.478	33.136	1.00	0.00	O
ATOM	2244	HG	SER	B	235	-15.164	-20.327	32.799	1.00	0.00	H
ATOM	2245	C	SER	B	235	-13.855	-17.076	31.927	1.00	0.00	C
ATOM	2246	O	SER	B	235	-13.763	-17.404	30.751	1.00	0.00	O
ATOM	2247	N	GLY	B	236	-12.812	-16.739	32.698	1.00	0.00	N
ATOM	2248	H	GLY	B	236	-12.992	-16.282	33.570	1.00	0.00	H
ATOM	2249	CA	GLY	B	236	-11.460	-16.914	32.163	1.00	0.00	C
ATOM	2250	C	GLY	B	236	-11.150	-18.324	31.666	1.00	0.00	C
ATOM	2251	O	GLY	B	236	-10.330	-18.553	30.783	1.00	0.00	O
ATOM	2252	N	LYS	B	237	-11.872	-19.279	32.278	1.00	0.00	N
ATOM	2253	H	LYS	B	237	-12.617	-19.029	32.897	1.00	0.00	H
ATOM	2254	CA	LYS	B	237	-11.678	-20.672	31.886	1.00	0.00	C
ATOM	2255	CB	LYS	B	237	-12.177	-21.598	32.996	1.00	0.00	C
ATOM	2256	CG	LYS	B	237	-11.627	-21.233	34.377	1.00	0.00	C
ATOM	2257	CD	LYS	B	237	-12.282	-22.035	35.502	1.00	0.00	C
ATOM	2258	CE	LYS	B	237	-11.779	-21.605	36.882	1.00	0.00	C
ATOM	2259	NZ	LYS	B	237	-12.490	-22.359	37.924	1.00	0.00	N
ATOM	2260	HZ1	LYS	B	237	-12.316	-23.377	37.800	1.00	0.00	H
ATOM	2261	HZ2	LYS	B	237	-12.148	-22.061	38.860	1.00	0.00	H
ATOM	2262	HZ3	LYS	B	237	-13.510	-22.169	37.853	1.00	0.00	H

```
ATOM   2263  C   LYS B 237     -12.297 -21.054  30.547  1.00  0.00           C
ATOM   2264  O   LYS B 237     -12.066 -22.141  30.029  1.00  0.00           O
ATOM   2265  N   GLY B 238     -13.072 -20.107  29.982  1.00  0.00           N
ATOM   2266  H   GLY B 238     -13.243 -19.245  30.458  1.00  0.00           H
ATOM   2267  CA  GLY B 238     -13.716 -20.319  28.682  1.00  0.00           C
ATOM   2268  C   GLY B 238     -12.785 -20.794  27.578  1.00  0.00           C
ATOM   2269  O   GLY B 238     -13.120 -21.638  26.753  1.00  0.00           O
ATOM   2270  N   GLN B 239     -11.551 -20.264  27.657  1.00  0.00           N
ATOM   2271  H   GLN B 239     -11.417 -19.529  28.322  1.00  0.00           H
ATOM   2272  CA  GLN B 239     -10.469 -20.721  26.774  1.00  0.00           C
ATOM   2273  CB  GLN B 239      -9.127 -20.192  27.280  1.00  0.00           C
ATOM   2274  CG  GLN B 239      -9.106 -18.673  27.441  1.00  0.00           C
ATOM   2275  CD  GLN B 239      -7.783 -18.244  28.034  1.00  0.00           C
ATOM   2276  OE1 GLN B 239      -6.767 -18.140  27.366  1.00  0.00           O
ATOM   2277  NE2 GLN B 239      -7.836 -17.998  29.344  1.00  0.00           N
ATOM   2278 HE21 GLN B 239      -6.995 -17.711  29.798  1.00  0.00           H
ATOM   2279 HE22 GLN B 239      -8.689 -18.107  29.858  1.00  0.00           H
ATOM   2280  C   GLN B 239     -10.381 -22.234  26.593  1.00  0.00           C
ATOM   2281  O   GLN B 239     -10.159 -22.759  25.510  1.00  0.00           O
ATOM   2282  N   GLN B 240     -10.589 -22.918  27.727  1.00  0.00           N
ATOM   2283  H   GLN B 240     -10.810 -22.428  28.568  1.00  0.00           H
ATOM   2284  CA  GLN B 240     -10.688 -24.369  27.638  1.00  0.00           C
ATOM   2285  CB  GLN B 240      -9.986 -25.016  28.834  1.00  0.00           C
ATOM   2286  CG  GLN B 240      -8.496 -24.658  28.916  1.00  0.00           C
ATOM   2287  CD  GLN B 240      -7.772 -25.185  27.692  1.00  0.00           C
ATOM   2288  OE1 GLN B 240      -7.942 -26.323  27.279  1.00  0.00           O
ATOM   2289  NE2 GLN B 240      -6.960 -24.299  27.110  1.00  0.00           N
ATOM   2290 HE21 GLN B 240      -6.471 -24.582  26.287  1.00  0.00           H
ATOM   2291 HE22 GLN B 240      -6.831 -23.376  27.466  1.00  0.00           H
ATOM   2292  C   GLN B 240     -12.113 -24.872  27.487  1.00  0.00           C
ATOM   2293  O   GLN B 240     -12.419 -25.663  26.604  1.00  0.00           O
ATOM   2294  N   GLN B 241     -12.982 -24.364  28.383  1.00  0.00           N
ATOM   2295  H   GLN B 241     -12.637 -23.695  29.041  1.00  0.00           H
ATOM   2296  CA  GLN B 241     -14.375 -24.832  28.426  1.00  0.00           C
ATOM   2297  CB  GLN B 241     -15.195 -24.009  29.423  1.00  0.00           C
ATOM   2298  CG  GLN B 241     -14.701 -24.061  30.869  1.00  0.00           C
ATOM   2299  CD  GLN B 241     -15.575 -23.155  31.715  1.00  0.00           C
ATOM   2300  OE1 GLN B 241     -15.244 -22.016  32.024  1.00  0.00           O
ATOM   2301  NE2 GLN B 241     -16.729 -23.716  32.086  1.00  0.00           N
ATOM   2302 HE21 GLN B 241     -17.375 -23.184  32.630  1.00  0.00           H
ATOM   2303 HE22 GLN B 241     -16.961 -24.651  31.823  1.00  0.00           H
ATOM   2304  C   GLN B 241     -15.092 -24.821  27.084  1.00  0.00           C
ATOM   2305  O   GLN B 241     -15.645 -25.806  26.611  1.00  0.00           O
ATOM   2306  N   GLN B 242     -15.044 -23.628  26.487  1.00  0.00           N
ATOM   2307  H   GLN B 242     -14.520 -22.893  26.914  1.00  0.00           H
ATOM   2308  CA  GLN B 242     -15.558 -23.488  25.131  1.00  0.00           C
ATOM   2309  CB  GLN B 242     -15.870 -22.019  24.854  1.00  0.00           C
ATOM   2310  CG  GLN B 242     -16.834 -21.415  25.876  1.00  0.00           C
ATOM   2311  CD  GLN B 242     -16.698 -19.909  25.839  1.00  0.00           C
ATOM   2312  OE1 GLN B 242     -15.691 -19.344  26.239  1.00  0.00           O
ATOM   2313  NE2 GLN B 242     -17.761 -19.272  25.344  1.00  0.00           N
ATOM   2314 HE21 GLN B 242     -17.718 -18.276  25.302  1.00  0.00           H
ATOM   2315 HE22 GLN B 242     -18.572 -19.758  25.026  1.00  0.00           H
ATOM   2316  C   GLN B 242     -14.552 -24.012  24.127  1.00  0.00           C
ATOM   2317  O   GLN B 242     -14.853 -24.832  23.267  1.00  0.00           O
ATOM   2318  N   GLY B 243     -13.319 -23.501  24.306  1.00  0.00           N
ATOM   2319  H   GLY B 243     -13.175 -22.849  25.054  1.00  0.00           H
ATOM   2320  CA  GLY B 243     -12.232 -23.778  23.364  1.00  0.00           C
ATOM   2321  C   GLY B 243     -12.117 -25.215  22.896  1.00  0.00           C
```

```
ATOM   2322  O   GLY B 243     -12.326 -25.530  21.729  1.00  0.00           O
ATOM   2323  N   GLN B 244     -11.812 -26.093  23.864  1.00  0.00           N
ATOM   2324  H   GLN B 244     -11.701 -25.761  24.803  1.00  0.00           H
ATOM   2325  CA  GLN B 244     -11.727 -27.516  23.524  1.00  0.00           C
ATOM   2326  CB  GLN B 244     -11.424 -28.379  24.747  1.00  0.00           C
ATOM   2327  CG  GLN B 244     -10.042 -28.195  25.371  1.00  0.00           C
ATOM   2328  CD  GLN B 244      -9.838 -29.300  26.391  1.00  0.00           C
ATOM   2329  OE1 GLN B 244     -10.383 -30.397  26.276  1.00  0.00           O
ATOM   2330  NE2 GLN B 244      -9.031 -28.975  27.398  1.00  0.00           N
ATOM   2331 HE21 GLN B 244      -8.834 -29.602  28.148  1.00  0.00           H
ATOM   2332 HE22 GLN B 244      -8.597 -28.070  27.406  1.00  0.00           H
ATOM   2333  C   GLN B 244     -12.982 -28.069  22.871  1.00  0.00           C
ATOM   2334  O   GLN B 244     -12.948 -28.830  21.912  1.00  0.00           O
ATOM   2335  N   THR B 245     -14.105 -27.638  23.439  1.00  0.00           N
ATOM   2336  H   THR B 245     -14.085 -27.009  24.218  1.00  0.00           H
ATOM   2337  CA  THR B 245     -15.390 -28.135  22.966  1.00  0.00           C
ATOM   2338  CB  THR B 245     -16.414 -27.781  24.046  1.00  0.00           C
ATOM   2339  OG1 THR B 245     -15.869 -28.181  25.308  1.00  0.00           O
ATOM   2340  HG1 THR B 245     -16.009 -27.450  25.909  1.00  0.00           H
ATOM   2341  CG2 THR B 245     -17.796 -28.412  23.847  1.00  0.00           C
ATOM   2342  C   THR B 245     -15.795 -27.706  21.554  1.00  0.00           C
ATOM   2343  O   THR B 245     -16.644 -28.326  20.911  1.00  0.00           O
ATOM   2344  N   VAL B 246     -15.137 -26.642  21.070  1.00  0.00           N
ATOM   2345  H   VAL B 246     -14.525 -26.110  21.662  1.00  0.00           H
ATOM   2346  CA  VAL B 246     -15.278 -26.357  19.642  1.00  0.00           C
ATOM   2347  CB  VAL B 246     -15.267 -24.845  19.371  1.00  0.00           C
ATOM   2348  CG1 VAL B 246     -15.630 -24.539  17.914  1.00  0.00           C
ATOM   2349  CG2 VAL B 246     -16.202 -24.100  20.327  1.00  0.00           C
ATOM   2350  C   VAL B 246     -14.220 -27.082  18.819  1.00  0.00           C
ATOM   2351  O   VAL B 246     -14.502 -27.781  17.851  1.00  0.00           O
ATOM   2352  N   THR B 247     -12.977 -26.926  19.295  1.00  0.00           N
ATOM   2353  H   THR B 247     -12.849 -26.305  20.066  1.00  0.00           H
ATOM   2354  CA  THR B 247     -11.814 -27.581  18.688  1.00  0.00           C
ATOM   2355  CB  THR B 247     -10.636 -27.434  19.662  1.00  0.00           C
ATOM   2356  OG1 THR B 247     -10.358 -26.043  19.872  1.00  0.00           O
ATOM   2357  HG1 THR B 247     -11.040 -25.709  20.442  1.00  0.00           H
ATOM   2358  CG2 THR B 247      -9.374 -28.181  19.241  1.00  0.00           C
ATOM   2359  C   THR B 247     -12.038 -29.031  18.262  1.00  0.00           C
ATOM   2360  O   THR B 247     -11.861 -29.410  17.112  1.00  0.00           O
ATOM   2361  N   LYS B 248     -12.497 -29.823  19.243  1.00  0.00           N
ATOM   2362  H   LYS B 248     -12.644 -29.431  20.150  1.00  0.00           H
ATOM   2363  CA  LYS B 248     -12.722 -31.245  18.979  1.00  0.00           C
ATOM   2364  CB  LYS B 248     -13.112 -31.947  20.281  1.00  0.00           C
ATOM   2365  CG  LYS B 248     -11.953 -31.916  21.280  1.00  0.00           C
ATOM   2366  CD  LYS B 248     -12.349 -32.276  22.712  1.00  0.00           C
ATOM   2367  CE  LYS B 248     -11.144 -32.171  23.648  1.00  0.00           C
ATOM   2368  NZ  LYS B 248     -11.572 -32.247  25.051  1.00  0.00           N
ATOM   2369  HZ1 LYS B 248     -11.841 -33.212  25.321  1.00  0.00           H
ATOM   2370  HZ2 LYS B 248     -10.788 -31.914  25.657  1.00  0.00           H
ATOM   2371  HZ3 LYS B 248     -12.356 -31.589  25.219  1.00  0.00           H
ATOM   2372  C   LYS B 248     -13.710 -31.546  17.863  1.00  0.00           C
ATOM   2373  O   LYS B 248     -13.557 -32.499  17.111  1.00  0.00           O
ATOM   2374  N   LYS B 249     -14.727 -30.670  17.767  1.00  0.00           N
ATOM   2375  H   LYS B 249     -14.707 -29.822  18.294  1.00  0.00           H
ATOM   2376  CA  LYS B 249     -15.649 -30.851  16.645  1.00  0.00           C
ATOM   2377  CB  LYS B 249     -16.874 -29.945  16.756  1.00  0.00           C
ATOM   2378  CG  LYS B 249     -17.671 -30.136  18.043  1.00  0.00           C
ATOM   2379  CD  LYS B 249     -18.970 -29.329  18.013  1.00  0.00           C
ATOM   2380  CE  LYS B 249     -19.782 -29.460  19.302  1.00  0.00           C
```

ATOM	2381	NZ	LYS	B	249	-19.077	-28.793	20.403	1.00	0.00	N
ATOM	2382	HZ1	LYS	B	249	-18.077	-29.091	20.430	1.00	0.00	H
ATOM	2383	HZ2	LYS	B	249	-19.080	-27.767	20.251	1.00	0.00	H
ATOM	2384	HZ3	LYS	B	249	-19.524	-29.018	21.312	1.00	0.00	H
ATOM	2385	C	LYS	B	249	-14.964	-30.613	15.317	1.00	0.00	C
ATOM	2386	O	LYS	B	249	-14.980	-31.441	14.422	1.00	0.00	O
ATOM	2387	N	SER	B	250	-14.294	-29.457	15.261	1.00	0.00	N
ATOM	2388	H	SER	B	250	-14.378	-28.791	16.003	1.00	0.00	H
ATOM	2389	CA	SER	B	250	-13.514	-29.117	14.070	1.00	0.00	C
ATOM	2390	CB	SER	B	250	-12.789	-27.798	14.340	1.00	0.00	C
ATOM	2391	OG	SER	B	250	-13.618	-26.964	15.167	1.00	0.00	O
ATOM	2392	HG	SER	B	250	-13.356	-27.133	16.065	1.00	0.00	H
ATOM	2393	C	SER	B	250	-12.553	-30.209	13.599	1.00	0.00	C
ATOM	2394	O	SER	B	250	-12.426	-30.529	12.422	1.00	0.00	O
ATOM	2395	N	ALA	B	251	-11.906	-30.810	14.610	1.00	0.00	N
ATOM	2396	H	ALA	B	251	-12.042	-30.472	15.542	1.00	0.00	H
ATOM	2397	CA	ALA	B	251	-11.042	-31.957	14.348	1.00	0.00	C
ATOM	2398	CB	ALA	B	251	-10.338	-32.387	15.636	1.00	0.00	C
ATOM	2399	C	ALA	B	251	-11.767	-33.153	13.750	1.00	0.00	C
ATOM	2400	O	ALA	B	251	-11.307	-33.781	12.801	1.00	0.00	O
ATOM	2401	N	ALA	B	252	-12.943	-33.432	14.336	1.00	0.00	N
ATOM	2402	H	ALA	B	252	-13.283	-32.871	15.094	1.00	0.00	H
ATOM	2403	CA	ALA	B	252	-13.757	-34.528	13.810	1.00	0.00	C
ATOM	2404	CB	ALA	B	252	-14.991	-34.759	14.685	1.00	0.00	C
ATOM	2405	C	ALA	B	252	-14.198	-34.318	12.373	1.00	0.00	C
ATOM	2406	O	ALA	B	252	-14.085	-35.198	11.529	1.00	0.00	O
ATOM	2407	N	GLU	B	253	-14.654	-33.085	12.107	1.00	0.00	N
ATOM	2408	H	GLU	B	253	-14.797	-32.442	12.858	1.00	0.00	H
ATOM	2409	CA	GLU	B	253	-15.035	-32.743	10.736	1.00	0.00	C
ATOM	2410	CB	GLU	B	253	-15.575	-31.310	10.651	1.00	0.00	C
ATOM	2411	CG	GLU	B	253	-16.719	-30.987	11.624	1.00	0.00	C
ATOM	2412	CD	GLU	B	253	-17.875	-31.954	11.448	1.00	0.00	C
ATOM	2413	OE1	GLU	B	253	-18.522	-31.945	10.402	1.00	0.00	O
ATOM	2414	OE2	GLU	B	253	-18.134	-32.744	12.354	1.00	0.00	O
ATOM	2415	C	GLU	B	253	-13.909	-32.949	9.738	1.00	0.00	C
ATOM	2416	O	GLU	B	253	-14.056	-33.580	8.698	1.00	0.00	O
ATOM	2417	N	ALA	B	254	-12.737	-32.438	10.147	1.00	0.00	N
ATOM	2418	H	ALA	B	254	-12.706	-31.899	10.992	1.00	0.00	H
ATOM	2419	CA	ALA	B	254	-11.546	-32.649	9.325	1.00	0.00	C
ATOM	2420	CB	ALA	B	254	-10.334	-31.981	9.969	1.00	0.00	C
ATOM	2421	C	ALA	B	254	-11.207	-34.111	9.068	1.00	0.00	C
ATOM	2422	O	ALA	B	254	-10.799	-34.514	7.986	1.00	0.00	O
ATOM	2423	N	SER	B	255	-11.390	-34.909	10.126	1.00	0.00	N
ATOM	2424	H	SER	B	255	-11.801	-34.572	10.975	1.00	0.00	H
ATOM	2425	CA	SER	B	255	-11.053	-36.312	9.927	1.00	0.00	C
ATOM	2426	CB	SER	B	255	-10.499	-36.909	11.224	1.00	0.00	C
ATOM	2427	OG	SER	B	255	-11.297	-36.514	12.343	1.00	0.00	O
ATOM	2428	HG	SER	B	255	-11.117	-35.591	12.503	1.00	0.00	H
ATOM	2429	C	SER	B	255	-12.144	-37.169	9.300	1.00	0.00	C
ATOM	2430	O	SER	B	255	-11.891	-38.271	8.831	1.00	0.00	O
ATOM	2431	N	LYS	B	256	-13.368	-36.616	9.266	1.00	0.00	N
ATOM	2432	H	LYS	B	256	-13.496	-35.728	9.713	1.00	0.00	H
ATOM	2433	CA	LYS	B	256	-14.551	-37.353	8.807	1.00	0.00	C
ATOM	2434	CB	LYS	B	256	-15.691	-36.366	8.575	1.00	0.00	C
ATOM	2435	CG	LYS	B	256	-16.888	-36.594	9.495	1.00	0.00	C
ATOM	2436	CD	LYS	B	256	-17.768	-35.347	9.558	1.00	0.00	C
ATOM	2437	CE	LYS	B	256	-19.028	-35.530	10.403	1.00	0.00	C
ATOM	2438	NZ	LYS	B	256	-19.587	-34.211	10.708	1.00	0.00	N
ATOM	2439	HZ1	LYS	B	256	-20.613	-34.174	10.810	1.00	0.00	H

```
ATOM   2440  HZ2 LYS B 256     -19.151 -33.845  11.581  1.00  0.00           H
ATOM   2441  HZ3 LYS B 256     -19.268 -33.486  10.027  1.00  0.00           H
ATOM   2442  C   LYS B 256     -14.384 -38.273   7.606  1.00  0.00           C
ATOM   2443  O   LYS B 256     -14.857 -39.409   7.579  1.00  0.00           O
ATOM   2444  N   LYS B 257     -13.670 -37.732   6.609  1.00  0.00           N
ATOM   2445  H   LYS B 257     -13.305 -36.807   6.716  1.00  0.00           H
ATOM   2446  CA  LYS B 257     -13.295 -38.640   5.531  1.00  0.00           C
ATOM   2447  CB  LYS B 257     -13.582 -38.028   4.147  1.00  0.00           C
ATOM   2448  CG  LYS B 257     -13.543 -39.058   3.013  1.00  0.00           C
ATOM   2449  CD  LYS B 257     -13.831 -38.447   1.640  1.00  0.00           C
ATOM   2450  CE  LYS B 257     -13.794 -39.491   0.520  1.00  0.00           C
ATOM   2451  NZ  LYS B 257     -14.089 -38.845  -0.767  1.00  0.00           N
ATOM   2452  HZ1 LYS B 257     -14.062 -39.557  -1.526  1.00  0.00           H
ATOM   2453  HZ2 LYS B 257     -15.035 -38.414  -0.732  1.00  0.00           H
ATOM   2454  HZ3 LYS B 257     -13.380 -38.109  -0.958  1.00  0.00           H
ATOM   2455  C   LYS B 257     -11.924 -39.316   5.654  1.00  0.00           C
ATOM   2456  O   LYS B 257     -11.878 -40.536   5.735  1.00  0.00           O
ATOM   2457  N   PRO B 258     -10.799 -38.541   5.656  1.00  0.00           N
ATOM   2458  CD  PRO B 258     -10.659 -37.083   5.687  1.00  0.00           C
ATOM   2459  CA  PRO B 258      -9.495 -39.216   5.542  1.00  0.00           C
ATOM   2460  CB  PRO B 258      -8.584 -38.081   5.069  1.00  0.00           C
ATOM   2461  CG  PRO B 258      -9.159 -36.840   5.742  1.00  0.00           C
ATOM   2462  C   PRO B 258      -8.971 -39.908   6.799  1.00  0.00           C
ATOM   2463  O   PRO B 258      -7.954 -40.589   6.759  1.00  0.00           O
ATOM   2464  N   ARG B 259      -9.689 -39.683   7.915  1.00  0.00           N
ATOM   2465  H   ARG B 259     -10.500 -39.104   7.847  1.00  0.00           H
ATOM   2466  CA  ARG B 259      -9.455 -40.385   9.183  1.00  0.00           C
ATOM   2467  CB  ARG B 259     -10.099 -41.771   9.126  1.00  0.00           C
ATOM   2468  CG  ARG B 259     -11.593 -41.507   8.983  1.00  0.00           C
ATOM   2469  CD  ARG B 259     -12.459 -42.630   8.436  1.00  0.00           C
ATOM   2470  NE  ARG B 259     -13.746 -42.029   8.101  1.00  0.00           N
ATOM   2471  HE  ARG B 259     -13.714 -41.219   7.506  1.00  0.00           H
ATOM   2472  CZ  ARG B 259     -14.902 -42.429   8.656  1.00  0.00           C
ATOM   2473  NH1 ARG B 259     -14.948 -43.526   9.410  1.00  0.00           N
ATOM   2474 HH11 ARG B 259     -14.112 -44.057   9.554  1.00  0.00           H
ATOM   2475 HH12 ARG B 259     -15.792 -43.843   9.843  1.00  0.00           H
ATOM   2476  NH2 ARG B 259     -15.989 -41.696   8.438  1.00  0.00           N
ATOM   2477 HH21 ARG B 259     -15.872 -40.832   7.929  1.00  0.00           H
ATOM   2478 HH22 ARG B 259     -16.899 -41.948   8.760  1.00  0.00           H
ATOM   2479  C   ARG B 259      -8.044 -40.386   9.730  1.00  0.00           C
ATOM   2480  O   ARG B 259      -7.581 -41.301  10.398  1.00  0.00           O
ATOM   2481  N   GLN B 260      -7.376 -39.268   9.434  1.00  0.00           N
ATOM   2482  H   GLN B 260      -7.795 -38.535   8.899  1.00  0.00           H
ATOM   2483  CA  GLN B 260      -6.033 -39.128   9.966  1.00  0.00           C
ATOM   2484  CB  GLN B 260      -5.026 -39.105   8.821  1.00  0.00           C
ATOM   2485  CG  GLN B 260      -4.926 -40.463   8.131  1.00  0.00           C
ATOM   2486  CD  GLN B 260      -4.288 -40.283   6.776  1.00  0.00           C
ATOM   2487  OE1 GLN B 260      -3.097 -40.020   6.641  1.00  0.00           O
ATOM   2488  NE2 GLN B 260      -5.148 -40.437   5.769  1.00  0.00           N
ATOM   2489 HE21 GLN B 260      -4.858 -40.347   4.819  1.00  0.00           H
ATOM   2490 HE22 GLN B 260      -6.108 -40.649   5.979  1.00  0.00           H
ATOM   2491  C   GLN B 260      -5.934 -37.867  10.781  1.00  0.00           C
ATOM   2492  O   GLN B 260      -6.436 -36.816  10.403  1.00  0.00           O
ATOM   2493  N   LYS B 261      -5.221 -38.000  11.910  1.00  0.00           N
ATOM   2494  H   LYS B 261      -4.905 -38.907  12.183  1.00  0.00           H
ATOM   2495  CA  LYS B 261      -4.983 -36.791  12.705  1.00  0.00           C
ATOM   2496  CB  LYS B 261      -4.293 -37.128  14.027  1.00  0.00           C
ATOM   2497  CG  LYS B 261      -5.189 -37.973  14.937  1.00  0.00           C
ATOM   2498  CD  LYS B 261      -4.572 -38.231  16.314  1.00  0.00           C
```

```
ATOM   2499  CE  LYS B 261      -5.507 -39.029  17.227  1.00  0.00           C
ATOM   2500  NZ  LYS B 261      -4.883 -39.200  18.547  1.00  0.00           N
ATOM   2501  HZ1 LYS B 261      -5.522 -39.741  19.164  1.00  0.00           H
ATOM   2502  HZ2 LYS B 261      -3.984 -39.713  18.446  1.00  0.00           H
ATOM   2503  HZ3 LYS B 261      -4.704 -38.266  18.969  1.00  0.00           H
ATOM   2504  C   LYS B 261      -4.244 -35.689  11.958  1.00  0.00           C
ATOM   2505  O   LYS B 261      -4.418 -34.509  12.212  1.00  0.00           O
ATOM   2506  N   ARG B 262      -3.462 -36.146  10.960  1.00  0.00           N
ATOM   2507  H   ARG B 262      -3.320 -37.129  10.894  1.00  0.00           H
ATOM   2508  CA  ARG B 262      -2.849 -35.231   9.989  1.00  0.00           C
ATOM   2509  CB  ARG B 262      -2.297 -36.026   8.803  1.00  0.00           C
ATOM   2510  CG  ARG B 262      -1.206 -37.038   9.163  1.00  0.00           C
ATOM   2511  CD  ARG B 262      -0.778 -37.865   7.948  1.00  0.00           C
ATOM   2512  NE  ARG B 262       0.360 -38.728   8.268  1.00  0.00           N
ATOM   2513  HE  ARG B 262       1.091 -38.316   8.815  1.00  0.00           H
ATOM   2514  CZ  ARG B 262       0.455 -39.977   7.758  1.00  0.00           C
ATOM   2515  NH1 ARG B 262      -0.530 -40.497   7.029  1.00  0.00           N
ATOM   2516 HH11 ARG B 262      -1.388 -39.988   6.871  1.00  0.00           H
ATOM   2517 HH12 ARG B 262      -0.479 -41.404   6.614  1.00  0.00           H
ATOM   2518  NH2 ARG B 262       1.553 -40.694   7.997  1.00  0.00           N
ATOM   2519 HH21 ARG B 262       2.302 -40.314   8.541  1.00  0.00           H
ATOM   2520 HH22 ARG B 262       1.658 -41.623   7.641  1.00  0.00           H
ATOM   2521  C   ARG B 262      -3.768 -34.134   9.461  1.00  0.00           C
ATOM   2522  O   ARG B 262      -3.398 -32.976   9.337  1.00  0.00           O
ATOM   2523  N   THR B 263      -4.999 -34.560   9.150  1.00  0.00           N
ATOM   2524  H   THR B 263      -5.313 -35.502   9.275  1.00  0.00           H
ATOM   2525  CA  THR B 263      -5.941 -33.586   8.613  1.00  0.00           C
ATOM   2526  CB  THR B 263      -7.062 -34.349   7.913  1.00  0.00           C
ATOM   2527  OG1 THR B 263      -6.512 -35.511   7.268  1.00  0.00           O
ATOM   2528  HG1 THR B 263      -6.033 -35.187   6.515  1.00  0.00           H
ATOM   2529  CG2 THR B 263      -7.812 -33.471   6.907  1.00  0.00           C
ATOM   2530  C   THR B 263      -6.477 -32.591   9.638  1.00  0.00           C
ATOM   2531  O   THR B 263      -6.815 -31.452   9.332  1.00  0.00           O
ATOM   2532  N   ALA B 264      -6.515 -33.071  10.891  1.00  0.00           N
ATOM   2533  H   ALA B 264      -6.086 -33.949  11.100  1.00  0.00           H
ATOM   2534  CA  ALA B 264      -6.942 -32.208  11.990  1.00  0.00           C
ATOM   2535  CB  ALA B 264      -7.462 -33.058  13.149  1.00  0.00           C
ATOM   2536  C   ALA B 264      -5.825 -31.298  12.477  1.00  0.00           C
ATOM   2537  O   ALA B 264      -5.344 -31.366  13.601  1.00  0.00           O
ATOM   2538  N   THR B 265      -5.428 -30.430  11.542  1.00  0.00           N
ATOM   2539  H   THR B 265      -5.944 -30.400  10.688  1.00  0.00           H
ATOM   2540  CA  THR B 265      -4.284 -29.557  11.769  1.00  0.00           C
ATOM   2541  CB  THR B 265      -3.975 -28.810  10.470  1.00  0.00           C
ATOM   2542  OG1 THR B 265      -5.158 -28.170   9.974  1.00  0.00           O
ATOM   2543  HG1 THR B 265      -5.593 -28.806   9.414  1.00  0.00           H
ATOM   2544  CG2 THR B 265      -3.381 -29.726   9.400  1.00  0.00           C
ATOM   2545  C   THR B 265      -4.490 -28.577  12.911  1.00  0.00           C
ATOM   2546  O   THR B 265      -5.581 -28.416  13.453  1.00  0.00           O
ATOM   2547  N   LYS B 266      -3.390 -27.873  13.237  1.00  0.00           N
ATOM   2548  H   LYS B 266      -2.509 -28.088  12.819  1.00  0.00           H
ATOM   2549  CA  LYS B 266      -3.529 -26.850  14.273  1.00  0.00           C
ATOM   2550  CB  LYS B 266      -2.161 -26.244  14.636  1.00  0.00           C
ATOM   2551  CG  LYS B 266      -2.124 -25.214  15.783  1.00  0.00           C
ATOM   2552  CD  LYS B 266      -2.838 -25.623  17.083  1.00  0.00           C
ATOM   2553  CE  LYS B 266      -2.693 -24.568  18.189  1.00  0.00           C
ATOM   2554  NZ  LYS B 266      -3.915 -24.469  18.999  1.00  0.00           N
ATOM   2555  HZ1 LYS B 266      -4.563 -25.280  18.945  1.00  0.00           H
ATOM   2556  HZ2 LYS B 266      -3.704 -24.233  19.993  1.00  0.00           H
ATOM   2557  HZ3 LYS B 266      -4.475 -23.638  18.712  1.00  0.00           H
```

ATOM	2558	C	LYS	B	266	-4.609	-25.809	14.007	1.00	0.00	C
ATOM	2559	O	LYS	B	266	-5.233	-25.341	14.939	1.00	0.00	O
ATOM	2560	N	ALA	B	267	-4.872	-25.515	12.719	1.00	0.00	N
ATOM	2561	H	ALA	B	267	-4.344	-25.927	11.979	1.00	0.00	H
ATOM	2562	CA	ALA	B	267	-5.999	-24.612	12.445	1.00	0.00	C
ATOM	2563	CB	ALA	B	267	-6.164	-24.406	10.938	1.00	0.00	C
ATOM	2564	C	ALA	B	267	-7.333	-25.071	13.034	1.00	0.00	C
ATOM	2565	O	ALA	B	267	-8.048	-24.341	13.713	1.00	0.00	O
ATOM	2566	N	TYR	B	268	-7.611	-26.365	12.792	1.00	0.00	N
ATOM	2567	H	TYR	B	268	-6.962	-26.931	12.284	1.00	0.00	H
ATOM	2568	CA	TYR	B	268	-8.790	-26.958	13.435	1.00	0.00	C
ATOM	2569	CB	TYR	B	268	-9.019	-28.393	12.946	1.00	0.00	C
ATOM	2570	CG	TYR	B	268	-9.298	-28.409	11.462	1.00	0.00	C
ATOM	2571	CD1	TYR	B	268	-8.250	-28.727	10.575	1.00	0.00	C
ATOM	2572	CE1	TYR	B	268	-8.505	-28.740	9.194	1.00	0.00	C
ATOM	2573	CD2	TYR	B	268	-10.596	-28.105	11.004	1.00	0.00	C
ATOM	2574	CE2	TYR	B	268	-10.853	-28.118	9.623	1.00	0.00	C
ATOM	2575	CZ	TYR	B	268	-9.804	-28.437	8.737	1.00	0.00	C
ATOM	2576	OH	TYR	B	268	-10.058	-28.452	7.380	1.00	0.00	O
ATOM	2577	HH	TYR	B	268	-9.343	-28.876	6.925	1.00	0.00	H
ATOM	2578	C	TYR	B	268	-8.696	-26.943	14.952	1.00	0.00	C
ATOM	2579	O	TYR	B	268	-9.656	-26.738	15.681	1.00	0.00	O
ATOM	2580	N	ASN	B	269	-7.443	-27.126	15.383	1.00	0.00	N
ATOM	2581	H	ASN	B	269	-6.717	-27.269	14.711	1.00	0.00	H
ATOM	2582	CA	ASN	B	269	-7.141	-27.023	16.807	1.00	0.00	C
ATOM	2583	CB	ASN	B	269	-5.986	-27.961	17.178	1.00	0.00	C
ATOM	2584	CG	ASN	B	269	-6.408	-29.416	17.093	1.00	0.00	C
ATOM	2585	OD1	ASN	B	269	-6.885	-30.013	18.047	1.00	0.00	O
ATOM	2586	ND2	ASN	B	269	-6.167	-29.987	15.911	1.00	0.00	N
ATOM	2587	HD21	ASN	B	269	-6.319	-30.966	15.783	1.00	0.00	H
ATOM	2588	HD22	ASN	B	269	-5.820	-29.502	15.108	1.00	0.00	H
ATOM	2589	C	ASN	B	269	-6.850	-25.609	17.303	1.00	0.00	C
ATOM	2590	O	ASN	B	269	-6.161	-25.420	18.303	1.00	0.00	O
ATOM	2591	N	VAL	B	270	-7.383	-24.620	16.564	1.00	0.00	N
ATOM	2592	H	VAL	B	270	-7.792	-24.837	15.679	1.00	0.00	H
ATOM	2593	CA	VAL	B	270	-7.431	-23.248	17.071	1.00	0.00	C
ATOM	2594	CB	VAL	B	270	-6.634	-22.239	16.211	1.00	0.00	C
ATOM	2595	CG1	VAL	B	270	-6.781	-20.807	16.737	1.00	0.00	C
ATOM	2596	CG2	VAL	B	270	-5.149	-22.564	16.117	1.00	0.00	C
ATOM	2597	C	VAL	B	270	-8.875	-22.794	17.155	1.00	0.00	C
ATOM	2598	O	VAL	B	270	-9.336	-22.334	18.198	1.00	0.00	O
ATOM	2599	N	THR	B	271	-9.541	-22.956	15.993	1.00	0.00	N
ATOM	2600	H	THR	B	271	-9.022	-23.347	15.231	1.00	0.00	H
ATOM	2601	CA	THR	B	271	-10.833	-22.343	15.666	1.00	0.00	C
ATOM	2602	CB	THR	B	271	-11.996	-22.811	16.570	1.00	0.00	C
ATOM	2603	OG1	THR	B	271	-11.930	-22.256	17.886	1.00	0.00	O
ATOM	2604	HG1	THR	B	271	-11.131	-22.606	18.266	1.00	0.00	H
ATOM	2605	CG2	THR	B	271	-12.056	-24.338	16.627	1.00	0.00	C
ATOM	2606	C	THR	B	271	-10.723	-20.831	15.474	1.00	0.00	C
ATOM	2607	O	THR	B	271	-9.658	-20.348	15.111	1.00	0.00	O
ATOM	2608	N	GLN	B	272	-11.821	-20.083	15.685	1.00	0.00	N
ATOM	2609	H	GLN	B	272	-12.617	-20.446	16.168	1.00	0.00	H
ATOM	2610	CA	GLN	B	272	-11.720	-18.661	15.344	1.00	0.00	C
ATOM	2611	CB	GLN	B	272	-13.108	-18.067	15.068	1.00	0.00	C
ATOM	2612	CG	GLN	B	272	-13.093	-16.628	14.522	1.00	0.00	C
ATOM	2613	CD	GLN	B	272	-14.512	-16.117	14.368	1.00	0.00	C
ATOM	2614	OE1	GLN	B	272	-15.483	-16.789	14.690	1.00	0.00	O
ATOM	2615	NE2	GLN	B	272	-14.597	-14.912	13.804	1.00	0.00	N
ATOM	2616	HE21	GLN	B	272	-15.499	-14.529	13.562	1.00	0.00	H

ATOM	2617	HE22	GLN	B	272	-13.818	-14.338	13.565	1.00	0.00	H
ATOM	2618	C	GLN	B	272	-10.974	-17.830	16.377	1.00	0.00	C
ATOM	2619	O	GLN	B	272	-11.571	-17.173	17.220	1.00	0.00	O
ATOM	2620	N	ALA	B	273	-9.636	-17.853	16.250	1.00	0.00	N
ATOM	2621	H	ALA	B	273	-9.256	-18.499	15.583	1.00	0.00	H
ATOM	2622	CA	ALA	B	273	-8.769	-17.140	17.197	1.00	0.00	C
ATOM	2623	CB	ALA	B	273	-7.343	-17.065	16.657	1.00	0.00	C
ATOM	2624	C	ALA	B	273	-9.205	-15.728	17.553	1.00	0.00	C
ATOM	2625	O	ALA	B	273	-9.169	-15.308	18.699	1.00	0.00	O
ATOM	2626	N	PHE	B	274	-9.660	-15.033	16.494	1.00	0.00	N
ATOM	2627	H	PHE	B	274	-9.598	-15.466	15.598	1.00	0.00	H
ATOM	2628	CA	PHE	B	274	-10.154	-13.659	16.629	1.00	0.00	C
ATOM	2629	CB	PHE	B	274	-10.860	-13.227	15.337	1.00	0.00	C
ATOM	2630	CG	PHE	B	274	-9.958	-13.365	14.131	1.00	0.00	C
ATOM	2631	CD1	PHE	B	274	-10.258	-14.347	13.161	1.00	0.00	C
ATOM	2632	CD2	PHE	B	274	-8.841	-12.512	13.985	1.00	0.00	C
ATOM	2633	CE1	PHE	B	274	-9.433	-14.477	12.028	1.00	0.00	C
ATOM	2634	CE2	PHE	B	274	-8.014	-12.640	12.853	1.00	0.00	C
ATOM	2635	CZ	PHE	B	274	-8.319	-13.622	11.887	1.00	0.00	C
ATOM	2636	C	PHE	B	274	-11.071	-13.391	17.818	1.00	0.00	C
ATOM	2637	O	PHE	B	274	-10.966	-12.386	18.506	1.00	0.00	O
ATOM	2638	N	GLY	B	275	-11.992	-14.347	18.015	1.00	0.00	N
ATOM	2639	H	GLY	B	275	-11.992	-15.172	17.451	1.00	0.00	H
ATOM	2640	CA	GLY	B	275	-12.874	-14.197	19.169	1.00	0.00	C
ATOM	2641	C	GLY	B	275	-12.558	-15.159	20.298	1.00	0.00	C
ATOM	2642	O	GLY	B	275	-12.795	-14.905	21.474	1.00	0.00	O
ATOM	2643	N	ARG	B	276	-12.026	-16.314	19.878	1.00	0.00	N
ATOM	2644	H	ARG	B	276	-11.725	-16.418	18.934	1.00	0.00	H
ATOM	2645	CA	ARG	B	276	-11.742	-17.351	20.857	1.00	0.00	C
ATOM	2646	CB	ARG	B	276	-11.759	-18.732	20.186	1.00	0.00	C
ATOM	2647	CG	ARG	B	276	-12.004	-19.910	21.139	1.00	0.00	C
ATOM	2648	CD	ARG	B	276	-10.761	-20.400	21.885	1.00	0.00	C
ATOM	2649	NE	ARG	B	276	-9.819	-21.011	20.951	1.00	0.00	N
ATOM	2650	HE	ARG	B	276	-10.165	-21.653	20.264	1.00	0.00	H
ATOM	2651	CZ	ARG	B	276	-8.494	-20.770	21.013	1.00	0.00	C
ATOM	2652	NH1	ARG	B	276	-7.988	-19.871	21.846	1.00	0.00	N
ATOM	2653	HH11	ARG	B	276	-8.565	-19.339	22.477	1.00	0.00	H
ATOM	2654	HH12	ARG	B	276	-7.012	-19.637	21.860	1.00	0.00	H
ATOM	2655	NH2	ARG	B	276	-7.706	-21.459	20.205	1.00	0.00	N
ATOM	2656	HH21	ARG	B	276	-8.115	-22.064	19.519	1.00	0.00	H
ATOM	2657	HH22	ARG	B	276	-6.698	-21.422	20.235	1.00	0.00	H
ATOM	2658	C	ARG	B	276	-10.482	-17.068	21.645	1.00	0.00	C
ATOM	2659	O	ARG	B	276	-9.372	-17.442	21.284	1.00	0.00	O
ATOM	2660	N	ARG	B	277	-10.768	-16.404	22.775	1.00	0.00	N
ATOM	2661	H	ARG	B	277	-11.696	-16.031	22.792	1.00	0.00	H
ATOM	2662	CA	ARG	B	277	-9.826	-16.096	23.854	1.00	0.00	C
ATOM	2663	CB	ARG	B	277	-10.596	-16.125	25.173	1.00	0.00	C
ATOM	2664	CG	ARG	B	277	-11.714	-15.081	25.210	1.00	0.00	C
ATOM	2665	CD	ARG	B	277	-11.156	-13.657	25.248	1.00	0.00	C
ATOM	2666	NE	ARG	B	277	-12.198	-12.655	25.037	1.00	0.00	N
ATOM	2667	HE	ARG	B	277	-12.340	-12.373	24.088	1.00	0.00	H
ATOM	2668	CZ	ARG	B	277	-12.917	-12.137	26.057	1.00	0.00	C
ATOM	2669	NH1	ARG	B	277	-12.765	-12.557	27.313	1.00	0.00	N
ATOM	2670	HH11	ARG	B	277	-12.105	-13.277	27.567	1.00	0.00	H
ATOM	2671	HH12	ARG	B	277	-13.338	-12.145	28.033	1.00	0.00	H
ATOM	2672	NH2	ARG	B	277	-13.796	-11.178	25.792	1.00	0.00	N
ATOM	2673	HH21	ARG	B	277	-13.936	-10.820	24.873	1.00	0.00	H
ATOM	2674	HH22	ARG	B	277	-14.350	-10.781	26.543	1.00	0.00	H
ATOM	2675	C	ARG	B	277	-8.574	-16.956	23.919	1.00	0.00	C

```
ATOM   2676  O   ARG B 277      -8.619 -18.180  23.821  1.00  0.00           O
ATOM   2677  N   GLY B 278      -7.455 -16.230  24.024  1.00  0.00           N
ATOM   2678  H   GLY B 278      -7.533 -15.256  24.255  1.00  0.00           H
ATOM   2679  CA  GLY B 278      -6.164 -16.820  23.674  1.00  0.00           C
ATOM   2680  C   GLY B 278      -5.986 -16.985  22.166  1.00  0.00           C
ATOM   2681  O   GLY B 278      -6.044 -18.091  21.632  1.00  0.00           O
ATOM   2682  N   PRO B 279      -5.779 -15.834  21.476  1.00  0.00           N
ATOM   2683  CD  PRO B 279      -5.616 -14.487  22.024  1.00  0.00           C
ATOM   2684  CA  PRO B 279      -5.751 -15.854  20.007  1.00  0.00           C
ATOM   2685  CB  PRO B 279      -5.965 -14.374  19.667  1.00  0.00           C
ATOM   2686  CG  PRO B 279      -5.316 -13.606  20.818  1.00  0.00           C
ATOM   2687  C   PRO B 279      -4.475 -16.444  19.415  1.00  0.00           C
ATOM   2688  O   PRO B 279      -3.561 -15.751  18.994  1.00  0.00           O
ATOM   2689  N   GLU B 280      -4.473 -17.783  19.369  1.00  0.00           N
ATOM   2690  H   GLU B 280      -5.240 -18.256  19.804  1.00  0.00           H
ATOM   2691  CA  GLU B 280      -3.273 -18.509  18.937  1.00  0.00           C
ATOM   2692  CB  GLU B 280      -3.492 -20.014  19.068  1.00  0.00           C
ATOM   2693  CG  GLU B 280      -3.955 -20.499  20.443  1.00  0.00           C
ATOM   2694  CD  GLU B 280      -4.371 -21.949  20.319  1.00  0.00           C
ATOM   2695  OE1 GLU B 280      -3.815 -22.803  20.998  1.00  0.00           O
ATOM   2696  OE2 GLU B 280      -5.239 -22.254  19.508  1.00  0.00           O
ATOM   2697  C   GLU B 280      -2.785 -18.243  17.519  1.00  0.00           C
ATOM   2698  O   GLU B 280      -1.612 -18.367  17.196  1.00  0.00           O
ATOM   2699  N   GLN B 281      -3.756 -17.941  16.649  1.00  0.00           N
ATOM   2700  H   GLN B 281      -4.665 -17.685  16.965  1.00  0.00           H
ATOM   2701  CA  GLN B 281      -3.369 -17.959  15.244  1.00  0.00           C
ATOM   2702  CB  GLN B 281      -4.501 -18.567  14.415  1.00  0.00           C
ATOM   2703  CG  GLN B 281      -4.063 -19.241  13.111  1.00  0.00           C
ATOM   2704  CD  GLN B 281      -5.210 -20.072  12.568  1.00  0.00           C
ATOM   2705  OE1 GLN B 281      -6.041 -20.583  13.302  1.00  0.00           O
ATOM   2706  NE2 GLN B 281      -5.238 -20.194  11.239  1.00  0.00           N
ATOM   2707 HE21 GLN B 281      -6.012 -20.688  10.849  1.00  0.00           H
ATOM   2708 HE22 GLN B 281      -4.549 -19.825  10.614  1.00  0.00           H
ATOM   2709  C   GLN B 281      -2.860 -16.631  14.712  1.00  0.00           C
ATOM   2710  O   GLN B 281      -3.605 -15.697  14.423  1.00  0.00           O
ATOM   2711  N   THR B 282      -1.528 -16.610  14.594  1.00  0.00           N
ATOM   2712  H   THR B 282      -0.974 -17.345  14.993  1.00  0.00           H
ATOM   2713  CA  THR B 282      -0.826 -15.447  14.056  1.00  0.00           C
ATOM   2714  CB  THR B 282       0.685 -15.743  14.078  1.00  0.00           C
ATOM   2715  OG1 THR B 282       0.953 -16.878  14.916  1.00  0.00           O
ATOM   2716  HG1 THR B 282       1.902 -16.951  14.987  1.00  0.00           H
ATOM   2717  CG2 THR B 282       1.515 -14.539  14.525  1.00  0.00           C
ATOM   2718  C   THR B 282      -1.332 -14.987  12.683  1.00  0.00           C
ATOM   2719  O   THR B 282      -2.078 -15.670  11.984  1.00  0.00           O
ATOM   2720  N   GLN B 283      -0.956 -13.752  12.328  1.00  0.00           N
ATOM   2721  H   GLN B 283      -0.209 -13.266  12.781  1.00  0.00           H
ATOM   2722  CA  GLN B 283      -1.671 -13.172  11.193  1.00  0.00           C
ATOM   2723  CB  GLN B 283      -1.895 -11.685  11.458  1.00  0.00           C
ATOM   2724  CG  GLN B 283      -2.651 -11.449  12.776  1.00  0.00           C
ATOM   2725  CD  GLN B 283      -4.112 -11.847  12.646  1.00  0.00           C
ATOM   2726  OE1 GLN B 283      -4.934 -11.072  12.181  1.00  0.00           O
ATOM   2727  NE2 GLN B 283      -4.422 -13.070  13.098  1.00  0.00           N
ATOM   2728 HE21 GLN B 283      -5.379 -13.342  13.027  1.00  0.00           H
ATOM   2729 HE22 GLN B 283      -3.792 -13.738  13.500  1.00  0.00           H
ATOM   2730  C   GLN B 283      -1.092 -13.470   9.816  1.00  0.00           C
ATOM   2731  O   GLN B 283      -1.583 -13.035   8.780  1.00  0.00           O
ATOM   2732  N   GLY B 284      -0.037 -14.293   9.840  1.00  0.00           N
ATOM   2733  H   GLY B 284       0.401 -14.585  10.691  1.00  0.00           H
ATOM   2734  CA  GLY B 284       0.331 -14.944   8.592  1.00  0.00           C
```

ATOM	2735	C	GLY	B	284	-0.050	-16.405	8.636	1.00	0.00	C
ATOM	2736	O	GLY	B	284	0.574	-17.258	8.023	1.00	0.00	O
ATOM	2737	N	ASN	B	285	-1.100	-16.652	9.425	1.00	0.00	N
ATOM	2738	H	ASN	B	285	-1.545	-15.909	9.918	1.00	0.00	H
ATOM	2739	CA	ASN	B	285	-1.469	-18.033	9.706	1.00	0.00	C
ATOM	2740	CB	ASN	B	285	-1.056	-18.494	11.117	1.00	0.00	C
ATOM	2741	CG	ASN	B	285	0.356	-18.075	11.502	1.00	0.00	C
ATOM	2742	OD1	ASN	B	285	0.764	-16.928	11.359	1.00	0.00	O
ATOM	2743	ND2	ASN	B	285	1.083	-19.047	12.057	1.00	0.00	N
ATOM	2744	HD21	ASN	B	285	2.002	-18.805	12.370	1.00	0.00	H
ATOM	2745	HD22	ASN	B	285	0.753	-19.979	12.179	1.00	0.00	H
ATOM	2746	C	ASN	B	285	-2.960	-18.199	9.513	1.00	0.00	C
ATOM	2747	O	ASN	B	285	-3.451	-19.237	9.089	1.00	0.00	O
ATOM	2748	N	PHE	B	286	-3.674	-17.097	9.797	1.00	0.00	N
ATOM	2749	H	PHE	B	286	-3.258	-16.343	10.304	1.00	0.00	H
ATOM	2750	CA	PHE	B	286	-4.906	-16.897	9.037	1.00	0.00	C
ATOM	2751	CB	PHE	B	286	-5.920	-16.052	9.816	1.00	0.00	C
ATOM	2752	CG	PHE	B	286	-6.806	-16.907	10.693	1.00	0.00	C
ATOM	2753	CD1	PHE	B	286	-6.758	-16.742	12.094	1.00	0.00	C
ATOM	2754	CD2	PHE	B	286	-7.689	-17.838	10.099	1.00	0.00	C
ATOM	2755	CE1	PHE	B	286	-7.618	-17.502	12.912	1.00	0.00	C
ATOM	2756	CE2	PHE	B	286	-8.545	-18.602	10.916	1.00	0.00	C
ATOM	2757	CZ	PHE	B	286	-8.506	-18.421	12.315	1.00	0.00	C
ATOM	2758	C	PHE	B	286	-4.591	-16.213	7.719	1.00	0.00	C
ATOM	2759	O	PHE	B	286	-3.650	-15.428	7.620	1.00	0.00	O
ATOM	2760	N	GLY	B	287	-5.409	-16.566	6.719	1.00	0.00	N
ATOM	2761	H	GLY	B	287	-6.183	-17.188	6.838	1.00	0.00	H
ATOM	2762	CA	GLY	B	287	-5.212	-15.939	5.421	1.00	0.00	C
ATOM	2763	C	GLY	B	287	-6.014	-14.664	5.257	1.00	0.00	C
ATOM	2764	O	GLY	B	287	-5.847	-13.726	6.024	1.00	0.00	O
ATOM	2765	N	ASP	B	288	-6.832	-14.625	4.200	1.00	0.00	N
ATOM	2766	H	ASP	B	288	-7.109	-15.480	3.752	1.00	0.00	H
ATOM	2767	CA	ASP	B	288	-7.172	-13.278	3.728	1.00	0.00	C
ATOM	2768	CB	ASP	B	288	-6.506	-12.969	2.389	1.00	0.00	C
ATOM	2769	CG	ASP	B	288	-5.051	-12.637	2.616	1.00	0.00	C
ATOM	2770	OD1	ASP	B	288	-4.337	-13.477	3.147	1.00	0.00	O
ATOM	2771	OD2	ASP	B	288	-4.619	-11.535	2.289	1.00	0.00	O
ATOM	2772	C	ASP	B	288	-8.640	-12.963	3.662	1.00	0.00	C
ATOM	2773	O	ASP	B	288	-9.118	-11.951	4.161	1.00	0.00	O
ATOM	2774	N	GLN	B	289	-9.377	-13.906	3.066	1.00	0.00	N
ATOM	2775	H	GLN	B	289	-8.945	-14.755	2.752	1.00	0.00	H
ATOM	2776	CA	GLN	B	289	-10.813	-13.807	3.314	1.00	0.00	C
ATOM	2777	CB	GLN	B	289	-11.582	-14.753	2.388	1.00	0.00	C
ATOM	2778	CG	GLN	B	289	-11.378	-14.474	0.894	1.00	0.00	C
ATOM	2779	CD	GLN	B	289	-11.965	-13.127	0.518	1.00	0.00	C
ATOM	2780	OE1	GLN	B	289	-13.136	-12.840	0.718	1.00	0.00	O
ATOM	2781	NE2	GLN	B	289	-11.089	-12.299	-0.048	1.00	0.00	N
ATOM	2782	HE21	GLN	B	289	-11.376	-11.376	-0.314	1.00	0.00	H
ATOM	2783	HE22	GLN	B	289	-10.123	-12.538	-0.165	1.00	0.00	H
ATOM	2784	C	GLN	B	289	-11.086	-14.113	4.778	1.00	0.00	C
ATOM	2785	O	GLN	B	289	-11.759	-13.390	5.499	1.00	0.00	O
ATOM	2786	N	GLU	B	290	-10.408	-15.196	5.171	1.00	0.00	N
ATOM	2787	H	GLU	B	290	-9.894	-15.651	4.446	1.00	0.00	H
ATOM	2788	CA	GLU	B	290	-10.451	-15.820	6.490	1.00	0.00	C
ATOM	2789	CB	GLU	B	290	-9.489	-17.020	6.534	1.00	0.00	C
ATOM	2790	CG	GLU	B	290	-9.471	-17.923	5.285	1.00	0.00	C
ATOM	2791	CD	GLU	B	290	-8.414	-17.459	4.289	1.00	0.00	C
ATOM	2792	OE1	GLU	B	290	-7.264	-17.833	4.455	1.00	0.00	O
ATOM	2793	OE2	GLU	B	290	-8.715	-16.720	3.353	1.00	0.00	O

```
ATOM   2794  C   GLU B 290     -10.234 -14.927   7.706  1.00  0.00           C
ATOM   2795  O   GLU B 290     -10.594 -15.285   8.820  1.00  0.00           O
ATOM   2796  N   LEU B 291      -9.647 -13.737   7.458  1.00  0.00           N
ATOM   2797  H   LEU B 291      -9.369 -13.504   6.528  1.00  0.00           H
ATOM   2798  CA  LEU B 291      -9.554 -12.755   8.546  1.00  0.00           C
ATOM   2799  CB  LEU B 291      -8.867 -11.473   8.072  1.00  0.00           C
ATOM   2800  CG  LEU B 291      -7.378 -11.630   7.761  1.00  0.00           C
ATOM   2801  CD1 LEU B 291      -6.805 -10.366   7.125  1.00  0.00           C
ATOM   2802  CD2 LEU B 291      -6.563 -12.062   8.982  1.00  0.00           C
ATOM   2803  C   LEU B 291     -10.899 -12.403   9.166  1.00  0.00           C
ATOM   2804  O   LEU B 291     -11.049 -12.211  10.366  1.00  0.00           O
ATOM   2805  N   ILE B 292     -11.886 -12.347   8.265  1.00  0.00           N
ATOM   2806  H   ILE B 292     -11.713 -12.547   7.300  1.00  0.00           H
ATOM   2807  CA  ILE B 292     -13.257 -12.327   8.749  1.00  0.00           C
ATOM   2808  CB  ILE B 292     -14.037 -11.191   8.073  1.00  0.00           C
ATOM   2809  CG2 ILE B 292     -15.491 -11.146   8.543  1.00  0.00           C
ATOM   2810  CG1 ILE B 292     -13.344  -9.839   8.273  1.00  0.00           C
ATOM   2811  CD1 ILE B 292     -13.307  -9.390   9.735  1.00  0.00           C
ATOM   2812  C   ILE B 292     -13.847 -13.680   8.415  1.00  0.00           C
ATOM   2813  O   ILE B 292     -13.855 -14.094   7.264  1.00  0.00           O
ATOM   2814  N   ARG B 293     -14.315 -14.378   9.459  1.00  0.00           N
ATOM   2815  H   ARG B 293     -14.429 -13.948  10.358  1.00  0.00           H
ATOM   2816  CA  ARG B 293     -14.608 -15.795   9.229  1.00  0.00           C
ATOM   2817  CB  ARG B 293     -14.851 -16.471  10.579  1.00  0.00           C
ATOM   2818  CG  ARG B 293     -15.055 -17.989  10.532  1.00  0.00           C
ATOM   2819  CD  ARG B 293     -15.606 -18.530  11.855  1.00  0.00           C
ATOM   2820  NE  ARG B 293     -16.700 -17.682  12.332  1.00  0.00           N
ATOM   2821  HE  ARG B 293     -16.474 -16.947  12.986  1.00  0.00           H
ATOM   2822  CZ  ARG B 293     -17.938 -17.749  11.816  1.00  0.00           C
ATOM   2823  NH1 ARG B 293     -18.294 -18.754  11.022  1.00  0.00           N
ATOM   2824 HH11 ARG B 293     -17.665 -19.506  10.833  1.00  0.00           H
ATOM   2825 HH12 ARG B 293     -19.206 -18.747  10.597  1.00  0.00           H
ATOM   2826  NH2 ARG B 293     -18.804 -16.786  12.086  1.00  0.00           N
ATOM   2827 HH21 ARG B 293     -18.501 -15.990  12.632  1.00  0.00           H
ATOM   2828 HH22 ARG B 293     -19.738 -16.823  11.730  1.00  0.00           H
ATOM   2829  C   ARG B 293     -15.728 -16.081   8.225  1.00  0.00           C
ATOM   2830  O   ARG B 293     -15.778 -17.120   7.579  1.00  0.00           O
ATOM   2831  N   GLN B 294     -16.632 -15.096   8.120  1.00  0.00           N
ATOM   2832  H   GLN B 294     -16.534 -14.284   8.697  1.00  0.00           H
ATOM   2833  CA  GLN B 294     -17.636 -15.114   7.058  1.00  0.00           C
ATOM   2834  CB  GLN B 294     -18.752 -16.143   7.339  1.00  0.00           C
ATOM   2835  CG  GLN B 294     -19.615 -15.834   8.567  1.00  0.00           C
ATOM   2836  CD  GLN B 294     -20.543 -16.981   8.906  1.00  0.00           C
ATOM   2837  OE1 GLN B 294     -20.435 -17.602   9.958  1.00  0.00           O
ATOM   2838  NE2 GLN B 294     -21.467 -17.242   7.980  1.00  0.00           N
ATOM   2839 HE21 GLN B 294     -22.074 -18.024   8.104  1.00  0.00           H
ATOM   2840 HE22 GLN B 294     -21.569 -16.663   7.171  1.00  0.00           H
ATOM   2841  C   GLN B 294     -18.198 -13.716   6.916  1.00  0.00           C
ATOM   2842  O   GLN B 294     -18.048 -12.891   7.807  1.00  0.00           O
ATOM   2843  N   GLY B 295     -18.896 -13.475   5.795  1.00  0.00           N
ATOM   2844  H   GLY B 295     -18.893 -14.147   5.057  1.00  0.00           H
ATOM   2845  CA  GLY B 295     -19.523 -12.156   5.640  1.00  0.00           C
ATOM   2846  C   GLY B 295     -20.376 -11.733   6.830  1.00  0.00           C
ATOM   2847  O   GLY B 295     -20.309 -10.625   7.344  1.00  0.00           O
ATOM   2848  N   THR B 296     -21.162 -12.711   7.285  1.00  0.00           N
ATOM   2849  H   THR B 296     -21.189 -13.624   6.881  1.00  0.00           H
ATOM   2850  CA  THR B 296     -22.017 -12.453   8.436  1.00  0.00           C
ATOM   2851  CB  THR B 296     -23.254 -13.333   8.270  1.00  0.00           C
ATOM   2852  OG1 THR B 296     -22.882 -14.585   7.666  1.00  0.00           O
```

ATOM	2853	HG1	THR	B	296	-23.700	-15.007	7.422	1.00	0.00	H
ATOM	2854	CG2	THR	B	296	-24.314	-12.640	7.412	1.00	0.00	C
ATOM	2855	C	THR	B	296	-21.365	-12.639	9.805	1.00	0.00	C
ATOM	2856	O	THR	B	296	-22.049	-12.859	10.802	1.00	0.00	O
ATOM	2857	N	ASP	B	297	-20.029	-12.553	9.823	1.00	0.00	N
ATOM	2858	H	ASP	B	297	-19.513	-12.349	8.990	1.00	0.00	H
ATOM	2859	CA	ASP	B	297	-19.297	-12.658	11.088	1.00	0.00	C
ATOM	2860	CB	ASP	B	297	-18.054	-13.506	10.802	1.00	0.00	C
ATOM	2861	CG	ASP	B	297	-17.133	-13.749	11.968	1.00	0.00	C
ATOM	2862	OD1	ASP	B	297	-17.485	-14.519	12.855	1.00	0.00	O
ATOM	2863	OD2	ASP	B	297	-16.026	-13.225	11.941	1.00	0.00	O
ATOM	2864	C	ASP	B	297	-18.981	-11.264	11.604	1.00	0.00	C
ATOM	2865	O	ASP	B	297	-19.321	-10.877	12.716	1.00	0.00	O
ATOM	2866	N	TYR	B	298	-18.400	-10.501	10.657	1.00	0.00	N
ATOM	2867	H	TYR	B	298	-18.134	-10.985	9.824	1.00	0.00	H
ATOM	2868	CA	TYR	B	298	-17.970	-9.109	10.834	1.00	0.00	C
ATOM	2869	CB	TYR	B	298	-18.022	-8.397	9.476	1.00	0.00	C
ATOM	2870	CG	TYR	B	298	-17.548	-6.960	9.543	1.00	0.00	C
ATOM	2871	CD1	TYR	B	298	-16.197	-6.674	9.827	1.00	0.00	C
ATOM	2872	CE1	TYR	B	298	-15.786	-5.332	9.869	1.00	0.00	C
ATOM	2873	CD2	TYR	B	298	-18.488	-5.939	9.306	1.00	0.00	C
ATOM	2874	CE2	TYR	B	298	-18.074	-4.599	9.337	1.00	0.00	C
ATOM	2875	CZ	TYR	B	298	-16.727	-4.312	9.625	1.00	0.00	C
ATOM	2876	OH	TYR	B	298	-16.329	-2.992	9.672	1.00	0.00	O
ATOM	2877	HH	TYR	B	298	-15.660	-2.868	10.333	1.00	0.00	H
ATOM	2878	C	TYR	B	298	-18.613	-8.276	11.935	1.00	0.00	C
ATOM	2879	O	TYR	B	298	-17.918	-7.680	12.740	1.00	0.00	O
ATOM	2880	N	LYS	B	299	-19.958	-8.263	11.925	1.00	0.00	N
ATOM	2881	H	LYS	B	299	-20.383	-8.772	11.181	1.00	0.00	H
ATOM	2882	CA	LYS	B	299	-20.790	-7.622	12.956	1.00	0.00	C
ATOM	2883	CB	LYS	B	299	-21.793	-8.623	13.526	1.00	0.00	C
ATOM	2884	CG	LYS	B	299	-22.683	-9.368	12.538	1.00	0.00	C
ATOM	2885	CD	LYS	B	299	-23.423	-10.457	13.312	1.00	0.00	C
ATOM	2886	CE	LYS	B	299	-24.353	-11.315	12.460	1.00	0.00	C
ATOM	2887	NZ	LYS	B	299	-23.954	-12.721	12.605	1.00	0.00	N
ATOM	2888	HZ1	LYS	B	299	-23.753	-12.942	13.600	1.00	0.00	H
ATOM	2889	HZ2	LYS	B	299	-24.692	-13.351	12.234	1.00	0.00	H
ATOM	2890	HZ3	LYS	B	299	-23.079	-12.873	12.062	1.00	0.00	H
ATOM	2891	C	LYS	B	299	-20.121	-6.938	14.149	1.00	0.00	C
ATOM	2892	O	LYS	B	299	-20.195	-5.728	14.332	1.00	0.00	O
ATOM	2893	N	HIS	B	300	-19.508	-7.789	14.984	1.00	0.00	N
ATOM	2894	H	HIS	B	300	-19.338	-8.729	14.687	1.00	0.00	H
ATOM	2895	CA	HIS	B	300	-19.018	-7.266	16.257	1.00	0.00	C
ATOM	2896	CB	HIS	B	300	-19.009	-8.367	17.316	1.00	0.00	C
ATOM	2897	CG	HIS	B	300	-20.422	-8.862	17.510	1.00	0.00	C
ATOM	2898	ND1	HIS	B	300	-21.394	-8.129	18.081	1.00	0.00	N
ATOM	2899	HD1	HIS	B	300	-21.304	-7.209	18.424	1.00	0.00	H
ATOM	2900	CD2	HIS	B	300	-20.955	-10.097	17.129	1.00	0.00	C
ATOM	2901	NE2	HIS	B	300	-22.265	-10.095	17.479	1.00	0.00	N
ATOM	2902	CE1	HIS	B	300	-22.534	-8.885	18.067	1.00	0.00	C
ATOM	2903	C	HIS	B	300	-17.678	-6.566	16.182	1.00	0.00	C
ATOM	2904	O	HIS	B	300	-17.377	-5.650	16.936	1.00	0.00	O
ATOM	2905	N	TRP	B	301	-16.875	-7.014	15.210	1.00	0.00	N
ATOM	2906	H	TRP	B	301	-17.208	-7.696	14.559	1.00	0.00	H
ATOM	2907	CA	TRP	B	301	-15.549	-6.416	15.046	1.00	0.00	C
ATOM	2908	CB	TRP	B	301	-14.776	-7.101	13.904	1.00	0.00	C
ATOM	2909	CG	TRP	B	301	-14.832	-8.612	14.022	1.00	0.00	C
ATOM	2910	CD2	TRP	B	301	-14.415	-9.449	15.123	1.00	0.00	C
ATOM	2911	CE2	TRP	B	301	-14.697	-10.809	14.754	1.00	0.00	C

```
ATOM   2912  CE3 TRP B 301     -13.833  -9.169  16.377  1.00  0.00           C
ATOM   2913  CD1 TRP B 301     -15.330  -9.505  13.061  1.00  0.00           C
ATOM   2914  NE1 TRP B 301     -15.256 -10.795  13.483  1.00  0.00           N
ATOM   2915  HE1 TRP B 301     -15.556 -11.592  12.981  1.00  0.00           H
ATOM   2916  CZ2 TRP B 301     -14.398 -11.856  15.649  1.00  0.00           C
ATOM   2917  CZ3 TRP B 301     -13.536 -10.226  17.263  1.00  0.00           C
ATOM   2918  CH2 TRP B 301     -13.821 -11.559  16.901  1.00  0.00           C
ATOM   2919  C   TRP B 301     -15.451  -4.880  15.008  1.00  0.00           C
ATOM   2920  O   TRP B 301     -14.646  -4.311  15.737  1.00  0.00           O
ATOM   2921  N   PRO B 302     -16.296  -4.177  14.192  1.00  0.00           N
ATOM   2922  CD  PRO B 302     -17.276  -4.639  13.211  1.00  0.00           C
ATOM   2923  CA  PRO B 302     -16.260  -2.709  14.265  1.00  0.00           C
ATOM   2924  CB  PRO B 302     -17.219  -2.276  13.148  1.00  0.00           C
ATOM   2925  CG  PRO B 302     -18.183  -3.442  12.969  1.00  0.00           C
ATOM   2926  C   PRO B 302     -16.623  -2.136  15.632  1.00  0.00           C
ATOM   2927  O   PRO B 302     -16.127  -1.098  16.053  1.00  0.00           O
ATOM   2928  N   GLN B 303     -17.504  -2.862  16.339  1.00  0.00           N
ATOM   2929  H   GLN B 303     -17.813  -3.756  16.013  1.00  0.00           H
ATOM   2930  CA  GLN B 303     -17.806  -2.414  17.699  1.00  0.00           C
ATOM   2931  CB  GLN B 303     -18.968  -3.220  18.284  1.00  0.00           C
ATOM   2932  CG  GLN B 303     -20.184  -3.291  17.356  1.00  0.00           C
ATOM   2933  CD  GLN B 303     -21.267  -4.125  18.007  1.00  0.00           C
ATOM   2934  OE1 GLN B 303     -21.098  -5.291  18.340  1.00  0.00           O
ATOM   2935  NE2 GLN B 303     -22.416  -3.468  18.186  1.00  0.00           N
ATOM   2936 HE21 GLN B 303     -23.146  -3.913  18.699  1.00  0.00           H
ATOM   2937 HE22 GLN B 303     -22.555  -2.551  17.806  1.00  0.00           H
ATOM   2938  C   GLN B 303     -16.578  -2.486  18.594  1.00  0.00           C
ATOM   2939  O   GLN B 303     -16.213  -1.550  19.293  1.00  0.00           O
ATOM   2940  N   ILE B 304     -15.904  -3.640  18.477  1.00  0.00           N
ATOM   2941  H   ILE B 304     -16.264  -4.357  17.879  1.00  0.00           H
ATOM   2942  CA  ILE B 304     -14.666  -3.822  19.235  1.00  0.00           C
ATOM   2943  CB  ILE B 304     -14.169  -5.269  19.087  1.00  0.00           C
ATOM   2944  CG2 ILE B 304     -12.840  -5.531  19.809  1.00  0.00           C
ATOM   2945  CG1 ILE B 304     -15.274  -6.206  19.591  1.00  0.00           C
ATOM   2946  CD1 ILE B 304     -14.954  -7.693  19.451  1.00  0.00           C
ATOM   2947  C   ILE B 304     -13.593  -2.779  18.941  1.00  0.00           C
ATOM   2948  O   ILE B 304     -12.956  -2.246  19.840  1.00  0.00           O
ATOM   2949  N   ALA B 305     -13.466  -2.443  17.648  1.00  0.00           N
ATOM   2950  H   ALA B 305     -13.987  -2.947  16.956  1.00  0.00           H
ATOM   2951  CA  ALA B 305     -12.542  -1.358  17.298  1.00  0.00           C
ATOM   2952  CB  ALA B 305     -12.429  -1.220  15.779  1.00  0.00           C
ATOM   2953  C   ALA B 305     -12.876   0.005  17.905  1.00  0.00           C
ATOM   2954  O   ALA B 305     -12.016   0.859  18.119  1.00  0.00           O
ATOM   2955  N   GLN B 306     -14.176   0.165  18.202  1.00  0.00           N
ATOM   2956  H   GLN B 306     -14.843  -0.552  17.992  1.00  0.00           H
ATOM   2957  CA  GLN B 306     -14.593   1.362  18.929  1.00  0.00           C
ATOM   2958  CB  GLN B 306     -16.050   1.695  18.605  1.00  0.00           C
ATOM   2959  CG  GLN B 306     -16.223   2.027  17.120  1.00  0.00           C
ATOM   2960  CD  GLN B 306     -17.691   2.183  16.788  1.00  0.00           C
ATOM   2961  OE1 GLN B 306     -18.277   3.252  16.910  1.00  0.00           O
ATOM   2962  NE2 GLN B 306     -18.265   1.055  16.361  1.00  0.00           N
ATOM   2963 HE21 GLN B 306     -19.245   1.016  16.155  1.00  0.00           H
ATOM   2964 HE22 GLN B 306     -17.690   0.245  16.245  1.00  0.00           H
ATOM   2965  C   GLN B 306     -14.332   1.333  20.433  1.00  0.00           C
ATOM   2966  O   GLN B 306     -14.296   2.360  21.101  1.00  0.00           O
ATOM   2967  N   PHE B 307     -14.105   0.120  20.951  1.00  0.00           N
ATOM   2968  H   PHE B 307     -14.173  -0.704  20.388  1.00  0.00           H
ATOM   2969  CA  PHE B 307     -13.726   0.079  22.364  1.00  0.00           C
ATOM   2970  CB  PHE B 307     -14.260  -1.191  23.051  1.00  0.00           C
```

ATOM	2971	CG	PHE	B	307	-15.679	-1.535	22.640	1.00	0.00	C
ATOM	2972	CD1	PHE	B	307	-15.970	-2.874	22.300	1.00	0.00	C
ATOM	2973	CD2	PHE	B	307	-16.687	-0.544	22.602	1.00	0.00	C
ATOM	2974	CE1	PHE	B	307	-17.278	-3.229	21.916	1.00	0.00	C
ATOM	2975	CE2	PHE	B	307	-17.994	-0.895	22.213	1.00	0.00	C
ATOM	2976	CZ	PHE	B	307	-18.277	-2.234	21.876	1.00	0.00	C
ATOM	2977	C	PHE	B	307	-12.220	0.191	22.560	1.00	0.00	C
ATOM	2978	O	PHE	B	307	-11.690	1.062	23.256	1.00	0.00	O
ATOM	2979	N	ALA	B	308	-11.564	-0.760	21.873	1.00	0.00	N
ATOM	2980	H	ALA	B	308	-12.111	-1.379	21.306	1.00	0.00	H
ATOM	2981	CA	ALA	B	308	-10.111	-0.913	21.861	1.00	0.00	C
ATOM	2982	CB	ALA	B	308	-9.736	-2.052	20.913	1.00	0.00	C
ATOM	2983	C	ALA	B	308	-9.399	0.325	21.362	1.00	0.00	C
ATOM	2984	O	ALA	B	308	-9.759	0.867	20.326	1.00	0.00	O
ATOM	2985	N	PRO	B	309	-8.368	0.774	22.120	1.00	0.00	N
ATOM	2986	CD	PRO	B	309	-7.882	0.204	23.371	1.00	0.00	C
ATOM	2987	CA	PRO	B	309	-7.629	1.971	21.703	1.00	0.00	C
ATOM	2988	CB	PRO	B	309	-6.638	2.182	22.861	1.00	0.00	C
ATOM	2989	CG	PRO	B	309	-6.511	0.833	23.569	1.00	0.00	C
ATOM	2990	C	PRO	B	309	-7.038	1.887	20.295	1.00	0.00	C
ATOM	2991	O	PRO	B	309	-7.703	2.258	19.333	1.00	0.00	O
ATOM	2992	N	SER	B	310	-5.801	1.383	20.200	1.00	0.00	N
ATOM	2993	H	SER	B	310	-5.240	1.148	20.993	1.00	0.00	H
ATOM	2994	CA	SER	B	310	-5.140	1.390	18.899	1.00	0.00	C
ATOM	2995	CB	SER	B	310	-4.072	2.479	18.944	1.00	0.00	C
ATOM	2996	OG	SER	B	310	-3.338	2.347	20.168	1.00	0.00	O
ATOM	2997	HG	SER	B	310	-2.747	1.612	20.025	1.00	0.00	H
ATOM	2998	C	SER	B	310	-4.547	0.031	18.573	1.00	0.00	C
ATOM	2999	O	SER	B	310	-3.352	-0.205	18.704	1.00	0.00	O
ATOM	3000	N	ALA	B	311	-5.456	-0.878	18.204	1.00	0.00	N
ATOM	3001	H	ALA	B	311	-6.388	-0.589	17.995	1.00	0.00	H
ATOM	3002	CA	ALA	B	311	-5.007	-2.261	18.061	1.00	0.00	C
ATOM	3003	CB	ALA	B	311	-6.183	-3.217	18.293	1.00	0.00	C
ATOM	3004	C	ALA	B	311	-4.334	-2.573	16.731	1.00	0.00	C
ATOM	3005	O	ALA	B	311	-4.227	-1.747	15.830	1.00	0.00	O
ATOM	3006	N	SER	B	312	-3.893	-3.841	16.642	1.00	0.00	N
ATOM	3007	H	SER	B	312	-4.020	-4.464	17.414	1.00	0.00	H
ATOM	3008	CA	SER	B	312	-3.424	-4.287	15.335	1.00	0.00	C
ATOM	3009	CB	SER	B	312	-2.375	-5.393	15.487	1.00	0.00	C
ATOM	3010	OG	SER	B	312	-1.178	-4.876	16.090	1.00	0.00	O
ATOM	3011	HG	SER	B	312	-1.464	-4.247	16.749	1.00	0.00	H
ATOM	3012	C	SER	B	312	-4.540	-4.670	14.366	1.00	0.00	C
ATOM	3013	O	SER	B	312	-5.036	-3.823	13.637	1.00	0.00	O
ATOM	3014	N	ALA	B	313	-4.894	-5.972	14.356	1.00	0.00	N
ATOM	3015	H	ALA	B	313	-4.602	-6.608	15.068	1.00	0.00	H
ATOM	3016	CA	ALA	B	313	-5.634	-6.502	13.204	1.00	0.00	C
ATOM	3017	CB	ALA	B	313	-5.905	-7.997	13.395	1.00	0.00	C
ATOM	3018	C	ALA	B	313	-6.929	-5.780	12.862	1.00	0.00	C
ATOM	3019	O	ALA	B	313	-7.038	-5.093	11.857	1.00	0.00	O
ATOM	3020	N	PHE	B	314	-7.898	-5.927	13.785	1.00	0.00	N
ATOM	3021	H	PHE	B	314	-7.748	-6.538	14.559	1.00	0.00	H
ATOM	3022	CA	PHE	B	314	-9.211	-5.307	13.562	1.00	0.00	C
ATOM	3023	CB	PHE	B	314	-10.129	-5.551	14.768	1.00	0.00	C
ATOM	3024	CG	PHE	B	314	-10.158	-7.020	15.126	1.00	0.00	C
ATOM	3025	CD1	PHE	B	314	-9.504	-7.449	16.301	1.00	0.00	C
ATOM	3026	CD2	PHE	B	314	-10.821	-7.939	14.282	1.00	0.00	C
ATOM	3027	CE1	PHE	B	314	-9.497	-8.817	16.628	1.00	0.00	C
ATOM	3028	CE2	PHE	B	314	-10.813	-9.308	14.607	1.00	0.00	C
ATOM	3029	CZ	PHE	B	314	-10.147	-9.732	15.776	1.00	0.00	C

```
ATOM   3030  C   PHE B 314      -9.147  -3.819  13.254  1.00  0.00           C
ATOM   3031  O   PHE B 314      -9.822  -3.271  12.393  1.00  0.00           O
ATOM   3032  N   PHE B 315      -8.244  -3.192  14.012  1.00  0.00           N
ATOM   3033  H   PHE B 315      -7.666  -3.746  14.605  1.00  0.00           H
ATOM   3034  CA  PHE B 315      -7.959  -1.775  13.832  1.00  0.00           C
ATOM   3035  CB  PHE B 315      -7.372  -1.308  15.168  1.00  0.00           C
ATOM   3036  CG  PHE B 315      -7.320   0.188  15.379  1.00  0.00           C
ATOM   3037  CD1 PHE B 315      -8.347   0.801  16.128  1.00  0.00           C
ATOM   3038  CD2 PHE B 315      -6.237   0.941  14.872  1.00  0.00           C
ATOM   3039  CE1 PHE B 315      -8.273   2.180  16.398  1.00  0.00           C
ATOM   3040  CE2 PHE B 315      -6.163   2.321  15.141  1.00  0.00           C
ATOM   3041  CZ  PHE B 315      -7.174   2.922  15.918  1.00  0.00           C
ATOM   3042  C   PHE B 315      -7.022  -1.551  12.648  1.00  0.00           C
ATOM   3043  O   PHE B 315      -5.887  -1.115  12.804  1.00  0.00           O
ATOM   3044  N   GLY B 316      -7.532  -1.883  11.454  1.00  0.00           N
ATOM   3045  H   GLY B 316      -8.447  -2.290  11.412  1.00  0.00           H
ATOM   3046  CA  GLY B 316      -6.688  -1.737  10.271  1.00  0.00           C
ATOM   3047  C   GLY B 316      -6.214  -3.050   9.667  1.00  0.00           C
ATOM   3048  O   GLY B 316      -5.033  -3.397   9.700  1.00  0.00           O
ATOM   3049  N   MET B 317      -7.197  -3.755   9.090  1.00  0.00           N
ATOM   3050  H   MET B 317      -8.093  -3.329   8.967  1.00  0.00           H
ATOM   3051  CA  MET B 317      -6.894  -5.056   8.495  1.00  0.00           C
ATOM   3052  CB  MET B 317      -8.168  -5.833   8.175  1.00  0.00           C
ATOM   3053  CG  MET B 317      -8.782  -6.571   9.364  1.00  0.00           C
ATOM   3054  SD  MET B 317      -7.721  -7.883   9.989  1.00  0.00           S
ATOM   3055  CE  MET B 317      -8.941  -8.733  11.004  1.00  0.00           C
ATOM   3056  C   MET B 317      -6.017  -5.011   7.260  1.00  0.00           C
ATOM   3057  O   MET B 317      -5.267  -5.942   6.989  1.00  0.00           O
ATOM   3058  N   SER B 318      -6.093  -3.888   6.527  1.00  0.00           N
ATOM   3059  H   SER B 318      -6.712  -3.126   6.730  1.00  0.00           H
ATOM   3060  CA  SER B 318      -5.252  -3.833   5.329  1.00  0.00           C
ATOM   3061  CB  SER B 318      -5.776  -2.771   4.364  1.00  0.00           C
ATOM   3062  OG  SER B 318      -6.154  -1.603   5.098  1.00  0.00           O
ATOM   3063  HG  SER B 318      -6.351  -0.949   4.438  1.00  0.00           H
ATOM   3064  C   SER B 318      -3.749  -3.700   5.548  1.00  0.00           C
ATOM   3065  O   SER B 318      -2.966  -3.649   4.611  1.00  0.00           O
ATOM   3066  N   ARG B 319      -3.348  -3.696   6.835  1.00  0.00           N
ATOM   3067  H   ARG B 319      -4.012  -3.690   7.581  1.00  0.00           H
ATOM   3068  CA  ARG B 319      -1.916  -3.910   7.059  1.00  0.00           C
ATOM   3069  CB  ARG B 319      -1.503  -3.584   8.493  1.00  0.00           C
ATOM   3070  CG  ARG B 319      -1.877  -2.215   9.060  1.00  0.00           C
ATOM   3071  CD  ARG B 319      -1.315  -2.159  10.483  1.00  0.00           C
ATOM   3072  NE  ARG B 319      -1.873  -1.096  11.322  1.00  0.00           N
ATOM   3073  HE  ARG B 319      -1.370  -0.233  11.396  1.00  0.00           H
ATOM   3074  CZ  ARG B 319      -2.973  -1.350  12.067  1.00  0.00           C
ATOM   3075  NH1 ARG B 319      -3.735  -2.406  11.820  1.00  0.00           N
ATOM   3076 HH11 ARG B 319      -3.626  -2.991  11.011  1.00  0.00           H
ATOM   3077 HH12 ARG B 319      -4.502  -2.627  12.437  1.00  0.00           H
ATOM   3078  NH2 ARG B 319      -3.311  -0.542  13.064  1.00  0.00           N
ATOM   3079 HH21 ARG B 319      -2.765   0.254  13.327  1.00  0.00           H
ATOM   3080 HH22 ARG B 319      -4.163  -0.735  13.569  1.00  0.00           H
ATOM   3081  C   ARG B 319      -1.438  -5.329   6.761  1.00  0.00           C
ATOM   3082  O   ARG B 319      -0.250  -5.599   6.620  1.00  0.00           O
ATOM   3083  N   ILE B 320      -2.438  -6.225   6.762  1.00  0.00           N
ATOM   3084  H   ILE B 320      -3.381  -5.902   6.794  1.00  0.00           H
ATOM   3085  CA  ILE B 320      -2.205  -7.665   6.761  1.00  0.00           C
ATOM   3086  CB  ILE B 320      -2.804  -8.243   8.063  1.00  0.00           C
ATOM   3087  CG2 ILE B 320      -2.830  -9.774   8.121  1.00  0.00           C
ATOM   3088  CG1 ILE B 320      -2.098  -7.639   9.282  1.00  0.00           C
```

ATOM	3089	CD1	ILE	B	320	-2.837	-7.899	10.594	1.00	0.00	C
ATOM	3090	C	ILE	B	320	-2.794	-8.324	5.519	1.00	0.00	C
ATOM	3091	O	ILE	B	320	-2.130	-9.038	4.774	1.00	0.00	O
ATOM	3092	N	GLY	B	321	-4.098	-8.080	5.346	1.00	0.00	N
ATOM	3093	H	GLY	B	321	-4.591	-7.402	5.892	1.00	0.00	H
ATOM	3094	CA	GLY	B	321	-4.764	-8.789	4.261	1.00	0.00	C
ATOM	3095	C	GLY	B	321	-4.842	-7.979	2.989	1.00	0.00	C
ATOM	3096	O	GLY	B	321	-5.240	-6.820	2.996	1.00	0.00	O
ATOM	3097	N	MET	B	322	-4.443	-8.649	1.899	1.00	0.00	N
ATOM	3098	H	MET	B	322	-4.166	-9.606	2.000	1.00	0.00	H
ATOM	3099	CA	MET	B	322	-4.583	-8.000	0.598	1.00	0.00	C
ATOM	3100	CB	MET	B	322	-3.245	-7.848	-0.122	1.00	0.00	C
ATOM	3101	CG	MET	B	322	-2.459	-6.623	0.349	1.00	0.00	C
ATOM	3102	SD	MET	B	322	-1.840	-6.753	2.031	1.00	0.00	S
ATOM	3103	CE	MET	B	322	-1.264	-5.059	2.167	1.00	0.00	C
ATOM	3104	C	MET	B	322	-5.584	-8.678	-0.309	1.00	0.00	C
ATOM	3105	O	MET	B	322	-6.402	-8.026	-0.944	1.00	0.00	O
ATOM	3106	N	GLU	B	323	-5.523	-10.020	-0.329	1.00	0.00	N
ATOM	3107	H	GLU	B	323	-4.934	-10.520	0.310	1.00	0.00	H
ATOM	3108	CA	GLU	B	323	-6.561	-10.716	-1.096	1.00	0.00	C
ATOM	3109	CB	GLU	B	323	-5.979	-12.002	-1.714	1.00	0.00	C
ATOM	3110	CG	GLU	B	323	-4.945	-11.756	-2.839	1.00	0.00	C
ATOM	3111	CD	GLU	B	323	-4.439	-13.039	-3.517	1.00	0.00	C
ATOM	3112	OE1	GLU	B	323	-3.454	-12.985	-4.251	1.00	0.00	O
ATOM	3113	OE2	GLU	B	323	-5.008	-14.110	-3.327	1.00	0.00	O
ATOM	3114	C	GLU	B	323	-7.794	-10.958	-0.218	1.00	0.00	C
ATOM	3115	O	GLU	B	323	-8.300	-12.065	-0.051	1.00	0.00	O
ATOM	3116	N	VAL	B	324	-8.206	-9.833	0.397	1.00	0.00	N
ATOM	3117	H	VAL	B	324	-7.965	-8.974	-0.059	1.00	0.00	H
ATOM	3118	CA	VAL	B	324	-8.913	-9.821	1.682	1.00	0.00	C
ATOM	3119	CB	VAL	B	324	-8.353	-8.630	2.502	1.00	0.00	C
ATOM	3120	CG1	VAL	B	324	-8.747	-7.279	1.892	1.00	0.00	C
ATOM	3121	CG2	VAL	B	324	-8.644	-8.694	4.006	1.00	0.00	C
ATOM	3122	C	VAL	B	324	-10.434	-9.788	1.557	1.00	0.00	C
ATOM	3123	O	VAL	B	324	-10.987	-9.461	0.512	1.00	0.00	O
ATOM	3124	N	THR	B	325	-11.107	-10.129	2.665	1.00	0.00	N
ATOM	3125	H	THR	B	325	-10.636	-10.447	3.492	1.00	0.00	H
ATOM	3126	CA	THR	B	325	-12.537	-9.827	2.728	1.00	0.00	C
ATOM	3127	CB	THR	B	325	-13.143	-10.433	3.999	1.00	0.00	C
ATOM	3128	OG1	THR	B	325	-12.124	-10.661	4.982	1.00	0.00	O
ATOM	3129	HG1	THR	B	325	-12.107	-11.603	5.150	1.00	0.00	H
ATOM	3130	CG2	THR	B	325	-13.958	-11.696	3.727	1.00	0.00	C
ATOM	3131	C	THR	B	325	-12.827	-8.327	2.706	1.00	0.00	C
ATOM	3132	O	THR	B	325	-12.184	-7.531	3.386	1.00	0.00	O
ATOM	3133	N	PRO	B	326	-13.868	-7.946	1.922	1.00	0.00	N
ATOM	3134	CD	PRO	B	326	-14.617	-8.780	0.987	1.00	0.00	C
ATOM	3135	CA	PRO	B	326	-14.352	-6.556	1.961	1.00	0.00	C
ATOM	3136	CB	PRO	B	326	-15.637	-6.628	1.131	1.00	0.00	C
ATOM	3137	CG	PRO	B	326	-15.405	-7.777	0.152	1.00	0.00	C
ATOM	3138	C	PRO	B	326	-14.568	-6.017	3.372	1.00	0.00	C
ATOM	3139	O	PRO	B	326	-14.148	-4.924	3.738	1.00	0.00	O
ATOM	3140	N	SER	B	327	-15.188	-6.886	4.183	1.00	0.00	N
ATOM	3141	H	SER	B	327	-15.643	-7.707	3.837	1.00	0.00	H
ATOM	3142	CA	SER	B	327	-15.366	-6.579	5.599	1.00	0.00	C
ATOM	3143	CB	SER	B	327	-16.091	-7.762	6.236	1.00	0.00	C
ATOM	3144	OG	SER	B	327	-17.070	-8.253	5.307	1.00	0.00	O
ATOM	3145	HG	SER	B	327	-17.932	-7.985	5.634	1.00	0.00	H
ATOM	3146	C	SER	B	327	-14.120	-6.142	6.369	1.00	0.00	C
ATOM	3147	O	SER	B	327	-14.169	-5.259	7.214	1.00	0.00	O

ATOM	3148	N	GLY	B	328	-12.976	-6.750	6.008	1.00	0.00	N
ATOM	3149	H	GLY	B	328	-12.952	-7.440	5.284	1.00	0.00	H
ATOM	3150	CA	GLY	B	328	-11.720	-6.299	6.615	1.00	0.00	C
ATOM	3151	C	GLY	B	328	-11.339	-4.863	6.272	1.00	0.00	C
ATOM	3152	O	GLY	B	328	-10.767	-4.111	7.058	1.00	0.00	O
ATOM	3153	N	THR	B	329	-11.721	-4.481	5.049	1.00	0.00	N
ATOM	3154	H	THR	B	329	-12.231	-5.110	4.462	1.00	0.00	H
ATOM	3155	CA	THR	B	329	-11.527	-3.075	4.699	1.00	0.00	C
ATOM	3156	CB	THR	B	329	-11.756	-2.879	3.198	1.00	0.00	C
ATOM	3157	OG1	THR	B	329	-11.059	-3.894	2.464	1.00	0.00	O
ATOM	3158	HG1	THR	B	329	-11.478	-4.725	2.650	1.00	0.00	H
ATOM	3159	CG2	THR	B	329	-11.317	-1.492	2.724	1.00	0.00	C
ATOM	3160	C	THR	B	329	-12.379	-2.130	5.547	1.00	0.00	C
ATOM	3161	O	THR	B	329	-11.926	-1.102	6.038	1.00	0.00	O
ATOM	3162	N	TRP	B	330	-13.629	-2.568	5.766	1.00	0.00	N
ATOM	3163	H	TRP	B	330	-13.953	-3.412	5.332	1.00	0.00	H
ATOM	3164	CA	TRP	B	330	-14.488	-1.778	6.653	1.00	0.00	C
ATOM	3165	CB	TRP	B	330	-15.939	-2.249	6.542	1.00	0.00	C
ATOM	3166	CG	TRP	B	330	-16.432	-2.134	5.116	1.00	0.00	C
ATOM	3167	CD2	TRP	B	330	-17.222	-3.092	4.380	1.00	0.00	C
ATOM	3168	CE2	TRP	B	330	-17.447	-2.544	3.074	1.00	0.00	C
ATOM	3169	CE3	TRP	B	330	-17.757	-4.354	4.715	1.00	0.00	C
ATOM	3170	CD1	TRP	B	330	-16.227	-1.067	4.226	1.00	0.00	C
ATOM	3171	NE1	TRP	B	330	-16.820	-1.303	3.025	1.00	0.00	N
ATOM	3172	HE1	TRP	B	330	-16.817	-0.698	2.252	1.00	0.00	H
ATOM	3173	CZ2	TRP	B	330	-18.201	-3.270	2.128	1.00	0.00	C
ATOM	3174	CZ3	TRP	B	330	-18.510	-5.070	3.762	1.00	0.00	C
ATOM	3175	CH2	TRP	B	330	-18.730	-4.532	2.475	1.00	0.00	C
ATOM	3176	C	TRP	B	330	-14.011	-1.714	8.104	1.00	0.00	C
ATOM	3177	O	TRP	B	330	-14.101	-0.702	8.792	1.00	0.00	O
ATOM	3178	N	LEU	B	331	-13.398	-2.831	8.528	1.00	0.00	N
ATOM	3179	H	LEU	B	331	-13.472	-3.665	7.982	1.00	0.00	H
ATOM	3180	CA	LEU	B	331	-12.662	-2.789	9.793	1.00	0.00	C
ATOM	3181	CB	LEU	B	331	-12.027	-4.137	10.100	1.00	0.00	C
ATOM	3182	CG	LEU	B	331	-12.795	-4.909	11.161	1.00	0.00	C
ATOM	3183	CD1	LEU	B	331	-12.160	-6.273	11.399	1.00	0.00	C
ATOM	3184	CD2	LEU	B	331	-12.983	-4.101	12.446	1.00	0.00	C
ATOM	3185	C	LEU	B	331	-11.595	-1.717	9.839	1.00	0.00	C
ATOM	3186	O	LEU	B	331	-11.495	-0.930	10.769	1.00	0.00	O
ATOM	3187	N	THR	B	332	-10.831	-1.687	8.742	1.00	0.00	N
ATOM	3188	H	THR	B	332	-10.984	-2.371	8.029	1.00	0.00	H
ATOM	3189	CA	THR	B	332	-9.822	-0.641	8.604	1.00	0.00	C
ATOM	3190	CB	THR	B	332	-9.052	-0.874	7.303	1.00	0.00	C
ATOM	3191	OG1	THR	B	332	-8.625	-2.248	7.250	1.00	0.00	O
ATOM	3192	HG1	THR	B	332	-9.386	-2.764	6.990	1.00	0.00	H
ATOM	3193	CG2	THR	B	332	-7.861	0.077	7.155	1.00	0.00	C
ATOM	3194	C	THR	B	332	-10.355	0.784	8.746	1.00	0.00	C
ATOM	3195	O	THR	B	332	-9.803	1.600	9.477	1.00	0.00	O
ATOM	3196	N	TYR	B	333	-11.504	1.016	8.080	1.00	0.00	N
ATOM	3197	H	TYR	B	333	-11.851	0.322	7.447	1.00	0.00	H
ATOM	3198	CA	TYR	B	333	-12.245	2.263	8.317	1.00	0.00	C
ATOM	3199	CB	TYR	B	333	-13.625	2.207	7.674	1.00	0.00	C
ATOM	3200	CG	TYR	B	333	-13.697	2.576	6.214	1.00	0.00	C
ATOM	3201	CD1	TYR	B	333	-13.698	1.558	5.240	1.00	0.00	C
ATOM	3202	CE1	TYR	B	333	-13.920	1.906	3.897	1.00	0.00	C
ATOM	3203	CD2	TYR	B	333	-13.835	3.935	5.875	1.00	0.00	C
ATOM	3204	CE2	TYR	B	333	-14.052	4.283	4.534	1.00	0.00	C
ATOM	3205	CZ	TYR	B	333	-14.118	3.263	3.566	1.00	0.00	C
ATOM	3206	OH	TYR	B	333	-14.395	3.611	2.259	1.00	0.00	O

ATOM	3207	HH	TYR	B	333	-14.319	2.851	1.699	1.00	0.00	H
ATOM	3208	C	TYR	B	333	-12.474	2.553	9.792	1.00	0.00	C
ATOM	3209	O	TYR	B	333	-12.144	3.604	10.327	1.00	0.00	O
ATOM	3210	N	THR	B	334	-13.057	1.529	10.431	1.00	0.00	N
ATOM	3211	H	THR	B	334	-13.213	0.677	9.935	1.00	0.00	H
ATOM	3212	CA	THR	B	334	-13.406	1.681	11.842	1.00	0.00	C
ATOM	3213	CB	THR	B	334	-14.201	0.465	12.323	1.00	0.00	C
ATOM	3214	OG1	THR	B	334	-15.146	0.017	11.335	1.00	0.00	O
ATOM	3215	HG1	THR	B	334	-14.694	-0.114	10.508	1.00	0.00	H
ATOM	3216	CG2	THR	B	334	-14.935	0.797	13.623	1.00	0.00	C
ATOM	3217	C	THR	B	334	-12.208	1.957	12.749	1.00	0.00	C
ATOM	3218	O	THR	B	334	-12.264	2.689	13.731	1.00	0.00	O
ATOM	3219	N	GLY	B	335	-11.082	1.361	12.332	1.00	0.00	N
ATOM	3220	H	GLY	B	335	-11.106	0.767	11.528	1.00	0.00	H
ATOM	3221	CA	GLY	B	335	-9.829	1.661	13.010	1.00	0.00	C
ATOM	3222	C	GLY	B	335	-9.463	3.129	12.942	1.00	0.00	C
ATOM	3223	O	GLY	B	335	-9.279	3.797	13.951	1.00	0.00	O
ATOM	3224	N	ALA	B	336	-9.386	3.604	11.692	1.00	0.00	N
ATOM	3225	H	ALA	B	336	-9.633	3.019	10.917	1.00	0.00	H
ATOM	3226	CA	ALA	B	336	-8.980	4.994	11.492	1.00	0.00	C
ATOM	3227	CB	ALA	B	336	-8.926	5.304	9.998	1.00	0.00	C
ATOM	3228	C	ALA	B	336	-9.853	6.015	12.210	1.00	0.00	C
ATOM	3229	O	ALA	B	336	-9.372	6.876	12.939	1.00	0.00	O
ATOM	3230	N	ILE	B	337	-11.173	5.835	12.016	1.00	0.00	N
ATOM	3231	H	ILE	B	337	-11.487	5.105	11.406	1.00	0.00	H
ATOM	3232	CA	ILE	B	337	-12.108	6.732	12.706	1.00	0.00	C
ATOM	3233	CB	ILE	B	337	-13.560	6.515	12.247	1.00	0.00	C
ATOM	3234	CG2	ILE	B	337	-13.648	6.667	10.731	1.00	0.00	C
ATOM	3235	CG1	ILE	B	337	-14.160	5.189	12.729	1.00	0.00	C
ATOM	3236	CD1	ILE	B	337	-15.671	5.072	12.550	1.00	0.00	C
ATOM	3237	C	ILE	B	337	-12.017	6.685	14.224	1.00	0.00	C
ATOM	3238	O	ILE	B	337	-12.270	7.645	14.939	1.00	0.00	O
ATOM	3239	N	LYS	B	338	-11.613	5.505	14.713	1.00	0.00	N
ATOM	3240	H	LYS	B	338	-11.415	4.701	14.149	1.00	0.00	H
ATOM	3241	CA	LYS	B	338	-11.367	5.550	16.138	1.00	0.00	C
ATOM	3242	CB	LYS	B	338	-12.137	4.481	16.907	1.00	0.00	C
ATOM	3243	CG	LYS	B	338	-12.473	5.053	18.288	1.00	0.00	C
ATOM	3244	CD	LYS	B	338	-12.327	4.086	19.461	1.00	0.00	C
ATOM	3245	CE	LYS	B	338	-10.906	3.658	19.852	1.00	0.00	C
ATOM	3246	NZ	LYS	B	338	-10.273	2.816	18.826	1.00	0.00	N
ATOM	3247	HZ1	LYS	B	338	-9.880	3.404	18.063	1.00	0.00	H
ATOM	3248	HZ2	LYS	B	338	-9.487	2.273	19.246	1.00	0.00	H
ATOM	3249	HZ3	LYS	B	338	-10.950	2.120	18.450	1.00	0.00	H
ATOM	3250	C	LYS	B	338	-9.907	5.590	16.524	1.00	0.00	C
ATOM	3251	O	LYS	B	338	-9.459	4.901	17.438	1.00	0.00	O
ATOM	3252	N	LEU	B	339	-9.197	6.476	15.832	1.00	0.00	N
ATOM	3253	H	LEU	B	339	-9.521	6.835	14.955	1.00	0.00	H
ATOM	3254	CA	LEU	B	339	-8.076	7.055	16.553	1.00	0.00	C
ATOM	3255	CB	LEU	B	339	-6.735	6.598	15.981	1.00	0.00	C
ATOM	3256	CG	LEU	B	339	-5.603	6.602	17.015	1.00	0.00	C
ATOM	3257	CD1	LEU	B	339	-5.999	5.889	18.312	1.00	0.00	C
ATOM	3258	CD2	LEU	B	339	-4.313	6.014	16.441	1.00	0.00	C
ATOM	3259	C	LEU	B	339	-8.225	8.557	16.669	1.00	0.00	C
ATOM	3260	O	LEU	B	339	-7.960	9.143	17.711	1.00	0.00	O
ATOM	3261	N	ASP	B	340	-8.753	9.129	15.577	1.00	0.00	N
ATOM	3262	H	ASP	B	340	-8.880	8.655	14.702	1.00	0.00	H
ATOM	3263	CA	ASP	B	340	-9.340	10.455	15.724	1.00	0.00	C
ATOM	3264	CB	ASP	B	340	-8.350	11.569	15.345	1.00	0.00	C
ATOM	3265	CG	ASP	B	340	-8.885	12.941	15.738	1.00	0.00	C

ATOM	3266	OD1	ASP	B	340	-9.607	13.068	16.725	1.00	0.00	O
ATOM	3267	OD2	ASP	B	340	-8.593	13.926	15.072	1.00	0.00	O
ATOM	3268	C	ASP	B	340	-10.610	10.554	14.907	1.00	0.00	C
ATOM	3269	O	ASP	B	340	-10.743	9.927	13.867	1.00	0.00	O
ATOM	3270	N	ASP	B	341	-11.523	11.372	15.438	1.00	0.00	N
ATOM	3271	H	ASP	B	341	-11.262	11.833	16.286	1.00	0.00	H
ATOM	3272	CA	ASP	B	341	-12.746	11.691	14.705	1.00	0.00	C
ATOM	3273	CB	ASP	B	341	-13.940	11.535	15.657	1.00	0.00	C
ATOM	3274	CG	ASP	B	341	-15.254	11.351	14.917	1.00	0.00	C
ATOM	3275	OD1	ASP	B	341	-16.213	12.067	15.201	1.00	0.00	O
ATOM	3276	OD2	ASP	B	341	-15.358	10.452	14.087	1.00	0.00	O
ATOM	3277	C	ASP	B	341	-12.693	13.087	14.089	1.00	0.00	C
ATOM	3278	O	ASP	B	341	-13.363	13.425	13.121	1.00	0.00	O
ATOM	3279	N	LYS	B	342	-11.836	13.918	14.720	1.00	0.00	N
ATOM	3280	H	LYS	B	342	-11.176	13.541	15.370	1.00	0.00	H
ATOM	3281	CA	LYS	B	342	-11.784	15.326	14.322	1.00	0.00	C
ATOM	3282	CB	LYS	B	342	-10.985	16.164	15.320	1.00	0.00	C
ATOM	3283	CG	LYS	B	342	-11.474	16.135	16.770	1.00	0.00	C
ATOM	3284	CD	LYS	B	342	-10.492	16.836	17.721	1.00	0.00	C
ATOM	3285	CE	LYS	B	342	-9.347	15.964	18.267	1.00	0.00	C
ATOM	3286	NZ	LYS	B	342	-8.540	15.361	17.198	1.00	0.00	N
ATOM	3287	HZ1	LYS	B	342	-8.020	16.024	16.598	1.00	0.00	H
ATOM	3288	HZ2	LYS	B	342	-7.909	14.617	17.553	1.00	0.00	H
ATOM	3289	HZ3	LYS	B	342	-9.156	14.804	16.559	1.00	0.00	H
ATOM	3290	C	LYS	B	342	-11.203	15.572	12.944	1.00	0.00	C
ATOM	3291	O	LYS	B	342	-11.614	16.482	12.233	1.00	0.00	O
ATOM	3292	N	ASP	B	343	-10.207	14.733	12.607	1.00	0.00	N
ATOM	3293	H	ASP	B	343	-9.867	14.061	13.268	1.00	0.00	H
ATOM	3294	CA	ASP	B	343	-9.612	14.839	11.272	1.00	0.00	C
ATOM	3295	CB	ASP	B	343	-8.556	13.732	11.069	1.00	0.00	C
ATOM	3296	CG	ASP	B	343	-8.125	13.582	9.610	1.00	0.00	C
ATOM	3297	OD1	ASP	B	343	-8.162	12.475	9.091	1.00	0.00	O
ATOM	3298	OD2	ASP	B	343	-7.827	14.576	8.957	1.00	0.00	O
ATOM	3299	C	ASP	B	343	-10.650	14.828	10.158	1.00	0.00	C
ATOM	3300	O	ASP	B	343	-11.462	13.915	10.037	1.00	0.00	O
ATOM	3301	N	PRO	B	344	-10.582	15.890	9.315	1.00	0.00	N
ATOM	3302	CD	PRO	B	344	-9.743	17.077	9.466	1.00	0.00	C
ATOM	3303	CA	PRO	B	344	-11.388	15.927	8.095	1.00	0.00	C
ATOM	3304	CB	PRO	B	344	-10.706	17.031	7.284	1.00	0.00	C
ATOM	3305	CG	PRO	B	344	-10.197	18.005	8.344	1.00	0.00	C
ATOM	3306	C	PRO	B	344	-11.530	14.608	7.350	1.00	0.00	C
ATOM	3307	O	PRO	B	344	-12.626	14.267	6.911	1.00	0.00	O
ATOM	3308	N	ASN	B	345	-10.399	13.887	7.237	1.00	0.00	N
ATOM	3309	H	ASN	B	345	-9.566	14.147	7.741	1.00	0.00	H
ATOM	3310	CA	ASN	B	345	-10.468	12.616	6.516	1.00	0.00	C
ATOM	3311	CB	ASN	B	345	-9.099	12.035	6.146	1.00	0.00	C
ATOM	3312	CG	ASN	B	345	-8.206	13.044	5.463	1.00	0.00	C
ATOM	3313	OD1	ASN	B	345	-8.256	13.253	4.259	1.00	0.00	O
ATOM	3314	ND2	ASN	B	345	-7.353	13.646	6.291	1.00	0.00	N
ATOM	3315	HD21	ASN	B	345	-6.676	14.295	5.958	1.00	0.00	H
ATOM	3316	HD22	ASN	B	345	-7.391	13.471	7.286	1.00	0.00	H
ATOM	3317	C	ASN	B	345	-11.220	11.544	7.274	1.00	0.00	C
ATOM	3318	O	ASN	B	345	-12.090	10.873	6.728	1.00	0.00	O
ATOM	3319	N	PHE	B	346	-10.863	11.390	8.561	1.00	0.00	N
ATOM	3320	H	PHE	B	346	-10.130	11.956	8.952	1.00	0.00	H
ATOM	3321	CA	PHE	B	346	-11.533	10.349	9.350	1.00	0.00	C
ATOM	3322	CB	PHE	B	346	-10.910	10.204	10.743	1.00	0.00	C
ATOM	3323	CG	PHE	B	346	-9.443	9.812	10.706	1.00	0.00	C
ATOM	3324	CD1	PHE	B	346	-8.593	10.284	11.730	1.00	0.00	C

```
ATOM   3325  CD2 PHE B 346      -8.933   8.985   9.678  1.00  0.00           C
ATOM   3326  CE1 PHE B 346      -7.225   9.950  11.717  1.00  0.00           C
ATOM   3327  CE2 PHE B 346      -7.566   8.652   9.656  1.00  0.00           C
ATOM   3328  CZ  PHE B 346      -6.724   9.145  10.674  1.00  0.00           C
ATOM   3329  C   PHE B 346     -13.037  10.541   9.440  1.00  0.00           C
ATOM   3330  O   PHE B 346     -13.833   9.629   9.247  1.00  0.00           O
ATOM   3331  N   LYS B 347     -13.386  11.822   9.622  1.00  0.00           N
ATOM   3332  H   LYS B 347     -12.673  12.451   9.940  1.00  0.00           H
ATOM   3333  CA  LYS B 347     -14.759  12.292   9.432  1.00  0.00           C
ATOM   3334  CB  LYS B 347     -14.712  13.816   9.315  1.00  0.00           C
ATOM   3335  CG  LYS B 347     -16.035  14.567   9.146  1.00  0.00           C
ATOM   3336  CD  LYS B 347     -15.778  16.017   8.712  1.00  0.00           C
ATOM   3337  CE  LYS B 347     -15.810  16.280   7.195  1.00  0.00           C
ATOM   3338  NZ  LYS B 347     -15.041  15.286   6.440  1.00  0.00           N
ATOM   3339  HZ1 LYS B 347     -14.968  15.488   5.417  1.00  0.00           H
ATOM   3340  HZ2 LYS B 347     -14.087  15.074   6.785  1.00  0.00           H
ATOM   3341  HZ3 LYS B 347     -15.571  14.390   6.383  1.00  0.00           H
ATOM   3342  C   LYS B 347     -15.462  11.675   8.225  1.00  0.00           C
ATOM   3343  O   LYS B 347     -16.520  11.065   8.324  1.00  0.00           O
ATOM   3344  N   ASP B 348     -14.821  11.849   7.058  1.00  0.00           N
ATOM   3345  H   ASP B 348     -13.891  12.216   6.999  1.00  0.00           H
ATOM   3346  CA  ASP B 348     -15.503  11.298   5.885  1.00  0.00           C
ATOM   3347  CB  ASP B 348     -15.004  11.916   4.575  1.00  0.00           C
ATOM   3348  CG  ASP B 348     -15.409  13.375   4.534  1.00  0.00           C
ATOM   3349  OD1 ASP B 348     -16.499  13.716   4.991  1.00  0.00           O
ATOM   3350  OD2 ASP B 348     -14.599  14.219   4.165  1.00  0.00           O
ATOM   3351  C   ASP B 348     -15.516   9.790   5.816  1.00  0.00           C
ATOM   3352  O   ASP B 348     -16.441   9.176   5.300  1.00  0.00           O
ATOM   3353  N   GLN B 349     -14.475   9.200   6.417  1.00  0.00           N
ATOM   3354  H   GLN B 349     -13.753   9.764   6.821  1.00  0.00           H
ATOM   3355  CA  GLN B 349     -14.460   7.743   6.524  1.00  0.00           C
ATOM   3356  CB  GLN B 349     -13.125   7.276   7.084  1.00  0.00           C
ATOM   3357  CG  GLN B 349     -12.010   7.549   6.085  1.00  0.00           C
ATOM   3358  CD  GLN B 349     -10.688   7.501   6.804  1.00  0.00           C
ATOM   3359  OE1 GLN B 349     -10.275   6.503   7.372  1.00  0.00           O
ATOM   3360  NE2 GLN B 349     -10.023   8.651   6.738  1.00  0.00           N
ATOM   3361 HE21 GLN B 349      -9.128   8.730   7.173  1.00  0.00           H
ATOM   3362 HE22 GLN B 349     -10.443   9.420   6.257  1.00  0.00           H
ATOM   3363  C   GLN B 349     -15.631   7.151   7.288  1.00  0.00           C
ATOM   3364  O   GLN B 349     -16.244   6.189   6.843  1.00  0.00           O
ATOM   3365  N   VAL B 350     -15.981   7.791   8.418  1.00  0.00           N
ATOM   3366  H   VAL B 350     -15.434   8.559   8.761  1.00  0.00           H
ATOM   3367  CA  VAL B 350     -17.213   7.314   9.056  1.00  0.00           C
ATOM   3368  CB  VAL B 350     -17.349   7.790  10.520  1.00  0.00           C
ATOM   3369  CG1 VAL B 350     -17.316   9.306  10.700  1.00  0.00           C
ATOM   3370  CG2 VAL B 350     -18.575   7.163  11.187  1.00  0.00           C
ATOM   3371  C   VAL B 350     -18.460   7.569   8.216  1.00  0.00           C
ATOM   3372  O   VAL B 350     -19.321   6.712   8.054  1.00  0.00           O
ATOM   3373  N   ILE B 351     -18.467   8.757   7.590  1.00  0.00           N
ATOM   3374  H   ILE B 351     -17.743   9.424   7.783  1.00  0.00           H
ATOM   3375  CA  ILE B 351     -19.545   9.041   6.637  1.00  0.00           C
ATOM   3376  CB  ILE B 351     -19.467  10.520   6.204  1.00  0.00           C
ATOM   3377  CG2 ILE B 351     -20.545  10.937   5.198  1.00  0.00           C
ATOM   3378  CG1 ILE B 351     -19.539  11.421   7.442  1.00  0.00           C
ATOM   3379  CD1 ILE B 351     -19.259  12.894   7.136  1.00  0.00           C
ATOM   3380  C   ILE B 351     -19.608   8.066   5.451  1.00  0.00           C
ATOM   3381  O   ILE B 351     -20.628   7.895   4.784  1.00  0.00           O
ATOM   3382  N   LEU B 352     -18.473   7.402   5.198  1.00  0.00           N
ATOM   3383  H   LEU B 352     -17.633   7.635   5.693  1.00  0.00           H
```

```
ATOM   3384  CA  LEU B 352     -18.507   6.294   4.250  1.00  0.00           C
ATOM   3385  CB  LEU B 352     -17.139   6.071   3.605  1.00  0.00           C
ATOM   3386  CG  LEU B 352     -16.682   7.259   2.755  1.00  0.00           C
ATOM   3387  CD1 LEU B 352     -15.229   7.110   2.305  1.00  0.00           C
ATOM   3388  CD2 LEU B 352     -17.629   7.526   1.583  1.00  0.00           C
ATOM   3389  C   LEU B 352     -19.042   5.013   4.856  1.00  0.00           C
ATOM   3390  O   LEU B 352     -19.876   4.338   4.265  1.00  0.00           O
ATOM   3391  N   LEU B 353     -18.577   4.724   6.081  1.00  0.00           N
ATOM   3392  H   LEU B 353     -17.887   5.314   6.502  1.00  0.00           H
ATOM   3393  CA  LEU B 353     -19.099   3.559   6.800  1.00  0.00           C
ATOM   3394  CB  LEU B 353     -18.533   3.460   8.211  1.00  0.00           C
ATOM   3395  CG  LEU B 353     -17.165   2.796   8.257  1.00  0.00           C
ATOM   3396  CD1 LEU B 353     -16.680   2.654   9.699  1.00  0.00           C
ATOM   3397  CD2 LEU B 353     -17.159   1.457   7.514  1.00  0.00           C
ATOM   3398  C   LEU B 353     -20.605   3.493   6.885  1.00  0.00           C
ATOM   3399  O   LEU B 353     -21.212   2.459   6.640  1.00  0.00           O
ATOM   3400  N   ASN B 354     -21.187   4.668   7.174  1.00  0.00           N
ATOM   3401  H   ASN B 354     -20.594   5.430   7.446  1.00  0.00           H
ATOM   3402  CA  ASN B 354     -22.651   4.765   7.224  1.00  0.00           C
ATOM   3403  CB  ASN B 354     -23.139   6.204   7.453  1.00  0.00           C
ATOM   3404  CG  ASN B 354     -22.440   6.913   8.601  1.00  0.00           C
ATOM   3405  OD1 ASN B 354     -21.971   8.032   8.450  1.00  0.00           O
ATOM   3406  ND2 ASN B 354     -22.372   6.231   9.750  1.00  0.00           N
ATOM   3407 HD21 ASN B 354     -21.875   6.649  10.512  1.00  0.00           H
ATOM   3408 HD22 ASN B 354     -22.785   5.333   9.887  1.00  0.00           H
ATOM   3409  C   ASN B 354     -23.348   4.230   5.981  1.00  0.00           C
ATOM   3410  O   ASN B 354     -24.404   3.621   6.033  1.00  0.00           O
ATOM   3411  N   LYS B 355     -22.684   4.488   4.841  1.00  0.00           N
ATOM   3412  H   LYS B 355     -21.765   4.878   4.883  1.00  0.00           H
ATOM   3413  CA  LYS B 355     -23.245   4.015   3.576  1.00  0.00           C
ATOM   3414  CB  LYS B 355     -22.637   4.784   2.401  1.00  0.00           C
ATOM   3415  CG  LYS B 355     -22.693   6.305   2.547  1.00  0.00           C
ATOM   3416  CD  LYS B 355     -22.057   7.010   1.346  1.00  0.00           C
ATOM   3417  CE  LYS B 355     -22.052   8.536   1.466  1.00  0.00           C
ATOM   3418  NZ  LYS B 355     -21.172   8.951   2.566  1.00  0.00           N
ATOM   3419  HZ1 LYS B 355     -20.196   8.643   2.388  1.00  0.00           H
ATOM   3420  HZ2 LYS B 355     -21.192   9.981   2.688  1.00  0.00           H
ATOM   3421  HZ3 LYS B 355     -21.472   8.503   3.460  1.00  0.00           H
ATOM   3422  C   LYS B 355     -23.076   2.524   3.327  1.00  0.00           C
ATOM   3423  O   LYS B 355     -23.843   1.901   2.609  1.00  0.00           O
ATOM   3424  N   HIS B 356     -22.002   1.987   3.922  1.00  0.00           N
ATOM   3425  H   HIS B 356     -21.486   2.511   4.598  1.00  0.00           H
ATOM   3426  CA  HIS B 356     -21.716   0.582   3.644  1.00  0.00           C
ATOM   3427  CB  HIS B 356     -20.205   0.327   3.591  1.00  0.00           C
ATOM   3428  CG  HIS B 356     -19.558   1.002   2.406  1.00  0.00           C
ATOM   3429  ND1 HIS B 356     -19.236   2.307   2.367  1.00  0.00           N
ATOM   3430  HD1 HIS B 356     -19.408   2.968   3.069  1.00  0.00           H
ATOM   3431  CD2 HIS B 356     -19.165   0.410   1.203  1.00  0.00           C
ATOM   3432  NE2 HIS B 356     -18.597   1.379   0.444  1.00  0.00           N
ATOM   3433  CE1 HIS B 356     -18.641   2.548   1.158  1.00  0.00           C
ATOM   3434  C   HIS B 356     -22.340  -0.362   4.653  1.00  0.00           C
ATOM   3435  O   HIS B 356     -23.162  -1.219   4.357  1.00  0.00           O
ATOM   3436  N   ILE B 357     -21.872  -0.198   5.894  1.00  0.00           N
ATOM   3437  H   ILE B 357     -21.370   0.624   6.160  1.00  0.00           H
ATOM   3438  CA  ILE B 357     -22.148  -1.288   6.821  1.00  0.00           C
ATOM   3439  CB  ILE B 357     -20.924  -1.619   7.685  1.00  0.00           C
ATOM   3440  CG2 ILE B 357     -19.796  -2.127   6.791  1.00  0.00           C
ATOM   3441  CG1 ILE B 357     -20.466  -0.464   8.574  1.00  0.00           C
ATOM   3442  CD1 ILE B 357     -19.317  -0.883   9.496  1.00  0.00           C
```

ATOM	3443	C	ILE	B	357	-23.417	-1.191	7.642	1.00	0.00	C
ATOM	3444	O	ILE	B	357	-23.560	-1.851	8.661	1.00	0.00	O
ATOM	3445	N	ASP	B	358	-24.374	-0.399	7.124	1.00	0.00	N
ATOM	3446	H	ASP	B	358	-24.203	0.138	6.300	1.00	0.00	H
ATOM	3447	CA	ASP	B	358	-25.706	-0.430	7.740	1.00	0.00	C
ATOM	3448	CB	ASP	B	358	-26.646	0.571	7.041	1.00	0.00	C
ATOM	3449	CG	ASP	B	358	-28.063	0.447	7.581	1.00	0.00	C
ATOM	3450	OD1	ASP	B	358	-28.321	0.824	8.724	1.00	0.00	O
ATOM	3451	OD2	ASP	B	358	-28.899	-0.157	6.912	1.00	0.00	O
ATOM	3452	C	ASP	B	358	-26.311	-1.835	7.807	1.00	0.00	C
ATOM	3453	O	ASP	B	358	-27.022	-2.201	8.740	1.00	0.00	O
ATOM	3454	N	ALA	B	359	-25.960	-2.636	6.784	1.00	0.00	N
ATOM	3455	H	ALA	B	359	-25.464	-2.234	6.014	1.00	0.00	H
ATOM	3456	CA	ALA	B	359	-26.371	-4.043	6.800	1.00	0.00	C
ATOM	3457	CB	ALA	B	359	-25.793	-4.777	5.589	1.00	0.00	C
ATOM	3458	C	ALA	B	359	-25.965	-4.793	8.064	1.00	0.00	C
ATOM	3459	O	ALA	B	359	-26.696	-5.601	8.626	1.00	0.00	O
ATOM	3460	N	TYR	B	360	-24.746	-4.462	8.511	1.00	0.00	N
ATOM	3461	H	TYR	B	360	-24.251	-3.690	8.114	1.00	0.00	H
ATOM	3462	CA	TYR	B	360	-24.334	-4.986	9.805	1.00	0.00	C
ATOM	3463	CB	TYR	B	360	-22.810	-4.947	9.947	1.00	0.00	C
ATOM	3464	CG	TYR	B	360	-22.123	-5.800	8.905	1.00	0.00	C
ATOM	3465	CD1	TYR	B	360	-21.561	-5.180	7.770	1.00	0.00	C
ATOM	3466	CE1	TYR	B	360	-20.809	-5.953	6.868	1.00	0.00	C
ATOM	3467	CD2	TYR	B	360	-22.030	-7.190	9.115	1.00	0.00	C
ATOM	3468	CE2	TYR	B	360	-21.285	-7.962	8.211	1.00	0.00	C
ATOM	3469	CZ	TYR	B	360	-20.641	-7.328	7.127	1.00	0.00	C
ATOM	3470	OH	TYR	B	360	-19.800	-8.074	6.320	1.00	0.00	O
ATOM	3471	HH	TYR	B	360	-19.899	-8.992	6.567	1.00	0.00	H
ATOM	3472	C	TYR	B	360	-24.970	-4.194	10.929	1.00	0.00	C
ATOM	3473	O	TYR	B	360	-24.407	-3.239	11.443	1.00	0.00	O
ATOM	3474	N	LYS	B	361	-26.172	-4.648	11.318	1.00	0.00	N
ATOM	3475	H	LYS	B	361	-26.587	-5.362	10.751	1.00	0.00	H
ATOM	3476	CA	LYS	B	361	-26.936	-3.894	12.321	1.00	0.00	C
ATOM	3477	CB	LYS	B	361	-28.328	-4.499	12.525	1.00	0.00	C
ATOM	3478	CG	LYS	B	361	-29.240	-4.277	11.317	1.00	0.00	C
ATOM	3479	CD	LYS	B	361	-29.339	-2.792	10.962	1.00	0.00	C
ATOM	3480	CE	LYS	B	361	-30.138	-2.520	9.690	1.00	0.00	C
ATOM	3481	NZ	LYS	B	361	-29.552	-1.348	9.041	1.00	0.00	N
ATOM	3482	HZ1	LYS	B	361	-29.592	-0.439	9.545	1.00	0.00	H
ATOM	3483	HZ2	LYS	B	361	-28.531	-1.478	8.892	1.00	0.00	H
ATOM	3484	HZ3	LYS	B	361	-29.887	-1.140	8.075	1.00	0.00	H
ATOM	3485	C	LYS	B	361	-26.299	-3.587	13.673	1.00	0.00	C
ATOM	3486	O	LYS	B	361	-26.827	-2.812	14.455	1.00	0.00	O
ATOM	3487	N	THR	B	362	-25.143	-4.204	13.923	1.00	0.00	N
ATOM	3488	H	THR	B	362	-24.704	-4.795	13.248	1.00	0.00	H
ATOM	3489	CA	THR	B	362	-24.363	-3.864	15.110	1.00	0.00	C
ATOM	3490	CB	THR	B	362	-23.318	-4.960	15.240	1.00	0.00	C
ATOM	3491	OG1	THR	B	362	-22.839	-5.304	13.931	1.00	0.00	O
ATOM	3492	HG1	THR	B	362	-21.896	-5.145	13.960	1.00	0.00	H
ATOM	3493	CG2	THR	B	362	-23.900	-6.199	15.920	1.00	0.00	C
ATOM	3494	C	THR	B	362	-23.715	-2.479	15.066	1.00	0.00	C
ATOM	3495	O	THR	B	362	-23.511	-1.800	16.072	1.00	0.00	O
ATOM	3496	N	PHE	B	363	-23.398	-2.083	13.826	1.00	0.00	N
ATOM	3497	H	PHE	B	363	-23.605	-2.669	13.042	1.00	0.00	H
ATOM	3498	CA	PHE	B	363	-22.838	-0.752	13.624	1.00	0.00	C
ATOM	3499	CB	PHE	B	363	-22.170	-0.653	12.243	1.00	0.00	C
ATOM	3500	CG	PHE	B	363	-21.353	0.611	12.106	1.00	0.00	C
ATOM	3501	CD1	PHE	B	363	-20.152	0.753	12.836	1.00	0.00	C

ATOM	3502	CD2	PHE	B	363	-21.799	1.621	11.224	1.00	0.00	C
ATOM	3503	CE1	PHE	B	363	-19.366	1.908	12.655	1.00	0.00	C
ATOM	3504	CE2	PHE	B	363	-21.013	2.773	11.040	1.00	0.00	C
ATOM	3505	CZ	PHE	B	363	-19.798	2.897	11.747	1.00	0.00	C
ATOM	3506	C	PHE	B	363	-23.761	0.415	13.979	1.00	0.00	C
ATOM	3507	O	PHE	B	363	-23.392	1.200	14.842	1.00	0.00	O
ATOM	3508	N	PRO	B	364	-24.981	0.525	13.368	1.00	0.00	N
ATOM	3509	CD	PRO	B	364	-25.580	-0.291	12.310	1.00	0.00	C
ATOM	3510	CA	PRO	B	364	-25.875	1.627	13.757	1.00	0.00	C
ATOM	3511	CB	PRO	B	364	-27.208	1.272	13.077	1.00	0.00	C
ATOM	3512	CG	PRO	B	364	-26.784	0.506	11.827	1.00	0.00	C
ATOM	3513	C	PRO	B	364	-25.944	2.065	15.231	1.00	0.00	C
ATOM	3514	O	PRO	B	364	-25.688	3.228	15.506	1.00	0.00	O
ATOM	3515	N	PRO	B	365	-26.245	1.158	16.207	1.00	0.00	N
ATOM	3516	CD	PRO	B	365	-26.602	-0.256	16.116	1.00	0.00	C
ATOM	3517	CA	PRO	B	365	-26.249	1.631	17.600	1.00	0.00	C
ATOM	3518	CB	PRO	B	365	-26.884	0.460	18.358	1.00	0.00	C
ATOM	3519	CG	PRO	B	365	-26.520	-0.776	17.544	1.00	0.00	C
ATOM	3520	C	PRO	B	365	-24.885	2.037	18.157	1.00	0.00	C
ATOM	3521	O	PRO	B	365	-24.789	2.779	19.125	1.00	0.00	O
ATOM	3522	N	THR	B	366	-23.821	1.523	17.522	1.00	0.00	N
ATOM	3523	H	THR	B	366	-23.921	0.982	16.689	1.00	0.00	H
ATOM	3524	CA	THR	B	366	-22.525	2.048	17.940	1.00	0.00	C
ATOM	3525	CB	THR	B	366	-21.431	0.973	17.883	1.00	0.00	C
ATOM	3526	OG1	THR	B	366	-21.358	0.321	16.604	1.00	0.00	O
ATOM	3527	HG1	THR	B	366	-22.229	-0.008	16.396	1.00	0.00	H
ATOM	3528	CG2	THR	B	366	-21.648	-0.057	18.990	1.00	0.00	C
ATOM	3529	C	THR	B	366	-22.107	3.339	17.254	1.00	0.00	C
ATOM	3530	O	THR	B	366	-21.130	3.983	17.624	1.00	0.00	O
ATOM	3531	N	GLU	B	367	-22.909	3.750	16.257	1.00	0.00	N
ATOM	3532	H	GLU	B	367	-23.676	3.215	15.902	1.00	0.00	H
ATOM	3533	CA	GLU	B	367	-22.598	5.088	15.769	1.00	0.00	C
ATOM	3534	CB	GLU	B	367	-23.111	5.416	14.359	1.00	0.00	C
ATOM	3535	CG	GLU	B	367	-21.997	5.386	13.299	1.00	0.00	C
ATOM	3536	CD	GLU	B	367	-20.691	5.975	13.821	1.00	0.00	C
ATOM	3537	OE1	GLU	B	367	-20.651	7.115	14.276	1.00	0.00	O
ATOM	3538	OE2	GLU	B	367	-19.700	5.257	13.870	1.00	0.00	O
ATOM	3539	C	GLU	B	367	-22.742	6.265	16.725	1.00	0.00	C
ATOM	3540	O	GLU	B	367	-21.727	6.885	17.027	1.00	0.00	O
ATOM	3541	N	PRO	B	368	-23.978	6.567	17.231	1.00	0.00	N
ATOM	3542	CD	PRO	B	368	-25.222	5.805	17.129	1.00	0.00	C
ATOM	3543	CA	PRO	B	368	-24.180	7.799	18.006	1.00	0.00	C
ATOM	3544	CB	PRO	B	368	-25.510	7.548	18.719	1.00	0.00	C
ATOM	3545	CG	PRO	B	368	-26.297	6.708	17.720	1.00	0.00	C
ATOM	3546	C	PRO	B	368	-23.056	8.186	18.945	1.00	0.00	C
ATOM	3547	O	PRO	B	368	-22.753	7.533	19.936	1.00	0.00	O
ATOM	3548	N	LYS	B	369	-22.430	9.315	18.584	1.00	0.00	N
ATOM	3549	H	LYS	B	369	-22.735	9.794	17.763	1.00	0.00	H
ATOM	3550	CA	LYS	B	369	-21.154	9.601	19.238	1.00	0.00	C
ATOM	3551	CB	LYS	B	369	-20.297	10.522	18.362	1.00	0.00	C
ATOM	3552	CG	LYS	B	369	-20.202	9.979	16.930	1.00	0.00	C
ATOM	3553	CD	LYS	B	369	-19.104	10.616	16.081	1.00	0.00	C
ATOM	3554	CE	LYS	B	369	-19.080	10.065	14.650	1.00	0.00	C
ATOM	3555	NZ	LYS	B	369	-17.870	10.533	13.970	1.00	0.00	N
ATOM	3556	HZ1	LYS	B	369	-17.890	10.444	12.941	1.00	0.00	H
ATOM	3557	HZ2	LYS	B	369	-17.593	11.505	14.233	1.00	0.00	H
ATOM	3558	HZ3	LYS	B	369	-17.026	10.014	14.299	1.00	0.00	H
ATOM	3559	C	LYS	B	369	-21.200	10.044	20.696	1.00	0.00	C
ATOM	3560	O	LYS	B	369	-20.185	10.364	21.311	1.00	0.00	O

```
ATOM   3561  N   LYS B 370     -22.425  10.021  21.246  1.00  0.00           N
ATOM   3562  H   LYS B 370     -23.199   9.759  20.675  1.00  0.00           H
ATOM   3563  CA  LYS B 370     -22.528   9.944  22.700  1.00  0.00           C
ATOM   3564  CB  LYS B 370     -23.870  10.515  23.174  1.00  0.00           C
ATOM   3565  CG  LYS B 370     -24.184  11.925  22.667  1.00  0.00           C
ATOM   3566  CD  LYS B 370     -25.587  12.380  23.080  1.00  0.00           C
ATOM   3567  CE  LYS B 370     -25.953  13.768  22.546  1.00  0.00           C
ATOM   3568  NZ  LYS B 370     -27.326  14.108  22.949  1.00  0.00           N
ATOM   3569  HZ1 LYS B 370     -27.988  13.402  22.569  1.00  0.00           H
ATOM   3570  HZ2 LYS B 370     -27.395  14.122  23.987  1.00  0.00           H
ATOM   3571  HZ3 LYS B 370     -27.576  15.047  22.577  1.00  0.00           H
ATOM   3572  C   LYS B 370     -22.390   8.492  23.141  1.00  0.00           C
ATOM   3573  O   LYS B 370     -21.461   8.098  23.837  1.00  0.00           O
ATOM   3574  N   ASP B 371     -23.364   7.715  22.650  1.00  0.00           N
ATOM   3575  H   ASP B 371     -24.112   8.091  22.106  1.00  0.00           H
ATOM   3576  CA  ASP B 371     -23.517   6.307  23.002  1.00  0.00           C
ATOM   3577  CB  ASP B 371     -24.776   5.745  22.337  1.00  0.00           C
ATOM   3578  CG  ASP B 371     -25.996   6.248  23.090  1.00  0.00           C
ATOM   3579  OD1 ASP B 371     -26.150   7.457  23.283  1.00  0.00           O
ATOM   3580  OD2 ASP B 371     -26.770   5.427  23.575  1.00  0.00           O
ATOM   3581  C   ASP B 371     -22.322   5.397  22.818  1.00  0.00           C
ATOM   3582  O   ASP B 371     -22.156   4.445  23.571  1.00  0.00           O
ATOM   3583  N   LYS B 372     -21.440   5.758  21.864  1.00  0.00           N
ATOM   3584  H   LYS B 372     -21.742   6.425  21.180  1.00  0.00           H
ATOM   3585  CA  LYS B 372     -20.155   5.042  21.784  1.00  0.00           C
ATOM   3586  CB  LYS B 372     -19.132   5.731  20.875  1.00  0.00           C
ATOM   3587  CG  LYS B 372     -19.523   5.975  19.422  1.00  0.00           C
ATOM   3588  CD  LYS B 372     -18.271   6.195  18.566  1.00  0.00           C
ATOM   3589  CE  LYS B 372     -18.537   6.575  17.106  1.00  0.00           C
ATOM   3590  NZ  LYS B 372     -19.421   5.608  16.454  1.00  0.00           N
ATOM   3591  HZ1 LYS B 372     -18.992   4.666  16.358  1.00  0.00           H
ATOM   3592  HZ2 LYS B 372     -20.317   5.497  16.970  1.00  0.00           H
ATOM   3593  HZ3 LYS B 372     -19.657   5.948  15.493  1.00  0.00           H
ATOM   3594  C   LYS B 372     -19.457   4.856  23.126  1.00  0.00           C
ATOM   3595  O   LYS B 372     -18.939   3.803  23.475  1.00  0.00           O
ATOM   3596  N   LYS B 373     -19.476   5.966  23.881  1.00  0.00           N
ATOM   3597  H   LYS B 373     -20.039   6.745  23.602  1.00  0.00           H
ATOM   3598  CA  LYS B 373     -18.802   5.924  25.174  1.00  0.00           C
ATOM   3599  CB  LYS B 373     -18.576   7.338  25.716  1.00  0.00           C
ATOM   3600  CG  LYS B 373     -17.623   8.167  24.848  1.00  0.00           C
ATOM   3601  CD  LYS B 373     -18.300   9.324  24.108  1.00  0.00           C
ATOM   3602  CE  LYS B 373     -17.328  10.084  23.201  1.00  0.00           C
ATOM   3603  NZ  LYS B 373     -18.016  11.224  22.580  1.00  0.00           N
ATOM   3604  HZ1 LYS B 373     -18.908  10.889  22.162  1.00  0.00           H
ATOM   3605  HZ2 LYS B 373     -18.231  11.950  23.290  1.00  0.00           H
ATOM   3606  HZ3 LYS B 373     -17.432  11.631  21.823  1.00  0.00           H
ATOM   3607  C   LYS B 373     -19.468   5.034  26.209  1.00  0.00           C
ATOM   3608  O   LYS B 373     -18.811   4.499  27.091  1.00  0.00           O
ATOM   3609  N   LYS B 374     -20.795   4.889  26.052  1.00  0.00           N
ATOM   3610  H   LYS B 374     -21.248   5.267  25.246  1.00  0.00           H
ATOM   3611  CA  LYS B 374     -21.492   3.921  26.901  1.00  0.00           C
ATOM   3612  CB  LYS B 374     -23.007   4.046  26.748  1.00  0.00           C
ATOM   3613  CG  LYS B 374     -23.621   5.415  27.030  1.00  0.00           C
ATOM   3614  CD  LYS B 374     -25.063   5.402  26.525  1.00  0.00           C
ATOM   3615  CE  LYS B 374     -25.824   6.719  26.676  1.00  0.00           C
ATOM   3616  NZ  LYS B 374     -26.962   6.693  25.748  1.00  0.00           N
ATOM   3617  HZ1 LYS B 374     -26.741   7.257  24.897  1.00  0.00           H
ATOM   3618  HZ2 LYS B 374     -27.057   5.748  25.312  1.00  0.00           H
ATOM   3619  HZ3 LYS B 374     -27.863   6.996  26.152  1.00  0.00           H
```

ATOM	3620	C	LYS	B	374	-21.083	2.503	26.548	1.00	0.00	C
ATOM	3621	O	LYS	B	374	-20.616	1.736	27.376	1.00	0.00	O
ATOM	3622	N	LYS	B	375	-21.225	2.218	25.240	1.00	0.00	N
ATOM	3623	H	LYS	B	375	-21.610	2.923	24.645	1.00	0.00	H
ATOM	3624	CA	LYS	B	375	-20.886	0.893	24.709	1.00	0.00	C
ATOM	3625	CB	LYS	B	375	-21.051	0.911	23.189	1.00	0.00	C
ATOM	3626	CG	LYS	B	375	-22.516	1.071	22.786	1.00	0.00	C
ATOM	3627	CD	LYS	B	375	-23.298	-0.239	22.897	1.00	0.00	C
ATOM	3628	CE	LYS	B	375	-24.811	-0.017	22.943	1.00	0.00	C
ATOM	3629	NZ	LYS	B	375	-25.189	0.392	24.298	1.00	0.00	N
ATOM	3630	HZ1	LYS	B	375	-25.186	-0.435	24.935	1.00	0.00	H
ATOM	3631	HZ2	LYS	B	375	-24.452	0.978	24.750	1.00	0.00	H
ATOM	3632	HZ3	LYS	B	375	-26.102	0.869	24.368	1.00	0.00	H
ATOM	3633	C	LYS	B	375	-19.508	0.398	25.112	1.00	0.00	C
ATOM	3634	O	LYS	B	375	-19.291	-0.750	25.492	1.00	0.00	O
ATOM	3635	N	ALA	B	376	-18.577	1.363	25.065	1.00	0.00	N
ATOM	3636	H	ALA	B	376	-18.817	2.244	24.653	1.00	0.00	H
ATOM	3637	CA	ALA	B	376	-17.239	1.096	25.582	1.00	0.00	C
ATOM	3638	CB	ALA	B	376	-16.400	2.375	25.547	1.00	0.00	C
ATOM	3639	C	ALA	B	376	-17.227	0.504	26.984	1.00	0.00	C
ATOM	3640	O	ALA	B	376	-16.605	-0.522	27.247	1.00	0.00	O
ATOM	3641	N	ASP	B	377	-17.963	1.186	27.878	1.00	0.00	N
ATOM	3642	H	ASP	B	377	-18.525	1.972	27.612	1.00	0.00	H
ATOM	3643	CA	ASP	B	377	-18.054	0.662	29.240	1.00	0.00	C
ATOM	3644	CB	ASP	B	377	-18.761	1.616	30.208	1.00	0.00	C
ATOM	3645	CG	ASP	B	377	-18.163	3.010	30.237	1.00	0.00	C
ATOM	3646	OD1	ASP	B	377	-16.948	3.189	30.255	1.00	0.00	O
ATOM	3647	OD2	ASP	B	377	-18.928	3.959	30.272	1.00	0.00	O
ATOM	3648	C	ASP	B	377	-18.736	-0.689	29.305	1.00	0.00	C
ATOM	3649	O	ASP	B	377	-18.208	-1.640	29.870	1.00	0.00	O
ATOM	3650	N	GLU	B	378	-19.916	-0.736	28.661	1.00	0.00	N
ATOM	3651	H	GLU	B	378	-20.248	0.111	28.244	1.00	0.00	H
ATOM	3652	CA	GLU	B	378	-20.743	-1.946	28.611	1.00	0.00	C
ATOM	3653	CB	GLU	B	378	-21.853	-1.768	27.570	1.00	0.00	C
ATOM	3654	CG	GLU	B	378	-22.840	-0.653	27.938	1.00	0.00	C
ATOM	3655	CD	GLU	B	378	-23.748	-0.326	26.764	1.00	0.00	C
ATOM	3656	OE1	GLU	B	378	-24.350	-1.229	26.189	1.00	0.00	O
ATOM	3657	OE2	GLU	B	378	-23.862	0.843	26.402	1.00	0.00	O
ATOM	3658	C	GLU	B	378	-19.960	-3.227	28.363	1.00	0.00	C
ATOM	3659	O	GLU	B	378	-20.050	-4.203	29.101	1.00	0.00	O
ATOM	3660	N	THR	B	379	-19.115	-3.139	27.320	1.00	0.00	N
ATOM	3661	H	THR	B	379	-19.200	-2.327	26.740	1.00	0.00	H
ATOM	3662	CA	THR	B	379	-18.187	-4.221	26.971	1.00	0.00	C
ATOM	3663	CB	THR	B	379	-17.077	-3.648	26.081	1.00	0.00	C
ATOM	3664	OG1	THR	B	379	-17.652	-2.909	24.999	1.00	0.00	O
ATOM	3665	HG1	THR	B	379	-18.005	-2.095	25.346	1.00	0.00	H
ATOM	3666	CG2	THR	B	379	-16.131	-4.724	25.536	1.00	0.00	C
ATOM	3667	C	THR	B	379	-17.588	-5.016	28.135	1.00	0.00	C
ATOM	3668	O	THR	B	379	-17.585	-6.246	28.158	1.00	0.00	O
ATOM	3669	N	GLN	B	380	-17.073	-4.251	29.107	1.00	0.00	N
ATOM	3670	H	GLN	B	380	-17.240	-3.262	29.106	1.00	0.00	H
ATOM	3671	CA	GLN	B	380	-16.428	-4.901	30.246	1.00	0.00	C
ATOM	3672	CB	GLN	B	380	-14.894	-4.867	30.112	1.00	0.00	C
ATOM	3673	CG	GLN	B	380	-14.264	-3.607	29.496	1.00	0.00	C
ATOM	3674	CD	GLN	B	380	-14.645	-2.377	30.285	1.00	0.00	C
ATOM	3675	OE1	GLN	B	380	-14.326	-2.210	31.454	1.00	0.00	O
ATOM	3676	NE2	GLN	B	380	-15.417	-1.541	29.600	1.00	0.00	N
ATOM	3677	HE21	GLN	B	380	-15.885	-0.801	30.076	1.00	0.00	H
ATOM	3678	HE22	GLN	B	380	-15.601	-1.603	28.617	1.00	0.00	H

ATOM	3679	C	GLN	B	380	-16.905	-4.351	31.579	1.00	0.00	C
ATOM	3680	O	GLN	B	380	-16.166	-4.252	32.558	1.00	0.00	O
ATOM	3681	N	ALA	B	381	-18.193	-3.983	31.544	1.00	0.00	N
ATOM	3682	H	ALA	B	381	-18.730	-4.119	30.709	1.00	0.00	H
ATOM	3683	CA	ALA	B	381	-18.832	-3.438	32.734	1.00	0.00	C
ATOM	3684	CB	ALA	B	381	-20.131	-2.724	32.345	1.00	0.00	C
ATOM	3685	C	ALA	B	381	-19.125	-4.512	33.763	1.00	0.00	C
ATOM	3686	O	ALA	B	381	-18.729	-5.664	33.628	1.00	0.00	O
ATOM	3687	N	LEU	B	382	-19.845	-4.087	34.811	1.00	0.00	N
ATOM	3688	H	LEU	B	382	-20.164	-3.143	34.875	1.00	0.00	H
ATOM	3689	CA	LEU	B	382	-20.221	-5.072	35.818	1.00	0.00	C
ATOM	3690	CB	LEU	B	382	-19.854	-4.564	37.216	1.00	0.00	C
ATOM	3691	CG	LEU	B	382	-18.388	-4.167	37.411	1.00	0.00	C
ATOM	3692	CD1	LEU	B	382	-18.169	-3.504	38.771	1.00	0.00	C
ATOM	3693	CD2	LEU	B	382	-17.434	-5.340	37.196	1.00	0.00	C
ATOM	3694	C	LEU	B	382	-21.704	-5.402	35.779	1.00	0.00	C
ATOM	3695	O	LEU	B	382	-22.524	-4.674	36.327	1.00	0.00	O
ATOM	3696	N	PRO	B	383	-22.047	-6.542	35.131	1.00	0.00	N
ATOM	3697	CD	PRO	B	383	-21.196	-7.432	34.348	1.00	0.00	C
ATOM	3698	CA	PRO	B	383	-23.442	-6.983	35.195	1.00	0.00	C
ATOM	3699	CB	PRO	B	383	-23.488	-8.107	34.153	1.00	0.00	C
ATOM	3700	CG	PRO	B	383	-22.065	-8.655	34.076	1.00	0.00	C
ATOM	3701	C	PRO	B	383	-23.815	-7.406	36.607	1.00	0.00	C
ATOM	3702	O	PRO	B	383	-23.201	-8.278	37.223	1.00	0.00	O
ATOM	3703	N	GLN	B	384	-24.823	-6.679	37.100	1.00	0.00	N
ATOM	3704	H	GLN	B	384	-25.299	-6.047	36.492	1.00	0.00	H
ATOM	3705	CA	GLN	B	384	-25.244	-6.866	38.484	1.00	0.00	C
ATOM	3706	CB	GLN	B	384	-25.862	-5.567	39.010	1.00	0.00	C
ATOM	3707	CG	GLN	B	384	-25.013	-4.316	38.746	1.00	0.00	C
ATOM	3708	CD	GLN	B	384	-23.690	-4.375	39.488	1.00	0.00	C
ATOM	3709	OE1	GLN	B	384	-23.539	-5.015	40.520	1.00	0.00	O
ATOM	3710	NE2	GLN	B	384	-22.743	-3.612	38.945	1.00	0.00	N
ATOM	3711	HE21	GLN	B	384	-21.938	-3.331	39.470	1.00	0.00	H
ATOM	3712	HE22	GLN	B	384	-22.840	-3.316	37.994	1.00	0.00	H
ATOM	3713	C	GLN	B	384	-26.199	-8.034	38.627	1.00	0.00	C
ATOM	3714	O	GLN	B	384	-26.590	-8.657	37.645	1.00	0.00	O
ATOM	3715	N	ARG	B	385	-26.557	-8.316	39.887	1.00	0.00	N
ATOM	3716	H	ARG	B	385	-26.326	-7.695	40.635	1.00	0.00	H
ATOM	3717	CA	ARG	B	385	-27.413	-9.483	40.097	1.00	0.00	C
ATOM	3718	CB	ARG	B	385	-27.465	-9.870	41.574	1.00	0.00	C
ATOM	3719	CG	ARG	B	385	-26.082	-9.970	42.217	1.00	0.00	C
ATOM	3720	CD	ARG	B	385	-26.165	-10.462	43.659	1.00	0.00	C
ATOM	3721	NE	ARG	B	385	-24.884	-10.307	44.338	1.00	0.00	N
ATOM	3722	HE	ARG	B	385	-24.562	-9.358	44.430	1.00	0.00	H
ATOM	3723	CZ	ARG	B	385	-24.196	-11.369	44.794	1.00	0.00	C
ATOM	3724	NH1	ARG	B	385	-24.766	-12.573	44.836	1.00	0.00	N
ATOM	3725	HH11	ARG	B	385	-25.757	-12.653	44.681	1.00	0.00	H
ATOM	3726	HH12	ARG	B	385	-24.257	-13.411	45.032	1.00	0.00	H
ATOM	3727	NH2	ARG	B	385	-22.944	-11.192	45.208	1.00	0.00	N
ATOM	3728	HH21	ARG	B	385	-22.547	-10.272	45.190	1.00	0.00	H
ATOM	3729	HH22	ARG	B	385	-22.375	-11.948	45.535	1.00	0.00	H
ATOM	3730	C	ARG	B	385	-28.818	-9.304	39.555	1.00	0.00	C
ATOM	3731	O	ARG	B	385	-29.534	-8.366	39.880	1.00	0.00	O
ATOM	3732	N	GLN	B	386	-29.187	-10.252	38.689	1.00	0.00	N
ATOM	3733	H	GLN	B	386	-28.592	-11.029	38.490	1.00	0.00	H
ATOM	3734	CA	GLN	B	386	-30.532	-10.135	38.139	1.00	0.00	C
ATOM	3735	CB	GLN	B	386	-30.608	-10.766	36.743	1.00	0.00	C
ATOM	3736	CG	GLN	B	386	-29.579	-10.232	35.736	1.00	0.00	C
ATOM	3737	CD	GLN	B	386	-29.796	-8.755	35.464	1.00	0.00	C

ATOM	3738	OE1	GLN	B	386	-30.744	-8.333	34.806	1.00	0.00	O
ATOM	3739	NE2	GLN	B	386	-28.865	-7.966	36.002	1.00	0.00	N
ATOM	3740	HE21	GLN	B	386	-28.914	-6.975	35.915	1.00	0.00	H
ATOM	3741	HE22	GLN	B	386	-28.100	-8.371	36.508	1.00	0.00	H
ATOM	3742	C	GLN	B	386	-31.600	-10.702	39.055	1.00	0.00	C
ATOM	3743	O	GLN	B	386	-32.042	-11.834	38.907	1.00	0.00	O
ATOM	3744	N	LYS	B	387	-32.033	-9.844	39.999	1.00	0.00	N
ATOM	3745	H	LYS	B	387	-31.533	-8.984	40.127	1.00	0.00	H
ATOM	3746	CA	LYS	B	387	-33.130	-10.270	40.882	1.00	0.00	C
ATOM	3747	CB	LYS	B	387	-33.530	-9.190	41.882	1.00	0.00	C
ATOM	3748	CG	LYS	B	387	-32.961	-9.475	43.270	1.00	0.00	C
ATOM	3749	CD	LYS	B	387	-33.825	-8.856	44.367	1.00	0.00	C
ATOM	3750	CE	LYS	B	387	-33.287	-9.145	45.766	1.00	0.00	C
ATOM	3751	NZ	LYS	B	387	-34.304	-8.793	46.759	1.00	0.00	N
ATOM	3752	HZ1	LYS	B	387	-34.860	-7.944	46.524	1.00	0.00	H
ATOM	3753	HZ2	LYS	B	387	-35.071	-9.497	46.772	1.00	0.00	H
ATOM	3754	HZ3	LYS	B	387	-33.947	-8.699	47.735	1.00	0.00	H
ATOM	3755	C	LYS	B	387	-34.379	-10.786	40.183	1.00	0.00	C
ATOM	3756	O	LYS	B	387	-35.095	-11.641	40.687	1.00	0.00	O
ATOM	3757	N	LYS	B	388	-34.542	-10.265	38.951	1.00	0.00	N
ATOM	3758	H	LYS	B	388	-33.980	-9.472	38.736	1.00	0.00	H
ATOM	3759	CA	LYS	B	388	-35.494	-10.797	37.969	1.00	0.00	C
ATOM	3760	CB	LYS	B	388	-35.016	-10.492	36.552	1.00	0.00	C
ATOM	3761	CG	LYS	B	388	-34.815	-9.014	36.231	1.00	0.00	C
ATOM	3762	CD	LYS	B	388	-34.076	-8.875	34.903	1.00	0.00	C
ATOM	3763	CE	LYS	B	388	-33.804	-7.429	34.499	1.00	0.00	C
ATOM	3764	NZ	LYS	B	388	-32.815	-7.441	33.415	1.00	0.00	N
ATOM	3765	HZ1	LYS	B	388	-31.939	-7.868	33.786	1.00	0.00	H
ATOM	3766	HZ2	LYS	B	388	-33.157	-8.018	32.622	1.00	0.00	H
ATOM	3767	HZ3	LYS	B	388	-32.607	-6.474	33.100	1.00	0.00	H
ATOM	3768	C	LYS	B	388	-35.774	-12.290	38.044	1.00	0.00	C
ATOM	3769	O	LYS	B	388	-36.914	-12.725	37.989	1.00	0.00	O
ATOM	3770	N	GLN	B	389	-34.684	-13.064	38.182	1.00	0.00	N
ATOM	3771	H	GLN	B	389	-33.780	-12.638	38.250	1.00	0.00	H
ATOM	3772	CA	GLN	B	389	-34.880	-14.516	38.197	1.00	0.00	C
ATOM	3773	CB	GLN	B	389	-33.541	-15.269	38.174	1.00	0.00	C
ATOM	3774	CG	GLN	B	389	-32.435	-14.672	37.289	1.00	0.00	C
ATOM	3775	CD	GLN	B	389	-32.934	-14.370	35.889	1.00	0.00	C
ATOM	3776	OE1	GLN	B	389	-33.157	-13.226	35.516	1.00	0.00	O
ATOM	3777	NE2	GLN	B	389	-33.097	-15.446	35.117	1.00	0.00	N
ATOM	3778	HE21	GLN	B	389	-33.422	-15.307	34.184	1.00	0.00	H
ATOM	3779	HE22	GLN	B	389	-32.899	-16.366	35.451	1.00	0.00	H
ATOM	3780	C	GLN	B	389	-35.763	-15.002	39.339	1.00	0.00	C
ATOM	3781	O	GLN	B	389	-36.641	-15.846	39.185	1.00	0.00	O
ATOM	3782	N	GLN	B	390	-35.522	-14.376	40.505	1.00	0.00	N
ATOM	3783	H	GLN	B	390	-34.870	-13.618	40.532	1.00	0.00	H
ATOM	3784	CA	GLN	B	390	-36.466	-14.605	41.596	1.00	0.00	C
ATOM	3785	CB	GLN	B	390	-36.015	-13.957	42.909	1.00	0.00	C
ATOM	3786	CG	GLN	B	390	-34.709	-14.500	43.495	1.00	0.00	C
ATOM	3787	CD	GLN	B	390	-34.590	-14.023	44.930	1.00	0.00	C
ATOM	3788	OE1	GLN	B	390	-33.718	-13.248	45.308	1.00	0.00	O
ATOM	3789	NE2	GLN	B	390	-35.549	-14.513	45.722	1.00	0.00	N
ATOM	3790	HE21	GLN	B	390	-35.642	-14.169	46.657	1.00	0.00	H
ATOM	3791	HE22	GLN	B	390	-36.206	-15.198	45.415	1.00	0.00	H
ATOM	3792	C	GLN	B	390	-37.859	-14.116	41.252	1.00	0.00	C
ATOM	3793	O	GLN	B	390	-38.829	-14.853	41.343	1.00	0.00	O
ATOM	3794	N	THR	B	391	-37.909	-12.847	40.819	1.00	0.00	N
ATOM	3795	H	THR	B	391	-37.073	-12.297	40.734	1.00	0.00	H
ATOM	3796	CA	THR	B	391	-39.197	-12.220	40.516	1.00	0.00	C

```
ATOM   3797  CB  THR B 391     -38.930 -10.834  39.927  1.00  0.00           C
ATOM   3798  OG1 THR B 391     -37.789 -10.245  40.571  1.00  0.00           O
ATOM   3799  HG1 THR B 391     -38.043 -10.080  41.472  1.00  0.00           H
ATOM   3800  CG2 THR B 391     -40.148  -9.912  40.019  1.00  0.00           C
ATOM   3801  C   THR B 391     -40.148 -13.030  39.633  1.00  0.00           C
ATOM   3802  O   THR B 391     -41.318 -13.221  39.944  1.00  0.00           O
ATOM   3803  N   VAL B 392     -39.577 -13.539  38.529  1.00  0.00           N
ATOM   3804  H   VAL B 392     -38.606 -13.370  38.360  1.00  0.00           H
ATOM   3805  CA  VAL B 392     -40.373 -14.374  37.629  1.00  0.00           C
ATOM   3806  CB  VAL B 392     -39.563 -14.749  36.375  1.00  0.00           C
ATOM   3807  CG1 VAL B 392     -40.359 -15.631  35.406  1.00  0.00           C
ATOM   3808  CG2 VAL B 392     -39.051 -13.493  35.665  1.00  0.00           C
ATOM   3809  C   VAL B 392     -40.918 -15.608  38.330  1.00  0.00           C
ATOM   3810  O   VAL B 392     -42.104 -15.903  38.288  1.00  0.00           O
ATOM   3811  N   THR B 393     -39.998 -16.283  39.034  1.00  0.00           N
ATOM   3812  H   THR B 393     -39.043 -15.987  39.039  1.00  0.00           H
ATOM   3813  CA  THR B 393     -40.421 -17.441  39.824  1.00  0.00           C
ATOM   3814  CB  THR B 393     -39.187 -18.064  40.486  1.00  0.00           C
ATOM   3815  OG1 THR B 393     -38.131 -18.195  39.523  1.00  0.00           O
ATOM   3816  HG1 THR B 393     -37.650 -17.371  39.505  1.00  0.00           H
ATOM   3817  CG2 THR B 393     -39.485 -19.420  41.132  1.00  0.00           C
ATOM   3818  C   THR B 393     -41.522 -17.138  40.843  1.00  0.00           C
ATOM   3819  O   THR B 393     -42.435 -17.922  41.088  1.00  0.00           O
ATOM   3820  N   LEU B 394     -41.413 -15.924  41.405  1.00  0.00           N
ATOM   3821  H   LEU B 394     -40.672 -15.310  41.134  1.00  0.00           H
ATOM   3822  CA  LEU B 394     -42.407 -15.506  42.388  1.00  0.00           C
ATOM   3823  CB  LEU B 394     -41.985 -14.222  43.110  1.00  0.00           C
ATOM   3824  CG  LEU B 394     -40.651 -14.346  43.857  1.00  0.00           C
ATOM   3825  CD1 LEU B 394     -40.239 -13.024  44.507  1.00  0.00           C
ATOM   3826  CD2 LEU B 394     -40.637 -15.510  44.851  1.00  0.00           C
ATOM   3827  C   LEU B 394     -43.820 -15.388  41.850  1.00  0.00           C
ATOM   3828  O   LEU B 394     -44.784 -15.575  42.578  1.00  0.00           O
ATOM   3829  N   LEU B 395     -43.919 -15.118  40.535  1.00  0.00           N
ATOM   3830  H   LEU B 395     -43.105 -14.987  39.966  1.00  0.00           H
ATOM   3831  CA  LEU B 395     -45.274 -15.095  39.984  1.00  0.00           C
ATOM   3832  CB  LEU B 395     -45.305 -14.485  38.571  1.00  0.00           C
ATOM   3833  CG  LEU B 395     -46.716 -14.133  38.091  1.00  0.00           C
ATOM   3834  CD1 LEU B 395     -47.352 -13.034  38.945  1.00  0.00           C
ATOM   3835  CD2 LEU B 395     -46.739 -13.789  36.602  1.00  0.00           C
ATOM   3836  C   LEU B 395     -46.084 -16.389  40.157  1.00  0.00           C
ATOM   3837  O   LEU B 395     -47.088 -16.357  40.854  1.00  0.00           O
ATOM   3838  N   PRO B 396     -45.646 -17.548  39.576  1.00  0.00           N
ATOM   3839  CD  PRO B 396     -44.573 -17.785  38.612  1.00  0.00           C
ATOM   3840  CA  PRO B 396     -46.334 -18.801  39.930  1.00  0.00           C
ATOM   3841  CB  PRO B 396     -45.586 -19.866  39.121  1.00  0.00           C
ATOM   3842  CG  PRO B 396     -44.934 -19.113  37.967  1.00  0.00           C
ATOM   3843  C   PRO B 396     -46.324 -19.110  41.421  1.00  0.00           C
ATOM   3844  O   PRO B 396     -47.278 -19.626  41.988  1.00  0.00           O
ATOM   3845  N   ALA B 397     -45.193 -18.745  42.054  1.00  0.00           N
ATOM   3846  H   ALA B 397     -44.414 -18.374  41.545  1.00  0.00           H
ATOM   3847  CA  ALA B 397     -45.097 -18.960  43.498  1.00  0.00           C
ATOM   3848  CB  ALA B 397     -43.766 -18.431  44.033  1.00  0.00           C
ATOM   3849  C   ALA B 397     -46.231 -18.372  44.327  1.00  0.00           C
ATOM   3850  O   ALA B 397     -46.640 -18.936  45.332  1.00  0.00           O
ATOM   3851  N   ALA B 398     -46.747 -17.233  43.836  1.00  0.00           N
ATOM   3852  H   ALA B 398     -46.319 -16.781  43.051  1.00  0.00           H
ATOM   3853  CA  ALA B 398     -47.904 -16.629  44.494  1.00  0.00           C
ATOM   3854  CB  ALA B 398     -48.262 -15.307  43.813  1.00  0.00           C
ATOM   3855  C   ALA B 398     -49.130 -17.527  44.557  1.00  0.00           C
```

```
ATOM   3856  O   ALA B 398     -49.825 -17.600  45.561  1.00  0.00           O
ATOM   3857  N   ASP B 399     -49.333 -18.247  43.445  1.00  0.00           N
ATOM   3858  H   ASP B 399     -48.707 -18.165  42.671  1.00  0.00           H
ATOM   3859  CA  ASP B 399     -50.420 -19.226  43.412  1.00  0.00           C
ATOM   3860  CB  ASP B 399     -50.610 -19.680  41.959  1.00  0.00           C
ATOM   3861  CG  ASP B 399     -51.823 -20.574  41.799  1.00  0.00           C
ATOM   3862  OD1 ASP B 399     -52.922 -20.052  41.638  1.00  0.00           O
ATOM   3863  OD2 ASP B 399     -51.658 -21.791  41.800  1.00  0.00           O
ATOM   3864  C   ASP B 399     -50.172 -20.411  44.337  1.00  0.00           C
ATOM   3865  O   ASP B 399     -50.993 -20.818  45.151  1.00  0.00           O
ATOM   3866  N   LEU B 400     -48.955 -20.946  44.169  1.00  0.00           N
ATOM   3867  H   LEU B 400     -48.330 -20.482  43.541  1.00  0.00           H
ATOM   3868  CA  LEU B 400     -48.643 -22.244  44.762  1.00  0.00           C
ATOM   3869  CB  LEU B 400     -47.396 -22.824  44.090  1.00  0.00           C
ATOM   3870  CG  LEU B 400     -47.495 -22.909  42.564  1.00  0.00           C
ATOM   3871  CD1 LEU B 400     -46.144 -23.243  41.932  1.00  0.00           C
ATOM   3872  CD2 LEU B 400     -48.603 -23.858  42.100  1.00  0.00           C
ATOM   3873  C   LEU B 400     -48.499 -22.308  46.278  1.00  0.00           C
ATOM   3874  O   LEU B 400     -47.393 -22.289  46.807  1.00  0.00           O
ATOM   3875  N   ASP B 401     -49.665 -22.463  46.934  1.00  0.00           N
ATOM   3876  H   ASP B 401     -50.478 -22.289  46.373  1.00  0.00           H
ATOM   3877  CA  ASP B 401     -49.828 -22.724  48.375  1.00  0.00           C
ATOM   3878  CB  ASP B 401     -50.372 -24.152  48.611  1.00  0.00           C
ATOM   3879  CG  ASP B 401     -50.470 -24.468  50.101  1.00  0.00           C
ATOM   3880  OD1 ASP B 401     -51.154 -23.762  50.834  1.00  0.00           O
ATOM   3881  OD2 ASP B 401     -49.742 -25.330  50.577  1.00  0.00           O
ATOM   3882  C   ASP B 401     -48.687 -22.398  49.341  1.00  0.00           C
ATOM   3883  O   ASP B 401     -48.759 -21.492  50.168  1.00  0.00           O
ATOM   3884  N   ASP B 402     -47.638 -23.227  49.293  1.00  0.00           N
ATOM   3885  H   ASP B 402     -47.566 -23.918  48.577  1.00  0.00           H
ATOM   3886  CA  ASP B 402     -46.619 -22.948  50.297  1.00  0.00           C
ATOM   3887  CB  ASP B 402     -45.843 -24.190  50.706  1.00  0.00           C
ATOM   3888  CG  ASP B 402     -45.550 -24.015  52.178  1.00  0.00           C
ATOM   3889  OD1 ASP B 402     -46.454 -24.249  52.978  1.00  0.00           O
ATOM   3890  OD2 ASP B 402     -44.448 -23.593  52.513  1.00  0.00           O
ATOM   3891  C   ASP B 402     -45.716 -21.787  49.963  1.00  0.00           C
ATOM   3892  O   ASP B 402     -45.370 -20.949  50.786  1.00  0.00           O
ATOM   3893  N   PHE B 403     -45.411 -21.723  48.665  1.00  0.00           N
ATOM   3894  H   PHE B 403     -45.841 -22.357  48.021  1.00  0.00           H
ATOM   3895  CA  PHE B 403     -44.699 -20.541  48.195  1.00  0.00           C
ATOM   3896  CB  PHE B 403     -44.273 -20.731  46.743  1.00  0.00           C
ATOM   3897  CG  PHE B 403     -43.470 -22.002  46.587  1.00  0.00           C
ATOM   3898  CD1 PHE B 403     -44.067 -23.119  45.962  1.00  0.00           C
ATOM   3899  CD2 PHE B 403     -42.144 -22.055  47.069  1.00  0.00           C
ATOM   3900  CE1 PHE B 403     -43.331 -24.312  45.827  1.00  0.00           C
ATOM   3901  CE2 PHE B 403     -41.407 -23.248  46.936  1.00  0.00           C
ATOM   3902  CZ  PHE B 403     -42.010 -24.365  46.319  1.00  0.00           C
ATOM   3903  C   PHE B 403     -45.495 -19.262  48.411  1.00  0.00           C
ATOM   3904  O   PHE B 403     -44.962 -18.231  48.802  1.00  0.00           O
ATOM   3905  N   SER B 404     -46.820 -19.414  48.262  1.00  0.00           N
ATOM   3906  H   SER B 404     -47.213 -20.174  47.739  1.00  0.00           H
ATOM   3907  CA  SER B 404     -47.715 -18.354  48.725  1.00  0.00           C
ATOM   3908  CB  SER B 404     -49.163 -18.774  48.495  1.00  0.00           C
ATOM   3909  OG  SER B 404     -49.220 -19.590  47.322  1.00  0.00           O
ATOM   3910  HG  SER B 404     -49.721 -19.080  46.684  1.00  0.00           H
ATOM   3911  C   SER B 404     -47.499 -17.935  50.173  1.00  0.00           C
ATOM   3912  O   SER B 404     -47.337 -16.765  50.489  1.00  0.00           O
ATOM   3913  N   LYS B 405     -47.446 -18.945  51.060  1.00  0.00           N
ATOM   3914  H   LYS B 405     -47.571 -19.888  50.745  1.00  0.00           H
```

ATOM	3915	CA	LYS	B	405	-47.160	-18.612	52.462	1.00	0.00	C
ATOM	3916	CB	LYS	B	405	-47.228	-19.842	53.370	1.00	0.00	C
ATOM	3917	CG	LYS	B	405	-48.506	-20.662	53.213	1.00	0.00	C
ATOM	3918	CD	LYS	B	405	-48.521	-21.883	54.133	1.00	0.00	C
ATOM	3919	CE	LYS	B	405	-49.496	-22.967	53.666	1.00	0.00	C
ATOM	3920	NZ	LYS	B	405	-49.033	-23.509	52.386	1.00	0.00	N
ATOM	3921	HZ1	LYS	B	405	-48.102	-23.972	52.479	1.00	0.00	H
ATOM	3922	HZ2	LYS	B	405	-49.712	-24.205	51.992	1.00	0.00	H
ATOM	3923	HZ3	LYS	B	405	-48.982	-22.764	51.665	1.00	0.00	H
ATOM	3924	C	LYS	B	405	-45.828	-17.910	52.680	1.00	0.00	C
ATOM	3925	O	LYS	B	405	-45.677	-17.018	53.506	1.00	0.00	O
ATOM	3926	N	GLN	B	406	-44.854	-18.338	51.869	1.00	0.00	N
ATOM	3927	H	GLN	B	406	-45.048	-19.084	51.229	1.00	0.00	H
ATOM	3928	CA	GLN	B	406	-43.543	-17.695	51.920	1.00	0.00	C
ATOM	3929	CB	GLN	B	406	-42.552	-18.537	51.118	1.00	0.00	C
ATOM	3930	CG	GLN	B	406	-42.437	-19.931	51.745	1.00	0.00	C
ATOM	3931	CD	GLN	B	406	-41.907	-20.932	50.740	1.00	0.00	C
ATOM	3932	OE1	GLN	B	406	-41.058	-20.643	49.908	1.00	0.00	O
ATOM	3933	NE2	GLN	B	406	-42.455	-22.145	50.852	1.00	0.00	N
ATOM	3934	HE21	GLN	B	406	-42.157	-22.884	50.257	1.00	0.00	H
ATOM	3935	HE22	GLN	B	406	-43.173	-22.357	51.526	1.00	0.00	H
ATOM	3936	C	GLN	B	406	-43.565	-16.228	51.512	1.00	0.00	C
ATOM	3937	O	GLN	B	406	-42.950	-15.369	52.130	1.00	0.00	O
ATOM	3938	N	LEU	B	407	-44.369	-15.964	50.477	1.00	0.00	N
ATOM	3939	H	LEU	B	407	-44.832	-16.709	49.993	1.00	0.00	H
ATOM	3940	CA	LEU	B	407	-44.584	-14.567	50.099	1.00	0.00	C
ATOM	3941	CB	LEU	B	407	-45.225	-14.515	48.715	1.00	0.00	C
ATOM	3942	CG	LEU	B	407	-44.243	-14.987	47.641	1.00	0.00	C
ATOM	3943	CD1	LEU	B	407	-44.931	-15.308	46.318	1.00	0.00	C
ATOM	3944	CD2	LEU	B	407	-43.100	-13.991	47.455	1.00	0.00	C
ATOM	3945	C	LEU	B	407	-45.360	-13.757	51.129	1.00	0.00	C
ATOM	3946	O	LEU	B	407	-45.088	-12.593	51.396	1.00	0.00	O
ATOM	3947	N	GLN	B	408	-46.314	-14.453	51.767	1.00	0.00	N
ATOM	3948	H	GLN	B	408	-46.519	-15.377	51.449	1.00	0.00	H
ATOM	3949	CA	GLN	B	408	-47.016	-13.857	52.908	1.00	0.00	C
ATOM	3950	CB	GLN	B	408	-48.084	-14.819	53.428	1.00	0.00	C
ATOM	3951	CG	GLN	B	408	-49.264	-14.936	52.464	1.00	0.00	C
ATOM	3952	CD	GLN	B	408	-49.993	-16.250	52.657	1.00	0.00	C
ATOM	3953	OE1	GLN	B	408	-49.896	-16.919	53.678	1.00	0.00	O
ATOM	3954	NE2	GLN	B	408	-50.733	-16.614	51.604	1.00	0.00	N
ATOM	3955	HE21	GLN	B	408	-51.243	-17.469	51.648	1.00	0.00	H
ATOM	3956	HE22	GLN	B	408	-50.792	-16.055	50.775	1.00	0.00	H
ATOM	3957	C	GLN	B	408	-46.084	-13.428	54.030	1.00	0.00	C
ATOM	3958	O	GLN	B	408	-46.251	-12.383	54.646	1.00	0.00	O
ATOM	3959	N	GLN	B	409	-45.049	-14.261	54.229	1.00	0.00	N
ATOM	3960	H	GLN	B	409	-45.017	-15.121	53.718	1.00	0.00	H
ATOM	3961	CA	GLN	B	409	-43.987	-13.879	55.164	1.00	0.00	C
ATOM	3962	CB	GLN	B	409	-42.943	-14.988	55.255	1.00	0.00	C
ATOM	3963	CG	GLN	B	409	-43.503	-16.317	55.757	1.00	0.00	C
ATOM	3964	CD	GLN	B	409	-42.594	-17.449	55.321	1.00	0.00	C
ATOM	3965	OE1	GLN	B	409	-41.414	-17.288	55.034	1.00	0.00	O
ATOM	3966	NE2	GLN	B	409	-43.214	-18.630	55.262	1.00	0.00	N
ATOM	3967	HE21	GLN	B	409	-42.695	-19.440	54.994	1.00	0.00	H
ATOM	3968	HE22	GLN	B	409	-44.188	-18.701	55.468	1.00	0.00	H
ATOM	3969	C	GLN	B	409	-43.314	-12.556	54.826	1.00	0.00	C
ATOM	3970	O	GLN	B	409	-43.022	-11.740	55.690	1.00	0.00	O
ATOM	3971	N	SER	B	410	-43.111	-12.357	53.518	1.00	0.00	N
ATOM	3972	H	SER	B	410	-43.323	-13.031	52.807	1.00	0.00	H
ATOM	3973	CA	SER	B	410	-42.583	-11.060	53.111	1.00	0.00	C

```
ATOM   3974  CB  SER B 410     -41.617 -11.266  51.949  1.00  0.00           C
ATOM   3975  OG  SER B 410     -42.178 -12.178  50.992  1.00  0.00           O
ATOM   3976  HG  SER B 410     -42.084 -11.746  50.139  1.00  0.00           H
ATOM   3977  C   SER B 410     -43.631 -10.008  52.772  1.00  0.00           C
ATOM   3978  O   SER B 410     -43.359  -9.047  52.064  1.00  0.00           O
ATOM   3979  N   MET B 411     -44.858 -10.234  53.286  1.00  0.00           N
ATOM   3980  H   MET B 411     -45.001 -11.023  53.882  1.00  0.00           H
ATOM   3981  CA  MET B 411     -45.977  -9.316  53.033  1.00  0.00           C
ATOM   3982  CB  MET B 411     -45.852  -8.059  53.902  1.00  0.00           C
ATOM   3983  CG  MET B 411     -45.794  -8.377  55.398  1.00  0.00           C
ATOM   3984  SD  MET B 411     -45.485  -6.915  56.401  1.00  0.00           S
ATOM   3985  CE  MET B 411     -43.834  -6.538  55.787  1.00  0.00           C
ATOM   3986  C   MET B 411     -46.204  -8.960  51.569  1.00  0.00           C
ATOM   3987  O   MET B 411     -46.482  -7.828  51.192  1.00  0.00           O
ATOM   3988  N   SER B 412     -46.049 -10.002  50.751  1.00  0.00           N
ATOM   3989  H   SER B 412     -45.949 -10.928  51.113  1.00  0.00           H
ATOM   3990  CA  SER B 412     -46.107  -9.767  49.318  1.00  0.00           C
ATOM   3991  CB  SER B 412     -44.727 -10.077  48.719  1.00  0.00           C
ATOM   3992  OG  SER B 412     -44.253 -11.361  49.140  1.00  0.00           O
ATOM   3993  HG  SER B 412     -44.258 -11.384  50.094  1.00  0.00           H
ATOM   3994  C   SER B 412     -47.260 -10.526  48.684  1.00  0.00           C
ATOM   3995  O   SER B 412     -47.968 -11.263  49.360  1.00  0.00           O
ATOM   3996  N   SER B 413     -47.439 -10.293  47.369  1.00  0.00           N
ATOM   3997  H   SER B 413     -46.774  -9.748  46.867  1.00  0.00           H
ATOM   3998  CA  SER B 413     -48.521 -10.980  46.656  1.00  0.00           C
ATOM   3999  CB  SER B 413     -48.558 -10.470  45.207  1.00  0.00           C
ATOM   4000  OG  SER B 413     -49.592 -11.075  44.420  1.00  0.00           O
ATOM   4001  HG  SER B 413     -50.388 -11.128  44.955  1.00  0.00           H
ATOM   4002  C   SER B 413     -48.363 -12.487  46.732  1.00  0.00           C
ATOM   4003  O   SER B 413     -47.285 -13.028  46.518  1.00  0.00           O
ATOM   4004  N   ALA B 414     -49.477 -13.120  47.096  1.00  0.00           N
ATOM   4005  H   ALA B 414     -50.353 -12.627  47.160  1.00  0.00           H
ATOM   4006  CA  ALA B 414     -49.420 -14.536  47.428  1.00  0.00           C
ATOM   4007  CB  ALA B 414     -49.133 -14.700  48.912  1.00  0.00           C
ATOM   4008  C   ALA B 414     -50.740 -15.186  47.093  1.00  0.00           C
ATOM   4009  O   ALA B 414     -51.344 -15.917  47.873  1.00  0.00           O
ATOM   4010  N   ASP B 415     -51.154 -14.803  45.885  1.00  0.00           N
ATOM   4011  H   ASP B 415     -50.553 -14.268  45.296  1.00  0.00           H
ATOM   4012  CA  ASP B 415     -52.531 -14.991  45.461  1.00  0.00           C
ATOM   4013  CB  ASP B 415     -53.181 -13.621  45.194  1.00  0.00           C
ATOM   4014  CG  ASP B 415     -52.765 -12.580  46.228  1.00  0.00           C
ATOM   4015  OD1 ASP B 415     -51.835 -11.818  45.962  1.00  0.00           O
ATOM   4016  OD2 ASP B 415     -53.352 -12.543  47.304  1.00  0.00           O
ATOM   4017  C   ASP B 415     -52.516 -15.805  44.189  1.00  0.00           C
ATOM   4018  O   ASP B 415     -51.501 -15.874  43.505  1.00  0.00           O
ATOM   4019  N   SER B 416     -53.672 -16.404  43.875  1.00  0.00           N
ATOM   4020  H   SER B 416     -54.453 -16.372  44.494  1.00  0.00           H
ATOM   4021  CA  SER B 416     -53.731 -17.151  42.620  1.00  0.00           C
ATOM   4022  CB  SER B 416     -55.062 -17.884  42.503  1.00  0.00           C
ATOM   4023  OG  SER B 416     -55.060 -18.966  43.437  1.00  0.00           O
ATOM   4024  HG  SER B 416     -54.364 -19.551  43.119  1.00  0.00           H
ATOM   4025  C   SER B 416     -53.431 -16.358  41.362  1.00  0.00           C
ATOM   4026  O   SER B 416     -54.165 -15.467  40.948  1.00  0.00           O
ATOM   4027  N   THR B 417     -52.289 -16.731  40.786  1.00  0.00           N
ATOM   4028  H   THR B 417     -51.729 -17.431  41.229  1.00  0.00           H
ATOM   4029  CA  THR B 417     -51.800 -16.028  39.611  1.00  0.00           C
ATOM   4030  CB  THR B 417     -50.503 -15.316  40.008  1.00  0.00           C
ATOM   4031  OG1 THR B 417     -49.662 -16.263  40.685  1.00  0.00           O
ATOM   4032  HG1 THR B 417     -48.899 -15.799  41.021  1.00  0.00           H
```

```
ATOM   4033  CG2 THR B 417     -50.737 -14.077  40.875  1.00  0.00           C
ATOM   4034  C   THR B 417     -51.614 -16.999  38.449  1.00  0.00           C
ATOM   4035  O   THR B 417     -52.535 -17.297  37.698  1.00  0.00           O
ATOM   4036  N   GLN B 418     -50.378 -17.509  38.325  1.00  0.00           N
ATOM   4037  H   GLN B 418     -49.703 -17.369  39.051  1.00  0.00           H
ATOM   4038  CA  GLN B 418     -50.195 -18.551  37.329  1.00  0.00           C
ATOM   4039  CB  GLN B 418     -48.860 -18.384  36.585  1.00  0.00           C
ATOM   4040  CG  GLN B 418     -48.527 -19.483  35.558  1.00  0.00           C
ATOM   4041  CD  GLN B 418     -49.650 -19.652  34.552  1.00  0.00           C
ATOM   4042  OE1 GLN B 418     -49.863 -18.833  33.670  1.00  0.00           O
ATOM   4043  NE2 GLN B 418     -50.371 -20.766  34.718  1.00  0.00           N
ATOM   4044 HE21 GLN B 418     -51.120 -20.937  34.082  1.00  0.00           H
ATOM   4045 HE22 GLN B 418     -50.202 -21.432  35.450  1.00  0.00           H
ATOM   4046  C   GLN A 418     -50.339 -19.906  37.977  1.00  0.00           C
ATOM   4047  O   GLN A 418     -49.375 -20.541  38.387  1.00  0.00           O
ATOM   4048  N   ALA A 419     -51.614 -20.312  38.023  1.00  0.00           N
ATOM   4049  H   ALA A 419     -52.329 -19.661  37.767  1.00  0.00           H
ATOM   4050  CA  ALA A 419     -51.927 -21.619  38.599  1.00  0.00           C
ATOM   4051  CB  ALA A 419     -53.419 -21.923  38.453  1.00  0.00           C
ATOM   4052  C   ALA A 419     -51.133 -22.765  38.002  1.00  0.00           C
ATOM   4053  O   ALA A 419     -50.814 -22.765  36.812  1.00  0.00           O
END
```
