# Supplementary material for: Analysing the impact of the two most common SARS-CoV-2 nucleocapsid protein variants on interactions with membrane protein in silico
Source: J Genet Eng Biotechnol. 2021 Sep 20;19:138. doi: 10.1186/s43141-021-00233-z (PMC8451389; doi:10.1186/s43141-021-00233-z)
Supplement: Supplementary file 4 — Supplementary File 2. – Docked complex between wild type N protein and M protein. [file 43141_2021_233_MOESM4_ESM.pdf]

```
ATOM      1  N   MET A   1      -0.342 -11.082  -6.050  1.00  0.00           N
ATOM      2  H   MET A   1       0.317 -11.021  -6.802  1.00  0.00           H
ATOM      3  CA  MET A   1      -1.675 -10.475  -6.225  1.00  0.00           C
ATOM      4  CB  MET A   1      -1.544  -9.001  -6.604  1.00  0.00           C
ATOM      5  CG  MET A   1      -2.419  -8.073  -5.764  1.00  0.00           C
ATOM      6  SD  MET A   1      -4.097  -8.677  -5.543  1.00  0.00           S
ATOM      7  CE  MET A   1      -4.753  -7.225  -4.711  1.00  0.00           C
ATOM      8  C   MET A   1      -2.330 -11.226  -7.340  1.00  0.00           C
ATOM      9  O   MET A   1      -2.491 -10.729  -8.454  1.00  0.00           O
ATOM     10  N   ALA A   2      -2.742 -12.472  -7.018  1.00  0.00           N
ATOM     11  H   ALA A   2      -2.546 -12.740  -6.077  1.00  0.00           H
ATOM     12  CA  ALA A   2      -3.362 -13.402  -7.927  1.00  0.00           C
ATOM     13  CB  ALA A   2      -3.527 -14.769  -7.260  1.00  0.00           C
ATOM     14  C   ALA A   2      -4.693 -12.952  -8.444  1.00  0.00           C
ATOM     15  O   ALA A   2      -4.988 -13.158  -9.622  1.00  0.00           O
ATOM     16  N   ASP A   3      -5.493 -12.283  -7.583  1.00  0.00           N
ATOM     17  H   ASP A   3      -5.219 -12.195  -6.628  1.00  0.00           H
ATOM     18  CA  ASP A   3      -6.814 -11.813  -7.911  1.00  0.00           C
ATOM     19  CB  ASP A   3      -7.466 -11.186  -6.676  1.00  0.00           C
ATOM     20  CG  ASP A   3      -7.730 -12.244  -5.629  1.00  0.00           C
ATOM     21  OD1 ASP A   3      -7.227 -12.129  -4.509  1.00  0.00           O
ATOM     22  OD2 ASP A   3      -8.450 -13.191  -5.929  1.00  0.00           O
ATOM     23  C   ASP A   3      -6.805 -10.802  -9.021  1.00  0.00           C
ATOM     24  O   ASP A   3      -7.661 -10.869  -9.902  1.00  0.00           O
ATOM     25  N   SER A   4      -5.849  -9.848  -8.994  1.00  0.00           N
ATOM     26  H   SER A   4      -5.232  -9.772  -8.213  1.00  0.00           H
ATOM     27  CA  SER A   4      -5.714  -8.846 -10.016  1.00  0.00           C
ATOM     28  CB  SER A   4      -5.146  -7.662  -9.272  1.00  0.00           C
ATOM     29  OG  SER A   4      -5.497  -7.903  -7.906  1.00  0.00           O
ATOM     30  HG  SER A   4      -5.369  -7.083  -7.446  1.00  0.00           H
ATOM     31  C   SER A   4      -4.916  -9.329 -11.201  1.00  0.00           C
ATOM     32  O   SER A   4      -5.236  -8.979 -12.338  1.00  0.00           O
ATOM     33  N   ASN A   5      -3.886 -10.185 -10.964  1.00  0.00           N
ATOM     34  H   ASN A   5      -3.695 -10.439 -10.014  1.00  0.00           H
ATOM     35  CA  ASN A   5      -3.021 -10.720 -11.997  1.00  0.00           C
ATOM     36  CB  ASN A   5      -1.876 -11.566 -11.451  1.00  0.00           C
ATOM     37  CG  ASN A   5      -1.079 -12.123 -12.617  1.00  0.00           C
ATOM     38  OD1 ASN A   5      -0.263 -11.438 -13.231  1.00  0.00           O
ATOM     39  ND2 ASN A   5      -1.315 -13.428 -12.856  1.00  0.00           N
ATOM     40 HD21 ASN A   5      -0.820 -13.931 -13.566  1.00  0.00           H
ATOM     41 HD22 ASN A   5      -1.987 -13.943 -12.321  1.00  0.00           H
ATOM     42  C   ASN A   5      -3.810 -11.557 -12.959  1.00  0.00           C
ATOM     43  O   ASN A   5      -3.569 -11.477 -14.163  1.00  0.00           O
ATOM     44  N   GLY A   6      -4.816 -12.309 -12.451  1.00  0.00           N
ATOM     45  H   GLY A   6      -4.996 -12.304 -11.464  1.00  0.00           H
ATOM     46  CA  GLY A   6      -5.652 -13.165 -13.249  1.00  0.00           C
ATOM     47  C   GLY A   6      -6.450 -12.408 -14.269  1.00  0.00           C
ATOM     48  O   GLY A   6      -6.793 -12.986 -15.299  1.00  0.00           O
ATOM     49  N   THR A   7      -6.788 -11.124 -13.985  1.00  0.00           N
ATOM     50  H   THR A   7      -6.552 -10.735 -13.096  1.00  0.00           H
ATOM     51  CA  THR A   7      -7.532 -10.313 -14.907  1.00  0.00           C
ATOM     52  CB  THR A   7      -8.342  -9.362 -14.050  1.00  0.00           C
ATOM     53  OG1 THR A   7      -7.862  -9.466 -12.705  1.00  0.00           O
ATOM     54  HG1 THR A   7      -7.110  -8.888 -12.633  1.00  0.00           H
ATOM     55  CG2 THR A   7      -9.836  -9.680 -14.109  1.00  0.00           C
ATOM     56  C   THR A   7      -6.618  -9.621 -15.882  1.00  0.00           C
ATOM     57  O   THR A   7      -6.913  -9.586 -17.074  1.00  0.00           O
ATOM     58  N   ILE A   8      -5.450  -9.124 -15.399  1.00  0.00           N
ATOM     59  H   ILE A   8      -5.255  -9.278 -14.429  1.00  0.00           H
```

```
ATOM     60  CA  ILE A   8      -4.466  -8.397 -16.184  1.00  0.00           C
ATOM     61  CB  ILE A   8      -3.411  -7.799 -15.258  1.00  0.00           C
ATOM     62  CG2 ILE A   8      -2.226  -7.200 -16.013  1.00  0.00           C
ATOM     63  CG1 ILE A   8      -4.102  -6.752 -14.385  1.00  0.00           C
ATOM     64  CD1 ILE A   8      -3.171  -6.120 -13.359  1.00  0.00           C
ATOM     65  C   ILE A   8      -3.881  -9.249 -17.285  1.00  0.00           C
ATOM     66  O   ILE A   8      -3.612  -8.738 -18.374  1.00  0.00           O
ATOM     67  N   THR A   9      -3.703 -10.561 -17.025  1.00  0.00           N
ATOM     68  H   THR A   9      -4.002 -10.952 -16.153  1.00  0.00           H
ATOM     69  CA  THR A   9      -3.192 -11.511 -17.979  1.00  0.00           C
ATOM     70  CB  THR A   9      -2.828 -12.770 -17.219  1.00  0.00           C
ATOM     71  OG1 THR A   9      -3.827 -13.001 -16.232  1.00  0.00           O
ATOM     72  HG1 THR A   9      -3.450 -12.743 -15.400  1.00  0.00           H
ATOM     73  CG2 THR A   9      -1.472 -12.633 -16.528  1.00  0.00           C
ATOM     74  C   THR A   9      -4.151 -11.760 -19.127  1.00  0.00           C
ATOM     75  O   THR A   9      -3.702 -12.039 -20.241  1.00  0.00           O
ATOM     76  N   VAL A  10      -5.484 -11.667 -18.869  1.00  0.00           N
ATOM     77  H   VAL A  10      -5.767 -11.324 -17.973  1.00  0.00           H
ATOM     78  CA  VAL A  10      -6.544 -11.892 -19.834  1.00  0.00           C
ATOM     79  CB  VAL A  10      -7.892 -11.993 -19.109  1.00  0.00           C
ATOM     80  CG1 VAL A  10      -9.098 -12.035 -20.048  1.00  0.00           C
ATOM     81  CG2 VAL A  10      -7.879 -13.219 -18.200  1.00  0.00           C
ATOM     82  C   VAL A  10      -6.552 -10.825 -20.907  1.00  0.00           C
ATOM     83  O   VAL A  10      -6.408  -9.631 -20.636  1.00  0.00           O
ATOM     84  N   GLU A  11      -6.770 -11.282 -22.166  1.00  0.00           N
ATOM     85  H   GLU A  11      -6.760 -12.278 -22.263  1.00  0.00           H
ATOM     86  CA  GLU A  11      -6.800 -10.488 -23.369  1.00  0.00           C
ATOM     87  CB  GLU A  11      -6.971 -11.334 -24.634  1.00  0.00           C
ATOM     88  CG  GLU A  11      -6.107 -12.594 -24.695  1.00  0.00           C
ATOM     89  CD  GLU A  11      -4.723 -12.281 -24.179  1.00  0.00           C
ATOM     90  OE1 GLU A  11      -4.045 -11.418 -24.727  1.00  0.00           O
ATOM     91  OE2 GLU A  11      -4.326 -12.866 -23.177  1.00  0.00           O
ATOM     92  C   GLU A  11      -7.863  -9.433 -23.367  1.00  0.00           C
ATOM     93  O   GLU A  11      -7.623  -8.332 -23.866  1.00  0.00           O
ATOM     94  N   GLU A  12      -9.041  -9.735 -22.772  1.00  0.00           N
ATOM     95  H   GLU A  12      -9.174 -10.660 -22.422  1.00  0.00           H
ATOM     96  CA  GLU A  12     -10.147  -8.818 -22.712  1.00  0.00           C
ATOM     97  CB  GLU A  12     -11.387  -9.536 -22.180  1.00  0.00           C
ATOM     98  CG  GLU A  12     -12.624  -9.261 -23.036  1.00  0.00           C
ATOM     99  CD  GLU A  12     -12.330  -9.630 -24.478  1.00  0.00           C
ATOM    100  OE1 GLU A  12     -12.750  -8.905 -25.377  1.00  0.00           O
ATOM    101  OE2 GLU A  12     -11.626 -10.609 -24.728  1.00  0.00           O
ATOM    102  C   GLU A  12      -9.777  -7.614 -21.883  1.00  0.00           C
ATOM    103  O   GLU A  12     -10.106  -6.491 -22.273  1.00  0.00           O
ATOM    104  N   LEU A  13      -9.060  -7.817 -20.744  1.00  0.00           N
ATOM    105  H   LEU A  13      -8.700  -8.724 -20.530  1.00  0.00           H
ATOM    106  CA  LEU A  13      -8.660  -6.704 -19.924  1.00  0.00           C
ATOM    107  CB  LEU A  13      -8.290  -7.106 -18.499  1.00  0.00           C
ATOM    108  CG  LEU A  13      -8.120  -5.867 -17.611  1.00  0.00           C
ATOM    109  CD1 LEU A  13      -9.398  -5.028 -17.561  1.00  0.00           C
ATOM    110  CD2 LEU A  13      -7.622  -6.209 -16.211  1.00  0.00           C
ATOM    111  C   LEU A  13      -7.530  -5.978 -20.605  1.00  0.00           C
ATOM    112  O   LEU A  13      -7.483  -4.755 -20.511  1.00  0.00           O
ATOM    113  N   LYS A  14      -6.643  -6.694 -21.357  1.00  0.00           N
ATOM    114  H   LYS A  14      -6.718  -7.693 -21.345  1.00  0.00           H
ATOM    115  CA  LYS A  14      -5.522  -6.101 -22.065  1.00  0.00           C
ATOM    116  CB  LYS A  14      -4.666  -7.115 -22.831  1.00  0.00           C
ATOM    117  CG  LYS A  14      -3.866  -8.129 -22.009  1.00  0.00           C
ATOM    118  CD  LYS A  14      -2.761  -8.730 -22.883  1.00  0.00           C
```

```
ATOM    119  CE  LYS A  14      -1.972  -9.885 -22.261  1.00  0.00           C
ATOM    120  NZ  LYS A  14      -2.748 -11.123 -22.347  1.00  0.00           N
ATOM    121  HZ1 LYS A  14      -3.600 -11.131 -21.752  1.00  0.00           H
ATOM    122  HZ2 LYS A  14      -3.108 -11.327 -23.305  1.00  0.00           H
ATOM    123  HZ3 LYS A  14      -2.227 -11.973 -22.073  1.00  0.00           H
ATOM    124  C   LYS A  14      -5.982  -5.048 -23.034  1.00  0.00           C
ATOM    125  O   LYS A  14      -5.310  -4.031 -23.193  1.00  0.00           O
ATOM    126  N   LYS A  15      -7.163  -5.256 -23.664  1.00  0.00           N
ATOM    127  H   LYS A  15      -7.618  -6.130 -23.483  1.00  0.00           H
ATOM    128  CA  LYS A  15      -7.757  -4.353 -24.624  1.00  0.00           C
ATOM    129  CB  LYS A  15      -9.037  -4.966 -25.181  1.00  0.00           C
ATOM    130  CG  LYS A  15      -8.729  -6.204 -26.022  1.00  0.00           C
ATOM    131  CD  LYS A  15      -9.951  -7.101 -26.203  1.00  0.00           C
ATOM    132  CE  LYS A  15      -9.640  -8.370 -26.999  1.00  0.00           C
ATOM    133  NZ  LYS A  15     -10.751  -9.314 -26.850  1.00  0.00           N
ATOM    134  HZ1 LYS A  15     -11.673  -8.828 -26.779  1.00  0.00           H
ATOM    135  HZ2 LYS A  15     -10.798 -10.063 -27.560  1.00  0.00           H
ATOM    136  HZ3 LYS A  15     -10.714  -9.768 -25.911  1.00  0.00           H
ATOM    137  C   LYS A  15      -8.025  -3.022 -23.959  1.00  0.00           C
ATOM    138  O   LYS A  15      -7.770  -1.972 -24.549  1.00  0.00           O
ATOM    139  N   LEU A  16      -8.529  -3.057 -22.702  1.00  0.00           N
ATOM    140  H   LEU A  16      -8.611  -3.953 -22.263  1.00  0.00           H
ATOM    141  CA  LEU A  16      -8.843  -1.905 -21.897  1.00  0.00           C
ATOM    142  CB  LEU A  16      -9.821  -2.298 -20.795  1.00  0.00           C
ATOM    143  CG  LEU A  16     -11.113  -2.885 -21.363  1.00  0.00           C
ATOM    144  CD1 LEU A  16     -12.000  -3.475 -20.266  1.00  0.00           C
ATOM    145  CD2 LEU A  16     -11.861  -1.871 -22.229  1.00  0.00           C
ATOM    146  C   LEU A  16      -7.591  -1.298 -21.320  1.00  0.00           C
ATOM    147  O   LEU A  16      -7.484  -0.076 -21.222  1.00  0.00           O
ATOM    148  N   LEU A  17      -6.600  -2.159 -20.972  1.00  0.00           N
ATOM    149  H   LEU A  17      -6.775  -3.128 -21.134  1.00  0.00           H
ATOM    150  CA  LEU A  17      -5.316  -1.828 -20.396  1.00  0.00           C
ATOM    151  CB  LEU A  17      -4.582  -3.130 -20.051  1.00  0.00           C
ATOM    152  CG  LEU A  17      -3.260  -3.049 -19.280  1.00  0.00           C
ATOM    153  CD1 LEU A  17      -3.069  -4.315 -18.445  1.00  0.00           C
ATOM    154  CD2 LEU A  17      -2.037  -2.800 -20.170  1.00  0.00           C
ATOM    155  C   LEU A  17      -4.557  -0.967 -21.358  1.00  0.00           C
ATOM    156  O   LEU A  17      -3.824  -0.091 -20.919  1.00  0.00           O
ATOM    157  N   GLU A  18      -4.734  -1.176 -22.685  1.00  0.00           N
ATOM    158  H   GLU A  18      -5.239  -1.990 -22.978  1.00  0.00           H
ATOM    159  CA  GLU A  18      -4.068  -0.401 -23.701  1.00  0.00           C
ATOM    160  CB  GLU A  18      -4.364  -0.925 -25.104  1.00  0.00           C
ATOM    161  CG  GLU A  18      -3.839  -2.335 -25.366  1.00  0.00           C
ATOM    162  CD  GLU A  18      -2.340  -2.387 -25.157  1.00  0.00           C
ATOM    163  OE1 GLU A  18      -1.627  -1.536 -25.688  1.00  0.00           O
ATOM    164  OE2 GLU A  18      -1.857  -3.304 -24.494  1.00  0.00           O
ATOM    165  C   GLU A  18      -4.488   1.047 -23.613  1.00  0.00           C
ATOM    166  O   GLU A  18      -3.693   1.932 -23.923  1.00  0.00           O
ATOM    167  N   GLN A  19      -5.754   1.317 -23.216  1.00  0.00           N
ATOM    168  H   GLN A  19      -6.312   0.555 -22.886  1.00  0.00           H
ATOM    169  CA  GLN A  19      -6.265   2.655 -23.075  1.00  0.00           C
ATOM    170  CB  GLN A  19      -7.775   2.613 -23.253  1.00  0.00           C
ATOM    171  CG  GLN A  19      -8.141   1.829 -24.511  1.00  0.00           C
ATOM    172  CD  GLN A  19      -9.601   1.444 -24.456  1.00  0.00           C
ATOM    173  OE1 GLN A  19     -10.481   2.262 -24.213  1.00  0.00           O
ATOM    174  NE2 GLN A  19      -9.802   0.137 -24.697  1.00  0.00           N
ATOM    175 HE21 GLN A  19     -10.717  -0.264 -24.711  1.00  0.00           H
ATOM    176 HE22 GLN A  19      -9.012  -0.466 -24.856  1.00  0.00           H
ATOM    177  C   GLN A  19      -5.892   3.277 -21.749  1.00  0.00           C
```

```
ATOM    178  O   GLN A  19      -5.472   4.435 -21.699  1.00  0.00           O
ATOM    179  N   TRP A  20      -6.027   2.492 -20.652  1.00  0.00           N
ATOM    180  H   TRP A  20      -6.346   1.554 -20.806  1.00  0.00           H
ATOM    181  CA  TRP A  20      -5.793   2.923 -19.292  1.00  0.00           C
ATOM    182  CB  TRP A  20      -6.454   1.958 -18.306  1.00  0.00           C
ATOM    183  CG  TRP A  20      -7.948   1.892 -18.531  1.00  0.00           C
ATOM    184  CD2 TRP A  20      -8.854   0.853 -18.106  1.00  0.00           C
ATOM    185  CE2 TRP A  20     -10.154   1.218 -18.520  1.00  0.00           C
ATOM    186  CE3 TRP A  20      -8.668  -0.331 -17.405  1.00  0.00           C
ATOM    187  CD1 TRP A  20      -8.761   2.833 -19.184  1.00  0.00           C
ATOM    188  NE1 TRP A  20     -10.061   2.437 -19.182  1.00  0.00           N
ATOM    189  HE1 TRP A  20     -10.821   2.931 -19.554  1.00  0.00           H
ATOM    190  CZ2 TRP A  20     -11.229   0.389 -18.225  1.00  0.00           C
ATOM    191  CZ3 TRP A  20      -9.749  -1.153 -17.113  1.00  0.00           C
ATOM    192  CH2 TRP A  20     -11.028  -0.792 -17.521  1.00  0.00           C
ATOM    193  C   TRP A  20      -4.347   3.140 -18.921  1.00  0.00           C
ATOM    194  O   TRP A  20      -4.020   4.140 -18.275  1.00  0.00           O
ATOM    195  N   ASN A  21      -3.468   2.202 -19.339  1.00  0.00           N
ATOM    196  H   ASN A  21      -3.846   1.437 -19.859  1.00  0.00           H
ATOM    197  CA  ASN A  21      -2.038   2.187 -19.148  1.00  0.00           C
ATOM    198  CB  ASN A  21      -1.276   2.833 -20.309  1.00  0.00           C
ATOM    199  CG  ASN A  21      -0.741   1.707 -21.181  1.00  0.00           C
ATOM    200  OD1 ASN A  21       0.118   0.926 -20.786  1.00  0.00           O
ATOM    201  ND2 ASN A  21      -1.318   1.643 -22.396  1.00  0.00           N
ATOM    202 HD21 ASN A  21      -1.090   0.929 -23.058  1.00  0.00           H
ATOM    203 HD22 ASN A  21      -2.039   2.265 -22.714  1.00  0.00           H
ATOM    204  C   ASN A  21      -1.491   2.463 -17.782  1.00  0.00           C
ATOM    205  O   ASN A  21      -1.497   1.588 -16.912  1.00  0.00           O
ATOM    206  N   LEU A  22      -1.052   3.732 -17.585  1.00  0.00           N
ATOM    207  H   LEU A  22      -1.228   4.356 -18.343  1.00  0.00           H
ATOM    208  CA  LEU A  22      -0.392   4.287 -16.438  1.00  0.00           C
ATOM    209  CB  LEU A  22      -0.277   5.807 -16.569  1.00  0.00           C
ATOM    210  CG  LEU A  22       0.321   6.324 -17.880  1.00  0.00           C
ATOM    211  CD1 LEU A  22       0.314   7.852 -17.927  1.00  0.00           C
ATOM    212  CD2 LEU A  22       1.713   5.763 -18.157  1.00  0.00           C
ATOM    213  C   LEU A  22      -1.087   3.982 -15.159  1.00  0.00           C
ATOM    214  O   LEU A  22      -0.436   3.603 -14.185  1.00  0.00           O
ATOM    215  N   VAL A  23      -2.433   4.055 -15.180  1.00  0.00           N
ATOM    216  H   VAL A  23      -2.874   4.219 -16.063  1.00  0.00           H
ATOM    217  CA  VAL A  23      -3.225   3.855 -14.008  1.00  0.00           C
ATOM    218  CB  VAL A  23      -4.661   4.274 -14.251  1.00  0.00           C
ATOM    219  CG1 VAL A  23      -5.393   4.245 -12.917  1.00  0.00           C
ATOM    220  CG2 VAL A  23      -4.739   5.642 -14.931  1.00  0.00           C
ATOM    221  C   VAL A  23      -3.146   2.418 -13.529  1.00  0.00           C
ATOM    222  O   VAL A  23      -3.091   2.198 -12.320  1.00  0.00           O
ATOM    223  N   ILE A  24      -3.097   1.414 -14.444  1.00  0.00           N
ATOM    224  H   ILE A  24      -2.947   1.620 -15.411  1.00  0.00           H
ATOM    225  CA  ILE A  24      -3.042   0.039 -14.001  1.00  0.00           C
ATOM    226  CB  ILE A  24      -3.562  -0.895 -15.095  1.00  0.00           C
ATOM    227  CG2 ILE A  24      -3.370  -2.366 -14.720  1.00  0.00           C
ATOM    228  CG1 ILE A  24      -5.018  -0.590 -15.425  1.00  0.00           C
ATOM    229  CD1 ILE A  24      -5.578  -1.604 -16.420  1.00  0.00           C
ATOM    230  C   ILE A  24      -1.639  -0.351 -13.635  1.00  0.00           C
ATOM    231  O   ILE A  24      -1.460  -0.969 -12.588  1.00  0.00           O
ATOM    232  N   GLY A  25      -0.620   0.056 -14.430  1.00  0.00           N
ATOM    233  H   GLY A  25      -0.837   0.597 -15.246  1.00  0.00           H
ATOM    234  CA  GLY A  25       0.747  -0.332 -14.174  1.00  0.00           C
ATOM    235  C   GLY A  25       1.321   0.167 -12.897  1.00  0.00           C
ATOM    236  O   GLY A  25       1.957  -0.598 -12.168  1.00  0.00           O
```

```
ATOM    237  N   PHE A  26       1.035   1.446 -12.591  1.00  0.00           N
ATOM    238  H   PHE A  26       0.456   1.977 -13.214  1.00  0.00           H
ATOM    239  CA  PHE A  26       1.522   2.111 -11.419  1.00  0.00           C
ATOM    240  CB  PHE A  26       1.149   3.593 -11.529  1.00  0.00           C
ATOM    241  CG  PHE A  26       1.576   4.431 -10.346  1.00  0.00           C
ATOM    242  CD1 PHE A  26       2.802   5.082 -10.362  1.00  0.00           C
ATOM    243  CD2 PHE A  26       0.731   4.583  -9.253  1.00  0.00           C
ATOM    244  CE1 PHE A  26       3.179   5.884  -9.293  1.00  0.00           C
ATOM    245  CE2 PHE A  26       1.105   5.389  -8.186  1.00  0.00           C
ATOM    246  CZ  PHE A  26       2.330   6.041  -8.205  1.00  0.00           C
ATOM    247  C   PHE A  26       0.954   1.509 -10.165  1.00  0.00           C
ATOM    248  O   PHE A  26       1.697   1.220  -9.221  1.00  0.00           O
ATOM    249  N   LEU A  27      -0.371   1.252 -10.172  1.00  0.00           N
ATOM    250  H   LEU A  27      -0.909   1.418 -10.999  1.00  0.00           H
ATOM    251  CA  LEU A  27      -1.031   0.738  -9.012  1.00  0.00           C
ATOM    252  CB  LEU A  27      -2.536   0.961  -9.109  1.00  0.00           C
ATOM    253  CG  LEU A  27      -2.855   2.451  -9.216  1.00  0.00           C
ATOM    254  CD1 LEU A  27      -4.340   2.698  -9.465  1.00  0.00           C
ATOM    255  CD2 LEU A  27      -2.323   3.235  -8.019  1.00  0.00           C
ATOM    256  C   LEU A  27      -0.705  -0.690  -8.747  1.00  0.00           C
ATOM    257  O   LEU A  27      -0.324  -1.015  -7.628  1.00  0.00           O
ATOM    258  N   PHE A  28      -0.748  -1.538  -9.796  1.00  0.00           N
ATOM    259  H   PHE A  28      -0.981  -1.167 -10.697  1.00  0.00           H
ATOM    260  CA  PHE A  28      -0.510  -2.951  -9.699  1.00  0.00           C
ATOM    261  CB  PHE A  28      -0.883  -3.636 -11.013  1.00  0.00           C
ATOM    262  CG  PHE A  28      -0.759  -5.137 -10.897  1.00  0.00           C
ATOM    263  CD1 PHE A  28      -1.500  -5.830  -9.947  1.00  0.00           C
ATOM    264  CD2 PHE A  28       0.081  -5.827 -11.763  1.00  0.00           C
ATOM    265  CE1 PHE A  28      -1.426  -7.217  -9.888  1.00  0.00           C
ATOM    266  CE2 PHE A  28       0.153  -7.214 -11.705  1.00  0.00           C
ATOM    267  CZ  PHE A  28      -0.613  -7.910 -10.777  1.00  0.00           C
ATOM    268  C   PHE A  28       0.885  -3.325  -9.305  1.00  0.00           C
ATOM    269  O   PHE A  28       1.031  -4.125  -8.380  1.00  0.00           O
ATOM    270  N   LEU A  29       1.931  -2.732  -9.927  1.00  0.00           N
ATOM    271  H   LEU A  29       1.787  -2.017 -10.617  1.00  0.00           H
ATOM    272  CA  LEU A  29       3.278  -3.113  -9.581  1.00  0.00           C
ATOM    273  CB  LEU A  29       4.285  -2.663 -10.634  1.00  0.00           C
ATOM    274  CG  LEU A  29       4.068  -3.360 -11.976  1.00  0.00           C
ATOM    275  CD1 LEU A  29       5.185  -3.014 -12.953  1.00  0.00           C
ATOM    276  CD2 LEU A  29       3.910  -4.875 -11.830  1.00  0.00           C
ATOM    277  C   LEU A  29       3.688  -2.672  -8.218  1.00  0.00           C
ATOM    278  O   LEU A  29       4.326  -3.448  -7.500  1.00  0.00           O
ATOM    279  N   THR A  30       3.292  -1.440  -7.816  1.00  0.00           N
ATOM    280  H   THR A  30       2.755  -0.856  -8.427  1.00  0.00           H
ATOM    281  CA  THR A  30       3.642  -0.943  -6.512  1.00  0.00           C
ATOM    282  CB  THR A  30       3.461   0.564  -6.415  1.00  0.00           C
ATOM    283  OG1 THR A  30       4.173   1.226  -7.476  1.00  0.00           O
ATOM    284  HG1 THR A  30       3.761   0.958  -8.303  1.00  0.00           H
ATOM    285  CG2 THR A  30       3.998   1.037  -5.060  1.00  0.00           C
ATOM    286  C   THR A  30       2.895  -1.755  -5.466  1.00  0.00           C
ATOM    287  O   THR A  30       3.462  -2.067  -4.421  1.00  0.00           O
ATOM    288  N   TRP A  31       1.636  -2.169  -5.758  1.00  0.00           N
ATOM    289  H   TRP A  31       1.218  -1.902  -6.629  1.00  0.00           H
ATOM    290  CA  TRP A  31       0.806  -2.945  -4.865  1.00  0.00           C
ATOM    291  CB  TRP A  31      -0.636  -3.081  -5.363  1.00  0.00           C
ATOM    292  CG  TRP A  31      -1.615  -3.292  -4.223  1.00  0.00           C
ATOM    293  CD2 TRP A  31      -3.050  -3.409  -4.329  1.00  0.00           C
ATOM    294  CE2 TRP A  31      -3.553  -3.589  -3.001  1.00  0.00           C
ATOM    295  CE3 TRP A  31      -3.901  -3.372  -5.448  1.00  0.00           C
```

ATOM	296	CD1	TRP	A	31	-1.338	-3.403	-2.848	1.00	0.00	C
ATOM	297	NE1	TRP	A	31	-2.475	-3.578	-2.125	1.00	0.00	N
ATOM	298	HE1	TRP	A	31	-2.531	-3.685	-1.152	1.00	0.00	H
ATOM	299	CZ2	TRP	A	31	-4.932	-3.727	-2.808	1.00	0.00	C
ATOM	300	CZ3	TRP	A	31	-5.280	-3.510	-5.248	1.00	0.00	C
ATOM	301	CH2	TRP	A	31	-5.763	-3.685	-3.939	1.00	0.00	C
ATOM	302	C	TRP	A	31	1.429	-4.297	-4.621	1.00	0.00	C
ATOM	303	O	TRP	A	31	1.373	-4.777	-3.490	1.00	0.00	O
ATOM	304	N	ILE	A	32	2.072	-4.922	-5.650	1.00	0.00	N
ATOM	305	H	ILE	A	32	2.059	-4.491	-6.555	1.00	0.00	H
ATOM	306	CA	ILE	A	32	2.714	-6.217	-5.499	1.00	0.00	C
ATOM	307	CB	ILE	A	32	3.249	-6.741	-6.833	1.00	0.00	C
ATOM	308	CG2	ILE	A	32	4.170	-7.945	-6.628	1.00	0.00	C
ATOM	309	CG1	ILE	A	32	2.113	-7.082	-7.793	1.00	0.00	C
ATOM	310	CD1	ILE	A	32	2.663	-7.606	-9.118	1.00	0.00	C
ATOM	311	C	ILE	A	32	3.856	-6.046	-4.525	1.00	0.00	C
ATOM	312	O	ILE	A	32	3.998	-6.859	-3.610	1.00	0.00	O
ATOM	313	N	CYS	A	33	4.637	-4.943	-4.661	1.00	0.00	N
ATOM	314	H	CYS	A	33	4.469	-4.326	-5.433	1.00	0.00	H
ATOM	315	CA	CYS	A	33	5.764	-4.662	-3.803	1.00	0.00	C
ATOM	316	CB	CYS	A	33	6.507	-3.419	-4.291	1.00	0.00	C
ATOM	317	SG	CYS	A	33	7.121	-3.547	-5.990	1.00	0.00	S
ATOM	318	C	CYS	A	33	5.304	-4.484	-2.381	1.00	0.00	C
ATOM	319	O	CYS	A	33	5.937	-5.012	-1.465	1.00	0.00	O
ATOM	320	N	LEU	A	34	4.150	-3.794	-2.182	1.00	0.00	N
ATOM	321	H	LEU	A	34	3.675	-3.428	-2.985	1.00	0.00	H
ATOM	322	CA	LEU	A	34	3.583	-3.545	-0.881	1.00	0.00	C
ATOM	323	CB	LEU	A	34	2.398	-2.583	-0.965	1.00	0.00	C
ATOM	324	CG	LEU	A	34	2.790	-1.190	-1.458	1.00	0.00	C
ATOM	325	CD1	LEU	A	34	1.567	-0.289	-1.630	1.00	0.00	C
ATOM	326	CD2	LEU	A	34	3.865	-0.547	-0.581	1.00	0.00	C
ATOM	327	C	LEU	A	34	3.168	-4.832	-0.240	1.00	0.00	C
ATOM	328	O	LEU	A	34	3.427	-5.020	0.946	1.00	0.00	O
ATOM	329	N	LEU	A	35	2.561	-5.763	-1.016	1.00	0.00	N
ATOM	330	H	LEU	A	35	2.405	-5.561	-1.986	1.00	0.00	H
ATOM	331	CA	LEU	A	35	2.135	-7.031	-0.488	1.00	0.00	C
ATOM	332	CB	LEU	A	35	1.203	-7.775	-1.441	1.00	0.00	C
ATOM	333	CG	LEU	A	35	-0.164	-7.091	-1.528	1.00	0.00	C
ATOM	334	CD1	LEU	A	35	-1.126	-7.878	-2.407	1.00	0.00	C
ATOM	335	CD2	LEU	A	35	-0.778	-6.827	-0.152	1.00	0.00	C
ATOM	336	C	LEU	A	35	3.301	-7.857	-0.057	1.00	0.00	C
ATOM	337	O	LEU	A	35	3.206	-8.528	0.969	1.00	0.00	O
ATOM	338	N	GLN	A	36	4.439	-7.771	-0.791	1.00	0.00	N
ATOM	339	H	GLN	A	36	4.443	-7.192	-1.611	1.00	0.00	H
ATOM	340	CA	GLN	A	36	5.637	-8.506	-0.470	1.00	0.00	C
ATOM	341	CB	GLN	A	36	6.687	-8.348	-1.568	1.00	0.00	C
ATOM	342	CG	GLN	A	36	6.263	-9.046	-2.862	1.00	0.00	C
ATOM	343	CD	GLN	A	36	7.315	-8.808	-3.922	1.00	0.00	C
ATOM	344	OE1	GLN	A	36	7.975	-7.774	-3.945	1.00	0.00	O
ATOM	345	NE2	GLN	A	36	7.444	-9.826	-4.790	1.00	0.00	N
ATOM	346	HE21	GLN	A	36	8.080	-9.813	-5.560	1.00	0.00	H
ATOM	347	HE22	GLN	A	36	6.893	-10.666	-4.712	1.00	0.00	H
ATOM	348	C	GLN	A	36	6.148	-8.036	0.869	1.00	0.00	C
ATOM	349	O	GLN	A	36	6.435	-8.868	1.730	1.00	0.00	O
ATOM	350	N	PHE	A	37	6.174	-6.696	1.104	1.00	0.00	N
ATOM	351	H	PHE	A	37	5.904	-6.084	0.356	1.00	0.00	H
ATOM	352	CA	PHE	A	37	6.613	-6.116	2.358	1.00	0.00	C
ATOM	353	CB	PHE	A	37	6.740	-4.595	2.273	1.00	0.00	C
ATOM	354	CG	PHE	A	37	7.635	-4.177	1.132	1.00	0.00	C

```
ATOM    355  CD1 PHE A  37       7.337  -3.018   0.426  1.00  0.00           C
ATOM    356  CD2 PHE A  37       8.748  -4.936   0.786  1.00  0.00           C
ATOM    357  CE1 PHE A  37       8.138  -2.628  -0.639  1.00  0.00           C
ATOM    358  CE2 PHE A  37       9.549  -4.545  -0.280  1.00  0.00           C
ATOM    359  CZ  PHE A  37       9.240  -3.396  -0.998  1.00  0.00           C
ATOM    360  C   PHE A  37       5.655  -6.458   3.478  1.00  0.00           C
ATOM    361  O   PHE A  37       6.088  -6.719   4.601  1.00  0.00           O
ATOM    362  N   ALA A  38       4.336  -6.530   3.160  1.00  0.00           N
ATOM    363  H   ALA A  38       4.112  -6.266   2.220  1.00  0.00           H
ATOM    364  CA  ALA A  38       3.244  -6.809   4.063  1.00  0.00           C
ATOM    365  CB  ALA A  38       1.893  -6.711   3.355  1.00  0.00           C
ATOM    366  C   ALA A  38       3.354  -8.140   4.736  1.00  0.00           C
ATOM    367  O   ALA A  38       2.734  -8.333   5.783  1.00  0.00           O
ATOM    368  N   TYR A  39       4.144  -9.089   4.168  1.00  0.00           N
ATOM    369  H   TYR A  39       4.622  -8.855   3.318  1.00  0.00           H
ATOM    370  CA  TYR A  39       4.323 -10.387   4.771  1.00  0.00           C
ATOM    371  CB  TYR A  39       5.234 -11.262   3.893  1.00  0.00           C
ATOM    372  CG  TYR A  39       4.534 -11.885   2.698  1.00  0.00           C
ATOM    373  CD1 TYR A  39       3.647 -11.168   1.906  1.00  0.00           C
ATOM    374  CE1 TYR A  39       3.039 -11.756   0.804  1.00  0.00           C
ATOM    375  CD2 TYR A  39       4.801 -13.209   2.374  1.00  0.00           C
ATOM    376  CE2 TYR A  39       4.189 -13.807   1.278  1.00  0.00           C
ATOM    377  CZ  TYR A  39       3.309 -13.080   0.486  1.00  0.00           C
ATOM    378  OH  TYR A  39       2.705 -13.667  -0.609  1.00  0.00           O
ATOM    379  HH  TYR A  39       2.337 -12.994  -1.166  1.00  0.00           H
ATOM    380  C   TYR A  39       4.961 -10.250   6.133  1.00  0.00           C
ATOM    381  O   TYR A  39       4.651 -11.027   7.036  1.00  0.00           O
ATOM    382  N   ALA A  40       5.828  -9.220   6.319  1.00  0.00           N
ATOM    383  H   ALA A  40       5.997  -8.600   5.549  1.00  0.00           H
ATOM    384  CA  ALA A  40       6.487  -8.945   7.574  1.00  0.00           C
ATOM    385  CB  ALA A  40       7.508  -7.816   7.429  1.00  0.00           C
ATOM    386  C   ALA A  40       5.474  -8.535   8.611  1.00  0.00           C
ATOM    387  O   ALA A  40       5.609  -8.899   9.784  1.00  0.00           O
ATOM    388  N   ASN A  41       4.426  -7.785   8.170  1.00  0.00           N
ATOM    389  H   ASN A  41       4.401  -7.569   7.195  1.00  0.00           H
ATOM    390  CA  ASN A  41       3.361  -7.288   9.005  1.00  0.00           C
ATOM    391  CB  ASN A  41       2.476  -6.296   8.248  1.00  0.00           C
ATOM    392  CG  ASN A  41       2.506  -4.952   8.941  1.00  0.00           C
ATOM    393  OD1 ASN A  41       3.552  -4.438   9.307  1.00  0.00           O
ATOM    394  ND2 ASN A  41       1.309  -4.390   9.087  1.00  0.00           N
ATOM    395 HD21 ASN A  41       1.201  -3.477   9.479  1.00  0.00           H
ATOM    396 HD22 ASN A  41       0.461  -4.829   8.783  1.00  0.00           H
ATOM    397  C   ASN A  41       2.541  -8.435   9.504  1.00  0.00           C
ATOM    398  O   ASN A  41       2.215  -8.482  10.691  1.00  0.00           O
ATOM    399  N   ARG A  42       2.242  -9.410   8.609  1.00  0.00           N
ATOM    400  H   ARG A  42       2.574  -9.265   7.675  1.00  0.00           H
ATOM    401  CA  ARG A  42       1.449 -10.569   8.935  1.00  0.00           C
ATOM    402  CB  ARG A  42       1.050 -11.297   7.654  1.00  0.00           C
ATOM    403  CG  ARG A  42       0.250 -10.404   6.702  1.00  0.00           C
ATOM    404  CD  ARG A  42      -0.088 -11.117   5.397  1.00  0.00           C
ATOM    405  NE  ARG A  42      -0.942 -10.311   4.528  1.00  0.00           N
ATOM    406  HE  ARG A  42      -1.912 -10.185   4.775  1.00  0.00           H
ATOM    407  CZ  ARG A  42      -0.491  -9.790   3.369  1.00  0.00           C
ATOM    408  NH1 ARG A  42       0.798  -9.883   3.037  1.00  0.00           N
ATOM    409 HH11 ARG A  42       1.431 -10.342   3.659  1.00  0.00           H
ATOM    410 HH12 ARG A  42       1.164  -9.504   2.185  1.00  0.00           H
ATOM    411  NH2 ARG A  42      -1.355  -9.195   2.557  1.00  0.00           N
ATOM    412 HH21 ARG A  42      -2.337  -9.203   2.795  1.00  0.00           H
ATOM    413 HH22 ARG A  42      -1.114  -8.753   1.697  1.00  0.00           H
```

ATOM	414	C	ARG	A	42	2.171	-11.469	9.901	1.00	0.00	C
ATOM	415	O	ARG	A	42	1.545	-12.002	10.819	1.00	0.00	O
ATOM	416	N	ASN	A	43	3.508	-11.628	9.732	1.00	0.00	N
ATOM	417	H	ASN	A	43	3.927	-11.202	8.929	1.00	0.00	H
ATOM	418	CA	ASN	A	43	4.326	-12.435	10.610	1.00	0.00	C
ATOM	419	CB	ASN	A	43	5.619	-12.888	9.924	1.00	0.00	C
ATOM	420	CG	ASN	A	43	5.313	-14.054	8.999	1.00	0.00	C
ATOM	421	OD1	ASN	A	43	4.264	-14.679	9.061	1.00	0.00	O
ATOM	422	ND2	ASN	A	43	6.263	-14.308	8.092	1.00	0.00	N
ATOM	423	HD21	ASN	A	43	6.048	-15.000	7.396	1.00	0.00	H
ATOM	424	HD22	ASN	A	43	7.138	-13.830	8.084	1.00	0.00	H
ATOM	425	C	ASN	A	43	4.572	-11.794	11.959	1.00	0.00	C
ATOM	426	O	ASN	A	43	5.008	-12.480	12.886	1.00	0.00	O
ATOM	427	N	ARG	A	44	4.280	-10.470	12.093	1.00	0.00	N
ATOM	428	H	ARG	A	44	3.967	-9.997	11.271	1.00	0.00	H
ATOM	429	CA	ARG	A	44	4.418	-9.678	13.295	1.00	0.00	C
ATOM	430	CB	ARG	A	44	3.363	-10.032	14.348	1.00	0.00	C
ATOM	431	CG	ARG	A	44	1.942	-9.796	13.830	1.00	0.00	C
ATOM	432	CD	ARG	A	44	0.890	-9.962	14.925	1.00	0.00	C
ATOM	433	NE	ARG	A	44	-0.468	-9.855	14.387	1.00	0.00	N
ATOM	434	HE	ARG	A	44	-0.826	-8.953	14.121	1.00	0.00	H
ATOM	435	CZ	ARG	A	44	-1.235	-10.954	14.243	1.00	0.00	C
ATOM	436	NH1	ARG	A	44	-0.765	-12.158	14.573	1.00	0.00	N
ATOM	437	HH11	ARG	A	44	0.173	-12.246	14.919	1.00	0.00	H
ATOM	438	HH12	ARG	A	44	-1.310	-12.989	14.478	1.00	0.00	H
ATOM	439	NH2	ARG	A	44	-2.463	-10.812	13.758	1.00	0.00	N
ATOM	440	HH21	ARG	A	44	-2.756	-9.885	13.480	1.00	0.00	H
ATOM	441	HH22	ARG	A	44	-3.105	-11.568	13.645	1.00	0.00	H
ATOM	442	C	ARG	A	44	5.807	-9.564	13.870	1.00	0.00	C
ATOM	443	O	ARG	A	44	6.021	-9.739	15.074	1.00	0.00	O
ATOM	444	N	PHE	A	45	6.798	-9.272	12.993	1.00	0.00	N
ATOM	445	H	PHE	A	45	6.532	-9.058	12.052	1.00	0.00	H
ATOM	446	CA	PHE	A	45	8.170	-9.087	13.415	1.00	0.00	C
ATOM	447	CB	PHE	A	45	9.116	-8.997	12.213	1.00	0.00	C
ATOM	448	CG	PHE	A	45	9.149	-10.279	11.413	1.00	0.00	C
ATOM	449	CD1	PHE	A	45	9.317	-10.221	10.035	1.00	0.00	C
ATOM	450	CD2	PHE	A	45	9.038	-11.512	12.043	1.00	0.00	C
ATOM	451	CE1	PHE	A	45	9.398	-11.393	9.293	1.00	0.00	C
ATOM	452	CE2	PHE	A	45	9.122	-12.685	11.303	1.00	0.00	C
ATOM	453	CZ	PHE	A	45	9.314	-12.626	9.928	1.00	0.00	C
ATOM	454	C	PHE	A	45	8.227	-7.777	14.169	1.00	0.00	C
ATOM	455	O	PHE	A	45	7.605	-6.803	13.753	1.00	0.00	O
ATOM	456	N	LEU	A	46	8.971	-7.683	15.294	1.00	0.00	N
ATOM	457	H	LEU	A	46	9.414	-8.506	15.648	1.00	0.00	H
ATOM	458	CA	LEU	A	46	9.058	-6.424	16.013	1.00	0.00	C
ATOM	459	CB	LEU	A	46	9.768	-6.593	17.353	1.00	0.00	C
ATOM	460	CG	LEU	A	46	8.860	-7.217	18.411	1.00	0.00	C
ATOM	461	CD1	LEU	A	46	9.595	-7.439	19.733	1.00	0.00	C
ATOM	462	CD2	LEU	A	46	7.578	-6.404	18.596	1.00	0.00	C
ATOM	463	C	LEU	A	46	9.742	-5.360	15.182	1.00	0.00	C
ATOM	464	O	LEU	A	46	9.476	-4.165	15.330	1.00	0.00	O
ATOM	465	N	TYR	A	47	10.601	-5.802	14.237	1.00	0.00	N
ATOM	466	H	TYR	A	47	10.733	-6.789	14.170	1.00	0.00	H
ATOM	467	CA	TYR	A	47	11.328	-4.971	13.320	1.00	0.00	C
ATOM	468	CB	TYR	A	47	12.543	-5.732	12.802	1.00	0.00	C
ATOM	469	CG	TYR	A	47	13.630	-5.577	13.838	1.00	0.00	C
ATOM	470	CD1	TYR	A	47	14.096	-6.663	14.573	1.00	0.00	C
ATOM	471	CE1	TYR	A	47	15.092	-6.481	15.529	1.00	0.00	C
ATOM	472	CD2	TYR	A	47	14.156	-4.313	14.062	1.00	0.00	C

ATOM	473	CE2	TYR	A	47	15.149	-4.132	15.013	1.00	0.00	C
ATOM	474	CZ	TYR	A	47	15.625	-5.213	15.743	1.00	0.00	C
ATOM	475	OH	TYR	A	47	16.632	-4.991	16.659	1.00	0.00	O
ATOM	476	HH	TYR	A	47	16.730	-5.751	17.237	1.00	0.00	H
ATOM	477	C	TYR	A	47	10.495	-4.327	12.238	1.00	0.00	C
ATOM	478	O	TYR	A	47	11.051	-3.556	11.449	1.00	0.00	O
ATOM	479	N	ILE	A	48	9.149	-4.592	12.202	1.00	0.00	N
ATOM	480	H	ILE	A	48	8.762	-5.257	12.842	1.00	0.00	H
ATOM	481	CA	ILE	A	48	8.221	-4.022	11.238	1.00	0.00	C
ATOM	482	CB	ILE	A	48	6.796	-4.380	11.668	1.00	0.00	C
ATOM	483	CG2	ILE	A	48	5.778	-3.306	11.300	1.00	0.00	C
ATOM	484	CG1	ILE	A	48	6.370	-5.747	11.149	1.00	0.00	C
ATOM	485	CD1	ILE	A	48	5.082	-6.178	11.850	1.00	0.00	C
ATOM	486	C	ILE	A	48	8.364	-2.530	11.199	1.00	0.00	C
ATOM	487	O	ILE	A	48	8.359	-2.001	10.093	1.00	0.00	O
ATOM	488	N	ILE	A	49	8.638	-1.867	12.352	1.00	0.00	N
ATOM	489	H	ILE	A	49	8.707	-2.418	13.180	1.00	0.00	H
ATOM	490	CA	ILE	A	49	8.740	-0.429	12.458	1.00	0.00	C
ATOM	491	CB	ILE	A	49	9.097	-0.021	13.893	1.00	0.00	C
ATOM	492	CG2	ILE	A	49	10.470	-0.539	14.328	1.00	0.00	C
ATOM	493	CG1	ILE	A	49	8.913	1.484	14.086	1.00	0.00	C
ATOM	494	CD1	ILE	A	49	8.940	1.909	15.552	1.00	0.00	C
ATOM	495	C	ILE	A	49	9.633	0.202	11.407	1.00	0.00	C
ATOM	496	O	ILE	A	49	9.257	1.229	10.844	1.00	0.00	O
ATOM	497	N	LYS	A	50	10.780	-0.424	11.068	1.00	0.00	N
ATOM	498	H	LYS	A	50	10.940	-1.353	11.404	1.00	0.00	H
ATOM	499	CA	LYS	A	50	11.655	0.130	10.070	1.00	0.00	C
ATOM	500	CB	LYS	A	50	13.054	-0.460	10.249	1.00	0.00	C
ATOM	501	CG	LYS	A	50	13.558	-0.271	11.685	1.00	0.00	C
ATOM	502	CD	LYS	A	50	14.927	-0.905	11.947	1.00	0.00	C
ATOM	503	CE	LYS	A	50	15.401	-0.720	13.392	1.00	0.00	C
ATOM	504	NZ	LYS	A	50	16.682	-1.416	13.596	1.00	0.00	N
ATOM	505	HZ1	LYS	A	50	16.574	-2.429	13.388	1.00	0.00	H
ATOM	506	HZ2	LYS	A	50	17.406	-1.010	12.969	1.00	0.00	H
ATOM	507	HZ3	LYS	A	50	16.986	-1.309	14.586	1.00	0.00	H
ATOM	508	C	LYS	A	50	11.152	-0.007	8.657	1.00	0.00	C
ATOM	509	O	LYS	A	50	11.374	0.889	7.844	1.00	0.00	O
ATOM	510	N	LEU	A	51	10.470	-1.130	8.337	1.00	0.00	N
ATOM	511	H	LEU	A	51	10.185	-1.728	9.086	1.00	0.00	H
ATOM	512	CA	LEU	A	51	9.947	-1.411	7.019	1.00	0.00	C
ATOM	513	CB	LEU	A	51	9.732	-2.919	6.890	1.00	0.00	C
ATOM	514	CG	LEU	A	51	10.981	-3.720	7.264	1.00	0.00	C
ATOM	515	CD1	LEU	A	51	10.656	-5.184	7.560	1.00	0.00	C
ATOM	516	CD2	LEU	A	51	12.088	-3.572	6.218	1.00	0.00	C
ATOM	517	C	LEU	A	51	8.670	-0.678	6.702	1.00	0.00	C
ATOM	518	O	LEU	A	51	8.463	-0.205	5.579	1.00	0.00	O
ATOM	519	N	ILE	A	52	7.812	-0.520	7.733	1.00	0.00	N
ATOM	520	H	ILE	A	52	8.099	-0.870	8.623	1.00	0.00	H
ATOM	521	CA	ILE	A	52	6.505	0.068	7.656	1.00	0.00	C
ATOM	522	CB	ILE	A	52	5.747	-0.149	8.975	1.00	0.00	C
ATOM	523	CG2	ILE	A	52	6.067	0.918	10.023	1.00	0.00	C
ATOM	524	CG1	ILE	A	52	4.250	-0.301	8.726	1.00	0.00	C
ATOM	525	CD1	ILE	A	52	3.450	-0.604	9.993	1.00	0.00	C
ATOM	526	C	ILE	A	52	6.551	1.512	7.239	1.00	0.00	C
ATOM	527	O	ILE	A	52	5.566	2.008	6.704	1.00	0.00	O
ATOM	528	N	PHE	A	53	7.694	2.213	7.435	1.00	0.00	N
ATOM	529	H	PHE	A	53	8.469	1.765	7.880	1.00	0.00	H
ATOM	530	CA	PHE	A	53	7.821	3.601	7.065	1.00	0.00	C
ATOM	531	CB	PHE	A	53	9.182	4.142	7.501	1.00	0.00	C

ATOM	532	CG	PHE	A	53	9.294	4.159	9.006	1.00	0.00	C
ATOM	533	CD1	PHE	A	53	10.550	4.132	9.600	1.00	0.00	C
ATOM	534	CD2	PHE	A	53	8.152	4.211	9.798	1.00	0.00	C
ATOM	535	CE1	PHE	A	53	10.667	4.166	10.984	1.00	0.00	C
ATOM	536	CE2	PHE	A	53	8.269	4.238	11.182	1.00	0.00	C
ATOM	537	CZ	PHE	A	53	9.526	4.225	11.775	1.00	0.00	C
ATOM	538	C	PHE	A	53	7.639	3.747	5.571	1.00	0.00	C
ATOM	539	O	PHE	A	53	6.827	4.560	5.129	1.00	0.00	O
ATOM	540	N	LEU	A	54	8.324	2.892	4.774	1.00	0.00	N
ATOM	541	H	LEU	A	54	8.858	2.171	5.217	1.00	0.00	H
ATOM	542	CA	LEU	A	54	8.246	2.897	3.333	1.00	0.00	C
ATOM	543	CB	LEU	A	54	9.431	2.154	2.721	1.00	0.00	C
ATOM	544	CG	LEU	A	54	10.731	2.952	2.829	1.00	0.00	C
ATOM	545	CD1	LEU	A	54	11.944	2.129	2.395	1.00	0.00	C
ATOM	546	CD2	LEU	A	54	10.638	4.273	2.063	1.00	0.00	C
ATOM	547	C	LEU	A	54	6.948	2.298	2.870	1.00	0.00	C
ATOM	548	O	LEU	A	54	6.346	2.787	1.906	1.00	0.00	O
ATOM	549	N	TRP	A	55	6.490	1.242	3.595	1.00	0.00	N
ATOM	550	H	TRP	A	55	7.049	0.918	4.360	1.00	0.00	H
ATOM	551	CA	TRP	A	55	5.285	0.518	3.279	1.00	0.00	C
ATOM	552	CB	TRP	A	55	5.198	-0.662	4.248	1.00	0.00	C
ATOM	553	CG	TRP	A	55	4.060	-1.590	3.909	1.00	0.00	C
ATOM	554	CD2	TRP	A	55	2.938	-1.969	4.735	1.00	0.00	C
ATOM	555	CE2	TRP	A	55	2.152	-2.903	3.984	1.00	0.00	C
ATOM	556	CE3	TRP	A	55	2.539	-1.608	6.038	1.00	0.00	C
ATOM	557	CD1	TRP	A	55	3.894	-2.304	2.717	1.00	0.00	C
ATOM	558	NE1	TRP	A	55	2.778	-3.077	2.756	1.00	0.00	N
ATOM	559	HE1	TRP	A	55	2.487	-3.685	2.045	1.00	0.00	H
ATOM	560	CZ2	TRP	A	55	0.984	-3.458	4.549	1.00	0.00	C
ATOM	561	CZ3	TRP	A	55	1.370	-2.169	6.594	1.00	0.00	C
ATOM	562	CH2	TRP	A	55	0.598	-3.089	5.854	1.00	0.00	C
ATOM	563	C	TRP	A	55	4.067	1.388	3.367	1.00	0.00	C
ATOM	564	O	TRP	A	55	3.171	1.224	2.549	1.00	0.00	O
ATOM	565	N	LEU	A	56	3.999	2.300	4.359	1.00	0.00	N
ATOM	566	H	LEU	A	56	4.769	2.372	4.994	1.00	0.00	H
ATOM	567	CA	LEU	A	56	2.904	3.218	4.552	1.00	0.00	C
ATOM	568	CB	LEU	A	56	2.787	3.667	6.000	1.00	0.00	C
ATOM	569	CG	LEU	A	56	2.485	2.511	6.947	1.00	0.00	C
ATOM	570	CD1	LEU	A	56	2.510	2.982	8.397	1.00	0.00	C
ATOM	571	CD2	LEU	A	56	1.195	1.773	6.585	1.00	0.00	C
ATOM	572	C	LEU	A	56	3.007	4.404	3.641	1.00	0.00	C
ATOM	573	O	LEU	A	56	1.989	4.968	3.240	1.00	0.00	O
ATOM	574	N	LEU	A	57	4.256	4.798	3.308	1.00	0.00	N
ATOM	575	H	LEU	A	57	5.029	4.297	3.699	1.00	0.00	H
ATOM	576	CA	LEU	A	57	4.568	5.935	2.492	1.00	0.00	C
ATOM	577	CB	LEU	A	57	6.082	6.123	2.517	1.00	0.00	C
ATOM	578	CG	LEU	A	57	6.609	7.407	1.895	1.00	0.00	C
ATOM	579	CD1	LEU	A	57	6.015	8.644	2.560	1.00	0.00	C
ATOM	580	CD2	LEU	A	57	8.136	7.439	1.916	1.00	0.00	C
ATOM	581	C	LEU	A	57	4.065	5.809	1.067	1.00	0.00	C
ATOM	582	O	LEU	A	57	3.342	6.697	0.611	1.00	0.00	O
ATOM	583	N	TRP	A	58	4.355	4.681	0.363	1.00	0.00	N
ATOM	584	H	TRP	A	58	4.905	3.976	0.818	1.00	0.00	H
ATOM	585	CA	TRP	A	58	3.922	4.488	-1.014	1.00	0.00	C
ATOM	586	CB	TRP	A	58	4.546	3.238	-1.642	1.00	0.00	C
ATOM	587	CG	TRP	A	58	6.013	3.484	-1.891	1.00	0.00	C
ATOM	588	CD2	TRP	A	58	7.068	2.508	-2.001	1.00	0.00	C
ATOM	589	CE2	TRP	A	58	8.272	3.207	-2.250	1.00	0.00	C
ATOM	590	CE3	TRP	A	58	7.081	1.122	-1.912	1.00	0.00	C

ATOM	591	CD1	TRP	A	58	6.641	4.723	-2.084	1.00	0.00	C
ATOM	592	NE1	TRP	A	58	7.974	4.565	-2.296	1.00	0.00	N
ATOM	593	HE1	TRP	A	58	8.616	5.291	-2.449	1.00	0.00	H
ATOM	594	CZ2	TRP	A	58	9.457	2.499	-2.399	1.00	0.00	C
ATOM	595	CZ3	TRP	A	58	8.274	0.428	-2.067	1.00	0.00	C
ATOM	596	CH2	TRP	A	58	9.459	1.113	-2.307	1.00	0.00	C
ATOM	597	C	TRP	A	58	2.421	4.562	-1.255	1.00	0.00	C
ATOM	598	O	TRP	A	58	2.032	5.261	-2.203	1.00	0.00	O
ATOM	599	N	PRO	A	59	1.526	3.951	-0.466	1.00	0.00	N
ATOM	600	CD	PRO	A	59	1.804	3.037	0.637	1.00	0.00	C
ATOM	601	CA	PRO	A	59	0.106	4.032	-0.663	1.00	0.00	C
ATOM	602	CB	PRO	A	59	-0.515	2.984	0.263	1.00	0.00	C
ATOM	603	CG	PRO	A	59	0.491	2.852	1.384	1.00	0.00	C
ATOM	604	C	PRO	A	59	-0.466	5.402	-0.491	1.00	0.00	C
ATOM	605	O	PRO	A	59	-1.641	5.531	-0.813	1.00	0.00	O
ATOM	606	N	VAL	A	60	0.295	6.420	-0.004	1.00	0.00	N
ATOM	607	H	VAL	A	60	1.242	6.265	0.287	1.00	0.00	H
ATOM	608	CA	VAL	A	60	-0.220	7.772	0.048	1.00	0.00	C
ATOM	609	CB	VAL	A	60	0.724	8.685	0.826	1.00	0.00	C
ATOM	610	CG1	VAL	A	60	0.227	10.131	0.811	1.00	0.00	C
ATOM	611	CG2	VAL	A	60	0.921	8.155	2.247	1.00	0.00	C
ATOM	612	C	VAL	A	60	-0.406	8.252	-1.378	1.00	0.00	C
ATOM	613	O	VAL	A	60	-1.493	8.705	-1.755	1.00	0.00	O
ATOM	614	N	THR	A	61	0.636	8.044	-2.216	1.00	0.00	N
ATOM	615	H	THR	A	61	1.413	7.509	-1.883	1.00	0.00	H
ATOM	616	CA	THR	A	61	0.632	8.434	-3.597	1.00	0.00	C
ATOM	617	CB	THR	A	61	2.047	8.398	-4.126	1.00	0.00	C
ATOM	618	OG1	THR	A	61	2.888	9.267	-3.354	1.00	0.00	O
ATOM	619	HG1	THR	A	61	2.730	9.083	-2.436	1.00	0.00	H
ATOM	620	CG2	THR	A	61	2.030	8.801	-5.596	1.00	0.00	C
ATOM	621	C	THR	A	61	-0.298	7.527	-4.346	1.00	0.00	C
ATOM	622	O	THR	A	61	-1.081	8.008	-5.167	1.00	0.00	O
ATOM	623	N	LEU	A	62	-0.271	6.207	-4.020	1.00	0.00	N
ATOM	624	H	LEU	A	62	0.391	5.887	-3.340	1.00	0.00	H
ATOM	625	CA	LEU	A	62	-1.104	5.247	-4.696	1.00	0.00	C
ATOM	626	CB	LEU	A	62	-0.811	3.819	-4.249	1.00	0.00	C
ATOM	627	CG	LEU	A	62	0.184	3.084	-5.142	1.00	0.00	C
ATOM	628	CD1	LEU	A	62	1.581	3.697	-5.082	1.00	0.00	C
ATOM	629	CD2	LEU	A	62	0.174	1.583	-4.852	1.00	0.00	C
ATOM	630	C	LEU	A	62	-2.556	5.545	-4.508	1.00	0.00	C
ATOM	631	O	LEU	A	62	-3.289	5.499	-5.488	1.00	0.00	O
ATOM	632	N	ALA	A	63	-2.981	5.930	-3.281	1.00	0.00	N
ATOM	633	H	ALA	A	63	-2.319	6.002	-2.535	1.00	0.00	H
ATOM	634	CA	ALA	A	63	-4.353	6.231	-2.959	1.00	0.00	C
ATOM	635	CB	ALA	A	63	-4.572	6.466	-1.476	1.00	0.00	C
ATOM	636	C	ALA	A	63	-4.829	7.445	-3.663	1.00	0.00	C
ATOM	637	O	ALA	A	63	-5.933	7.419	-4.202	1.00	0.00	O
ATOM	638	N	CYS	A	64	-3.987	8.509	-3.717	1.00	0.00	N
ATOM	639	H	CYS	A	64	-3.108	8.476	-3.234	1.00	0.00	H
ATOM	640	CA	CYS	A	64	-4.357	9.739	-4.372	1.00	0.00	C
ATOM	641	CB	CYS	A	64	-3.302	10.812	-4.107	1.00	0.00	C
ATOM	642	SG	CYS	A	64	-3.239	11.323	-2.372	1.00	0.00	S
ATOM	643	C	CYS	A	64	-4.554	9.509	-5.846	1.00	0.00	C
ATOM	644	O	CYS	A	64	-5.495	10.040	-6.440	1.00	0.00	O
ATOM	645	N	PHE	A	65	-3.692	8.654	-6.440	1.00	0.00	N
ATOM	646	H	PHE	A	65	-2.985	8.216	-5.881	1.00	0.00	H
ATOM	647	CA	PHE	A	65	-3.712	8.334	-7.836	1.00	0.00	C
ATOM	648	CB	PHE	A	65	-2.407	7.635	-8.217	1.00	0.00	C
ATOM	649	CG	PHE	A	65	-2.295	7.507	-9.715	1.00	0.00	C

ATOM	650	CD1	PHE	A	65	-2.335	8.643	-10.515	1.00	0.00	C
ATOM	651	CD2	PHE	A	65	-2.138	6.254	-10.294	1.00	0.00	C
ATOM	652	CE1	PHE	A	65	-2.202	8.525	-11.894	1.00	0.00	C
ATOM	653	CE2	PHE	A	65	-2.002	6.138	-11.671	1.00	0.00	C
ATOM	654	CZ	PHE	A	65	-2.028	7.273	-12.471	1.00	0.00	C
ATOM	655	C	PHE	A	65	-4.916	7.478	-8.183	1.00	0.00	C
ATOM	656	O	PHE	A	65	-5.544	7.739	-9.208	1.00	0.00	O
ATOM	657	N	VAL	A	66	-5.297	6.486	-7.324	1.00	0.00	N
ATOM	658	H	VAL	A	66	-4.746	6.336	-6.502	1.00	0.00	H
ATOM	659	CA	VAL	A	66	-6.422	5.596	-7.579	1.00	0.00	C
ATOM	660	CB	VAL	A	66	-6.664	4.478	-6.554	1.00	0.00	C
ATOM	661	CG1	VAL	A	66	-7.792	3.606	-7.103	1.00	0.00	C
ATOM	662	CG2	VAL	A	66	-5.471	3.611	-6.189	1.00	0.00	C
ATOM	663	C	VAL	A	66	-7.678	6.439	-7.524	1.00	0.00	C
ATOM	664	O	VAL	A	66	-8.498	6.371	-8.439	1.00	0.00	O
ATOM	665	N	LEU	A	67	-7.814	7.295	-6.478	1.00	0.00	N
ATOM	666	H	LEU	A	67	-7.086	7.313	-5.787	1.00	0.00	H
ATOM	667	CA	LEU	A	67	-8.993	8.101	-6.288	1.00	0.00	C
ATOM	668	CB	LEU	A	67	-8.960	8.842	-4.950	1.00	0.00	C
ATOM	669	CG	LEU	A	67	-8.843	7.897	-3.749	1.00	0.00	C
ATOM	670	CD1	LEU	A	67	-8.663	8.658	-2.437	1.00	0.00	C
ATOM	671	CD2	LEU	A	67	-9.965	6.861	-3.696	1.00	0.00	C
ATOM	672	C	LEU	A	67	-9.224	9.043	-7.421	1.00	0.00	C
ATOM	673	O	LEU	A	67	-10.363	9.170	-7.869	1.00	0.00	O
ATOM	674	N	ALA	A	68	-8.146	9.665	-7.950	1.00	0.00	N
ATOM	675	H	ALA	A	68	-7.248	9.508	-7.540	1.00	0.00	H
ATOM	676	CA	ALA	A	68	-8.258	10.569	-9.064	1.00	0.00	C
ATOM	677	CB	ALA	A	68	-6.938	11.307	-9.293	1.00	0.00	C
ATOM	678	C	ALA	A	68	-8.631	9.816	-10.321	1.00	0.00	C
ATOM	679	O	ALA	A	68	-9.481	10.267	-11.091	1.00	0.00	O
ATOM	680	N	ALA	A	69	-8.040	8.614	-10.504	1.00	0.00	N
ATOM	681	H	ALA	A	69	-7.430	8.254	-9.793	1.00	0.00	H
ATOM	682	CA	ALA	A	69	-8.232	7.780	-11.657	1.00	0.00	C
ATOM	683	CB	ALA	A	69	-6.901	7.202	-12.099	1.00	0.00	C
ATOM	684	C	ALA	A	69	-9.289	6.705	-11.568	1.00	0.00	C
ATOM	685	O	ALA	A	69	-9.383	5.882	-12.484	1.00	0.00	O
ATOM	686	N	VAL	A	70	-10.123	6.690	-10.492	1.00	0.00	N
ATOM	687	H	VAL	A	70	-9.896	7.299	-9.727	1.00	0.00	H
ATOM	688	CA	VAL	A	70	-11.181	5.710	-10.300	1.00	0.00	C
ATOM	689	CB	VAL	A	70	-11.857	5.866	-8.921	1.00	0.00	C
ATOM	690	CG1	VAL	A	70	-12.698	7.141	-8.792	1.00	0.00	C
ATOM	691	CG2	VAL	A	70	-12.644	4.608	-8.539	1.00	0.00	C
ATOM	692	C	VAL	A	70	-12.163	5.744	-11.458	1.00	0.00	C
ATOM	693	O	VAL	A	70	-12.759	4.729	-11.804	1.00	0.00	O
ATOM	694	N	TYR	A	71	-12.316	6.922	-12.102	1.00	0.00	N
ATOM	695	H	TYR	A	71	-11.807	7.694	-11.729	1.00	0.00	H
ATOM	696	CA	TYR	A	71	-13.172	7.138	-13.235	1.00	0.00	C
ATOM	697	CB	TYR	A	71	-13.227	8.624	-13.588	1.00	0.00	C
ATOM	698	CG	TYR	A	71	-14.179	8.818	-14.743	1.00	0.00	C
ATOM	699	CD1	TYR	A	71	-15.540	8.601	-14.560	1.00	0.00	C
ATOM	700	CE1	TYR	A	71	-16.417	8.755	-15.626	1.00	0.00	C
ATOM	701	CD2	TYR	A	71	-13.697	9.200	-15.989	1.00	0.00	C
ATOM	702	CE2	TYR	A	71	-14.574	9.352	-17.056	1.00	0.00	C
ATOM	703	CZ	TYR	A	71	-15.934	9.127	-16.875	1.00	0.00	C
ATOM	704	OH	TYR	A	71	-16.805	9.275	-17.935	1.00	0.00	O
ATOM	705	HH	TYR	A	71	-16.314	9.465	-18.723	1.00	0.00	H
ATOM	706	C	TYR	A	71	-12.655	6.321	-14.404	1.00	0.00	C
ATOM	707	O	TYR	A	71	-13.452	5.753	-15.155	1.00	0.00	O
ATOM	708	N	ARG	A	72	-11.310	6.258	-14.568	1.00	0.00	N

```
ATOM    709  H   ARG A  72     -10.728   6.581 -13.823  1.00  0.00           H
ATOM    710  CA  ARG A  72     -10.686   5.531 -15.636  1.00  0.00           C
ATOM    711  CB  ARG A  72      -9.249   5.992 -15.872  1.00  0.00           C
ATOM    712  CG  ARG A  72      -9.056   7.387 -16.457  1.00  0.00           C
ATOM    713  CD  ARG A  72      -7.561   7.615 -16.689  1.00  0.00           C
ATOM    714  NE  ARG A  72      -7.290   8.867 -17.391  1.00  0.00           N
ATOM    715  HE  ARG A  72      -7.688   9.693 -16.976  1.00  0.00           H
ATOM    716  CZ  ARG A  72      -6.502   8.828 -18.509  1.00  0.00           C
ATOM    717  NH1 ARG A  72      -6.034   7.636 -18.955  1.00  0.00           N
ATOM    718 HH11 ARG A  72      -6.237   6.774 -18.479  1.00  0.00           H
ATOM    719 HH12 ARG A  72      -5.465   7.539 -19.778  1.00  0.00           H
ATOM    720  NH2 ARG A  72      -6.204   9.982 -19.151  1.00  0.00           N
ATOM    721 HH21 ARG A  72      -6.548  10.867 -18.821  1.00  0.00           H
ATOM    722 HH22 ARG A  72      -5.631  10.013 -19.975  1.00  0.00           H
ATOM    723  C   ARG A  72     -10.620   4.049 -15.423  1.00  0.00           C
ATOM    724  O   ARG A  72     -10.908   3.317 -16.366  1.00  0.00           O
ATOM    725  N   ILE A  73     -10.247   3.561 -14.208  1.00  0.00           N
ATOM    726  H   ILE A  73     -10.058   4.188 -13.450  1.00  0.00           H
ATOM    727  CA  ILE A  73     -10.141   2.125 -14.019  1.00  0.00           C
ATOM    728  CB  ILE A  73      -8.778   1.700 -13.492  1.00  0.00           C
ATOM    729  CG2 ILE A  73      -7.687   2.028 -14.504  1.00  0.00           C
ATOM    730  CG1 ILE A  73      -8.544   2.233 -12.079  1.00  0.00           C
ATOM    731  CD1 ILE A  73      -7.435   1.472 -11.353  1.00  0.00           C
ATOM    732  C   ILE A  73     -11.180   1.389 -13.235  1.00  0.00           C
ATOM    733  O   ILE A  73     -11.226   0.158 -13.329  1.00  0.00           O
ATOM    734  N   ASN A  74     -12.058   2.107 -12.495  1.00  0.00           N
ATOM    735  H   ASN A  74     -11.941   3.097 -12.435  1.00  0.00           H
ATOM    736  CA  ASN A  74     -13.057   1.523 -11.623  1.00  0.00           C
ATOM    737  CB  ASN A  74     -14.173   0.670 -12.225  1.00  0.00           C
ATOM    738  CG  ASN A  74     -15.193   0.475 -11.111  1.00  0.00           C
ATOM    739  OD1 ASN A  74     -15.046  -0.361 -10.222  1.00  0.00           O
ATOM    740  ND2 ASN A  74     -16.233   1.322 -11.191  1.00  0.00           N
ATOM    741 HD21 ASN A  74     -16.951   1.365 -10.497  1.00  0.00           H
ATOM    742 HD22 ASN A  74     -16.312   1.958 -11.960  1.00  0.00           H
ATOM    743  C   ASN A  74     -12.254   0.802 -10.554  1.00  0.00           C
ATOM    744  O   ASN A  74     -11.485   1.487  -9.874  1.00  0.00           O
ATOM    745  N   TRP A  75     -12.385  -0.550 -10.374  1.00  0.00           N
ATOM    746  H   TRP A  75     -13.021  -1.053 -10.954  1.00  0.00           H
ATOM    747  CA  TRP A  75     -11.659  -1.331  -9.381  1.00  0.00           C
ATOM    748  CB  TRP A  75     -10.166  -1.446  -9.714  1.00  0.00           C
ATOM    749  CG  TRP A  75      -9.916  -2.262 -10.963  1.00  0.00           C
ATOM    750  CD2 TRP A  75      -8.636  -2.574 -11.551  1.00  0.00           C
ATOM    751  CE2 TRP A  75      -8.872  -3.361 -12.701  1.00  0.00           C
ATOM    752  CE3 TRP A  75      -7.332  -2.254 -11.191  1.00  0.00           C
ATOM    753  CD1 TRP A  75     -10.858  -2.882 -11.798  1.00  0.00           C
ATOM    754  NE1 TRP A  75     -10.247  -3.530 -12.825  1.00  0.00           N
ATOM    755  HE1 TRP A  75     -10.699  -4.017 -13.544  1.00  0.00           H
ATOM    756  CZ2 TRP A  75      -7.797  -3.803 -13.462  1.00  0.00           C
ATOM    757  CZ3 TRP A  75      -6.266  -2.705 -11.959  1.00  0.00           C
ATOM    758  CH2 TRP A  75      -6.498  -3.477 -13.091  1.00  0.00           C
ATOM    759  C   TRP A  75     -11.824  -0.697  -8.024  1.00  0.00           C
ATOM    760  O   TRP A  75     -10.866  -0.388  -7.313  1.00  0.00           O
ATOM    761  N   ILE A  76     -13.109  -0.490  -7.671  1.00  0.00           N
ATOM    762  H   ILE A  76     -13.777  -0.688  -8.395  1.00  0.00           H
ATOM    763  CA  ILE A  76     -13.573   0.155  -6.473  1.00  0.00           C
ATOM    764  CB  ILE A  76     -15.102   0.344  -6.589  1.00  0.00           C
ATOM    765  CG2 ILE A  76     -15.969  -0.607  -5.753  1.00  0.00           C
ATOM    766  CG1 ILE A  76     -15.449   1.823  -6.426  1.00  0.00           C
ATOM    767  CD1 ILE A  76     -16.901   2.134  -6.801  1.00  0.00           C
```

ATOM	768	C	ILE	A	76	-13.088	-0.549	-5.230	1.00	0.00	C
ATOM	769	O	ILE	A	76	-12.678	0.135	-4.294	1.00	0.00	O
ATOM	770	N	THR	A	77	-13.046	-1.905	-5.216	1.00	0.00	N
ATOM	771	H	THR	A	77	-13.419	-2.432	-5.976	1.00	0.00	H
ATOM	772	CA	THR	A	77	-12.604	-2.655	-4.059	1.00	0.00	C
ATOM	773	CB	THR	A	77	-12.861	-4.139	-4.313	1.00	0.00	C
ATOM	774	OG1	THR	A	77	-14.040	-4.309	-5.122	1.00	0.00	O
ATOM	775	HG1	THR	A	77	-14.033	-5.220	-5.394	1.00	0.00	H
ATOM	776	CG2	THR	A	77	-12.960	-4.936	-3.010	1.00	0.00	C
ATOM	777	C	THR	A	77	-11.130	-2.395	-3.811	1.00	0.00	C
ATOM	778	O	THR	A	77	-10.734	-2.153	-2.665	1.00	0.00	O
ATOM	779	N	GLY	A	78	-10.314	-2.353	-4.898	1.00	0.00	N
ATOM	780	H	GLY	A	78	-10.703	-2.355	-5.817	1.00	0.00	H
ATOM	781	CA	GLY	A	78	-8.898	-2.116	-4.781	1.00	0.00	C
ATOM	782	C	GLY	A	78	-8.621	-0.695	-4.387	1.00	0.00	C
ATOM	783	O	GLY	A	78	-7.688	-0.442	-3.623	1.00	0.00	O
ATOM	784	N	GLY	A	79	-9.469	0.246	-4.869	1.00	0.00	N
ATOM	785	H	GLY	A	79	-10.155	-0.043	-5.537	1.00	0.00	H
ATOM	786	CA	GLY	A	79	-9.341	1.648	-4.592	1.00	0.00	C
ATOM	787	C	GLY	A	79	-9.598	1.984	-3.166	1.00	0.00	C
ATOM	788	O	GLY	A	79	-8.868	2.790	-2.590	1.00	0.00	O
ATOM	789	N	ILE	A	80	-10.623	1.337	-2.564	1.00	0.00	N
ATOM	790	H	ILE	A	80	-11.162	0.717	-3.138	1.00	0.00	H
ATOM	791	CA	ILE	A	80	-10.989	1.557	-1.187	1.00	0.00	C
ATOM	792	CB	ILE	A	80	-12.170	0.766	-0.568	1.00	0.00	C
ATOM	793	CG2	ILE	A	80	-12.800	1.599	0.543	1.00	0.00	C
ATOM	794	CG1	ILE	A	80	-13.193	0.085	-1.457	1.00	0.00	C
ATOM	795	CD1	ILE	A	80	-14.089	-0.898	-0.698	1.00	0.00	C
ATOM	796	C	ILE	A	80	-9.898	0.992	-0.318	1.00	0.00	C
ATOM	797	O	ILE	A	80	-9.507	1.652	0.641	1.00	0.00	O
ATOM	798	N	ALA	A	81	-9.327	-0.184	-0.686	1.00	0.00	N
ATOM	799	H	ALA	A	81	-9.707	-0.671	-1.476	1.00	0.00	H
ATOM	800	CA	ALA	A	81	-8.289	-0.822	0.090	1.00	0.00	C
ATOM	801	CB	ALA	A	81	-7.933	-2.189	-0.499	1.00	0.00	C
ATOM	802	C	ALA	A	81	-7.047	0.019	0.170	1.00	0.00	C
ATOM	803	O	ALA	A	81	-6.486	0.183	1.255	1.00	0.00	O
ATOM	804	N	ILE	A	82	-6.630	0.615	-0.971	1.00	0.00	N
ATOM	805	H	ILE	A	82	-7.144	0.431	-1.810	1.00	0.00	H
ATOM	806	CA	ILE	A	82	-5.452	1.441	-1.027	1.00	0.00	C
ATOM	807	CB	ILE	A	82	-4.980	1.650	-2.464	1.00	0.00	C
ATOM	808	CG2	ILE	A	82	-3.769	2.575	-2.499	1.00	0.00	C
ATOM	809	CG1	ILE	A	82	-4.629	0.303	-3.099	1.00	0.00	C
ATOM	810	CD1	ILE	A	82	-4.083	0.438	-4.521	1.00	0.00	C
ATOM	811	C	ILE	A	82	-5.709	2.743	-0.285	1.00	0.00	C
ATOM	812	O	ILE	A	82	-4.832	3.204	0.448	1.00	0.00	O
ATOM	813	N	ALA	A	83	-6.931	3.326	-0.407	1.00	0.00	N
ATOM	814	H	ALA	A	83	-7.604	2.902	-1.017	1.00	0.00	H
ATOM	815	CA	ALA	A	83	-7.284	4.565	0.253	1.00	0.00	C
ATOM	816	CB	ALA	A	83	-8.671	5.043	-0.179	1.00	0.00	C
ATOM	817	C	ALA	A	83	-7.264	4.417	1.750	1.00	0.00	C
ATOM	818	O	ALA	A	83	-6.779	5.312	2.446	1.00	0.00	O
ATOM	819	N	MET	A	84	-7.752	3.258	2.263	1.00	0.00	N
ATOM	820	H	MET	A	84	-8.118	2.591	1.612	1.00	0.00	H
ATOM	821	CA	MET	A	84	-7.794	2.966	3.674	1.00	0.00	C
ATOM	822	CB	MET	A	84	-8.693	1.773	3.993	1.00	0.00	C
ATOM	823	CG	MET	A	84	-10.163	2.091	3.720	1.00	0.00	C
ATOM	824	SD	MET	A	84	-10.695	3.595	4.552	1.00	0.00	S
ATOM	825	CE	MET	A	84	-11.206	4.523	3.098	1.00	0.00	C
ATOM	826	C	MET	A	84	-6.407	2.795	4.201	1.00	0.00	C

ATOM	827	O	MET	A	84	-6.068	3.443	5.191	1.00	0.00	O
ATOM	828	N	ALA	A	85	-5.519	2.160	3.389	1.00	0.00	N
ATOM	829	H	ALA	A	85	-5.880	1.711	2.568	1.00	0.00	H
ATOM	830	CA	ALA	A	85	-4.142	1.925	3.749	1.00	0.00	C
ATOM	831	CB	ALA	A	85	-3.428	1.086	2.688	1.00	0.00	C
ATOM	832	C	ALA	A	85	-3.431	3.237	3.910	1.00	0.00	C
ATOM	833	O	ALA	A	85	-2.652	3.390	4.845	1.00	0.00	O
ATOM	834	N	CYS	A	86	-3.726	4.219	3.026	1.00	0.00	N
ATOM	835	H	CYS	A	86	-4.363	4.024	2.277	1.00	0.00	H
ATOM	836	CA	CYS	A	86	-3.132	5.530	3.076	1.00	0.00	C
ATOM	837	CB	CYS	A	86	-3.472	6.270	1.794	1.00	0.00	C
ATOM	838	SG	CYS	A	86	-3.007	8.019	1.788	1.00	0.00	S
ATOM	839	C	CYS	A	86	-3.551	6.344	4.263	1.00	0.00	C
ATOM	840	O	CYS	A	86	-2.691	6.915	4.931	1.00	0.00	O
ATOM	841	N	LEU	A	87	-4.867	6.380	4.583	1.00	0.00	N
ATOM	842	H	LEU	A	87	-5.513	5.847	4.031	1.00	0.00	H
ATOM	843	CA	LEU	A	87	-5.370	7.177	5.677	1.00	0.00	C
ATOM	844	CB	LEU	A	87	-6.897	7.240	5.633	1.00	0.00	C
ATOM	845	CG	LEU	A	87	-7.439	7.881	4.351	1.00	0.00	C
ATOM	846	CD1	LEU	A	87	-8.957	7.741	4.248	1.00	0.00	C
ATOM	847	CD2	LEU	A	87	-6.979	9.331	4.185	1.00	0.00	C
ATOM	848	C	LEU	A	87	-4.899	6.670	7.007	1.00	0.00	C
ATOM	849	O	LEU	A	87	-4.457	7.451	7.853	1.00	0.00	O
ATOM	850	N	VAL	A	88	-4.925	5.329	7.176	1.00	0.00	N
ATOM	851	H	VAL	A	88	-5.251	4.768	6.412	1.00	0.00	H
ATOM	852	CA	VAL	A	88	-4.503	4.668	8.386	1.00	0.00	C
ATOM	853	CB	VAL	A	88	-4.980	3.208	8.404	1.00	0.00	C
ATOM	854	CG1	VAL	A	88	-4.472	2.428	9.622	1.00	0.00	C
ATOM	855	CG2	VAL	A	88	-6.507	3.162	8.331	1.00	0.00	C
ATOM	856	C	VAL	A	88	-2.996	4.780	8.467	1.00	0.00	C
ATOM	857	O	VAL	A	88	-2.448	4.992	9.550	1.00	0.00	O
ATOM	858	N	GLY	A	89	-2.321	4.719	7.294	1.00	0.00	N
ATOM	859	H	GLY	A	89	-2.827	4.572	6.444	1.00	0.00	H
ATOM	860	CA	GLY	A	89	-0.898	4.793	7.161	1.00	0.00	C
ATOM	861	C	GLY	A	89	-0.329	6.093	7.593	1.00	0.00	C
ATOM	862	O	GLY	A	89	0.727	6.100	8.215	1.00	0.00	O
ATOM	863	N	LEU	A	90	-1.024	7.214	7.298	1.00	0.00	N
ATOM	864	H	LEU	A	90	-1.849	7.130	6.735	1.00	0.00	H
ATOM	865	CA	LEU	A	90	-0.577	8.528	7.686	1.00	0.00	C
ATOM	866	CB	LEU	A	90	-1.485	9.590	7.071	1.00	0.00	C
ATOM	867	CG	LEU	A	90	-1.272	9.739	5.568	1.00	0.00	C
ATOM	868	CD1	LEU	A	90	-2.376	10.563	4.904	1.00	0.00	C
ATOM	869	CD2	LEU	A	90	0.119	10.291	5.269	1.00	0.00	C
ATOM	870	C	LEU	A	90	-0.586	8.665	9.180	1.00	0.00	C
ATOM	871	O	LEU	A	90	0.359	9.216	9.751	1.00	0.00	O
ATOM	872	N	MET	A	91	-1.629	8.096	9.839	1.00	0.00	N
ATOM	873	H	MET	A	91	-2.321	7.614	9.299	1.00	0.00	H
ATOM	874	CA	MET	A	91	-1.768	8.151	11.274	1.00	0.00	C
ATOM	875	CB	MET	A	91	-3.152	7.685	11.725	1.00	0.00	C
ATOM	876	CG	MET	A	91	-4.279	8.546	11.155	1.00	0.00	C
ATOM	877	SD	MET	A	91	-5.887	8.094	11.824	1.00	0.00	S
ATOM	878	CE	MET	A	91	-5.833	6.338	11.441	1.00	0.00	C
ATOM	879	C	MET	A	91	-0.698	7.335	11.935	1.00	0.00	C
ATOM	880	O	MET	A	91	0.007	7.849	12.805	1.00	0.00	O
ATOM	881	N	TRP	A	92	-0.397	6.152	11.348	1.00	0.00	N
ATOM	882	H	TRP	A	92	-0.965	5.838	10.584	1.00	0.00	H
ATOM	883	CA	TRP	A	92	0.603	5.262	11.861	1.00	0.00	C
ATOM	884	CB	TRP	A	92	0.487	3.885	11.216	1.00	0.00	C
ATOM	885	CG	TRP	A	92	-0.661	3.093	11.800	1.00	0.00	C

ATOM	886	CD2	TRP	A	92	-0.860	1.667	11.715	1.00	0.00	C
ATOM	887	CE2	TRP	A	92	-2.075	1.363	12.415	1.00	0.00	C
ATOM	888	CE3	TRP	A	92	-0.114	0.631	11.114	1.00	0.00	C
ATOM	889	CD1	TRP	A	92	-1.754	3.572	12.540	1.00	0.00	C
ATOM	890	NE1	TRP	A	92	-2.588	2.560	12.903	1.00	0.00	N
ATOM	891	HE1	TRP	A	92	-3.413	2.660	13.424	1.00	0.00	H
ATOM	892	CZ2	TRP	A	92	-2.522	0.026	12.497	1.00	0.00	C
ATOM	893	CZ3	TRP	A	92	-0.571	-0.700	11.205	1.00	0.00	C
ATOM	894	CH2	TRP	A	92	-1.768	-1.002	11.891	1.00	0.00	C
ATOM	895	C	TRP	A	92	1.980	5.818	11.695	1.00	0.00	C
ATOM	896	O	TRP	A	92	2.799	5.603	12.581	1.00	0.00	O
ATOM	897	N	LEU	A	93	2.271	6.552	10.587	1.00	0.00	N
ATOM	898	H	LEU	A	93	1.571	6.660	9.879	1.00	0.00	H
ATOM	899	CA	LEU	A	93	3.571	7.148	10.370	1.00	0.00	C
ATOM	900	CB	LEU	A	93	3.657	7.822	9.001	1.00	0.00	C
ATOM	901	CG	LEU	A	93	3.859	6.832	7.859	1.00	0.00	C
ATOM	902	CD1	LEU	A	93	3.815	7.514	6.491	1.00	0.00	C
ATOM	903	CD2	LEU	A	93	5.135	6.015	8.057	1.00	0.00	C
ATOM	904	C	LEU	A	93	3.846	8.163	11.429	1.00	0.00	C
ATOM	905	O	LEU	A	93	4.943	8.176	11.984	1.00	0.00	O
ATOM	906	N	SER	A	94	2.824	8.976	11.784	1.00	0.00	N
ATOM	907	H	SER	A	94	1.949	8.905	11.307	1.00	0.00	H
ATOM	908	CA	SER	A	94	2.957	10.002	12.783	1.00	0.00	C
ATOM	909	CB	SER	A	94	1.694	10.845	12.690	1.00	0.00	C
ATOM	910	OG	SER	A	94	1.515	11.191	11.310	1.00	0.00	O
ATOM	911	HG	SER	A	94	1.011	10.502	10.889	1.00	0.00	H
ATOM	912	C	SER	A	94	3.237	9.381	14.124	1.00	0.00	C
ATOM	913	O	SER	A	94	4.137	9.838	14.825	1.00	0.00	O
ATOM	914	N	TYR	A	95	2.516	8.291	14.480	1.00	0.00	N
ATOM	915	H	TYR	A	95	1.835	7.937	13.835	1.00	0.00	H
ATOM	916	CA	TYR	A	95	2.704	7.626	15.742	1.00	0.00	C
ATOM	917	CB	TYR	A	95	1.495	6.747	16.055	1.00	0.00	C
ATOM	918	CG	TYR	A	95	0.281	7.627	16.227	1.00	0.00	C
ATOM	919	CD1	TYR	A	95	-0.940	7.246	15.685	1.00	0.00	C
ATOM	920	CE1	TYR	A	95	-2.050	8.069	15.831	1.00	0.00	C
ATOM	921	CD2	TYR	A	95	0.391	8.822	16.929	1.00	0.00	C
ATOM	922	CE2	TYR	A	95	-0.719	9.644	17.077	1.00	0.00	C
ATOM	923	CZ	TYR	A	95	-1.939	9.267	16.527	1.00	0.00	C
ATOM	924	OH	TYR	A	95	-3.041	10.083	16.675	1.00	0.00	O
ATOM	925	HH	TYR	A	95	-2.801	10.841	17.191	1.00	0.00	H
ATOM	926	C	TYR	A	95	3.972	6.856	15.914	1.00	0.00	C
ATOM	927	O	TYR	A	95	4.612	6.998	16.952	1.00	0.00	O
ATOM	928	N	PHE	A	96	4.395	6.069	14.904	1.00	0.00	N
ATOM	929	H	PHE	A	96	3.877	6.059	14.049	1.00	0.00	H
ATOM	930	CA	PHE	A	96	5.590	5.267	14.989	1.00	0.00	C
ATOM	931	CB	PHE	A	96	5.704	4.306	13.805	1.00	0.00	C
ATOM	932	CG	PHE	A	96	4.693	3.193	13.924	1.00	0.00	C
ATOM	933	CD1	PHE	A	96	4.463	2.592	15.156	1.00	0.00	C
ATOM	934	CD2	PHE	A	96	4.001	2.760	12.799	1.00	0.00	C
ATOM	935	CE1	PHE	A	96	3.537	1.561	15.264	1.00	0.00	C
ATOM	936	CE2	PHE	A	96	3.075	1.730	12.908	1.00	0.00	C
ATOM	937	CZ	PHE	A	96	2.841	1.131	14.140	1.00	0.00	C
ATOM	938	C	PHE	A	96	6.827	6.104	15.057	1.00	0.00	C
ATOM	939	O	PHE	A	96	7.741	5.787	15.820	1.00	0.00	O
ATOM	940	N	ILE	A	97	6.863	7.189	14.254	1.00	0.00	N
ATOM	941	H	ILE	A	97	6.069	7.414	13.686	1.00	0.00	H
ATOM	942	CA	ILE	A	97	8.000	8.065	14.179	1.00	0.00	C
ATOM	943	CB	ILE	A	97	7.996	8.789	12.835	1.00	0.00	C
ATOM	944	CG2	ILE	A	97	9.062	9.882	12.725	1.00	0.00	C

ATOM	945	CG1	ILE	A	97	8.155	7.712	11.759	1.00	0.00	C
ATOM	946	CD1	ILE	A	97	7.766	8.138	10.347	1.00	0.00	C
ATOM	947	C	ILE	A	97	8.083	8.985	15.372	1.00	0.00	C
ATOM	948	O	ILE	A	97	9.150	9.079	15.984	1.00	0.00	O
ATOM	949	N	ALA	A	98	6.961	9.648	15.748	1.00	0.00	N
ATOM	950	H	ALA	A	98	6.099	9.490	15.264	1.00	0.00	H
ATOM	951	CA	ALA	A	98	6.975	10.571	16.856	1.00	0.00	C
ATOM	952	CB	ALA	A	98	5.803	11.552	16.797	1.00	0.00	C
ATOM	953	C	ALA	A	98	6.991	9.921	18.203	1.00	0.00	C
ATOM	954	O	ALA	A	98	7.837	10.273	19.027	1.00	0.00	O
ATOM	955	N	SER	A	99	6.082	8.942	18.446	1.00	0.00	N
ATOM	956	H	SER	A	99	5.553	8.548	17.698	1.00	0.00	H
ATOM	957	CA	SER	A	99	6.028	8.287	19.724	1.00	0.00	C
ATOM	958	CB	SER	A	99	4.623	7.737	19.907	1.00	0.00	C
ATOM	959	OG	SER	A	99	3.752	8.534	19.097	1.00	0.00	O
ATOM	960	HG	SER	A	99	3.822	8.155	18.227	1.00	0.00	H
ATOM	961	C	SER	A	99	7.136	7.288	19.810	1.00	0.00	C
ATOM	962	O	SER	A	99	7.196	6.305	19.067	1.00	0.00	O
ATOM	963	N	PHE	A	100	8.036	7.558	20.773	1.00	0.00	N
ATOM	964	H	PHE	A	100	7.942	8.450	21.214	1.00	0.00	H
ATOM	965	CA	PHE	A	100	9.191	6.756	21.063	1.00	0.00	C
ATOM	966	CB	PHE	A	100	10.225	7.584	21.819	1.00	0.00	C
ATOM	967	CG	PHE	A	100	10.556	8.794	20.979	1.00	0.00	C
ATOM	968	CD1	PHE	A	100	10.408	10.071	21.506	1.00	0.00	C
ATOM	969	CD2	PHE	A	100	10.998	8.628	19.670	1.00	0.00	C
ATOM	970	CE1	PHE	A	100	10.689	11.183	20.721	1.00	0.00	C
ATOM	971	CE2	PHE	A	100	11.278	9.740	18.886	1.00	0.00	C
ATOM	972	CZ	PHE	A	100	11.120	11.018	19.409	1.00	0.00	C
ATOM	973	C	PHE	A	100	8.806	5.510	21.798	1.00	0.00	C
ATOM	974	O	PHE	A	100	9.466	4.484	21.644	1.00	0.00	O
ATOM	975	N	ARG	A	101	7.694	5.579	22.570	1.00	0.00	N
ATOM	976	H	ARG	A	101	7.231	6.462	22.584	1.00	0.00	H
ATOM	977	CA	ARG	A	101	7.160	4.518	23.387	1.00	0.00	C
ATOM	978	CB	ARG	A	101	5.870	4.961	24.078	1.00	0.00	C
ATOM	979	CG	ARG	A	101	5.925	6.196	24.980	1.00	0.00	C
ATOM	980	CD	ARG	A	101	4.511	6.492	25.486	1.00	0.00	C
ATOM	981	NE	ARG	A	101	4.455	7.616	26.419	1.00	0.00	N
ATOM	982	HE	ARG	A	101	4.692	8.540	26.099	1.00	0.00	H
ATOM	983	CZ	ARG	A	101	3.964	7.457	27.667	1.00	0.00	C
ATOM	984	NH1	ARG	A	101	3.528	6.284	28.103	1.00	0.00	N
ATOM	985	HH11	ARG	A	101	3.513	5.472	27.506	1.00	0.00	H
ATOM	986	HH12	ARG	A	101	3.177	6.208	29.042	1.00	0.00	H
ATOM	987	NH2	ARG	A	101	3.895	8.500	28.479	1.00	0.00	N
ATOM	988	HH21	ARG	A	101	4.196	9.398	28.148	1.00	0.00	H
ATOM	989	HH22	ARG	A	101	3.525	8.395	29.409	1.00	0.00	H
ATOM	990	C	ARG	A	101	6.816	3.287	22.601	1.00	0.00	C
ATOM	991	O	ARG	A	101	6.957	2.183	23.129	1.00	0.00	O
ATOM	992	N	LEU	A	102	6.372	3.443	21.327	1.00	0.00	N
ATOM	993	H	LEU	A	102	6.379	4.353	20.916	1.00	0.00	H
ATOM	994	CA	LEU	A	102	6.021	2.321	20.487	1.00	0.00	C
ATOM	995	CB	LEU	A	102	5.318	2.809	19.222	1.00	0.00	C
ATOM	996	CG	LEU	A	102	4.022	3.546	19.563	1.00	0.00	C
ATOM	997	CD1	LEU	A	102	3.338	4.107	18.320	1.00	0.00	C
ATOM	998	CD2	LEU	A	102	3.072	2.673	20.385	1.00	0.00	C
ATOM	999	C	LEU	A	102	7.215	1.466	20.166	1.00	0.00	C
ATOM	1000	O	LEU	A	102	7.091	0.240	20.127	1.00	0.00	O
ATOM	1001	N	PHE	A	103	8.387	2.104	19.923	1.00	0.00	N
ATOM	1002	H	PHE	A	103	8.433	3.093	20.067	1.00	0.00	H
ATOM	1003	CA	PHE	A	103	9.612	1.408	19.623	1.00	0.00	C

ATOM	1004	CB	PHE	A	103	10.643	2.359	19.012	1.00	0.00	C
ATOM	1005	CG	PHE	A	103	11.908	1.598	18.688	1.00	0.00	C
ATOM	1006	CD1	PHE	A	103	11.942	0.741	17.595	1.00	0.00	C
ATOM	1007	CD2	PHE	A	103	13.037	1.747	19.485	1.00	0.00	C
ATOM	1008	CE1	PHE	A	103	13.098	0.024	17.307	1.00	0.00	C
ATOM	1009	CE2	PHE	A	103	14.191	1.028	19.201	1.00	0.00	C
ATOM	1010	CZ	PHE	A	103	14.220	0.161	18.115	1.00	0.00	C
ATOM	1011	C	PHE	A	103	10.133	0.813	20.911	1.00	0.00	C
ATOM	1012	O	PHE	A	103	10.451	-0.381	20.972	1.00	0.00	O
ATOM	1013	N	ALA	A	104	10.176	1.671	21.968	1.00	0.00	N
ATOM	1014	H	ALA	A	104	9.904	2.613	21.776	1.00	0.00	H
ATOM	1015	CA	ALA	A	104	10.650	1.405	23.304	1.00	0.00	C
ATOM	1016	CB	ALA	A	104	9.661	0.501	24.047	1.00	0.00	C
ATOM	1017	C	ALA	A	104	12.025	0.786	23.192	1.00	0.00	C
ATOM	1018	O	ALA	A	104	12.891	1.354	22.522	1.00	0.00	O
ATOM	1019	N	ARG	A	105	12.283	-0.335	23.897	1.00	0.00	N
ATOM	1020	H	ARG	A	105	11.611	-0.756	24.506	1.00	0.00	H
ATOM	1021	CA	ARG	A	105	13.523	-1.050	23.802	1.00	0.00	C
ATOM	1022	CB	ARG	A	105	14.332	-0.963	25.096	1.00	0.00	C
ATOM	1023	CG	ARG	A	105	14.958	0.423	25.262	1.00	0.00	C
ATOM	1024	CD	ARG	A	105	15.802	0.795	24.039	1.00	0.00	C
ATOM	1025	NE	ARG	A	105	16.419	2.112	24.186	1.00	0.00	N
ATOM	1026	HE	ARG	A	105	17.143	2.167	24.882	1.00	0.00	H
ATOM	1027	CZ	ARG	A	105	16.011	3.143	23.383	1.00	0.00	C
ATOM	1028	NH1	ARG	A	105	14.992	2.956	22.515	1.00	0.00	N
ATOM	1029	HH11	ARG	A	105	14.465	2.092	22.479	1.00	0.00	H
ATOM	1030	HH12	ARG	A	105	14.678	3.654	21.868	1.00	0.00	H
ATOM	1031	NH2	ARG	A	105	16.635	4.341	23.473	1.00	0.00	N
ATOM	1032	HH21	ARG	A	105	17.395	4.488	24.113	1.00	0.00	H
ATOM	1033	HH22	ARG	A	105	16.372	5.131	22.911	1.00	0.00	H
ATOM	1034	C	ARG	A	105	13.199	-2.477	23.430	1.00	0.00	C
ATOM	1035	O	ARG	A	105	14.085	-3.258	23.084	1.00	0.00	O
ATOM	1036	N	THR	A	106	11.891	-2.834	23.462	1.00	0.00	N
ATOM	1037	H	THR	A	106	11.177	-2.164	23.643	1.00	0.00	H
ATOM	1038	CA	THR	A	106	11.359	-4.140	23.185	1.00	0.00	C
ATOM	1039	CB	THR	A	106	9.907	-4.014	23.596	1.00	0.00	C
ATOM	1040	OG1	THR	A	106	9.833	-2.969	24.582	1.00	0.00	O
ATOM	1041	HG1	THR	A	106	8.960	-3.022	24.958	1.00	0.00	H
ATOM	1042	CG2	THR	A	106	9.320	-5.329	24.111	1.00	0.00	C
ATOM	1043	C	THR	A	106	11.571	-4.538	21.747	1.00	0.00	C
ATOM	1044	O	THR	A	106	11.956	-5.681	21.496	1.00	0.00	O
ATOM	1045	N	ARG	A	107	11.401	-3.597	20.785	1.00	0.00	N
ATOM	1046	H	ARG	A	107	11.161	-2.659	21.047	1.00	0.00	H
ATOM	1047	CA	ARG	A	107	11.551	-3.904	19.379	1.00	0.00	C
ATOM	1048	CB	ARG	A	107	11.030	-2.787	18.489	1.00	0.00	C
ATOM	1049	CG	ARG	A	107	9.527	-2.644	18.688	1.00	0.00	C
ATOM	1050	CD	ARG	A	107	8.898	-1.705	17.668	1.00	0.00	C
ATOM	1051	NE	ARG	A	107	7.467	-1.585	17.916	1.00	0.00	N
ATOM	1052	HE	ARG	A	107	7.223	-0.967	18.677	1.00	0.00	H
ATOM	1053	CZ	ARG	A	107	6.595	-2.351	17.202	1.00	0.00	C
ATOM	1054	NH1	ARG	A	107	7.063	-3.190	16.247	1.00	0.00	N
ATOM	1055	HH11	ARG	A	107	8.052	-3.284	16.057	1.00	0.00	H
ATOM	1056	HH12	ARG	A	107	6.463	-3.768	15.687	1.00	0.00	H
ATOM	1057	NH2	ARG	A	107	5.273	-2.255	17.470	1.00	0.00	N
ATOM	1058	HH21	ARG	A	107	4.943	-1.633	18.188	1.00	0.00	H
ATOM	1059	HH22	ARG	A	107	4.574	-2.784	16.982	1.00	0.00	H
ATOM	1060	C	ARG	A	107	12.942	-4.321	18.990	1.00	0.00	C
ATOM	1061	O	ARG	A	107	13.096	-5.212	18.152	1.00	0.00	O
ATOM	1062	N	SER	A	108	13.969	-3.713	19.623	1.00	0.00	N

ATOM	1063	H	SER	A	108	13.734	-3.027	20.307	1.00	0.00	H
ATOM	1064	CA	SER	A	108	15.359	-4.003	19.377	1.00	0.00	C
ATOM	1065	CB	SER	A	108	16.110	-2.836	20.003	1.00	0.00	C
ATOM	1066	OG	SER	A	108	15.190	-2.124	20.847	1.00	0.00	O
ATOM	1067	HG	SER	A	108	15.197	-2.594	21.681	1.00	0.00	H
ATOM	1068	C	SER	A	108	15.789	-5.357	19.901	1.00	0.00	C
ATOM	1069	O	SER	A	108	16.745	-5.941	19.386	1.00	0.00	O
ATOM	1070	N	MET	A	109	15.067	-5.881	20.926	1.00	0.00	N
ATOM	1071	H	MET	A	109	14.243	-5.401	21.227	1.00	0.00	H
ATOM	1072	CA	MET	A	109	15.341	-7.146	21.572	1.00	0.00	C
ATOM	1073	CB	MET	A	109	14.526	-7.309	22.856	1.00	0.00	C
ATOM	1074	CG	MET	A	109	14.888	-6.287	23.934	1.00	0.00	C
ATOM	1075	SD	MET	A	109	16.612	-6.389	24.442	1.00	0.00	S
ATOM	1076	CE	MET	A	109	16.588	-8.067	25.095	1.00	0.00	C
ATOM	1077	C	MET	A	109	15.178	-8.369	20.712	1.00	0.00	C
ATOM	1078	O	MET	A	109	15.786	-9.395	21.022	1.00	0.00	O
ATOM	1079	N	TRP	A	110	14.348	-8.305	19.640	1.00	0.00	N
ATOM	1080	H	TRP	A	110	13.987	-7.406	19.397	1.00	0.00	H
ATOM	1081	CA	TRP	A	110	14.073	-9.410	18.745	1.00	0.00	C
ATOM	1082	CB	TRP	A	110	13.049	-8.867	17.741	1.00	0.00	C
ATOM	1083	CG	TRP	A	110	12.583	-9.854	16.696	1.00	0.00	C
ATOM	1084	CD2	TRP	A	110	11.276	-10.452	16.596	1.00	0.00	C
ATOM	1085	CE2	TRP	A	110	11.258	-11.252	15.432	1.00	0.00	C
ATOM	1086	CE3	TRP	A	110	10.141	-10.367	17.391	1.00	0.00	C
ATOM	1087	CD1	TRP	A	110	13.287	-10.332	15.579	1.00	0.00	C
ATOM	1088	NE1	TRP	A	110	12.509	-11.158	14.828	1.00	0.00	N
ATOM	1089	HE1	TRP	A	110	12.792	-11.612	14.001	1.00	0.00	H
ATOM	1090	CZ2	TRP	A	110	10.097	-11.939	15.100	1.00	0.00	C
ATOM	1091	CZ3	TRP	A	110	8.988	-11.061	17.046	1.00	0.00	C
ATOM	1092	CH2	TRP	A	110	8.967	-11.847	15.902	1.00	0.00	C
ATOM	1093	C	TRP	A	110	15.354	-9.839	18.068	1.00	0.00	C
ATOM	1094	O	TRP	A	110	15.990	-9.047	17.369	1.00	0.00	O
ATOM	1095	N	SER	A	111	15.767	-11.110	18.311	1.00	0.00	N
ATOM	1096	H	SER	A	111	15.325	-11.663	19.019	1.00	0.00	H
ATOM	1097	CA	SER	A	111	16.970	-11.622	17.709	1.00	0.00	C
ATOM	1098	CB	SER	A	111	17.895	-11.702	18.901	1.00	0.00	C
ATOM	1099	OG	SER	A	111	17.025	-11.748	20.038	1.00	0.00	O
ATOM	1100	HG	SER	A	111	16.851	-10.839	20.284	1.00	0.00	H
ATOM	1101	C	SER	A	111	16.691	-12.934	17.016	1.00	0.00	C
ATOM	1102	O	SER	A	111	16.752	-13.022	15.790	1.00	0.00	O
ATOM	1103	N	PHE	A	112	16.359	-13.984	17.806	1.00	0.00	N
ATOM	1104	H	PHE	A	112	16.255	-13.808	18.789	1.00	0.00	H
ATOM	1105	CA	PHE	A	112	16.062	-15.315	17.315	1.00	0.00	C
ATOM	1106	CB	PHE	A	112	16.956	-16.346	18.001	1.00	0.00	C
ATOM	1107	CG	PHE	A	112	18.398	-15.954	17.790	1.00	0.00	C
ATOM	1108	CD1	PHE	A	112	19.193	-15.617	18.879	1.00	0.00	C
ATOM	1109	CD2	PHE	A	112	18.925	-15.922	16.504	1.00	0.00	C
ATOM	1110	CE1	PHE	A	112	20.515	-15.236	18.680	1.00	0.00	C
ATOM	1111	CE2	PHE	A	112	20.247	-15.541	16.307	1.00	0.00	C
ATOM	1112	CZ	PHE	A	112	21.040	-15.193	17.394	1.00	0.00	C
ATOM	1113	C	PHE	A	112	14.606	-15.631	17.532	1.00	0.00	C
ATOM	1114	O	PHE	A	112	14.189	-16.778	17.406	1.00	0.00	O
ATOM	1115	N	ASN	A	113	13.801	-14.598	17.847	1.00	0.00	N
ATOM	1116	H	ASN	A	113	14.220	-13.694	17.919	1.00	0.00	H
ATOM	1117	CA	ASN	A	113	12.401	-14.667	18.160	1.00	0.00	C
ATOM	1118	CB	ASN	A	113	11.853	-13.336	18.676	1.00	0.00	C
ATOM	1119	CG	ASN	A	113	12.626	-12.867	19.878	1.00	0.00	C
ATOM	1120	OD1	ASN	A	113	13.795	-12.518	19.757	1.00	0.00	O
ATOM	1121	ND2	ASN	A	113	11.925	-12.846	21.025	1.00	0.00	N

```
ATOM   1122 HD21 ASN A 113      10.971 -13.152  21.041  1.00  0.00           H
ATOM   1123 HD22 ASN A 113      12.324 -12.522  21.885  1.00  0.00           H
ATOM   1124  C   ASN A 113      11.340 -15.215  17.241  1.00  0.00           C
ATOM   1125  O   ASN A 113      10.379 -15.685  17.855  1.00  0.00           O
ATOM   1126  N   PRO A 114      11.313 -15.202  15.895  1.00  0.00           N
ATOM   1127  CD  PRO A 114      12.352 -14.662  15.021  1.00  0.00           C
ATOM   1128  CA  PRO A 114      10.204 -15.770  15.149  1.00  0.00           C
ATOM   1129  CB  PRO A 114      10.517 -15.396  13.696  1.00  0.00           C
ATOM   1130  CG  PRO A 114      12.027 -15.194  13.636  1.00  0.00           C
ATOM   1131  C   PRO A 114      10.036 -17.245  15.402  1.00  0.00           C
ATOM   1132  O   PRO A 114      10.969 -18.018  15.173  1.00  0.00           O
ATOM   1133  N   GLU A 115       8.818 -17.623  15.856  1.00  0.00           N
ATOM   1134  H   GLU A 115       8.099 -16.930  15.877  1.00  0.00           H
ATOM   1135  CA  GLU A 115       8.585 -18.881  16.525  1.00  0.00           C
ATOM   1136  CB  GLU A 115       7.178 -19.011  17.139  1.00  0.00           C
ATOM   1137  CG  GLU A 115       6.284 -17.779  17.338  1.00  0.00           C
ATOM   1138  CD  GLU A 115       4.837 -18.202  17.105  1.00  0.00           C
ATOM   1139  OE1 GLU A 115       3.969 -17.928  17.940  1.00  0.00           O
ATOM   1140  OE2 GLU A 115       4.582 -18.818  16.070  1.00  0.00           O
ATOM   1141  C   GLU A 115       8.644 -20.023  15.562  1.00  0.00           C
ATOM   1142  O   GLU A 115       9.244 -21.057  15.858  1.00  0.00           O
ATOM   1143  N   THR A 116       8.059 -19.815  14.360  1.00  0.00           N
ATOM   1144  H   THR A 116       7.549 -18.969  14.194  1.00  0.00           H
ATOM   1145  CA  THR A 116       7.996 -20.790  13.304  1.00  0.00           C
ATOM   1146  CB  THR A 116       7.049 -20.238  12.235  1.00  0.00           C
ATOM   1147  OG1 THR A 116       6.189 -19.221  12.783  1.00  0.00           O
ATOM   1148  HG1 THR A 116       5.623 -19.608  13.449  1.00  0.00           H
ATOM   1149  CG2 THR A 116       6.256 -21.353  11.553  1.00  0.00           C
ATOM   1150  C   THR A 116       9.400 -21.036  12.787  1.00  0.00           C
ATOM   1151  O   THR A 116       9.745 -22.172  12.469  1.00  0.00           O
ATOM   1152  N   ASN A 117      10.245 -19.976  12.734  1.00  0.00           N
ATOM   1153  H   ASN A 117       9.916 -19.106  13.100  1.00  0.00           H
ATOM   1154  CA  ASN A 117      11.615 -20.040  12.277  1.00  0.00           C
ATOM   1155  CB  ASN A 117      12.241 -18.654  12.214  1.00  0.00           C
ATOM   1156  CG  ASN A 117      13.669 -18.722  12.721  1.00  0.00           C
ATOM   1157  OD1 ASN A 117      14.588 -19.110  12.011  1.00  0.00           O
ATOM   1158  ND2 ASN A 117      13.806 -18.267  13.984  1.00  0.00           N
ATOM   1159 HD21 ASN A 117      13.008 -17.985  14.527  1.00  0.00           H
ATOM   1160 HD22 ASN A 117      14.697 -18.202  14.435  1.00  0.00           H
ATOM   1161  C   ASN A 117      12.440 -20.914  13.183  1.00  0.00           C
ATOM   1162  O   ASN A 117      13.261 -21.682  12.690  1.00  0.00           O
ATOM   1163  N   ILE A 118      12.216 -20.835  14.515  1.00  0.00           N
ATOM   1164  H   ILE A 118      11.499 -20.218  14.844  1.00  0.00           H
ATOM   1165  CA  ILE A 118      12.950 -21.603  15.500  1.00  0.00           C
ATOM   1166  CB  ILE A 118      12.594 -21.154  16.919  1.00  0.00           C
ATOM   1167  CG2 ILE A 118      13.322 -22.001  17.964  1.00  0.00           C
ATOM   1168  CG1 ILE A 118      12.836 -19.666  17.135  1.00  0.00           C
ATOM   1169  CD1 ILE A 118      12.237 -19.182  18.456  1.00  0.00           C
ATOM   1170  C   ILE A 118      12.578 -23.066  15.366  1.00  0.00           C
ATOM   1171  O   ILE A 118      13.463 -23.921  15.299  1.00  0.00           O
ATOM   1172  N   LEU A 119      11.256 -23.350  15.270  1.00  0.00           N
ATOM   1173  H   LEU A 119      10.605 -22.589  15.321  1.00  0.00           H
ATOM   1174  CA  LEU A 119      10.731 -24.688  15.205  1.00  0.00           C
ATOM   1175  CB  LEU A 119       9.213 -24.628  15.403  1.00  0.00           C
ATOM   1176  CG  LEU A 119       8.549 -25.977  15.692  1.00  0.00           C
ATOM   1177  CD1 LEU A 119       8.963 -26.530  17.057  1.00  0.00           C
ATOM   1178  CD2 LEU A 119       7.029 -25.908  15.537  1.00  0.00           C
ATOM   1179  C   LEU A 119      11.059 -25.445  13.945  1.00  0.00           C
ATOM   1180  O   LEU A 119      11.546 -26.575  14.026  1.00  0.00           O
```

```
ATOM   1181  N   LEU A 120      10.839 -24.825  12.769  1.00  0.00           N
ATOM   1182  H   LEU A 120      10.504 -23.885  12.790  1.00  0.00           H
ATOM   1183  CA  LEU A 120      11.067 -25.477  11.509  1.00  0.00           C
ATOM   1184  CB  LEU A 120       9.907 -25.180  10.560  1.00  0.00           C
ATOM   1185  CG  LEU A 120       8.520 -25.523  11.108  1.00  0.00           C
ATOM   1186  CD1 LEU A 120       7.424 -25.008  10.175  1.00  0.00           C
ATOM   1187  CD2 LEU A 120       8.360 -27.015  11.414  1.00  0.00           C
ATOM   1188  C   LEU A 120      12.381 -25.206  10.841  1.00  0.00           C
ATOM   1189  O   LEU A 120      12.738 -25.948   9.923  1.00  0.00           O
ATOM   1190  N   ASN A 121      13.145 -24.177  11.298  1.00  0.00           N
ATOM   1191  H   ASN A 121      12.803 -23.614  12.053  1.00  0.00           H
ATOM   1192  CA  ASN A 121      14.419 -23.764  10.728  1.00  0.00           C
ATOM   1193  CB  ASN A 121      15.618 -24.711  10.947  1.00  0.00           C
ATOM   1194  CG  ASN A 121      15.287 -25.955  11.756  1.00  0.00           C
ATOM   1195  OD1 ASN A 121      15.287 -27.062  11.217  1.00  0.00           O
ATOM   1196  ND2 ASN A 121      14.977 -25.737  13.053  1.00  0.00           N
ATOM   1197 HD21 ASN A 121      14.685 -26.480  13.660  1.00  0.00           H
ATOM   1198 HD22 ASN A 121      14.984 -24.839  13.507  1.00  0.00           H
ATOM   1199  C   ASN A 121      14.244 -23.363   9.280  1.00  0.00           C
ATOM   1200  O   ASN A 121      15.033 -23.725   8.402  1.00  0.00           O
ATOM   1201  N   VAL A 122      13.159 -22.595   9.020  1.00  0.00           N
ATOM   1202  H   VAL A 122      12.663 -22.203   9.794  1.00  0.00           H
ATOM   1203  CA  VAL A 122      12.802 -22.132   7.706  1.00  0.00           C
ATOM   1204  CB  VAL A 122      11.331 -22.439   7.430  1.00  0.00           C
ATOM   1205  CG1 VAL A 122      10.857 -21.858   6.107  1.00  0.00           C
ATOM   1206  CG2 VAL A 122      11.129 -23.952   7.428  1.00  0.00           C
ATOM   1207  C   VAL A 122      13.152 -20.665   7.561  1.00  0.00           C
ATOM   1208  O   VAL A 122      12.551 -19.830   8.242  1.00  0.00           O
ATOM   1209  N   PRO A 123      14.071 -20.319   6.648  1.00  0.00           N
ATOM   1210  CD  PRO A 123      14.796 -21.296   5.842  1.00  0.00           C
ATOM   1211  CA  PRO A 123      14.518 -18.958   6.397  1.00  0.00           C
ATOM   1212  CB  PRO A 123      15.640 -19.132   5.369  1.00  0.00           C
ATOM   1213  CG  PRO A 123      16.096 -20.583   5.501  1.00  0.00           C
ATOM   1214  C   PRO A 123      13.426 -18.035   5.922  1.00  0.00           C
ATOM   1215  O   PRO A 123      13.585 -16.823   6.069  1.00  0.00           O
ATOM   1216  N   LEU A 124      12.309 -18.587   5.394  1.00  0.00           N
ATOM   1217  H   LEU A 124      12.306 -19.575   5.257  1.00  0.00           H
ATOM   1218  CA  LEU A 124      11.138 -17.858   4.970  1.00  0.00           C
ATOM   1219  CB  LEU A 124      10.114 -18.800   4.341  1.00  0.00           C
ATOM   1220  CG  LEU A 124      10.581 -19.508   3.066  1.00  0.00           C
ATOM   1221  CD1 LEU A 124       9.626 -20.630   2.655  1.00  0.00           C
ATOM   1222  CD2 LEU A 124      10.812 -18.534   1.914  1.00  0.00           C
ATOM   1223  C   LEU A 124      10.511 -17.093   6.109  1.00  0.00           C
ATOM   1224  O   LEU A 124       9.787 -16.121   5.876  1.00  0.00           O
ATOM   1225  N   HIS A 125      10.751 -17.546   7.363  1.00  0.00           N
ATOM   1226  H   HIS A 125      11.381 -18.310   7.513  1.00  0.00           H
ATOM   1227  CA  HIS A 125      10.229 -16.913   8.539  1.00  0.00           C
ATOM   1228  CB  HIS A 125       9.558 -17.923   9.471  1.00  0.00           C
ATOM   1229  CG  HIS A 125       8.458 -18.644   8.731  1.00  0.00           C
ATOM   1230  ND1 HIS A 125       7.313 -18.066   8.319  1.00  0.00           N
ATOM   1231  HD1 HIS A 125       7.038 -17.132   8.444  1.00  0.00           H
ATOM   1232  CD2 HIS A 125       8.442 -19.989   8.351  1.00  0.00           C
ATOM   1233  NE2 HIS A 125       7.274 -20.215   7.703  1.00  0.00           N
ATOM   1234  CE1 HIS A 125       6.578 -19.032   7.682  1.00  0.00           C
ATOM   1235  C   HIS A 125      11.278 -16.111   9.269  1.00  0.00           C
ATOM   1236  O   HIS A 125      11.035 -15.666  10.392  1.00  0.00           O
ATOM   1237  N   GLY A 126      12.475 -15.906   8.650  1.00  0.00           N
ATOM   1238  H   GLY A 126      12.564 -16.139   7.682  1.00  0.00           H
ATOM   1239  CA  GLY A 126      13.561 -15.143   9.227  1.00  0.00           C
```

```
ATOM   1240  C   GLY A 126      13.243 -13.670   9.172  1.00  0.00           C
ATOM   1241  O   GLY A 126      12.318 -13.249   8.473  1.00  0.00           O
ATOM   1242  N   THR A 127      14.030 -12.842   9.903  1.00  0.00           N
ATOM   1243  H   THR A 127      14.740 -13.189  10.513  1.00  0.00           H
ATOM   1244  CA  THR A 127      13.793 -11.420   9.953  1.00  0.00           C
ATOM   1245  CB  THR A 127      14.568 -10.952  11.171  1.00  0.00           C
ATOM   1246  OG1 THR A 127      14.451 -11.973  12.176  1.00  0.00           O
ATOM   1247  HG1 THR A 127      15.176 -11.842  12.781  1.00  0.00           H
ATOM   1248  CG2 THR A 127      14.094  -9.592  11.689  1.00  0.00           C
ATOM   1249  C   THR A 127      14.142 -10.778   8.628  1.00  0.00           C
ATOM   1250  O   THR A 127      15.250 -10.904   8.102  1.00  0.00           O
ATOM   1251  N   ILE A 128      13.138 -10.054   8.083  1.00  0.00           N
ATOM   1252  H   ILE A 128      12.271 -10.077   8.579  1.00  0.00           H
ATOM   1253  CA  ILE A 128      13.197  -9.369   6.815  1.00  0.00           C
ATOM   1254  CB  ILE A 128      11.782  -9.052   6.321  1.00  0.00           C
ATOM   1255  CG2 ILE A 128      11.770  -8.175   5.067  1.00  0.00           C
ATOM   1256  CG1 ILE A 128      11.047 -10.371   6.071  1.00  0.00           C
ATOM   1257  CD1 ILE A 128       9.677 -10.170   5.424  1.00  0.00           C
ATOM   1258  C   ILE A 128      14.085  -8.151   6.903  1.00  0.00           C
ATOM   1259  O   ILE A 128      14.751  -7.812   5.926  1.00  0.00           O
ATOM   1260  N   LEU A 129      14.132  -7.485   8.082  1.00  0.00           N
ATOM   1261  H   LEU A 129      13.586  -7.832   8.840  1.00  0.00           H
ATOM   1262  CA  LEU A 129      14.947  -6.312   8.298  1.00  0.00           C
ATOM   1263  CB  LEU A 129      14.613  -5.723   9.673  1.00  0.00           C
ATOM   1264  CG  LEU A 129      15.197  -4.341   9.998  1.00  0.00           C
ATOM   1265  CD1 LEU A 129      16.584  -4.401  10.645  1.00  0.00           C
ATOM   1266  CD2 LEU A 129      15.131  -3.398   8.795  1.00  0.00           C
ATOM   1267  C   LEU A 129      16.408  -6.648   8.142  1.00  0.00           C
ATOM   1268  O   LEU A 129      17.152  -5.852   7.569  1.00  0.00           O
ATOM   1269  N   THR A 130      16.838  -7.831   8.634  1.00  0.00           N
ATOM   1270  H   THR A 130      16.182  -8.428   9.086  1.00  0.00           H
ATOM   1271  CA  THR A 130      18.207  -8.272   8.542  1.00  0.00           C
ATOM   1272  CB  THR A 130      18.317  -9.399   9.549  1.00  0.00           C
ATOM   1273  OG1 THR A 130      16.991  -9.875   9.817  1.00  0.00           O
ATOM   1274  HG1 THR A 130      16.803 -10.520   9.136  1.00  0.00           H
ATOM   1275  CG2 THR A 130      18.964  -8.918  10.849  1.00  0.00           C
ATOM   1276  C   THR A 130      18.599  -8.656   7.136  1.00  0.00           C
ATOM   1277  O   THR A 130      19.764  -8.488   6.769  1.00  0.00           O
ATOM   1278  N   ARG A 131      17.618  -9.129   6.316  1.00  0.00           N
ATOM   1279  H   ARG A 131      16.690  -9.172   6.682  1.00  0.00           H
ATOM   1280  CA  ARG A 131      17.813  -9.568   4.947  1.00  0.00           C
ATOM   1281  CB  ARG A 131      16.554 -10.026   4.197  1.00  0.00           C
ATOM   1282  CG  ARG A 131      17.028 -10.525   2.821  1.00  0.00           C
ATOM   1283  CD  ARG A 131      16.029 -10.565   1.670  1.00  0.00           C
ATOM   1284  NE  ARG A 131      15.282  -9.319   1.553  1.00  0.00           N
ATOM   1285  HE  ARG A 131      15.795  -8.531   1.197  1.00  0.00           H
ATOM   1286  CZ  ARG A 131      13.972  -9.367   1.930  1.00  0.00           C
ATOM   1287  NH1 ARG A 131      13.467 -10.536   2.392  1.00  0.00           N
ATOM   1288 HH11 ARG A 131      14.031 -11.380   2.439  1.00  0.00           H
ATOM   1289 HH12 ARG A 131      12.525 -10.632   2.714  1.00  0.00           H
ATOM   1290  NH2 ARG A 131      13.210  -8.252   1.838  1.00  0.00           N
ATOM   1291 HH21 ARG A 131      13.592  -7.386   1.500  1.00  0.00           H
ATOM   1292 HH22 ARG A 131      12.241  -8.233   2.099  1.00  0.00           H
ATOM   1293  C   ARG A 131      18.608  -8.639   4.046  1.00  0.00           C
ATOM   1294  O   ARG A 131      19.531  -9.198   3.454  1.00  0.00           O
ATOM   1295  N   PRO A 132      18.411  -7.319   3.872  1.00  0.00           N
ATOM   1296  CD  PRO A 132      17.358  -6.518   4.493  1.00  0.00           C
ATOM   1297  CA  PRO A 132      19.220  -6.512   2.977  1.00  0.00           C
ATOM   1298  CB  PRO A 132      18.483  -5.170   2.924  1.00  0.00           C
```

ATOM	1299	CG	PRO	A	132	17.765	-5.072	4.263	1.00	0.00	C
ATOM	1300	C	PRO	A	132	20.667	-6.380	3.362	1.00	0.00	C
ATOM	1301	O	PRO	A	132	21.485	-6.171	2.466	1.00	0.00	O
ATOM	1302	N	LEU	A	133	20.993	-6.491	4.672	1.00	0.00	N
ATOM	1303	H	LEU	A	133	20.288	-6.766	5.325	1.00	0.00	H
ATOM	1304	CA	LEU	A	133	22.350	-6.414	5.151	1.00	0.00	C
ATOM	1305	CB	LEU	A	133	22.339	-6.188	6.661	1.00	0.00	C
ATOM	1306	CG	LEU	A	133	21.427	-5.029	7.072	1.00	0.00	C
ATOM	1307	CD1	LEU	A	133	21.167	-5.018	8.579	1.00	0.00	C
ATOM	1308	CD2	LEU	A	133	21.939	-3.683	6.558	1.00	0.00	C
ATOM	1309	C	LEU	A	133	23.067	-7.700	4.817	1.00	0.00	C
ATOM	1310	O	LEU	A	133	24.216	-7.677	4.372	1.00	0.00	O
ATOM	1311	N	LEU	A	134	22.362	-8.844	4.999	1.00	0.00	N
ATOM	1312	H	LEU	A	134	21.415	-8.754	5.308	1.00	0.00	H
ATOM	1313	CA	LEU	A	134	22.856	-10.176	4.747	1.00	0.00	C
ATOM	1314	CB	LEU	A	134	22.064	-11.155	5.597	1.00	0.00	C
ATOM	1315	CG	LEU	A	134	22.162	-10.767	7.072	1.00	0.00	C
ATOM	1316	CD1	LEU	A	134	21.285	-11.651	7.957	1.00	0.00	C
ATOM	1317	CD2	LEU	A	134	23.616	-10.713	7.548	1.00	0.00	C
ATOM	1318	C	LEU	A	134	22.972	-10.483	3.277	1.00	0.00	C
ATOM	1319	O	LEU	A	134	23.878	-11.214	2.868	1.00	0.00	O
ATOM	1320	N	GLU	A	135	22.047	-9.905	2.464	1.00	0.00	N
ATOM	1321	H	GLU	A	135	21.386	-9.302	2.903	1.00	0.00	H
ATOM	1322	CA	GLU	A	135	21.879	-10.089	1.037	1.00	0.00	C
ATOM	1323	CB	GLU	A	135	23.065	-9.608	0.193	1.00	0.00	C
ATOM	1324	CG	GLU	A	135	23.195	-8.083	0.169	1.00	0.00	C
ATOM	1325	CD	GLU	A	135	24.009	-7.658	-1.040	1.00	0.00	C
ATOM	1326	OE1	GLU	A	135	23.410	-7.371	-2.077	1.00	0.00	O
ATOM	1327	OE2	GLU	A	135	25.235	-7.605	-0.946	1.00	0.00	O
ATOM	1328	C	GLU	A	135	21.553	-11.542	0.775	1.00	0.00	C
ATOM	1329	O	GLU	A	135	21.965	-12.136	-0.225	1.00	0.00	O
ATOM	1330	N	SER	A	136	20.773	-12.136	1.712	1.00	0.00	N
ATOM	1331	H	SER	A	136	20.384	-11.588	2.451	1.00	0.00	H
ATOM	1332	CA	SER	A	136	20.362	-13.505	1.657	1.00	0.00	C
ATOM	1333	CB	SER	A	136	19.959	-13.898	3.054	1.00	0.00	C
ATOM	1334	OG	SER	A	136	20.857	-13.203	3.907	1.00	0.00	O
ATOM	1335	HG	SER	A	136	21.724	-13.382	3.556	1.00	0.00	H
ATOM	1336	C	SER	A	136	19.251	-13.696	0.675	1.00	0.00	C
ATOM	1337	O	SER	A	136	18.255	-12.969	0.661	1.00	0.00	O
ATOM	1338	N	GLU	A	137	19.457	-14.703	-0.190	1.00	0.00	N
ATOM	1339	H	GLU	A	137	20.259	-15.286	-0.039	1.00	0.00	H
ATOM	1340	CA	GLU	A	137	18.528	-15.108	-1.206	1.00	0.00	C
ATOM	1341	CB	GLU	A	137	19.211	-15.929	-2.314	1.00	0.00	C
ATOM	1342	CG	GLU	A	137	20.738	-16.136	-2.259	1.00	0.00	C
ATOM	1343	CD	GLU	A	137	21.212	-17.051	-1.132	1.00	0.00	C
ATOM	1344	OE1	GLU	A	137	22.314	-17.569	-1.235	1.00	0.00	O
ATOM	1345	OE2	GLU	A	137	20.519	-17.223	-0.134	1.00	0.00	O
ATOM	1346	C	GLU	A	137	17.434	-15.931	-0.576	1.00	0.00	C
ATOM	1347	O	GLU	A	137	16.302	-15.942	-1.059	1.00	0.00	O
ATOM	1348	N	LEU	A	138	17.778	-16.653	0.515	1.00	0.00	N
ATOM	1349	H	LEU	A	138	18.765	-16.723	0.674	1.00	0.00	H
ATOM	1350	CA	LEU	A	138	16.894	-17.508	1.253	1.00	0.00	C
ATOM	1351	CB	LEU	A	138	17.689	-18.498	2.103	1.00	0.00	C
ATOM	1352	CG	LEU	A	138	18.544	-19.459	1.277	1.00	0.00	C
ATOM	1353	CD1	LEU	A	138	19.293	-20.447	2.170	1.00	0.00	C
ATOM	1354	CD2	LEU	A	138	17.735	-20.169	0.189	1.00	0.00	C
ATOM	1355	C	LEU	A	138	15.880	-16.805	2.102	1.00	0.00	C
ATOM	1356	O	LEU	A	138	14.719	-17.217	2.121	1.00	0.00	O
ATOM	1357	N	VAL	A	139	16.268	-15.711	2.797	1.00	0.00	N

```
ATOM   1358  H   VAL A 139      17.153 -15.305   2.580  1.00  0.00           H
ATOM   1359  CA  VAL A 139      15.338 -15.046   3.675  1.00  0.00           C
ATOM   1360  CB  VAL A 139      16.062 -14.251   4.763  1.00  0.00           C
ATOM   1361  CG1 VAL A 139      15.069 -13.596   5.729  1.00  0.00           C
ATOM   1362  CG2 VAL A 139      17.056 -15.148   5.502  1.00  0.00           C
ATOM   1363  C   VAL A 139      14.407 -14.169   2.883  1.00  0.00           C
ATOM   1364  O   VAL A 139      14.675 -12.998   2.625  1.00  0.00           O
ATOM   1365  N   ILE A 140      13.270 -14.763   2.457  1.00  0.00           N
ATOM   1366  H   ILE A 140      13.089 -15.730   2.662  1.00  0.00           H
ATOM   1367  CA  ILE A 140      12.254 -14.050   1.726  1.00  0.00           C
ATOM   1368  CB  ILE A 140      12.109 -14.567   0.304  1.00  0.00           C
ATOM   1369  CG2 ILE A 140      11.003 -13.772  -0.368  1.00  0.00           C
ATOM   1370  CG1 ILE A 140      13.404 -14.495  -0.499  1.00  0.00           C
ATOM   1371  CD1 ILE A 140      13.206 -15.012  -1.925  1.00  0.00           C
ATOM   1372  C   ILE A 140      11.031 -14.405   2.527  1.00  0.00           C
ATOM   1373  O   ILE A 140      10.786 -15.583   2.788  1.00  0.00           O
ATOM   1374  N   GLY A 141      10.224 -13.392   2.911  1.00  0.00           N
ATOM   1375  H   GLY A 141      10.367 -12.489   2.522  1.00  0.00           H
ATOM   1376  CA  GLY A 141       9.052 -13.607   3.722  1.00  0.00           C
ATOM   1377  C   GLY A 141       7.986 -14.460   3.103  1.00  0.00           C
ATOM   1378  O   GLY A 141       7.661 -14.326   1.921  1.00  0.00           O
ATOM   1379  N   ALA A 142       7.448 -15.377   3.942  1.00  0.00           N
ATOM   1380  H   ALA A 142       7.800 -15.481   4.875  1.00  0.00           H
ATOM   1381  CA  ALA A 142       6.379 -16.260   3.557  1.00  0.00           C
ATOM   1382  CB  ALA A 142       6.835 -17.710   3.430  1.00  0.00           C
ATOM   1383  C   ALA A 142       5.289 -16.179   4.574  1.00  0.00           C
ATOM   1384  O   ALA A 142       5.560 -16.145   5.780  1.00  0.00           O
ATOM   1385  N   VAL A 143       4.024 -16.117   4.080  1.00  0.00           N
ATOM   1386  H   VAL A 143       3.859 -16.213   3.093  1.00  0.00           H
ATOM   1387  CA  VAL A 143       2.851 -16.061   4.942  1.00  0.00           C
ATOM   1388  CB  VAL A 143       2.341 -14.646   5.237  1.00  0.00           C
ATOM   1389  CG1 VAL A 143       3.354 -13.798   5.991  1.00  0.00           C
ATOM   1390  CG2 VAL A 143       1.811 -13.983   3.972  1.00  0.00           C
ATOM   1391  C   VAL A 143       1.713 -16.843   4.357  1.00  0.00           C
ATOM   1392  O   VAL A 143       1.724 -17.194   3.177  1.00  0.00           O
ATOM   1393  N   ILE A 144       0.717 -17.161   5.219  1.00  0.00           N
ATOM   1394  H   ILE A 144       0.762 -16.779   6.141  1.00  0.00           H
ATOM   1395  CA  ILE A 144      -0.463 -17.871   4.817  1.00  0.00           C
ATOM   1396  CB  ILE A 144      -1.058 -18.590   6.019  1.00  0.00           C
ATOM   1397  CG2 ILE A 144      -2.462 -19.114   5.712  1.00  0.00           C
ATOM   1398  CG1 ILE A 144      -0.084 -19.652   6.531  1.00  0.00           C
ATOM   1399  CD1 ILE A 144      -0.228 -19.943   8.023  1.00  0.00           C
ATOM   1400  C   ILE A 144      -1.429 -16.825   4.307  1.00  0.00           C
ATOM   1401  O   ILE A 144      -1.847 -15.925   5.043  1.00  0.00           O
ATOM   1402  N   LEU A 145      -1.764 -16.928   3.005  1.00  0.00           N
ATOM   1403  H   LEU A 145      -1.281 -17.632   2.483  1.00  0.00           H
ATOM   1404  CA  LEU A 145      -2.714 -16.068   2.355  1.00  0.00           C
ATOM   1405  CB  LEU A 145      -2.055 -15.122   1.350  1.00  0.00           C
ATOM   1406  CG  LEU A 145      -1.311 -13.966   2.018  1.00  0.00           C
ATOM   1407  CD1 LEU A 145      -0.635 -13.052   0.997  1.00  0.00           C
ATOM   1408  CD2 LEU A 145      -2.220 -13.181   2.963  1.00  0.00           C
ATOM   1409  C   LEU A 145      -3.697 -16.967   1.684  1.00  0.00           C
ATOM   1410  O   LEU A 145      -3.315 -17.845   0.907  1.00  0.00           O
ATOM   1411  N   ARG A 146      -4.998 -16.752   1.993  1.00  0.00           N
ATOM   1412  H   ARG A 146      -5.161 -16.006   2.641  1.00  0.00           H
ATOM   1413  CA  ARG A 146      -6.133 -17.492   1.481  1.00  0.00           C
ATOM   1414  CB  ARG A 146      -6.472 -17.071   0.052  1.00  0.00           C
ATOM   1415  CG  ARG A 146      -6.905 -15.603   0.068  1.00  0.00           C
ATOM   1416  CD  ARG A 146      -7.356 -15.075  -1.289  1.00  0.00           C
```

```
ATOM   1417  NE  ARG A 146      -8.547 -15.772  -1.776  1.00  0.00           N
ATOM   1418  HE  ARG A 146      -8.879 -16.558  -1.244  1.00  0.00           H
ATOM   1419  CZ  ARG A 146      -9.113 -15.335  -2.939  1.00  0.00           C
ATOM   1420  NH1 ARG A 146      -8.612 -14.245  -3.527  1.00  0.00           N
ATOM   1421 HH11 ARG A 146      -7.863 -13.690  -3.164  1.00  0.00           H
ATOM   1422 HH12 ARG A 146      -8.920 -13.880  -4.419  1.00  0.00           H
ATOM   1423  NH2 ARG A 146     -10.152 -15.988  -3.498  1.00  0.00           N
ATOM   1424 HH21 ARG A 146     -10.568 -16.800  -3.081  1.00  0.00           H
ATOM   1425 HH22 ARG A 146     -10.545 -15.675  -4.370  1.00  0.00           H
ATOM   1426  C   ARG A 146      -6.018 -18.987   1.689  1.00  0.00           C
ATOM   1427  O   ARG A 146      -6.414 -19.797   0.846  1.00  0.00           O
ATOM   1428  N   GLY A 147      -5.453 -19.366   2.858  1.00  0.00           N
ATOM   1429  H   GLY A 147      -5.179 -18.649   3.497  1.00  0.00           H
ATOM   1430  CA  GLY A 147      -5.267 -20.733   3.261  1.00  0.00           C
ATOM   1431  C   GLY A 147      -4.074 -21.401   2.639  1.00  0.00           C
ATOM   1432  O   GLY A 147      -3.903 -22.609   2.814  1.00  0.00           O
ATOM   1433  N   HIS A 148      -3.226 -20.646   1.900  1.00  0.00           N
ATOM   1434  H   HIS A 148      -3.345 -19.656   1.846  1.00  0.00           H
ATOM   1435  CA  HIS A 148      -2.071 -21.219   1.261  1.00  0.00           C
ATOM   1436  CB  HIS A 148      -2.276 -21.219  -0.257  1.00  0.00           C
ATOM   1437  CG  HIS A 148      -3.413 -22.145  -0.632  1.00  0.00           C
ATOM   1438  ND1 HIS A 148      -4.723 -21.910  -0.394  1.00  0.00           N
ATOM   1439  HD1 HIS A 148      -5.141 -21.131   0.043  1.00  0.00           H
ATOM   1440  CD2 HIS A 148      -3.291 -23.395  -1.242  1.00  0.00           C
ATOM   1441  NE2 HIS A 148      -4.537 -23.917  -1.361  1.00  0.00           N
ATOM   1442  CE1 HIS A 148      -5.420 -23.002  -0.843  1.00  0.00           C
ATOM   1443  C   HIS A 148      -0.825 -20.480   1.642  1.00  0.00           C
ATOM   1444  O   HIS A 148      -0.815 -19.250   1.695  1.00  0.00           O
ATOM   1445  N   LEU A 149       0.267 -21.232   1.931  1.00  0.00           N
ATOM   1446  H   LEU A 149       0.191 -22.223   1.863  1.00  0.00           H
ATOM   1447  CA  LEU A 149       1.562 -20.667   2.249  1.00  0.00           C
ATOM   1448  CB  LEU A 149       2.507 -21.762   2.740  1.00  0.00           C
ATOM   1449  CG  LEU A 149       2.399 -22.051   4.235  1.00  0.00           C
ATOM   1450  CD1 LEU A 149       2.935 -23.440   4.586  1.00  0.00           C
ATOM   1451  CD2 LEU A 149       3.082 -20.955   5.057  1.00  0.00           C
ATOM   1452  C   LEU A 149       2.060 -20.101   0.946  1.00  0.00           C
ATOM   1453  O   LEU A 149       2.040 -20.799  -0.070  1.00  0.00           O
ATOM   1454  N   ARG A 150       2.484 -18.818   0.941  1.00  0.00           N
ATOM   1455  H   ARG A 150       2.498 -18.256   1.771  1.00  0.00           H
ATOM   1456  CA  ARG A 150       2.974 -18.177  -0.252  1.00  0.00           C
ATOM   1457  CB  ARG A 150       1.974 -17.189  -0.839  1.00  0.00           C
ATOM   1458  CG  ARG A 150       0.641 -17.863  -1.114  1.00  0.00           C
ATOM   1459  CD  ARG A 150      -0.398 -16.921  -1.695  1.00  0.00           C
ATOM   1460  NE  ARG A 150      -1.715 -17.449  -1.366  1.00  0.00           N
ATOM   1461  HE  ARG A 150      -1.943 -17.468  -0.388  1.00  0.00           H
ATOM   1462  CZ  ARG A 150      -2.535 -18.006  -2.271  1.00  0.00           C
ATOM   1463  NH1 ARG A 150      -2.190 -18.065  -3.558  1.00  0.00           N
ATOM   1464 HH11 ARG A 150      -1.304 -17.697  -3.849  1.00  0.00           H
ATOM   1465 HH12 ARG A 150      -2.780 -18.472  -4.255  1.00  0.00           H
ATOM   1466  NH2 ARG A 150      -3.689 -18.501  -1.836  1.00  0.00           N
ATOM   1467 HH21 ARG A 150      -3.881 -18.439  -0.850  1.00  0.00           H
ATOM   1468 HH22 ARG A 150      -4.361 -18.934  -2.437  1.00  0.00           H
ATOM   1469  C   ARG A 150       4.261 -17.456   0.048  1.00  0.00           C
ATOM   1470  O   ARG A 150       4.368 -16.800   1.089  1.00  0.00           O
ATOM   1471  N   ILE A 151       5.269 -17.625  -0.856  1.00  0.00           N
ATOM   1472  H   ILE A 151       5.031 -18.138  -1.685  1.00  0.00           H
ATOM   1473  CA  ILE A 151       6.573 -16.972  -0.784  1.00  0.00           C
ATOM   1474  CB  ILE A 151       7.682 -17.760  -1.494  1.00  0.00           C
ATOM   1475  CG2 ILE A 151       8.985 -16.961  -1.518  1.00  0.00           C
```

ATOM	1476	CG1	ILE	A	151	7.930	-19.122	-0.863	1.00	0.00	C
ATOM	1477	CD1	ILE	A	151	8.995	-19.917	-1.622	1.00	0.00	C
ATOM	1478	C	ILE	A	151	6.365	-15.670	-1.521	1.00	0.00	C
ATOM	1479	O	ILE	A	151	5.881	-15.675	-2.655	1.00	0.00	O
ATOM	1480	N	ALA	A	152	6.784	-14.540	-0.909	1.00	0.00	N
ATOM	1481	H	ALA	A	152	7.142	-14.615	0.026	1.00	0.00	H
ATOM	1482	CA	ALA	A	152	6.614	-13.231	-1.492	1.00	0.00	C
ATOM	1483	CB	ALA	A	152	6.946	-12.148	-0.466	1.00	0.00	C
ATOM	1484	C	ALA	A	152	7.433	-12.958	-2.715	1.00	0.00	C
ATOM	1485	O	ALA	A	152	6.903	-12.450	-3.704	1.00	0.00	O
ATOM	1486	N	GLY	A	153	8.731	-13.325	-2.680	1.00	0.00	N
ATOM	1487	H	GLY	A	153	9.031	-13.842	-1.883	1.00	0.00	H
ATOM	1488	CA	GLY	A	153	9.640	-13.080	-3.766	1.00	0.00	C
ATOM	1489	C	GLY	A	153	9.441	-13.962	-4.950	1.00	0.00	C
ATOM	1490	O	GLY	A	153	9.628	-13.508	-6.078	1.00	0.00	O
ATOM	1491	N	HIS	A	154	9.066	-15.238	-4.714	1.00	0.00	N
ATOM	1492	H	HIS	A	154	8.856	-15.521	-3.783	1.00	0.00	H
ATOM	1493	CA	HIS	A	154	8.883	-16.181	-5.786	1.00	0.00	C
ATOM	1494	CB	HIS	A	154	9.494	-17.533	-5.421	1.00	0.00	C
ATOM	1495	CG	HIS	A	154	10.979	-17.448	-5.660	1.00	0.00	C
ATOM	1496	ND1	HIS	A	154	11.501	-17.112	-6.851	1.00	0.00	N
ATOM	1497	HD1	HIS	A	154	10.979	-16.936	-7.669	1.00	0.00	H
ATOM	1498	CD2	HIS	A	154	12.017	-17.659	-4.750	1.00	0.00	C
ATOM	1499	NE2	HIS	A	154	13.177	-17.439	-5.417	1.00	0.00	N
ATOM	1500	CE1	HIS	A	154	12.862	-17.100	-6.708	1.00	0.00	C
ATOM	1501	C	HIS	A	154	7.471	-16.344	-6.245	1.00	0.00	C
ATOM	1502	O	HIS	A	154	7.253	-16.839	-7.353	1.00	0.00	O
ATOM	1503	N	HIS	A	155	6.483	-15.896	-5.423	1.00	0.00	N
ATOM	1504	H	HIS	A	155	6.716	-15.492	-4.540	1.00	0.00	H
ATOM	1505	CA	HIS	A	155	5.063	-16.006	-5.692	1.00	0.00	C
ATOM	1506	CB	HIS	A	155	4.636	-15.129	-6.873	1.00	0.00	C
ATOM	1507	CG	HIS	A	155	4.755	-13.670	-6.500	1.00	0.00	C
ATOM	1508	ND1	HIS	A	155	3.968	-13.070	-5.587	1.00	0.00	N
ATOM	1509	HD1	HIS	A	155	3.262	-13.493	-5.060	1.00	0.00	H
ATOM	1510	CD2	HIS	A	155	5.650	-12.725	-7.011	1.00	0.00	C
ATOM	1511	NE2	HIS	A	155	5.387	-11.546	-6.391	1.00	0.00	N
ATOM	1512	CE1	HIS	A	155	4.354	-11.759	-5.516	1.00	0.00	C
ATOM	1513	C	HIS	A	155	4.633	-17.444	-5.886	1.00	0.00	C
ATOM	1514	O	HIS	A	155	3.764	-17.762	-6.704	1.00	0.00	O
ATOM	1515	N	LEU	A	156	5.262	-18.349	-5.097	1.00	0.00	N
ATOM	1516	H	LEU	A	156	5.838	-18.012	-4.356	1.00	0.00	H
ATOM	1517	CA	LEU	A	156	4.989	-19.759	-5.130	1.00	0.00	C
ATOM	1518	CB	LEU	A	156	6.278	-20.563	-5.018	1.00	0.00	C
ATOM	1519	CG	LEU	A	156	7.176	-20.447	-6.241	1.00	0.00	C
ATOM	1520	CD1	LEU	A	156	8.420	-21.317	-6.075	1.00	0.00	C
ATOM	1521	CD2	LEU	A	156	6.415	-20.750	-7.532	1.00	0.00	C
ATOM	1522	C	LEU	A	156	4.103	-20.165	-4.012	1.00	0.00	C
ATOM	1523	O	LEU	A	156	4.157	-19.579	-2.928	1.00	0.00	O
ATOM	1524	N	GLY	A	157	3.275	-21.193	-4.306	1.00	0.00	N
ATOM	1525	H	GLY	A	157	3.537	-21.755	-5.097	1.00	0.00	H
ATOM	1526	CA	GLY	A	157	2.309	-21.766	-3.419	1.00	0.00	C
ATOM	1527	C	GLY	A	157	2.809	-22.852	-2.514	1.00	0.00	C
ATOM	1528	O	GLY	A	157	3.931	-23.358	-2.611	1.00	0.00	O
ATOM	1529	N	ARG	A	158	1.850	-23.284	-1.670	1.00	0.00	N
ATOM	1530	H	ARG	A	158	1.039	-22.706	-1.705	1.00	0.00	H
ATOM	1531	CA	ARG	A	158	1.921	-24.269	-0.621	1.00	0.00	C
ATOM	1532	CB	ARG	A	158	0.564	-24.425	0.076	1.00	0.00	C
ATOM	1533	CG	ARG	A	158	0.638	-25.271	1.355	1.00	0.00	C
ATOM	1534	CD	ARG	A	158	-0.727	-25.579	1.973	1.00	0.00	C

```
ATOM   1535  NE  ARG A 158      -1.571 -26.330   1.042  1.00  0.00           N
ATOM   1536  HE  ARG A 158      -1.147 -27.095   0.555  1.00  0.00           H
ATOM   1537  CZ  ARG A 158      -2.865 -25.981   0.863  1.00  0.00           C
ATOM   1538  NH1 ARG A 158      -3.415 -25.025   1.607  1.00  0.00           N
ATOM   1539 HH11 ARG A 158      -2.938 -24.638   2.411  1.00  0.00           H
ATOM   1540 HH12 ARG A 158      -4.325 -24.654   1.431  1.00  0.00           H
ATOM   1541  NH2 ARG A 158      -3.585 -26.586  -0.081  1.00  0.00           N
ATOM   1542 HH21 ARG A 158      -3.199 -27.329  -0.629  1.00  0.00           H
ATOM   1543 HH22 ARG A 158      -4.527 -26.302  -0.271  1.00  0.00           H
ATOM   1544  C   ARG A 158       2.422 -25.603  -1.091  1.00  0.00           C
ATOM   1545  O   ARG A 158       3.199 -26.244  -0.381  1.00  0.00           O
ATOM   1546  N   CYS A 159       2.034 -26.015  -2.319  1.00  0.00           N
ATOM   1547  H   CYS A 159       1.537 -25.393  -2.921  1.00  0.00           H
ATOM   1548  CA  CYS A 159       2.427 -27.277  -2.891  1.00  0.00           C
ATOM   1549  CB  CYS A 159       1.604 -27.480  -4.156  1.00  0.00           C
ATOM   1550  SG  CYS A 159      -0.123 -27.040  -3.811  1.00  0.00           S
ATOM   1551  C   CYS A 159       3.908 -27.348  -3.139  1.00  0.00           C
ATOM   1552  O   CYS A 159       4.496 -28.425  -3.021  1.00  0.00           O
ATOM   1553  N   ASP A 160       4.528 -26.204  -3.509  1.00  0.00           N
ATOM   1554  H   ASP A 160       4.027 -25.349  -3.659  1.00  0.00           H
ATOM   1555  CA  ASP A 160       5.939 -26.128  -3.774  1.00  0.00           C
ATOM   1556  CB  ASP A 160       6.212 -24.975  -4.753  1.00  0.00           C
ATOM   1557  CG  ASP A 160       4.962 -24.608  -5.549  1.00  0.00           C
ATOM   1558  OD1 ASP A 160       4.383 -25.471  -6.205  1.00  0.00           O
ATOM   1559  OD2 ASP A 160       4.548 -23.452  -5.495  1.00  0.00           O
ATOM   1560  C   ASP A 160       6.727 -25.953  -2.498  1.00  0.00           C
ATOM   1561  O   ASP A 160       7.765 -26.591  -2.312  1.00  0.00           O
ATOM   1562  N   ILE A 161       6.211 -25.091  -1.584  1.00  0.00           N
ATOM   1563  H   ILE A 161       5.371 -24.623  -1.870  1.00  0.00           H
ATOM   1564  CA  ILE A 161       6.843 -24.736  -0.330  1.00  0.00           C
ATOM   1565  CB  ILE A 161       6.078 -23.605   0.352  1.00  0.00           C
ATOM   1566  CG2 ILE A 161       6.633 -23.282   1.741  1.00  0.00           C
ATOM   1567  CG1 ILE A 161       6.071 -22.372  -0.537  1.00  0.00           C
ATOM   1568  CD1 ILE A 161       5.244 -21.265   0.100  1.00  0.00           C
ATOM   1569  C   ILE A 161       6.960 -25.899   0.612  1.00  0.00           C
ATOM   1570  O   ILE A 161       8.034 -26.082   1.182  1.00  0.00           O
ATOM   1571  N   LYS A 162       5.901 -26.732   0.756  1.00  0.00           N
ATOM   1572  H   LYS A 162       5.086 -26.583   0.193  1.00  0.00           H
ATOM   1573  CA  LYS A 162       5.925 -27.856   1.665  1.00  0.00           C
ATOM   1574  CB  LYS A 162       4.544 -28.450   1.966  1.00  0.00           C
ATOM   1575  CG  LYS A 162       4.342 -28.347   3.484  1.00  0.00           C
ATOM   1576  CD  LYS A 162       3.112 -29.030   4.093  1.00  0.00           C
ATOM   1577  CE  LYS A 162       3.072 -28.730   5.600  1.00  0.00           C
ATOM   1578  NZ  LYS A 162       2.006 -29.469   6.285  1.00  0.00           N
ATOM   1579  HZ1 LYS A 162       1.056 -29.280   5.911  1.00  0.00           H
ATOM   1580  HZ2 LYS A 162       2.177 -30.501   6.320  1.00  0.00           H
ATOM   1581  HZ3 LYS A 162       1.930 -29.208   7.292  1.00  0.00           H
ATOM   1582  C   LYS A 162       6.970 -28.868   1.333  1.00  0.00           C
ATOM   1583  O   LYS A 162       7.492 -29.515   2.244  1.00  0.00           O
ATOM   1584  N   ASP A 163       7.300 -29.009   0.028  1.00  0.00           N
ATOM   1585  H   ASP A 163       6.883 -28.438  -0.676  1.00  0.00           H
ATOM   1586  CA  ASP A 163       8.283 -29.954  -0.423  1.00  0.00           C
ATOM   1587  CB  ASP A 163       8.232 -30.066  -1.948  1.00  0.00           C
ATOM   1588  CG  ASP A 163       8.292 -31.523  -2.368  1.00  0.00           C
ATOM   1589  OD1 ASP A 163       9.166 -31.875  -3.160  1.00  0.00           O
ATOM   1590  OD2 ASP A 163       7.449 -32.300  -1.918  1.00  0.00           O
ATOM   1591  C   ASP A 163       9.654 -29.568   0.058  1.00  0.00           C
ATOM   1592  O   ASP A 163      10.343 -30.413   0.635  1.00  0.00           O
ATOM   1593  N   LEU A 164      10.067 -28.283  -0.125  1.00  0.00           N
```

ATOM	1594	H	LEU	A	164	9.462	-27.617	-0.561	1.00	0.00	H
ATOM	1595	CA	LEU	A	164	11.377	-27.851	0.319	1.00	0.00	C
ATOM	1596	CB	LEU	A	164	12.288	-27.656	-0.895	1.00	0.00	C
ATOM	1597	CG	LEU	A	164	12.493	-28.883	-1.786	1.00	0.00	C
ATOM	1598	CD1	LEU	A	164	13.180	-28.503	-3.098	1.00	0.00	C
ATOM	1599	CD2	LEU	A	164	13.233	-30.011	-1.064	1.00	0.00	C
ATOM	1600	C	LEU	A	164	11.300	-26.517	1.042	1.00	0.00	C
ATOM	1601	O	LEU	A	164	11.701	-25.500	0.466	1.00	0.00	O
ATOM	1602	N	PRO	A	165	10.796	-26.432	2.276	1.00	0.00	N
ATOM	1603	CD	PRO	A	165	10.290	-27.570	3.043	1.00	0.00	C
ATOM	1604	CA	PRO	A	165	10.710	-25.180	3.002	1.00	0.00	C
ATOM	1605	CB	PRO	A	165	9.763	-25.533	4.154	1.00	0.00	C
ATOM	1606	CG	PRO	A	165	10.013	-27.013	4.433	1.00	0.00	C
ATOM	1607	C	PRO	A	165	12.056	-24.667	3.441	1.00	0.00	C
ATOM	1608	O	PRO	A	165	12.224	-23.449	3.497	1.00	0.00	O
ATOM	1609	N	LYS	A	166	13.003	-25.578	3.765	1.00	0.00	N
ATOM	1610	H	LYS	A	166	12.866	-26.532	3.494	1.00	0.00	H
ATOM	1611	CA	LYS	A	166	14.316	-25.222	4.230	1.00	0.00	C
ATOM	1612	CB	LYS	A	166	14.981	-26.409	4.920	1.00	0.00	C
ATOM	1613	CG	LYS	A	166	14.253	-26.936	6.152	1.00	0.00	C
ATOM	1614	CD	LYS	A	166	14.910	-28.224	6.649	1.00	0.00	C
ATOM	1615	CE	LYS	A	166	14.337	-28.642	7.995	1.00	0.00	C
ATOM	1616	NZ	LYS	A	166	14.483	-27.491	8.889	1.00	0.00	N
ATOM	1617	HZ1	LYS	A	166	14.573	-27.783	9.885	1.00	0.00	H
ATOM	1618	HZ2	LYS	A	166	15.329	-26.937	8.658	1.00	0.00	H
ATOM	1619	HZ3	LYS	A	166	13.651	-26.867	8.834	1.00	0.00	H
ATOM	1620	C	LYS	A	166	15.171	-24.769	3.086	1.00	0.00	C
ATOM	1621	O	LYS	A	166	15.869	-23.761	3.207	1.00	0.00	O
ATOM	1622	N	GLU	A	167	15.108	-25.505	1.947	1.00	0.00	N
ATOM	1623	H	GLU	A	167	14.530	-26.318	1.930	1.00	0.00	H
ATOM	1624	CA	GLU	A	167	15.876	-25.199	0.767	1.00	0.00	C
ATOM	1625	CB	GLU	A	167	15.874	-26.460	-0.113	1.00	0.00	C
ATOM	1626	CG	GLU	A	167	16.411	-27.765	0.520	1.00	0.00	C
ATOM	1627	CD	GLU	A	167	15.612	-28.255	1.725	1.00	0.00	C
ATOM	1628	OE1	GLU	A	167	14.379	-28.212	1.702	1.00	0.00	O
ATOM	1629	OE2	GLU	A	167	16.231	-28.629	2.716	1.00	0.00	O
ATOM	1630	C	GLU	A	167	15.330	-24.009	0.039	1.00	0.00	C
ATOM	1631	O	GLU	A	167	16.109	-23.232	-0.515	1.00	0.00	O
ATOM	1632	N	ILE	A	168	13.980	-23.842	0.050	1.00	0.00	N
ATOM	1633	H	ILE	A	168	13.441	-24.601	0.413	1.00	0.00	H
ATOM	1634	CA	ILE	A	168	13.227	-22.776	-0.574	1.00	0.00	C
ATOM	1635	CB	ILE	A	168	13.728	-21.410	-0.133	1.00	0.00	C
ATOM	1636	CG2	ILE	A	168	13.037	-20.247	-0.849	1.00	0.00	C
ATOM	1637	CG1	ILE	A	168	13.535	-21.374	1.380	1.00	0.00	C
ATOM	1638	CD1	ILE	A	168	14.276	-20.240	2.056	1.00	0.00	C
ATOM	1639	C	ILE	A	168	13.220	-22.984	-2.055	1.00	0.00	C
ATOM	1640	O	ILE	A	168	14.228	-22.807	-2.742	1.00	0.00	O
ATOM	1641	N	THR	A	169	12.040	-23.424	-2.547	1.00	0.00	N
ATOM	1642	H	THR	A	169	11.279	-23.612	-1.927	1.00	0.00	H
ATOM	1643	CA	THR	A	169	11.793	-23.707	-3.936	1.00	0.00	C
ATOM	1644	CB	THR	A	169	10.414	-24.351	-3.931	1.00	0.00	C
ATOM	1645	OG1	THR	A	169	10.241	-25.012	-2.666	1.00	0.00	O
ATOM	1646	HG1	THR	A	169	9.488	-25.598	-2.744	1.00	0.00	H
ATOM	1647	CG2	THR	A	169	10.214	-25.314	-5.102	1.00	0.00	C
ATOM	1648	C	THR	A	169	11.882	-22.395	-4.683	1.00	0.00	C
ATOM	1649	O	THR	A	169	11.261	-21.399	-4.305	1.00	0.00	O
ATOM	1650	N	VAL	A	170	12.724	-22.390	-5.740	1.00	0.00	N
ATOM	1651	H	VAL	A	170	13.147	-23.247	-6.028	1.00	0.00	H
ATOM	1652	CA	VAL	A	170	12.970	-21.240	-6.568	1.00	0.00	C

ATOM	1653	CB	VAL	A	170	14.481	-21.066	-6.750	1.00	0.00	C
ATOM	1654	CG1	VAL	A	170	14.828	-19.983	-7.773	1.00	0.00	C
ATOM	1655	CG2	VAL	A	170	15.149	-20.815	-5.396	1.00	0.00	C
ATOM	1656	C	VAL	A	170	12.278	-21.390	-7.896	1.00	0.00	C
ATOM	1657	O	VAL	A	170	12.413	-22.415	-8.570	1.00	0.00	O
ATOM	1658	N	ALA	A	171	11.486	-20.354	-8.257	1.00	0.00	N
ATOM	1659	H	ALA	A	171	11.322	-19.578	-7.643	1.00	0.00	H
ATOM	1660	CA	ALA	A	171	10.781	-20.276	-9.512	1.00	0.00	C
ATOM	1661	CB	ALA	A	171	9.574	-21.213	-9.572	1.00	0.00	C
ATOM	1662	C	ALA	A	171	10.324	-18.856	-9.667	1.00	0.00	C
ATOM	1663	O	ALA	A	171	9.854	-18.242	-8.706	1.00	0.00	O
ATOM	1664	N	THR	A	172	10.481	-18.301	-10.890	1.00	0.00	N
ATOM	1665	H	THR	A	172	10.644	-18.880	-11.686	1.00	0.00	H
ATOM	1666	CA	THR	A	172	10.084	-16.947	-11.187	1.00	0.00	C
ATOM	1667	CB	THR	A	172	11.208	-16.333	-12.002	1.00	0.00	C
ATOM	1668	OG1	THR	A	172	12.456	-16.656	-11.371	1.00	0.00	O
ATOM	1669	HG1	THR	A	172	12.504	-17.600	-11.328	1.00	0.00	H
ATOM	1670	CG2	THR	A	172	11.046	-14.820	-12.175	1.00	0.00	C
ATOM	1671	C	THR	A	172	8.753	-16.996	-11.904	1.00	0.00	C
ATOM	1672	O	THR	A	172	8.562	-17.801	-12.819	1.00	0.00	O
ATOM	1673	N	SER	A	173	7.803	-16.133	-11.472	1.00	0.00	N
ATOM	1674	H	SER	A	173	7.913	-15.600	-10.635	1.00	0.00	H
ATOM	1675	CA	SER	A	173	6.491	-16.044	-12.057	1.00	0.00	C
ATOM	1676	CB	SER	A	173	5.698	-16.700	-10.943	1.00	0.00	C
ATOM	1677	OG	SER	A	173	6.696	-17.323	-10.113	1.00	0.00	O
ATOM	1678	HG	SER	A	173	6.498	-17.141	-9.194	1.00	0.00	H
ATOM	1679	C	SER	A	173	6.221	-14.581	-12.275	1.00	0.00	C
ATOM	1680	O	SER	A	173	6.277	-13.790	-11.328	1.00	0.00	O
ATOM	1681	N	ARG	A	174	5.952	-14.183	-13.543	1.00	0.00	N
ATOM	1682	H	ARG	A	174	5.831	-14.838	-14.290	1.00	0.00	H
ATOM	1683	CA	ARG	A	174	5.659	-12.809	-13.855	1.00	0.00	C
ATOM	1684	CB	ARG	A	174	6.845	-12.158	-14.566	1.00	0.00	C
ATOM	1685	CG	ARG	A	174	7.725	-11.311	-13.653	1.00	0.00	C
ATOM	1686	CD	ARG	A	174	8.862	-10.639	-14.422	1.00	0.00	C
ATOM	1687	NE	ARG	A	174	9.522	-9.653	-13.572	1.00	0.00	N
ATOM	1688	HE	ARG	A	174	9.126	-9.536	-12.654	1.00	0.00	H
ATOM	1689	CZ	ARG	A	174	10.584	-8.932	-14.037	1.00	0.00	C
ATOM	1690	NH1	ARG	A	174	11.063	-9.159	-15.281	1.00	0.00	N
ATOM	1691	HH11	ARG	A	174	10.595	-9.766	-15.942	1.00	0.00	H
ATOM	1692	HH12	ARG	A	174	11.893	-8.723	-15.638	1.00	0.00	H
ATOM	1693	NH2	ARG	A	174	11.117	-7.981	-13.239	1.00	0.00	N
ATOM	1694	HH21	ARG	A	174	10.630	-7.699	-12.398	1.00	0.00	H
ATOM	1695	HH22	ARG	A	174	11.972	-7.499	-13.441	1.00	0.00	H
ATOM	1696	C	ARG	A	174	4.457	-12.657	-14.733	1.00	0.00	C
ATOM	1697	O	ARG	A	174	4.042	-13.572	-15.449	1.00	0.00	O
ATOM	1698	N	THR	A	175	3.875	-11.441	-14.653	1.00	0.00	N
ATOM	1699	H	THR	A	175	4.223	-10.762	-14.007	1.00	0.00	H
ATOM	1700	CA	THR	A	175	2.710	-10.985	-15.365	1.00	0.00	C
ATOM	1701	CB	THR	A	175	2.393	-9.648	-14.738	1.00	0.00	C
ATOM	1702	OG1	THR	A	175	2.490	-9.831	-13.323	1.00	0.00	O
ATOM	1703	HG1	THR	A	175	1.721	-10.348	-13.081	1.00	0.00	H
ATOM	1704	CG2	THR	A	175	1.033	-9.086	-15.155	1.00	0.00	C
ATOM	1705	C	THR	A	175	3.054	-10.948	-16.838	1.00	0.00	C
ATOM	1706	O	THR	A	175	4.162	-10.570	-17.222	1.00	0.00	O
ATOM	1707	N	LEU	A	176	2.085	-11.344	-17.694	1.00	0.00	N
ATOM	1708	H	LEU	A	176	1.222	-11.657	-17.301	1.00	0.00	H
ATOM	1709	CA	LEU	A	176	2.251	-11.390	-19.128	1.00	0.00	C
ATOM	1710	CB	LEU	A	176	1.040	-12.026	-19.810	1.00	0.00	C
ATOM	1711	CG	LEU	A	176	0.848	-13.502	-19.458	1.00	0.00	C

```
ATOM   1712  CD1 LEU A 176      -0.386 -14.087 -20.148  1.00  0.00           C
ATOM   1713  CD2 LEU A 176       2.107 -14.323 -19.739  1.00  0.00           C
ATOM   1714  C   LEU A 176       2.496 -10.025 -19.717  1.00  0.00           C
ATOM   1715  O   LEU A 176       3.299  -9.887 -20.641  1.00  0.00           O
ATOM   1716  N   SER A 177       1.831  -8.991 -19.162  1.00  0.00           N
ATOM   1717  H   SER A 177       1.161  -9.158 -18.443  1.00  0.00           H
ATOM   1718  CA  SER A 177       1.943  -7.631 -19.615  1.00  0.00           C
ATOM   1719  CB  SER A 177       0.483  -7.209 -19.629  1.00  0.00           C
ATOM   1720  OG  SER A 177      -0.273  -8.408 -19.362  1.00  0.00           O
ATOM   1721  HG  SER A 177      -1.185  -8.138 -19.271  1.00  0.00           H
ATOM   1722  C   SER A 177       2.850  -6.796 -18.734  1.00  0.00           C
ATOM   1723  O   SER A 177       2.809  -5.566 -18.803  1.00  0.00           O
ATOM   1724  N   TYR A 178       3.742  -7.456 -17.946  1.00  0.00           N
ATOM   1725  H   TYR A 178       3.778  -8.454 -18.027  1.00  0.00           H
ATOM   1726  CA  TYR A 178       4.659  -6.854 -16.997  1.00  0.00           C
ATOM   1727  CB  TYR A 178       5.552  -7.944 -16.406  1.00  0.00           C
ATOM   1728  CG  TYR A 178       6.360  -7.476 -15.219  1.00  0.00           C
ATOM   1729  CD1 TYR A 178       5.913  -7.738 -13.928  1.00  0.00           C
ATOM   1730  CE1 TYR A 178       6.733  -7.460 -12.840  1.00  0.00           C
ATOM   1731  CD2 TYR A 178       7.572  -6.826 -15.412  1.00  0.00           C
ATOM   1732  CE2 TYR A 178       8.384  -6.535 -14.326  1.00  0.00           C
ATOM   1733  CZ  TYR A 178       7.995  -6.917 -13.048  1.00  0.00           C
ATOM   1734  OH  TYR A 178       8.893  -6.800 -12.001  1.00  0.00           O
ATOM   1735  HH  TYR A 178       8.398  -6.634 -11.202  1.00  0.00           H
ATOM   1736  C   TYR A 178       5.500  -5.741 -17.558  1.00  0.00           C
ATOM   1737  O   TYR A 178       5.675  -4.735 -16.870  1.00  0.00           O
ATOM   1738  N   TYR A 179       6.007  -5.873 -18.804  1.00  0.00           N
ATOM   1739  H   TYR A 179       5.765  -6.686 -19.332  1.00  0.00           H
ATOM   1740  CA  TYR A 179       6.846  -4.856 -19.394  1.00  0.00           C
ATOM   1741  CB  TYR A 179       7.654  -5.494 -20.521  1.00  0.00           C
ATOM   1742  CG  TYR A 179       8.369  -6.654 -19.860  1.00  0.00           C
ATOM   1743  CD1 TYR A 179       8.099  -7.969 -20.228  1.00  0.00           C
ATOM   1744  CE1 TYR A 179       8.666  -9.021 -19.514  1.00  0.00           C
ATOM   1745  CD2 TYR A 179       9.268  -6.401 -18.829  1.00  0.00           C
ATOM   1746  CE2 TYR A 179       9.833  -7.452 -18.116  1.00  0.00           C
ATOM   1747  CZ  TYR A 179       9.512  -8.765 -18.438  1.00  0.00           C
ATOM   1748  OH  TYR A 179      10.027  -9.809 -17.686  1.00  0.00           O
ATOM   1749  HH  TYR A 179       9.855 -10.616 -18.168  1.00  0.00           H
ATOM   1750  C   TYR A 179       6.106  -3.584 -19.691  1.00  0.00           C
ATOM   1751  O   TYR A 179       6.641  -2.495 -19.461  1.00  0.00           O
ATOM   1752  N   LYS A 180       4.836  -3.701 -20.157  1.00  0.00           N
ATOM   1753  H   LYS A 180       4.404  -4.602 -20.171  1.00  0.00           H
ATOM   1754  CA  LYS A 180       4.017  -2.550 -20.459  1.00  0.00           C
ATOM   1755  CB  LYS A 180       2.738  -2.921 -21.203  1.00  0.00           C
ATOM   1756  CG  LYS A 180       2.942  -3.121 -22.703  1.00  0.00           C
ATOM   1757  CD  LYS A 180       1.598  -3.148 -23.428  1.00  0.00           C
ATOM   1758  CE  LYS A 180       1.732  -3.180 -24.951  1.00  0.00           C
ATOM   1759  NZ  LYS A 180       0.401  -3.039 -25.541  1.00  0.00           N
ATOM   1760  HZ1 LYS A 180      -0.281  -3.748 -25.196  1.00  0.00           H
ATOM   1761  HZ2 LYS A 180      -0.090  -2.176 -25.213  1.00  0.00           H
ATOM   1762  HZ3 LYS A 180       0.361  -3.001 -26.573  1.00  0.00           H
ATOM   1763  C   LYS A 180       3.654  -1.870 -19.173  1.00  0.00           C
ATOM   1764  O   LYS A 180       3.672  -0.641 -19.093  1.00  0.00           O
ATOM   1765  N   LEU A 181       3.378  -2.681 -18.122  1.00  0.00           N
ATOM   1766  H   LEU A 181       3.383  -3.671 -18.273  1.00  0.00           H
ATOM   1767  CA  LEU A 181       3.012  -2.200 -16.818  1.00  0.00           C
ATOM   1768  CB  LEU A 181       2.567  -3.341 -15.907  1.00  0.00           C
ATOM   1769  CG  LEU A 181       1.318  -4.070 -16.393  1.00  0.00           C
ATOM   1770  CD1 LEU A 181       0.939  -5.194 -15.432  1.00  0.00           C
```

ATOM	1771	CD2	LEU	A	181	0.153	-3.119	-16.660	1.00	0.00	C
ATOM	1772	C	LEU	A	181	4.183	-1.486	-16.193	1.00	0.00	C
ATOM	1773	O	LEU	A	181	3.978	-0.483	-15.513	1.00	0.00	O
ATOM	1774	N	GLY	A	182	5.433	-1.956	-16.446	1.00	0.00	N
ATOM	1775	H	GLY	A	182	5.517	-2.792	-16.988	1.00	0.00	H
ATOM	1776	CA	GLY	A	182	6.640	-1.366	-15.917	1.00	0.00	C
ATOM	1777	C	GLY	A	182	6.884	-0.016	-16.509	1.00	0.00	C
ATOM	1778	O	GLY	A	182	7.249	0.910	-15.784	1.00	0.00	O
ATOM	1779	N	ALA	A	183	6.642	0.126	-17.835	1.00	0.00	N
ATOM	1780	H	ALA	A	183	6.337	-0.674	-18.358	1.00	0.00	H
ATOM	1781	CA	ALA	A	183	6.822	1.374	-18.533	1.00	0.00	C
ATOM	1782	CB	ALA	A	183	6.642	1.180	-20.039	1.00	0.00	C
ATOM	1783	C	ALA	A	183	5.801	2.363	-18.036	1.00	0.00	C
ATOM	1784	O	ALA	A	183	6.124	3.534	-17.830	1.00	0.00	O
ATOM	1785	N	SER	A	184	4.563	1.873	-17.769	1.00	0.00	N
ATOM	1786	H	SER	A	184	4.353	0.924	-18.005	1.00	0.00	H
ATOM	1787	CA	SER	A	184	3.459	2.656	-17.277	1.00	0.00	C
ATOM	1788	CB	SER	A	184	2.210	1.802	-17.391	1.00	0.00	C
ATOM	1789	OG	SER	A	184	1.991	1.572	-18.781	1.00	0.00	O
ATOM	1790	HG	SER	A	184	2.394	0.743	-19.022	1.00	0.00	H
ATOM	1791	C	SER	A	184	3.749	3.176	-15.892	1.00	0.00	C
ATOM	1792	O	SER	A	184	3.472	4.339	-15.614	1.00	0.00	O
ATOM	1793	N	GLN	A	185	4.363	2.337	-15.021	1.00	0.00	N
ATOM	1794	H	GLN	A	185	4.541	1.404	-15.339	1.00	0.00	H
ATOM	1795	CA	GLN	A	185	4.747	2.642	-13.657	1.00	0.00	C
ATOM	1796	CB	GLN	A	185	5.350	1.366	-13.060	1.00	0.00	C
ATOM	1797	CG	GLN	A	185	6.088	1.494	-11.725	1.00	0.00	C
ATOM	1798	CD	GLN	A	185	5.114	1.677	-10.584	1.00	0.00	C
ATOM	1799	OE1	GLN	A	185	4.553	0.725	-10.049	1.00	0.00	O
ATOM	1800	NE2	GLN	A	185	4.982	2.957	-10.204	1.00	0.00	N
ATOM	1801	HE21	GLN	A	185	4.434	3.202	-9.404	1.00	0.00	H
ATOM	1802	HE22	GLN	A	185	5.416	3.703	-10.719	1.00	0.00	H
ATOM	1803	C	GLN	A	185	5.737	3.776	-13.628	1.00	0.00	C
ATOM	1804	O	GLN	A	185	5.568	4.728	-12.861	1.00	0.00	O
ATOM	1805	N	ARG	A	186	6.749	3.710	-14.526	1.00	0.00	N
ATOM	1806	H	ARG	A	186	6.767	2.932	-15.158	1.00	0.00	H
ATOM	1807	CA	ARG	A	186	7.804	4.687	-14.594	1.00	0.00	C
ATOM	1808	CB	ARG	A	186	8.897	4.244	-15.560	1.00	0.00	C
ATOM	1809	CG	ARG	A	186	9.599	2.946	-15.180	1.00	0.00	C
ATOM	1810	CD	ARG	A	186	10.552	2.521	-16.295	1.00	0.00	C
ATOM	1811	NE	ARG	A	186	11.195	1.246	-15.990	1.00	0.00	N
ATOM	1812	HE	ARG	A	186	10.644	0.621	-15.426	1.00	0.00	H
ATOM	1813	CZ	ARG	A	186	12.422	0.992	-16.534	1.00	0.00	C
ATOM	1814	NH1	ARG	A	186	13.018	1.941	-17.293	1.00	0.00	N
ATOM	1815	HH11	ARG	A	186	12.582	2.831	-17.457	1.00	0.00	H
ATOM	1816	HH12	ARG	A	186	13.915	1.811	-17.726	1.00	0.00	H
ATOM	1817	NH2	ARG	A	186	13.019	-0.201	-16.306	1.00	0.00	N
ATOM	1818	HH21	ARG	A	186	12.578	-0.903	-15.741	1.00	0.00	H
ATOM	1819	HH22	ARG	A	186	13.919	-0.439	-16.683	1.00	0.00	H
ATOM	1820	C	ARG	A	186	7.328	6.032	-15.042	1.00	0.00	C
ATOM	1821	O	ARG	A	186	7.678	7.038	-14.421	1.00	0.00	O
ATOM	1822	N	VAL	A	187	6.477	6.071	-16.095	1.00	0.00	N
ATOM	1823	H	VAL	A	187	6.198	5.211	-16.527	1.00	0.00	H
ATOM	1824	CA	VAL	A	187	5.976	7.314	-16.624	1.00	0.00	C
ATOM	1825	CB	VAL	A	187	5.320	7.105	-17.990	1.00	0.00	C
ATOM	1826	CG1	VAL	A	187	4.686	8.397	-18.512	1.00	0.00	C
ATOM	1827	CG2	VAL	A	187	6.331	6.536	-18.985	1.00	0.00	C
ATOM	1828	C	VAL	A	187	5.014	7.933	-15.640	1.00	0.00	C
ATOM	1829	O	VAL	A	187	5.118	9.130	-15.381	1.00	0.00	O

```
ATOM   1830  N   ALA A 188       4.129   7.119 -15.017  1.00  0.00           N
ATOM   1831  H   ALA A 188       4.146   6.141 -15.229  1.00  0.00           H
ATOM   1832  CA  ALA A 188       3.131   7.584 -14.085  1.00  0.00           C
ATOM   1833  CB  ALA A 188       2.130   6.487 -13.736  1.00  0.00           C
ATOM   1834  C   ALA A 188       3.691   8.130 -12.815  1.00  0.00           C
ATOM   1835  O   ALA A 188       3.105   9.066 -12.274  1.00  0.00           O
ATOM   1836  N   GLY A 189       4.836   7.591 -12.324  1.00  0.00           N
ATOM   1837  H   GLY A 189       5.294   6.850 -12.818  1.00  0.00           H
ATOM   1838  CA  GLY A 189       5.430   8.074 -11.101  1.00  0.00           C
ATOM   1839  C   GLY A 189       5.872   9.504 -11.232  1.00  0.00           C
ATOM   1840  O   GLY A 189       5.663  10.297 -10.313  1.00  0.00           O
ATOM   1841  N   ASP A 190       6.474   9.853 -12.392  1.00  0.00           N
ATOM   1842  H   ASP A 190       6.694   9.157 -13.075  1.00  0.00           H
ATOM   1843  CA  ASP A 190       6.946  11.190 -12.642  1.00  0.00           C
ATOM   1844  CB  ASP A 190       8.167  11.167 -13.561  1.00  0.00           C
ATOM   1845  CG  ASP A 190       9.328  10.518 -12.828  1.00  0.00           C
ATOM   1846  OD1 ASP A 190       9.240  10.346 -11.611  1.00  0.00           O
ATOM   1847  OD2 ASP A 190      10.318  10.186 -13.475  1.00  0.00           O
ATOM   1848  C   ASP A 190       5.889  12.149 -13.119  1.00  0.00           C
ATOM   1849  O   ASP A 190       5.879  13.305 -12.692  1.00  0.00           O
ATOM   1850  N   SER A 191       4.962  11.682 -13.990  1.00  0.00           N
ATOM   1851  H   SER A 191       4.978  10.712 -14.230  1.00  0.00           H
ATOM   1852  CA  SER A 191       3.926  12.515 -14.554  1.00  0.00           C
ATOM   1853  CB  SER A 191       3.636  12.222 -16.032  1.00  0.00           C
ATOM   1854  OG  SER A 191       3.433  10.831 -16.269  1.00  0.00           O
ATOM   1855  HG  SER A 191       4.299  10.447 -16.229  1.00  0.00           H
ATOM   1856  C   SER A 191       2.694  12.660 -13.704  1.00  0.00           C
ATOM   1857  O   SER A 191       1.904  13.583 -13.922  1.00  0.00           O
ATOM   1858  N   GLY A 192       2.523  11.779 -12.696  1.00  0.00           N
ATOM   1859  H   GLY A 192       3.239  11.111 -12.491  1.00  0.00           H
ATOM   1860  CA  GLY A 192       1.385  11.800 -11.820  1.00  0.00           C
ATOM   1861  C   GLY A 192       1.803  12.510 -10.584  1.00  0.00           C
ATOM   1862  O   GLY A 192       2.357  13.612 -10.645  1.00  0.00           O
ATOM   1863  N   PHE A 193       1.513  11.891  -9.417  1.00  0.00           N
ATOM   1864  H   PHE A 193       1.267  10.924  -9.443  1.00  0.00           H
ATOM   1865  CA  PHE A 193       1.880  12.497  -8.173  1.00  0.00           C
ATOM   1866  CB  PHE A 193       1.049  11.953  -7.009  1.00  0.00           C
ATOM   1867  CG  PHE A 193      -0.411  12.307  -7.178  1.00  0.00           C
ATOM   1868  CD1 PHE A 193      -1.264  11.426  -7.879  1.00  0.00           C
ATOM   1869  CD2 PHE A 193      -0.899  13.513  -6.630  1.00  0.00           C
ATOM   1870  CE1 PHE A 193      -2.623  11.759  -8.040  1.00  0.00           C
ATOM   1871  CE2 PHE A 193      -2.259  13.846  -6.788  1.00  0.00           C
ATOM   1872  CZ  PHE A 193      -3.107  12.966  -7.493  1.00  0.00           C
ATOM   1873  C   PHE A 193       3.340  12.177  -8.022  1.00  0.00           C
ATOM   1874  O   PHE A 193       3.739  11.034  -7.776  1.00  0.00           O
ATOM   1875  N   ALA A 194       4.142  13.262  -8.115  1.00  0.00           N
ATOM   1876  H   ALA A 194       3.683  14.077  -8.468  1.00  0.00           H
ATOM   1877  CA  ALA A 194       5.585  13.292  -8.052  1.00  0.00           C
ATOM   1878  CB  ALA A 194       6.107  14.705  -8.313  1.00  0.00           C
ATOM   1879  C   ALA A 194       6.123  12.814  -6.741  1.00  0.00           C
ATOM   1880  O   ALA A 194       7.292  12.431  -6.650  1.00  0.00           O
ATOM   1881  N   ALA A 195       5.254  12.811  -5.702  1.00  0.00           N
ATOM   1882  H   ALA A 195       4.314  13.072  -5.899  1.00  0.00           H
ATOM   1883  CA  ALA A 195       5.562  12.390  -4.369  1.00  0.00           C
ATOM   1884  CB  ALA A 195       4.314  12.479  -3.493  1.00  0.00           C
ATOM   1885  C   ALA A 195       6.083  10.980  -4.375  1.00  0.00           C
ATOM   1886  O   ALA A 195       7.017  10.718  -3.628  1.00  0.00           O
ATOM   1887  N   TYR A 196       5.566  10.076  -5.256  1.00  0.00           N
ATOM   1888  H   TYR A 196       4.875  10.381  -5.913  1.00  0.00           H
```

```
ATOM   1889  CA  TYR A 196       6.007   8.693  -5.338  1.00  0.00           C
ATOM   1890  CB  TYR A 196       5.248   7.964  -6.444  1.00  0.00           C
ATOM   1891  CG  TYR A 196       5.640   6.508  -6.528  1.00  0.00           C
ATOM   1892  CD1 TYR A 196       5.159   5.602  -5.591  1.00  0.00           C
ATOM   1893  CE1 TYR A 196       5.486   4.255  -5.699  1.00  0.00           C
ATOM   1894  CD2 TYR A 196       6.467   6.069  -7.557  1.00  0.00           C
ATOM   1895  CE2 TYR A 196       6.799   4.724  -7.659  1.00  0.00           C
ATOM   1896  CZ  TYR A 196       6.299   3.813  -6.736  1.00  0.00           C
ATOM   1897  OH  TYR A 196       6.608   2.470  -6.855  1.00  0.00           O
ATOM   1898  HH  TYR A 196       5.783   2.010  -7.039  1.00  0.00           H
ATOM   1899  C   TYR A 196       7.467   8.636  -5.700  1.00  0.00           C
ATOM   1900  O   TYR A 196       8.214   7.864  -5.094  1.00  0.00           O
ATOM   1901  N   SER A 197       7.890   9.482  -6.669  1.00  0.00           N
ATOM   1902  H   SER A 197       7.278  10.083  -7.187  1.00  0.00           H
ATOM   1903  CA  SER A 197       9.249   9.527  -7.136  1.00  0.00           C
ATOM   1904  CB  SER A 197       9.221  10.382  -8.378  1.00  0.00           C
ATOM   1905  OG  SER A 197       7.986  10.050  -9.010  1.00  0.00           O
ATOM   1906  HG  SER A 197       8.136  10.240  -9.942  1.00  0.00           H
ATOM   1907  C   SER A 197      10.154   9.995  -6.044  1.00  0.00           C
ATOM   1908  O   SER A 197      11.216   9.410  -5.860  1.00  0.00           O
ATOM   1909  N   ARG A 198       9.710  11.002  -5.255  1.00  0.00           N
ATOM   1910  H   ARG A 198       8.817  11.396  -5.481  1.00  0.00           H
ATOM   1911  CA  ARG A 198      10.471  11.571  -4.165  1.00  0.00           C
ATOM   1912  CB  ARG A 198       9.716  12.687  -3.462  1.00  0.00           C
ATOM   1913  CG  ARG A 198       9.376  13.927  -4.267  1.00  0.00           C
ATOM   1914  CD  ARG A 198       8.562  14.831  -3.349  1.00  0.00           C
ATOM   1915  NE  ARG A 198       8.311  16.127  -3.960  1.00  0.00           N
ATOM   1916  HE  ARG A 198       9.045  16.438  -4.579  1.00  0.00           H
ATOM   1917  CZ  ARG A 198       7.244  16.849  -3.513  1.00  0.00           C
ATOM   1918  NH1 ARG A 198       6.406  16.315  -2.596  1.00  0.00           N
ATOM   1919 HH11 ARG A 198       6.542  15.399  -2.179  1.00  0.00           H
ATOM   1920 HH12 ARG A 198       5.601  16.784  -2.227  1.00  0.00           H
ATOM   1921  NH2 ARG A 198       7.061  18.102  -3.985  1.00  0.00           N
ATOM   1922 HH21 ARG A 198       7.704  18.489  -4.655  1.00  0.00           H
ATOM   1923 HH22 ARG A 198       6.312  18.696  -3.683  1.00  0.00           H
ATOM   1924  C   ARG A 198      10.670  10.538  -3.086  1.00  0.00           C
ATOM   1925  O   ARG A 198      11.779  10.387  -2.577  1.00  0.00           O
ATOM   1926  N   TYR A 199       9.606   9.747  -2.800  1.00  0.00           N
ATOM   1927  H   TYR A 199       8.759   9.946  -3.294  1.00  0.00           H
ATOM   1928  CA  TYR A 199       9.564   8.725  -1.783  1.00  0.00           C
ATOM   1929  CB  TYR A 199       8.158   8.119  -1.691  1.00  0.00           C
ATOM   1930  CG  TYR A 199       7.154   9.141  -1.201  1.00  0.00           C
ATOM   1931  CD1 TYR A 199       7.590  10.331  -0.632  1.00  0.00           C
ATOM   1932  CE1 TYR A 199       6.672  11.288  -0.224  1.00  0.00           C
ATOM   1933  CD2 TYR A 199       5.789   8.898  -1.320  1.00  0.00           C
ATOM   1934  CE2 TYR A 199       4.869   9.851  -0.890  1.00  0.00           C
ATOM   1935  CZ  TYR A 199       5.312  11.056  -0.353  1.00  0.00           C
ATOM   1936  OH  TYR A 199       4.414  12.025   0.046  1.00  0.00           O
ATOM   1937  HH  TYR A 199       4.902  12.840   0.213  1.00  0.00           H
ATOM   1938  C   TYR A 199      10.585   7.650  -2.011  1.00  0.00           C
ATOM   1939  O   TYR A 199      11.129   7.114  -1.044  1.00  0.00           O
ATOM   1940  N   ARG A 200      10.819   7.291  -3.294  1.00  0.00           N
ATOM   1941  H   ARG A 200      10.355   7.812  -4.014  1.00  0.00           H
ATOM   1942  CA  ARG A 200      11.779   6.288  -3.656  1.00  0.00           C
ATOM   1943  CB  ARG A 200      11.281   5.592  -4.918  1.00  0.00           C
ATOM   1944  CG  ARG A 200      11.517   4.092  -4.832  1.00  0.00           C
ATOM   1945  CD  ARG A 200      10.931   3.312  -6.004  1.00  0.00           C
ATOM   1946  NE  ARG A 200      11.230   1.892  -5.840  1.00  0.00           N
ATOM   1947  HE  ARG A 200      12.193   1.702  -5.595  1.00  0.00           H
```

ATOM	1948	CZ	ARG	A	200	10.226	0.979	-6.015	1.00	0.00	C
ATOM	1949	NH1	ARG	A	200	8.977	1.405	-6.314	1.00	0.00	N
ATOM	1950	HH11	ARG	A	200	8.728	2.376	-6.414	1.00	0.00	H
ATOM	1951	HH12	ARG	A	200	8.193	0.795	-6.461	1.00	0.00	H
ATOM	1952	NH2	ARG	A	200	10.498	-0.340	-5.886	1.00	0.00	N
ATOM	1953	HH21	ARG	A	200	11.425	-0.657	-5.664	1.00	0.00	H
ATOM	1954	HH22	ARG	A	200	9.799	-1.052	-6.004	1.00	0.00	H
ATOM	1955	C	ARG	A	200	13.185	6.833	-3.806	1.00	0.00	C
ATOM	1956	O	ARG	A	200	14.131	6.272	-3.249	1.00	0.00	O
ATOM	1957	N	ILE	A	201	13.330	7.991	-4.493	1.00	0.00	N
ATOM	1958	H	ILE	A	201	12.505	8.460	-4.814	1.00	0.00	H
ATOM	1959	CA	ILE	A	201	14.599	8.628	-4.795	1.00	0.00	C
ATOM	1960	CB	ILE	A	201	14.408	9.703	-5.876	1.00	0.00	C
ATOM	1961	CG2	ILE	A	201	15.618	10.624	-6.062	1.00	0.00	C
ATOM	1962	CG1	ILE	A	201	14.040	9.022	-7.196	1.00	0.00	C
ATOM	1963	CD1	ILE	A	201	13.709	10.032	-8.295	1.00	0.00	C
ATOM	1964	C	ILE	A	201	15.308	9.162	-3.575	1.00	0.00	C
ATOM	1965	O	ILE	A	201	16.541	9.191	-3.567	1.00	0.00	O
ATOM	1966	N	GLY	A	202	14.567	9.515	-2.499	1.00	0.00	N
ATOM	1967	H	GLY	A	202	13.569	9.571	-2.582	1.00	0.00	H
ATOM	1968	CA	GLY	A	202	15.146	10.043	-1.286	1.00	0.00	C
ATOM	1969	C	GLY	A	202	16.105	9.091	-0.633	1.00	0.00	C
ATOM	1970	O	GLY	A	202	17.085	9.522	-0.023	1.00	0.00	O
ATOM	1971	N	ASN	A	203	15.827	7.773	-0.753	1.00	0.00	N
ATOM	1972	H	ASN	A	203	15.074	7.513	-1.357	1.00	0.00	H
ATOM	1973	CA	ASN	A	203	16.669	6.753	-0.196	1.00	0.00	C
ATOM	1974	CB	ASN	A	203	15.908	5.432	-0.075	1.00	0.00	C
ATOM	1975	CG	ASN	A	203	15.369	5.248	1.327	1.00	0.00	C
ATOM	1976	OD1	ASN	A	203	16.026	4.704	2.205	1.00	0.00	O
ATOM	1977	ND2	ASN	A	203	14.113	5.707	1.487	1.00	0.00	N
ATOM	1978	HD21	ASN	A	203	13.656	5.627	2.373	1.00	0.00	H
ATOM	1979	HD22	ASN	A	203	13.601	6.130	0.737	1.00	0.00	H
ATOM	1980	C	ASN	A	203	17.868	6.527	-1.069	1.00	0.00	C
ATOM	1981	O	ASN	A	203	18.971	6.341	-0.552	1.00	0.00	O
ATOM	1982	N	TYR	A	204	17.668	6.548	-2.413	1.00	0.00	N
ATOM	1983	H	TYR	A	204	16.784	6.859	-2.757	1.00	0.00	H
ATOM	1984	CA	TYR	A	204	18.732	6.285	-3.345	1.00	0.00	C
ATOM	1985	CB	TYR	A	204	18.179	5.804	-4.694	1.00	0.00	C
ATOM	1986	CG	TYR	A	204	17.070	4.776	-4.594	1.00	0.00	C
ATOM	1987	CD1	TYR	A	204	16.021	4.845	-5.504	1.00	0.00	C
ATOM	1988	CE1	TYR	A	204	15.008	3.893	-5.485	1.00	0.00	C
ATOM	1989	CD2	TYR	A	204	17.090	3.756	-3.648	1.00	0.00	C
ATOM	1990	CE2	TYR	A	204	16.068	2.810	-3.621	1.00	0.00	C
ATOM	1991	CZ	TYR	A	204	15.028	2.871	-4.544	1.00	0.00	C
ATOM	1992	OH	TYR	A	204	14.021	1.917	-4.524	1.00	0.00	O
ATOM	1993	HH	TYR	A	204	14.184	1.358	-3.772	1.00	0.00	H
ATOM	1994	C	TYR	A	204	19.698	7.389	-3.658	1.00	0.00	C
ATOM	1995	O	TYR	A	204	20.890	7.216	-3.391	1.00	0.00	O
ATOM	1996	N	LYS	A	205	19.221	8.545	-4.193	1.00	0.00	N
ATOM	1997	H	LYS	A	205	18.236	8.709	-4.218	1.00	0.00	H
ATOM	1998	CA	LYS	A	205	20.142	9.584	-4.563	1.00	0.00	C
ATOM	1999	CB	LYS	A	205	20.068	9.848	-6.067	1.00	0.00	C
ATOM	2000	CG	LYS	A	205	21.187	10.781	-6.536	1.00	0.00	C
ATOM	2001	CD	LYS	A	205	21.123	11.091	-8.031	1.00	0.00	C
ATOM	2002	CE	LYS	A	205	22.250	12.029	-8.468	1.00	0.00	C
ATOM	2003	NZ	LYS	A	205	22.149	12.282	-9.912	1.00	0.00	N
ATOM	2004	HZ1	LYS	A	205	22.914	12.924	-10.204	1.00	0.00	H
ATOM	2005	HZ2	LYS	A	205	22.236	11.384	-10.429	1.00	0.00	H
ATOM	2006	HZ3	LYS	A	205	21.229	12.719	-10.125	1.00	0.00	H

```
ATOM   2007  C   LYS A 205      20.027  10.866  -3.801  1.00  0.00           C
ATOM   2008  O   LYS A 205      20.951  11.202  -3.057  1.00  0.00           O
ATOM   2009  N   LEU A 206      18.892  11.592  -3.953  1.00  0.00           N
ATOM   2010  H   LEU A 206      18.071  11.165  -4.331  1.00  0.00           H
ATOM   2011  CA  LEU A 206      18.752  12.861  -3.294  1.00  0.00           C
ATOM   2012  CB  LEU A 206      18.039  13.847  -4.213  1.00  0.00           C
ATOM   2013  CG  LEU A 206      18.776  14.064  -5.534  1.00  0.00           C
ATOM   2014  CD1 LEU A 206      17.949  14.897  -6.512  1.00  0.00           C
ATOM   2015  CD2 LEU A 206      20.173  14.649  -5.316  1.00  0.00           C
ATOM   2016  C   LEU A 206      18.068  12.786  -1.979  1.00  0.00           C
ATOM   2017  O   LEU A 206      16.858  12.575  -1.891  1.00  0.00           O
ATOM   2018  N   ASN A 207      18.877  12.999  -0.925  1.00  0.00           N
ATOM   2019  H   ASN A 207      19.859  13.064  -1.107  1.00  0.00           H
ATOM   2020  CA  ASN A 207      18.435  12.992   0.442  1.00  0.00           C
ATOM   2021  CB  ASN A 207      19.568  12.794   1.450  1.00  0.00           C
ATOM   2022  CG  ASN A 207      20.090  11.379   1.407  1.00  0.00           C
ATOM   2023  OD1 ASN A 207      21.106  11.105   0.778  1.00  0.00           O
ATOM   2024  ND2 ASN A 207      19.344  10.502   2.097  1.00  0.00           N
ATOM   2025 HD21 ASN A 207      19.511   9.517   2.049  1.00  0.00           H
ATOM   2026 HD22 ASN A 207      18.578  10.817   2.666  1.00  0.00           H
ATOM   2027  C   ASN A 207      17.757  14.287   0.800  1.00  0.00           C
ATOM   2028  O   ASN A 207      16.835  14.304   1.613  1.00  0.00           O
ATOM   2029  N   THR A 208      18.214  15.404   0.192  1.00  0.00           N
ATOM   2030  H   THR A 208      18.999  15.333  -0.420  1.00  0.00           H
ATOM   2031  CA  THR A 208      17.719  16.740   0.424  1.00  0.00           C
ATOM   2032  CB  THR A 208      18.964  17.577   0.254  1.00  0.00           C
ATOM   2033  OG1 THR A 208      20.075  16.669   0.325  1.00  0.00           O
ATOM   2034  HG1 THR A 208      20.852  17.204   0.436  1.00  0.00           H
ATOM   2035  CG2 THR A 208      19.077  18.690   1.298  1.00  0.00           C
ATOM   2036  C   THR A 208      16.550  17.179  -0.425  1.00  0.00           C
ATOM   2037  O   THR A 208      15.987  18.251  -0.179  1.00  0.00           O
ATOM   2038  N   ASP A 209      16.144  16.355  -1.423  1.00  0.00           N
ATOM   2039  H   ASP A 209      16.549  15.446  -1.505  1.00  0.00           H
ATOM   2040  CA  ASP A 209      15.061  16.667  -2.331  1.00  0.00           C
ATOM   2041  CB  ASP A 209      14.837  15.578  -3.379  1.00  0.00           C
ATOM   2042  CG  ASP A 209      13.352  15.527  -3.710  1.00  0.00           C
ATOM   2043  OD1 ASP A 209      12.863  16.397  -4.429  1.00  0.00           O
ATOM   2044  OD2 ASP A 209      12.676  14.627  -3.220  1.00  0.00           O
ATOM   2045  C   ASP A 209      13.753  16.845  -1.603  1.00  0.00           C
ATOM   2046  O   ASP A 209      12.998  17.765  -1.918  1.00  0.00           O
ATOM   2047  N   HIS A 210      13.508  15.999  -0.577  1.00  0.00           N
ATOM   2048  H   HIS A 210      14.145  15.233  -0.517  1.00  0.00           H
ATOM   2049  CA  HIS A 210      12.320  15.975   0.242  1.00  0.00           C
ATOM   2050  CB  HIS A 210      12.414  14.947   1.377  1.00  0.00           C
ATOM   2051  CG  HIS A 210      12.421  13.490   0.980  1.00  0.00           C
ATOM   2052  ND1 HIS A 210      12.340  12.507   1.901  1.00  0.00           N
ATOM   2053  HD1 HIS A 210      12.233  12.625   2.869  1.00  0.00           H
ATOM   2054  CD2 HIS A 210      12.501  12.914  -0.290  1.00  0.00           C
ATOM   2055  NE2 HIS A 210      12.468  11.569  -0.110  1.00  0.00           N
ATOM   2056  CE1 HIS A 210      12.368  11.315   1.227  1.00  0.00           C
ATOM   2057  C   HIS A 210      12.164  17.267   0.988  1.00  0.00           C
ATOM   2058  O   HIS A 210      11.054  17.791   1.070  1.00  0.00           O
ATOM   2059  N   SER A 211      13.292  17.800   1.517  1.00  0.00           N
ATOM   2060  H   SER A 211      14.163  17.339   1.356  1.00  0.00           H
ATOM   2061  CA  SER A 211      13.353  19.019   2.279  1.00  0.00           C
ATOM   2062  CB  SER A 211      14.730  18.959   2.904  1.00  0.00           C
ATOM   2063  OG  SER A 211      15.078  17.564   2.932  1.00  0.00           O
ATOM   2064  HG  SER A 211      15.780  17.469   3.565  1.00  0.00           H
ATOM   2065  C   SER A 211      13.068  20.219   1.420  1.00  0.00           C
```

ATOM	2066	O	SER	A	211	12.359	21.125	1.862	1.00	0.00	O
ATOM	2067	N	SER	A	212	13.593	20.229	0.167	1.00	0.00	N
ATOM	2068	H	SER	A	212	14.148	19.457	-0.140	1.00	0.00	H
ATOM	2069	CA	SER	A	212	13.411	21.310	-0.774	1.00	0.00	C
ATOM	2070	CB	SER	A	212	14.438	21.074	-1.869	1.00	0.00	C
ATOM	2071	OG	SER	A	212	15.673	20.749	-1.214	1.00	0.00	O
ATOM	2072	HG	SER	A	212	15.710	19.803	-1.130	1.00	0.00	H
ATOM	2073	C	SER	A	212	11.969	21.364	-1.218	1.00	0.00	C
ATOM	2074	O	SER	A	212	11.365	22.436	-1.265	1.00	0.00	O
ATOM	2075	N	SER	A	213	11.387	20.176	-1.506	1.00	0.00	N
ATOM	2076	H	SER	A	213	11.945	19.345	-1.526	1.00	0.00	H
ATOM	2077	CA	SER	A	213	10.023	19.979	-1.929	1.00	0.00	C
ATOM	2078	CB	SER	A	213	10.014	18.557	-2.461	1.00	0.00	C
ATOM	2079	OG	SER	A	213	10.860	18.492	-3.615	1.00	0.00	O
ATOM	2080	HG	SER	A	213	11.569	17.855	-3.450	1.00	0.00	H
ATOM	2081	C	SER	A	213	9.068	20.226	-0.790	1.00	0.00	C
ATOM	2082	O	SER	A	213	7.915	20.595	-1.022	1.00	0.00	O
ATOM	2083	N	SER	A	214	9.560	20.050	0.465	1.00	0.00	N
ATOM	2084	H	SER	A	214	10.490	19.698	0.541	1.00	0.00	H
ATOM	2085	CA	SER	A	214	8.845	20.189	1.708	1.00	0.00	C
ATOM	2086	CB	SER	A	214	8.446	21.652	1.820	1.00	0.00	C
ATOM	2087	OG	SER	A	214	9.571	22.425	1.376	1.00	0.00	O
ATOM	2088	HG	SER	A	214	10.368	21.953	1.601	1.00	0.00	H
ATOM	2089	C	SER	A	214	7.762	19.150	1.820	1.00	0.00	C
ATOM	2090	O	SER	A	214	6.642	19.421	2.263	1.00	0.00	O
ATOM	2091	N	ASP	A	215	8.110	17.901	1.402	1.00	0.00	N
ATOM	2092	H	ASP	A	215	9.077	17.710	1.226	1.00	0.00	H
ATOM	2093	CA	ASP	A	215	7.200	16.790	1.480	1.00	0.00	C
ATOM	2094	CB	ASP	A	215	7.555	15.684	0.498	1.00	0.00	C
ATOM	2095	CG	ASP	A	215	6.398	14.708	0.422	1.00	0.00	C
ATOM	2096	OD1	ASP	A	215	5.959	14.209	1.457	1.00	0.00	O
ATOM	2097	OD2	ASP	A	215	5.923	14.457	-0.683	1.00	0.00	O
ATOM	2098	C	ASP	A	215	7.418	16.313	2.884	1.00	0.00	C
ATOM	2099	O	ASP	A	215	8.387	15.619	3.205	1.00	0.00	O
ATOM	2100	N	ASN	A	216	6.442	16.684	3.730	1.00	0.00	N
ATOM	2101	H	ASN	A	216	5.704	17.210	3.308	1.00	0.00	H
ATOM	2102	CA	ASN	A	216	6.404	16.417	5.142	1.00	0.00	C
ATOM	2103	CB	ASN	A	216	5.316	17.209	5.862	1.00	0.00	C
ATOM	2104	CG	ASN	A	216	5.076	16.567	7.218	1.00	0.00	C
ATOM	2105	OD1	ASN	A	216	4.182	15.744	7.378	1.00	0.00	O
ATOM	2106	ND2	ASN	A	216	5.905	17.002	8.190	1.00	0.00	N
ATOM	2107	HD21	ASN	A	216	5.837	16.597	9.104	1.00	0.00	H
ATOM	2108	HD22	ASN	A	216	6.592	17.711	8.025	1.00	0.00	H
ATOM	2109	C	ASN	A	216	6.246	14.972	5.482	1.00	0.00	C
ATOM	2110	O	ASN	A	216	6.796	14.517	6.486	1.00	0.00	O
ATOM	2111	N	ILE	A	217	5.498	14.222	4.642	1.00	0.00	N
ATOM	2112	H	ILE	A	217	5.258	14.607	3.749	1.00	0.00	H
ATOM	2113	CA	ILE	A	217	5.238	12.816	4.851	1.00	0.00	C
ATOM	2114	CB	ILE	A	217	4.172	12.334	3.872	1.00	0.00	C
ATOM	2115	CG2	ILE	A	217	3.726	10.913	4.206	1.00	0.00	C
ATOM	2116	CG1	ILE	A	217	2.995	13.310	3.842	1.00	0.00	C
ATOM	2117	CD1	ILE	A	217	1.996	12.984	2.733	1.00	0.00	C
ATOM	2118	C	ILE	A	217	6.555	12.081	4.657	1.00	0.00	C
ATOM	2119	O	ILE	A	217	6.954	11.286	5.513	1.00	0.00	O
ATOM	2120	N	ALA	A	218	7.294	12.425	3.571	1.00	0.00	N
ATOM	2121	H	ALA	A	218	6.904	13.092	2.929	1.00	0.00	H
ATOM	2122	CA	ALA	A	218	8.565	11.829	3.234	1.00	0.00	C
ATOM	2123	CB	ALA	A	218	9.055	12.385	1.906	1.00	0.00	C
ATOM	2124	C	ALA	A	218	9.609	12.123	4.274	1.00	0.00	C

ATOM	2125	O	ALA	A	218	10.396	11.238	4.610	1.00	0.00	O
ATOM	2126	N	LEU	A	219	9.603	13.362	4.828	1.00	0.00	N
ATOM	2127	H	LEU	A	219	8.934	14.020	4.472	1.00	0.00	H
ATOM	2128	CA	LEU	A	219	10.525	13.797	5.852	1.00	0.00	C
ATOM	2129	CB	LEU	A	219	10.354	15.291	6.121	1.00	0.00	C
ATOM	2130	CG	LEU	A	219	10.993	16.142	5.024	1.00	0.00	C
ATOM	2131	CD1	LEU	A	219	10.575	17.611	5.108	1.00	0.00	C
ATOM	2132	CD2	LEU	A	219	12.512	15.973	5.011	1.00	0.00	C
ATOM	2133	C	LEU	A	219	10.403	13.012	7.122	1.00	0.00	C
ATOM	2134	O	LEU	A	219	11.402	12.851	7.823	1.00	0.00	O
ATOM	2135	N	LEU	A	220	9.185	12.511	7.452	1.00	0.00	N
ATOM	2136	H	LEU	A	220	8.401	12.670	6.849	1.00	0.00	H
ATOM	2137	CA	LEU	A	220	8.977	11.730	8.643	1.00	0.00	C
ATOM	2138	CB	LEU	A	220	7.484	11.505	8.879	1.00	0.00	C
ATOM	2139	CG	LEU	A	220	6.698	12.783	9.162	1.00	0.00	C
ATOM	2140	CD1	LEU	A	220	5.193	12.515	9.221	1.00	0.00	C
ATOM	2141	CD2	LEU	A	220	7.209	13.504	10.409	1.00	0.00	C
ATOM	2142	C	LEU	A	220	9.683	10.409	8.490	1.00	0.00	C
ATOM	2143	O	LEU	A	220	10.391	9.975	9.400	1.00	0.00	O
ATOM	2144	N	VAL	A	221	9.532	9.783	7.298	1.00	0.00	N
ATOM	2145	H	VAL	A	221	8.987	10.262	6.608	1.00	0.00	H
ATOM	2146	CA	VAL	A	221	10.089	8.495	6.954	1.00	0.00	C
ATOM	2147	CB	VAL	A	221	9.480	8.033	5.633	1.00	0.00	C
ATOM	2148	CG1	VAL	A	221	10.156	6.776	5.084	1.00	0.00	C
ATOM	2149	CG2	VAL	A	221	7.971	7.863	5.810	1.00	0.00	C
ATOM	2150	C	VAL	A	221	11.596	8.505	6.900	1.00	0.00	C
ATOM	2151	O	VAL	A	221	12.225	7.583	7.427	1.00	0.00	O
ATOM	2152	N	GLN	A	222	12.194	9.556	6.296	1.00	0.00	N
ATOM	2153	H	GLN	A	222	11.599	10.293	5.970	1.00	0.00	H
ATOM	2154	CA	GLN	A	222	13.620	9.665	6.163	1.00	0.00	C
ATOM	2155	CB	GLN	A	222	14.046	10.615	5.034	1.00	0.00	C
ATOM	2156	CG	GLN	A	222	15.446	10.235	4.528	1.00	0.00	C
ATOM	2157	CD	GLN	A	222	16.132	11.352	3.755	1.00	0.00	C
ATOM	2158	OE1	GLN	A	222	17.318	11.601	3.942	1.00	0.00	O
ATOM	2159	NE2	GLN	A	222	15.353	12.018	2.885	1.00	0.00	N
ATOM	2160	HE21	GLN	A	222	15.729	12.810	2.390	1.00	0.00	H
ATOM	2161	HE22	GLN	A	222	14.412	11.762	2.673	1.00	0.00	H
ATOM	2162	C	GLN	A	222	14.245	10.110	7.490	1.00	0.00	C
ATOM	2163	O	GLN	A	222	15.080	9.332	8.020	1.00	0.00	O
ATOM	1	N	MET	B	1	-44.463	17.085	-3.696	1.00	0.00	N
ATOM	2	H	MET	B	1	-44.356	16.713	-4.625	1.00	0.00	H
ATOM	3	CA	MET	B	1	-44.813	18.497	-3.562	1.00	0.00	C
ATOM	4	CB	MET	B	1	-45.063	18.858	-2.097	1.00	0.00	C
ATOM	5	CG	MET	B	1	-45.824	20.174	-1.911	1.00	0.00	C
ATOM	6	SD	MET	B	1	-46.033	20.624	-0.180	1.00	0.00	S
ATOM	7	CE	MET	B	1	-44.314	21.018	0.184	1.00	0.00	C
ATOM	8	C	MET	B	1	-43.765	19.412	-4.168	1.00	0.00	C
ATOM	9	O	MET	B	1	-44.062	20.414	-4.806	1.00	0.00	O
ATOM	10	N	SER	B	2	-42.516	18.990	-3.949	1.00	0.00	N
ATOM	11	H	SER	B	2	-42.332	18.182	-3.390	1.00	0.00	H
ATOM	12	CA	SER	B	2	-41.360	19.737	-4.443	1.00	0.00	C
ATOM	13	CB	SER	B	2	-40.105	19.052	-3.916	1.00	0.00	C
ATOM	14	OG	SER	B	2	-40.443	18.389	-2.686	1.00	0.00	O
ATOM	15	HG	SER	B	2	-39.623	18.344	-2.199	1.00	0.00	H
ATOM	16	C	SER	B	2	-41.297	19.889	-5.953	1.00	0.00	C
ATOM	17	O	SER	B	2	-40.737	20.826	-6.501	1.00	0.00	O
ATOM	18	N	ASP	B	3	-41.936	18.903	-6.594	1.00	0.00	N
ATOM	19	H	ASP	B	3	-42.442	18.206	-6.088	1.00	0.00	H
ATOM	20	CA	ASP	B	3	-42.081	18.889	-8.044	1.00	0.00	C

ATOM	21	CB	ASP	B	3	-42.589	17.498	-8.469	1.00	0.00	C
ATOM	22	CG	ASP	B	3	-43.941	17.105	-7.861	1.00	0.00	C
ATOM	23	OD1	ASP	B	3	-44.486	17.806	-7.012	1.00	0.00	O
ATOM	24	OD2	ASP	B	3	-44.510	16.111	-8.294	1.00	0.00	O
ATOM	25	C	ASP	B	3	-42.936	20.013	-8.621	1.00	0.00	C
ATOM	26	O	ASP	B	3	-42.824	20.393	-9.784	1.00	0.00	O
ATOM	27	N	ASN	B	4	-43.817	20.540	-7.759	1.00	0.00	N
ATOM	28	H	ASN	B	4	-43.809	20.210	-6.815	1.00	0.00	H
ATOM	29	CA	ASN	B	4	-44.629	21.673	-8.198	1.00	0.00	C
ATOM	30	CB	ASN	B	4	-45.810	21.931	-7.262	1.00	0.00	C
ATOM	31	CG	ASN	B	4	-46.722	20.726	-7.219	1.00	0.00	C
ATOM	32	OD1	ASN	B	4	-47.547	20.479	-8.093	1.00	0.00	O
ATOM	33	ND2	ASN	B	4	-46.556	19.978	-6.137	1.00	0.00	N
ATOM	34	HD21	ASN	B	4	-47.137	19.187	-5.932	1.00	0.00	H
ATOM	35	HD22	ASN	B	4	-45.813	20.202	-5.508	1.00	0.00	H
ATOM	36	C	ASN	B	4	-43.788	22.922	-8.308	1.00	0.00	C
ATOM	37	O	ASN	B	4	-42.701	23.019	-7.759	1.00	0.00	O
ATOM	38	N	GLY	B	5	-44.316	23.885	-9.069	1.00	0.00	N
ATOM	39	H	GLY	B	5	-45.188	23.800	-9.548	1.00	0.00	H
ATOM	40	CA	GLY	B	5	-43.389	24.953	-9.414	1.00	0.00	C
ATOM	41	C	GLY	B	5	-42.484	24.497	-10.542	1.00	0.00	C
ATOM	42	O	GLY	B	5	-42.935	23.871	-11.496	1.00	0.00	O
ATOM	43	N	PRO	B	6	-41.182	24.826	-10.385	1.00	0.00	N
ATOM	44	CD	PRO	B	6	-40.592	25.454	-9.207	1.00	0.00	C
ATOM	45	CA	PRO	B	6	-40.214	24.596	-11.461	1.00	0.00	C
ATOM	46	CB	PRO	B	6	-38.892	25.107	-10.865	1.00	0.00	C
ATOM	47	CG	PRO	B	6	-39.101	25.197	-9.353	1.00	0.00	C
ATOM	48	C	PRO	B	6	-40.164	23.191	-12.048	1.00	0.00	C
ATOM	49	O	PRO	B	6	-40.435	23.008	-13.229	1.00	0.00	O
ATOM	50	N	GLN	B	7	-39.805	22.207	-11.201	1.00	0.00	N
ATOM	51	H	GLN	B	7	-39.659	22.418	-10.232	1.00	0.00	H
ATOM	52	CA	GLN	B	7	-39.474	20.879	-11.739	1.00	0.00	C
ATOM	53	CB	GLN	B	7	-39.181	19.845	-10.644	1.00	0.00	C
ATOM	54	CG	GLN	B	7	-37.929	20.100	-9.804	1.00	0.00	C
ATOM	55	CD	GLN	B	7	-38.273	20.916	-8.579	1.00	0.00	C
ATOM	56	OE1	GLN	B	7	-38.611	22.092	-8.653	1.00	0.00	O
ATOM	57	NE2	GLN	B	7	-38.188	20.230	-7.444	1.00	0.00	N
ATOM	58	HE21	GLN	B	7	-38.495	20.606	-6.572	1.00	0.00	H
ATOM	59	HE22	GLN	B	7	-37.828	19.289	-7.406	1.00	0.00	H
ATOM	60	C	GLN	B	7	-40.478	20.268	-12.702	1.00	0.00	C
ATOM	61	O	GLN	B	7	-40.141	19.634	-13.694	1.00	0.00	O
ATOM	62	N	ASN	B	8	-41.751	20.486	-12.367	1.00	0.00	N
ATOM	63	H	ASN	B	8	-41.999	20.942	-11.509	1.00	0.00	H
ATOM	64	CA	ASN	B	8	-42.731	19.902	-13.269	1.00	0.00	C
ATOM	65	CB	ASN	B	8	-43.438	18.748	-12.557	1.00	0.00	C
ATOM	66	CG	ASN	B	8	-44.042	17.751	-13.530	1.00	0.00	C
ATOM	67	OD1	ASN	B	8	-45.142	17.245	-13.326	1.00	0.00	O
ATOM	68	ND2	ASN	B	8	-43.261	17.427	-14.568	1.00	0.00	N
ATOM	69	HD21	ASN	B	8	-43.610	16.774	-15.237	1.00	0.00	H
ATOM	70	HD22	ASN	B	8	-42.344	17.811	-14.686	1.00	0.00	H
ATOM	71	C	ASN	B	8	-43.687	20.897	-13.893	1.00	0.00	C
ATOM	72	O	ASN	B	8	-44.771	20.548	-14.338	1.00	0.00	O
ATOM	73	N	GLN	B	9	-43.226	22.163	-13.902	1.00	0.00	N
ATOM	74	H	GLN	B	9	-42.315	22.354	-13.530	1.00	0.00	H
ATOM	75	CA	GLN	B	9	-43.986	23.276	-14.486	1.00	0.00	C
ATOM	76	CB	GLN	B	9	-43.962	23.235	-16.018	1.00	0.00	C
ATOM	77	CG	GLN	B	9	-42.561	23.152	-16.627	1.00	0.00	C
ATOM	78	CD	GLN	B	9	-42.673	23.105	-18.139	1.00	0.00	C
ATOM	79	OE1	GLN	B	9	-42.131	23.927	-18.861	1.00	0.00	O

ATOM	80	NE2	GLN	B	9	-43.409	22.090	-18.605	1.00	0.00	N
ATOM	81	HE21	GLN	B	9	-43.506	22.021	-19.596	1.00	0.00	H
ATOM	82	HE22	GLN	B	9	-43.854	21.435	-17.998	1.00	0.00	H
ATOM	83	C	GLN	B	9	-45.415	23.439	-13.992	1.00	0.00	C
ATOM	84	O	GLN	B	9	-46.332	23.738	-14.747	1.00	0.00	O
ATOM	85	N	ARG	B	10	-45.582	23.216	-12.683	1.00	0.00	N
ATOM	86	H	ARG	B	10	-44.778	23.126	-12.093	1.00	0.00	H
ATOM	87	CA	ARG	B	10	-46.961	23.328	-12.215	1.00	0.00	C
ATOM	88	CB	ARG	B	10	-47.381	22.139	-11.353	1.00	0.00	C
ATOM	89	CG	ARG	B	10	-47.030	20.785	-11.957	1.00	0.00	C
ATOM	90	CD	ARG	B	10	-47.637	19.637	-11.161	1.00	0.00	C
ATOM	91	NE	ARG	B	10	-46.909	18.403	-11.427	1.00	0.00	N
ATOM	92	HE	ARG	B	10	-46.790	18.087	-12.374	1.00	0.00	H
ATOM	93	CZ	ARG	B	10	-46.278	17.762	-10.424	1.00	0.00	C
ATOM	94	NH1	ARG	B	10	-46.358	18.189	-9.167	1.00	0.00	N
ATOM	95	HH11	ARG	B	10	-46.924	18.975	-8.901	1.00	0.00	H
ATOM	96	HH12	ARG	B	10	-45.823	17.725	-8.441	1.00	0.00	H
ATOM	97	NH2	ARG	B	10	-45.552	16.694	-10.715	1.00	0.00	N
ATOM	98	HH21	ARG	B	10	-45.446	16.400	-11.669	1.00	0.00	H
ATOM	99	HH22	ARG	B	10	-45.087	16.193	-9.972	1.00	0.00	H
ATOM	100	C	ARG	B	10	-47.206	24.593	-11.436	1.00	0.00	C
ATOM	101	O	ARG	B	10	-46.352	25.052	-10.687	1.00	0.00	O
ATOM	102	N	ASN	B	11	-48.428	25.114	-11.601	1.00	0.00	N
ATOM	103	H	ASN	B	11	-49.020	24.765	-12.330	1.00	0.00	H
ATOM	104	CA	ASN	B	11	-48.851	26.252	-10.779	1.00	0.00	C
ATOM	105	CB	ASN	B	11	-50.196	26.830	-11.239	1.00	0.00	C
ATOM	106	CG	ASN	B	11	-50.148	27.251	-12.690	1.00	0.00	C
ATOM	107	OD1	ASN	B	11	-49.964	26.445	-13.589	1.00	0.00	O
ATOM	108	ND2	ASN	B	11	-50.328	28.559	-12.892	1.00	0.00	N
ATOM	109	HD21	ASN	B	11	-50.304	28.878	-13.837	1.00	0.00	H
ATOM	110	HD22	ASN	B	11	-50.486	29.198	-12.141	1.00	0.00	H
ATOM	111	C	ASN	B	11	-48.970	25.899	-9.309	1.00	0.00	C
ATOM	112	O	ASN	B	11	-50.027	25.516	-8.824	1.00	0.00	O
ATOM	113	N	ALA	B	12	-47.833	26.041	-8.615	1.00	0.00	N
ATOM	114	H	ALA	B	12	-47.000	26.278	-9.115	1.00	0.00	H
ATOM	115	CA	ALA	B	12	-47.824	25.743	-7.183	1.00	0.00	C
ATOM	116	CB	ALA	B	12	-46.414	25.914	-6.612	1.00	0.00	C
ATOM	117	C	ALA	B	12	-48.790	26.603	-6.382	1.00	0.00	C
ATOM	118	O	ALA	B	12	-48.694	27.824	-6.344	1.00	0.00	O
ATOM	119	N	PRO	B	13	-49.759	25.915	-5.740	1.00	0.00	N
ATOM	120	CD	PRO	B	13	-49.993	24.473	-5.744	1.00	0.00	C
ATOM	121	CA	PRO	B	13	-50.715	26.660	-4.923	1.00	0.00	C
ATOM	122	CB	PRO	B	13	-51.809	25.613	-4.688	1.00	0.00	C
ATOM	123	CG	PRO	B	13	-51.081	24.267	-4.695	1.00	0.00	C
ATOM	124	C	PRO	B	13	-50.055	27.142	-3.645	1.00	0.00	C
ATOM	125	O	PRO	B	13	-49.165	26.501	-3.100	1.00	0.00	O
ATOM	126	N	ARG	B	14	-50.548	28.295	-3.163	1.00	0.00	N
ATOM	127	H	ARG	B	14	-51.227	28.803	-3.686	1.00	0.00	H
ATOM	128	CA	ARG	B	14	-50.017	28.734	-1.872	1.00	0.00	C
ATOM	129	CB	ARG	B	14	-50.547	30.114	-1.471	1.00	0.00	C
ATOM	130	CG	ARG	B	14	-50.218	31.215	-2.483	1.00	0.00	C
ATOM	131	CD	ARG	B	14	-50.450	32.631	-1.943	1.00	0.00	C
ATOM	132	NE	ARG	B	14	-51.838	32.853	-1.528	1.00	0.00	N
ATOM	133	HE	ARG	B	14	-52.545	32.330	-2.007	1.00	0.00	H
ATOM	134	CZ	ARG	B	14	-52.132	33.784	-0.591	1.00	0.00	C
ATOM	135	NH1	ARG	B	14	-51.160	34.493	-0.014	1.00	0.00	N
ATOM	136	HH11	ARG	B	14	-50.204	34.338	-0.269	1.00	0.00	H
ATOM	137	HH12	ARG	B	14	-51.347	35.187	0.683	1.00	0.00	H
ATOM	138	NH2	ARG	B	14	-53.403	33.991	-0.248	1.00	0.00	N

ATOM	139	HH21	ARG	B	14	-54.141	33.473	-0.682	1.00	0.00	H
ATOM	140	HH22	ARG	B	14	-53.657	34.665	0.448	1.00	0.00	H
ATOM	141	C	ARG	B	14	-50.280	27.733	-0.760	1.00	0.00	C
ATOM	142	O	ARG	B	14	-51.354	27.139	-0.671	1.00	0.00	O
ATOM	143	N	ILE	B	15	-49.232	27.565	0.050	1.00	0.00	N
ATOM	144	H	ILE	B	15	-48.426	28.141	-0.073	1.00	0.00	H
ATOM	145	CA	ILE	B	15	-49.314	26.686	1.212	1.00	0.00	C
ATOM	146	CB	ILE	B	15	-47.936	26.014	1.396	1.00	0.00	C
ATOM	147	CG2	ILE	B	15	-47.904	24.938	2.485	1.00	0.00	C
ATOM	148	CG1	ILE	B	15	-47.468	25.418	0.062	1.00	0.00	C
ATOM	149	CD1	ILE	B	15	-46.042	24.863	0.104	1.00	0.00	C
ATOM	150	C	ILE	B	15	-49.756	27.540	2.398	1.00	0.00	C
ATOM	151	O	ILE	B	15	-49.712	28.759	2.307	1.00	0.00	O
ATOM	152	N	THR	B	16	-50.237	26.877	3.468	1.00	0.00	N
ATOM	153	H	THR	B	16	-50.132	25.887	3.511	1.00	0.00	H
ATOM	154	CA	THR	B	16	-50.550	27.603	4.705	1.00	0.00	C
ATOM	155	CB	THR	B	16	-49.229	27.988	5.393	1.00	0.00	C
ATOM	156	OG1	THR	B	16	-48.249	26.972	5.124	1.00	0.00	O
ATOM	157	HG1	THR	B	16	-48.590	26.156	5.483	1.00	0.00	H
ATOM	158	CG2	THR	B	16	-49.372	28.190	6.905	1.00	0.00	C
ATOM	159	C	THR	B	16	-51.550	28.761	4.602	1.00	0.00	C
ATOM	160	O	THR	B	16	-51.435	29.815	5.213	1.00	0.00	O
ATOM	161	N	PHE	B	17	-52.584	28.487	3.791	1.00	0.00	N
ATOM	162	H	PHE	B	17	-52.621	27.615	3.314	1.00	0.00	H
ATOM	163	CA	PHE	B	17	-53.694	29.431	3.685	1.00	0.00	C
ATOM	164	CB	PHE	B	17	-53.563	30.330	2.446	1.00	0.00	C
ATOM	165	CG	PHE	B	17	-52.496	31.379	2.660	1.00	0.00	C
ATOM	166	CD1	PHE	B	17	-51.258	31.251	1.996	1.00	0.00	C
ATOM	167	CD2	PHE	B	17	-52.749	32.465	3.527	1.00	0.00	C
ATOM	168	CE1	PHE	B	17	-50.246	32.204	2.222	1.00	0.00	C
ATOM	169	CE2	PHE	B	17	-51.741	33.422	3.752	1.00	0.00	C
ATOM	170	CZ	PHE	B	17	-50.495	33.275	3.105	1.00	0.00	C
ATOM	171	C	PHE	B	17	-55.018	28.700	3.644	1.00	0.00	C
ATOM	172	O	PHE	B	17	-55.119	27.573	3.166	1.00	0.00	O
ATOM	173	N	GLY	B	18	-56.032	29.395	4.160	1.00	0.00	N
ATOM	174	H	GLY	B	18	-55.917	30.316	4.537	1.00	0.00	H
ATOM	175	CA	GLY	B	18	-57.365	28.810	4.179	1.00	0.00	C
ATOM	176	C	GLY	B	18	-58.343	29.875	4.607	1.00	0.00	C
ATOM	177	O	GLY	B	18	-58.086	31.059	4.432	1.00	0.00	O
ATOM	178	N	GLY	B	19	-59.453	29.404	5.192	1.00	0.00	N
ATOM	179	H	GLY	B	19	-59.551	28.428	5.363	1.00	0.00	H
ATOM	180	CA	GLY	B	19	-60.310	30.379	5.862	1.00	0.00	C
ATOM	181	C	GLY	B	19	-59.705	30.786	7.195	1.00	0.00	C
ATOM	182	O	GLY	B	19	-58.634	30.319	7.569	1.00	0.00	O
ATOM	183	N	PRO	B	20	-60.437	31.670	7.907	1.00	0.00	N
ATOM	184	CD	PRO	B	20	-61.729	32.239	7.540	1.00	0.00	C
ATOM	185	CA	PRO	B	20	-59.940	32.140	9.203	1.00	0.00	C
ATOM	186	CB	PRO	B	20	-60.961	33.224	9.569	1.00	0.00	C
ATOM	187	CG	PRO	B	20	-62.247	32.840	8.838	1.00	0.00	C
ATOM	188	C	PRO	B	20	-59.804	31.018	10.223	1.00	0.00	C
ATOM	189	O	PRO	B	20	-60.190	29.871	9.996	1.00	0.00	O
ATOM	190	N	SER	B	21	-59.197	31.396	11.355	1.00	0.00	N
ATOM	191	H	SER	B	21	-58.886	32.335	11.506	1.00	0.00	H
ATOM	192	CA	SER	B	21	-58.858	30.413	12.381	1.00	0.00	C
ATOM	193	CB	SER	B	21	-58.264	31.157	13.572	1.00	0.00	C
ATOM	194	OG	SER	B	21	-57.436	32.217	13.073	1.00	0.00	O
ATOM	195	HG	SER	B	21	-56.984	32.568	13.840	1.00	0.00	H
ATOM	196	C	SER	B	21	-59.999	29.500	12.792	1.00	0.00	C
ATOM	197	O	SER	B	21	-59.908	28.280	12.739	1.00	0.00	O

ATOM	198	N	ASP	B	22	-61.105	30.161	13.143	1.00	0.00	N
ATOM	199	H	ASP	B	22	-61.133	31.162	13.087	1.00	0.00	H
ATOM	200	CA	ASP	B	22	-62.382	29.489	13.393	1.00	0.00	C
ATOM	201	CB	ASP	B	22	-63.484	30.559	13.523	1.00	0.00	C
ATOM	202	CG	ASP	B	22	-63.401	31.556	12.371	1.00	0.00	C
ATOM	203	OD1	ASP	B	22	-62.566	32.455	12.429	1.00	0.00	O
ATOM	204	OD2	ASP	B	22	-64.114	31.383	11.388	1.00	0.00	O
ATOM	205	C	ASP	B	22	-62.736	28.422	12.359	1.00	0.00	C
ATOM	206	O	ASP	B	22	-62.839	27.235	12.651	1.00	0.00	O
ATOM	207	N	SER	B	23	-62.847	28.893	11.112	1.00	0.00	N
ATOM	208	H	SER	B	23	-62.768	29.884	10.982	1.00	0.00	H
ATOM	209	CA	SER	B	23	-63.219	28.011	10.012	1.00	0.00	C
ATOM	210	CB	SER	B	23	-63.462	28.853	8.761	1.00	0.00	C
ATOM	211	OG	SER	B	23	-64.506	29.810	8.986	1.00	0.00	O
ATOM	212	HG	SER	B	23	-64.325	30.289	9.798	1.00	0.00	H
ATOM	213	C	SER	B	23	-62.231	26.889	9.725	1.00	0.00	C
ATOM	214	O	SER	B	23	-62.555	25.870	9.129	1.00	0.00	O
ATOM	215	N	THR	B	24	-60.990	27.106	10.175	1.00	0.00	N
ATOM	216	H	THR	B	24	-60.748	27.941	10.674	1.00	0.00	H
ATOM	217	CA	THR	B	24	-60.029	26.034	9.955	1.00	0.00	C
ATOM	218	CB	THR	B	24	-58.750	26.617	9.356	1.00	0.00	C
ATOM	219	OG1	THR	B	24	-58.324	27.768	10.102	1.00	0.00	O
ATOM	220	HG1	THR	B	24	-58.878	28.495	9.810	1.00	0.00	H
ATOM	221	CG2	THR	B	24	-58.929	26.972	7.880	1.00	0.00	C
ATOM	222	C	THR	B	24	-59.728	25.185	11.181	1.00	0.00	C
ATOM	223	O	THR	B	24	-58.745	24.448	11.226	1.00	0.00	O
ATOM	224	N	GLY	B	25	-60.588	25.332	12.208	1.00	0.00	N
ATOM	225	H	GLY	B	25	-61.365	25.962	12.138	1.00	0.00	H
ATOM	226	CA	GLY	B	25	-60.349	24.597	13.454	1.00	0.00	C
ATOM	227	C	GLY	B	25	-58.992	24.880	14.088	1.00	0.00	C
ATOM	228	O	GLY	B	25	-58.347	24.041	14.709	1.00	0.00	O
ATOM	229	N	SER	B	26	-58.556	26.113	13.850	1.00	0.00	N
ATOM	230	H	SER	B	26	-59.161	26.748	13.375	1.00	0.00	H
ATOM	231	CA	SER	B	26	-57.248	26.546	14.316	1.00	0.00	C
ATOM	232	CB	SER	B	26	-56.508	27.238	13.167	1.00	0.00	C
ATOM	233	OG	SER	B	26	-56.692	26.485	11.952	1.00	0.00	O
ATOM	234	HG	SER	B	26	-57.224	27.062	11.398	1.00	0.00	H
ATOM	235	C	SER	B	26	-57.403	27.436	15.528	1.00	0.00	C
ATOM	236	O	SER	B	26	-57.162	28.637	15.501	1.00	0.00	O
ATOM	237	N	ASN	B	27	-57.884	26.769	16.590	1.00	0.00	N
ATOM	238	H	ASN	B	27	-57.976	25.776	16.528	1.00	0.00	H
ATOM	239	CA	ASN	B	27	-58.223	27.510	17.807	1.00	0.00	C
ATOM	240	CB	ASN	B	27	-58.859	26.573	18.841	1.00	0.00	C
ATOM	241	CG	ASN	B	27	-59.496	27.395	19.946	1.00	0.00	C
ATOM	242	OD1	ASN	B	27	-59.929	28.517	19.739	1.00	0.00	O
ATOM	243	ND2	ASN	B	27	-59.513	26.800	21.141	1.00	0.00	N
ATOM	244	HD21	ASN	B	27	-59.899	27.324	21.899	1.00	0.00	H
ATOM	245	HD22	ASN	B	27	-59.159	25.879	21.289	1.00	0.00	H
ATOM	246	C	ASN	B	27	-57.035	28.253	18.395	1.00	0.00	C
ATOM	247	O	ASN	B	27	-55.902	27.791	18.314	1.00	0.00	O
ATOM	248	N	GLN	B	28	-57.345	29.426	18.954	1.00	0.00	N
ATOM	249	H	GLN	B	28	-58.300	29.695	19.105	1.00	0.00	H
ATOM	250	CA	GLN	B	28	-56.259	30.303	19.365	1.00	0.00	C
ATOM	251	CB	GLN	B	28	-55.839	31.202	18.200	1.00	0.00	C
ATOM	252	CG	GLN	B	28	-56.925	32.136	17.658	1.00	0.00	C
ATOM	253	CD	GLN	B	28	-56.285	33.072	16.658	1.00	0.00	C
ATOM	254	OE1	GLN	B	28	-56.182	32.785	15.471	1.00	0.00	O
ATOM	255	NE2	GLN	B	28	-55.809	34.195	17.194	1.00	0.00	N
ATOM	256	HE21	GLN	B	28	-55.306	34.874	16.656	1.00	0.00	H

ATOM	257	HE22	GLN	B	28	-55.921	34.358	18.177	1.00	0.00	H
ATOM	258	C	GLN	B	28	-56.627	31.130	20.576	1.00	0.00	C
ATOM	259	O	GLN	B	28	-57.771	31.166	21.007	1.00	0.00	O
ATOM	260	N	ASN	B	29	-55.599	31.807	21.098	1.00	0.00	N
ATOM	261	H	ASN	B	29	-54.709	31.765	20.644	1.00	0.00	H
ATOM	262	CA	ASN	B	29	-55.919	32.874	22.044	1.00	0.00	C
ATOM	263	CB	ASN	B	29	-54.862	32.987	23.156	1.00	0.00	C
ATOM	264	CG	ASN	B	29	-53.449	33.113	22.613	1.00	0.00	C
ATOM	265	OD1	ASN	B	29	-52.758	32.127	22.382	1.00	0.00	O
ATOM	266	ND2	ASN	B	29	-53.004	34.360	22.474	1.00	0.00	N
ATOM	267	HD21	ASN	B	29	-52.058	34.551	22.230	1.00	0.00	H
ATOM	268	HD22	ASN	B	29	-53.591	35.173	22.538	1.00	0.00	H
ATOM	269	C	ASN	B	29	-56.104	34.176	21.292	1.00	0.00	C
ATOM	270	O	ASN	B	29	-56.038	34.198	20.070	1.00	0.00	O
ATOM	271	N	GLY	B	30	-56.312	35.264	22.056	1.00	0.00	N
ATOM	272	H	GLY	B	30	-56.432	35.198	23.043	1.00	0.00	H
ATOM	273	CA	GLY	B	30	-56.323	36.575	21.396	1.00	0.00	C
ATOM	274	C	GLY	B	30	-55.054	36.807	20.597	1.00	0.00	C
ATOM	275	O	GLY	B	30	-53.981	36.375	21.010	1.00	0.00	O
ATOM	276	N	GLU	B	31	-55.258	37.414	19.416	1.00	0.00	N
ATOM	277	H	GLU	B	31	-56.093	37.965	19.376	1.00	0.00	H
ATOM	278	CA	GLU	B	31	-54.175	37.726	18.481	1.00	0.00	C
ATOM	279	CB	GLU	B	31	-53.253	38.830	19.039	1.00	0.00	C
ATOM	280	CG	GLU	B	31	-53.930	40.178	19.362	1.00	0.00	C
ATOM	281	CD	GLU	B	31	-54.946	40.028	20.486	1.00	0.00	C
ATOM	282	OE1	GLU	B	31	-56.144	40.042	20.203	1.00	0.00	O
ATOM	283	OE2	GLU	B	31	-54.548	39.810	21.629	1.00	0.00	O
ATOM	284	C	GLU	B	31	-53.399	36.528	17.948	1.00	0.00	C
ATOM	285	O	GLU	B	31	-53.594	36.103	16.815	1.00	0.00	O
ATOM	286	N	ARG	B	32	-52.511	35.992	18.803	1.00	0.00	N
ATOM	287	H	ARG	B	32	-52.532	36.348	19.737	1.00	0.00	H
ATOM	288	CA	ARG	B	32	-51.691	34.837	18.429	1.00	0.00	C
ATOM	289	CB	ARG	B	32	-50.825	34.421	19.619	1.00	0.00	C
ATOM	290	CG	ARG	B	32	-49.826	33.304	19.307	1.00	0.00	C
ATOM	291	CD	ARG	B	32	-49.118	32.795	20.562	1.00	0.00	C
ATOM	292	NE	ARG	B	32	-50.075	32.231	21.513	1.00	0.00	N
ATOM	293	HE	ARG	B	32	-51.059	32.225	21.299	1.00	0.00	H
ATOM	294	CZ	ARG	B	32	-49.663	31.698	22.680	1.00	0.00	C
ATOM	295	NH1	ARG	B	32	-48.375	31.669	22.995	1.00	0.00	N
ATOM	296	HH11	ARG	B	32	-47.674	32.071	22.409	1.00	0.00	H
ATOM	297	HH12	ARG	B	32	-48.106	31.217	23.853	1.00	0.00	H
ATOM	298	NH2	ARG	B	32	-50.555	31.201	23.524	1.00	0.00	N
ATOM	299	HH21	ARG	B	32	-51.534	31.240	23.295	1.00	0.00	H
ATOM	300	HH22	ARG	B	32	-50.281	30.791	24.408	1.00	0.00	H
ATOM	301	C	ARG	B	32	-52.482	33.649	17.902	1.00	0.00	C
ATOM	302	O	ARG	B	32	-53.184	32.954	18.629	1.00	0.00	O
ATOM	303	N	SER	B	33	-52.313	33.466	16.590	1.00	0.00	N
ATOM	304	H	SER	B	33	-51.699	34.041	16.049	1.00	0.00	H
ATOM	305	CA	SER	B	33	-53.060	32.437	15.885	1.00	0.00	C
ATOM	306	CB	SER	B	33	-52.990	32.757	14.390	1.00	0.00	C
ATOM	307	OG	SER	B	33	-51.712	33.337	14.084	1.00	0.00	O
ATOM	308	HG	SER	B	33	-51.805	33.742	13.229	1.00	0.00	H
ATOM	309	C	SER	B	33	-52.636	31.015	16.199	1.00	0.00	C
ATOM	310	O	SER	B	33	-51.553	30.751	16.716	1.00	0.00	O
ATOM	311	N	GLY	B	34	-53.540	30.089	15.836	1.00	0.00	N
ATOM	312	H	GLY	B	34	-54.422	30.372	15.458	1.00	0.00	H
ATOM	313	CA	GLY	B	34	-53.199	28.672	15.968	1.00	0.00	C
ATOM	314	C	GLY	B	34	-52.291	28.184	14.851	1.00	0.00	C
ATOM	315	O	GLY	B	34	-52.665	27.404	13.982	1.00	0.00	O

ATOM	316	N	ALA	B	35	-51.058	28.706	14.899	1.00	0.00	N
ATOM	317	H	ALA	B	35	-50.828	29.302	15.671	1.00	0.00	H
ATOM	318	CA	ALA	B	35	-50.127	28.457	13.805	1.00	0.00	C
ATOM	319	CB	ALA	B	35	-49.007	29.500	13.813	1.00	0.00	C
ATOM	320	C	ALA	B	35	-49.527	27.064	13.813	1.00	0.00	C
ATOM	321	O	ALA	B	35	-48.454	26.807	14.364	1.00	0.00	O
ATOM	322	N	ARG	B	36	-50.273	26.172	13.147	1.00	0.00	N
ATOM	323	H	ARG	B	36	-51.174	26.471	12.823	1.00	0.00	H
ATOM	324	CA	ARG	B	36	-49.763	24.822	12.916	1.00	0.00	C
ATOM	325	CB	ARG	B	36	-50.801	23.926	12.236	1.00	0.00	C
ATOM	326	CG	ARG	B	36	-52.159	23.803	12.922	1.00	0.00	C
ATOM	327	CD	ARG	B	36	-53.062	22.859	12.120	1.00	0.00	C
ATOM	328	NE	ARG	B	36	-54.427	22.832	12.643	1.00	0.00	N
ATOM	329	HE	ARG	B	36	-54.627	22.249	13.431	1.00	0.00	H
ATOM	330	CZ	ARG	B	36	-55.373	23.638	12.113	1.00	0.00	C
ATOM	331	NH1	ARG	B	36	-55.118	24.423	11.073	1.00	0.00	N
ATOM	332	HH11	ARG	B	36	-54.300	24.304	10.497	1.00	0.00	H
ATOM	333	HH12	ARG	B	36	-55.761	25.166	10.859	1.00	0.00	H
ATOM	334	NH2	ARG	B	36	-56.587	23.662	12.637	1.00	0.00	N
ATOM	335	HH21	ARG	B	36	-56.855	23.171	13.469	1.00	0.00	H
ATOM	336	HH22	ARG	B	36	-57.298	24.214	12.185	1.00	0.00	H
ATOM	337	C	ARG	B	36	-48.520	24.809	12.049	1.00	0.00	C
ATOM	338	O	ARG	B	36	-48.570	25.021	10.846	1.00	0.00	O
ATOM	339	N	SER	B	37	-47.397	24.493	12.699	1.00	0.00	N
ATOM	340	H	SER	B	37	-47.353	24.453	13.696	1.00	0.00	H
ATOM	341	CA	SER	B	37	-46.150	24.413	11.939	1.00	0.00	C
ATOM	342	CB	SER	B	37	-45.001	24.179	12.917	1.00	0.00	C
ATOM	343	OG	SER	B	37	-45.282	24.853	14.158	1.00	0.00	O
ATOM	344	HG	SER	B	37	-44.473	24.788	14.654	1.00	0.00	H
ATOM	345	C	SER	B	37	-46.117	23.396	10.796	1.00	0.00	C
ATOM	346	O	SER	B	37	-45.274	23.435	9.913	1.00	0.00	O
ATOM	347	N	LYS	B	38	-47.082	22.464	10.856	1.00	0.00	N
ATOM	348	H	LYS	B	38	-47.783	22.504	11.563	1.00	0.00	H
ATOM	349	CA	LYS	B	38	-47.164	21.493	9.770	1.00	0.00	C
ATOM	350	CB	LYS	B	38	-47.365	20.082	10.330	1.00	0.00	C
ATOM	351	CG	LYS	B	38	-46.266	19.589	11.276	1.00	0.00	C
ATOM	352	CD	LYS	B	38	-46.561	18.172	11.775	1.00	0.00	C
ATOM	353	CE	LYS	B	38	-45.491	17.622	12.721	1.00	0.00	C
ATOM	354	NZ	LYS	B	38	-45.863	16.262	13.142	1.00	0.00	N
ATOM	355	HZ1	LYS	B	38	-45.141	15.886	13.789	1.00	0.00	H
ATOM	356	HZ2	LYS	B	38	-46.782	16.284	13.628	1.00	0.00	H
ATOM	357	HZ3	LYS	B	38	-45.932	15.645	12.307	1.00	0.00	H
ATOM	358	C	LYS	B	38	-48.255	21.786	8.751	1.00	0.00	C
ATOM	359	O	LYS	B	38	-48.759	20.876	8.105	1.00	0.00	O
ATOM	360	N	GLN	B	39	-48.639	23.071	8.646	1.00	0.00	N
ATOM	361	H	GLN	B	39	-48.182	23.808	9.148	1.00	0.00	H
ATOM	362	CA	GLN	B	39	-49.760	23.362	7.750	1.00	0.00	C
ATOM	363	CB	GLN	B	39	-50.496	24.636	8.188	1.00	0.00	C
ATOM	364	CG	GLN	B	39	-51.865	24.859	7.524	1.00	0.00	C
ATOM	365	CD	GLN	B	39	-52.849	23.795	7.964	1.00	0.00	C
ATOM	366	OE1	GLN	B	39	-53.418	23.852	9.049	1.00	0.00	O
ATOM	367	NE2	GLN	B	39	-53.041	22.818	7.070	1.00	0.00	N
ATOM	368	HE21	GLN	B	39	-53.684	22.087	7.286	1.00	0.00	H
ATOM	369	HE22	GLN	B	39	-52.569	22.777	6.184	1.00	0.00	H
ATOM	370	C	GLN	B	39	-49.422	23.396	6.265	1.00	0.00	C
ATOM	371	O	GLN	B	39	-49.352	24.439	5.620	1.00	0.00	O
ATOM	372	N	ARG	B	40	-49.253	22.178	5.738	1.00	0.00	N
ATOM	373	H	ARG	B	40	-49.347	21.380	6.334	1.00	0.00	H
ATOM	374	CA	ARG	B	40	-49.237	22.062	4.287	1.00	0.00	C

ATOM	375	CB	ARG	B	40	-48.558	20.763	3.846	1.00	0.00	C
ATOM	376	CG	ARG	B	40	-47.111	20.629	4.320	1.00	0.00	C
ATOM	377	CD	ARG	B	40	-46.469	19.341	3.804	1.00	0.00	C
ATOM	378	NE	ARG	B	40	-45.126	19.162	4.353	1.00	0.00	N
ATOM	379	HE	ARG	B	40	-44.938	19.574	5.246	1.00	0.00	H
ATOM	380	CZ	ARG	B	40	-44.225	18.394	3.703	1.00	0.00	C
ATOM	381	NH1	ARG	B	40	-44.499	17.864	2.520	1.00	0.00	N
ATOM	382	HH11	ARG	B	40	-45.379	17.973	2.040	1.00	0.00	H
ATOM	383	HH12	ARG	B	40	-43.800	17.315	2.055	1.00	0.00	H
ATOM	384	NH2	ARG	B	40	-43.035	18.148	4.241	1.00	0.00	N
ATOM	385	HH21	ARG	B	40	-42.757	18.524	5.123	1.00	0.00	H
ATOM	386	HH22	ARG	B	40	-42.392	17.557	3.737	1.00	0.00	H
ATOM	387	C	ARG	B	40	-50.650	22.128	3.747	1.00	0.00	C
ATOM	388	O	ARG	B	40	-51.621	22.123	4.497	1.00	0.00	O
ATOM	389	N	ARG	B	41	-50.717	22.187	2.412	1.00	0.00	N
ATOM	390	H	ARG	B	41	-49.892	22.247	1.849	1.00	0.00	H
ATOM	391	CA	ARG	B	41	-52.016	22.042	1.765	1.00	0.00	C
ATOM	392	CB	ARG	B	41	-52.499	23.371	1.186	1.00	0.00	C
ATOM	393	CG	ARG	B	41	-52.640	24.494	2.212	1.00	0.00	C
ATOM	394	CD	ARG	B	41	-53.020	25.820	1.560	1.00	0.00	C
ATOM	395	NE	ARG	B	41	-54.402	25.835	1.092	1.00	0.00	N
ATOM	396	HE	ARG	B	41	-55.095	25.893	1.818	1.00	0.00	H
ATOM	397	CZ	ARG	B	41	-54.690	25.804	-0.225	1.00	0.00	C
ATOM	398	NH1	ARG	B	41	-53.717	25.896	-1.132	1.00	0.00	N
ATOM	399	HH11	ARG	B	41	-52.771	26.095	-0.838	1.00	0.00	H
ATOM	400	HH12	ARG	B	41	-53.880	25.786	-2.110	1.00	0.00	H
ATOM	401	NH2	ARG	B	41	-55.960	25.683	-0.606	1.00	0.00	N
ATOM	402	HH21	ARG	B	41	-56.683	25.618	0.084	1.00	0.00	H
ATOM	403	HH22	ARG	B	41	-56.225	25.651	-1.570	1.00	0.00	H
ATOM	404	C	ARG	B	41	-51.887	21.042	0.640	1.00	0.00	C
ATOM	405	O	ARG	B	41	-51.256	21.329	-0.369	1.00	0.00	O
ATOM	406	N	PRO	B	42	-52.483	19.849	0.846	1.00	0.00	N
ATOM	407	CD	PRO	B	42	-53.240	19.431	2.022	1.00	0.00	C
ATOM	408	CA	PRO	B	42	-52.410	18.824	-0.200	1.00	0.00	C
ATOM	409	CB	PRO	B	42	-53.051	17.613	0.492	1.00	0.00	C
ATOM	410	CG	PRO	B	42	-53.988	18.190	1.554	1.00	0.00	C
ATOM	411	C	PRO	B	42	-53.123	19.270	-1.477	1.00	0.00	C
ATOM	412	O	PRO	B	42	-54.200	19.845	-1.394	1.00	0.00	O
ATOM	413	N	GLN	B	43	-52.534	19.035	-2.673	1.00	0.00	N
ATOM	414	H	GLN	B	43	-53.088	19.377	-3.428	1.00	0.00	H
ATOM	415	CA	GLN	B	43	-51.217	18.448	-2.960	1.00	0.00	C
ATOM	416	CB	GLN	B	43	-50.062	19.408	-2.633	1.00	0.00	C
ATOM	417	CG	GLN	B	43	-49.929	20.542	-3.652	1.00	0.00	C
ATOM	418	CD	GLN	B	43	-49.467	19.963	-4.974	1.00	0.00	C
ATOM	419	OE1	GLN	B	43	-48.888	18.887	-5.039	1.00	0.00	O
ATOM	420	NE2	GLN	B	43	-49.724	20.730	-6.036	1.00	0.00	N
ATOM	421	HE21	GLN	B	43	-49.353	20.442	-6.922	1.00	0.00	H
ATOM	422	HE22	GLN	B	43	-50.245	21.578	-5.974	1.00	0.00	H
ATOM	423	C	GLN	B	43	-50.935	17.040	-2.472	1.00	0.00	C
ATOM	424	O	GLN	B	43	-49.958	16.760	-1.789	1.00	0.00	O
ATOM	425	N	GLY	B	44	-51.844	16.147	-2.889	1.00	0.00	N
ATOM	426	H	GLY	B	44	-52.592	16.433	-3.485	1.00	0.00	H
ATOM	427	CA	GLY	B	44	-51.593	14.730	-2.621	1.00	0.00	C
ATOM	428	C	GLY	B	44	-50.725	14.078	-3.686	1.00	0.00	C
ATOM	429	O	GLY	B	44	-51.060	13.064	-4.279	1.00	0.00	O
ATOM	430	N	LEU	B	45	-49.594	14.748	-3.919	1.00	0.00	N
ATOM	431	H	LEU	B	45	-49.332	15.488	-3.300	1.00	0.00	H
ATOM	432	CA	LEU	B	45	-48.628	14.247	-4.889	1.00	0.00	C
ATOM	433	CB	LEU	B	45	-48.253	15.420	-5.809	1.00	0.00	C

ATOM	434	CG	LEU	B	45	-49.406	15.790	-6.750	1.00	0.00	C
ATOM	435	CD1	LEU	B	45	-49.079	17.003	-7.618	1.00	0.00	C
ATOM	436	CD2	LEU	B	45	-49.854	14.603	-7.607	1.00	0.00	C
ATOM	437	C	LEU	B	45	-47.469	13.669	-4.092	1.00	0.00	C
ATOM	438	O	LEU	B	45	-47.435	13.868	-2.880	1.00	0.00	O
ATOM	439	N	PRO	B	46	-46.523	12.937	-4.744	1.00	0.00	N
ATOM	440	CD	PRO	B	46	-46.472	12.541	-6.151	1.00	0.00	C
ATOM	441	CA	PRO	B	46	-45.365	12.445	-3.984	1.00	0.00	C
ATOM	442	CB	PRO	B	46	-44.573	11.652	-5.032	1.00	0.00	C
ATOM	443	CG	PRO	B	46	-45.006	12.209	-6.386	1.00	0.00	C
ATOM	444	C	PRO	B	46	-44.573	13.561	-3.314	1.00	0.00	C
ATOM	445	O	PRO	B	46	-43.752	14.243	-3.910	1.00	0.00	O
ATOM	446	N	ASN	B	47	-44.892	13.710	-2.020	1.00	0.00	N
ATOM	447	H	ASN	B	47	-45.596	13.097	-1.665	1.00	0.00	H
ATOM	448	CA	ASN	B	47	-44.433	14.860	-1.239	1.00	0.00	C
ATOM	449	CB	ASN	B	47	-45.293	14.981	0.023	1.00	0.00	C
ATOM	450	CG	ASN	B	47	-46.258	16.149	-0.066	1.00	0.00	C
ATOM	451	OD1	ASN	B	47	-46.105	17.154	0.618	1.00	0.00	O
ATOM	452	ND2	ASN	B	47	-47.287	15.960	-0.895	1.00	0.00	N
ATOM	453	HD21	ASN	B	47	-48.030	16.627	-0.980	1.00	0.00	H
ATOM	454	HD22	ASN	B	47	-47.363	15.140	-1.464	1.00	0.00	H
ATOM	455	C	ASN	B	47	-42.959	14.827	-0.877	1.00	0.00	C
ATOM	456	O	ASN	B	47	-42.598	14.583	0.269	1.00	0.00	O
ATOM	457	N	ASN	B	48	-42.134	15.080	-1.905	1.00	0.00	N
ATOM	458	H	ASN	B	48	-42.582	15.308	-2.773	1.00	0.00	H
ATOM	459	CA	ASN	B	48	-40.675	14.887	-1.900	1.00	0.00	C
ATOM	460	CB	ASN	B	48	-40.183	15.073	-3.343	1.00	0.00	C
ATOM	461	CG	ASN	B	48	-38.750	14.612	-3.516	1.00	0.00	C
ATOM	462	OD1	ASN	B	48	-38.197	13.879	-2.702	1.00	0.00	O
ATOM	463	ND2	ASN	B	48	-38.184	15.032	-4.650	1.00	0.00	N
ATOM	464	HD21	ASN	B	48	-37.319	14.656	-4.972	1.00	0.00	H
ATOM	465	HD22	ASN	B	48	-38.569	15.746	-5.248	1.00	0.00	H
ATOM	466	C	ASN	B	48	-39.882	15.762	-0.930	1.00	0.00	C
ATOM	467	O	ASN	B	48	-39.093	16.617	-1.309	1.00	0.00	O
ATOM	468	N	THR	B	49	-40.161	15.552	0.362	1.00	0.00	N
ATOM	469	H	THR	B	49	-40.702	14.749	0.612	1.00	0.00	H
ATOM	470	CA	THR	B	49	-39.794	16.594	1.317	1.00	0.00	C
ATOM	471	CB	THR	B	49	-41.010	17.491	1.611	1.00	0.00	C
ATOM	472	OG1	THR	B	49	-42.136	16.702	2.039	1.00	0.00	O
ATOM	473	HG1	THR	B	49	-42.149	15.931	1.467	1.00	0.00	H
ATOM	474	CG2	THR	B	49	-41.420	18.378	0.435	1.00	0.00	C
ATOM	475	C	THR	B	49	-39.193	16.094	2.620	1.00	0.00	C
ATOM	476	O	THR	B	49	-39.188	16.798	3.624	1.00	0.00	O
ATOM	477	N	ALA	B	50	-38.729	14.836	2.576	1.00	0.00	N
ATOM	478	H	ALA	B	50	-38.645	14.360	1.698	1.00	0.00	H
ATOM	479	CA	ALA	B	50	-38.039	14.347	3.769	1.00	0.00	C
ATOM	480	CB	ALA	B	50	-38.045	12.815	3.794	1.00	0.00	C
ATOM	481	C	ALA	B	50	-36.612	14.866	3.783	1.00	0.00	C
ATOM	482	O	ALA	B	50	-36.211	15.601	2.892	1.00	0.00	O
ATOM	483	N	SER	B	51	-35.852	14.463	4.801	1.00	0.00	N
ATOM	484	H	SER	B	51	-36.173	13.814	5.487	1.00	0.00	H
ATOM	485	CA	SER	B	51	-34.442	14.812	4.680	1.00	0.00	C
ATOM	486	CB	SER	B	51	-33.885	15.219	6.040	1.00	0.00	C
ATOM	487	OG	SER	B	51	-34.157	16.613	6.205	1.00	0.00	O
ATOM	488	HG	SER	B	51	-33.718	17.022	5.462	1.00	0.00	H
ATOM	489	C	SER	B	51	-33.638	13.720	4.014	1.00	0.00	C
ATOM	490	O	SER	B	51	-34.135	12.623	3.796	1.00	0.00	O
ATOM	491	N	TRP	B	52	-32.387	14.063	3.680	1.00	0.00	N
ATOM	492	H	TRP	B	52	-32.085	15.006	3.827	1.00	0.00	H

ATOM	493	CA	TRP	B	52	-31.513	12.997	3.196	1.00	0.00	C
ATOM	494	CB	TRP	B	52	-30.688	13.494	1.999	1.00	0.00	C
ATOM	495	CG	TRP	B	52	-30.128	12.322	1.223	1.00	0.00	C
ATOM	496	CD2	TRP	B	52	-30.792	11.522	0.221	1.00	0.00	C
ATOM	497	CE2	TRP	B	52	-29.869	10.502	-0.190	1.00	0.00	C
ATOM	498	CE3	TRP	B	52	-32.081	11.574	-0.349	1.00	0.00	C
ATOM	499	CD1	TRP	B	52	-28.852	11.754	1.354	1.00	0.00	C
ATOM	500	NE1	TRP	B	52	-28.694	10.686	0.528	1.00	0.00	N
ATOM	501	HE1	TRP	B	52	-27.885	10.135	0.436	1.00	0.00	H
ATOM	502	CZ2	TRP	B	52	-30.255	9.550	-1.157	1.00	0.00	C
ATOM	503	CZ3	TRP	B	52	-32.456	10.616	-1.314	1.00	0.00	C
ATOM	504	CH2	TRP	B	52	-31.550	9.608	-1.713	1.00	0.00	C
ATOM	505	C	TRP	B	52	-30.643	12.427	4.308	1.00	0.00	C
ATOM	506	O	TRP	B	52	-30.509	11.225	4.517	1.00	0.00	O
ATOM	507	N	PHE	B	53	-30.057	13.382	5.031	1.00	0.00	N
ATOM	508	H	PHE	B	53	-30.267	14.346	4.886	1.00	0.00	H
ATOM	509	CA	PHE	B	53	-29.240	12.983	6.167	1.00	0.00	C
ATOM	510	CB	PHE	B	53	-27.852	13.603	6.047	1.00	0.00	C
ATOM	511	CG	PHE	B	53	-27.003	12.891	5.023	1.00	0.00	C
ATOM	512	CD1	PHE	B	53	-26.780	13.493	3.767	1.00	0.00	C
ATOM	513	CD2	PHE	B	53	-26.426	11.644	5.349	1.00	0.00	C
ATOM	514	CE1	PHE	B	53	-25.957	12.845	2.826	1.00	0.00	C
ATOM	515	CE2	PHE	B	53	-25.603	10.994	4.410	1.00	0.00	C
ATOM	516	CZ	PHE	B	53	-25.378	11.603	3.160	1.00	0.00	C
ATOM	517	C	PHE	B	53	-29.895	13.402	7.465	1.00	0.00	C
ATOM	518	O	PHE	B	53	-30.947	14.035	7.458	1.00	0.00	O
ATOM	519	N	THR	B	54	-29.225	13.039	8.568	1.00	0.00	N
ATOM	520	H	THR	B	54	-28.399	12.478	8.508	1.00	0.00	H
ATOM	521	CA	THR	B	54	-29.670	13.537	9.860	1.00	0.00	C
ATOM	522	CB	THR	B	54	-29.075	12.687	10.999	1.00	0.00	C
ATOM	523	OG1	THR	B	54	-27.646	12.591	10.915	1.00	0.00	O
ATOM	524	HG1	THR	B	54	-27.358	12.294	11.781	1.00	0.00	H
ATOM	525	CG2	THR	B	54	-29.697	11.290	11.023	1.00	0.00	C
ATOM	526	C	THR	B	54	-29.389	15.022	10.029	1.00	0.00	C
ATOM	527	O	THR	B	54	-28.875	15.694	9.142	1.00	0.00	O
ATOM	528	N	ALA	B	55	-29.795	15.515	11.205	1.00	0.00	N
ATOM	529	H	ALA	B	55	-30.074	14.909	11.944	1.00	0.00	H
ATOM	530	CA	ALA	B	55	-29.640	16.946	11.414	1.00	0.00	C
ATOM	531	CB	ALA	B	55	-30.627	17.418	12.479	1.00	0.00	C
ATOM	532	C	ALA	B	55	-28.235	17.334	11.829	1.00	0.00	C
ATOM	533	O	ALA	B	55	-27.425	16.505	12.232	1.00	0.00	O
ATOM	534	N	LEU	B	56	-28.000	18.646	11.744	1.00	0.00	N
ATOM	535	H	LEU	B	56	-28.706	19.231	11.344	1.00	0.00	H
ATOM	536	CA	LEU	B	56	-26.786	19.168	12.372	1.00	0.00	C
ATOM	537	CB	LEU	B	56	-26.376	20.487	11.709	1.00	0.00	C
ATOM	538	CG	LEU	B	56	-26.318	20.414	10.177	1.00	0.00	C
ATOM	539	CD1	LEU	B	56	-25.894	21.740	9.547	1.00	0.00	C
ATOM	540	CD2	LEU	B	56	-25.441	19.268	9.682	1.00	0.00	C
ATOM	541	C	LEU	B	56	-27.008	19.352	13.864	1.00	0.00	C
ATOM	542	O	LEU	B	56	-28.150	19.362	14.317	1.00	0.00	O
ATOM	543	N	THR	B	57	-25.900	19.494	14.613	1.00	0.00	N
ATOM	544	H	THR	B	57	-24.969	19.453	14.243	1.00	0.00	H
ATOM	545	CA	THR	B	57	-26.109	19.747	16.037	1.00	0.00	C
ATOM	546	CB	THR	B	57	-24.795	19.701	16.827	1.00	0.00	C
ATOM	547	OG1	THR	B	57	-23.732	20.378	16.132	1.00	0.00	O
ATOM	548	HG1	THR	B	57	-23.049	20.585	16.775	1.00	0.00	H
ATOM	549	CG2	THR	B	57	-24.422	18.266	17.185	1.00	0.00	C
ATOM	550	C	THR	B	57	-26.812	21.051	16.338	1.00	0.00	C
ATOM	551	O	THR	B	57	-26.621	22.063	15.675	1.00	0.00	O

ATOM	552	N	GLN	B	58	-27.586	20.983	17.430	1.00	0.00	N
ATOM	553	H	GLN	B	58	-27.707	20.090	17.858	1.00	0.00	H
ATOM	554	CA	GLN	B	58	-28.195	22.186	17.999	1.00	0.00	C
ATOM	555	CB	GLN	B	58	-29.313	21.767	18.954	1.00	0.00	C
ATOM	556	CG	GLN	B	58	-30.436	21.020	18.250	1.00	0.00	C
ATOM	557	CD	GLN	B	58	-31.408	20.448	19.258	1.00	0.00	C
ATOM	558	OE1	GLN	B	58	-31.417	19.253	19.537	1.00	0.00	O
ATOM	559	NE2	GLN	B	58	-32.255	21.335	19.779	1.00	0.00	N
ATOM	560	HE21	GLN	B	58	-32.883	21.065	20.506	1.00	0.00	H
ATOM	561	HE22	GLN	B	58	-32.260	22.279	19.441	1.00	0.00	H
ATOM	562	C	GLN	B	58	-27.213	23.082	18.744	1.00	0.00	C
ATOM	563	O	GLN	B	58	-27.458	23.506	19.866	1.00	0.00	O
ATOM	564	N	HIS	B	59	-26.065	23.322	18.106	1.00	0.00	N
ATOM	565	H	HIS	B	59	-25.994	23.164	17.120	1.00	0.00	H
ATOM	566	CA	HIS	B	59	-24.994	23.940	18.880	1.00	0.00	C
ATOM	567	CB	HIS	B	59	-23.627	23.484	18.359	1.00	0.00	C
ATOM	568	CG	HIS	B	59	-23.393	23.903	16.924	1.00	0.00	C
ATOM	569	ND1	HIS	B	59	-23.839	23.222	15.848	1.00	0.00	N
ATOM	570	HD1	HIS	B	59	-24.341	22.377	15.824	1.00	0.00	H
ATOM	571	CD2	HIS	B	59	-22.686	25.025	16.484	1.00	0.00	C
ATOM	572	NE2	HIS	B	59	-22.715	25.008	15.133	1.00	0.00	N
ATOM	573	CE1	HIS	B	59	-23.422	23.904	14.737	1.00	0.00	C
ATOM	574	C	HIS	B	59	-25.099	25.448	19.016	1.00	0.00	C
ATOM	575	O	HIS	B	59	-24.674	26.046	19.994	1.00	0.00	O
ATOM	576	N	GLY	B	60	-25.717	26.037	17.992	1.00	0.00	N
ATOM	577	H	GLY	B	60	-26.045	25.539	17.188	1.00	0.00	H
ATOM	578	CA	GLY	B	60	-25.901	27.478	18.046	1.00	0.00	C
ATOM	579	C	GLY	B	60	-26.856	27.874	16.954	1.00	0.00	C
ATOM	580	O	GLY	B	60	-27.243	27.058	16.127	1.00	0.00	O
ATOM	581	N	LYS	B	61	-27.231	29.159	16.972	1.00	0.00	N
ATOM	582	H	LYS	B	61	-26.887	29.797	17.658	1.00	0.00	H
ATOM	583	CA	LYS	B	61	-28.168	29.507	15.910	1.00	0.00	C
ATOM	584	CB	LYS	B	61	-29.234	30.517	16.369	1.00	0.00	C
ATOM	585	CG	LYS	B	61	-29.981	30.107	17.648	1.00	0.00	C
ATOM	586	CD	LYS	B	61	-31.516	30.152	17.544	1.00	0.00	C
ATOM	587	CE	LYS	B	61	-32.183	29.902	18.906	1.00	0.00	C
ATOM	588	NZ	LYS	B	61	-33.626	29.650	18.780	1.00	0.00	N
ATOM	589	HZ1	LYS	B	61	-34.109	29.801	19.690	1.00	0.00	H
ATOM	590	HZ2	LYS	B	61	-34.136	30.269	18.112	1.00	0.00	H
ATOM	591	HZ3	LYS	B	61	-33.870	28.673	18.510	1.00	0.00	H
ATOM	592	C	LYS	B	61	-27.551	29.914	14.581	1.00	0.00	C
ATOM	593	O	LYS	B	61	-28.210	29.800	13.557	1.00	0.00	O
ATOM	594	N	GLU	B	62	-26.291	30.401	14.629	1.00	0.00	N
ATOM	595	H	GLU	B	62	-25.805	30.484	15.495	1.00	0.00	H
ATOM	596	CA	GLU	B	62	-25.781	30.979	13.378	1.00	0.00	C
ATOM	597	CB	GLU	B	62	-25.885	32.511	13.375	1.00	0.00	C
ATOM	598	CG	GLU	B	62	-27.313	33.075	13.277	1.00	0.00	C
ATOM	599	CD	GLU	B	62	-27.949	32.872	11.904	1.00	0.00	C
ATOM	600	OE1	GLU	B	62	-28.754	33.696	11.493	1.00	0.00	O
ATOM	601	OE2	GLU	B	62	-27.672	31.908	11.198	1.00	0.00	O
ATOM	602	C	GLU	B	62	-24.414	30.576	12.841	1.00	0.00	C
ATOM	603	O	GLU	B	62	-24.090	30.875	11.695	1.00	0.00	O
ATOM	604	N	ASP	B	63	-23.598	29.889	13.665	1.00	0.00	N
ATOM	605	H	ASP	B	63	-23.888	29.543	14.553	1.00	0.00	H
ATOM	606	CA	ASP	B	63	-22.388	29.381	13.004	1.00	0.00	C
ATOM	607	CB	ASP	B	63	-21.185	29.180	13.953	1.00	0.00	C
ATOM	608	CG	ASP	B	63	-19.855	29.093	13.184	1.00	0.00	C
ATOM	609	OD1	ASP	B	63	-19.846	28.954	11.964	1.00	0.00	O
ATOM	610	OD2	ASP	B	63	-18.797	29.251	13.780	1.00	0.00	O

ATOM	611	C	ASP	B	63	-22.697	28.108	12.248	1.00	0.00	C
ATOM	612	O	ASP	B	63	-22.548	27.010	12.754	1.00	0.00	O
ATOM	613	N	LEU	B	64	-23.205	28.315	11.026	1.00	0.00	N
ATOM	614	H	LEU	B	64	-23.352	29.264	10.749	1.00	0.00	H
ATOM	615	CA	LEU	B	64	-23.694	27.150	10.292	1.00	0.00	C
ATOM	616	CB	LEU	B	64	-25.174	26.888	10.598	1.00	0.00	C
ATOM	617	CG	LEU	B	64	-25.342	26.183	11.947	1.00	0.00	C
ATOM	618	CD1	LEU	B	64	-26.221	26.960	12.922	1.00	0.00	C
ATOM	619	CD2	LEU	B	64	-25.725	24.716	11.791	1.00	0.00	C
ATOM	620	C	LEU	B	64	-23.446	27.172	8.801	1.00	0.00	C
ATOM	621	O	LEU	B	64	-23.132	26.154	8.202	1.00	0.00	O
ATOM	622	N	LYS	B	65	-23.591	28.370	8.200	1.00	0.00	N
ATOM	623	H	LYS	B	65	-23.802	29.182	8.736	1.00	0.00	H
ATOM	624	CA	LYS	B	65	-23.301	28.390	6.761	1.00	0.00	C
ATOM	625	CB	LYS	B	65	-23.711	29.711	6.095	1.00	0.00	C
ATOM	626	CG	LYS	B	65	-25.213	30.035	6.127	1.00	0.00	C
ATOM	627	CD	LYS	B	65	-25.748	30.451	4.747	1.00	0.00	C
ATOM	628	CE	LYS	B	65	-27.189	30.975	4.738	1.00	0.00	C
ATOM	629	NZ	LYS	B	65	-27.651	31.210	3.359	1.00	0.00	N
ATOM	630	HZ1	LYS	B	65	-27.846	30.294	2.905	1.00	0.00	H
ATOM	631	HZ2	LYS	B	65	-28.544	31.746	3.336	1.00	0.00	H
ATOM	632	HZ3	LYS	B	65	-26.941	31.716	2.794	1.00	0.00	H
ATOM	633	C	LYS	B	65	-21.856	28.023	6.443	1.00	0.00	C
ATOM	634	O	LYS	B	65	-20.934	28.326	7.197	1.00	0.00	O
ATOM	635	N	PHE	B	66	-21.735	27.289	5.331	1.00	0.00	N
ATOM	636	H	PHE	B	66	-22.476	27.283	4.660	1.00	0.00	H
ATOM	637	CA	PHE	B	66	-20.488	26.575	5.073	1.00	0.00	C
ATOM	638	CB	PHE	B	66	-20.768	25.085	4.826	1.00	0.00	C
ATOM	639	CG	PHE	B	66	-21.288	24.380	6.060	1.00	0.00	C
ATOM	640	CD1	PHE	B	66	-22.531	23.714	5.990	1.00	0.00	C
ATOM	641	CD2	PHE	B	66	-20.524	24.376	7.250	1.00	0.00	C
ATOM	642	CE1	PHE	B	66	-23.016	23.028	7.120	1.00	0.00	C
ATOM	643	CE2	PHE	B	66	-21.008	23.689	8.380	1.00	0.00	C
ATOM	644	CZ	PHE	B	66	-22.248	23.021	8.303	1.00	0.00	C
ATOM	645	C	PHE	B	66	-19.769	27.147	3.870	1.00	0.00	C
ATOM	646	O	PHE	B	66	-20.388	27.690	2.960	1.00	0.00	O
ATOM	647	N	PRO	B	67	-18.422	27.033	3.877	1.00	0.00	N
ATOM	648	CD	PRO	B	67	-17.545	26.722	4.997	1.00	0.00	C
ATOM	649	CA	PRO	B	67	-17.691	27.259	2.626	1.00	0.00	C
ATOM	650	CB	PRO	B	67	-16.227	27.122	3.054	1.00	0.00	C
ATOM	651	CG	PRO	B	67	-16.227	27.347	4.566	1.00	0.00	C
ATOM	652	C	PRO	B	67	-18.092	26.262	1.555	1.00	0.00	C
ATOM	653	O	PRO	B	67	-18.477	25.139	1.847	1.00	0.00	O
ATOM	654	N	ARG	B	68	-18.010	26.741	0.308	1.00	0.00	N
ATOM	655	H	ARG	B	68	-17.570	27.620	0.155	1.00	0.00	H
ATOM	656	CA	ARG	B	68	-18.364	25.865	-0.806	1.00	0.00	C
ATOM	657	CB	ARG	B	68	-18.615	26.749	-2.035	1.00	0.00	C
ATOM	658	CG	ARG	B	68	-19.236	25.996	-3.204	1.00	0.00	C
ATOM	659	CD	ARG	B	68	-19.483	26.813	-4.465	1.00	0.00	C
ATOM	660	NE	ARG	B	68	-19.803	25.890	-5.548	1.00	0.00	N
ATOM	661	HE	ARG	B	68	-20.520	25.210	-5.378	1.00	0.00	H
ATOM	662	CZ	ARG	B	68	-19.031	25.857	-6.653	1.00	0.00	C
ATOM	663	NH1	ARG	B	68	-18.078	26.758	-6.852	1.00	0.00	N
ATOM	664	HH11	ARG	B	68	-17.901	27.517	-6.234	1.00	0.00	H
ATOM	665	HH12	ARG	B	68	-17.459	26.660	-7.653	1.00	0.00	H
ATOM	666	NH2	ARG	B	68	-19.201	24.902	-7.551	1.00	0.00	N
ATOM	667	HH21	ARG	B	68	-19.778	24.096	-7.386	1.00	0.00	H
ATOM	668	HH22	ARG	B	68	-18.676	24.955	-8.410	1.00	0.00	H
ATOM	669	C	ARG	B	68	-17.282	24.811	-1.029	1.00	0.00	C

ATOM	670	O	ARG	B	68	-16.133	25.021	-0.658	1.00	0.00	O
ATOM	671	N	GLY	B	69	-17.686	23.670	-1.607	1.00	0.00	N
ATOM	672	H	GLY	B	69	-18.646	23.533	-1.851	1.00	0.00	H
ATOM	673	CA	GLY	B	69	-16.757	22.546	-1.740	1.00	0.00	C
ATOM	674	C	GLY	B	69	-16.751	21.710	-0.478	1.00	0.00	C
ATOM	675	O	GLY	B	69	-17.150	20.552	-0.411	1.00	0.00	O
ATOM	676	N	GLN	B	70	-16.332	22.422	0.572	1.00	0.00	N
ATOM	677	H	GLN	B	70	-16.006	23.348	0.388	1.00	0.00	H
ATOM	678	CA	GLN	B	70	-16.624	21.970	1.926	1.00	0.00	C
ATOM	679	CB	GLN	B	70	-15.982	22.958	2.897	1.00	0.00	C
ATOM	680	CG	GLN	B	70	-14.495	23.225	2.630	1.00	0.00	C
ATOM	681	CD	GLN	B	70	-14.006	24.300	3.580	1.00	0.00	C
ATOM	682	OE1	GLN	B	70	-14.594	24.560	4.622	1.00	0.00	O
ATOM	683	NE2	GLN	B	70	-12.896	24.927	3.195	1.00	0.00	N
ATOM	684	HE21	GLN	B	70	-12.440	25.576	3.800	1.00	0.00	H
ATOM	685	HE22	GLN	B	70	-12.528	24.773	2.275	1.00	0.00	H
ATOM	686	C	GLN	B	70	-18.130	21.864	2.167	1.00	0.00	C
ATOM	687	O	GLN	B	70	-18.949	22.270	1.351	1.00	0.00	O
ATOM	688	N	GLY	B	71	-18.477	21.293	3.327	1.00	0.00	N
ATOM	689	H	GLY	B	71	-17.784	20.890	3.928	1.00	0.00	H
ATOM	690	CA	GLY	B	71	-19.885	21.382	3.721	1.00	0.00	C
ATOM	691	C	GLY	B	71	-20.873	20.416	3.082	1.00	0.00	C
ATOM	692	O	GLY	B	71	-21.953	20.184	3.616	1.00	0.00	O
ATOM	693	N	VAL	B	72	-20.481	19.852	1.932	1.00	0.00	N
ATOM	694	H	VAL	B	72	-19.597	20.087	1.529	1.00	0.00	H
ATOM	695	CA	VAL	B	72	-21.390	18.906	1.280	1.00	0.00	C
ATOM	696	CB	VAL	B	72	-21.057	18.774	-0.211	1.00	0.00	C
ATOM	697	CG1	VAL	B	72	-22.169	18.032	-0.955	1.00	0.00	C
ATOM	698	CG2	VAL	B	72	-20.742	20.124	-0.849	1.00	0.00	C
ATOM	699	C	VAL	B	72	-21.351	17.535	1.933	1.00	0.00	C
ATOM	700	O	VAL	B	72	-20.325	16.868	1.941	1.00	0.00	O
ATOM	701	N	PRO	B	73	-22.501	17.108	2.498	1.00	0.00	N
ATOM	702	CD	PRO	B	73	-23.804	17.761	2.521	1.00	0.00	C
ATOM	703	CA	PRO	B	73	-22.490	15.811	3.171	1.00	0.00	C
ATOM	704	CB	PRO	B	73	-23.819	15.822	3.926	1.00	0.00	C
ATOM	705	CG	PRO	B	73	-24.739	16.696	3.080	1.00	0.00	C
ATOM	706	C	PRO	B	73	-22.349	14.680	2.173	1.00	0.00	C
ATOM	707	O	PRO	B	73	-22.913	14.680	1.085	1.00	0.00	O
ATOM	708	N	ILE	B	74	-21.541	13.704	2.595	1.00	0.00	N
ATOM	709	H	ILE	B	74	-21.124	13.784	3.503	1.00	0.00	H
ATOM	710	CA	ILE	B	74	-21.226	12.631	1.655	1.00	0.00	C
ATOM	711	CB	ILE	B	74	-19.993	11.856	2.153	1.00	0.00	C
ATOM	712	CG2	ILE	B	74	-19.548	10.778	1.158	1.00	0.00	C
ATOM	713	CG1	ILE	B	74	-18.850	12.811	2.515	1.00	0.00	C
ATOM	714	CD1	ILE	B	74	-17.736	12.134	3.315	1.00	0.00	C
ATOM	715	C	ILE	B	74	-22.395	11.689	1.387	1.00	0.00	C
ATOM	716	O	ILE	B	74	-22.558	10.684	2.074	1.00	0.00	O
ATOM	717	N	ASN	B	75	-23.187	12.033	0.360	1.00	0.00	N
ATOM	718	H	ASN	B	75	-23.035	12.920	-0.083	1.00	0.00	H
ATOM	719	CA	ASN	B	75	-24.123	11.033	-0.161	1.00	0.00	C
ATOM	720	CB	ASN	B	75	-25.318	11.686	-0.877	1.00	0.00	C
ATOM	721	CG	ASN	B	75	-26.270	10.628	-1.429	1.00	0.00	C
ATOM	722	OD1	ASN	B	75	-26.456	9.557	-0.853	1.00	0.00	O
ATOM	723	ND2	ASN	B	75	-26.822	10.951	-2.598	1.00	0.00	N
ATOM	724	HD21	ASN	B	75	-27.406	10.347	-3.142	1.00	0.00	H
ATOM	725	HD22	ASN	B	75	-26.632	11.857	-2.989	1.00	0.00	H
ATOM	726	C	ASN	B	75	-23.398	10.078	-1.085	1.00	0.00	C
ATOM	727	O	ASN	B	75	-22.541	10.472	-1.864	1.00	0.00	O
ATOM	728	N	THR	B	76	-23.760	8.800	-0.942	1.00	0.00	N

ATOM	729	H	THR	B	76	-24.546	8.547	-0.377	1.00	0.00	H
ATOM	730	CA	THR	B	76	-23.047	7.823	-1.752	1.00	0.00	C
ATOM	731	CB	THR	B	76	-22.426	6.742	-0.856	1.00	0.00	C
ATOM	732	OG1	THR	B	76	-23.405	5.815	-0.354	1.00	0.00	O
ATOM	733	HG1	THR	B	76	-23.793	5.443	-1.148	1.00	0.00	H
ATOM	734	CG2	THR	B	76	-21.633	7.382	0.287	1.00	0.00	C
ATOM	735	C	THR	B	76	-23.913	7.211	-2.831	1.00	0.00	C
ATOM	736	O	THR	B	76	-23.811	6.032	-3.137	1.00	0.00	O
ATOM	737	N	ASN	B	77	-24.818	8.051	-3.336	1.00	0.00	N
ATOM	738	H	ASN	B	77	-24.776	9.031	-3.125	1.00	0.00	H
ATOM	739	CA	ASN	B	77	-25.795	7.594	-4.321	1.00	0.00	C
ATOM	740	CB	ASN	B	77	-27.154	7.234	-3.694	1.00	0.00	C
ATOM	741	CG	ASN	B	77	-27.065	6.143	-2.644	1.00	0.00	C
ATOM	742	OD1	ASN	B	77	-27.028	4.947	-2.909	1.00	0.00	O
ATOM	743	ND2	ASN	B	77	-27.093	6.619	-1.394	1.00	0.00	N
ATOM	744	HD21	ASN	B	77	-27.149	5.962	-0.642	1.00	0.00	H
ATOM	745	HD22	ASN	B	77	-27.070	7.607	-1.221	1.00	0.00	H
ATOM	746	C	ASN	B	77	-25.992	8.765	-5.252	1.00	0.00	C
ATOM	747	O	ASN	B	77	-27.055	9.369	-5.321	1.00	0.00	O
ATOM	748	N	SER	B	78	-24.863	9.114	-5.881	1.00	0.00	N
ATOM	749	H	SER	B	78	-24.028	8.574	-5.799	1.00	0.00	H
ATOM	750	CA	SER	B	78	-24.759	10.465	-6.422	1.00	0.00	C
ATOM	751	CB	SER	B	78	-24.427	11.441	-5.276	1.00	0.00	C
ATOM	752	OG	SER	B	78	-24.172	10.722	-4.048	1.00	0.00	O
ATOM	753	HG	SER	B	78	-23.367	11.080	-3.678	1.00	0.00	H
ATOM	754	C	SER	B	78	-23.698	10.526	-7.505	1.00	0.00	C
ATOM	755	O	SER	B	78	-22.723	9.779	-7.471	1.00	0.00	O
ATOM	756	N	SER	B	79	-23.921	11.433	-8.465	1.00	0.00	N
ATOM	757	H	SER	B	79	-24.755	11.993	-8.485	1.00	0.00	H
ATOM	758	CA	SER	B	79	-22.853	11.720	-9.423	1.00	0.00	C
ATOM	759	CB	SER	B	79	-23.534	12.257	-10.692	1.00	0.00	C
ATOM	760	OG	SER	B	79	-24.068	13.555	-10.429	1.00	0.00	O
ATOM	761	HG	SER	B	79	-25.023	13.460	-10.367	1.00	0.00	H
ATOM	762	C	SER	B	79	-21.862	12.710	-8.797	1.00	0.00	C
ATOM	763	O	SER	B	79	-22.083	13.151	-7.669	1.00	0.00	O
ATOM	764	N	PRO	B	80	-20.781	13.110	-9.528	1.00	0.00	N
ATOM	765	CD	PRO	B	80	-20.198	12.555	-10.751	1.00	0.00	C
ATOM	766	CA	PRO	B	80	-20.072	14.309	-9.062	1.00	0.00	C
ATOM	767	CB	PRO	B	80	-18.902	14.435	-10.048	1.00	0.00	C
ATOM	768	CG	PRO	B	80	-19.315	13.666	-11.302	1.00	0.00	C
ATOM	769	C	PRO	B	80	-20.990	15.525	-8.982	1.00	0.00	C
ATOM	770	O	PRO	B	80	-21.077	16.231	-7.983	1.00	0.00	O
ATOM	771	N	ASP	B	81	-21.742	15.677	-10.073	1.00	0.00	N
ATOM	772	H	ASP	B	81	-21.589	15.139	-10.906	1.00	0.00	H
ATOM	773	CA	ASP	B	81	-22.712	16.759	-10.205	1.00	0.00	C
ATOM	774	CB	ASP	B	81	-23.260	16.761	-11.631	1.00	0.00	C
ATOM	775	CG	ASP	B	81	-22.085	16.780	-12.589	1.00	0.00	C
ATOM	776	OD1	ASP	B	81	-21.686	15.709	-13.040	1.00	0.00	O
ATOM	777	OD2	ASP	B	81	-21.539	17.852	-12.831	1.00	0.00	O
ATOM	778	C	ASP	B	81	-23.836	16.769	-9.190	1.00	0.00	C
ATOM	779	O	ASP	B	81	-24.521	17.769	-8.988	1.00	0.00	O
ATOM	780	N	ASP	B	82	-23.997	15.620	-8.525	1.00	0.00	N
ATOM	781	H	ASP	B	82	-23.515	14.797	-8.828	1.00	0.00	H
ATOM	782	CA	ASP	B	82	-24.998	15.587	-7.467	1.00	0.00	C
ATOM	783	CB	ASP	B	82	-25.838	14.313	-7.497	1.00	0.00	C
ATOM	784	CG	ASP	B	82	-26.502	14.123	-8.844	1.00	0.00	C
ATOM	785	OD1	ASP	B	82	-26.941	15.100	-9.452	1.00	0.00	O
ATOM	786	OD2	ASP	B	82	-26.539	12.986	-9.302	1.00	0.00	O
ATOM	787	C	ASP	B	82	-24.472	15.809	-6.072	1.00	0.00	C

ATOM	788	O	ASP	B	82	-25.106	15.474	-5.078	1.00	0.00	O
ATOM	789	N	GLN	B	83	-23.301	16.443	-6.004	1.00	0.00	N
ATOM	790	H	GLN	B	83	-22.753	16.644	-6.820	1.00	0.00	H
ATOM	791	CA	GLN	B	83	-22.907	16.928	-4.685	1.00	0.00	C
ATOM	792	CB	GLN	B	83	-21.393	16.912	-4.581	1.00	0.00	C
ATOM	793	CG	GLN	B	83	-20.895	15.476	-4.580	1.00	0.00	C
ATOM	794	CD	GLN	B	83	-19.476	15.436	-5.084	1.00	0.00	C
ATOM	795	OE1	GLN	B	83	-18.581	16.151	-4.653	1.00	0.00	O
ATOM	796	NE2	GLN	B	83	-19.311	14.511	-6.021	1.00	0.00	N
ATOM	797	HE21	GLN	B	83	-18.400	14.259	-6.340	1.00	0.00	H
ATOM	798	HE22	GLN	B	83	-20.113	14.054	-6.406	1.00	0.00	H
ATOM	799	C	GLN	B	83	-23.475	18.299	-4.377	1.00	0.00	C
ATOM	800	O	GLN	B	83	-22.769	19.232	-4.022	1.00	0.00	O
ATOM	801	N	ILE	B	84	-24.800	18.386	-4.574	1.00	0.00	N
ATOM	802	H	ILE	B	84	-25.338	17.566	-4.769	1.00	0.00	H
ATOM	803	CA	ILE	B	84	-25.469	19.665	-4.374	1.00	0.00	C
ATOM	804	CB	ILE	B	84	-25.785	20.383	-5.694	1.00	0.00	C
ATOM	805	CG2	ILE	B	84	-26.348	21.783	-5.430	1.00	0.00	C
ATOM	806	CG1	ILE	B	84	-24.582	20.434	-6.631	1.00	0.00	C
ATOM	807	CD1	ILE	B	84	-24.819	21.363	-7.811	1.00	0.00	C
ATOM	808	C	ILE	B	84	-26.751	19.464	-3.609	1.00	0.00	C
ATOM	809	O	ILE	B	84	-27.627	18.696	-3.996	1.00	0.00	O
ATOM	810	N	GLY	B	85	-26.827	20.203	-2.511	1.00	0.00	N
ATOM	811	H	GLY	B	85	-26.104	20.857	-2.267	1.00	0.00	H
ATOM	812	CA	GLY	B	85	-28.055	20.140	-1.747	1.00	0.00	C
ATOM	813	C	GLY	B	85	-28.158	21.363	-0.888	1.00	0.00	C
ATOM	814	O	GLY	B	85	-27.354	22.285	-0.982	1.00	0.00	O
ATOM	815	N	TYR	B	86	-29.176	21.329	-0.036	1.00	0.00	N
ATOM	816	H	TYR	B	86	-29.798	20.548	0.036	1.00	0.00	H
ATOM	817	CA	TYR	B	86	-29.267	22.454	0.869	1.00	0.00	C
ATOM	818	CB	TYR	B	86	-30.224	23.525	0.324	1.00	0.00	C
ATOM	819	CG	TYR	B	86	-31.669	23.086	0.223	1.00	0.00	C
ATOM	820	CD1	TYR	B	86	-32.530	23.316	1.317	1.00	0.00	C
ATOM	821	CE1	TYR	B	86	-33.880	22.947	1.212	1.00	0.00	C
ATOM	822	CD2	TYR	B	86	-32.125	22.488	-0.969	1.00	0.00	C
ATOM	823	CE2	TYR	B	86	-33.477	22.124	-1.073	1.00	0.00	C
ATOM	824	CZ	TYR	B	86	-34.338	22.354	0.019	1.00	0.00	C
ATOM	825	OH	TYR	B	86	-35.667	21.985	-0.080	1.00	0.00	O
ATOM	826	HH	TYR	B	86	-35.751	21.521	-0.915	1.00	0.00	H
ATOM	827	C	TYR	B	86	-29.584	22.027	2.276	1.00	0.00	C
ATOM	828	O	TYR	B	86	-30.408	21.154	2.520	1.00	0.00	O
ATOM	829	N	TYR	B	87	-28.880	22.683	3.201	1.00	0.00	N
ATOM	830	H	TYR	B	87	-28.244	23.395	2.902	1.00	0.00	H
ATOM	831	CA	TYR	B	87	-29.378	22.574	4.566	1.00	0.00	C
ATOM	832	CB	TYR	B	87	-28.286	22.832	5.601	1.00	0.00	C
ATOM	833	CG	TYR	B	87	-27.154	21.845	5.480	1.00	0.00	C
ATOM	834	CD1	TYR	B	87	-26.025	22.188	4.708	1.00	0.00	C
ATOM	835	CE1	TYR	B	87	-24.947	21.292	4.645	1.00	0.00	C
ATOM	836	CD2	TYR	B	87	-27.251	20.617	6.162	1.00	0.00	C
ATOM	837	CE2	TYR	B	87	-26.172	19.724	6.098	1.00	0.00	C
ATOM	838	CZ	TYR	B	87	-25.030	20.078	5.355	1.00	0.00	C
ATOM	839	OH	TYR	B	87	-23.964	19.208	5.330	1.00	0.00	O
ATOM	840	HH	TYR	B	87	-23.268	19.581	4.789	1.00	0.00	H
ATOM	841	C	TYR	B	87	-30.457	23.608	4.752	1.00	0.00	C
ATOM	842	O	TYR	B	87	-30.463	24.626	4.071	1.00	0.00	O
ATOM	843	N	ARG	B	88	-31.347	23.320	5.697	1.00	0.00	N
ATOM	844	H	ARG	B	88	-31.348	22.416	6.126	1.00	0.00	H
ATOM	845	CA	ARG	B	88	-32.403	24.288	5.929	1.00	0.00	C
ATOM	846	CB	ARG	B	88	-33.580	23.969	5.010	1.00	0.00	C

ATOM	847	CG	ARG	B	88	-34.654	25.052	4.947	1.00	0.00	C
ATOM	848	CD	ARG	B	88	-36.019	24.455	4.577	1.00	0.00	C
ATOM	849	NE	ARG	B	88	-36.330	23.296	5.422	1.00	0.00	N
ATOM	850	HE	ARG	B	88	-36.207	22.389	5.017	1.00	0.00	H
ATOM	851	CZ	ARG	B	88	-36.560	23.429	6.743	1.00	0.00	C
ATOM	852	NH1	ARG	B	88	-36.800	24.632	7.257	1.00	0.00	N
ATOM	853	HH11	ARG	B	88	-36.976	25.404	6.645	1.00	0.00	H
ATOM	854	HH12	ARG	B	88	-36.772	24.821	8.244	1.00	0.00	H
ATOM	855	NH2	ARG	B	88	-36.514	22.348	7.513	1.00	0.00	N
ATOM	856	HH21	ARG	B	88	-36.332	21.448	7.093	1.00	0.00	H
ATOM	857	HH22	ARG	B	88	-36.645	22.396	8.500	1.00	0.00	H
ATOM	858	C	ARG	B	88	-32.799	24.323	7.392	1.00	0.00	C
ATOM	859	O	ARG	B	88	-33.137	23.314	8.005	1.00	0.00	O
ATOM	860	N	ARG	B	89	-32.723	25.550	7.924	1.00	0.00	N
ATOM	861	H	ARG	B	89	-32.353	26.262	7.326	1.00	0.00	H
ATOM	862	CA	ARG	B	89	-33.003	25.806	9.338	1.00	0.00	C
ATOM	863	CB	ARG	B	89	-32.897	27.313	9.609	1.00	0.00	C
ATOM	864	CG	ARG	B	89	-32.710	27.684	11.084	1.00	0.00	C
ATOM	865	CD	ARG	B	89	-32.969	29.162	11.412	1.00	0.00	C
ATOM	866	NE	ARG	B	89	-32.145	30.099	10.641	1.00	0.00	N
ATOM	867	HE	ARG	B	89	-32.500	30.433	9.767	1.00	0.00	H
ATOM	868	CZ	ARG	B	89	-30.971	30.578	11.119	1.00	0.00	C
ATOM	869	NH1	ARG	B	89	-30.453	30.118	12.248	1.00	0.00	N
ATOM	870	HH11	ARG	B	89	-30.932	29.450	12.809	1.00	0.00	H
ATOM	871	HH12	ARG	B	89	-29.537	30.421	12.552	1.00	0.00	H
ATOM	872	NH2	ARG	B	89	-30.320	31.520	10.460	1.00	0.00	N
ATOM	873	HH21	ARG	B	89	-30.610	31.901	9.577	1.00	0.00	H
ATOM	874	HH22	ARG	B	89	-29.473	31.913	10.848	1.00	0.00	H
ATOM	875	C	ARG	B	89	-34.356	25.305	9.827	1.00	0.00	C
ATOM	876	O	ARG	B	89	-35.399	25.579	9.242	1.00	0.00	O
ATOM	877	N	ALA	B	90	-34.311	24.601	10.961	1.00	0.00	N
ATOM	878	H	ALA	B	90	-33.467	24.183	11.305	1.00	0.00	H
ATOM	879	CA	ALA	B	90	-35.564	24.442	11.685	1.00	0.00	C
ATOM	880	CB	ALA	B	90	-36.170	23.057	11.453	1.00	0.00	C
ATOM	881	C	ALA	B	90	-35.357	24.666	13.165	1.00	0.00	C
ATOM	882	O	ALA	B	90	-34.830	23.822	13.883	1.00	0.00	O
ATOM	883	N	THR	B	91	-35.786	25.856	13.593	1.00	0.00	N
ATOM	884	H	THR	B	91	-36.286	26.501	13.013	1.00	0.00	H
ATOM	885	CA	THR	B	91	-35.693	26.194	15.011	1.00	0.00	C
ATOM	886	CB	THR	B	91	-36.007	27.683	15.149	1.00	0.00	C
ATOM	887	OG1	THR	B	91	-36.960	28.074	14.152	1.00	0.00	O
ATOM	888	HG1	THR	B	91	-37.257	28.938	14.424	1.00	0.00	H
ATOM	889	CG2	THR	B	91	-34.739	28.529	15.013	1.00	0.00	C
ATOM	890	C	THR	B	91	-36.575	25.313	15.886	1.00	0.00	C
ATOM	891	O	THR	B	91	-37.106	24.299	15.434	1.00	0.00	O
ATOM	892	N	ARG	B	92	-36.693	25.698	17.162	1.00	0.00	N
ATOM	893	H	ARG	B	92	-36.299	26.548	17.530	1.00	0.00	H
ATOM	894	CA	ARG	B	92	-37.590	24.896	17.984	1.00	0.00	C
ATOM	895	CB	ARG	B	92	-36.828	24.094	19.043	1.00	0.00	C
ATOM	896	CG	ARG	B	92	-36.686	22.607	18.708	1.00	0.00	C
ATOM	897	CD	ARG	B	92	-35.739	22.330	17.542	1.00	0.00	C
ATOM	898	NE	ARG	B	92	-35.871	20.951	17.077	1.00	0.00	N
ATOM	899	HE	ARG	B	92	-35.782	20.230	17.765	1.00	0.00	H
ATOM	900	CZ	ARG	B	92	-36.059	20.697	15.763	1.00	0.00	C
ATOM	901	NH1	ARG	B	92	-36.209	21.687	14.887	1.00	0.00	N
ATOM	902	HH11	ARG	B	92	-36.258	22.653	15.177	1.00	0.00	H
ATOM	903	HH12	ARG	B	92	-36.280	21.519	13.906	1.00	0.00	H
ATOM	904	NH2	ARG	B	92	-36.086	19.432	15.353	1.00	0.00	N
ATOM	905	HH21	ARG	B	92	-36.054	18.682	16.011	1.00	0.00	H

ATOM	906	HH22	ARG	B	92	-36.127	19.179	14.377	1.00	0.00	H
ATOM	907	C	ARG	B	92	-38.629	25.772	18.632	1.00	0.00	C
ATOM	908	O	ARG	B	92	-38.400	26.349	19.682	1.00	0.00	O
ATOM	909	N	ARG	B	93	-39.782	25.852	17.955	1.00	0.00	N
ATOM	910	H	ARG	B	93	-39.904	25.294	17.136	1.00	0.00	H
ATOM	911	CA	ARG	B	93	-40.839	26.731	18.457	1.00	0.00	C
ATOM	912	CB	ARG	B	93	-41.752	27.139	17.297	1.00	0.00	C
ATOM	913	CG	ARG	B	93	-42.583	28.397	17.565	1.00	0.00	C
ATOM	914	CD	ARG	B	93	-43.797	28.493	16.640	1.00	0.00	C
ATOM	915	NE	ARG	B	93	-44.765	27.438	16.950	1.00	0.00	N
ATOM	916	HE	ARG	B	93	-44.625	26.945	17.815	1.00	0.00	H
ATOM	917	CZ	ARG	B	93	-45.822	27.209	16.138	1.00	0.00	C
ATOM	918	NH1	ARG	B	93	-45.960	27.859	14.987	1.00	0.00	N
ATOM	919	HH11	ARG	B	93	-45.293	28.540	14.685	1.00	0.00	H
ATOM	920	HH12	ARG	B	93	-46.752	27.655	14.402	1.00	0.00	H
ATOM	921	NH2	ARG	B	93	-46.751	26.325	16.482	1.00	0.00	N
ATOM	922	HH21	ARG	B	93	-46.694	25.801	17.332	1.00	0.00	H
ATOM	923	HH22	ARG	B	93	-47.534	26.183	15.866	1.00	0.00	H
ATOM	924	C	ARG	B	93	-41.656	26.125	19.594	1.00	0.00	C
ATOM	925	O	ARG	B	93	-42.846	25.848	19.465	1.00	0.00	O
ATOM	926	N	ILE	B	94	-40.952	25.900	20.709	1.00	0.00	N
ATOM	927	H	ILE	B	94	-40.013	26.247	20.792	1.00	0.00	H
ATOM	928	CA	ILE	B	94	-41.618	25.267	21.842	1.00	0.00	C
ATOM	929	CB	ILE	B	94	-40.579	24.451	22.635	1.00	0.00	C
ATOM	930	CG2	ILE	B	94	-41.144	23.752	23.878	1.00	0.00	C
ATOM	931	CG1	ILE	B	94	-39.911	23.445	21.691	1.00	0.00	C
ATOM	932	CD1	ILE	B	94	-38.812	22.615	22.355	1.00	0.00	C
ATOM	933	C	ILE	B	94	-42.317	26.313	22.691	1.00	0.00	C
ATOM	934	O	ILE	B	94	-41.863	27.442	22.819	1.00	0.00	O
ATOM	935	N	ARG	B	95	-43.451	25.902	23.277	1.00	0.00	N
ATOM	936	H	ARG	B	95	-43.767	24.969	23.115	1.00	0.00	H
ATOM	937	CA	ARG	B	95	-44.147	26.814	24.186	1.00	0.00	C
ATOM	938	CB	ARG	B	95	-45.442	26.142	24.645	1.00	0.00	C
ATOM	939	CG	ARG	B	95	-46.525	27.099	25.141	1.00	0.00	C
ATOM	940	CD	ARG	B	95	-47.796	26.347	25.533	1.00	0.00	C
ATOM	941	NE	ARG	B	95	-48.830	27.264	26.005	1.00	0.00	N
ATOM	942	HE	ARG	B	95	-48.854	28.198	25.628	1.00	0.00	H
ATOM	943	CZ	ARG	B	95	-49.754	26.876	26.908	1.00	0.00	C
ATOM	944	NH1	ARG	B	95	-49.718	25.649	27.427	1.00	0.00	N
ATOM	945	HH11	ARG	B	95	-48.988	25.020	27.159	1.00	0.00	H
ATOM	946	HH12	ARG	B	95	-50.400	25.335	28.086	1.00	0.00	H
ATOM	947	NH2	ARG	B	95	-50.702	27.731	27.274	1.00	0.00	N
ATOM	948	HH21	ARG	B	95	-50.695	28.661	26.881	1.00	0.00	H
ATOM	949	HH22	ARG	B	95	-51.425	27.509	27.926	1.00	0.00	H
ATOM	950	C	ARG	B	95	-43.273	27.252	25.359	1.00	0.00	C
ATOM	951	O	ARG	B	95	-42.455	26.482	25.857	1.00	0.00	O
ATOM	952	N	GLY	B	96	-43.447	28.525	25.755	1.00	0.00	N
ATOM	953	H	GLY	B	96	-44.180	29.063	25.339	1.00	0.00	H
ATOM	954	CA	GLY	B	96	-42.637	29.052	26.857	1.00	0.00	C
ATOM	955	C	GLY	B	96	-43.050	28.486	28.203	1.00	0.00	C
ATOM	956	O	GLY	B	96	-42.780	27.333	28.528	1.00	0.00	O
ATOM	957	N	GLY	B	97	-43.759	29.338	28.948	1.00	0.00	N
ATOM	958	H	GLY	B	97	-43.872	30.286	28.642	1.00	0.00	H
ATOM	959	CA	GLY	B	97	-44.742	28.742	29.846	1.00	0.00	C
ATOM	960	C	GLY	B	97	-46.005	28.676	29.021	1.00	0.00	C
ATOM	961	O	GLY	B	97	-46.313	27.694	28.359	1.00	0.00	O
ATOM	962	N	ASP	B	98	-46.624	29.858	28.982	1.00	0.00	N
ATOM	963	H	ASP	B	98	-46.452	30.545	29.686	1.00	0.00	H
ATOM	964	CA	ASP	B	98	-47.095	30.265	27.666	1.00	0.00	C

ATOM	965	CB	ASP	B	98	-48.601	30.564	27.674	1.00	0.00	C
ATOM	966	CG	ASP	B	98	-49.186	30.347	26.281	1.00	0.00	C
ATOM	967	OD1	ASP	B	98	-50.396	30.242	26.150	1.00	0.00	O
ATOM	968	OD2	ASP	B	98	-48.449	30.228	25.308	1.00	0.00	O
ATOM	969	C	ASP	B	98	-46.226	31.425	27.190	1.00	0.00	C
ATOM	970	O	ASP	B	98	-45.061	31.503	27.573	1.00	0.00	O
ATOM	971	N	GLY	B	99	-46.805	32.335	26.392	1.00	0.00	N
ATOM	972	H	GLY	B	99	-47.709	32.143	26.011	1.00	0.00	H
ATOM	973	CA	GLY	B	99	-46.108	33.583	26.091	1.00	0.00	C
ATOM	974	C	GLY	B	99	-44.801	33.419	25.343	1.00	0.00	C
ATOM	975	O	GLY	B	99	-44.754	33.011	24.188	1.00	0.00	O
ATOM	976	N	LYS	B	100	-43.731	33.778	26.065	1.00	0.00	N
ATOM	977	H	LYS	B	100	-43.840	33.937	27.046	1.00	0.00	H
ATOM	978	CA	LYS	B	100	-42.427	33.846	25.413	1.00	0.00	C
ATOM	979	CB	LYS	B	100	-41.476	34.730	26.224	1.00	0.00	C
ATOM	980	CG	LYS	B	100	-40.173	35.048	25.482	1.00	0.00	C
ATOM	981	CD	LYS	B	100	-38.962	34.964	26.414	1.00	0.00	C
ATOM	982	CE	LYS	B	100	-37.888	33.978	25.935	1.00	0.00	C
ATOM	983	NZ	LYS	B	100	-38.455	32.631	25.763	1.00	0.00	N
ATOM	984	HZ1	LYS	B	100	-37.773	31.898	26.029	1.00	0.00	H
ATOM	985	HZ2	LYS	B	100	-39.333	32.501	26.312	1.00	0.00	H
ATOM	986	HZ3	LYS	B	100	-38.711	32.471	24.765	1.00	0.00	H
ATOM	987	C	LYS	B	100	-41.788	32.495	25.138	1.00	0.00	C
ATOM	988	O	LYS	B	100	-40.968	31.998	25.912	1.00	0.00	O
ATOM	989	N	MET	B	101	-42.166	31.965	23.964	1.00	0.00	N
ATOM	990	H	MET	B	101	-42.934	32.428	23.515	1.00	0.00	H
ATOM	991	CA	MET	B	101	-41.629	30.714	23.415	1.00	0.00	C
ATOM	992	CB	MET	B	101	-41.735	30.713	21.887	1.00	0.00	C
ATOM	993	CG	MET	B	101	-43.156	30.798	21.325	1.00	0.00	C
ATOM	994	SD	MET	B	101	-44.151	29.339	21.675	1.00	0.00	S
ATOM	995	CE	MET	B	101	-45.360	30.107	22.761	1.00	0.00	C
ATOM	996	C	MET	B	101	-40.202	30.352	23.792	1.00	0.00	C
ATOM	997	O	MET	B	101	-39.292	31.181	23.831	1.00	0.00	O
ATOM	998	N	LYS	B	102	-40.044	29.057	24.073	1.00	0.00	N
ATOM	999	H	LYS	B	102	-40.827	28.446	23.972	1.00	0.00	H
ATOM	1000	CA	LYS	B	102	-38.694	28.512	24.108	1.00	0.00	C
ATOM	1001	CB	LYS	B	102	-38.611	27.224	24.936	1.00	0.00	C
ATOM	1002	CG	LYS	B	102	-39.251	27.219	26.322	1.00	0.00	C
ATOM	1003	CD	LYS	B	102	-39.014	25.868	27.008	1.00	0.00	C
ATOM	1004	CE	LYS	B	102	-39.836	25.660	28.283	1.00	0.00	C
ATOM	1005	NZ	LYS	B	102	-41.246	25.438	27.938	1.00	0.00	N
ATOM	1006	HZ1	LYS	B	102	-41.470	25.894	27.030	1.00	0.00	H
ATOM	1007	HZ2	LYS	B	102	-41.475	24.431	27.869	1.00	0.00	H
ATOM	1008	HZ3	LYS	B	102	-41.874	25.897	28.634	1.00	0.00	H
ATOM	1009	C	LYS	B	102	-38.230	28.179	22.703	1.00	0.00	C
ATOM	1010	O	LYS	B	102	-38.180	27.015	22.331	1.00	0.00	O
ATOM	1011	N	ASP	B	103	-37.886	29.238	21.948	1.00	0.00	N
ATOM	1012	H	ASP	B	103	-37.959	30.171	22.296	1.00	0.00	H
ATOM	1013	CA	ASP	B	103	-37.201	28.935	20.689	1.00	0.00	C
ATOM	1014	CB	ASP	B	103	-37.240	30.109	19.689	1.00	0.00	C
ATOM	1015	CG	ASP	B	103	-36.356	29.859	18.463	1.00	0.00	C
ATOM	1016	OD1	ASP	B	103	-36.100	28.710	18.100	1.00	0.00	O
ATOM	1017	OD2	ASP	B	103	-35.794	30.814	17.936	1.00	0.00	O
ATOM	1018	C	ASP	B	103	-35.790	28.438	20.944	1.00	0.00	C
ATOM	1019	O	ASP	B	103	-34.827	29.189	21.099	1.00	0.00	O
ATOM	1020	N	LEU	B	104	-35.720	27.110	20.993	1.00	0.00	N
ATOM	1021	H	LEU	B	104	-36.573	26.611	20.816	1.00	0.00	H
ATOM	1022	CA	LEU	B	104	-34.429	26.507	21.282	1.00	0.00	C
ATOM	1023	CB	LEU	B	104	-34.618	25.118	21.898	1.00	0.00	C

ATOM	1024	CG	LEU	B	104	-35.343	25.146	23.246	1.00	0.00	C
ATOM	1025	CD1	LEU	B	104	-35.495	23.737	23.817	1.00	0.00	C
ATOM	1026	CD2	LEU	B	104	-34.678	26.091	24.250	1.00	0.00	C
ATOM	1027	C	LEU	B	104	-33.483	26.487	20.095	1.00	0.00	C
ATOM	1028	O	LEU	B	104	-33.742	27.031	19.025	1.00	0.00	O
ATOM	1029	N	SER	B	105	-32.336	25.849	20.344	1.00	0.00	N
ATOM	1030	H	SER	B	105	-32.184	25.378	21.211	1.00	0.00	H
ATOM	1031	CA	SER	B	105	-31.349	25.756	19.275	1.00	0.00	C
ATOM	1032	CB	SER	B	105	-30.049	25.273	19.914	1.00	0.00	C
ATOM	1033	OG	SER	B	105	-28.911	25.748	19.196	1.00	0.00	O
ATOM	1034	HG	SER	B	105	-28.170	25.297	19.601	1.00	0.00	H
ATOM	1035	C	SER	B	105	-31.849	24.871	18.133	1.00	0.00	C
ATOM	1036	O	SER	B	105	-32.467	23.834	18.361	1.00	0.00	O
ATOM	1037	N	PRO	B	106	-31.623	25.351	16.889	1.00	0.00	N
ATOM	1038	CD	PRO	B	106	-30.851	26.535	16.533	1.00	0.00	C
ATOM	1039	CA	PRO	B	106	-32.220	24.686	15.729	1.00	0.00	C
ATOM	1040	CB	PRO	B	106	-32.023	25.729	14.624	1.00	0.00	C
ATOM	1041	CG	PRO	B	106	-30.764	26.498	15.016	1.00	0.00	C
ATOM	1042	C	PRO	B	106	-31.588	23.350	15.390	1.00	0.00	C
ATOM	1043	O	PRO	B	106	-30.423	23.088	15.652	1.00	0.00	O
ATOM	1044	N	ARG	B	107	-32.424	22.524	14.750	1.00	0.00	N
ATOM	1045	H	ARG	B	107	-33.362	22.818	14.572	1.00	0.00	H
ATOM	1046	CA	ARG	B	107	-31.835	21.448	13.965	1.00	0.00	C
ATOM	1047	CB	ARG	B	107	-32.571	20.127	14.197	1.00	0.00	C
ATOM	1048	CG	ARG	B	107	-32.205	19.559	15.564	1.00	0.00	C
ATOM	1049	CD	ARG	B	107	-32.795	18.202	15.955	1.00	0.00	C
ATOM	1050	NE	ARG	B	107	-32.255	17.839	17.265	1.00	0.00	N
ATOM	1051	HE	ARG	B	107	-32.430	18.423	18.061	1.00	0.00	H
ATOM	1052	CZ	ARG	B	107	-31.262	16.935	17.384	1.00	0.00	C
ATOM	1053	NH1	ARG	B	107	-31.029	16.041	16.432	1.00	0.00	N
ATOM	1054	HH11	ARG	B	107	-31.587	15.980	15.609	1.00	0.00	H
ATOM	1055	HH12	ARG	B	107	-30.250	15.410	16.556	1.00	0.00	H
ATOM	1056	NH2	ARG	B	107	-30.493	16.954	18.463	1.00	0.00	N
ATOM	1057	HH21	ARG	B	107	-30.677	17.603	19.209	1.00	0.00	H
ATOM	1058	HH22	ARG	B	107	-29.695	16.337	18.515	1.00	0.00	H
ATOM	1059	C	ARG	B	107	-31.896	21.848	12.512	1.00	0.00	C
ATOM	1060	O	ARG	B	107	-32.936	22.258	12.010	1.00	0.00	O
ATOM	1061	N	TRP	B	108	-30.738	21.745	11.860	1.00	0.00	N
ATOM	1062	H	TRP	B	108	-29.925	21.368	12.304	1.00	0.00	H
ATOM	1063	CA	TRP	B	108	-30.785	22.020	10.429	1.00	0.00	C
ATOM	1064	CB	TRP	B	108	-29.542	22.769	9.942	1.00	0.00	C
ATOM	1065	CG	TRP	B	108	-29.418	24.148	10.556	1.00	0.00	C
ATOM	1066	CD2	TRP	B	108	-29.290	25.410	9.867	1.00	0.00	C
ATOM	1067	CE2	TRP	B	108	-29.151	26.430	10.867	1.00	0.00	C
ATOM	1068	CE3	TRP	B	108	-29.268	25.763	8.501	1.00	0.00	C
ATOM	1069	CD1	TRP	B	108	-29.353	24.473	11.920	1.00	0.00	C
ATOM	1070	NE1	TRP	B	108	-29.196	25.808	12.107	1.00	0.00	N
ATOM	1071	HE1	TRP	B	108	-29.066	26.247	12.975	1.00	0.00	H
ATOM	1072	CZ2	TRP	B	108	-28.989	27.778	10.483	1.00	0.00	C
ATOM	1073	CZ3	TRP	B	108	-29.106	27.116	8.134	1.00	0.00	C
ATOM	1074	CH2	TRP	B	108	-28.966	28.119	9.116	1.00	0.00	C
ATOM	1075	C	TRP	B	108	-30.897	20.702	9.711	1.00	0.00	C
ATOM	1076	O	TRP	B	108	-30.254	19.732	10.093	1.00	0.00	O
ATOM	1077	N	TYR	B	109	-31.764	20.695	8.699	1.00	0.00	N
ATOM	1078	H	TYR	B	109	-32.225	21.533	8.400	1.00	0.00	H
ATOM	1079	CA	TYR	B	109	-31.976	19.425	8.016	1.00	0.00	C
ATOM	1080	CB	TYR	B	109	-33.467	19.093	7.998	1.00	0.00	C
ATOM	1081	CG	TYR	B	109	-34.001	18.822	9.386	1.00	0.00	C
ATOM	1082	CD1	TYR	B	109	-34.568	19.882	10.124	1.00	0.00	C

ATOM	1083	CE1	TYR	B	109	-35.104	19.617	11.395	1.00	0.00	C
ATOM	1084	CD2	TYR	B	109	-33.933	17.511	9.898	1.00	0.00	C
ATOM	1085	CE2	TYR	B	109	-34.474	17.246	11.167	1.00	0.00	C
ATOM	1086	CZ	TYR	B	109	-35.064	18.301	11.894	1.00	0.00	C
ATOM	1087	OH	TYR	B	109	-35.628	18.036	13.132	1.00	0.00	O
ATOM	1088	HH	TYR	B	109	-35.774	17.093	13.171	1.00	0.00	H
ATOM	1089	C	TYR	B	109	-31.434	19.488	6.607	1.00	0.00	C
ATOM	1090	O	TYR	B	109	-31.495	20.530	5.968	1.00	0.00	O
ATOM	1091	N	PHE	B	110	-30.895	18.351	6.153	1.00	0.00	N
ATOM	1092	H	PHE	B	110	-30.914	17.519	6.708	1.00	0.00	H
ATOM	1093	CA	PHE	B	110	-30.386	18.358	4.784	1.00	0.00	C
ATOM	1094	CB	PHE	B	110	-29.100	17.520	4.707	1.00	0.00	C
ATOM	1095	CG	PHE	B	110	-28.482	17.585	3.327	1.00	0.00	C
ATOM	1096	CD1	PHE	B	110	-27.876	18.782	2.885	1.00	0.00	C
ATOM	1097	CD2	PHE	B	110	-28.534	16.443	2.499	1.00	0.00	C
ATOM	1098	CE1	PHE	B	110	-27.321	18.837	1.592	1.00	0.00	C
ATOM	1099	CE2	PHE	B	110	-27.981	16.497	1.205	1.00	0.00	C
ATOM	1100	CZ	PHE	B	110	-27.380	17.694	0.766	1.00	0.00	C
ATOM	1101	C	PHE	B	110	-31.428	17.875	3.791	1.00	0.00	C
ATOM	1102	O	PHE	B	110	-32.202	16.968	4.082	1.00	0.00	O
ATOM	1103	N	TYR	B	111	-31.403	18.509	2.615	1.00	0.00	N
ATOM	1104	H	TYR	B	111	-30.775	19.278	2.474	1.00	0.00	H
ATOM	1105	CA	TYR	B	111	-32.253	18.108	1.496	1.00	0.00	C
ATOM	1106	CB	TYR	B	111	-33.439	19.066	1.336	1.00	0.00	C
ATOM	1107	CG	TYR	B	111	-34.167	19.283	2.643	1.00	0.00	C
ATOM	1108	CD1	TYR	B	111	-33.848	20.416	3.417	1.00	0.00	C
ATOM	1109	CE1	TYR	B	111	-34.536	20.636	4.619	1.00	0.00	C
ATOM	1110	CD2	TYR	B	111	-35.143	18.355	3.055	1.00	0.00	C
ATOM	1111	CE2	TYR	B	111	-35.835	18.577	4.257	1.00	0.00	C
ATOM	1112	CZ	TYR	B	111	-35.532	19.726	5.017	1.00	0.00	C
ATOM	1113	OH	TYR	B	111	-36.238	19.979	6.182	1.00	0.00	O
ATOM	1114	HH	TYR	B	111	-36.639	19.150	6.438	1.00	0.00	H
ATOM	1115	C	TYR	B	111	-31.407	18.147	0.239	1.00	0.00	C
ATOM	1116	O	TYR	B	111	-30.318	18.710	0.250	1.00	0.00	O
ATOM	1117	N	TYR	B	112	-31.915	17.533	-0.835	1.00	0.00	N
ATOM	1118	H	TYR	B	112	-32.871	17.237	-0.881	1.00	0.00	H
ATOM	1119	CA	TYR	B	112	-31.150	17.673	-2.071	1.00	0.00	C
ATOM	1120	CB	TYR	B	112	-31.320	16.427	-2.942	1.00	0.00	C
ATOM	1121	CG	TYR	B	112	-30.013	15.684	-3.033	1.00	0.00	C
ATOM	1122	CD1	TYR	B	112	-29.689	14.714	-2.061	1.00	0.00	C
ATOM	1123	CE1	TYR	B	112	-28.457	14.046	-2.156	1.00	0.00	C
ATOM	1124	CD2	TYR	B	112	-29.148	15.995	-4.098	1.00	0.00	C
ATOM	1125	CE2	TYR	B	112	-27.919	15.331	-4.189	1.00	0.00	C
ATOM	1126	CZ	TYR	B	112	-27.586	14.368	-3.217	1.00	0.00	C
ATOM	1127	OH	TYR	B	112	-26.368	13.723	-3.309	1.00	0.00	O
ATOM	1128	HH	TYR	B	112	-25.829	14.211	-3.932	1.00	0.00	H
ATOM	1129	C	TYR	B	112	-31.590	18.919	-2.809	1.00	0.00	C
ATOM	1130	O	TYR	B	112	-32.623	19.492	-2.495	1.00	0.00	O
ATOM	1131	N	LEU	B	113	-30.786	19.335	-3.803	1.00	0.00	N
ATOM	1132	H	LEU	B	113	-29.946	18.841	-4.037	1.00	0.00	H
ATOM	1133	CA	LEU	B	113	-31.293	20.450	-4.607	1.00	0.00	C
ATOM	1134	CB	LEU	B	113	-30.207	20.954	-5.566	1.00	0.00	C
ATOM	1135	CG	LEU	B	113	-30.580	22.236	-6.322	1.00	0.00	C
ATOM	1136	CD1	LEU	B	113	-30.904	23.385	-5.370	1.00	0.00	C
ATOM	1137	CD2	LEU	B	113	-29.516	22.646	-7.338	1.00	0.00	C
ATOM	1138	C	LEU	B	113	-32.547	20.041	-5.363	1.00	0.00	C
ATOM	1139	O	LEU	B	113	-32.599	18.958	-5.932	1.00	0.00	O
ATOM	1140	N	GLY	B	114	-33.563	20.911	-5.322	1.00	0.00	N
ATOM	1141	H	GLY	B	114	-33.540	21.720	-4.734	1.00	0.00	H

ATOM	1142	CA	GLY	B	114	-34.811	20.495	-5.957	1.00	0.00	C
ATOM	1143	C	GLY	B	114	-35.757	19.809	-4.991	1.00	0.00	C
ATOM	1144	O	GLY	B	114	-36.924	20.159	-4.847	1.00	0.00	O
ATOM	1145	N	THR	B	115	-35.173	18.818	-4.306	1.00	0.00	N
ATOM	1146	H	THR	B	115	-34.196	18.638	-4.389	1.00	0.00	H
ATOM	1147	CA	THR	B	115	-35.995	18.116	-3.337	1.00	0.00	C
ATOM	1148	CB	THR	B	115	-35.462	16.685	-3.116	1.00	0.00	C
ATOM	1149	OG1	THR	B	115	-34.378	16.595	-2.183	1.00	0.00	O
ATOM	1150	HG1	THR	B	115	-34.161	15.662	-2.173	1.00	0.00	H
ATOM	1151	CG2	THR	B	115	-35.017	16.072	-4.441	1.00	0.00	C
ATOM	1152	C	THR	B	115	-36.168	18.922	-2.060	1.00	0.00	C
ATOM	1153	O	THR	B	115	-35.661	20.030	-1.923	1.00	0.00	O
ATOM	1154	N	GLY	B	116	-36.916	18.340	-1.117	1.00	0.00	N
ATOM	1155	H	GLY	B	116	-37.380	17.477	-1.307	1.00	0.00	H
ATOM	1156	CA	GLY	B	116	-37.181	19.124	0.083	1.00	0.00	C
ATOM	1157	C	GLY	B	116	-38.252	20.179	-0.140	1.00	0.00	C
ATOM	1158	O	GLY	B	116	-38.763	20.366	-1.236	1.00	0.00	O
ATOM	1159	N	PRO	B	117	-38.607	20.858	0.972	1.00	0.00	N
ATOM	1160	CD	PRO	B	117	-38.013	20.740	2.297	1.00	0.00	C
ATOM	1161	CA	PRO	B	117	-39.731	21.800	0.921	1.00	0.00	C
ATOM	1162	CB	PRO	B	117	-39.961	22.114	2.404	1.00	0.00	C
ATOM	1163	CG	PRO	B	117	-38.604	21.907	3.075	1.00	0.00	C
ATOM	1164	C	PRO	B	117	-39.525	23.032	0.050	1.00	0.00	C
ATOM	1165	O	PRO	B	117	-40.485	23.637	-0.404	1.00	0.00	O
ATOM	1166	N	GLU	B	118	-38.247	23.380	-0.161	1.00	0.00	N
ATOM	1167	H	GLU	B	118	-37.471	22.827	0.147	1.00	0.00	H
ATOM	1168	CA	GLU	B	118	-37.996	24.595	-0.936	1.00	0.00	C
ATOM	1169	CB	GLU	B	118	-36.641	25.184	-0.541	1.00	0.00	C
ATOM	1170	CG	GLU	B	118	-36.476	25.422	0.969	1.00	0.00	C
ATOM	1171	CD	GLU	B	118	-36.992	26.776	1.443	1.00	0.00	C
ATOM	1172	OE1	GLU	B	118	-37.362	27.622	0.633	1.00	0.00	O
ATOM	1173	OE2	GLU	B	118	-36.966	27.035	2.648	1.00	0.00	O
ATOM	1174	C	GLU	B	118	-38.080	24.413	-2.447	1.00	0.00	C
ATOM	1175	O	GLU	B	118	-37.892	25.352	-3.213	1.00	0.00	O
ATOM	1176	N	ALA	B	119	-38.368	23.154	-2.851	1.00	0.00	N
ATOM	1177	H	ALA	B	119	-38.468	22.415	-2.188	1.00	0.00	H
ATOM	1178	CA	ALA	B	119	-38.480	22.836	-4.277	1.00	0.00	C
ATOM	1179	CB	ALA	B	119	-39.729	23.495	-4.889	1.00	0.00	C
ATOM	1180	C	ALA	B	119	-37.213	23.179	-5.055	1.00	0.00	C
ATOM	1181	O	ALA	B	119	-36.133	23.318	-4.484	1.00	0.00	O
ATOM	1182	N	GLY	B	120	-37.362	23.337	-6.379	1.00	0.00	N
ATOM	1183	H	GLY	B	120	-38.218	23.131	-6.857	1.00	0.00	H
ATOM	1184	CA	GLY	B	120	-36.190	23.757	-7.141	1.00	0.00	C
ATOM	1185	C	GLY	B	120	-35.889	25.244	-7.100	1.00	0.00	C
ATOM	1186	O	GLY	B	120	-35.825	25.916	-8.124	1.00	0.00	O
ATOM	1187	N	LEU	B	121	-35.658	25.739	-5.874	1.00	0.00	N
ATOM	1188	H	LEU	B	121	-35.740	25.148	-5.070	1.00	0.00	H
ATOM	1189	CA	LEU	B	121	-35.026	27.054	-5.807	1.00	0.00	C
ATOM	1190	CB	LEU	B	121	-35.022	27.599	-4.375	1.00	0.00	C
ATOM	1191	CG	LEU	B	121	-36.385	28.072	-3.869	1.00	0.00	C
ATOM	1192	CD1	LEU	B	121	-36.273	28.645	-2.456	1.00	0.00	C
ATOM	1193	CD2	LEU	B	121	-37.061	29.059	-4.822	1.00	0.00	C
ATOM	1194	C	LEU	B	121	-33.608	26.983	-6.347	1.00	0.00	C
ATOM	1195	O	LEU	B	121	-32.870	26.037	-6.094	1.00	0.00	O
ATOM	1196	N	PRO	B	122	-33.240	28.011	-7.143	1.00	0.00	N
ATOM	1197	CD	PRO	B	122	-34.012	29.192	-7.516	1.00	0.00	C
ATOM	1198	CA	PRO	B	122	-31.906	27.980	-7.740	1.00	0.00	C
ATOM	1199	CB	PRO	B	122	-31.914	29.220	-8.642	1.00	0.00	C
ATOM	1200	CG	PRO	B	122	-32.960	30.160	-8.040	1.00	0.00	C

```
ATOM   1201  C   PRO B 122     -30.804  27.985  -6.696  1.00  0.00           C
ATOM   1202  O   PRO B 122     -30.825  28.724  -5.718  1.00  0.00           O
ATOM   1203  N   TYR B 123     -29.809  27.127  -6.971  1.00  0.00           N
ATOM   1204  H   TYR B 123     -29.937  26.488  -7.727  1.00  0.00           H
ATOM   1205  CA  TYR B 123     -28.603  27.118  -6.143  1.00  0.00           C
ATOM   1206  CB  TYR B 123     -27.636  26.055  -6.691  1.00  0.00           C
ATOM   1207  CG  TYR B 123     -26.373  25.948  -5.863  1.00  0.00           C
ATOM   1208  CD1 TYR B 123     -26.390  25.186  -4.679  1.00  0.00           C
ATOM   1209  CE1 TYR B 123     -25.220  25.108  -3.908  1.00  0.00           C
ATOM   1210  CD2 TYR B 123     -25.211  26.616  -6.300  1.00  0.00           C
ATOM   1211  CE2 TYR B 123     -24.046  26.548  -5.523  1.00  0.00           C
ATOM   1212  CZ  TYR B 123     -24.072  25.810  -4.325  1.00  0.00           C
ATOM   1213  OH  TYR B 123     -22.936  25.786  -3.540  1.00  0.00           O
ATOM   1214  HH  TYR B 123     -23.194  25.764  -2.619  1.00  0.00           H
ATOM   1215  C   TYR B 123     -27.945  28.490  -6.068  1.00  0.00           C
ATOM   1216  O   TYR B 123     -27.227  28.903  -6.976  1.00  0.00           O
ATOM   1217  N   GLY B 124     -28.216  29.168  -4.952  1.00  0.00           N
ATOM   1218  H   GLY B 124     -28.859  28.785  -4.288  1.00  0.00           H
ATOM   1219  CA  GLY B 124     -27.711  30.532  -4.845  1.00  0.00           C
ATOM   1220  C   GLY B 124     -28.792  31.599  -4.765  1.00  0.00           C
ATOM   1221  O   GLY B 124     -28.531  32.785  -4.915  1.00  0.00           O
ATOM   1222  N   ALA B 125     -30.027  31.147  -4.487  1.00  0.00           N
ATOM   1223  H   ALA B 125     -30.247  30.172  -4.473  1.00  0.00           H
ATOM   1224  CA  ALA B 125     -31.036  32.148  -4.144  1.00  0.00           C
ATOM   1225  CB  ALA B 125     -32.417  31.501  -4.018  1.00  0.00           C
ATOM   1226  C   ALA B 125     -30.688  32.832  -2.834  1.00  0.00           C
ATOM   1227  O   ALA B 125     -30.159  32.210  -1.918  1.00  0.00           O
ATOM   1228  N   ASN B 126     -30.973  34.142  -2.787  1.00  0.00           N
ATOM   1229  H   ASN B 126     -31.462  34.569  -3.546  1.00  0.00           H
ATOM   1230  CA  ASN B 126     -30.536  34.905  -1.614  1.00  0.00           C
ATOM   1231  CB  ASN B 126     -30.428  36.401  -1.935  1.00  0.00           C
ATOM   1232  CG  ASN B 126     -29.661  37.121  -0.836  1.00  0.00           C
ATOM   1233  OD1 ASN B 126     -28.436  37.170  -0.821  1.00  0.00           O
ATOM   1234  ND2 ASN B 126     -30.437  37.699   0.083  1.00  0.00           N
ATOM   1235 HD21 ASN B 126     -30.048  38.282   0.793  1.00  0.00           H
ATOM   1236 HD22 ASN B 126     -31.426  37.527   0.068  1.00  0.00           H
ATOM   1237  C   ASN B 126     -31.377  34.680  -0.372  1.00  0.00           C
ATOM   1238  O   ASN B 126     -32.211  35.496   0.000  1.00  0.00           O
ATOM   1239  N   LYS B 127     -31.109  33.537   0.259  1.00  0.00           N
ATOM   1240  H   LYS B 127     -30.420  32.906  -0.102  1.00  0.00           H
ATOM   1241  CA  LYS B 127     -31.908  33.204   1.423  1.00  0.00           C
ATOM   1242  CB  LYS B 127     -32.889  32.081   1.064  1.00  0.00           C
ATOM   1243  CG  LYS B 127     -34.167  32.065   1.909  1.00  0.00           C
ATOM   1244  CD  LYS B 127     -35.076  30.884   1.565  1.00  0.00           C
ATOM   1245  CE  LYS B 127     -36.338  30.806   2.427  1.00  0.00           C
ATOM   1246  NZ  LYS B 127     -37.051  29.557   2.143  1.00  0.00           N
ATOM   1247  HZ1 LYS B 127     -37.247  29.375   1.134  1.00  0.00           H
ATOM   1248  HZ2 LYS B 127     -36.496  28.706   2.384  1.00  0.00           H
ATOM   1249  HZ3 LYS B 127     -37.943  29.434   2.651  1.00  0.00           H
ATOM   1250  C   LYS B 127     -31.035  32.841   2.605  1.00  0.00           C
ATOM   1251  O   LYS B 127     -29.996  32.185   2.495  1.00  0.00           O
ATOM   1252  N   ASP B 128     -31.500  33.302   3.765  1.00  0.00           N
ATOM   1253  H   ASP B 128     -32.349  33.824   3.820  1.00  0.00           H
ATOM   1254  CA  ASP B 128     -30.915  32.680   4.940  1.00  0.00           C
ATOM   1255  CB  ASP B 128     -30.764  33.679   6.082  1.00  0.00           C
ATOM   1256  CG  ASP B 128     -29.680  33.151   6.991  1.00  0.00           C
ATOM   1257  OD1 ASP B 128     -29.977  32.730   8.097  1.00  0.00           O
ATOM   1258  OD2 ASP B 128     -28.524  33.104   6.595  1.00  0.00           O
ATOM   1259  C   ASP B 128     -31.720  31.449   5.307  1.00  0.00           C
```

ATOM	1260	O	ASP	B	128	-32.719	31.152	4.662	1.00	0.00	O
ATOM	1261	N	GLY	B	129	-31.238	30.694	6.297	1.00	0.00	N
ATOM	1262	H	GLY	B	129	-30.439	31.008	6.816	1.00	0.00	H
ATOM	1263	CA	GLY	B	129	-31.917	29.424	6.538	1.00	0.00	C
ATOM	1264	C	GLY	B	129	-31.448	28.296	5.634	1.00	0.00	C
ATOM	1265	O	GLY	B	129	-31.197	27.189	6.087	1.00	0.00	O
ATOM	1266	N	ILE	B	130	-31.329	28.625	4.342	1.00	0.00	N
ATOM	1267	H	ILE	B	130	-31.637	29.520	4.022	1.00	0.00	H
ATOM	1268	CA	ILE	B	130	-30.742	27.661	3.419	1.00	0.00	C
ATOM	1269	CB	ILE	B	130	-31.389	27.815	2.038	1.00	0.00	C
ATOM	1270	CG2	ILE	B	130	-30.739	26.895	1.011	1.00	0.00	C
ATOM	1271	CG1	ILE	B	130	-32.897	27.576	2.092	1.00	0.00	C
ATOM	1272	CD1	ILE	B	130	-33.524	27.677	0.700	1.00	0.00	C
ATOM	1273	C	ILE	B	130	-29.230	27.803	3.322	1.00	0.00	C
ATOM	1274	O	ILE	B	130	-28.703	28.870	3.018	1.00	0.00	O
ATOM	1275	N	ILE	B	131	-28.549	26.682	3.576	1.00	0.00	N
ATOM	1276	H	ILE	B	131	-29.068	25.863	3.822	1.00	0.00	H
ATOM	1277	CA	ILE	B	131	-27.134	26.617	3.221	1.00	0.00	C
ATOM	1278	CB	ILE	B	131	-26.336	25.879	4.303	1.00	0.00	C
ATOM	1279	CG2	ILE	B	131	-24.837	25.872	3.995	1.00	0.00	C
ATOM	1280	CG1	ILE	B	131	-26.622	26.438	5.694	1.00	0.00	C
ATOM	1281	CD1	ILE	B	131	-26.008	25.580	6.800	1.00	0.00	C
ATOM	1282	C	ILE	B	131	-26.988	25.901	1.892	1.00	0.00	C
ATOM	1283	O	ILE	B	131	-27.268	24.715	1.785	1.00	0.00	O
ATOM	1284	N	TRP	B	132	-26.559	26.673	0.886	1.00	0.00	N
ATOM	1285	H	TRP	B	132	-26.261	27.613	1.048	1.00	0.00	H
ATOM	1286	CA	TRP	B	132	-26.424	26.061	-0.435	1.00	0.00	C
ATOM	1287	CB	TRP	B	132	-26.536	27.123	-1.530	1.00	0.00	C
ATOM	1288	CG	TRP	B	132	-27.860	27.844	-1.594	1.00	0.00	C
ATOM	1289	CD2	TRP	B	132	-29.122	27.355	-2.093	1.00	0.00	C
ATOM	1290	CE2	TRP	B	132	-30.055	28.444	-2.021	1.00	0.00	C
ATOM	1291	CE3	TRP	B	132	-29.540	26.103	-2.591	1.00	0.00	C
ATOM	1292	CD1	TRP	B	132	-28.093	29.183	-1.245	1.00	0.00	C
ATOM	1293	NE1	TRP	B	132	-29.379	29.541	-1.497	1.00	0.00	N
ATOM	1294	HE1	TRP	B	132	-29.766	30.433	-1.347	1.00	0.00	H
ATOM	1295	CZ2	TRP	B	132	-31.386	28.260	-2.450	1.00	0.00	C
ATOM	1296	CZ3	TRP	B	132	-30.874	25.933	-3.014	1.00	0.00	C
ATOM	1297	CH2	TRP	B	132	-31.791	27.003	-2.945	1.00	0.00	C
ATOM	1298	C	TRP	B	132	-25.091	25.345	-0.616	1.00	0.00	C
ATOM	1299	O	TRP	B	132	-24.078	25.966	-0.928	1.00	0.00	O
ATOM	1300	N	VAL	B	133	-25.103	24.025	-0.421	1.00	0.00	N
ATOM	1301	H	VAL	B	133	-25.961	23.520	-0.291	1.00	0.00	H
ATOM	1302	CA	VAL	B	133	-23.818	23.350	-0.613	1.00	0.00	C
ATOM	1303	CB	VAL	B	133	-23.516	22.377	0.535	1.00	0.00	C
ATOM	1304	CG1	VAL	B	133	-23.227	23.151	1.820	1.00	0.00	C
ATOM	1305	CG2	VAL	B	133	-24.607	21.319	0.724	1.00	0.00	C
ATOM	1306	C	VAL	B	133	-23.661	22.679	-1.967	1.00	0.00	C
ATOM	1307	O	VAL	B	133	-24.594	22.101	-2.512	1.00	0.00	O
ATOM	1308	N	ALA	B	134	-22.426	22.802	-2.477	1.00	0.00	N
ATOM	1309	H	ALA	B	134	-21.724	23.334	-2.004	1.00	0.00	H
ATOM	1310	CA	ALA	B	134	-22.024	22.116	-3.704	1.00	0.00	C
ATOM	1311	CB	ALA	B	134	-22.475	22.861	-4.958	1.00	0.00	C
ATOM	1312	C	ALA	B	134	-20.519	22.084	-3.760	1.00	0.00	C
ATOM	1313	O	ALA	B	134	-19.884	22.988	-3.232	1.00	0.00	O
ATOM	1314	N	THR	B	135	-19.979	21.056	-4.415	1.00	0.00	N
ATOM	1315	H	THR	B	135	-20.514	20.293	-4.781	1.00	0.00	H
ATOM	1316	CA	THR	B	135	-18.558	21.130	-4.750	1.00	0.00	C
ATOM	1317	CB	THR	B	135	-18.014	19.706	-4.862	1.00	0.00	C
ATOM	1318	OG1	THR	B	135	-19.029	18.846	-5.395	1.00	0.00	O

ATOM	1319	HG1	THR	B	135	-18.713	17.955	-5.248	1.00	0.00	H
ATOM	1320	CG2	THR	B	135	-17.557	19.175	-3.504	1.00	0.00	C
ATOM	1321	C	THR	B	135	-18.346	21.939	-6.023	1.00	0.00	C
ATOM	1322	O	THR	B	135	-19.304	22.453	-6.607	1.00	0.00	O
ATOM	1323	N	GLU	B	136	-17.072	22.065	-6.426	1.00	0.00	N
ATOM	1324	H	GLU	B	136	-16.311	21.658	-5.913	1.00	0.00	H
ATOM	1325	CA	GLU	B	136	-16.831	22.737	-7.701	1.00	0.00	C
ATOM	1326	CB	GLU	B	136	-15.411	23.333	-7.733	1.00	0.00	C
ATOM	1327	CG	GLU	B	136	-15.115	24.355	-8.850	1.00	0.00	C
ATOM	1328	CD	GLU	B	136	-16.098	25.517	-8.811	1.00	0.00	C
ATOM	1329	OE1	GLU	B	136	-15.776	26.553	-8.244	1.00	0.00	O
ATOM	1330	OE2	GLU	B	136	-17.210	25.395	-9.324	1.00	0.00	O
ATOM	1331	C	GLU	B	136	-17.164	21.857	-8.898	1.00	0.00	C
ATOM	1332	O	GLU	B	136	-17.225	20.637	-8.801	1.00	0.00	O
ATOM	1333	N	GLY	B	137	-17.438	22.523	-10.030	1.00	0.00	N
ATOM	1334	H	GLY	B	137	-17.366	23.523	-10.041	1.00	0.00	H
ATOM	1335	CA	GLY	B	137	-17.898	21.780	-11.198	1.00	0.00	C
ATOM	1336	C	GLY	B	137	-19.389	21.510	-11.156	1.00	0.00	C
ATOM	1337	O	GLY	B	137	-20.174	22.115	-11.875	1.00	0.00	O
ATOM	1338	N	ALA	B	138	-19.718	20.579	-10.246	1.00	0.00	N
ATOM	1339	H	ALA	B	138	-18.955	20.187	-9.728	1.00	0.00	H
ATOM	1340	CA	ALA	B	138	-21.072	20.070	-10.016	1.00	0.00	C
ATOM	1341	CB	ALA	B	138	-21.285	19.867	-8.514	1.00	0.00	C
ATOM	1342	C	ALA	B	138	-22.253	20.845	-10.594	1.00	0.00	C
ATOM	1343	O	ALA	B	138	-22.660	21.903	-10.114	1.00	0.00	O
ATOM	1344	N	LEU	B	139	-22.811	20.242	-11.655	1.00	0.00	N
ATOM	1345	H	LEU	B	139	-22.373	19.414	-12.016	1.00	0.00	H
ATOM	1346	CA	LEU	B	139	-23.977	20.830	-12.312	1.00	0.00	C
ATOM	1347	CB	LEU	B	139	-24.453	19.972	-13.484	1.00	0.00	C
ATOM	1348	CG	LEU	B	139	-23.443	19.889	-14.631	1.00	0.00	C
ATOM	1349	CD1	LEU	B	139	-23.876	18.872	-15.687	1.00	0.00	C
ATOM	1350	CD2	LEU	B	139	-23.142	21.260	-15.239	1.00	0.00	C
ATOM	1351	C	LEU	B	139	-25.137	21.112	-11.381	1.00	0.00	C
ATOM	1352	O	LEU	B	139	-25.635	20.260	-10.648	1.00	0.00	O
ATOM	1353	N	ASN	B	140	-25.530	22.396	-11.455	1.00	0.00	N
ATOM	1354	H	ASN	B	140	-25.101	22.941	-12.174	1.00	0.00	H
ATOM	1355	CA	ASN	B	140	-26.510	22.988	-10.535	1.00	0.00	C
ATOM	1356	CB	ASN	B	140	-26.377	24.525	-10.516	1.00	0.00	C
ATOM	1357	CG	ASN	B	140	-25.179	25.098	-9.755	1.00	0.00	C
ATOM	1358	OD1	ASN	B	140	-25.193	26.258	-9.342	1.00	0.00	O
ATOM	1359	ND2	ASN	B	140	-24.117	24.297	-9.599	1.00	0.00	N
ATOM	1360	HD21	ASN	B	140	-23.289	24.623	-9.150	1.00	0.00	H
ATOM	1361	HD22	ASN	B	140	-24.035	23.344	-9.909	1.00	0.00	H
ATOM	1362	C	ASN	B	140	-27.959	22.619	-10.842	1.00	0.00	C
ATOM	1363	O	ASN	B	140	-28.837	23.467	-10.913	1.00	0.00	O
ATOM	1364	N	THR	B	141	-28.167	21.318	-11.054	1.00	0.00	N
ATOM	1365	H	THR	B	141	-27.460	20.637	-10.872	1.00	0.00	H
ATOM	1366	CA	THR	B	141	-29.474	20.825	-11.465	1.00	0.00	C
ATOM	1367	CB	THR	B	141	-29.230	19.571	-12.311	1.00	0.00	C
ATOM	1368	OG1	THR	B	141	-28.216	18.735	-11.712	1.00	0.00	O
ATOM	1369	HG1	THR	B	141	-28.046	18.062	-12.369	1.00	0.00	H
ATOM	1370	CG2	THR	B	141	-28.804	19.956	-13.728	1.00	0.00	C
ATOM	1371	C	THR	B	141	-30.368	20.508	-10.279	1.00	0.00	C
ATOM	1372	O	THR	B	141	-29.886	20.068	-9.243	1.00	0.00	O
ATOM	1373	N	PRO	B	142	-31.695	20.720	-10.460	1.00	0.00	N
ATOM	1374	CD	PRO	B	142	-32.334	21.521	-11.496	1.00	0.00	C
ATOM	1375	CA	PRO	B	142	-32.659	20.065	-9.564	1.00	0.00	C
ATOM	1376	CB	PRO	B	142	-34.010	20.548	-10.104	1.00	0.00	C
ATOM	1377	CG	PRO	B	142	-33.710	21.811	-10.912	1.00	0.00	C

ATOM	1378	C	PRO	B	142	-32.507	18.552	-9.657	1.00	0.00	C
ATOM	1379	O	PRO	B	142	-32.192	18.021	-10.715	1.00	0.00	O
ATOM	1380	N	LYS	B	143	-32.672	17.890	-8.507	1.00	0.00	N
ATOM	1381	H	LYS	B	143	-33.009	18.355	-7.687	1.00	0.00	H
ATOM	1382	CA	LYS	B	143	-32.239	16.495	-8.442	1.00	0.00	C
ATOM	1383	CB	LYS	B	143	-30.932	16.383	-7.647	1.00	0.00	C
ATOM	1384	CG	LYS	B	143	-29.785	17.113	-8.346	1.00	0.00	C
ATOM	1385	CD	LYS	B	143	-28.539	17.309	-7.490	1.00	0.00	C
ATOM	1386	CE	LYS	B	143	-27.544	18.296	-8.110	1.00	0.00	C
ATOM	1387	NZ	LYS	B	143	-27.062	17.854	-9.425	1.00	0.00	N
ATOM	1388	HZ1	LYS	B	143	-26.103	18.237	-9.584	1.00	0.00	H
ATOM	1389	HZ2	LYS	B	143	-26.997	16.813	-9.452	1.00	0.00	H
ATOM	1390	HZ3	LYS	B	143	-27.693	18.184	-10.183	1.00	0.00	H
ATOM	1391	C	LYS	B	143	-33.296	15.563	-7.888	1.00	0.00	C
ATOM	1392	O	LYS	B	143	-33.030	14.612	-7.162	1.00	0.00	O
ATOM	1393	N	ASP	B	144	-34.536	15.861	-8.291	1.00	0.00	N
ATOM	1394	H	ASP	B	144	-34.732	16.735	-8.739	1.00	0.00	H
ATOM	1395	CA	ASP	B	144	-35.670	15.118	-7.737	1.00	0.00	C
ATOM	1396	CB	ASP	B	144	-36.985	15.785	-8.137	1.00	0.00	C
ATOM	1397	CG	ASP	B	144	-37.063	17.131	-7.444	1.00	0.00	C
ATOM	1398	OD1	ASP	B	144	-36.276	18.016	-7.780	1.00	0.00	O
ATOM	1399	OD2	ASP	B	144	-37.901	17.289	-6.560	1.00	0.00	O
ATOM	1400	C	ASP	B	144	-35.701	13.622	-7.966	1.00	0.00	C
ATOM	1401	O	ASP	B	144	-36.340	12.876	-7.238	1.00	0.00	O
ATOM	1402	N	HIS	B	145	-34.926	13.200	-8.980	1.00	0.00	N
ATOM	1403	H	HIS	B	145	-34.476	13.885	-9.548	1.00	0.00	H
ATOM	1404	CA	HIS	B	145	-34.731	11.760	-9.180	1.00	0.00	C
ATOM	1405	CB	HIS	B	145	-33.900	11.497	-10.438	1.00	0.00	C
ATOM	1406	CG	HIS	B	145	-34.575	12.098	-11.648	1.00	0.00	C
ATOM	1407	ND1	HIS	B	145	-35.754	11.672	-12.138	1.00	0.00	N
ATOM	1408	HD1	HIS	B	145	-36.304	10.942	-11.785	1.00	0.00	H
ATOM	1409	CD2	HIS	B	145	-34.118	13.159	-12.435	1.00	0.00	C
ATOM	1410	NE2	HIS	B	145	-35.042	13.365	-13.404	1.00	0.00	N
ATOM	1411	CE1	HIS	B	145	-36.048	12.452	-13.224	1.00	0.00	C
ATOM	1412	C	HIS	B	145	-34.110	11.026	-7.997	1.00	0.00	C
ATOM	1413	O	HIS	B	145	-34.388	9.864	-7.738	1.00	0.00	O
ATOM	1414	N	ILE	B	146	-33.250	11.776	-7.291	1.00	0.00	N
ATOM	1415	H	ILE	B	146	-33.097	12.730	-7.544	1.00	0.00	H
ATOM	1416	CA	ILE	B	146	-32.664	11.246	-6.059	1.00	0.00	C
ATOM	1417	CB	ILE	B	146	-31.398	12.053	-5.721	1.00	0.00	C
ATOM	1418	CG2	ILE	B	146	-30.679	11.557	-4.464	1.00	0.00	C
ATOM	1419	CG1	ILE	B	146	-30.453	12.063	-6.925	1.00	0.00	C
ATOM	1420	CD1	ILE	B	146	-29.222	12.936	-6.696	1.00	0.00	C
ATOM	1421	C	ILE	B	146	-33.670	11.247	-4.913	1.00	0.00	C
ATOM	1422	O	ILE	B	146	-33.746	10.340	-4.094	1.00	0.00	O
ATOM	1423	N	GLY	B	147	-34.481	12.313	-4.923	1.00	0.00	N
ATOM	1424	H	GLY	B	147	-34.308	13.068	-5.553	1.00	0.00	H
ATOM	1425	CA	GLY	B	147	-35.596	12.321	-3.983	1.00	0.00	C
ATOM	1426	C	GLY	B	147	-35.210	12.740	-2.579	1.00	0.00	C
ATOM	1427	O	GLY	B	147	-34.325	13.565	-2.359	1.00	0.00	O
ATOM	1428	N	THR	B	148	-35.938	12.129	-1.635	1.00	0.00	N
ATOM	1429	H	THR	B	148	-36.641	11.466	-1.890	1.00	0.00	H
ATOM	1430	CA	THR	B	148	-35.650	12.337	-0.222	1.00	0.00	C
ATOM	1431	CB	THR	B	148	-36.534	13.452	0.362	1.00	0.00	C
ATOM	1432	OG1	THR	B	148	-37.911	13.291	-0.008	1.00	0.00	O
ATOM	1433	HG1	THR	B	148	-37.919	13.343	-0.965	1.00	0.00	H
ATOM	1434	CG2	THR	B	148	-36.054	14.844	-0.041	1.00	0.00	C
ATOM	1435	C	THR	B	148	-35.824	11.025	0.524	1.00	0.00	C
ATOM	1436	O	THR	B	148	-36.485	10.107	0.047	1.00	0.00	O

ATOM	1437	N	ARG	B	149	-35.180	10.940	1.695	1.00	0.00	N
ATOM	1438	H	ARG	B	149	-34.752	11.729	2.139	1.00	0.00	H
ATOM	1439	CA	ARG	B	149	-35.188	9.647	2.375	1.00	0.00	C
ATOM	1440	CB	ARG	B	149	-33.968	9.523	3.284	1.00	0.00	C
ATOM	1441	CG	ARG	B	149	-32.645	9.563	2.522	1.00	0.00	C
ATOM	1442	CD	ARG	B	149	-32.340	8.288	1.741	1.00	0.00	C
ATOM	1443	NE	ARG	B	149	-32.094	7.204	2.680	1.00	0.00	N
ATOM	1444	HE	ARG	B	149	-32.670	7.161	3.505	1.00	0.00	H
ATOM	1445	CZ	ARG	B	149	-31.192	6.235	2.471	1.00	0.00	C
ATOM	1446	NH1	ARG	B	149	-30.503	6.173	1.333	1.00	0.00	N
ATOM	1447	HH11	ARG	B	149	-30.682	6.835	0.605	1.00	0.00	H
ATOM	1448	HH12	ARG	B	149	-29.789	5.480	1.180	1.00	0.00	H
ATOM	1449	NH2	ARG	B	149	-31.012	5.348	3.437	1.00	0.00	N
ATOM	1450	HH21	ARG	B	149	-31.455	5.511	4.328	1.00	0.00	H
ATOM	1451	HH22	ARG	B	149	-30.465	4.513	3.316	1.00	0.00	H
ATOM	1452	C	ARG	B	149	-36.456	9.356	3.152	1.00	0.00	C
ATOM	1453	O	ARG	B	149	-36.570	9.607	4.345	1.00	0.00	O
ATOM	1454	N	ASN	B	150	-37.414	8.777	2.410	1.00	0.00	N
ATOM	1455	H	ASN	B	150	-37.249	8.727	1.423	1.00	0.00	H
ATOM	1456	CA	ASN	B	150	-38.604	8.217	3.062	1.00	0.00	C
ATOM	1457	CB	ASN	B	150	-39.494	7.534	2.007	1.00	0.00	C
ATOM	1458	CG	ASN	B	150	-40.717	6.872	2.631	1.00	0.00	C
ATOM	1459	OD1	ASN	B	150	-40.629	5.989	3.478	1.00	0.00	O
ATOM	1460	ND2	ASN	B	150	-41.878	7.335	2.167	1.00	0.00	N
ATOM	1461	HD21	ASN	B	150	-42.726	6.945	2.523	1.00	0.00	H
ATOM	1462	HD22	ASN	B	150	-41.916	8.052	1.473	1.00	0.00	H
ATOM	1463	C	ASN	B	150	-38.232	7.232	4.164	1.00	0.00	C
ATOM	1464	O	ASN	B	150	-37.593	6.224	3.898	1.00	0.00	O
ATOM	1465	N	PRO	B	151	-38.661	7.544	5.409	1.00	0.00	N
ATOM	1466	CD	PRO	B	151	-39.458	8.706	5.790	1.00	0.00	C
ATOM	1467	CA	PRO	B	151	-38.297	6.704	6.555	1.00	0.00	C
ATOM	1468	CB	PRO	B	151	-39.154	7.290	7.682	1.00	0.00	C
ATOM	1469	CG	PRO	B	151	-39.328	8.761	7.307	1.00	0.00	C
ATOM	1470	C	PRO	B	151	-38.464	5.199	6.370	1.00	0.00	C
ATOM	1471	O	PRO	B	151	-37.541	4.417	6.559	1.00	0.00	O
ATOM	1472	N	ALA	B	152	-39.699	4.826	6.001	1.00	0.00	N
ATOM	1473	H	ALA	B	152	-40.349	5.506	5.660	1.00	0.00	H
ATOM	1474	CA	ALA	B	152	-40.000	3.395	5.969	1.00	0.00	C
ATOM	1475	CB	ALA	B	152	-41.508	3.172	5.840	1.00	0.00	C
ATOM	1476	C	ALA	B	152	-39.293	2.646	4.853	1.00	0.00	C
ATOM	1477	O	ALA	B	152	-38.817	1.528	5.000	1.00	0.00	O
ATOM	1478	N	ASN	B	153	-39.240	3.331	3.702	1.00	0.00	N
ATOM	1479	H	ASN	B	153	-39.605	4.263	3.669	1.00	0.00	H
ATOM	1480	CA	ASN	B	153	-38.555	2.693	2.579	1.00	0.00	C
ATOM	1481	CB	ASN	B	153	-38.958	3.399	1.276	1.00	0.00	C
ATOM	1482	CG	ASN	B	153	-38.292	2.755	0.076	1.00	0.00	C
ATOM	1483	OD1	ASN	B	153	-37.144	3.027	-0.241	1.00	0.00	O
ATOM	1484	ND2	ASN	B	153	-39.066	1.892	-0.587	1.00	0.00	N
ATOM	1485	HD21	ASN	B	153	-38.689	1.451	-1.400	1.00	0.00	H
ATOM	1486	HD22	ASN	B	153	-40.000	1.685	-0.298	1.00	0.00	H
ATOM	1487	C	ASN	B	153	-37.044	2.640	2.770	1.00	0.00	C
ATOM	1488	O	ASN	B	153	-36.394	1.602	2.678	1.00	0.00	O
ATOM	1489	N	ASN	B	154	-36.521	3.835	3.045	1.00	0.00	N
ATOM	1490	H	ASN	B	154	-37.135	4.618	3.130	1.00	0.00	H
ATOM	1491	CA	ASN	B	154	-35.084	4.048	3.175	1.00	0.00	C
ATOM	1492	CB	ASN	B	154	-34.440	4.297	1.806	1.00	0.00	C
ATOM	1493	CG	ASN	B	154	-33.916	3.009	1.210	1.00	0.00	C
ATOM	1494	OD1	ASN	B	154	-33.073	2.318	1.774	1.00	0.00	O
ATOM	1495	ND2	ASN	B	154	-34.415	2.716	0.011	1.00	0.00	N

```
ATOM   1496 HD21 ASN B 154      -34.052   1.992  -0.569  1.00  0.00           H
ATOM   1497 HD22 ASN B 154      -35.211   3.224  -0.333  1.00  0.00           H
ATOM   1498  C   ASN B 154      -34.807   5.243   4.063  1.00  0.00           C
ATOM   1499  O   ASN B 154      -34.514   6.330   3.582  1.00  0.00           O
ATOM   1500  N   ALA B 155      -34.887   4.995   5.380  1.00  0.00           N
ATOM   1501  H   ALA B 155      -35.319   4.147   5.684  1.00  0.00           H
ATOM   1502  CA  ALA B 155      -34.605   6.055   6.357  1.00  0.00           C
ATOM   1503  CB  ALA B 155      -34.536   5.467   7.768  1.00  0.00           C
ATOM   1504  C   ALA B 155      -33.344   6.876   6.120  1.00  0.00           C
ATOM   1505  O   ALA B 155      -32.420   6.460   5.422  1.00  0.00           O
ATOM   1506  N   ALA B 156      -33.369   8.075   6.723  1.00  0.00           N
ATOM   1507  H   ALA B 156      -34.141   8.311   7.309  1.00  0.00           H
ATOM   1508  CA  ALA B 156      -32.241   8.993   6.567  1.00  0.00           C
ATOM   1509  CB  ALA B 156      -32.563  10.353   7.193  1.00  0.00           C
ATOM   1510  C   ALA B 156      -30.949   8.466   7.159  1.00  0.00           C
ATOM   1511  O   ALA B 156      -30.937   7.606   8.031  1.00  0.00           O
ATOM   1512  N   ILE B 157      -29.857   9.002   6.610  1.00  0.00           N
ATOM   1513  H   ILE B 157      -29.939   9.777   5.979  1.00  0.00           H
ATOM   1514  CA  ILE B 157      -28.555   8.476   7.015  1.00  0.00           C
ATOM   1515  CB  ILE B 157      -27.682   8.272   5.769  1.00  0.00           C
ATOM   1516  CG2 ILE B 157      -26.415   7.471   6.068  1.00  0.00           C
ATOM   1517  CG1 ILE B 157      -28.483   7.659   4.620  1.00  0.00           C
ATOM   1518  CD1 ILE B 157      -28.612   8.619   3.436  1.00  0.00           C
ATOM   1519  C   ILE B 157      -27.894   9.438   7.981  1.00  0.00           C
ATOM   1520  O   ILE B 157      -28.167  10.629   7.935  1.00  0.00           O
ATOM   1521  N   VAL B 158      -27.009   8.911   8.843  1.00  0.00           N
ATOM   1522  H   VAL B 158      -26.768   7.942   8.795  1.00  0.00           H
ATOM   1523  CA  VAL B 158      -26.232   9.853   9.650  1.00  0.00           C
ATOM   1524  CB  VAL B 158      -25.410   9.108  10.716  1.00  0.00           C
ATOM   1525  CG1 VAL B 158      -24.495  10.034  11.524  1.00  0.00           C
ATOM   1526  CG2 VAL B 158      -26.337   8.318  11.642  1.00  0.00           C
ATOM   1527  C   VAL B 158      -25.355  10.742   8.782  1.00  0.00           C
ATOM   1528  O   VAL B 158      -24.591  10.285   7.936  1.00  0.00           O
ATOM   1529  N   LEU B 159      -25.535  12.046   9.024  1.00  0.00           N
ATOM   1530  H   LEU B 159      -26.189  12.320   9.730  1.00  0.00           H
ATOM   1531  CA  LEU B 159      -24.766  13.034   8.281  1.00  0.00           C
ATOM   1532  CB  LEU B 159      -25.280  14.432   8.642  1.00  0.00           C
ATOM   1533  CG  LEU B 159      -24.854  15.545   7.681  1.00  0.00           C
ATOM   1534  CD1 LEU B 159      -26.018  16.477   7.365  1.00  0.00           C
ATOM   1535  CD2 LEU B 159      -23.626  16.312   8.172  1.00  0.00           C
ATOM   1536  C   LEU B 159      -23.270  12.885   8.489  1.00  0.00           C
ATOM   1537  O   LEU B 159      -22.783  12.606   9.577  1.00  0.00           O
ATOM   1538  N   GLN B 160      -22.559  13.082   7.376  1.00  0.00           N
ATOM   1539  H   GLN B 160      -23.017  13.273   6.510  1.00  0.00           H
ATOM   1540  CA  GLN B 160      -21.113  12.977   7.480  1.00  0.00           C
ATOM   1541  CB  GLN B 160      -20.690  11.562   7.088  1.00  0.00           C
ATOM   1542  CG  GLN B 160      -19.259  11.160   7.450  1.00  0.00           C
ATOM   1543  CD  GLN B 160      -19.092   9.684   7.142  1.00  0.00           C
ATOM   1544  OE1 GLN B 160      -19.844   9.092   6.372  1.00  0.00           O
ATOM   1545  NE2 GLN B 160      -18.084   9.099   7.792  1.00  0.00           N
ATOM   1546 HE21 GLN B 160      -17.909   8.123   7.666  1.00  0.00           H
ATOM   1547 HE22 GLN B 160      -17.493   9.608   8.417  1.00  0.00           H
ATOM   1548  C   GLN B 160      -20.452  14.044   6.640  1.00  0.00           C
ATOM   1549  O   GLN B 160      -20.450  13.985   5.414  1.00  0.00           O
ATOM   1550  N   LEU B 161      -19.921  15.038   7.366  1.00  0.00           N
ATOM   1551  H   LEU B 161      -19.921  14.985   8.363  1.00  0.00           H
ATOM   1552  CA  LEU B 161      -19.196  16.089   6.657  1.00  0.00           C
ATOM   1553  CB  LEU B 161      -19.184  17.414   7.430  1.00  0.00           C
ATOM   1554  CG  LEU B 161      -20.454  18.250   7.266  1.00  0.00           C
```

ATOM	1555	CD1	LEU	B	161	-20.186	19.726	7.557	1.00	0.00	C
ATOM	1556	CD2	LEU	B	161	-21.079	18.092	5.884	1.00	0.00	C
ATOM	1557	C	LEU	B	161	-17.772	15.697	6.340	1.00	0.00	C
ATOM	1558	O	LEU	B	161	-17.059	15.141	7.171	1.00	0.00	O
ATOM	1559	N	PRO	B	162	-17.363	16.027	5.097	1.00	0.00	N
ATOM	1560	CD	PRO	B	162	-18.145	16.649	4.034	1.00	0.00	C
ATOM	1561	CA	PRO	B	162	-15.975	15.790	4.719	1.00	0.00	C
ATOM	1562	CB	PRO	B	162	-16.013	16.069	3.211	1.00	0.00	C
ATOM	1563	CG	PRO	B	162	-17.114	17.105	3.013	1.00	0.00	C
ATOM	1564	C	PRO	B	162	-15.044	16.679	5.530	1.00	0.00	C
ATOM	1565	O	PRO	B	162	-15.125	17.907	5.549	1.00	0.00	O
ATOM	1566	N	GLN	B	163	-14.156	15.969	6.240	1.00	0.00	N
ATOM	1567	H	GLN	B	163	-14.154	14.973	6.176	1.00	0.00	H
ATOM	1568	CA	GLN	B	163	-13.228	16.691	7.104	1.00	0.00	C
ATOM	1569	CB	GLN	B	163	-12.559	15.727	8.087	1.00	0.00	C
ATOM	1570	CG	GLN	B	163	-13.564	15.016	9.006	1.00	0.00	C
ATOM	1571	CD	GLN	B	163	-14.306	16.024	9.869	1.00	0.00	C
ATOM	1572	OE1	GLN	B	163	-13.767	16.614	10.793	1.00	0.00	O
ATOM	1573	NE2	GLN	B	163	-15.584	16.203	9.522	1.00	0.00	N
ATOM	1574	HE21	GLN	B	163	-16.103	16.876	10.043	1.00	0.00	H
ATOM	1575	HE22	GLN	B	163	-16.014	15.692	8.774	1.00	0.00	H
ATOM	1576	C	GLN	B	163	-12.235	17.524	6.320	1.00	0.00	C
ATOM	1577	O	GLN	B	163	-11.393	17.029	5.577	1.00	0.00	O
ATOM	1578	N	GLY	B	164	-12.460	18.823	6.485	1.00	0.00	N
ATOM	1579	H	GLY	B	164	-13.085	19.076	7.226	1.00	0.00	H
ATOM	1580	CA	GLY	B	164	-11.856	19.871	5.670	1.00	0.00	C
ATOM	1581	C	GLY	B	164	-12.701	21.130	5.762	1.00	0.00	C
ATOM	1582	O	GLY	B	164	-12.229	22.254	5.663	1.00	0.00	O
ATOM	1583	N	THR	B	165	-13.997	20.865	5.994	1.00	0.00	N
ATOM	1584	H	THR	B	165	-14.311	19.918	5.975	1.00	0.00	H
ATOM	1585	CA	THR	B	165	-14.945	21.942	6.269	1.00	0.00	C
ATOM	1586	CB	THR	B	165	-16.321	21.295	6.496	1.00	0.00	C
ATOM	1587	OG1	THR	B	165	-16.613	20.367	5.432	1.00	0.00	O
ATOM	1588	HG1	THR	B	165	-16.109	19.571	5.596	1.00	0.00	H
ATOM	1589	CG2	THR	B	165	-17.452	22.322	6.621	1.00	0.00	C
ATOM	1590	C	THR	B	165	-14.527	22.876	7.412	1.00	0.00	C
ATOM	1591	O	THR	B	165	-13.950	22.452	8.405	1.00	0.00	O
ATOM	1592	N	THR	B	166	-14.834	24.170	7.241	1.00	0.00	N
ATOM	1593	H	THR	B	166	-15.289	24.437	6.392	1.00	0.00	H
ATOM	1594	CA	THR	B	166	-14.462	25.157	8.256	1.00	0.00	C
ATOM	1595	CB	THR	B	166	-14.294	26.527	7.593	1.00	0.00	C
ATOM	1596	OG1	THR	B	166	-13.465	26.398	6.428	1.00	0.00	O
ATOM	1597	HG1	THR	B	166	-13.857	25.734	5.865	1.00	0.00	H
ATOM	1598	CG2	THR	B	166	-13.707	27.583	8.537	1.00	0.00	C
ATOM	1599	C	THR	B	166	-15.423	25.170	9.439	1.00	0.00	C
ATOM	1600	O	THR	B	166	-16.294	26.027	9.628	1.00	0.00	O
ATOM	1601	N	LEU	B	167	-15.217	24.092	10.209	1.00	0.00	N
ATOM	1602	H	LEU	B	167	-14.440	23.501	9.984	1.00	0.00	H
ATOM	1603	CA	LEU	B	167	-16.160	23.677	11.238	1.00	0.00	C
ATOM	1604	CB	LEU	B	167	-15.677	22.405	11.941	1.00	0.00	C
ATOM	1605	CG	LEU	B	167	-15.560	21.182	11.025	1.00	0.00	C
ATOM	1606	CD1	LEU	B	167	-15.006	19.972	11.775	1.00	0.00	C
ATOM	1607	CD2	LEU	B	167	-16.875	20.848	10.321	1.00	0.00	C
ATOM	1608	C	LEU	B	167	-16.497	24.739	12.258	1.00	0.00	C
ATOM	1609	O	LEU	B	167	-15.650	25.265	12.970	1.00	0.00	O
ATOM	1610	N	PRO	B	168	-17.813	25.033	12.289	1.00	0.00	N
ATOM	1611	CD	PRO	B	168	-18.811	24.630	11.301	1.00	0.00	C
ATOM	1612	CA	PRO	B	168	-18.381	25.784	13.413	1.00	0.00	C
ATOM	1613	CB	PRO	B	168	-19.875	25.589	13.185	1.00	0.00	C

ATOM	1614	CG	PRO	B	168	-20.040	25.434	11.675	1.00	0.00	C
ATOM	1615	C	PRO	B	168	-17.939	25.232	14.756	1.00	0.00	C
ATOM	1616	O	PRO	B	168	-17.748	24.030	14.920	1.00	0.00	O
ATOM	1617	N	LYS	B	169	-17.761	26.147	15.713	1.00	0.00	N
ATOM	1618	H	LYS	B	169	-18.051	27.094	15.567	1.00	0.00	H
ATOM	1619	CA	LYS	B	169	-17.350	25.604	17.003	1.00	0.00	C
ATOM	1620	CB	LYS	B	169	-16.611	26.656	17.835	1.00	0.00	C
ATOM	1621	CG	LYS	B	169	-15.515	25.996	18.675	1.00	0.00	C
ATOM	1622	CD	LYS	B	169	-14.736	26.941	19.596	1.00	0.00	C
ATOM	1623	CE	LYS	B	169	-13.628	26.173	20.323	1.00	0.00	C
ATOM	1624	NZ	LYS	B	169	-12.963	27.001	21.337	1.00	0.00	N
ATOM	1625	HZ1	LYS	B	169	-12.269	26.408	21.842	1.00	0.00	H
ATOM	1626	HZ2	LYS	B	169	-12.406	27.775	20.918	1.00	0.00	H
ATOM	1627	HZ3	LYS	B	169	-13.642	27.399	22.011	1.00	0.00	H
ATOM	1628	C	LYS	B	169	-18.518	24.983	17.748	1.00	0.00	C
ATOM	1629	O	LYS	B	169	-19.411	25.670	18.221	1.00	0.00	O
ATOM	1630	N	GLY	B	170	-18.486	23.646	17.785	1.00	0.00	N
ATOM	1631	H	GLY	B	170	-17.687	23.143	17.451	1.00	0.00	H
ATOM	1632	CA	GLY	B	170	-19.683	22.942	18.236	1.00	0.00	C
ATOM	1633	C	GLY	B	170	-20.367	22.136	17.138	1.00	0.00	C
ATOM	1634	O	GLY	B	170	-21.343	21.421	17.357	1.00	0.00	O
ATOM	1635	N	PHE	B	171	-19.802	22.263	15.927	1.00	0.00	N
ATOM	1636	H	PHE	B	171	-18.984	22.814	15.772	1.00	0.00	H
ATOM	1637	CA	PHE	B	171	-20.364	21.455	14.853	1.00	0.00	C
ATOM	1638	CB	PHE	B	171	-19.864	21.893	13.474	1.00	0.00	C
ATOM	1639	CG	PHE	B	171	-20.601	21.123	12.402	1.00	0.00	C
ATOM	1640	CD1	PHE	B	171	-21.861	21.583	11.965	1.00	0.00	C
ATOM	1641	CD2	PHE	B	171	-20.037	19.932	11.892	1.00	0.00	C
ATOM	1642	CE1	PHE	B	171	-22.591	20.807	11.048	1.00	0.00	C
ATOM	1643	CE2	PHE	B	171	-20.771	19.152	10.982	1.00	0.00	C
ATOM	1644	CZ	PHE	B	171	-22.052	19.588	10.587	1.00	0.00	C
ATOM	1645	C	PHE	B	171	-20.084	19.983	15.024	1.00	0.00	C
ATOM	1646	O	PHE	B	171	-18.947	19.529	15.017	1.00	0.00	O
ATOM	1647	N	TYR	B	172	-21.199	19.263	15.121	1.00	0.00	N
ATOM	1648	H	TYR	B	172	-22.099	19.705	15.160	1.00	0.00	H
ATOM	1649	CA	TYR	B	172	-21.128	17.821	14.977	1.00	0.00	C
ATOM	1650	CB	TYR	B	172	-21.098	17.118	16.348	1.00	0.00	C
ATOM	1651	CG	TYR	B	172	-19.927	17.611	17.168	1.00	0.00	C
ATOM	1652	CD1	TYR	B	172	-20.136	18.646	18.104	1.00	0.00	C
ATOM	1653	CE1	TYR	B	172	-19.026	19.222	18.744	1.00	0.00	C
ATOM	1654	CD2	TYR	B	172	-18.649	17.060	16.943	1.00	0.00	C
ATOM	1655	CE2	TYR	B	172	-17.544	17.628	17.596	1.00	0.00	C
ATOM	1656	CZ	TYR	B	172	-17.739	18.728	18.453	1.00	0.00	C
ATOM	1657	OH	TYR	B	172	-16.639	19.344	19.013	1.00	0.00	O
ATOM	1658	HH	TYR	B	172	-16.690	20.285	18.856	1.00	0.00	H
ATOM	1659	C	TYR	B	172	-22.341	17.428	14.169	1.00	0.00	C
ATOM	1660	O	TYR	B	172	-23.326	18.154	14.103	1.00	0.00	O
ATOM	1661	N	ALA	B	173	-22.241	16.256	13.544	1.00	0.00	N
ATOM	1662	H	ALA	B	173	-21.428	15.687	13.639	1.00	0.00	H
ATOM	1663	CA	ALA	B	173	-23.478	15.770	12.945	1.00	0.00	C
ATOM	1664	CB	ALA	B	173	-23.165	14.842	11.781	1.00	0.00	C
ATOM	1665	C	ALA	B	173	-24.246	14.998	13.989	1.00	0.00	C
ATOM	1666	O	ALA	B	173	-23.649	14.409	14.886	1.00	0.00	O
ATOM	1667	N	GLU	B	174	-25.574	15.004	13.867	1.00	0.00	N
ATOM	1668	H	GLU	B	174	-26.038	15.480	13.116	1.00	0.00	H
ATOM	1669	CA	GLU	B	174	-26.307	14.169	14.813	1.00	0.00	C
ATOM	1670	CB	GLU	B	174	-27.766	14.607	14.871	1.00	0.00	C
ATOM	1671	CG	GLU	B	174	-27.907	15.975	15.555	1.00	0.00	C
ATOM	1672	CD	GLU	B	174	-28.029	15.836	17.068	1.00	0.00	C

ATOM	1673	OE1	GLU	B	174	-27.528	16.673	17.803	1.00	0.00	O
ATOM	1674	OE2	GLU	B	174	-28.689	14.920	17.547	1.00	0.00	O
ATOM	1675	C	GLU	B	174	-26.129	12.693	14.511	1.00	0.00	C
ATOM	1676	O	GLU	B	174	-26.665	12.136	13.559	1.00	0.00	O
ATOM	1677	N	GLY	B	175	-25.274	12.123	15.373	1.00	0.00	N
ATOM	1678	H	GLY	B	175	-24.915	12.701	16.104	1.00	0.00	H
ATOM	1679	CA	GLY	B	175	-24.787	10.758	15.185	1.00	0.00	C
ATOM	1680	C	GLY	B	175	-23.343	10.607	15.641	1.00	0.00	C
ATOM	1681	O	GLY	B	175	-22.922	9.578	16.153	1.00	0.00	O
ATOM	1682	N	SER	B	176	-22.604	11.723	15.474	1.00	0.00	N
ATOM	1683	H	SER	B	176	-23.028	12.505	15.019	1.00	0.00	H
ATOM	1684	CA	SER	B	176	-21.192	11.824	15.880	1.00	0.00	C
ATOM	1685	CB	SER	B	176	-20.848	13.297	16.065	1.00	0.00	C
ATOM	1686	OG	SER	B	176	-21.888	13.902	16.851	1.00	0.00	O
ATOM	1687	HG	SER	B	176	-22.437	14.380	16.235	1.00	0.00	H
ATOM	1688	C	SER	B	176	-20.797	11.065	17.139	1.00	0.00	C
ATOM	1689	O	SER	B	176	-19.849	10.293	17.162	1.00	0.00	O
ATOM	1690	N	ARG	B	177	-21.633	11.317	18.164	1.00	0.00	N
ATOM	1691	H	ARG	B	177	-22.261	12.066	17.955	1.00	0.00	H
ATOM	1692	CA	ARG	B	177	-21.560	10.746	19.516	1.00	0.00	C
ATOM	1693	CB	ARG	B	177	-22.851	11.088	20.267	1.00	0.00	C
ATOM	1694	CG	ARG	B	177	-23.156	12.589	20.272	1.00	0.00	C
ATOM	1695	CD	ARG	B	177	-24.550	12.936	20.800	1.00	0.00	C
ATOM	1696	NE	ARG	B	177	-24.761	14.382	20.741	1.00	0.00	N
ATOM	1697	HE	ARG	B	177	-24.141	14.954	21.281	1.00	0.00	H
ATOM	1698	CZ	ARG	B	177	-25.699	14.932	19.937	1.00	0.00	C
ATOM	1699	NH1	ARG	B	177	-26.543	14.179	19.237	1.00	0.00	N
ATOM	1700	HH11	ARG	B	177	-26.540	13.183	19.300	1.00	0.00	H
ATOM	1701	HH12	ARG	B	177	-27.222	14.611	18.622	1.00	0.00	H
ATOM	1702	NH2	ARG	B	177	-25.778	16.253	19.843	1.00	0.00	N
ATOM	1703	HH21	ARG	B	177	-25.196	16.867	20.372	1.00	0.00	H
ATOM	1704	HH22	ARG	B	177	-26.448	16.650	19.202	1.00	0.00	H
ATOM	1705	C	ARG	B	177	-21.262	9.256	19.664	1.00	0.00	C
ATOM	1706	O	ARG	B	177	-20.873	8.775	20.722	1.00	0.00	O
ATOM	1707	N	GLY	B	178	-21.463	8.520	18.564	1.00	0.00	N
ATOM	1708	H	GLY	B	178	-21.797	8.933	17.714	1.00	0.00	H
ATOM	1709	CA	GLY	B	178	-21.027	7.129	18.609	1.00	0.00	C
ATOM	1710	C	GLY	B	178	-19.518	6.923	18.535	1.00	0.00	C
ATOM	1711	O	GLY	B	178	-19.032	5.809	18.673	1.00	0.00	O
ATOM	1712	N	GLY	B	179	-18.787	8.019	18.303	1.00	0.00	N
ATOM	1713	H	GLY	B	179	-19.187	8.936	18.317	1.00	0.00	H
ATOM	1714	CA	GLY	B	179	-17.392	7.869	17.907	1.00	0.00	C
ATOM	1715	C	GLY	B	179	-17.279	7.698	16.407	1.00	0.00	C
ATOM	1716	O	GLY	B	179	-16.778	6.698	15.903	1.00	0.00	O
ATOM	1717	N	SER	B	180	-17.839	8.710	15.723	1.00	0.00	N
ATOM	1718	H	SER	B	180	-18.143	9.501	16.256	1.00	0.00	H
ATOM	1719	CA	SER	B	180	-17.815	8.775	14.257	1.00	0.00	C
ATOM	1720	CB	SER	B	180	-16.388	9.013	13.756	1.00	0.00	C
ATOM	1721	OG	SER	B	180	-15.763	9.993	14.588	1.00	0.00	O
ATOM	1722	HG	SER	B	180	-14.845	9.723	14.636	1.00	0.00	H
ATOM	1723	C	SER	B	180	-18.467	7.628	13.495	1.00	0.00	C
ATOM	1724	O	SER	B	180	-18.168	7.353	12.338	1.00	0.00	O
ATOM	1725	N	GLN	B	181	-19.394	6.955	14.191	1.00	0.00	N
ATOM	1726	H	GLN	B	181	-19.688	7.286	15.085	1.00	0.00	H
ATOM	1727	CA	GLN	B	181	-20.027	5.816	13.528	1.00	0.00	C
ATOM	1728	CB	GLN	B	181	-20.426	4.758	14.567	1.00	0.00	C
ATOM	1729	CG	GLN	B	181	-19.283	4.287	15.482	1.00	0.00	C
ATOM	1730	CD	GLN	B	181	-18.220	3.532	14.701	1.00	0.00	C
ATOM	1731	OE1	GLN	B	181	-18.360	2.368	14.358	1.00	0.00	O

ATOM	1732	NE2	GLN	B	181	-17.123	4.245	14.435	1.00	0.00	N
ATOM	1733	HE21	GLN	B	181	-16.410	3.831	13.876	1.00	0.00	H
ATOM	1734	HE22	GLN	B	181	-17.012	5.180	14.778	1.00	0.00	H
ATOM	1735	C	GLN	B	181	-21.196	6.219	12.638	1.00	0.00	C
ATOM	1736	O	GLN	B	181	-22.361	6.094	12.991	1.00	0.00	O
ATOM	1737	N	ALA	B	182	-20.824	6.746	11.463	1.00	0.00	N
ATOM	1738	H	ALA	B	182	-19.851	6.755	11.229	1.00	0.00	H
ATOM	1739	CA	ALA	B	182	-21.878	7.126	10.525	1.00	0.00	C
ATOM	1740	CB	ALA	B	182	-21.398	8.236	9.589	1.00	0.00	C
ATOM	1741	C	ALA	B	182	-22.358	5.952	9.695	1.00	0.00	C
ATOM	1742	O	ALA	B	182	-21.607	5.040	9.376	1.00	0.00	O
ATOM	1743	N	SER	B	183	-23.653	6.002	9.369	1.00	0.00	N
ATOM	1744	H	SER	B	183	-24.249	6.775	9.580	1.00	0.00	H
ATOM	1745	CA	SER	B	183	-24.220	4.831	8.714	1.00	0.00	C
ATOM	1746	CB	SER	B	183	-25.713	4.758	9.054	1.00	0.00	C
ATOM	1747	OG	SER	B	183	-26.257	6.076	9.227	1.00	0.00	O
ATOM	1748	HG	SER	B	183	-27.091	5.942	9.669	1.00	0.00	H
ATOM	1749	C	SER	B	183	-23.957	4.709	7.218	1.00	0.00	C
ATOM	1750	O	SER	B	183	-23.536	5.635	6.524	1.00	0.00	O
ATOM	1751	N	SER	B	184	-24.251	3.487	6.742	1.00	0.00	N
ATOM	1752	H	SER	B	184	-24.568	2.781	7.372	1.00	0.00	H
ATOM	1753	CA	SER	B	184	-24.328	3.290	5.298	1.00	0.00	C
ATOM	1754	CB	SER	B	184	-24.404	1.791	4.987	1.00	0.00	C
ATOM	1755	OG	SER	B	184	-24.470	1.569	3.573	1.00	0.00	O
ATOM	1756	HG	SER	B	184	-23.575	1.636	3.253	1.00	0.00	H
ATOM	1757	C	SER	B	184	-25.536	4.014	4.735	1.00	0.00	C
ATOM	1758	O	SER	B	184	-26.539	4.217	5.406	1.00	0.00	O
ATOM	1759	N	ARG	B	185	-25.405	4.399	3.462	1.00	0.00	N
ATOM	1760	H	ARG	B	185	-24.644	4.056	2.906	1.00	0.00	H
ATOM	1761	CA	ARG	B	185	-26.448	5.237	2.872	1.00	0.00	C
ATOM	1762	CB	ARG	B	185	-25.838	6.174	1.828	1.00	0.00	C
ATOM	1763	CG	ARG	B	185	-25.119	7.419	2.355	1.00	0.00	C
ATOM	1764	CD	ARG	B	185	-23.956	7.164	3.315	1.00	0.00	C
ATOM	1765	NE	ARG	B	185	-23.209	8.396	3.497	1.00	0.00	N
ATOM	1766	HE	ARG	B	185	-23.302	9.103	2.792	1.00	0.00	H
ATOM	1767	CZ	ARG	B	185	-22.298	8.574	4.467	1.00	0.00	C
ATOM	1768	NH1	ARG	B	185	-22.142	7.727	5.480	1.00	0.00	N
ATOM	1769	HH11	ARG	B	185	-22.749	6.936	5.627	1.00	0.00	H
ATOM	1770	HH12	ARG	B	185	-21.398	7.881	6.138	1.00	0.00	H
ATOM	1771	NH2	ARG	B	185	-21.525	9.642	4.382	1.00	0.00	N
ATOM	1772	HH21	ARG	B	185	-21.663	10.292	3.628	1.00	0.00	H
ATOM	1773	HH22	ARG	B	185	-20.803	9.792	5.064	1.00	0.00	H
ATOM	1774	C	ARG	B	185	-27.568	4.430	2.243	1.00	0.00	C
ATOM	1775	O	ARG	B	185	-28.136	4.802	1.220	1.00	0.00	O
ATOM	1776	N	SER	B	186	-27.809	3.272	2.883	1.00	0.00	N
ATOM	1777	H	SER	B	186	-27.515	3.199	3.835	1.00	0.00	H
ATOM	1778	CA	SER	B	186	-28.357	2.101	2.196	1.00	0.00	C
ATOM	1779	CB	SER	B	186	-29.791	2.317	1.712	1.00	0.00	C
ATOM	1780	OG	SER	B	186	-30.571	2.869	2.778	1.00	0.00	O
ATOM	1781	HG	SER	B	186	-31.461	2.561	2.605	1.00	0.00	H
ATOM	1782	C	SER	B	186	-27.493	1.596	1.057	1.00	0.00	C
ATOM	1783	O	SER	B	186	-27.970	1.003	0.100	1.00	0.00	O
ATOM	1784	N	SER	B	187	-26.184	1.847	1.206	1.00	0.00	N
ATOM	1785	H	SER	B	187	-25.795	2.230	2.045	1.00	0.00	H
ATOM	1786	CA	SER	B	187	-25.279	1.557	0.099	1.00	0.00	C
ATOM	1787	CB	SER	B	187	-23.887	2.061	0.474	1.00	0.00	C
ATOM	1788	OG	SER	B	187	-23.984	3.302	1.201	1.00	0.00	O
ATOM	1789	HG	SER	B	187	-23.680	3.984	0.598	1.00	0.00	H
ATOM	1790	C	SER	B	187	-25.271	0.093	-0.333	1.00	0.00	C

```
ATOM   1791  O   SER B 187     -25.198  -0.249  -1.506  1.00  0.00           O
ATOM   1792  N   SER B 188     -25.417  -0.766   0.687  1.00  0.00           N
ATOM   1793  H   SER B 188     -25.352  -0.477   1.644  1.00  0.00           H
ATOM   1794  CA  SER B 188     -25.562  -2.196   0.417  1.00  0.00           C
ATOM   1795  CB  SER B 188     -25.508  -2.955   1.742  1.00  0.00           C
ATOM   1796  OG  SER B 188     -24.778  -2.173   2.700  1.00  0.00           O
ATOM   1797  HG  SER B 188     -24.625  -2.740   3.447  1.00  0.00           H
ATOM   1798  C   SER B 188     -26.805  -2.595  -0.372  1.00  0.00           C
ATOM   1799  O   SER B 188     -26.840  -3.590  -1.080  1.00  0.00           O
ATOM   1800  N   ARG B 189     -27.850  -1.766  -0.217  1.00  0.00           N
ATOM   1801  H   ARG B 189     -27.737  -0.908   0.279  1.00  0.00           H
ATOM   1802  CA  ARG B 189     -29.019  -1.995  -1.062  1.00  0.00           C
ATOM   1803  CB  ARG B 189     -30.249  -1.251  -0.532  1.00  0.00           C
ATOM   1804  CG  ARG B 189     -30.862  -1.863   0.731  1.00  0.00           C
ATOM   1805  CD  ARG B 189     -32.084  -1.068   1.202  1.00  0.00           C
ATOM   1806  NE  ARG B 189     -32.845  -1.782   2.230  1.00  0.00           N
ATOM   1807  HE  ARG B 189     -32.560  -2.716   2.452  1.00  0.00           H
ATOM   1808  CZ  ARG B 189     -33.954  -1.217   2.764  1.00  0.00           C
ATOM   1809  NH1 ARG B 189     -34.276   0.036   2.473  1.00  0.00           N
ATOM   1810 HH11 ARG B 189     -33.662   0.633   1.947  1.00  0.00           H
ATOM   1811 HH12 ARG B 189     -35.142   0.456   2.767  1.00  0.00           H
ATOM   1812  NH2 ARG B 189     -34.735  -1.917   3.583  1.00  0.00           N
ATOM   1813 HH21 ARG B 189     -34.523  -2.865   3.822  1.00  0.00           H
ATOM   1814 HH22 ARG B 189     -35.557  -1.502   3.980  1.00  0.00           H
ATOM   1815  C   ARG B 189     -28.750  -1.622  -2.507  1.00  0.00           C
ATOM   1816  O   ARG B 189     -29.016  -2.400  -3.418  1.00  0.00           O
ATOM   1817  N   SER B 190     -28.175  -0.416  -2.661  1.00  0.00           N
ATOM   1818  H   SER B 190     -28.041   0.219  -1.896  1.00  0.00           H
ATOM   1819  CA  SER B 190     -27.809   0.063  -3.994  1.00  0.00           C
ATOM   1820  CB  SER B 190     -27.127   1.430  -3.861  1.00  0.00           C
ATOM   1821  OG  SER B 190     -27.774   2.183  -2.824  1.00  0.00           O
ATOM   1822  HG  SER B 190     -27.483   3.092  -2.923  1.00  0.00           H
ATOM   1823  C   SER B 190     -26.957  -0.910  -4.801  1.00  0.00           C
ATOM   1824  O   SER B 190     -27.260  -1.266  -5.937  1.00  0.00           O
ATOM   1825  N   ARG B 191     -25.887  -1.363  -4.133  1.00  0.00           N
ATOM   1826  H   ARG B 191     -25.698  -1.038  -3.206  1.00  0.00           H
ATOM   1827  CA  ARG B 191     -25.022  -2.352  -4.770  1.00  0.00           C
ATOM   1828  CB  ARG B 191     -23.862  -2.710  -3.852  1.00  0.00           C
ATOM   1829  CG  ARG B 191     -22.790  -3.531  -4.567  1.00  0.00           C
ATOM   1830  CD  ARG B 191     -21.709  -4.026  -3.612  1.00  0.00           C
ATOM   1831  NE  ARG B 191     -20.545  -4.492  -4.362  1.00  0.00           N
ATOM   1832  HE  ARG B 191     -20.607  -5.436  -4.704  1.00  0.00           H
ATOM   1833  CZ  ARG B 191     -19.517  -3.646  -4.602  1.00  0.00           C
ATOM   1834  NH1 ARG B 191     -19.560  -2.390  -4.165  1.00  0.00           N
ATOM   1835 HH11 ARG B 191     -20.400  -2.046  -3.730  1.00  0.00           H
ATOM   1836 HH12 ARG B 191     -18.808  -1.724  -4.229  1.00  0.00           H
ATOM   1837  NH2 ARG B 191     -18.456  -4.083  -5.274  1.00  0.00           N
ATOM   1838 HH21 ARG B 191     -18.434  -5.024  -5.609  1.00  0.00           H
ATOM   1839 HH22 ARG B 191     -17.675  -3.484  -5.456  1.00  0.00           H
ATOM   1840  C   ARG B 191     -25.746  -3.634  -5.108  1.00  0.00           C
ATOM   1841  O   ARG B 191     -26.244  -4.324  -4.233  1.00  0.00           O
ATOM   1842  N   ASN B 192     -25.764  -3.959  -6.405  1.00  0.00           N
ATOM   1843  H   ASN B 192     -25.393  -3.335  -7.092  1.00  0.00           H
ATOM   1844  CA  ASN B 192     -26.357  -5.268  -6.665  1.00  0.00           C
ATOM   1845  CB  ASN B 192     -27.109  -5.295  -7.995  1.00  0.00           C
ATOM   1846  CG  ASN B 192     -28.217  -6.327  -7.898  1.00  0.00           C
ATOM   1847  OD1 ASN B 192     -28.942  -6.409  -6.910  1.00  0.00           O
ATOM   1848  ND2 ASN B 192     -28.327  -7.116  -8.969  1.00  0.00           N
ATOM   1849 HD21 ASN B 192     -29.036  -7.819  -8.984  1.00  0.00           H
```

```
ATOM   1850 HD22 ASN B 192     -27.724  -7.018  -9.760  1.00  0.00           H
ATOM   1851  C   ASN B 192     -25.402  -6.442  -6.533  1.00  0.00           C
ATOM   1852  O   ASN B 192     -25.756  -7.538  -6.115  1.00  0.00           O
ATOM   1853  N   SER B 193     -24.157  -6.156  -6.926  1.00  0.00           N
ATOM   1854  H   SER B 193     -23.867  -5.238  -7.196  1.00  0.00           H
ATOM   1855  CA  SER B 193     -23.164  -7.221  -7.014  1.00  0.00           C
ATOM   1856  CB  SER B 193     -22.088  -6.798  -8.018  1.00  0.00           C
ATOM   1857  OG  SER B 193     -22.176  -5.381  -8.250  1.00  0.00           O
ATOM   1858  HG  SER B 193     -21.725  -5.217  -9.072  1.00  0.00           H
ATOM   1859  C   SER B 193     -22.556  -7.665  -5.693  1.00  0.00           C
ATOM   1860  O   SER B 193     -21.712  -6.997  -5.102  1.00  0.00           O
ATOM   1861  N   SER B 194     -23.023  -8.853  -5.275  1.00  0.00           N
ATOM   1862  H   SER B 194     -23.722  -9.297  -5.832  1.00  0.00           H
ATOM   1863  CA  SER B 194     -22.370  -9.572  -4.178  1.00  0.00           C
ATOM   1864  CB  SER B 194     -20.995 -10.076  -4.633  1.00  0.00           C
ATOM   1865  OG  SER B 194     -21.102 -10.516  -5.995  1.00  0.00           O
ATOM   1866  HG  SER B 194     -20.506 -11.249  -6.096  1.00  0.00           H
ATOM   1867  C   SER B 194     -22.310  -8.831  -2.851  1.00  0.00           C
ATOM   1868  O   SER B 194     -21.262  -8.597  -2.257  1.00  0.00           O
ATOM   1869  N   ARG B 195     -23.536  -8.476  -2.431  1.00  0.00           N
ATOM   1870  H   ARG B 195     -24.300  -8.712  -3.028  1.00  0.00           H
ATOM   1871  CA  ARG B 195     -23.748  -7.633  -1.253  1.00  0.00           C
ATOM   1872  CB  ARG B 195     -25.247  -7.546  -0.950  1.00  0.00           C
ATOM   1873  CG  ARG B 195     -25.998  -6.760  -2.020  1.00  0.00           C
ATOM   1874  CD  ARG B 195     -27.512  -6.727  -1.798  1.00  0.00           C
ATOM   1875  NE  ARG B 195     -28.108  -5.668  -2.610  1.00  0.00           N
ATOM   1876  HE  ARG B 195     -27.943  -4.723  -2.307  1.00  0.00           H
ATOM   1877  CZ  ARG B 195     -28.605  -5.892  -3.843  1.00  0.00           C
ATOM   1878  NH1 ARG B 195     -28.653  -7.121  -4.347  1.00  0.00           N
ATOM   1879 HH11 ARG B 195     -28.375  -7.913  -3.809  1.00  0.00           H
ATOM   1880 HH12 ARG B 195     -28.952  -7.247  -5.299  1.00  0.00           H
ATOM   1881  NH2 ARG B 195     -29.033  -4.874  -4.580  1.00  0.00           N
ATOM   1882 HH21 ARG B 195     -28.992  -3.932  -4.225  1.00  0.00           H
ATOM   1883 HH22 ARG B 195     -29.378  -5.033  -5.508  1.00  0.00           H
ATOM   1884  C   ARG B 195     -22.940  -7.949  -0.001  1.00  0.00           C
ATOM   1885  O   ARG B 195     -21.918  -7.329   0.247  1.00  0.00           O
ATOM   1886  N   ASN B 196     -23.447  -8.914   0.789  1.00  0.00           N
ATOM   1887  H   ASN B 196     -24.255  -9.420   0.482  1.00  0.00           H
ATOM   1888  CA  ASN B 196     -23.063  -9.047   2.206  1.00  0.00           C
ATOM   1889  CB  ASN B 196     -23.555 -10.377   2.797  1.00  0.00           C
ATOM   1890  CG  ASN B 196     -25.065 -10.481   2.715  1.00  0.00           C
ATOM   1891  OD1 ASN B 196     -25.673 -10.255   1.677  1.00  0.00           O
ATOM   1892  ND2 ASN B 196     -25.655 -10.836   3.859  1.00  0.00           N
ATOM   1893 HD21 ASN B 196     -26.649 -10.926   3.861  1.00  0.00           H
ATOM   1894 HD22 ASN B 196     -25.134 -11.016   4.692  1.00  0.00           H
ATOM   1895  C   ASN B 196     -21.597  -8.851   2.563  1.00  0.00           C
ATOM   1896  O   ASN B 196     -21.210  -7.923   3.264  1.00  0.00           O
ATOM   1897  N   SER B 197     -20.776  -9.762   2.031  1.00  0.00           N
ATOM   1898  H   SER B 197     -21.105 -10.544   1.506  1.00  0.00           H
ATOM   1899  CA  SER B 197     -19.368  -9.649   2.394  1.00  0.00           C
ATOM   1900  CB  SER B 197     -18.655 -10.967   2.119  1.00  0.00           C
ATOM   1901  OG  SER B 197     -19.221 -11.964   2.968  1.00  0.00           O
ATOM   1902  HG  SER B 197     -18.698 -11.908   3.770  1.00  0.00           H
ATOM   1903  C   SER B 197     -18.602  -8.484   1.799  1.00  0.00           C
ATOM   1904  O   SER B 197     -17.472  -8.207   2.171  1.00  0.00           O
ATOM   1905  N   THR B 198     -19.207  -7.783   0.836  1.00  0.00           N
ATOM   1906  H   THR B 198     -20.164  -7.927   0.586  1.00  0.00           H
ATOM   1907  CA  THR B 198     -18.436  -6.632   0.372  1.00  0.00           C
ATOM   1908  CB  THR B 198     -18.880  -6.203  -1.026  1.00  0.00           C
```

ATOM	1909	OG1	THR	B	198	-18.797	-7.361	-1.874	1.00	0.00	O
ATOM	1910	HG1	THR	B	198	-19.697	-7.694	-1.934	1.00	0.00	H
ATOM	1911	CG2	THR	B	198	-18.022	-5.074	-1.607	1.00	0.00	C
ATOM	1912	C	THR	B	198	-18.185	-5.510	1.383	1.00	0.00	C
ATOM	1913	O	THR	B	198	-17.028	-5.184	1.619	1.00	0.00	O
ATOM	1914	N	PRO	B	199	-19.246	-4.974	2.050	1.00	0.00	N
ATOM	1915	CD	PRO	B	199	-20.671	-4.999	1.738	1.00	0.00	C
ATOM	1916	CA	PRO	B	199	-18.962	-4.260	3.298	1.00	0.00	C
ATOM	1917	CB	PRO	B	199	-20.347	-3.826	3.793	1.00	0.00	C
ATOM	1918	CG	PRO	B	199	-21.363	-4.698	3.060	1.00	0.00	C
ATOM	1919	C	PRO	B	199	-18.166	-5.081	4.308	1.00	0.00	C
ATOM	1920	O	PRO	B	199	-17.171	-4.601	4.832	1.00	0.00	O
ATOM	1921	N	GLY	B	200	-18.616	-6.337	4.523	1.00	0.00	N
ATOM	1922	H	GLY	B	200	-19.467	-6.640	4.092	1.00	0.00	H
ATOM	1923	CA	GLY	B	200	-17.936	-7.240	5.466	1.00	0.00	C
ATOM	1924	C	GLY	B	200	-16.413	-7.185	5.467	1.00	0.00	C
ATOM	1925	O	GLY	B	200	-15.787	-6.578	6.330	1.00	0.00	O
ATOM	1926	N	SER	B	201	-15.838	-7.779	4.417	1.00	0.00	N
ATOM	1927	H	SER	B	201	-16.383	-8.355	3.804	1.00	0.00	H
ATOM	1928	CA	SER	B	201	-14.383	-7.754	4.297	1.00	0.00	C
ATOM	1929	CB	SER	B	201	-13.893	-8.920	3.433	1.00	0.00	C
ATOM	1930	OG	SER	B	201	-14.991	-9.673	2.891	1.00	0.00	O
ATOM	1931	HG	SER	B	201	-15.342	-10.175	3.645	1.00	0.00	H
ATOM	1932	C	SER	B	201	-13.717	-6.449	3.858	1.00	0.00	C
ATOM	1933	O	SER	B	201	-12.646	-6.440	3.257	1.00	0.00	O
ATOM	1934	N	SER	B	202	-14.392	-5.338	4.188	1.00	0.00	N
ATOM	1935	H	SER	B	202	-15.329	-5.396	4.534	1.00	0.00	H
ATOM	1936	CA	SER	B	202	-13.739	-4.034	4.119	1.00	0.00	C
ATOM	1937	CB	SER	B	202	-14.072	-3.347	2.792	1.00	0.00	C
ATOM	1938	OG	SER	B	202	-15.491	-3.185	2.660	1.00	0.00	O
ATOM	1939	HG	SER	B	202	-15.831	-4.032	2.380	1.00	0.00	H
ATOM	1940	C	SER	B	202	-14.124	-3.134	5.287	1.00	0.00	C
ATOM	1941	O	SER	B	202	-13.825	-1.943	5.317	1.00	0.00	O
ATOM	1942	N	ARG	B	203	-14.881	-3.750	6.221	1.00	0.00	N
ATOM	1943	H	ARG	B	203	-15.005	-4.743	6.198	1.00	0.00	H
ATOM	1944	CA	ARG	B	203	-15.658	-3.018	7.225	1.00	0.00	C
ATOM	1945	CB	ARG	B	203	-14.831	-2.746	8.480	1.00	0.00	C
ATOM	1946	CG	ARG	B	203	-14.595	-4.049	9.244	1.00	0.00	C
ATOM	1947	CD	ARG	B	203	-13.959	-3.836	10.616	1.00	0.00	C
ATOM	1948	NE	ARG	B	203	-12.574	-3.383	10.515	1.00	0.00	N
ATOM	1949	HE	ARG	B	203	-11.875	-4.017	10.171	1.00	0.00	H
ATOM	1950	CZ	ARG	B	203	-12.179	-2.154	10.898	1.00	0.00	C
ATOM	1951	NH1	ARG	B	203	-13.056	-1.245	11.319	1.00	0.00	N
ATOM	1952	HH11	ARG	B	203	-14.040	-1.464	11.382	1.00	0.00	H
ATOM	1953	HH12	ARG	B	203	-12.784	-0.323	11.584	1.00	0.00	H
ATOM	1954	NH2	ARG	B	203	-10.883	-1.872	10.846	1.00	0.00	N
ATOM	1955	HH21	ARG	B	203	-10.264	-2.603	10.533	1.00	0.00	H
ATOM	1956	HH22	ARG	B	203	-10.498	-0.989	11.105	1.00	0.00	H
ATOM	1957	C	ARG	B	203	-16.427	-1.790	6.758	1.00	0.00	C
ATOM	1958	O	ARG	B	203	-16.671	-0.850	7.502	1.00	0.00	O
ATOM	1959	N	GLY	B	204	-16.813	-1.847	5.473	1.00	0.00	N
ATOM	1960	H	GLY	B	204	-16.584	-2.662	4.938	1.00	0.00	H
ATOM	1961	CA	GLY	B	204	-17.627	-0.775	4.907	1.00	0.00	C
ATOM	1962	C	GLY	B	204	-16.987	0.602	4.812	1.00	0.00	C
ATOM	1963	O	GLY	B	204	-17.658	1.596	4.555	1.00	0.00	O
ATOM	1964	N	THR	B	205	-15.657	0.630	4.997	1.00	0.00	N
ATOM	1965	H	THR	B	205	-15.175	-0.217	5.221	1.00	0.00	H
ATOM	1966	CA	THR	B	205	-15.015	1.926	5.240	1.00	0.00	C
ATOM	1967	CB	THR	B	205	-13.601	1.703	5.801	1.00	0.00	C

ATOM	1968	OG1	THR	B	205	-13.531	0.463	6.525	1.00	0.00	O
ATOM	1969	HG1	THR	B	205	-13.415	-0.244	5.897	1.00	0.00	H
ATOM	1970	CG2	THR	B	205	-13.191	2.843	6.738	1.00	0.00	C
ATOM	1971	C	THR	B	205	-15.062	2.989	4.127	1.00	0.00	C
ATOM	1972	O	THR	B	205	-14.671	4.140	4.284	1.00	0.00	O
ATOM	1973	N	SER	B	206	-15.611	2.590	2.972	1.00	0.00	N
ATOM	1974	H	SER	B	206	-15.833	1.630	2.810	1.00	0.00	H
ATOM	1975	CA	SER	B	206	-16.218	3.657	2.181	1.00	0.00	C
ATOM	1976	CB	SER	B	206	-15.322	4.126	1.036	1.00	0.00	C
ATOM	1977	OG	SER	B	206	-14.040	4.544	1.525	1.00	0.00	O
ATOM	1978	HG	SER	B	206	-14.124	4.605	2.480	1.00	0.00	H
ATOM	1979	C	SER	B	206	-17.575	3.272	1.632	1.00	0.00	C
ATOM	1980	O	SER	B	206	-17.690	2.512	0.675	1.00	0.00	O
ATOM	1981	N	PRO	B	207	-18.626	3.870	2.240	1.00	0.00	N
ATOM	1982	CD	PRO	B	207	-18.578	4.813	3.353	1.00	0.00	C
ATOM	1983	CA	PRO	B	207	-19.993	3.581	1.789	1.00	0.00	C
ATOM	1984	CB	PRO	B	207	-20.854	4.420	2.743	1.00	0.00	C
ATOM	1985	CG	PRO	B	207	-19.932	5.506	3.301	1.00	0.00	C
ATOM	1986	C	PRO	B	207	-20.239	3.828	0.304	1.00	0.00	C
ATOM	1987	O	PRO	B	207	-21.044	3.167	-0.334	1.00	0.00	O
ATOM	1988	N	ALA	B	208	-19.465	4.775	-0.249	1.00	0.00	N
ATOM	1989	H	ALA	B	208	-18.866	5.331	0.322	1.00	0.00	H
ATOM	1990	CA	ALA	B	208	-19.553	4.993	-1.693	1.00	0.00	C
ATOM	1991	CB	ALA	B	208	-18.684	6.176	-2.088	1.00	0.00	C
ATOM	1992	C	ALA	B	208	-19.158	3.799	-2.538	1.00	0.00	C
ATOM	1993	O	ALA	B	208	-19.815	3.411	-3.495	1.00	0.00	O
ATOM	1994	N	ARG	B	209	-18.043	3.210	-2.103	1.00	0.00	N
ATOM	1995	H	ARG	B	209	-17.618	3.504	-1.251	1.00	0.00	H
ATOM	1996	CA	ARG	B	209	-17.524	2.067	-2.846	1.00	0.00	C
ATOM	1997	CB	ARG	B	209	-16.027	1.955	-2.591	1.00	0.00	C
ATOM	1998	CG	ARG	B	209	-15.377	3.293	-2.934	1.00	0.00	C
ATOM	1999	CD	ARG	B	209	-13.862	3.385	-2.782	1.00	0.00	C
ATOM	2000	NE	ARG	B	209	-13.457	4.734	-3.143	1.00	0.00	N
ATOM	2001	HE	ARG	B	209	-13.667	5.061	-4.073	1.00	0.00	H
ATOM	2002	CZ	ARG	B	209	-13.131	5.676	-2.241	1.00	0.00	C
ATOM	2003	NH1	ARG	B	209	-12.837	5.356	-0.981	1.00	0.00	N
ATOM	2004	HH11	ARG	B	209	-12.858	4.403	-0.680	1.00	0.00	H
ATOM	2005	HH12	ARG	B	209	-12.612	6.046	-0.296	1.00	0.00	H
ATOM	2006	NH2	ARG	B	209	-13.136	6.937	-2.645	1.00	0.00	N
ATOM	2007	HH21	ARG	B	209	-13.554	7.102	-3.557	1.00	0.00	H
ATOM	2008	HH22	ARG	B	209	-12.804	7.714	-2.121	1.00	0.00	H
ATOM	2009	C	ARG	B	209	-18.274	0.779	-2.566	1.00	0.00	C
ATOM	2010	O	ARG	B	209	-18.314	-0.161	-3.357	1.00	0.00	O
ATOM	2011	N	MET	B	210	-18.943	0.816	-1.401	1.00	0.00	N
ATOM	2012	H	MET	B	210	-18.743	1.539	-0.739	1.00	0.00	H
ATOM	2013	CA	MET	B	210	-20.013	-0.151	-1.175	1.00	0.00	C
ATOM	2014	CB	MET	B	210	-20.605	0.005	0.223	1.00	0.00	C
ATOM	2015	CG	MET	B	210	-19.641	-0.294	1.368	1.00	0.00	C
ATOM	2016	SD	MET	B	210	-20.331	0.234	2.944	1.00	0.00	S
ATOM	2017	CE	MET	B	210	-21.925	-0.587	2.829	1.00	0.00	C
ATOM	2018	C	MET	B	210	-21.119	-0.030	-2.211	1.00	0.00	C
ATOM	2019	O	MET	B	210	-21.441	-0.997	-2.882	1.00	0.00	O
ATOM	2020	N	ALA	B	211	-21.662	1.193	-2.331	1.00	0.00	N
ATOM	2021	H	ALA	B	211	-21.307	1.956	-1.789	1.00	0.00	H
ATOM	2022	CA	ALA	B	211	-22.799	1.385	-3.234	1.00	0.00	C
ATOM	2023	CB	ALA	B	211	-23.313	2.819	-3.150	1.00	0.00	C
ATOM	2024	C	ALA	B	211	-22.530	1.073	-4.691	1.00	0.00	C
ATOM	2025	O	ALA	B	211	-23.294	0.390	-5.359	1.00	0.00	O
ATOM	2026	N	GLY	B	212	-21.398	1.611	-5.165	1.00	0.00	N

ATOM	2027	H	GLY	B	212	-20.860	2.223	-4.585	1.00	0.00	H
ATOM	2028	CA	GLY	B	212	-21.125	1.464	-6.591	1.00	0.00	C
ATOM	2029	C	GLY	B	212	-21.898	2.460	-7.436	1.00	0.00	C
ATOM	2030	O	GLY	B	212	-21.351	3.418	-7.966	1.00	0.00	O
ATOM	2031	N	ASN	B	213	-23.210	2.187	-7.522	1.00	0.00	N
ATOM	2032	H	ASN	B	213	-23.524	1.332	-7.101	1.00	0.00	H
ATOM	2033	CA	ASN	B	213	-24.151	2.998	-8.304	1.00	0.00	C
ATOM	2034	CB	ASN	B	213	-25.611	2.592	-8.059	1.00	0.00	C
ATOM	2035	CG	ASN	B	213	-25.855	1.105	-8.227	1.00	0.00	C
ATOM	2036	OD1	ASN	B	213	-24.999	0.320	-8.612	1.00	0.00	O
ATOM	2037	ND2	ASN	B	213	-27.077	0.738	-7.839	1.00	0.00	N
ATOM	2038	HD21	ASN	B	213	-27.263	-0.239	-7.714	1.00	0.00	H
ATOM	2039	HD22	ASN	B	213	-27.793	1.400	-7.632	1.00	0.00	H
ATOM	2040	C	ASN	B	213	-24.049	4.484	-8.021	1.00	0.00	C
ATOM	2041	O	ASN	B	213	-24.571	4.991	-7.035	1.00	0.00	O
ATOM	2042	N	GLY	B	214	-23.332	5.168	-8.921	1.00	0.00	N
ATOM	2043	H	GLY	B	214	-22.811	4.656	-9.602	1.00	0.00	H
ATOM	2044	CA	GLY	B	214	-22.980	6.542	-8.573	1.00	0.00	C
ATOM	2045	C	GLY	B	214	-21.812	6.535	-7.606	1.00	0.00	C
ATOM	2046	O	GLY	B	214	-20.658	6.684	-7.985	1.00	0.00	O
ATOM	2047	N	GLY	B	215	-22.176	6.301	-6.334	1.00	0.00	N
ATOM	2048	H	GLY	B	215	-23.149	6.131	-6.167	1.00	0.00	H
ATOM	2049	CA	GLY	B	215	-21.180	5.983	-5.312	1.00	0.00	C
ATOM	2050	C	GLY	B	215	-19.923	6.834	-5.296	1.00	0.00	C
ATOM	2051	O	GLY	B	215	-19.909	7.975	-4.842	1.00	0.00	O
ATOM	2052	N	ASP	B	216	-18.851	6.212	-5.805	1.00	0.00	N
ATOM	2053	H	ASP	B	216	-18.955	5.343	-6.286	1.00	0.00	H
ATOM	2054	CA	ASP	B	216	-17.582	6.931	-5.714	1.00	0.00	C
ATOM	2055	CB	ASP	B	216	-16.374	6.007	-5.835	1.00	0.00	C
ATOM	2056	CG	ASP	B	216	-15.320	6.485	-4.855	1.00	0.00	C
ATOM	2057	OD1	ASP	B	216	-15.642	6.680	-3.688	1.00	0.00	O
ATOM	2058	OD2	ASP	B	216	-14.160	6.632	-5.226	1.00	0.00	O
ATOM	2059	C	ASP	B	216	-17.440	8.141	-6.612	1.00	0.00	C
ATOM	2060	O	ASP	B	216	-16.693	9.069	-6.345	1.00	0.00	O
ATOM	2061	N	ALA	B	217	-18.269	8.152	-7.667	1.00	0.00	N
ATOM	2062	H	ALA	B	217	-18.863	7.369	-7.854	1.00	0.00	H
ATOM	2063	CA	ALA	B	217	-18.367	9.385	-8.450	1.00	0.00	C
ATOM	2064	CB	ALA	B	217	-19.404	9.218	-9.560	1.00	0.00	C
ATOM	2065	C	ALA	B	217	-18.699	10.621	-7.616	1.00	0.00	C
ATOM	2066	O	ALA	B	217	-18.284	11.738	-7.901	1.00	0.00	O
ATOM	2067	N	ALA	B	218	-19.438	10.357	-6.526	1.00	0.00	N
ATOM	2068	H	ALA	B	218	-19.779	9.434	-6.345	1.00	0.00	H
ATOM	2069	CA	ALA	B	218	-19.525	11.412	-5.527	1.00	0.00	C
ATOM	2070	CB	ALA	B	218	-20.736	11.205	-4.620	1.00	0.00	C
ATOM	2071	C	ALA	B	218	-18.272	11.551	-4.672	1.00	0.00	C
ATOM	2072	O	ALA	B	218	-17.612	12.586	-4.640	1.00	0.00	O
ATOM	2073	N	LEU	B	219	-17.975	10.453	-3.956	1.00	0.00	N
ATOM	2074	H	LEU	B	219	-18.482	9.605	-4.122	1.00	0.00	H
ATOM	2075	CA	LEU	B	219	-16.936	10.559	-2.928	1.00	0.00	C
ATOM	2076	CB	LEU	B	219	-16.837	9.265	-2.115	1.00	0.00	C
ATOM	2077	CG	LEU	B	219	-15.828	9.263	-0.956	1.00	0.00	C
ATOM	2078	CD1	LEU	B	219	-16.070	10.387	0.053	1.00	0.00	C
ATOM	2079	CD2	LEU	B	219	-15.759	7.900	-0.268	1.00	0.00	C
ATOM	2080	C	LEU	B	219	-15.572	11.037	-3.400	1.00	0.00	C
ATOM	2081	O	LEU	B	219	-14.941	11.854	-2.746	1.00	0.00	O
ATOM	2082	N	ALA	B	220	-15.152	10.544	-4.571	1.00	0.00	N
ATOM	2083	H	ALA	B	220	-15.659	9.798	-5.004	1.00	0.00	H
ATOM	2084	CA	ALA	B	220	-13.906	11.039	-5.158	1.00	0.00	C
ATOM	2085	CB	ALA	B	220	-13.703	10.472	-6.564	1.00	0.00	C

ATOM	2086	C	ALA	B	220	-13.793	12.556	-5.214	1.00	0.00	C
ATOM	2087	O	ALA	B	220	-12.791	13.136	-4.815	1.00	0.00	O
ATOM	2088	N	LEU	B	221	-14.882	13.192	-5.679	1.00	0.00	N
ATOM	2089	H	LEU	B	221	-15.721	12.681	-5.873	1.00	0.00	H
ATOM	2090	CA	LEU	B	221	-14.832	14.655	-5.703	1.00	0.00	C
ATOM	2091	CB	LEU	B	221	-15.956	15.215	-6.580	1.00	0.00	C
ATOM	2092	CG	LEU	B	221	-15.884	16.724	-6.850	1.00	0.00	C
ATOM	2093	CD1	LEU	B	221	-14.553	17.145	-7.479	1.00	0.00	C
ATOM	2094	CD2	LEU	B	221	-17.075	17.199	-7.684	1.00	0.00	C
ATOM	2095	C	LEU	B	221	-14.793	15.276	-4.313	1.00	0.00	C
ATOM	2096	O	LEU	B	221	-13.946	16.107	-4.007	1.00	0.00	O
ATOM	2097	N	LEU	B	222	-15.685	14.761	-3.450	1.00	0.00	N
ATOM	2098	H	LEU	B	222	-16.383	14.131	-3.800	1.00	0.00	H
ATOM	2099	CA	LEU	B	222	-15.642	15.144	-2.030	1.00	0.00	C
ATOM	2100	CB	LEU	B	222	-16.719	14.377	-1.263	1.00	0.00	C
ATOM	2101	CG	LEU	B	222	-18.129	14.791	-1.678	1.00	0.00	C
ATOM	2102	CD1	LEU	B	222	-19.167	13.710	-1.385	1.00	0.00	C
ATOM	2103	CD2	LEU	B	222	-18.512	16.142	-1.078	1.00	0.00	C
ATOM	2104	C	LEU	B	222	-14.286	14.962	-1.349	1.00	0.00	C
ATOM	2105	O	LEU	B	222	-13.904	15.633	-0.392	1.00	0.00	O
ATOM	2106	N	LEU	B	223	-13.532	14.010	-1.903	1.00	0.00	N
ATOM	2107	H	LEU	B	223	-13.893	13.454	-2.653	1.00	0.00	H
ATOM	2108	CA	LEU	B	223	-12.160	13.896	-1.447	1.00	0.00	C
ATOM	2109	CB	LEU	B	223	-11.602	12.501	-1.723	1.00	0.00	C
ATOM	2110	CG	LEU	B	223	-12.332	11.430	-0.911	1.00	0.00	C
ATOM	2111	CD1	LEU	B	223	-11.983	10.023	-1.386	1.00	0.00	C
ATOM	2112	CD2	LEU	B	223	-12.123	11.603	0.595	1.00	0.00	C
ATOM	2113	C	LEU	B	223	-11.261	14.975	-2.003	1.00	0.00	C
ATOM	2114	O	LEU	B	223	-10.575	15.647	-1.240	1.00	0.00	O
ATOM	2115	N	LEU	B	224	-11.287	15.091	-3.336	1.00	0.00	N
ATOM	2116	H	LEU	B	224	-11.989	14.594	-3.851	1.00	0.00	H
ATOM	2117	CA	LEU	B	224	-10.295	15.905	-4.034	1.00	0.00	C
ATOM	2118	CB	LEU	B	224	-10.223	15.504	-5.509	1.00	0.00	C
ATOM	2119	CG	LEU	B	224	-9.838	14.034	-5.715	1.00	0.00	C
ATOM	2120	CD1	LEU	B	224	-10.014	13.602	-7.170	1.00	0.00	C
ATOM	2121	CD2	LEU	B	224	-8.439	13.714	-5.182	1.00	0.00	C
ATOM	2122	C	LEU	B	224	-10.445	17.407	-3.890	1.00	0.00	C
ATOM	2123	O	LEU	B	224	-9.478	18.102	-3.596	1.00	0.00	O
ATOM	2124	N	ASP	B	225	-11.689	17.879	-4.086	1.00	0.00	N
ATOM	2125	H	ASP	B	225	-12.430	17.240	-4.301	1.00	0.00	H
ATOM	2126	CA	ASP	B	225	-12.003	19.306	-3.912	1.00	0.00	C
ATOM	2127	CB	ASP	B	225	-13.520	19.487	-4.133	1.00	0.00	C
ATOM	2128	CG	ASP	B	225	-14.028	20.902	-3.890	1.00	0.00	C
ATOM	2129	OD1	ASP	B	225	-14.686	21.445	-4.770	1.00	0.00	O
ATOM	2130	OD2	ASP	B	225	-13.804	21.445	-2.810	1.00	0.00	O
ATOM	2131	C	ASP	B	225	-11.540	19.790	-2.543	1.00	0.00	C
ATOM	2132	O	ASP	B	225	-10.616	20.582	-2.370	1.00	0.00	O
ATOM	2133	N	ARG	B	226	-12.177	19.152	-1.554	1.00	0.00	N
ATOM	2134	H	ARG	B	226	-13.007	18.650	-1.795	1.00	0.00	H
ATOM	2135	CA	ARG	B	226	-11.824	19.407	-0.166	1.00	0.00	C
ATOM	2136	CB	ARG	B	226	-12.665	18.473	0.688	1.00	0.00	C
ATOM	2137	CG	ARG	B	226	-12.331	18.460	2.175	1.00	0.00	C
ATOM	2138	CD	ARG	B	226	-12.677	17.089	2.738	1.00	0.00	C
ATOM	2139	NE	ARG	B	226	-12.066	16.046	1.920	1.00	0.00	N
ATOM	2140	HE	ARG	B	226	-12.528	15.849	1.045	1.00	0.00	H
ATOM	2141	CZ	ARG	B	226	-10.879	15.506	2.260	1.00	0.00	C
ATOM	2142	NH1	ARG	B	226	-10.335	15.727	3.457	1.00	0.00	N
ATOM	2143	HH11	ARG	B	226	-10.814	16.289	4.143	1.00	0.00	H
ATOM	2144	HH12	ARG	B	226	-9.444	15.358	3.717	1.00	0.00	H

```
ATOM   2145  NH2 ARG B 226     -10.247  14.757   1.369  1.00  0.00           N
ATOM   2146 HH21 ARG B 226     -10.577  14.743   0.420  1.00  0.00           H
ATOM   2147 HH22 ARG B 226      -9.424  14.234   1.590  1.00  0.00           H
ATOM   2148  C   ARG B 226     -10.334  19.322   0.155  1.00  0.00           C
ATOM   2149  O   ARG B 226      -9.808  20.169   0.860  1.00  0.00           O
ATOM   2150  N   LEU B 227      -9.652  18.297  -0.400  1.00  0.00           N
ATOM   2151  H   LEU B 227     -10.122  17.625  -0.974  1.00  0.00           H
ATOM   2152  CA  LEU B 227      -8.190  18.268  -0.219  1.00  0.00           C
ATOM   2153  CB  LEU B 227      -7.545  17.056  -0.893  1.00  0.00           C
ATOM   2154  CG  LEU B 227      -7.628  15.755  -0.097  1.00  0.00           C
ATOM   2155  CD1 LEU B 227      -7.045  14.581  -0.885  1.00  0.00           C
ATOM   2156  CD2 LEU B 227      -7.019  15.876   1.302  1.00  0.00           C
ATOM   2157  C   LEU B 227      -7.491  19.510  -0.738  1.00  0.00           C
ATOM   2158  O   LEU B 227      -6.653  20.131  -0.097  1.00  0.00           O
ATOM   2159  N   ASN B 228      -7.919  19.882  -1.952  1.00  0.00           N
ATOM   2160  H   ASN B 228      -8.704  19.408  -2.357  1.00  0.00           H
ATOM   2161  CA  ASN B 228      -7.356  21.084  -2.560  1.00  0.00           C
ATOM   2162  CB  ASN B 228      -7.954  21.329  -3.947  1.00  0.00           C
ATOM   2163  CG  ASN B 228      -7.134  20.647  -5.025  1.00  0.00           C
ATOM   2164  OD1 ASN B 228      -6.360  21.265  -5.755  1.00  0.00           O
ATOM   2165  ND2 ASN B 228      -7.344  19.333  -5.121  1.00  0.00           N
ATOM   2166 HD21 ASN B 228      -6.870  18.760  -5.785  1.00  0.00           H
ATOM   2167 HD22 ASN B 228      -8.013  18.898  -4.513  1.00  0.00           H
ATOM   2168  C   ASN B 228      -7.488  22.326  -1.697  1.00  0.00           C
ATOM   2169  O   ASN B 228      -6.598  23.167  -1.657  1.00  0.00           O
ATOM   2170  N   GLN B 229      -8.615  22.389  -0.979  1.00  0.00           N
ATOM   2171  H   GLN B 229      -9.316  21.679  -1.085  1.00  0.00           H
ATOM   2172  CA  GLN B 229      -8.751  23.493  -0.030  1.00  0.00           C
ATOM   2173  CB  GLN B 229     -10.226  23.727   0.297  1.00  0.00           C
ATOM   2174  CG  GLN B 229     -11.107  23.846  -0.952  1.00  0.00           C
ATOM   2175  CD  GLN B 229     -12.472  24.352  -0.542  1.00  0.00           C
ATOM   2176  OE1 GLN B 229     -12.608  25.111   0.409  1.00  0.00           O
ATOM   2177  NE2 GLN B 229     -13.479  23.929  -1.304  1.00  0.00           N
ATOM   2178 HE21 GLN B 229     -14.388  24.323  -1.170  1.00  0.00           H
ATOM   2179 HE22 GLN B 229     -13.384  23.225  -2.018  1.00  0.00           H
ATOM   2180  C   GLN B 229      -7.926  23.338   1.246  1.00  0.00           C
ATOM   2181  O   GLN B 229      -7.221  24.233   1.696  1.00  0.00           O
ATOM   2182  N   LEU B 230      -8.036  22.134   1.821  1.00  0.00           N
ATOM   2183  H   LEU B 230      -8.585  21.443   1.356  1.00  0.00           H
ATOM   2184  CA  LEU B 230      -7.390  21.826   3.099  1.00  0.00           C
ATOM   2185  CB  LEU B 230      -7.773  20.389   3.500  1.00  0.00           C
ATOM   2186  CG  LEU B 230      -7.591  19.945   4.963  1.00  0.00           C
ATOM   2187  CD1 LEU B 230      -6.151  19.591   5.339  1.00  0.00           C
ATOM   2188  CD2 LEU B 230      -8.229  20.922   5.948  1.00  0.00           C
ATOM   2189  C   LEU B 230      -5.883  22.042   3.093  1.00  0.00           C
ATOM   2190  O   LEU B 230      -5.288  22.562   4.030  1.00  0.00           O
ATOM   2191  N   GLU B 231      -5.301  21.612   1.971  1.00  0.00           N
ATOM   2192  H   GLU B 231      -5.857  21.198   1.249  1.00  0.00           H
ATOM   2193  CA  GLU B 231      -3.846  21.614   1.868  1.00  0.00           C
ATOM   2194  CB  GLU B 231      -3.381  20.248   1.373  1.00  0.00           C
ATOM   2195  CG  GLU B 231      -4.009  19.041   2.075  1.00  0.00           C
ATOM   2196  CD  GLU B 231      -3.728  17.818   1.232  1.00  0.00           C
ATOM   2197  OE1 GLU B 231      -4.428  17.597   0.246  1.00  0.00           O
ATOM   2198  OE2 GLU B 231      -2.754  17.116   1.487  1.00  0.00           O
ATOM   2199  C   GLU B 231      -3.297  22.685   0.939  1.00  0.00           C
ATOM   2200  O   GLU B 231      -2.211  22.555   0.387  1.00  0.00           O
ATOM   2201  N   SER B 232      -4.115  23.737   0.733  1.00  0.00           N
ATOM   2202  H   SER B 232      -4.975  23.811   1.239  1.00  0.00           H
ATOM   2203  CA  SER B 232      -3.709  24.799  -0.198  1.00  0.00           C
```

```
ATOM   2204  CB  SER B 232      -2.536  25.619   0.351  1.00  0.00           C
ATOM   2205  OG  SER B 232      -2.878  26.224   1.600  1.00  0.00           O
ATOM   2206  HG  SER B 232      -3.797  26.473   1.505  1.00  0.00           H
ATOM   2207  C   SER B 232      -3.391  24.393  -1.636  1.00  0.00           C
ATOM   2208  O   SER B 232      -2.717  25.103  -2.377  1.00  0.00           O
ATOM   2209  N   LYS B 233      -3.900  23.216  -2.022  1.00  0.00           N
ATOM   2210  H   LYS B 233      -4.566  22.761  -1.429  1.00  0.00           H
ATOM   2211  CA  LYS B 233      -3.571  22.742  -3.364  1.00  0.00           C
ATOM   2212  CB  LYS B 233      -3.689  21.218  -3.431  1.00  0.00           C
ATOM   2213  CG  LYS B 233      -2.591  20.543  -2.604  1.00  0.00           C
ATOM   2214  CD  LYS B 233      -2.783  19.037  -2.421  1.00  0.00           C
ATOM   2215  CE  LYS B 233      -1.663  18.426  -1.571  1.00  0.00           C
ATOM   2216  NZ  LYS B 233      -2.119  17.161  -0.982  1.00  0.00           N
ATOM   2217  HZ1 LYS B 233      -3.149  17.208  -0.820  1.00  0.00           H
ATOM   2218  HZ2 LYS B 233      -1.883  16.306  -1.512  1.00  0.00           H
ATOM   2219  HZ3 LYS B 233      -1.788  17.082   0.006  1.00  0.00           H
ATOM   2220  C   LYS B 233      -4.336  23.435  -4.480  1.00  0.00           C
ATOM   2221  O   LYS B 233      -3.922  23.458  -5.636  1.00  0.00           O
ATOM   2222  N   MET B 234      -5.471  24.043  -4.107  1.00  0.00           N
ATOM   2223  H   MET B 234      -5.778  23.995  -3.155  1.00  0.00           H
ATOM   2224  CA  MET B 234      -6.047  24.979  -5.071  1.00  0.00           C
ATOM   2225  CB  MET B 234      -7.547  25.191  -4.872  1.00  0.00           C
ATOM   2226  CG  MET B 234      -8.376  24.387  -5.876  1.00  0.00           C
ATOM   2227  SD  MET B 234      -7.884  24.668  -7.588  1.00  0.00           S
ATOM   2228  CE  MET B 234      -8.392  26.391  -7.734  1.00  0.00           C
ATOM   2229  C   MET B 234      -5.338  26.311  -5.047  1.00  0.00           C
ATOM   2230  O   MET B 234      -4.993  26.888  -6.070  1.00  0.00           O
ATOM   2231  N   SER B 235      -5.094  26.735  -3.805  1.00  0.00           N
ATOM   2232  H   SER B 235      -5.449  26.255  -3.002  1.00  0.00           H
ATOM   2233  CA  SER B 235      -4.424  28.004  -3.547  1.00  0.00           C
ATOM   2234  CB  SER B 235      -4.228  28.146  -2.042  1.00  0.00           C
ATOM   2235  OG  SER B 235      -5.186  27.300  -1.390  1.00  0.00           O
ATOM   2236  HG  SER B 235      -5.591  27.820  -0.691  1.00  0.00           H
ATOM   2237  C   SER B 235      -3.134  28.229  -4.311  1.00  0.00           C
ATOM   2238  O   SER B 235      -2.877  29.305  -4.834  1.00  0.00           O
ATOM   2239  N   GLY B 236      -2.357  27.132  -4.402  1.00  0.00           N
ATOM   2240  H   GLY B 236      -2.602  26.322  -3.867  1.00  0.00           H
ATOM   2241  CA  GLY B 236      -1.178  27.142  -5.269  1.00  0.00           C
ATOM   2242  C   GLY B 236      -1.484  27.621  -6.678  1.00  0.00           C
ATOM   2243  O   GLY B 236      -1.014  28.663  -7.114  1.00  0.00           O
ATOM   2244  N   LYS B 237      -2.355  26.836  -7.337  1.00  0.00           N
ATOM   2245  H   LYS B 237      -2.723  26.045  -6.855  1.00  0.00           H
ATOM   2246  CA  LYS B 237      -2.731  27.162  -8.719  1.00  0.00           C
ATOM   2247  CB  LYS B 237      -3.831  26.237  -9.251  1.00  0.00           C
ATOM   2248  CG  LYS B 237      -3.681  24.747  -8.939  1.00  0.00           C
ATOM   2249  CD  LYS B 237      -4.807  23.934  -9.587  1.00  0.00           C
ATOM   2250  CE  LYS B 237      -4.937  22.508  -9.044  1.00  0.00           C
ATOM   2251  NZ  LYS B 237      -5.332  22.583  -7.634  1.00  0.00           N
ATOM   2252  HZ1 LYS B 237      -5.525  21.651  -7.209  1.00  0.00           H
ATOM   2253  HZ2 LYS B 237      -4.566  23.017  -7.080  1.00  0.00           H
ATOM   2254  HZ3 LYS B 237      -6.189  23.154  -7.516  1.00  0.00           H
ATOM   2255  C   LYS B 237      -3.190  28.598  -8.904  1.00  0.00           C
ATOM   2256  O   LYS B 237      -2.749  29.333  -9.779  1.00  0.00           O
ATOM   2257  N   GLY B 238      -4.102  28.977  -7.995  1.00  0.00           N
ATOM   2258  H   GLY B 238      -4.395  28.327  -7.292  1.00  0.00           H
ATOM   2259  CA  GLY B 238      -4.634  30.335  -8.064  1.00  0.00           C
ATOM   2260  C   GLY B 238      -3.586  31.426  -7.924  1.00  0.00           C
ATOM   2261  O   GLY B 238      -3.628  32.453  -8.588  1.00  0.00           O
ATOM   2262  N   GLN B 239      -2.616  31.162  -7.037  1.00  0.00           N
```

```
ATOM   2263  H   GLN B 239      -2.574  30.290  -6.545  1.00  0.00           H
ATOM   2264  CA  GLN B 239      -1.534  32.141  -6.955  1.00  0.00           C
ATOM   2265  CB  GLN B 239      -0.755  32.014  -5.649  1.00  0.00           C
ATOM   2266  CG  GLN B 239      -1.645  32.310  -4.443  1.00  0.00           C
ATOM   2267  CD  GLN B 239      -0.785  32.492  -3.213  1.00  0.00           C
ATOM   2268  OE1 GLN B 239      -0.658  31.622  -2.357  1.00  0.00           O
ATOM   2269  NE2 GLN B 239      -0.222  33.701  -3.146  1.00  0.00           N
ATOM   2270 HE21 GLN B 239       0.311  33.981  -2.352  1.00  0.00           H
ATOM   2271 HE22 GLN B 239      -0.333  34.360  -3.897  1.00  0.00           H
ATOM   2272  C   GLN B 239      -0.602  32.140  -8.152  1.00  0.00           C
ATOM   2273  O   GLN B 239      -0.114  33.176  -8.578  1.00  0.00           O
ATOM   2274  N   GLN B 240      -0.422  30.941  -8.720  1.00  0.00           N
ATOM   2275  H   GLN B 240      -0.847  30.130  -8.317  1.00  0.00           H
ATOM   2276  CA  GLN B 240       0.349  30.851  -9.962  1.00  0.00           C
ATOM   2277  CB  GLN B 240       0.527  29.383 -10.354  1.00  0.00           C
ATOM   2278  CG  GLN B 240       1.317  28.592  -9.309  1.00  0.00           C
ATOM   2279  CD  GLN B 240       1.193  27.108  -9.584  1.00  0.00           C
ATOM   2280  OE1 GLN B 240       0.433  26.383  -8.956  1.00  0.00           O
ATOM   2281  NE2 GLN B 240       1.993  26.669 -10.560  1.00  0.00           N
ATOM   2282 HE21 GLN B 240       1.967  25.697 -10.782  1.00  0.00           H
ATOM   2283 HE22 GLN B 240       2.600  27.289 -11.054  1.00  0.00           H
ATOM   2284  C   GLN B 240      -0.246  31.653 -11.112  1.00  0.00           C
ATOM   2285  O   GLN B 240       0.452  32.187 -11.961  1.00  0.00           O
ATOM   2286  N   GLN B 241      -1.585  31.731 -11.078  1.00  0.00           N
ATOM   2287  H   GLN B 241      -2.087  31.198 -10.395  1.00  0.00           H
ATOM   2288  CA  GLN B 241      -2.257  32.597 -12.046  1.00  0.00           C
ATOM   2289  CB  GLN B 241      -3.730  32.216 -12.171  1.00  0.00           C
ATOM   2290  CG  GLN B 241      -4.044  30.737 -12.368  1.00  0.00           C
ATOM   2291  CD  GLN B 241      -5.516  30.535 -12.077  1.00  0.00           C
ATOM   2292  OE1 GLN B 241      -6.125  31.214 -11.257  1.00  0.00           O
ATOM   2293  NE2 GLN B 241      -6.082  29.566 -12.799  1.00  0.00           N
ATOM   2294 HE21 GLN B 241      -7.056  29.390 -12.670  1.00  0.00           H
ATOM   2295 HE22 GLN B 241      -5.549  29.037 -13.457  1.00  0.00           H
ATOM   2296  C   GLN B 241      -2.208  34.077 -11.689  1.00  0.00           C
ATOM   2297  O   GLN B 241      -1.995  34.953 -12.515  1.00  0.00           O
ATOM   2298  N   GLN B 242      -2.509  34.318 -10.406  1.00  0.00           N
ATOM   2299  H   GLN B 242      -2.561  33.558  -9.759  1.00  0.00           H
ATOM   2300  CA  GLN B 242      -2.971  35.659 -10.051  1.00  0.00           C
ATOM   2301  CB  GLN B 242      -4.348  35.577  -9.394  1.00  0.00           C
ATOM   2302  CG  GLN B 242      -5.414  34.859 -10.221  1.00  0.00           C
ATOM   2303  CD  GLN B 242      -6.593  34.563  -9.325  1.00  0.00           C
ATOM   2304  OE1 GLN B 242      -6.936  35.335  -8.432  1.00  0.00           O
ATOM   2305  NE2 GLN B 242      -7.193  33.399  -9.582  1.00  0.00           N
ATOM   2306 HE21 GLN B 242      -8.013  33.125  -9.083  1.00  0.00           H
ATOM   2307 HE22 GLN B 242      -6.830  32.771 -10.278  1.00  0.00           H
ATOM   2308  C   GLN B 242      -2.074  36.454  -9.126  1.00  0.00           C
ATOM   2309  O   GLN B 242      -2.393  37.581  -8.759  1.00  0.00           O
ATOM   2310  N   GLY B 243      -0.978  35.813  -8.699  1.00  0.00           N
ATOM   2311  H   GLY B 243      -0.744  34.912  -9.065  1.00  0.00           H
ATOM   2312  CA  GLY B 243      -0.164  36.408  -7.639  1.00  0.00           C
ATOM   2313  C   GLY B 243      -0.866  36.459  -6.288  1.00  0.00           C
ATOM   2314  O   GLY B 243      -0.713  35.606  -5.419  1.00  0.00           O
ATOM   2315  N   GLN B 244      -1.663  37.532  -6.157  1.00  0.00           N
ATOM   2316  H   GLN B 244      -1.784  38.103  -6.971  1.00  0.00           H
ATOM   2317  CA  GLN B 244      -2.398  37.766  -4.919  1.00  0.00           C
ATOM   2318  CB  GLN B 244      -3.213  39.057  -5.007  1.00  0.00           C
ATOM   2319  CG  GLN B 244      -2.387  40.309  -4.695  1.00  0.00           C
ATOM   2320  CD  GLN B 244      -1.924  40.275  -3.248  1.00  0.00           C
ATOM   2321  OE1 GLN B 244      -2.474  39.588  -2.396  1.00  0.00           O
```

ATOM	2322	NE2	GLN	B	244	-0.860	41.044	-3.008	1.00	0.00	N
ATOM	2323	HE21	GLN	B	244	-0.479	41.062	-2.085	1.00	0.00	H
ATOM	2324	HE22	GLN	B	244	-0.441	41.598	-3.726	1.00	0.00	H
ATOM	2325	C	GLN	B	244	-3.274	36.621	-4.459	1.00	0.00	C
ATOM	2326	O	GLN	B	244	-4.104	36.075	-5.187	1.00	0.00	O
ATOM	2327	N	THR	B	245	-3.021	36.302	-3.191	1.00	0.00	N
ATOM	2328	H	THR	B	245	-2.353	36.841	-2.673	1.00	0.00	H
ATOM	2329	CA	THR	B	245	-3.516	35.095	-2.547	1.00	0.00	C
ATOM	2330	CB	THR	B	245	-3.068	35.161	-1.089	1.00	0.00	C
ATOM	2331	OG1	THR	B	245	-1.749	35.723	-1.033	1.00	0.00	O
ATOM	2332	HG1	THR	B	245	-1.462	35.664	-0.131	1.00	0.00	H
ATOM	2333	CG2	THR	B	245	-3.099	33.804	-0.390	1.00	0.00	C
ATOM	2334	C	THR	B	245	-4.996	34.771	-2.686	1.00	0.00	C
ATOM	2335	O	THR	B	245	-5.880	35.389	-2.101	1.00	0.00	O
ATOM	2336	N	VAL	B	246	-5.220	33.720	-3.487	1.00	0.00	N
ATOM	2337	H	VAL	B	246	-4.447	33.280	-3.938	1.00	0.00	H
ATOM	2338	CA	VAL	B	246	-6.542	33.101	-3.413	1.00	0.00	C
ATOM	2339	CB	VAL	B	246	-6.801	32.202	-4.625	1.00	0.00	C
ATOM	2340	CG1	VAL	B	246	-6.795	33.023	-5.914	1.00	0.00	C
ATOM	2341	CG2	VAL	B	246	-5.822	31.033	-4.685	1.00	0.00	C
ATOM	2342	C	VAL	B	246	-6.708	32.355	-2.098	1.00	0.00	C
ATOM	2343	O	VAL	B	246	-5.784	32.290	-1.293	1.00	0.00	O
ATOM	2344	N	THR	B	247	-7.925	31.844	-1.887	1.00	0.00	N
ATOM	2345	H	THR	B	247	-8.560	31.824	-2.662	1.00	0.00	H
ATOM	2346	CA	THR	B	247	-8.399	31.443	-0.555	1.00	0.00	C
ATOM	2347	CB	THR	B	247	-7.616	30.290	0.092	1.00	0.00	C
ATOM	2348	OG1	THR	B	247	-6.623	29.754	-0.793	1.00	0.00	O
ATOM	2349	HG1	THR	B	247	-5.993	30.459	-0.905	1.00	0.00	H
ATOM	2350	CG2	THR	B	247	-8.565	29.196	0.582	1.00	0.00	C
ATOM	2351	C	THR	B	247	-8.619	32.609	0.404	1.00	0.00	C
ATOM	2352	O	THR	B	247	-9.736	32.850	0.839	1.00	0.00	O
ATOM	2353	N	LYS	B	248	-7.545	33.400	0.605	1.00	0.00	N
ATOM	2354	H	LYS	B	248	-6.645	33.051	0.342	1.00	0.00	H
ATOM	2355	CA	LYS	B	248	-7.696	34.731	1.210	1.00	0.00	C
ATOM	2356	CB	LYS	B	248	-6.328	35.433	1.206	1.00	0.00	C
ATOM	2357	CG	LYS	B	248	-6.261	36.915	1.604	1.00	0.00	C
ATOM	2358	CD	LYS	B	248	-4.810	37.411	1.578	1.00	0.00	C
ATOM	2359	CE	LYS	B	248	-4.586	38.926	1.676	1.00	0.00	C
ATOM	2360	NZ	LYS	B	248	-5.070	39.480	2.946	1.00	0.00	N
ATOM	2361	HZ1	LYS	B	248	-6.109	39.517	2.929	1.00	0.00	H
ATOM	2362	HZ2	LYS	B	248	-4.812	38.904	3.775	1.00	0.00	H
ATOM	2363	HZ3	LYS	B	248	-4.718	40.443	3.097	1.00	0.00	H
ATOM	2364	C	LYS	B	248	-8.791	35.559	0.544	1.00	0.00	C
ATOM	2365	O	LYS	B	248	-9.638	36.153	1.190	1.00	0.00	O
ATOM	2366	N	LYS	B	249	-8.763	35.508	-0.799	1.00	0.00	N
ATOM	2367	H	LYS	B	249	-7.947	35.149	-1.254	1.00	0.00	H
ATOM	2368	CA	LYS	B	249	-9.959	35.936	-1.532	1.00	0.00	C
ATOM	2369	CB	LYS	B	249	-9.642	36.068	-3.021	1.00	0.00	C
ATOM	2370	CG	LYS	B	249	-8.520	37.016	-3.427	1.00	0.00	C
ATOM	2371	CD	LYS	B	249	-8.020	36.606	-4.813	1.00	0.00	C
ATOM	2372	CE	LYS	B	249	-6.952	37.523	-5.399	1.00	0.00	C
ATOM	2373	NZ	LYS	B	249	-6.261	36.806	-6.478	1.00	0.00	N
ATOM	2374	HZ1	LYS	B	249	-6.932	36.241	-7.042	1.00	0.00	H
ATOM	2375	HZ2	LYS	B	249	-5.771	37.456	-7.119	1.00	0.00	H
ATOM	2376	HZ3	LYS	B	249	-5.552	36.158	-6.071	1.00	0.00	H
ATOM	2377	C	LYS	B	249	-11.103	34.933	-1.404	1.00	0.00	C
ATOM	2378	O	LYS	B	249	-12.233	35.209	-1.014	1.00	0.00	O
ATOM	2379	N	SER	B	250	-10.739	33.711	-1.802	1.00	0.00	N
ATOM	2380	H	SER	B	250	-9.810	33.540	-2.117	1.00	0.00	H

ATOM	2381	CA	SER	B	250	-11.768	32.753	-2.183	1.00	0.00	C
ATOM	2382	CB	SER	B	250	-11.131	31.688	-3.068	1.00	0.00	C
ATOM	2383	OG	SER	B	250	-10.086	32.332	-3.820	1.00	0.00	O
ATOM	2384	HG	SER	B	250	-10.552	32.923	-4.427	1.00	0.00	H
ATOM	2385	C	SER	B	250	-12.719	32.198	-1.138	1.00	0.00	C
ATOM	2386	O	SER	B	250	-13.717	31.579	-1.475	1.00	0.00	O
ATOM	2387	N	ALA	B	251	-12.437	32.483	0.139	1.00	0.00	N
ATOM	2388	H	ALA	B	251	-11.597	32.967	0.390	1.00	0.00	H
ATOM	2389	CA	ALA	B	251	-13.485	32.183	1.114	1.00	0.00	C
ATOM	2390	CB	ALA	B	251	-12.910	32.171	2.529	1.00	0.00	C
ATOM	2391	C	ALA	B	251	-14.651	33.162	1.016	1.00	0.00	C
ATOM	2392	O	ALA	B	251	-15.830	32.815	1.057	1.00	0.00	O
ATOM	2393	N	ALA	B	252	-14.269	34.431	0.798	1.00	0.00	N
ATOM	2394	H	ALA	B	252	-13.300	34.677	0.718	1.00	0.00	H
ATOM	2395	CA	ALA	B	252	-15.311	35.404	0.469	1.00	0.00	C
ATOM	2396	CB	ALA	B	252	-14.743	36.824	0.445	1.00	0.00	C
ATOM	2397	C	ALA	B	252	-15.964	35.094	-0.867	1.00	0.00	C
ATOM	2398	O	ALA	B	252	-17.161	35.250	-1.077	1.00	0.00	O
ATOM	2399	N	GLU	B	253	-15.123	34.564	-1.764	1.00	0.00	N
ATOM	2400	H	GLU	B	253	-14.143	34.548	-1.553	1.00	0.00	H
ATOM	2401	CA	GLU	B	253	-15.686	34.118	-3.038	1.00	0.00	C
ATOM	2402	CB	GLU	B	253	-14.586	33.883	-4.068	1.00	0.00	C
ATOM	2403	CG	GLU	B	253	-13.640	35.087	-4.082	1.00	0.00	C
ATOM	2404	CD	GLU	B	253	-12.727	35.027	-5.277	1.00	0.00	C
ATOM	2405	OE1	GLU	B	253	-11.686	34.380	-5.195	1.00	0.00	O
ATOM	2406	OE2	GLU	B	253	-13.065	35.650	-6.282	1.00	0.00	O
ATOM	2407	C	GLU	B	253	-16.675	32.964	-2.957	1.00	0.00	C
ATOM	2408	O	GLU	B	253	-17.688	32.932	-3.645	1.00	0.00	O
ATOM	2409	N	ALA	B	254	-16.387	32.056	-2.016	1.00	0.00	N
ATOM	2410	H	ALA	B	254	-15.511	32.093	-1.539	1.00	0.00	H
ATOM	2411	CA	ALA	B	254	-17.368	31.024	-1.683	1.00	0.00	C
ATOM	2412	CB	ALA	B	254	-16.774	30.032	-0.684	1.00	0.00	C
ATOM	2413	C	ALA	B	254	-18.643	31.605	-1.096	1.00	0.00	C
ATOM	2414	O	ALA	B	254	-19.747	31.122	-1.294	1.00	0.00	O
ATOM	2415	N	SER	B	255	-18.443	32.717	-0.386	1.00	0.00	N
ATOM	2416	H	SER	B	255	-17.516	33.050	-0.218	1.00	0.00	H
ATOM	2417	CA	SER	B	255	-19.619	33.426	0.103	1.00	0.00	C
ATOM	2418	CB	SER	B	255	-19.189	34.329	1.256	1.00	0.00	C
ATOM	2419	OG	SER	B	255	-18.397	33.565	2.180	1.00	0.00	O
ATOM	2420	HG	SER	B	255	-17.554	33.381	1.770	1.00	0.00	H
ATOM	2421	C	SER	B	255	-20.434	34.163	-0.964	1.00	0.00	C
ATOM	2422	O	SER	B	255	-21.623	34.430	-0.796	1.00	0.00	O
ATOM	2423	N	LYS	B	256	-19.759	34.455	-2.100	1.00	0.00	N
ATOM	2424	H	LYS	B	256	-18.776	34.271	-2.138	1.00	0.00	H
ATOM	2425	CA	LYS	B	256	-20.466	35.061	-3.234	1.00	0.00	C
ATOM	2426	CB	LYS	B	256	-19.545	35.330	-4.439	1.00	0.00	C
ATOM	2427	CG	LYS	B	256	-18.407	36.345	-4.294	1.00	0.00	C
ATOM	2428	CD	LYS	B	256	-17.472	36.274	-5.514	1.00	0.00	C
ATOM	2429	CE	LYS	B	256	-16.262	37.215	-5.455	1.00	0.00	C
ATOM	2430	NZ	LYS	B	256	-15.252	36.808	-6.449	1.00	0.00	N
ATOM	2431	HZ1	LYS	B	256	-14.911	35.832	-6.286	1.00	0.00	H
ATOM	2432	HZ2	LYS	B	256	-15.580	36.885	-7.428	1.00	0.00	H
ATOM	2433	HZ3	LYS	B	256	-14.361	37.336	-6.338	1.00	0.00	H
ATOM	2434	C	LYS	B	256	-21.664	34.247	-3.709	1.00	0.00	C
ATOM	2435	O	LYS	B	256	-22.808	34.578	-3.412	1.00	0.00	O
ATOM	2436	N	LYS	B	257	-21.358	33.182	-4.471	1.00	0.00	N
ATOM	2437	H	LYS	B	257	-20.410	32.868	-4.526	1.00	0.00	H
ATOM	2438	CA	LYS	B	257	-22.447	32.517	-5.192	1.00	0.00	C
ATOM	2439	CB	LYS	B	257	-21.877	31.686	-6.366	1.00	0.00	C

ATOM	2440	CG	LYS	B	257	-22.786	31.388	-7.571	1.00	0.00	C
ATOM	2441	CD	LYS	B	257	-23.727	30.179	-7.465	1.00	0.00	C
ATOM	2442	CE	LYS	B	257	-24.432	29.920	-8.806	1.00	0.00	C
ATOM	2443	NZ	LYS	B	257	-25.319	28.751	-8.731	1.00	0.00	N
ATOM	2444	HZ1	LYS	B	257	-26.009	28.764	-9.504	1.00	0.00	H
ATOM	2445	HZ2	LYS	B	257	-24.813	27.840	-8.791	1.00	0.00	H
ATOM	2446	HZ3	LYS	B	257	-25.852	28.776	-7.841	1.00	0.00	H
ATOM	2447	C	LYS	B	257	-23.509	31.829	-4.327	1.00	0.00	C
ATOM	2448	O	LYS	B	257	-24.664	32.231	-4.357	1.00	0.00	O
ATOM	2449	N	PRO	B	258	-23.135	30.804	-3.515	1.00	0.00	N
ATOM	2450	CD	PRO	B	258	-21.826	30.173	-3.345	1.00	0.00	C
ATOM	2451	CA	PRO	B	258	-24.180	30.137	-2.726	1.00	0.00	C
ATOM	2452	CB	PRO	B	258	-23.529	28.785	-2.458	1.00	0.00	C
ATOM	2453	CG	PRO	B	258	-22.041	29.067	-2.326	1.00	0.00	C
ATOM	2454	C	PRO	B	258	-24.645	30.846	-1.449	1.00	0.00	C
ATOM	2455	O	PRO	B	258	-24.999	30.206	-0.465	1.00	0.00	O
ATOM	2456	N	ARG	B	259	-24.641	32.192	-1.496	1.00	0.00	N
ATOM	2457	H	ARG	B	259	-24.405	32.617	-2.368	1.00	0.00	H
ATOM	2458	CA	ARG	B	259	-25.231	33.011	-0.428	1.00	0.00	C
ATOM	2459	CB	ARG	B	259	-26.738	33.168	-0.658	1.00	0.00	C
ATOM	2460	CG	ARG	B	259	-27.103	33.547	-2.102	1.00	0.00	C
ATOM	2461	CD	ARG	B	259	-26.807	34.981	-2.570	1.00	0.00	C
ATOM	2462	NE	ARG	B	259	-25.385	35.311	-2.522	1.00	0.00	N
ATOM	2463	HE	ARG	B	259	-24.756	34.767	-3.088	1.00	0.00	H
ATOM	2464	CZ	ARG	B	259	-24.920	36.283	-1.711	1.00	0.00	C
ATOM	2465	NH1	ARG	B	259	-25.754	37.097	-1.065	1.00	0.00	N
ATOM	2466	HH11	ARG	B	259	-26.752	36.995	-1.173	1.00	0.00	H
ATOM	2467	HH12	ARG	B	259	-25.437	37.828	-0.464	1.00	0.00	H
ATOM	2468	NH2	ARG	B	259	-23.609	36.400	-1.554	1.00	0.00	N
ATOM	2469	HH21	ARG	B	259	-23.014	35.724	-2.009	1.00	0.00	H
ATOM	2470	HH22	ARG	B	259	-23.184	37.112	-0.999	1.00	0.00	H
ATOM	2471	C	ARG	B	259	-24.902	32.619	1.005	1.00	0.00	C
ATOM	2472	O	ARG	B	259	-25.747	32.448	1.885	1.00	0.00	O
ATOM	2473	N	GLN	B	260	-23.589	32.474	1.190	1.00	0.00	N
ATOM	2474	H	GLN	B	260	-22.944	32.746	0.475	1.00	0.00	H
ATOM	2475	CA	GLN	B	260	-23.137	31.890	2.444	1.00	0.00	C
ATOM	2476	CB	GLN	B	260	-21.955	30.969	2.155	1.00	0.00	C
ATOM	2477	CG	GLN	B	260	-22.235	29.765	1.251	1.00	0.00	C
ATOM	2478	CD	GLN	B	260	-23.040	28.687	1.957	1.00	0.00	C
ATOM	2479	OE1	GLN	B	260	-23.487	28.815	3.090	1.00	0.00	O
ATOM	2480	NE2	GLN	B	260	-23.182	27.570	1.239	1.00	0.00	N
ATOM	2481	HE21	GLN	B	260	-23.575	26.757	1.656	1.00	0.00	H
ATOM	2482	HE22	GLN	B	260	-22.889	27.498	0.285	1.00	0.00	H
ATOM	2483	C	GLN	B	260	-22.824	32.909	3.530	1.00	0.00	C
ATOM	2484	O	GLN	B	260	-21.680	33.205	3.841	1.00	0.00	O
ATOM	2485	N	LYS	B	261	-23.921	33.427	4.115	1.00	0.00	N
ATOM	2486	H	LYS	B	261	-24.793	33.178	3.696	1.00	0.00	H
ATOM	2487	CA	LYS	B	261	-23.842	34.438	5.183	1.00	0.00	C
ATOM	2488	CB	LYS	B	261	-25.199	34.526	5.895	1.00	0.00	C
ATOM	2489	CG	LYS	B	261	-25.340	35.665	6.917	1.00	0.00	C
ATOM	2490	CD	LYS	B	261	-26.550	35.454	7.832	1.00	0.00	C
ATOM	2491	CE	LYS	B	261	-26.366	34.228	8.736	1.00	0.00	C
ATOM	2492	NZ	LYS	B	261	-27.665	33.706	9.166	1.00	0.00	N
ATOM	2493	HZ1	LYS	B	261	-28.160	34.312	9.853	1.00	0.00	H
ATOM	2494	HZ2	LYS	B	261	-28.296	33.515	8.358	1.00	0.00	H
ATOM	2495	HZ3	LYS	B	261	-27.544	32.815	9.690	1.00	0.00	H
ATOM	2496	C	LYS	B	261	-22.733	34.240	6.217	1.00	0.00	C
ATOM	2497	O	LYS	B	261	-21.826	35.045	6.387	1.00	0.00	O
ATOM	2498	N	ARG	B	262	-22.852	33.103	6.921	1.00	0.00	N

ATOM	2499	H	ARG	B	262	-23.570	32.447	6.703	1.00	0.00	H
ATOM	2500	CA	ARG	B	262	-21.854	32.867	7.962	1.00	0.00	C
ATOM	2501	CB	ARG	B	262	-22.294	31.716	8.868	1.00	0.00	C
ATOM	2502	CG	ARG	B	262	-21.286	31.321	9.948	1.00	0.00	C
ATOM	2503	CD	ARG	B	262	-20.901	32.437	10.923	1.00	0.00	C
ATOM	2504	NE	ARG	B	262	-19.973	31.893	11.906	1.00	0.00	N
ATOM	2505	HE	ARG	B	262	-20.102	30.924	12.129	1.00	0.00	H
ATOM	2506	CZ	ARG	B	262	-19.023	32.590	12.554	1.00	0.00	C
ATOM	2507	NH1	ARG	B	262	-18.788	33.863	12.253	1.00	0.00	N
ATOM	2508	HH11	ARG	B	262	-19.290	34.298	11.494	1.00	0.00	H
ATOM	2509	HH12	ARG	B	262	-18.117	34.417	12.742	1.00	0.00	H
ATOM	2510	NH2	ARG	B	262	-18.317	31.973	13.497	1.00	0.00	N
ATOM	2511	HH21	ARG	B	262	-18.510	31.000	13.698	1.00	0.00	H
ATOM	2512	HH22	ARG	B	262	-17.599	32.421	14.026	1.00	0.00	H
ATOM	2513	C	ARG	B	262	-20.451	32.682	7.414	1.00	0.00	C
ATOM	2514	O	ARG	B	262	-19.486	33.215	7.954	1.00	0.00	O
ATOM	2515	N	THR	B	263	-20.387	31.965	6.286	1.00	0.00	N
ATOM	2516	H	THR	B	263	-21.156	31.463	5.894	1.00	0.00	H
ATOM	2517	CA	THR	B	263	-19.110	31.860	5.591	1.00	0.00	C
ATOM	2518	CB	THR	B	263	-19.312	31.080	4.304	1.00	0.00	C
ATOM	2519	OG1	THR	B	263	-20.164	29.957	4.579	1.00	0.00	O
ATOM	2520	HG1	THR	B	263	-20.294	29.498	3.751	1.00	0.00	H
ATOM	2521	CG2	THR	B	263	-17.998	30.659	3.646	1.00	0.00	C
ATOM	2522	C	THR	B	263	-18.400	33.177	5.341	1.00	0.00	C
ATOM	2523	O	THR	B	263	-17.200	33.262	5.530	1.00	0.00	O
ATOM	2524	N	ALA	B	264	-19.186	34.215	5.008	1.00	0.00	N
ATOM	2525	H	ALA	B	264	-20.146	34.055	4.775	1.00	0.00	H
ATOM	2526	CA	ALA	B	264	-18.572	35.539	4.876	1.00	0.00	C
ATOM	2527	CB	ALA	B	264	-19.615	36.581	4.468	1.00	0.00	C
ATOM	2528	C	ALA	B	264	-17.859	36.020	6.131	1.00	0.00	C
ATOM	2529	O	ALA	B	264	-16.755	36.549	6.107	1.00	0.00	O
ATOM	2530	N	THR	B	265	-18.529	35.778	7.265	1.00	0.00	N
ATOM	2531	H	THR	B	265	-19.412	35.311	7.217	1.00	0.00	H
ATOM	2532	CA	THR	B	265	-17.861	36.196	8.497	1.00	0.00	C
ATOM	2533	CB	THR	B	265	-18.861	36.306	9.651	1.00	0.00	C
ATOM	2534	OG1	THR	B	265	-19.445	35.032	9.953	1.00	0.00	O
ATOM	2535	HG1	THR	B	265	-19.714	34.662	9.114	1.00	0.00	H
ATOM	2536	CG2	THR	B	265	-19.958	37.323	9.331	1.00	0.00	C
ATOM	2537	C	THR	B	265	-16.631	35.374	8.871	1.00	0.00	C
ATOM	2538	O	THR	B	265	-15.642	35.895	9.367	1.00	0.00	O
ATOM	2539	N	LYS	B	266	-16.711	34.062	8.563	1.00	0.00	N
ATOM	2540	H	LYS	B	266	-17.549	33.706	8.148	1.00	0.00	H
ATOM	2541	CA	LYS	B	266	-15.499	33.237	8.687	1.00	0.00	C
ATOM	2542	CB	LYS	B	266	-15.795	31.769	8.329	1.00	0.00	C
ATOM	2543	CG	LYS	B	266	-16.891	31.028	9.115	1.00	0.00	C
ATOM	2544	CD	LYS	B	266	-17.144	29.639	8.503	1.00	0.00	C
ATOM	2545	CE	LYS	B	266	-18.302	28.821	9.097	1.00	0.00	C
ATOM	2546	NZ	LYS	B	266	-17.954	28.189	10.380	1.00	0.00	N
ATOM	2547	HZ1	LYS	B	266	-18.843	27.932	10.868	1.00	0.00	H
ATOM	2548	HZ2	LYS	B	266	-17.363	27.341	10.232	1.00	0.00	H
ATOM	2549	HZ3	LYS	B	266	-17.494	28.856	11.026	1.00	0.00	H
ATOM	2550	C	LYS	B	266	-14.378	33.750	7.780	1.00	0.00	C
ATOM	2551	O	LYS	B	266	-13.202	33.847	8.117	1.00	0.00	O
ATOM	2552	N	ALA	B	267	-14.848	34.103	6.578	1.00	0.00	N
ATOM	2553	H	ALA	B	267	-15.829	34.053	6.418	1.00	0.00	H
ATOM	2554	CA	ALA	B	267	-13.986	34.564	5.503	1.00	0.00	C
ATOM	2555	CB	ALA	B	267	-14.785	34.856	4.236	1.00	0.00	C
ATOM	2556	C	ALA	B	267	-13.220	35.806	5.852	1.00	0.00	C
ATOM	2557	O	ALA	B	267	-12.073	35.944	5.460	1.00	0.00	O

ATOM	2558	N	TYR	B	268	-13.865	36.689	6.631	1.00	0.00	N
ATOM	2559	H	TYR	B	268	-14.839	36.548	6.820	1.00	0.00	H
ATOM	2560	CA	TYR	B	268	-13.171	37.909	7.045	1.00	0.00	C
ATOM	2561	CB	TYR	B	268	-14.046	38.708	8.026	1.00	0.00	C
ATOM	2562	CG	TYR	B	268	-13.346	39.983	8.444	1.00	0.00	C
ATOM	2563	CD1	TYR	B	268	-13.081	40.982	7.483	1.00	0.00	C
ATOM	2564	CE1	TYR	B	268	-12.332	42.108	7.861	1.00	0.00	C
ATOM	2565	CD2	TYR	B	268	-12.948	40.118	9.789	1.00	0.00	C
ATOM	2566	CE2	TYR	B	268	-12.206	41.247	10.167	1.00	0.00	C
ATOM	2567	CZ	TYR	B	268	-11.875	42.207	9.191	1.00	0.00	C
ATOM	2568	OH	TYR	B	268	-11.057	43.264	9.547	1.00	0.00	O
ATOM	2569	HH	TYR	B	268	-11.217	43.995	8.961	1.00	0.00	H
ATOM	2570	C	TYR	B	268	-11.756	37.692	7.576	1.00	0.00	C
ATOM	2571	O	TYR	B	268	-10.799	38.320	7.135	1.00	0.00	O
ATOM	2572	N	ASN	B	269	-11.647	36.740	8.517	1.00	0.00	N
ATOM	2573	H	ASN	B	269	-12.450	36.208	8.797	1.00	0.00	H
ATOM	2574	CA	ASN	B	269	-10.304	36.497	9.059	1.00	0.00	C
ATOM	2575	CB	ASN	B	269	-10.332	35.575	10.278	1.00	0.00	C
ATOM	2576	CG	ASN	B	269	-11.025	36.256	11.440	1.00	0.00	C
ATOM	2577	OD1	ASN	B	269	-12.072	35.830	11.906	1.00	0.00	O
ATOM	2578	ND2	ASN	B	269	-10.399	37.340	11.906	1.00	0.00	N
ATOM	2579	HD21	ASN	B	269	-10.788	37.795	12.702	1.00	0.00	H
ATOM	2580	HD22	ASN	B	269	-9.568	37.717	11.487	1.00	0.00	H
ATOM	2581	C	ASN	B	269	-9.322	35.964	8.032	1.00	0.00	C
ATOM	2582	O	ASN	B	269	-8.169	36.370	7.942	1.00	0.00	O
ATOM	2583	N	VAL	B	270	-9.867	35.063	7.202	1.00	0.00	N
ATOM	2584	H	VAL	B	270	-10.825	34.807	7.342	1.00	0.00	H
ATOM	2585	CA	VAL	B	270	-9.107	34.538	6.062	1.00	0.00	C
ATOM	2586	CB	VAL	B	270	-9.995	33.515	5.331	1.00	0.00	C
ATOM	2587	CG1	VAL	B	270	-9.350	32.930	4.079	1.00	0.00	C
ATOM	2588	CG2	VAL	B	270	-10.442	32.407	6.288	1.00	0.00	C
ATOM	2589	C	VAL	B	270	-8.601	35.638	5.121	1.00	0.00	C
ATOM	2590	O	VAL	B	270	-7.464	35.651	4.653	1.00	0.00	O
ATOM	2591	N	THR	B	271	-9.519	36.591	4.909	1.00	0.00	N
ATOM	2592	H	THR	B	271	-10.355	36.552	5.450	1.00	0.00	H
ATOM	2593	CA	THR	B	271	-9.273	37.742	4.049	1.00	0.00	C
ATOM	2594	CB	THR	B	271	-10.582	38.513	3.814	1.00	0.00	C
ATOM	2595	OG1	THR	B	271	-11.625	37.628	3.388	1.00	0.00	O
ATOM	2596	HG1	THR	B	271	-11.564	36.843	3.920	1.00	0.00	H
ATOM	2597	CG2	THR	B	271	-10.427	39.654	2.803	1.00	0.00	C
ATOM	2598	C	THR	B	271	-8.194	38.644	4.621	1.00	0.00	C
ATOM	2599	O	THR	B	271	-7.376	39.216	3.906	1.00	0.00	O
ATOM	2600	N	GLN	B	272	-8.179	38.706	5.961	1.00	0.00	N
ATOM	2601	H	GLN	B	272	-8.895	38.255	6.498	1.00	0.00	H
ATOM	2602	CA	GLN	B	272	-7.024	39.330	6.598	1.00	0.00	C
ATOM	2603	CB	GLN	B	272	-7.283	39.566	8.092	1.00	0.00	C
ATOM	2604	CG	GLN	B	272	-8.554	40.334	8.454	1.00	0.00	C
ATOM	2605	CD	GLN	B	272	-8.765	40.194	9.949	1.00	0.00	C
ATOM	2606	OE1	GLN	B	272	-8.619	39.124	10.526	1.00	0.00	O
ATOM	2607	NE2	GLN	B	272	-9.082	41.329	10.573	1.00	0.00	N
ATOM	2608	HE21	GLN	B	272	-9.117	41.335	11.570	1.00	0.00	H
ATOM	2609	HE22	GLN	B	272	-9.325	42.170	10.083	1.00	0.00	H
ATOM	2610	C	GLN	B	272	-5.757	38.503	6.397	1.00	0.00	C
ATOM	2611	O	GLN	B	272	-4.966	38.736	5.479	1.00	0.00	O
ATOM	2612	N	ALA	B	273	-5.600	37.526	7.303	1.00	0.00	N
ATOM	2613	H	ALA	B	273	-6.390	37.199	7.828	1.00	0.00	H
ATOM	2614	CA	ALA	B	273	-4.298	36.898	7.498	1.00	0.00	C
ATOM	2615	CB	ALA	B	273	-4.116	36.521	8.971	1.00	0.00	C
ATOM	2616	C	ALA	B	273	-4.036	35.686	6.626	1.00	0.00	C

ATOM	2617	O	ALA	B	273	-3.782	34.585	7.091	1.00	0.00	O
ATOM	2618	N	PHE	B	274	-4.085	35.965	5.313	1.00	0.00	N
ATOM	2619	H	PHE	B	274	-4.359	36.893	5.077	1.00	0.00	H
ATOM	2620	CA	PHE	B	274	-3.581	35.019	4.312	1.00	0.00	C
ATOM	2621	CB	PHE	B	274	-2.048	35.093	4.215	1.00	0.00	C
ATOM	2622	CG	PHE	B	274	-1.598	36.408	3.617	1.00	0.00	C
ATOM	2623	CD1	PHE	B	274	-1.520	37.565	4.425	1.00	0.00	C
ATOM	2624	CD2	PHE	B	274	-1.255	36.451	2.249	1.00	0.00	C
ATOM	2625	CE1	PHE	B	274	-1.100	38.782	3.854	1.00	0.00	C
ATOM	2626	CE2	PHE	B	274	-0.833	37.667	1.676	1.00	0.00	C
ATOM	2627	CZ	PHE	B	274	-0.762	38.820	2.485	1.00	0.00	C
ATOM	2628	C	PHE	B	274	-4.035	33.576	4.460	1.00	0.00	C
ATOM	2629	O	PHE	B	274	-3.263	32.634	4.321	1.00	0.00	O
ATOM	2630	N	GLY	B	275	-5.341	33.440	4.732	1.00	0.00	N
ATOM	2631	H	GLY	B	275	-5.946	34.237	4.787	1.00	0.00	H
ATOM	2632	CA	GLY	B	275	-5.840	32.078	4.895	1.00	0.00	C
ATOM	2633	C	GLY	B	275	-5.851	31.316	3.585	1.00	0.00	C
ATOM	2634	O	GLY	B	275	-6.744	31.448	2.761	1.00	0.00	O
ATOM	2635	N	ARG	B	276	-4.796	30.512	3.415	1.00	0.00	N
ATOM	2636	H	ARG	B	276	-4.056	30.530	4.088	1.00	0.00	H
ATOM	2637	CA	ARG	B	276	-4.755	29.773	2.157	1.00	0.00	C
ATOM	2638	CB	ARG	B	276	-3.345	29.633	1.584	1.00	0.00	C
ATOM	2639	CG	ARG	B	276	-2.403	30.826	1.723	1.00	0.00	C
ATOM	2640	CD	ARG	B	276	-1.152	30.657	0.856	1.00	0.00	C
ATOM	2641	NE	ARG	B	276	-0.582	29.317	1.000	1.00	0.00	N
ATOM	2642	HE	ARG	B	276	-0.644	28.890	1.904	1.00	0.00	H
ATOM	2643	CZ	ARG	B	276	-0.010	28.697	-0.055	1.00	0.00	C
ATOM	2644	NH1	ARG	B	276	0.090	29.307	-1.236	1.00	0.00	N
ATOM	2645	HH11	ARG	B	276	-0.260	30.245	-1.373	1.00	0.00	H
ATOM	2646	HH12	ARG	B	276	0.512	28.876	-2.031	1.00	0.00	H
ATOM	2647	NH2	ARG	B	276	0.452	27.458	0.100	1.00	0.00	N
ATOM	2648	HH21	ARG	B	276	0.345	26.985	0.978	1.00	0.00	H
ATOM	2649	HH22	ARG	B	276	0.906	26.965	-0.643	1.00	0.00	H
ATOM	2650	C	ARG	B	276	-5.364	28.388	2.199	1.00	0.00	C
ATOM	2651	O	ARG	B	276	-5.237	27.627	1.248	1.00	0.00	O
ATOM	2652	N	ARG	B	277	-5.967	28.057	3.347	1.00	0.00	N
ATOM	2653	H	ARG	B	277	-6.164	28.702	4.084	1.00	0.00	H
ATOM	2654	CA	ARG	B	277	-6.334	26.656	3.512	1.00	0.00	C
ATOM	2655	CB	ARG	B	277	-5.130	25.834	3.983	1.00	0.00	C
ATOM	2656	CG	ARG	B	277	-4.468	26.356	5.262	1.00	0.00	C
ATOM	2657	CD	ARG	B	277	-3.450	25.363	5.820	1.00	0.00	C
ATOM	2658	NE	ARG	B	277	-4.119	24.115	6.182	1.00	0.00	N
ATOM	2659	HE	ARG	B	277	-4.567	23.581	5.458	1.00	0.00	H
ATOM	2660	CZ	ARG	B	277	-4.210	23.702	7.464	1.00	0.00	C
ATOM	2661	NH1	ARG	B	277	-3.646	24.381	8.455	1.00	0.00	N
ATOM	2662	HH11	ARG	B	277	-3.117	25.209	8.303	1.00	0.00	H
ATOM	2663	HH12	ARG	B	277	-3.773	24.046	9.408	1.00	0.00	H
ATOM	2664	NH2	ARG	B	277	-4.875	22.596	7.751	1.00	0.00	N
ATOM	2665	HH21	ARG	B	277	-5.404	22.112	7.055	1.00	0.00	H
ATOM	2666	HH22	ARG	B	277	-4.840	22.261	8.703	1.00	0.00	H
ATOM	2667	C	ARG	B	277	-7.481	26.467	4.472	1.00	0.00	C
ATOM	2668	O	ARG	B	277	-7.783	27.339	5.278	1.00	0.00	O
ATOM	2669	N	GLY	B	278	-8.078	25.272	4.365	1.00	0.00	N
ATOM	2670	H	GLY	B	278	-7.739	24.635	3.672	1.00	0.00	H
ATOM	2671	CA	GLY	B	278	-9.015	24.869	5.411	1.00	0.00	C
ATOM	2672	C	GLY	B	278	-8.281	24.482	6.686	1.00	0.00	C
ATOM	2673	O	GLY	B	278	-7.184	23.931	6.651	1.00	0.00	O
ATOM	2674	N	PRO	B	279	-8.922	24.819	7.826	1.00	0.00	N
ATOM	2675	CD	PRO	B	279	-10.236	25.444	7.936	1.00	0.00	C

ATOM	2676	CA	PRO	B	279	-8.282	24.586	9.124	1.00	0.00	C
ATOM	2677	CB	PRO	B	279	-9.117	25.483	10.042	1.00	0.00	C
ATOM	2678	CG	PRO	B	279	-10.519	25.442	9.435	1.00	0.00	C
ATOM	2679	C	PRO	B	279	-8.319	23.123	9.536	1.00	0.00	C
ATOM	2680	O	PRO	B	279	-9.065	22.302	9.014	1.00	0.00	O
ATOM	2681	N	GLU	B	280	-7.481	22.835	10.533	1.00	0.00	N
ATOM	2682	H	GLU	B	280	-6.868	23.555	10.863	1.00	0.00	H
ATOM	2683	CA	GLU	B	280	-7.483	21.487	11.098	1.00	0.00	C
ATOM	2684	CB	GLU	B	280	-6.208	21.140	11.894	1.00	0.00	C
ATOM	2685	CG	GLU	B	280	-5.261	22.274	12.326	1.00	0.00	C
ATOM	2686	CD	GLU	B	280	-4.663	22.959	11.108	1.00	0.00	C
ATOM	2687	OE1	GLU	B	280	-4.931	24.137	10.897	1.00	0.00	O
ATOM	2688	OE2	GLU	B	280	-3.991	22.312	10.315	1.00	0.00	O
ATOM	2689	C	GLU	B	280	-8.723	21.197	11.915	1.00	0.00	C
ATOM	2690	O	GLU	B	280	-9.256	22.032	12.640	1.00	0.00	O
ATOM	2691	N	GLN	B	281	-9.193	19.964	11.709	1.00	0.00	N
ATOM	2692	H	GLN	B	281	-8.613	19.296	11.245	1.00	0.00	H
ATOM	2693	CA	GLN	B	281	-10.551	19.632	12.133	1.00	0.00	C
ATOM	2694	CB	GLN	B	281	-11.090	18.515	11.243	1.00	0.00	C
ATOM	2695	CG	GLN	B	281	-11.001	18.829	9.749	1.00	0.00	C
ATOM	2696	CD	GLN	B	281	-12.058	19.836	9.352	1.00	0.00	C
ATOM	2697	OE1	GLN	B	281	-13.201	19.493	9.079	1.00	0.00	O
ATOM	2698	NE2	GLN	B	281	-11.624	21.089	9.257	1.00	0.00	N
ATOM	2699	HE21	GLN	B	281	-12.292	21.781	8.983	1.00	0.00	H
ATOM	2700	HE22	GLN	B	281	-10.678	21.380	9.413	1.00	0.00	H
ATOM	2701	C	GLN	B	281	-10.689	19.247	13.591	1.00	0.00	C
ATOM	2702	O	GLN	B	281	-10.898	18.089	13.930	1.00	0.00	O
ATOM	2703	N	THR	B	282	-10.587	20.267	14.453	1.00	0.00	N
ATOM	2704	H	THR	B	282	-10.419	21.202	14.132	1.00	0.00	H
ATOM	2705	CA	THR	B	282	-10.685	19.966	15.882	1.00	0.00	C
ATOM	2706	CB	THR	B	282	-10.456	21.238	16.688	1.00	0.00	C
ATOM	2707	OG1	THR	B	282	-11.210	22.326	16.116	1.00	0.00	O
ATOM	2708	HG1	THR	B	282	-10.640	23.101	16.151	1.00	0.00	H
ATOM	2709	CG2	THR	B	282	-8.962	21.553	16.746	1.00	0.00	C
ATOM	2710	C	THR	B	282	-11.977	19.287	16.296	1.00	0.00	C
ATOM	2711	O	THR	B	282	-11.991	18.310	17.029	1.00	0.00	O
ATOM	2712	N	GLN	B	283	-13.074	19.831	15.747	1.00	0.00	N
ATOM	2713	H	GLN	B	283	-12.946	20.636	15.170	1.00	0.00	H
ATOM	2714	CA	GLN	B	283	-14.378	19.263	16.100	1.00	0.00	C
ATOM	2715	CB	GLN	B	283	-15.516	20.121	15.534	1.00	0.00	C
ATOM	2716	CG	GLN	B	283	-15.350	21.635	15.739	1.00	0.00	C
ATOM	2717	CD	GLN	B	283	-15.237	21.977	17.211	1.00	0.00	C
ATOM	2718	OE1	GLN	B	283	-16.194	21.918	17.974	1.00	0.00	O
ATOM	2719	NE2	GLN	B	283	-14.007	22.344	17.590	1.00	0.00	N
ATOM	2720	HE21	GLN	B	283	-13.852	22.515	18.559	1.00	0.00	H
ATOM	2721	HE22	GLN	B	283	-13.243	22.429	16.944	1.00	0.00	H
ATOM	2722	C	GLN	B	283	-14.532	17.797	15.716	1.00	0.00	C
ATOM	2723	O	GLN	B	283	-14.901	16.942	16.510	1.00	0.00	O
ATOM	2724	N	GLY	B	284	-14.156	17.525	14.455	1.00	0.00	N
ATOM	2725	H	GLY	B	284	-13.870	18.253	13.838	1.00	0.00	H
ATOM	2726	CA	GLY	B	284	-14.145	16.127	14.018	1.00	0.00	C
ATOM	2727	C	GLY	B	284	-13.291	15.223	14.895	1.00	0.00	C
ATOM	2728	O	GLY	B	284	-13.677	14.128	15.285	1.00	0.00	O
ATOM	2729	N	ASN	B	285	-12.112	15.767	15.231	1.00	0.00	N
ATOM	2730	H	ASN	B	285	-11.859	16.663	14.864	1.00	0.00	H
ATOM	2731	CA	ASN	B	285	-11.230	15.044	16.147	1.00	0.00	C
ATOM	2732	CB	ASN	B	285	-9.904	15.795	16.295	1.00	0.00	C
ATOM	2733	CG	ASN	B	285	-8.877	14.921	16.984	1.00	0.00	C
ATOM	2734	OD1	ASN	B	285	-8.654	14.998	18.182	1.00	0.00	O

```
ATOM   2735  ND2 ASN B 285      -8.236  14.083  16.165  1.00  0.00           N
ATOM   2736 HD21 ASN B 285      -7.535  13.487  16.551  1.00  0.00           H
ATOM   2737 HD22 ASN B 285      -8.446  14.040  15.190  1.00  0.00           H
ATOM   2738  C   ASN B 285     -11.853  14.719  17.500  1.00  0.00           C
ATOM   2739  O   ASN B 285     -11.715  13.618  18.019  1.00  0.00           O
ATOM   2740  N   PHE B 286     -12.598  15.708  18.024  1.00  0.00           N
ATOM   2741  H   PHE B 286     -12.672  16.590  17.557  1.00  0.00           H
ATOM   2742  CA  PHE B 286     -13.325  15.452  19.269  1.00  0.00           C
ATOM   2743  CB  PHE B 286     -14.022  16.710  19.806  1.00  0.00           C
ATOM   2744  CG  PHE B 286     -13.070  17.869  20.022  1.00  0.00           C
ATOM   2745  CD1 PHE B 286     -11.786  17.661  20.579  1.00  0.00           C
ATOM   2746  CD2 PHE B 286     -13.504  19.163  19.663  1.00  0.00           C
ATOM   2747  CE1 PHE B 286     -10.925  18.760  20.767  1.00  0.00           C
ATOM   2748  CE2 PHE B 286     -12.646  20.263  19.853  1.00  0.00           C
ATOM   2749  CZ  PHE B 286     -11.363  20.050  20.399  1.00  0.00           C
ATOM   2750  C   PHE B 286     -14.343  14.336  19.138  1.00  0.00           C
ATOM   2751  O   PHE B 286     -14.454  13.460  19.986  1.00  0.00           O
ATOM   2752  N   GLY B 287     -15.057  14.383  18.003  1.00  0.00           N
ATOM   2753  H   GLY B 287     -14.925  15.154  17.376  1.00  0.00           H
ATOM   2754  CA  GLY B 287     -15.999  13.304  17.701  1.00  0.00           C
ATOM   2755  C   GLY B 287     -15.373  11.918  17.683  1.00  0.00           C
ATOM   2756  O   GLY B 287     -15.911  10.948  18.205  1.00  0.00           O
ATOM   2757  N   ASP B 288     -14.187  11.879  17.064  1.00  0.00           N
ATOM   2758  H   ASP B 288     -13.803  12.702  16.643  1.00  0.00           H
ATOM   2759  CA  ASP B 288     -13.538  10.580  16.953  1.00  0.00           C
ATOM   2760  CB  ASP B 288     -12.556  10.560  15.778  1.00  0.00           C
ATOM   2761  CG  ASP B 288     -12.360   9.117  15.363  1.00  0.00           C
ATOM   2762  OD1 ASP B 288     -11.287   8.565  15.584  1.00  0.00           O
ATOM   2763  OD2 ASP B 288     -13.322   8.486  14.933  1.00  0.00           O
ATOM   2764  C   ASP B 288     -12.898  10.042  18.224  1.00  0.00           C
ATOM   2765  O   ASP B 288     -12.898   8.837  18.483  1.00  0.00           O
ATOM   2766  N   GLN B 289     -12.350  10.990  18.998  1.00  0.00           N
ATOM   2767  H   GLN B 289     -12.406  11.949  18.719  1.00  0.00           H
ATOM   2768  CA  GLN B 289     -11.724  10.588  20.254  1.00  0.00           C
ATOM   2769  CB  GLN B 289     -10.544  11.478  20.629  1.00  0.00           C
ATOM   2770  CG  GLN B 289      -9.233  11.305  19.877  1.00  0.00           C
ATOM   2771  CD  GLN B 289      -8.181  12.033  20.690  1.00  0.00           C
ATOM   2772  OE1 GLN B 289      -7.603  11.506  21.638  1.00  0.00           O
ATOM   2773  NE2 GLN B 289      -7.974  13.292  20.312  1.00  0.00           N
ATOM   2774 HE21 GLN B 289      -7.334  13.888  20.788  1.00  0.00           H
ATOM   2775 HE22 GLN B 289      -8.492  13.703  19.558  1.00  0.00           H
ATOM   2776  C   GLN B 289     -12.656  10.570  21.447  1.00  0.00           C
ATOM   2777  O   GLN B 289     -12.863   9.544  22.076  1.00  0.00           O
ATOM   2778  N   GLU B 290     -13.162  11.769  21.767  1.00  0.00           N
ATOM   2779  H   GLU B 290     -13.095  12.541  21.136  1.00  0.00           H
ATOM   2780  CA  GLU B 290     -13.768  11.906  23.090  1.00  0.00           C
ATOM   2781  CB  GLU B 290     -13.448  13.264  23.735  1.00  0.00           C
ATOM   2782  CG  GLU B 290     -12.194  14.035  23.275  1.00  0.00           C
ATOM   2783  CD  GLU B 290     -10.854  13.368  23.581  1.00  0.00           C
ATOM   2784  OE1 GLU B 290     -10.757  12.462  24.409  1.00  0.00           O
ATOM   2785  OE2 GLU B 290      -9.865  13.774  22.980  1.00  0.00           O
ATOM   2786  C   GLU B 290     -15.269  11.666  23.125  1.00  0.00           C
ATOM   2787  O   GLU B 290     -15.841  11.206  24.107  1.00  0.00           O
ATOM   2788  N   LEU B 291     -15.912  12.001  21.995  1.00  0.00           N
ATOM   2789  H   LEU B 291     -15.385  12.300  21.199  1.00  0.00           H
ATOM   2790  CA  LEU B 291     -17.363  11.806  21.928  1.00  0.00           C
ATOM   2791  CB  LEU B 291     -18.008  12.822  20.983  1.00  0.00           C
ATOM   2792  CG  LEU B 291     -17.790  14.279  21.400  1.00  0.00           C
ATOM   2793  CD1 LEU B 291     -18.349  15.246  20.357  1.00  0.00           C
```

ATOM	2794	CD2	LEU	B	291	-18.342	14.574	22.796	1.00	0.00	C
ATOM	2795	C	LEU	B	291	-17.744	10.391	21.537	1.00	0.00	C
ATOM	2796	O	LEU	B	291	-18.354	10.122	20.508	1.00	0.00	O
ATOM	2797	N	ILE	B	292	-17.312	9.484	22.415	1.00	0.00	N
ATOM	2798	H	ILE	B	292	-16.913	9.771	23.288	1.00	0.00	H
ATOM	2799	CA	ILE	B	292	-17.192	8.121	21.939	1.00	0.00	C
ATOM	2800	CB	ILE	B	292	-15.685	7.808	21.855	1.00	0.00	C
ATOM	2801	CG2	ILE	B	292	-15.008	7.612	23.218	1.00	0.00	C
ATOM	2802	CG1	ILE	B	292	-15.389	6.720	20.833	1.00	0.00	C
ATOM	2803	CD1	ILE	B	292	-13.890	6.563	20.567	1.00	0.00	C
ATOM	2804	C	ILE	B	292	-18.042	7.115	22.706	1.00	0.00	C
ATOM	2805	O	ILE	B	292	-17.769	6.702	23.829	1.00	0.00	O
ATOM	2806	N	ARG	B	293	-19.146	6.745	22.046	1.00	0.00	N
ATOM	2807	H	ARG	B	293	-19.417	7.240	21.219	1.00	0.00	H
ATOM	2808	CA	ARG	B	293	-20.009	5.791	22.743	1.00	0.00	C
ATOM	2809	CB	ARG	B	293	-21.399	6.384	22.970	1.00	0.00	C
ATOM	2810	CG	ARG	B	293	-21.336	7.564	23.940	1.00	0.00	C
ATOM	2811	CD	ARG	B	293	-22.695	8.197	24.225	1.00	0.00	C
ATOM	2812	NE	ARG	B	293	-22.562	9.221	25.262	1.00	0.00	N
ATOM	2813	HE	ARG	B	293	-22.112	10.073	24.986	1.00	0.00	H
ATOM	2814	CZ	ARG	B	293	-22.944	8.972	26.535	1.00	0.00	C
ATOM	2815	NH1	ARG	B	293	-23.516	7.809	26.854	1.00	0.00	N
ATOM	2816	HH11	ARG	B	293	-23.660	7.112	26.148	1.00	0.00	H
ATOM	2817	HH12	ARG	B	293	-23.811	7.595	27.786	1.00	0.00	H
ATOM	2818	NH2	ARG	B	293	-22.744	9.900	27.469	1.00	0.00	N
ATOM	2819	HH21	ARG	B	293	-22.305	10.769	27.236	1.00	0.00	H
ATOM	2820	HH22	ARG	B	293	-23.018	9.758	28.422	1.00	0.00	H
ATOM	2821	C	ARG	B	293	-20.104	4.389	22.171	1.00	0.00	C
ATOM	2822	O	ARG	B	293	-20.720	3.510	22.759	1.00	0.00	O
ATOM	2823	N	GLN	B	294	-19.485	4.207	20.996	1.00	0.00	N
ATOM	2824	H	GLN	B	294	-19.055	4.981	20.532	1.00	0.00	H
ATOM	2825	CA	GLN	B	294	-19.429	2.856	20.434	1.00	0.00	C
ATOM	2826	CB	GLN	B	294	-20.462	2.669	19.316	1.00	0.00	C
ATOM	2827	CG	GLN	B	294	-21.884	2.398	19.820	1.00	0.00	C
ATOM	2828	CD	GLN	B	294	-21.957	1.022	20.461	1.00	0.00	C
ATOM	2829	OE1	GLN	B	294	-22.160	0.013	19.802	1.00	0.00	O
ATOM	2830	NE2	GLN	B	294	-21.789	1.015	21.785	1.00	0.00	N
ATOM	2831	HE21	GLN	B	294	-21.842	0.143	22.266	1.00	0.00	H
ATOM	2832	HE22	GLN	B	294	-21.590	1.853	22.298	1.00	0.00	H
ATOM	2833	C	GLN	B	294	-18.042	2.513	19.942	1.00	0.00	C
ATOM	2834	O	GLN	B	294	-17.466	1.474	20.240	1.00	0.00	O
ATOM	2835	N	GLY	B	295	-17.491	3.495	19.208	1.00	0.00	N
ATOM	2836	H	GLY	B	295	-18.070	4.253	18.918	1.00	0.00	H
ATOM	2837	CA	GLY	B	295	-16.101	3.420	18.761	1.00	0.00	C
ATOM	2838	C	GLY	B	295	-15.096	3.094	19.854	1.00	0.00	C
ATOM	2839	O	GLY	B	295	-14.054	2.508	19.606	1.00	0.00	O
ATOM	2840	N	THR	B	296	-15.485	3.480	21.075	1.00	0.00	N
ATOM	2841	H	THR	B	296	-16.330	3.995	21.204	1.00	0.00	H
ATOM	2842	CA	THR	B	296	-14.736	3.198	22.299	1.00	0.00	C
ATOM	2843	CB	THR	B	296	-15.680	3.493	23.467	1.00	0.00	C
ATOM	2844	OG1	THR	B	296	-16.675	4.446	23.040	1.00	0.00	O
ATOM	2845	HG1	THR	B	296	-16.909	4.965	23.810	1.00	0.00	H
ATOM	2846	CG2	THR	B	296	-14.933	3.976	24.711	1.00	0.00	C
ATOM	2847	C	THR	B	296	-14.189	1.785	22.368	1.00	0.00	C
ATOM	2848	O	THR	B	296	-13.020	1.528	22.623	1.00	0.00	O
ATOM	2849	N	ASP	B	297	-15.117	0.884	22.045	1.00	0.00	N
ATOM	2850	H	ASP	B	297	-16.068	1.149	21.890	1.00	0.00	H
ATOM	2851	CA	ASP	B	297	-14.718	-0.510	22.018	1.00	0.00	C
ATOM	2852	CB	ASP	B	297	-15.724	-1.321	22.823	1.00	0.00	C

ATOM	2853	CG	ASP	B	297	-15.540	-1.021	24.296	1.00	0.00	C
ATOM	2854	OD1	ASP	B	297	-14.809	-1.756	24.957	1.00	0.00	O
ATOM	2855	OD2	ASP	B	297	-16.096	-0.034	24.775	1.00	0.00	O
ATOM	2856	C	ASP	B	297	-14.528	-1.021	20.607	1.00	0.00	C
ATOM	2857	O	ASP	B	297	-13.614	-1.778	20.311	1.00	0.00	O
ATOM	2858	N	TYR	B	298	-15.393	-0.519	19.704	1.00	0.00	N
ATOM	2859	H	TYR	B	298	-16.139	0.058	20.043	1.00	0.00	H
ATOM	2860	CA	TYR	B	298	-15.287	-0.890	18.286	1.00	0.00	C
ATOM	2861	CB	TYR	B	298	-16.303	-0.080	17.457	1.00	0.00	C
ATOM	2862	CG	TYR	B	298	-16.188	-0.359	15.971	1.00	0.00	C
ATOM	2863	CD1	TYR	B	298	-16.822	-1.493	15.424	1.00	0.00	C
ATOM	2864	CE1	TYR	B	298	-16.664	-1.767	14.054	1.00	0.00	C
ATOM	2865	CD2	TYR	B	298	-15.430	0.527	15.176	1.00	0.00	C
ATOM	2866	CE2	TYR	B	298	-15.263	0.247	13.811	1.00	0.00	C
ATOM	2867	CZ	TYR	B	298	-15.871	-0.904	13.271	1.00	0.00	C
ATOM	2868	OH	TYR	B	298	-15.667	-1.195	11.933	1.00	0.00	O
ATOM	2869	HH	TYR	B	298	-16.521	-1.337	11.530	1.00	0.00	H
ATOM	2870	C	TYR	B	298	-13.881	-0.812	17.693	1.00	0.00	C
ATOM	2871	O	TYR	B	298	-13.397	-1.727	17.041	1.00	0.00	O
ATOM	2872	N	LYS	B	299	-13.245	0.340	17.950	1.00	0.00	N
ATOM	2873	H	LYS	B	299	-13.658	0.998	18.578	1.00	0.00	H
ATOM	2874	CA	LYS	B	299	-11.884	0.523	17.442	1.00	0.00	C
ATOM	2875	CB	LYS	B	299	-11.448	1.989	17.573	1.00	0.00	C
ATOM	2876	CG	LYS	B	299	-12.396	3.004	16.921	1.00	0.00	C
ATOM	2877	CD	LYS	B	299	-12.021	4.463	17.218	1.00	0.00	C
ATOM	2878	CE	LYS	B	299	-13.073	5.444	16.689	1.00	0.00	C
ATOM	2879	NZ	LYS	B	299	-12.670	6.838	16.902	1.00	0.00	N
ATOM	2880	HZ1	LYS	B	299	-12.668	7.192	17.881	1.00	0.00	H
ATOM	2881	HZ2	LYS	B	299	-11.757	7.081	16.458	1.00	0.00	H
ATOM	2882	HZ3	LYS	B	299	-13.306	7.467	16.368	1.00	0.00	H
ATOM	2883	C	LYS	B	299	-10.872	-0.374	18.138	1.00	0.00	C
ATOM	2884	O	LYS	B	299	-9.856	-0.772	17.583	1.00	0.00	O
ATOM	2885	N	HIS	B	300	-11.205	-0.656	19.404	1.00	0.00	N
ATOM	2886	H	HIS	B	300	-12.103	-0.422	19.773	1.00	0.00	H
ATOM	2887	CA	HIS	B	300	-10.258	-1.377	20.240	1.00	0.00	C
ATOM	2888	CB	HIS	B	300	-10.245	-0.788	21.657	1.00	0.00	C
ATOM	2889	CG	HIS	B	300	-9.652	0.606	21.642	1.00	0.00	C
ATOM	2890	ND1	HIS	B	300	-8.390	0.883	22.019	1.00	0.00	N
ATOM	2891	HD1	HIS	B	300	-7.720	0.255	22.366	1.00	0.00	H
ATOM	2892	CD2	HIS	B	300	-10.265	1.802	21.249	1.00	0.00	C
ATOM	2893	NE2	HIS	B	300	-9.354	2.796	21.393	1.00	0.00	N
ATOM	2894	CE1	HIS	B	300	-8.202	2.230	21.868	1.00	0.00	C
ATOM	2895	C	HIS	B	300	-10.438	-2.885	20.271	1.00	0.00	C
ATOM	2896	O	HIS	B	300	-9.640	-3.587	20.876	1.00	0.00	O
ATOM	2897	N	TRP	B	301	-11.481	-3.382	19.578	1.00	0.00	N
ATOM	2898	H	TRP	B	301	-12.173	-2.763	19.205	1.00	0.00	H
ATOM	2899	CA	TRP	B	301	-11.571	-4.838	19.413	1.00	0.00	C
ATOM	2900	CB	TRP	B	301	-12.834	-5.273	18.661	1.00	0.00	C
ATOM	2901	CG	TRP	B	301	-14.139	-4.866	19.303	1.00	0.00	C
ATOM	2902	CD2	TRP	B	301	-14.548	-4.923	20.686	1.00	0.00	C
ATOM	2903	CE2	TRP	B	301	-15.914	-4.481	20.739	1.00	0.00	C
ATOM	2904	CE3	TRP	B	301	-13.887	-5.305	21.872	1.00	0.00	C
ATOM	2905	CD1	TRP	B	301	-15.265	-4.395	18.612	1.00	0.00	C
ATOM	2906	NE1	TRP	B	301	-16.311	-4.167	19.446	1.00	0.00	N
ATOM	2907	HE1	TRP	B	301	-17.195	-3.829	19.188	1.00	0.00	H
ATOM	2908	CZ2	TRP	B	301	-16.594	-4.438	21.973	1.00	0.00	C
ATOM	2909	CZ3	TRP	B	301	-14.578	-5.253	23.099	1.00	0.00	C
ATOM	2910	CH2	TRP	B	301	-15.920	-4.821	23.149	1.00	0.00	C
ATOM	2911	C	TRP	B	301	-10.414	-5.364	18.586	1.00	0.00	C

ATOM	2912	O	TRP	B	301	-10.338	-5.093	17.393	1.00	0.00	O
ATOM	2913	N	PRO	B	302	-9.506	-6.144	19.214	1.00	0.00	N
ATOM	2914	CD	PRO	B	302	-9.537	-6.634	20.587	1.00	0.00	C
ATOM	2915	CA	PRO	B	302	-8.293	-6.517	18.483	1.00	0.00	C
ATOM	2916	CB	PRO	B	302	-7.470	-7.302	19.511	1.00	0.00	C
ATOM	2917	CG	PRO	B	302	-8.423	-7.671	20.645	1.00	0.00	C
ATOM	2918	C	PRO	B	302	-8.507	-7.207	17.140	1.00	0.00	C
ATOM	2919	O	PRO	B	302	-7.776	-6.958	16.191	1.00	0.00	O
ATOM	2920	N	GLN	B	303	-9.555	-8.051	17.058	1.00	0.00	N
ATOM	2921	H	GLN	B	303	-10.142	-8.211	17.853	1.00	0.00	H
ATOM	2922	CA	GLN	B	303	-9.866	-8.583	15.726	1.00	0.00	C
ATOM	2923	CB	GLN	B	303	-10.947	-9.665	15.767	1.00	0.00	C
ATOM	2924	CG	GLN	B	303	-10.381	-11.076	15.952	1.00	0.00	C
ATOM	2925	CD	GLN	B	303	-11.499	-12.100	15.874	1.00	0.00	C
ATOM	2926	OE1	GLN	B	303	-12.558	-11.868	15.313	1.00	0.00	O
ATOM	2927	NE2	GLN	B	303	-11.235	-13.265	16.473	1.00	0.00	N
ATOM	2928	HE21	GLN	B	303	-11.984	-13.929	16.491	1.00	0.00	H
ATOM	2929	HE22	GLN	B	303	-10.348	-13.521	16.862	1.00	0.00	H
ATOM	2930	C	GLN	B	303	-10.237	-7.521	14.704	1.00	0.00	C
ATOM	2931	O	GLN	B	303	-9.721	-7.492	13.597	1.00	0.00	O
ATOM	2932	N	ILE	B	304	-11.131	-6.619	15.133	1.00	0.00	N
ATOM	2933	H	ILE	B	304	-11.401	-6.604	16.094	1.00	0.00	H
ATOM	2934	CA	ILE	B	304	-11.556	-5.555	14.219	1.00	0.00	C
ATOM	2935	CB	ILE	B	304	-12.778	-4.841	14.822	1.00	0.00	C
ATOM	2936	CG2	ILE	B	304	-13.092	-3.469	14.217	1.00	0.00	C
ATOM	2937	CG1	ILE	B	304	-13.964	-5.808	14.707	1.00	0.00	C
ATOM	2938	CD1	ILE	B	304	-15.304	-5.231	15.164	1.00	0.00	C
ATOM	2939	C	ILE	B	304	-10.432	-4.619	13.785	1.00	0.00	C
ATOM	2940	O	ILE	B	304	-10.338	-4.197	12.636	1.00	0.00	O
ATOM	2941	N	ALA	B	305	-9.534	-4.372	14.748	1.00	0.00	N
ATOM	2942	H	ALA	B	305	-9.719	-4.678	15.681	1.00	0.00	H
ATOM	2943	CA	ALA	B	305	-8.305	-3.657	14.417	1.00	0.00	C
ATOM	2944	CB	ALA	B	305	-7.502	-3.370	15.687	1.00	0.00	C
ATOM	2945	C	ALA	B	305	-7.423	-4.405	13.426	1.00	0.00	C
ATOM	2946	O	ALA	B	305	-6.830	-3.835	12.520	1.00	0.00	O
ATOM	2947	N	GLN	B	306	-7.377	-5.732	13.623	1.00	0.00	N
ATOM	2948	H	GLN	B	306	-7.893	-6.155	14.369	1.00	0.00	H
ATOM	2949	CA	GLN	B	306	-6.652	-6.543	12.646	1.00	0.00	C
ATOM	2950	CB	GLN	B	306	-6.277	-7.907	13.230	1.00	0.00	C
ATOM	2951	CG	GLN	B	306	-5.335	-7.850	14.440	1.00	0.00	C
ATOM	2952	CD	GLN	B	306	-3.943	-7.397	14.040	1.00	0.00	C
ATOM	2953	OE1	GLN	B	306	-3.079	-8.199	13.698	1.00	0.00	O
ATOM	2954	NE2	GLN	B	306	-3.744	-6.079	14.130	1.00	0.00	N
ATOM	2955	HE21	GLN	B	306	-2.852	-5.686	13.916	1.00	0.00	H
ATOM	2956	HE22	GLN	B	306	-4.485	-5.470	14.413	1.00	0.00	H
ATOM	2957	C	GLN	B	306	-7.334	-6.688	11.294	1.00	0.00	C
ATOM	2958	O	GLN	B	306	-6.695	-6.986	10.291	1.00	0.00	O
ATOM	2959	N	PHE	B	307	-8.648	-6.419	11.291	1.00	0.00	N
ATOM	2960	H	PHE	B	307	-9.132	-6.261	12.151	1.00	0.00	H
ATOM	2961	CA	PHE	B	307	-9.322	-6.253	10.007	1.00	0.00	C
ATOM	2962	CB	PHE	B	307	-10.835	-6.525	10.088	1.00	0.00	C
ATOM	2963	CG	PHE	B	307	-11.209	-7.808	10.803	1.00	0.00	C
ATOM	2964	CD1	PHE	B	307	-12.311	-7.777	11.686	1.00	0.00	C
ATOM	2965	CD2	PHE	B	307	-10.492	-9.005	10.586	1.00	0.00	C
ATOM	2966	CE1	PHE	B	307	-12.696	-8.949	12.366	1.00	0.00	C
ATOM	2967	CE2	PHE	B	307	-10.874	-10.178	11.267	1.00	0.00	C
ATOM	2968	CZ	PHE	B	307	-11.970	-10.138	12.152	1.00	0.00	C
ATOM	2969	C	PHE	B	307	-9.092	-4.856	9.453	1.00	0.00	C
ATOM	2970	O	PHE	B	307	-9.964	-3.991	9.460	1.00	0.00	O

ATOM	2971	N	ALA	B	308	-7.856	-4.671	8.982	1.00	0.00	N
ATOM	2972	H	ALA	B	308	-7.183	-5.413	9.051	1.00	0.00	H
ATOM	2973	CA	ALA	B	308	-7.462	-3.408	8.368	1.00	0.00	C
ATOM	2974	CB	ALA	B	308	-6.929	-2.441	9.434	1.00	0.00	C
ATOM	2975	C	ALA	B	308	-6.362	-3.721	7.375	1.00	0.00	C
ATOM	2976	O	ALA	B	308	-5.580	-4.636	7.603	1.00	0.00	O
ATOM	2977	N	PRO	B	309	-6.306	-2.958	6.256	1.00	0.00	N
ATOM	2978	CD	PRO	B	309	-7.151	-1.823	5.890	1.00	0.00	C
ATOM	2979	CA	PRO	B	309	-5.290	-3.271	5.241	1.00	0.00	C
ATOM	2980	CB	PRO	B	309	-5.644	-2.311	4.099	1.00	0.00	C
ATOM	2981	CG	PRO	B	309	-6.394	-1.152	4.753	1.00	0.00	C
ATOM	2982	C	PRO	B	309	-3.861	-3.147	5.758	1.00	0.00	C
ATOM	2983	O	PRO	B	309	-2.974	-3.921	5.430	1.00	0.00	O
ATOM	2984	N	SER	B	310	-3.697	-2.149	6.634	1.00	0.00	N
ATOM	2985	H	SER	B	310	-4.398	-1.457	6.794	1.00	0.00	H
ATOM	2986	CA	SER	B	310	-2.398	-1.933	7.257	1.00	0.00	C
ATOM	2987	CB	SER	B	310	-2.330	-0.469	7.697	1.00	0.00	C
ATOM	2988	OG	SER	B	310	-3.307	0.297	6.963	1.00	0.00	O
ATOM	2989	HG	SER	B	310	-2.940	1.169	6.858	1.00	0.00	H
ATOM	2990	C	SER	B	310	-2.047	-2.872	8.408	1.00	0.00	C
ATOM	2991	O	SER	B	310	-0.992	-2.772	9.022	1.00	0.00	O
ATOM	2992	N	ALA	B	311	-3.005	-3.762	8.714	1.00	0.00	N
ATOM	2993	H	ALA	B	311	-3.760	-3.945	8.087	1.00	0.00	H
ATOM	2994	CA	ALA	B	311	-2.779	-4.644	9.850	1.00	0.00	C
ATOM	2995	CB	ALA	B	311	-4.090	-4.866	10.605	1.00	0.00	C
ATOM	2996	C	ALA	B	311	-2.114	-5.938	9.405	1.00	0.00	C
ATOM	2997	O	ALA	B	311	-1.121	-5.892	8.688	1.00	0.00	O
ATOM	2998	N	SER	B	312	-2.642	-7.096	9.840	1.00	0.00	N
ATOM	2999	H	SER	B	312	-3.465	-7.206	10.393	1.00	0.00	H
ATOM	3000	CA	SER	B	312	-1.846	-8.230	9.404	1.00	0.00	C
ATOM	3001	CB	SER	B	312	-0.898	-8.660	10.539	1.00	0.00	C
ATOM	3002	OG	SER	B	312	-1.473	-9.567	11.487	1.00	0.00	O
ATOM	3003	HG	SER	B	312	-0.702	-9.991	11.855	1.00	0.00	H
ATOM	3004	C	SER	B	312	-2.564	-9.313	8.608	1.00	0.00	C
ATOM	3005	O	SER	B	312	-3.010	-9.074	7.493	1.00	0.00	O
ATOM	3006	N	ALA	B	313	-2.658	-10.522	9.190	1.00	0.00	N
ATOM	3007	H	ALA	B	313	-2.365	-10.629	10.138	1.00	0.00	H
ATOM	3008	CA	ALA	B	313	-3.053	-11.690	8.395	1.00	0.00	C
ATOM	3009	CB	ALA	B	313	-3.097	-12.937	9.279	1.00	0.00	C
ATOM	3010	C	ALA	B	313	-4.366	-11.578	7.637	1.00	0.00	C
ATOM	3011	O	ALA	B	313	-4.533	-12.105	6.546	1.00	0.00	O
ATOM	3012	N	PHE	B	314	-5.297	-10.851	8.275	1.00	0.00	N
ATOM	3013	H	PHE	B	314	-5.075	-10.374	9.121	1.00	0.00	H
ATOM	3014	CA	PHE	B	314	-6.616	-10.727	7.658	1.00	0.00	C
ATOM	3015	CB	PHE	B	314	-7.617	-10.129	8.648	1.00	0.00	C
ATOM	3016	CG	PHE	B	314	-7.748	-11.021	9.864	1.00	0.00	C
ATOM	3017	CD1	PHE	B	314	-7.178	-10.607	11.087	1.00	0.00	C
ATOM	3018	CD2	PHE	B	314	-8.443	-12.247	9.763	1.00	0.00	C
ATOM	3019	CE1	PHE	B	314	-7.315	-11.421	12.228	1.00	0.00	C
ATOM	3020	CE2	PHE	B	314	-8.581	-13.063	10.903	1.00	0.00	C
ATOM	3021	CZ	PHE	B	314	-8.020	-12.639	12.126	1.00	0.00	C
ATOM	3022	C	PHE	B	314	-6.635	-9.971	6.341	1.00	0.00	C
ATOM	3023	O	PHE	B	314	-7.484	-10.188	5.485	1.00	0.00	O
ATOM	3024	N	PHE	B	315	-5.646	-9.076	6.189	1.00	0.00	N
ATOM	3025	H	PHE	B	315	-4.909	-8.974	6.857	1.00	0.00	H
ATOM	3026	CA	PHE	B	315	-5.522	-8.514	4.851	1.00	0.00	C
ATOM	3027	CB	PHE	B	315	-4.811	-7.155	4.875	1.00	0.00	C
ATOM	3028	CG	PHE	B	315	-4.848	-6.509	3.504	1.00	0.00	C
ATOM	3029	CD1	PHE	B	315	-6.087	-6.293	2.859	1.00	0.00	C

```
ATOM   3030  CD2 PHE B 315      -3.634  -6.132   2.890  1.00  0.00           C
ATOM   3031  CE1 PHE B 315      -6.115  -5.696   1.585  1.00  0.00           C
ATOM   3032  CE2 PHE B 315      -3.661  -5.531   1.616  1.00  0.00           C
ATOM   3033  CZ  PHE B 315      -4.900  -5.319   0.976  1.00  0.00           C
ATOM   3034  C   PHE B 315      -4.843  -9.488   3.912  1.00  0.00           C
ATOM   3035  O   PHE B 315      -3.628  -9.664   3.916  1.00  0.00           O
ATOM   3036  N   GLY B 316      -5.703 -10.120   3.107  1.00  0.00           N
ATOM   3037  H   GLY B 316      -6.676  -9.879   3.164  1.00  0.00           H
ATOM   3038  CA  GLY B 316      -5.182 -11.075   2.139  1.00  0.00           C
ATOM   3039  C   GLY B 316      -4.510 -10.398   0.963  1.00  0.00           C
ATOM   3040  O   GLY B 316      -3.542  -9.658   1.092  1.00  0.00           O
ATOM   3041  N   MET B 317      -5.086 -10.699  -0.205  1.00  0.00           N
ATOM   3042  H   MET B 317      -5.899 -11.274  -0.220  1.00  0.00           H
ATOM   3043  CA  MET B 317      -4.657  -9.925  -1.364  1.00  0.00           C
ATOM   3044  CB  MET B 317      -4.581 -10.810  -2.614  1.00  0.00           C
ATOM   3045  CG  MET B 317      -3.588 -11.972  -2.551  1.00  0.00           C
ATOM   3046  SD  MET B 317      -1.876 -11.437  -2.406  1.00  0.00           S
ATOM   3047  CE  MET B 317      -1.091 -12.999  -2.831  1.00  0.00           C
ATOM   3048  C   MET B 317      -5.613  -8.768  -1.587  1.00  0.00           C
ATOM   3049  O   MET B 317      -5.290  -7.600  -1.440  1.00  0.00           O
ATOM   3050  N   SER B 318      -6.835  -9.181  -1.941  1.00  0.00           N
ATOM   3051  H   SER B 318      -7.069 -10.145  -2.059  1.00  0.00           H
ATOM   3052  CA  SER B 318      -7.863  -8.216  -2.307  1.00  0.00           C
ATOM   3053  CB  SER B 318      -8.705  -8.890  -3.390  1.00  0.00           C
ATOM   3054  OG  SER B 318      -8.828 -10.289  -3.068  1.00  0.00           O
ATOM   3055  HG  SER B 318      -8.473 -10.769  -3.822  1.00  0.00           H
ATOM   3056  C   SER B 318      -8.717  -7.753  -1.136  1.00  0.00           C
ATOM   3057  O   SER B 318      -9.016  -6.581  -0.952  1.00  0.00           O
ATOM   3058  N   ARG B 319      -9.130  -8.763  -0.359  1.00  0.00           N
ATOM   3059  H   ARG B 319      -8.813  -9.689  -0.548  1.00  0.00           H
ATOM   3060  CA  ARG B 319     -10.036  -8.463   0.744  1.00  0.00           C
ATOM   3061  CB  ARG B 319     -11.213  -9.436   0.731  1.00  0.00           C
ATOM   3062  CG  ARG B 319     -12.188  -9.200  -0.420  1.00  0.00           C
ATOM   3063  CD  ARG B 319     -13.343 -10.204  -0.394  1.00  0.00           C
ATOM   3064  NE  ARG B 319     -14.397  -9.815  -1.331  1.00  0.00           N
ATOM   3065  HE  ARG B 319     -14.227  -9.983  -2.303  1.00  0.00           H
ATOM   3066  CZ  ARG B 319     -15.500  -9.181  -0.870  1.00  0.00           C
ATOM   3067  NH1 ARG B 319     -15.666  -9.001   0.436  1.00  0.00           N
ATOM   3068 HH11 ARG B 319     -15.058  -9.412   1.129  1.00  0.00           H
ATOM   3069 HH12 ARG B 319     -16.408  -8.461   0.837  1.00  0.00           H
ATOM   3070  NH2 ARG B 319     -16.412  -8.741  -1.740  1.00  0.00           N
ATOM   3071 HH21 ARG B 319     -16.287  -8.884  -2.719  1.00  0.00           H
ATOM   3072 HH22 ARG B 319     -17.253  -8.258  -1.462  1.00  0.00           H
ATOM   3073  C   ARG B 319      -9.362  -8.498   2.097  1.00  0.00           C
ATOM   3074  O   ARG B 319      -8.332  -9.138   2.290  1.00  0.00           O
ATOM   3075  N   ILE B 320     -10.023  -7.800   3.028  1.00  0.00           N
ATOM   3076  H   ILE B 320     -10.861  -7.295   2.808  1.00  0.00           H
ATOM   3077  CA  ILE B 320      -9.609  -7.901   4.422  1.00  0.00           C
ATOM   3078  CB  ILE B 320      -9.587  -6.529   5.099  1.00  0.00           C
ATOM   3079  CG2 ILE B 320      -8.644  -6.575   6.297  1.00  0.00           C
ATOM   3080  CG1 ILE B 320      -9.291  -5.371   4.143  1.00  0.00           C
ATOM   3081  CD1 ILE B 320      -9.818  -4.044   4.685  1.00  0.00           C
ATOM   3082  C   ILE B 320     -10.592  -8.784   5.160  1.00  0.00           C
ATOM   3083  O   ILE B 320     -11.633  -8.322   5.607  1.00  0.00           O
ATOM   3084  N   GLY B 321     -10.253 -10.076   5.224  1.00  0.00           N
ATOM   3085  H   GLY B 321      -9.325 -10.340   4.958  1.00  0.00           H
ATOM   3086  CA  GLY B 321     -11.216 -11.048   5.743  1.00  0.00           C
ATOM   3087  C   GLY B 321     -11.690 -10.805   7.168  1.00  0.00           C
ATOM   3088  O   GLY B 321     -11.027 -11.138   8.141  1.00  0.00           O
```

ATOM	3089	N	MET	B	322	-12.891	-10.220	7.235	1.00	0.00	N
ATOM	3090	H	MET	B	322	-13.375	-10.017	6.382	1.00	0.00	H
ATOM	3091	CA	MET	B	322	-13.531	-10.033	8.535	1.00	0.00	C
ATOM	3092	CB	MET	B	322	-14.546	-8.890	8.400	1.00	0.00	C
ATOM	3093	CG	MET	B	322	-15.312	-8.466	9.659	1.00	0.00	C
ATOM	3094	SD	MET	B	322	-16.573	-9.634	10.198	1.00	0.00	S
ATOM	3095	CE	MET	B	322	-17.634	-9.550	8.746	1.00	0.00	C
ATOM	3096	C	MET	B	322	-14.159	-11.331	9.016	1.00	0.00	C
ATOM	3097	O	MET	B	322	-14.141	-11.714	10.180	1.00	0.00	O
ATOM	3098	N	GLU	B	323	-14.685	-12.028	8.006	1.00	0.00	N
ATOM	3099	H	GLU	B	323	-14.609	-11.634	7.091	1.00	0.00	H
ATOM	3100	CA	GLU	B	323	-15.476	-13.244	8.152	1.00	0.00	C
ATOM	3101	CB	GLU	B	323	-16.111	-13.573	6.789	1.00	0.00	C
ATOM	3102	CG	GLU	B	323	-17.007	-12.495	6.134	1.00	0.00	C
ATOM	3103	CD	GLU	B	323	-16.274	-11.401	5.346	1.00	0.00	C
ATOM	3104	OE1	GLU	B	323	-15.050	-11.409	5.227	1.00	0.00	O
ATOM	3105	OE2	GLU	B	323	-16.949	-10.525	4.814	1.00	0.00	O
ATOM	3106	C	GLU	B	323	-14.766	-14.482	8.708	1.00	0.00	C
ATOM	3107	O	GLU	B	323	-15.284	-15.593	8.648	1.00	0.00	O
ATOM	3108	N	VAL	B	324	-13.539	-14.283	9.217	1.00	0.00	N
ATOM	3109	H	VAL	B	324	-13.230	-13.358	9.437	1.00	0.00	H
ATOM	3110	CA	VAL	B	324	-12.718	-15.458	9.495	1.00	0.00	C
ATOM	3111	CB	VAL	B	324	-11.232	-15.147	9.234	1.00	0.00	C
ATOM	3112	CG1	VAL	B	324	-10.332	-16.383	9.360	1.00	0.00	C
ATOM	3113	CG2	VAL	B	324	-11.056	-14.502	7.858	1.00	0.00	C
ATOM	3114	C	VAL	B	324	-12.926	-16.104	10.859	1.00	0.00	C
ATOM	3115	O	VAL	B	324	-12.903	-17.324	10.994	1.00	0.00	O
ATOM	3116	N	THR	B	325	-13.114	-15.254	11.877	1.00	0.00	N
ATOM	3117	H	THR	B	325	-13.136	-14.261	11.752	1.00	0.00	H
ATOM	3118	CA	THR	B	325	-12.908	-15.823	13.212	1.00	0.00	C
ATOM	3119	CB	THR	B	325	-11.653	-15.165	13.789	1.00	0.00	C
ATOM	3120	OG1	THR	B	325	-11.604	-13.784	13.392	1.00	0.00	O
ATOM	3121	HG1	THR	B	325	-12.244	-13.314	13.928	1.00	0.00	H
ATOM	3122	CG2	THR	B	325	-10.369	-15.878	13.362	1.00	0.00	C
ATOM	3123	C	THR	B	325	-14.047	-15.782	14.237	1.00	0.00	C
ATOM	3124	O	THR	B	325	-13.930	-15.112	15.257	1.00	0.00	O
ATOM	3125	N	PRO	B	326	-15.153	-16.529	13.978	1.00	0.00	N
ATOM	3126	CD	PRO	B	326	-15.461	-17.347	12.807	1.00	0.00	C
ATOM	3127	CA	PRO	B	326	-16.233	-16.540	14.973	1.00	0.00	C
ATOM	3128	CB	PRO	B	326	-17.421	-17.012	14.129	1.00	0.00	C
ATOM	3129	CG	PRO	B	326	-16.819	-17.973	13.103	1.00	0.00	C
ATOM	3130	C	PRO	B	326	-15.940	-17.465	16.149	1.00	0.00	C
ATOM	3131	O	PRO	B	326	-15.500	-18.595	15.978	1.00	0.00	O
ATOM	3132	N	SER	B	327	-16.236	-16.943	17.345	1.00	0.00	N
ATOM	3133	H	SER	B	327	-16.455	-15.975	17.464	1.00	0.00	H
ATOM	3134	CA	SER	B	327	-16.192	-17.768	18.555	1.00	0.00	C
ATOM	3135	CB	SER	B	327	-16.672	-16.898	19.708	1.00	0.00	C
ATOM	3136	OG	SER	B	327	-16.023	-15.623	19.619	1.00	0.00	O
ATOM	3137	HG	SER	B	327	-15.310	-15.680	20.255	1.00	0.00	H
ATOM	3138	C	SER	B	327	-17.020	-19.044	18.485	1.00	0.00	C
ATOM	3139	O	SER	B	327	-17.941	-19.154	17.679	1.00	0.00	O
ATOM	3140	N	GLY	B	328	-16.677	-19.998	19.364	1.00	0.00	N
ATOM	3141	H	GLY	B	328	-15.889	-19.895	19.982	1.00	0.00	H
ATOM	3142	CA	GLY	B	328	-17.469	-21.224	19.442	1.00	0.00	C
ATOM	3143	C	GLY	B	328	-17.234	-22.201	18.305	1.00	0.00	C
ATOM	3144	O	GLY	B	328	-16.547	-23.208	18.414	1.00	0.00	O
ATOM	3145	N	THR	B	329	-17.870	-21.866	17.180	1.00	0.00	N
ATOM	3146	H	THR	B	329	-18.319	-20.973	17.102	1.00	0.00	H
ATOM	3147	CA	THR	B	329	-17.770	-22.767	16.039	1.00	0.00	C

```
ATOM   3148  CB  THR B 329     -18.916 -22.458  15.080  1.00  0.00           C
ATOM   3149  OG1 THR B 329     -18.880 -21.072  14.713  1.00  0.00           O
ATOM   3150  HG1 THR B 329     -19.758 -20.842  14.431  1.00  0.00           H
ATOM   3151  CG2 THR B 329     -20.276 -22.813  15.685  1.00  0.00           C
ATOM   3152  C   THR B 329     -16.432 -22.696  15.320  1.00  0.00           C
ATOM   3153  O   THR B 329     -15.921 -23.672  14.779  1.00  0.00           O
ATOM   3154  N   TRP B 330     -15.896 -21.459  15.307  1.00  0.00           N
ATOM   3155  H   TRP B 330     -16.346 -20.712  15.800  1.00  0.00           H
ATOM   3156  CA  TRP B 330     -14.653 -21.176  14.588  1.00  0.00           C
ATOM   3157  CB  TRP B 330     -13.427 -21.706  15.351  1.00  0.00           C
ATOM   3158  CG  TRP B 330     -13.326 -21.002  16.682  1.00  0.00           C
ATOM   3159  CD2 TRP B 330     -12.796 -19.688  16.946  1.00  0.00           C
ATOM   3160  CE2 TRP B 330     -12.953 -19.439  18.350  1.00  0.00           C
ATOM   3161  CE3 TRP B 330     -12.220 -18.704  16.114  1.00  0.00           C
ATOM   3162  CD1 TRP B 330     -13.768 -21.476  17.926  1.00  0.00           C
ATOM   3163  NE1 TRP B 330     -13.555 -20.562  18.909  1.00  0.00           N
ATOM   3164  HE1 TRP B 330     -13.852 -20.659  19.849  1.00  0.00           H
ATOM   3165  CZ2 TRP B 330     -12.532 -18.209  18.893  1.00  0.00           C
ATOM   3166  CZ3 TRP B 330     -11.804 -17.478  16.670  1.00  0.00           C
ATOM   3167  CH2 TRP B 330     -11.961 -17.232  18.051  1.00  0.00           C
ATOM   3168  C   TRP B 330     -14.635 -21.631  13.144  1.00  0.00           C
ATOM   3169  O   TRP B 330     -13.606 -22.050  12.628  1.00  0.00           O
ATOM   3170  N   LEU B 331     -15.824 -21.551  12.514  1.00  0.00           N
ATOM   3171  H   LEU B 331     -16.608 -21.190  13.021  1.00  0.00           H
ATOM   3172  CA  LEU B 331     -15.977 -22.208  11.211  1.00  0.00           C
ATOM   3173  CB  LEU B 331     -17.377 -21.973  10.641  1.00  0.00           C
ATOM   3174  CG  LEU B 331     -18.491 -22.607  11.475  1.00  0.00           C
ATOM   3175  CD1 LEU B 331     -19.871 -22.171  10.985  1.00  0.00           C
ATOM   3176  CD2 LEU B 331     -18.364 -24.131  11.556  1.00  0.00           C
ATOM   3177  C   LEU B 331     -14.912 -21.853  10.188  1.00  0.00           C
ATOM   3178  O   LEU B 331     -14.059 -22.661   9.835  1.00  0.00           O
ATOM   3179  N   THR B 332     -14.971 -20.584   9.765  1.00  0.00           N
ATOM   3180  H   THR B 332     -15.689 -19.943  10.042  1.00  0.00           H
ATOM   3181  CA  THR B 332     -14.029 -20.126   8.748  1.00  0.00           C
ATOM   3182  CB  THR B 332     -14.309 -18.655   8.462  1.00  0.00           C
ATOM   3183  OG1 THR B 332     -15.679 -18.347   8.769  1.00  0.00           O
ATOM   3184  HG1 THR B 332     -15.765 -17.403   8.629  1.00  0.00           H
ATOM   3185  CG2 THR B 332     -13.948 -18.271   7.026  1.00  0.00           C
ATOM   3186  C   THR B 332     -12.564 -20.356   9.095  1.00  0.00           C
ATOM   3187  O   THR B 332     -11.778 -20.888   8.319  1.00  0.00           O
ATOM   3188  N   TYR B 333     -12.249 -19.994  10.349  1.00  0.00           N
ATOM   3189  H   TYR B 333     -12.883 -19.400  10.847  1.00  0.00           H
ATOM   3190  CA  TYR B 333     -10.899 -20.262  10.841  1.00  0.00           C
ATOM   3191  CB  TYR B 333     -10.723 -19.708  12.265  1.00  0.00           C
ATOM   3192  CG  TYR B 333      -9.259 -19.545  12.637  1.00  0.00           C
ATOM   3193  CD1 TYR B 333      -8.329 -19.101  11.670  1.00  0.00           C
ATOM   3194  CE1 TYR B 333      -6.973 -18.998  12.016  1.00  0.00           C
ATOM   3195  CD2 TYR B 333      -8.863 -19.840  13.958  1.00  0.00           C
ATOM   3196  CE2 TYR B 333      -7.504 -19.730  14.307  1.00  0.00           C
ATOM   3197  CZ  TYR B 333      -6.572 -19.334  13.323  1.00  0.00           C
ATOM   3198  OH  TYR B 333      -5.223 -19.285  13.624  1.00  0.00           O
ATOM   3199  HH  TYR B 333      -5.082 -19.778  14.435  1.00  0.00           H
ATOM   3200  C   TYR B 333     -10.456 -21.716  10.739  1.00  0.00           C
ATOM   3201  O   TYR B 333      -9.339 -22.021  10.353  1.00  0.00           O
ATOM   3202  N   THR B 334     -11.390 -22.619  11.069  1.00  0.00           N
ATOM   3203  H   THR B 334     -12.329 -22.307  11.218  1.00  0.00           H
ATOM   3204  CA  THR B 334     -11.051 -24.033  10.918  1.00  0.00           C
ATOM   3205  CB  THR B 334     -12.078 -24.915  11.643  1.00  0.00           C
ATOM   3206  OG1 THR B 334     -12.260 -24.443  12.992  1.00  0.00           O
```

ATOM	3207	HG1	THR	B	334	-12.724	-23.607	12.893	1.00	0.00	H
ATOM	3208	CG2	THR	B	334	-11.677	-26.395	11.661	1.00	0.00	C
ATOM	3209	C	THR	B	334	-10.843	-24.472	9.470	1.00	0.00	C
ATOM	3210	O	THR	B	334	-9.977	-25.285	9.173	1.00	0.00	O
ATOM	3211	N	GLY	B	335	-11.643	-23.861	8.576	1.00	0.00	N
ATOM	3212	H	GLY	B	335	-12.365	-23.227	8.863	1.00	0.00	H
ATOM	3213	CA	GLY	B	335	-11.403	-24.112	7.153	1.00	0.00	C
ATOM	3214	C	GLY	B	335	-10.007	-23.709	6.702	1.00	0.00	C
ATOM	3215	O	GLY	B	335	-9.328	-24.408	5.961	1.00	0.00	O
ATOM	3216	N	ALA	B	336	-9.586	-22.549	7.231	1.00	0.00	N
ATOM	3217	H	ALA	B	336	-10.204	-21.997	7.795	1.00	0.00	H
ATOM	3218	CA	ALA	B	336	-8.199	-22.159	6.988	1.00	0.00	C
ATOM	3219	CB	ALA	B	336	-7.924	-20.764	7.556	1.00	0.00	C
ATOM	3220	C	ALA	B	336	-7.184	-23.145	7.549	1.00	0.00	C
ATOM	3221	O	ALA	B	336	-6.254	-23.557	6.878	1.00	0.00	O
ATOM	3222	N	ILE	B	337	-7.429	-23.533	8.805	1.00	0.00	N
ATOM	3223	H	ILE	B	337	-8.247	-23.165	9.243	1.00	0.00	H
ATOM	3224	CA	ILE	B	337	-6.417	-24.216	9.611	1.00	0.00	C
ATOM	3225	CB	ILE	B	337	-7.039	-24.491	10.985	1.00	0.00	C
ATOM	3226	CG2	ILE	B	337	-6.614	-25.769	11.711	1.00	0.00	C
ATOM	3227	CG1	ILE	B	337	-6.792	-23.225	11.798	1.00	0.00	C
ATOM	3228	CD1	ILE	B	337	-7.639	-23.155	13.057	1.00	0.00	C
ATOM	3229	C	ILE	B	337	-5.588	-25.359	9.028	1.00	0.00	C
ATOM	3230	O	ILE	B	337	-4.365	-25.344	9.100	1.00	0.00	O
ATOM	3231	N	LYS	B	338	-6.253	-26.360	8.427	1.00	0.00	N
ATOM	3232	H	LYS	B	338	-7.252	-26.355	8.352	1.00	0.00	H
ATOM	3233	CA	LYS	B	338	-5.312	-27.278	7.772	1.00	0.00	C
ATOM	3234	CB	LYS	B	338	-5.267	-28.705	8.349	1.00	0.00	C
ATOM	3235	CG	LYS	B	338	-3.848	-29.272	8.142	1.00	0.00	C
ATOM	3236	CD	LYS	B	338	-3.571	-30.656	8.722	1.00	0.00	C
ATOM	3237	CE	LYS	B	338	-2.442	-31.410	8.005	1.00	0.00	C
ATOM	3238	NZ	LYS	B	338	-1.175	-30.674	8.024	1.00	0.00	N
ATOM	3239	HZ1	LYS	B	338	-0.425	-31.239	7.581	1.00	0.00	H
ATOM	3240	HZ2	LYS	B	338	-1.189	-29.743	7.552	1.00	0.00	H
ATOM	3241	HZ3	LYS	B	338	-0.817	-30.507	8.987	1.00	0.00	H
ATOM	3242	C	LYS	B	338	-5.268	-27.212	6.257	1.00	0.00	C
ATOM	3243	O	LYS	B	338	-4.825	-28.097	5.536	1.00	0.00	O
ATOM	3244	N	LEU	B	339	-5.690	-26.033	5.806	1.00	0.00	N
ATOM	3245	H	LEU	B	339	-6.197	-25.395	6.382	1.00	0.00	H
ATOM	3246	CA	LEU	B	339	-5.038	-25.524	4.614	1.00	0.00	C
ATOM	3247	CB	LEU	B	339	-6.005	-24.609	3.863	1.00	0.00	C
ATOM	3248	CG	LEU	B	339	-7.219	-25.324	3.275	1.00	0.00	C
ATOM	3249	CD1	LEU	B	339	-8.251	-24.313	2.778	1.00	0.00	C
ATOM	3250	CD2	LEU	B	339	-6.832	-26.318	2.178	1.00	0.00	C
ATOM	3251	C	LEU	B	339	-3.746	-24.794	4.972	1.00	0.00	C
ATOM	3252	O	LEU	B	339	-2.804	-24.744	4.186	1.00	0.00	O
ATOM	3253	N	ASP	B	340	-3.751	-24.227	6.190	1.00	0.00	N
ATOM	3254	H	ASP	B	340	-4.507	-24.318	6.837	1.00	0.00	H
ATOM	3255	CA	ASP	B	340	-2.677	-23.314	6.536	1.00	0.00	C
ATOM	3256	CB	ASP	B	340	-3.227	-22.038	7.209	1.00	0.00	C
ATOM	3257	CG	ASP	B	340	-3.756	-22.097	8.638	1.00	0.00	C
ATOM	3258	OD1	ASP	B	340	-3.166	-22.724	9.510	1.00	0.00	O
ATOM	3259	OD2	ASP	B	340	-4.721	-21.397	8.930	1.00	0.00	O
ATOM	3260	C	ASP	B	340	-1.467	-23.911	7.218	1.00	0.00	C
ATOM	3261	O	ASP	B	340	-0.361	-23.402	7.076	1.00	0.00	O
ATOM	3262	N	ASP	B	341	-1.714	-25.030	7.929	1.00	0.00	N
ATOM	3263	H	ASP	B	341	-2.667	-25.270	8.115	1.00	0.00	H
ATOM	3264	CA	ASP	B	341	-0.621	-25.770	8.578	1.00	0.00	C
ATOM	3265	CB	ASP	B	341	0.284	-26.464	7.542	1.00	0.00	C

ATOM	3266	CG	ASP	B	341	0.008	-27.960	7.475	1.00	0.00	C
ATOM	3267	OD1	ASP	B	341	-0.484	-28.471	6.471	1.00	0.00	O
ATOM	3268	OD2	ASP	B	341	0.386	-28.680	8.386	1.00	0.00	O
ATOM	3269	C	ASP	B	341	0.183	-24.973	9.602	1.00	0.00	C
ATOM	3270	O	ASP	B	341	1.328	-25.256	9.937	1.00	0.00	O
ATOM	3271	N	LYS	B	342	-0.497	-23.934	10.110	1.00	0.00	N
ATOM	3272	H	LYS	B	342	-1.445	-23.794	9.822	1.00	0.00	H
ATOM	3273	CA	LYS	B	342	0.088	-23.104	11.157	1.00	0.00	C
ATOM	3274	CB	LYS	B	342	-0.811	-21.879	11.285	1.00	0.00	C
ATOM	3275	CG	LYS	B	342	-0.440	-20.640	12.092	1.00	0.00	C
ATOM	3276	CD	LYS	B	342	-1.670	-19.748	12.363	1.00	0.00	C
ATOM	3277	CE	LYS	B	342	-2.638	-19.486	11.188	1.00	0.00	C
ATOM	3278	NZ	LYS	B	342	-3.677	-20.525	11.092	1.00	0.00	N
ATOM	3279	HZ1	LYS	B	342	-4.174	-20.677	11.997	1.00	0.00	H
ATOM	3280	HZ2	LYS	B	342	-4.371	-20.316	10.344	1.00	0.00	H
ATOM	3281	HZ3	LYS	B	342	-3.291	-21.435	10.755	1.00	0.00	H
ATOM	3282	C	LYS	B	342	0.126	-23.895	12.448	1.00	0.00	C
ATOM	3283	O	LYS	B	342	-0.753	-24.714	12.693	1.00	0.00	O
ATOM	3284	N	ASP	B	343	1.171	-23.608	13.246	1.00	0.00	N
ATOM	3285	H	ASP	B	343	1.842	-22.935	12.947	1.00	0.00	H
ATOM	3286	CA	ASP	B	343	1.395	-24.354	14.494	1.00	0.00	C
ATOM	3287	CB	ASP	B	343	0.347	-23.881	15.514	1.00	0.00	C
ATOM	3288	CG	ASP	B	343	0.442	-24.509	16.889	1.00	0.00	C
ATOM	3289	OD1	ASP	B	343	1.519	-24.514	17.471	1.00	0.00	O
ATOM	3290	OD2	ASP	B	343	-0.587	-24.950	17.396	1.00	0.00	O
ATOM	3291	C	ASP	B	343	1.468	-25.875	14.294	1.00	0.00	C
ATOM	3292	O	ASP	B	343	0.612	-26.659	14.693	1.00	0.00	O
ATOM	3293	N	PRO	B	344	2.543	-26.289	13.577	1.00	0.00	N
ATOM	3294	CD	PRO	B	344	3.617	-25.504	12.968	1.00	0.00	C
ATOM	3295	CA	PRO	B	344	2.647	-27.708	13.258	1.00	0.00	C
ATOM	3296	CB	PRO	B	344	3.320	-27.642	11.885	1.00	0.00	C
ATOM	3297	CG	PRO	B	344	4.289	-26.459	11.980	1.00	0.00	C
ATOM	3298	C	PRO	B	344	3.439	-28.496	14.293	1.00	0.00	C
ATOM	3299	O	PRO	B	344	4.656	-28.394	14.417	1.00	0.00	O
ATOM	3300	N	ASN	B	345	2.705	-29.365	15.003	1.00	0.00	N
ATOM	3301	H	ASN	B	345	1.711	-29.361	14.890	1.00	0.00	H
ATOM	3302	CA	ASN	B	345	3.468	-30.453	15.613	1.00	0.00	C
ATOM	3303	CB	ASN	B	345	2.662	-31.218	16.690	1.00	0.00	C
ATOM	3304	CG	ASN	B	345	2.002	-32.498	16.192	1.00	0.00	C
ATOM	3305	OD1	ASN	B	345	2.625	-33.548	16.079	1.00	0.00	O
ATOM	3306	ND2	ASN	B	345	0.706	-32.376	15.922	1.00	0.00	N
ATOM	3307	HD21	ASN	B	345	0.157	-33.172	15.682	1.00	0.00	H
ATOM	3308	HD22	ASN	B	345	0.269	-31.471	15.917	1.00	0.00	H
ATOM	3309	C	ASN	B	345	4.037	-31.363	14.539	1.00	0.00	C
ATOM	3310	O	ASN	B	345	3.427	-31.563	13.495	1.00	0.00	O
ATOM	3311	N	PHE	B	346	5.219	-31.907	14.847	1.00	0.00	N
ATOM	3312	H	PHE	B	346	5.630	-31.681	15.728	1.00	0.00	H
ATOM	3313	CA	PHE	B	346	5.991	-32.604	13.818	1.00	0.00	C
ATOM	3314	CB	PHE	B	346	7.352	-33.025	14.379	1.00	0.00	C
ATOM	3315	CG	PHE	B	346	8.227	-31.803	14.557	1.00	0.00	C
ATOM	3316	CD1	PHE	B	346	8.437	-31.271	15.848	1.00	0.00	C
ATOM	3317	CD2	PHE	B	346	8.825	-31.217	13.419	1.00	0.00	C
ATOM	3318	CE1	PHE	B	346	9.255	-30.134	16.002	1.00	0.00	C
ATOM	3319	CE2	PHE	B	346	9.642	-30.080	13.571	1.00	0.00	C
ATOM	3320	CZ	PHE	B	346	9.843	-29.547	14.861	1.00	0.00	C
ATOM	3321	C	PHE	B	346	5.339	-33.742	13.040	1.00	0.00	C
ATOM	3322	O	PHE	B	346	5.853	-34.178	12.020	1.00	0.00	O
ATOM	3323	N	LYS	B	347	4.171	-34.196	13.520	1.00	0.00	N
ATOM	3324	H	LYS	B	347	3.770	-33.791	14.342	1.00	0.00	H

```
ATOM   3325  CA  LYS B 347       3.405 -35.136  12.700  1.00  0.00           C
ATOM   3326  CB  LYS B 347       2.568 -36.021  13.624  1.00  0.00           C
ATOM   3327  CG  LYS B 347       3.408 -36.649  14.738  1.00  0.00           C
ATOM   3328  CD  LYS B 347       2.547 -37.300  15.819  1.00  0.00           C
ATOM   3329  CE  LYS B 347       3.383 -37.796  16.999  1.00  0.00           C
ATOM   3330  NZ  LYS B 347       2.494 -38.352  18.029  1.00  0.00           N
ATOM   3331  HZ1 LYS B 347       1.935 -39.130  17.625  1.00  0.00           H
ATOM   3332  HZ2 LYS B 347       3.066 -38.710  18.819  1.00  0.00           H
ATOM   3333  HZ3 LYS B 347       1.855 -37.607  18.374  1.00  0.00           H
ATOM   3334  C   LYS B 347       2.518 -34.473  11.647  1.00  0.00           C
ATOM   3335  O   LYS B 347       1.406 -34.916  11.383  1.00  0.00           O
ATOM   3336  N   ASP B 348       3.042 -33.363  11.096  1.00  0.00           N
ATOM   3337  H   ASP B 348       4.005 -33.164  11.273  1.00  0.00           H
ATOM   3338  CA  ASP B 348       2.289 -32.476  10.197  1.00  0.00           C
ATOM   3339  CB  ASP B 348       2.086 -33.109   8.807  1.00  0.00           C
ATOM   3340  CG  ASP B 348       2.095 -32.043   7.730  1.00  0.00           C
ATOM   3341  OD1 ASP B 348       1.091 -31.828   7.057  1.00  0.00           O
ATOM   3342  OD2 ASP B 348       3.118 -31.398   7.553  1.00  0.00           O
ATOM   3343  C   ASP B 348       0.967 -31.944  10.738  1.00  0.00           C
ATOM   3344  O   ASP B 348       0.072 -31.549  10.001  1.00  0.00           O
ATOM   3345  N   GLN B 349       0.823 -31.974  12.070  1.00  0.00           N
ATOM   3346  H   GLN B 349       1.594 -32.116  12.692  1.00  0.00           H
ATOM   3347  CA  GLN B 349      -0.526 -31.634  12.510  1.00  0.00           C
ATOM   3348  CB  GLN B 349      -1.243 -32.782  13.236  1.00  0.00           C
ATOM   3349  CG  GLN B 349      -1.372 -34.107  12.479  1.00  0.00           C
ATOM   3350  CD  GLN B 349      -1.915 -33.873  11.084  1.00  0.00           C
ATOM   3351  OE1 GLN B 349      -2.957 -33.265  10.873  1.00  0.00           O
ATOM   3352  NE2 GLN B 349      -1.131 -34.370  10.125  1.00  0.00           N
ATOM   3353 HE21 GLN B 349      -1.395 -34.293   9.168  1.00  0.00           H
ATOM   3354 HE22 GLN B 349      -0.258 -34.800  10.376  1.00  0.00           H
ATOM   3355  C   GLN B 349      -0.597 -30.398  13.357  1.00  0.00           C
ATOM   3356  O   GLN B 349       0.151 -30.225  14.310  1.00  0.00           O
ATOM   3357  N   VAL B 350      -1.569 -29.571  12.965  1.00  0.00           N
ATOM   3358  H   VAL B 350      -2.114 -29.833  12.172  1.00  0.00           H
ATOM   3359  CA  VAL B 350      -1.916 -28.368  13.720  1.00  0.00           C
ATOM   3360  CB  VAL B 350      -3.148 -27.768  13.028  1.00  0.00           C
ATOM   3361  CG1 VAL B 350      -3.892 -26.721  13.855  1.00  0.00           C
ATOM   3362  CG2 VAL B 350      -2.756 -27.248  11.643  1.00  0.00           C
ATOM   3363  C   VAL B 350      -2.171 -28.662  15.193  1.00  0.00           C
ATOM   3364  O   VAL B 350      -2.820 -29.645  15.537  1.00  0.00           O
ATOM   3365  N   ILE B 351      -1.642 -27.776  16.039  1.00  0.00           N
ATOM   3366  H   ILE B 351      -1.063 -27.017  15.723  1.00  0.00           H
ATOM   3367  CA  ILE B 351      -2.027 -27.898  17.436  1.00  0.00           C
ATOM   3368  CB  ILE B 351      -0.812 -28.111  18.366  1.00  0.00           C
ATOM   3369  CG2 ILE B 351      -1.011 -29.394  19.178  1.00  0.00           C
ATOM   3370  CG1 ILE B 351       0.544 -28.097  17.655  1.00  0.00           C
ATOM   3371  CD1 ILE B 351       1.720 -27.960  18.623  1.00  0.00           C
ATOM   3372  C   ILE B 351      -2.928 -26.748  17.886  1.00  0.00           C
ATOM   3373  O   ILE B 351      -3.518 -26.010  17.097  1.00  0.00           O
ATOM   3374  N   LEU B 352      -3.061 -26.630  19.217  1.00  0.00           N
ATOM   3375  H   LEU B 352      -2.519 -27.183  19.846  1.00  0.00           H
ATOM   3376  CA  LEU B 352      -4.014 -25.635  19.694  1.00  0.00           C
ATOM   3377  CB  LEU B 352      -4.518 -25.996  21.091  1.00  0.00           C
ATOM   3378  CG  LEU B 352      -5.342 -27.287  21.094  1.00  0.00           C
ATOM   3379  CD1 LEU B 352      -5.696 -27.729  22.514  1.00  0.00           C
ATOM   3380  CD2 LEU B 352      -6.584 -27.177  20.204  1.00  0.00           C
ATOM   3381  C   LEU B 352      -3.555 -24.191  19.631  1.00  0.00           C
ATOM   3382  O   LEU B 352      -4.364 -23.275  19.707  1.00  0.00           O
ATOM   3383  N   LEU B 353      -2.238 -24.002  19.472  1.00  0.00           N
```

```
ATOM   3384  H   LEU B 353      -1.610 -24.743  19.216  1.00  0.00           H
ATOM   3385  CA  LEU B 353      -1.784 -22.619  19.348  1.00  0.00           C
ATOM   3386  CB  LEU B 353      -0.311 -22.490  19.744  1.00  0.00           C
ATOM   3387  CG  LEU B 353      -0.026 -22.942  21.179  1.00  0.00           C
ATOM   3388  CD1 LEU B 353       1.474 -22.973  21.476  1.00  0.00           C
ATOM   3389  CD2 LEU B 353      -0.796 -22.108  22.206  1.00  0.00           C
ATOM   3390  C   LEU B 353      -2.047 -21.988  17.987  1.00  0.00           C
ATOM   3391  O   LEU B 353      -1.840 -20.787  17.800  1.00  0.00           O
ATOM   3392  N   ASN B 354      -2.555 -22.833  17.073  1.00  0.00           N
ATOM   3393  H   ASN B 354      -2.412 -23.819  17.208  1.00  0.00           H
ATOM   3394  CA  ASN B 354      -3.223 -22.329  15.875  1.00  0.00           C
ATOM   3395  CB  ASN B 354      -3.083 -23.354  14.741  1.00  0.00           C
ATOM   3396  CG  ASN B 354      -3.601 -22.846  13.408  1.00  0.00           C
ATOM   3397  OD1 ASN B 354      -4.195 -21.784  13.290  1.00  0.00           O
ATOM   3398  ND2 ASN B 354      -3.345 -23.661  12.381  1.00  0.00           N
ATOM   3399 HD21 ASN B 354      -3.669 -23.538  11.437  1.00  0.00           H
ATOM   3400 HD22 ASN B 354      -2.745 -24.452  12.539  1.00  0.00           H
ATOM   3401  C   ASN B 354      -4.684 -22.010  16.151  1.00  0.00           C
ATOM   3402  O   ASN B 354      -5.142 -20.886  15.988  1.00  0.00           O
ATOM   3403  N   LYS B 355      -5.398 -23.066  16.592  1.00  0.00           N
ATOM   3404  H   LYS B 355      -4.920 -23.932  16.754  1.00  0.00           H
ATOM   3405  CA  LYS B 355      -6.853 -22.932  16.745  1.00  0.00           C
ATOM   3406  CB  LYS B 355      -7.516 -24.269  17.092  1.00  0.00           C
ATOM   3407  CG  LYS B 355      -7.533 -25.298  15.960  1.00  0.00           C
ATOM   3408  CD  LYS B 355      -8.921 -25.915  15.732  1.00  0.00           C
ATOM   3409  CE  LYS B 355      -9.974 -24.891  15.289  1.00  0.00           C
ATOM   3410  NZ  LYS B 355     -11.266 -25.555  15.066  1.00  0.00           N
ATOM   3411  HZ1 LYS B 355     -11.751 -25.781  15.955  1.00  0.00           H
ATOM   3412  HZ2 LYS B 355     -11.874 -24.916  14.511  1.00  0.00           H
ATOM   3413  HZ3 LYS B 355     -11.134 -26.409  14.491  1.00  0.00           H
ATOM   3414  C   LYS B 355      -7.357 -21.892  17.734  1.00  0.00           C
ATOM   3415  O   LYS B 355      -8.357 -21.225  17.513  1.00  0.00           O
ATOM   3416  N   HIS B 356      -6.646 -21.842  18.869  1.00  0.00           N
ATOM   3417  H   HIS B 356      -5.819 -22.401  18.919  1.00  0.00           H
ATOM   3418  CA  HIS B 356      -6.951 -20.951  19.993  1.00  0.00           C
ATOM   3419  CB  HIS B 356      -6.273 -19.580  19.800  1.00  0.00           C
ATOM   3420  CG  HIS B 356      -6.995 -18.633  18.859  1.00  0.00           C
ATOM   3421  ND1 HIS B 356      -8.242 -18.167  19.061  1.00  0.00           N
ATOM   3422  HD1 HIS B 356      -8.866 -18.446  19.767  1.00  0.00           H
ATOM   3423  CD2 HIS B 356      -6.485 -18.029  17.705  1.00  0.00           C
ATOM   3424  NE2 HIS B 356      -7.436 -17.186  17.227  1.00  0.00           N
ATOM   3425  CE1 HIS B 356      -8.520 -17.280  18.060  1.00  0.00           C
ATOM   3426  C   HIS B 356      -8.388 -20.843  20.506  1.00  0.00           C
ATOM   3427  O   HIS B 356      -8.771 -19.846  21.121  1.00  0.00           O
ATOM   3428  N   ILE B 357      -9.147 -21.927  20.238  1.00  0.00           N
ATOM   3429  H   ILE B 357      -8.695 -22.670  19.755  1.00  0.00           H
ATOM   3430  CA  ILE B 357     -10.585 -22.003  20.543  1.00  0.00           C
ATOM   3431  CB  ILE B 357     -11.085 -23.457  20.441  1.00  0.00           C
ATOM   3432  CG2 ILE B 357     -12.572 -23.605  20.792  1.00  0.00           C
ATOM   3433  CG1 ILE B 357     -10.803 -24.027  19.051  1.00  0.00           C
ATOM   3434  CD1 ILE B 357     -11.156 -25.512  18.943  1.00  0.00           C
ATOM   3435  C   ILE B 357     -10.956 -21.403  21.885  1.00  0.00           C
ATOM   3436  O   ILE B 357     -10.584 -21.900  22.940  1.00  0.00           O
ATOM   3437  N   ASP B 358     -11.640 -20.258  21.761  1.00  0.00           N
ATOM   3438  H   ASP B 358     -11.985 -20.022  20.854  1.00  0.00           H
ATOM   3439  CA  ASP B 358     -12.206 -19.525  22.897  1.00  0.00           C
ATOM   3440  CB  ASP B 358     -13.590 -20.089  23.242  1.00  0.00           C
ATOM   3441  CG  ASP B 358     -14.425 -20.101  21.971  1.00  0.00           C
ATOM   3442  OD1 ASP B 358     -14.649 -19.049  21.385  1.00  0.00           O
```

```
ATOM   3443  OD2 ASP B 358     -14.789 -21.172  21.508  1.00  0.00           O
ATOM   3444  C   ASP B 358     -11.335 -19.248  24.119  1.00  0.00           C
ATOM   3445  O   ASP B 358     -11.802 -18.858  25.189  1.00  0.00           O
ATOM   3446  N   ALA B 359     -10.021 -19.394  23.879  1.00  0.00           N
ATOM   3447  H   ALA B 359      -9.765 -19.808  23.006  1.00  0.00           H
ATOM   3448  CA  ALA B 359      -9.003 -18.986  24.843  1.00  0.00           C
ATOM   3449  CB  ALA B 359      -7.930 -20.067  24.973  1.00  0.00           C
ATOM   3450  C   ALA B 359      -8.353 -17.675  24.432  1.00  0.00           C
ATOM   3451  O   ALA B 359      -8.017 -16.830  25.258  1.00  0.00           O
ATOM   3452  N   TYR B 360      -8.269 -17.542  23.088  1.00  0.00           N
ATOM   3453  H   TYR B 360      -8.433 -18.368  22.545  1.00  0.00           H
ATOM   3454  CA  TYR B 360      -8.048 -16.265  22.400  1.00  0.00           C
ATOM   3455  CB  TYR B 360      -8.930 -15.136  22.958  1.00  0.00           C
ATOM   3456  CG  TYR B 360     -10.369 -15.429  22.625  1.00  0.00           C
ATOM   3457  CD1 TYR B 360     -11.258 -15.796  23.655  1.00  0.00           C
ATOM   3458  CE1 TYR B 360     -12.586 -16.102  23.322  1.00  0.00           C
ATOM   3459  CD2 TYR B 360     -10.770 -15.335  21.280  1.00  0.00           C
ATOM   3460  CE2 TYR B 360     -12.093 -15.644  20.951  1.00  0.00           C
ATOM   3461  CZ  TYR B 360     -12.977 -16.031  21.971  1.00  0.00           C
ATOM   3462  OH  TYR B 360     -14.265 -16.344  21.617  1.00  0.00           O
ATOM   3463  HH  TYR B 360     -14.432 -17.261  21.846  1.00  0.00           H
ATOM   3464  C   TYR B 360      -6.631 -15.770  22.181  1.00  0.00           C
ATOM   3465  O   TYR B 360      -6.073 -14.979  22.938  1.00  0.00           O
ATOM   3466  N   LYS B 361      -6.079 -16.199  21.029  1.00  0.00           N
ATOM   3467  H   LYS B 361      -6.583 -16.854  20.468  1.00  0.00           H
ATOM   3468  CA  LYS B 361      -4.972 -15.385  20.516  1.00  0.00           C
ATOM   3469  CB  LYS B 361      -4.092 -16.112  19.476  1.00  0.00           C
ATOM   3470  CG  LYS B 361      -2.931 -16.949  20.053  1.00  0.00           C
ATOM   3471  CD  LYS B 361      -2.016 -17.592  18.987  1.00  0.00           C
ATOM   3472  CE  LYS B 361      -0.766 -18.298  19.557  1.00  0.00           C
ATOM   3473  NZ  LYS B 361       0.022 -18.961  18.498  1.00  0.00           N
ATOM   3474  HZ1 LYS B 361       0.834 -19.470  18.906  1.00  0.00           H
ATOM   3475  HZ2 LYS B 361      -0.583 -19.652  17.998  1.00  0.00           H
ATOM   3476  HZ3 LYS B 361       0.410 -18.277  17.818  1.00  0.00           H
ATOM   3477  C   LYS B 361      -5.503 -14.063  19.975  1.00  0.00           C
ATOM   3478  O   LYS B 361      -5.187 -12.997  20.497  1.00  0.00           O
ATOM   3479  N   THR B 362      -6.377 -14.177  18.963  1.00  0.00           N
ATOM   3480  H   THR B 362      -6.675 -15.072  18.633  1.00  0.00           H
ATOM   3481  CA  THR B 362      -7.103 -12.970  18.556  1.00  0.00           C
ATOM   3482  CB  THR B 362      -7.673 -13.186  17.150  1.00  0.00           C
ATOM   3483  OG1 THR B 362      -8.550 -14.319  17.131  1.00  0.00           O
ATOM   3484  HG1 THR B 362      -8.090 -15.038  16.699  1.00  0.00           H
ATOM   3485  CG2 THR B 362      -6.573 -13.339  16.096  1.00  0.00           C
ATOM   3486  C   THR B 362      -8.191 -12.634  19.579  1.00  0.00           C
ATOM   3487  O   THR B 362      -8.249 -13.298  20.608  1.00  0.00           O
ATOM   3488  N   PHE B 363      -9.027 -11.616  19.279  1.00  0.00           N
ATOM   3489  H   PHE B 363      -8.881 -11.028  18.484  1.00  0.00           H
ATOM   3490  CA  PHE B 363     -10.204 -11.377  20.129  1.00  0.00           C
ATOM   3491  CB  PHE B 363      -9.784 -10.811  21.505  1.00  0.00           C
ATOM   3492  CG  PHE B 363     -10.959 -10.474  22.400  1.00  0.00           C
ATOM   3493  CD1 PHE B 363     -11.991 -11.416  22.611  1.00  0.00           C
ATOM   3494  CD2 PHE B 363     -11.006  -9.197  23.000  1.00  0.00           C
ATOM   3495  CE1 PHE B 363     -13.103 -11.059  23.394  1.00  0.00           C
ATOM   3496  CE2 PHE B 363     -12.108  -8.844  23.802  1.00  0.00           C
ATOM   3497  CZ  PHE B 363     -13.147  -9.778  23.984  1.00  0.00           C
ATOM   3498  C   PHE B 363     -11.277 -10.518  19.454  1.00  0.00           C
ATOM   3499  O   PHE B 363     -11.039  -9.369  19.092  1.00  0.00           O
ATOM   3500  N   PRO B 364     -12.463 -11.152  19.282  1.00  0.00           N
ATOM   3501  CD  PRO B 364     -12.677 -12.580  19.504  1.00  0.00           C
```

ATOM	3502	CA	PRO	B	364	-13.688	-10.445	18.857	1.00	0.00	C
ATOM	3503	CB	PRO	B	364	-14.583	-11.659	18.527	1.00	0.00	C
ATOM	3504	CG	PRO	B	364	-14.181	-12.757	19.496	1.00	0.00	C
ATOM	3505	C	PRO	B	364	-14.202	-9.504	19.969	1.00	0.00	C
ATOM	3506	O	PRO	B	364	-13.503	-9.297	20.951	1.00	0.00	O
ATOM	3507	N	PRO	B	365	-15.425	-8.911	19.807	1.00	0.00	N
ATOM	3508	CD	PRO	B	365	-16.223	-8.789	18.585	1.00	0.00	C
ATOM	3509	CA	PRO	B	365	-16.070	-8.219	20.939	1.00	0.00	C
ATOM	3510	CB	PRO	B	365	-17.473	-7.916	20.402	1.00	0.00	C
ATOM	3511	CG	PRO	B	365	-17.272	-7.734	18.902	1.00	0.00	C
ATOM	3512	C	PRO	B	365	-16.096	-8.924	22.296	1.00	0.00	C
ATOM	3513	O	PRO	B	365	-15.844	-10.113	22.430	1.00	0.00	O
ATOM	3514	N	THR	B	366	-16.425	-8.094	23.298	1.00	0.00	N
ATOM	3515	H	THR	B	366	-16.764	-7.178	23.078	1.00	0.00	H
ATOM	3516	CA	THR	B	366	-16.282	-8.367	24.733	1.00	0.00	C
ATOM	3517	CB	THR	B	366	-17.129	-7.347	25.505	1.00	0.00	C
ATOM	3518	OG1	THR	B	366	-17.938	-6.590	24.590	1.00	0.00	O
ATOM	3519	HG1	THR	B	366	-18.403	-5.945	25.111	1.00	0.00	H
ATOM	3520	CG2	THR	B	366	-16.266	-6.419	26.360	1.00	0.00	C
ATOM	3521	C	THR	B	366	-16.526	-9.769	25.278	1.00	0.00	C
ATOM	3522	O	THR	B	366	-15.631	-10.448	25.776	1.00	0.00	O
ATOM	3523	N	GLU	B	367	-17.812	-10.144	25.206	1.00	0.00	N
ATOM	3524	H	GLU	B	367	-18.458	-9.526	24.759	1.00	0.00	H
ATOM	3525	CA	GLU	B	367	-18.284	-11.316	25.953	1.00	0.00	C
ATOM	3526	CB	GLU	B	367	-19.791	-11.538	25.731	1.00	0.00	C
ATOM	3527	CG	GLU	B	367	-20.649	-10.266	25.844	1.00	0.00	C
ATOM	3528	CD	GLU	B	367	-20.612	-9.704	27.253	1.00	0.00	C
ATOM	3529	OE1	GLU	B	367	-21.408	-10.136	28.083	1.00	0.00	O
ATOM	3530	OE2	GLU	B	367	-19.798	-8.826	27.541	1.00	0.00	O
ATOM	3531	C	GLU	B	367	-17.468	-12.618	25.892	1.00	0.00	C
ATOM	3532	O	GLU	B	367	-17.155	-13.185	26.931	1.00	0.00	O
ATOM	3533	N	PRO	B	368	-17.079	-13.084	24.673	1.00	0.00	N
ATOM	3534	CD	PRO	B	368	-17.494	-12.613	23.348	1.00	0.00	C
ATOM	3535	CA	PRO	B	368	-16.156	-14.231	24.571	1.00	0.00	C
ATOM	3536	CB	PRO	B	368	-15.740	-14.153	23.105	1.00	0.00	C
ATOM	3537	CG	PRO	B	368	-16.970	-13.647	22.370	1.00	0.00	C
ATOM	3538	C	PRO	B	368	-14.953	-14.333	25.523	1.00	0.00	C
ATOM	3539	O	PRO	B	368	-14.508	-15.415	25.898	1.00	0.00	O
ATOM	3540	N	LYS	B	369	-14.399	-13.166	25.883	1.00	0.00	N
ATOM	3541	H	LYS	B	369	-14.816	-12.292	25.622	1.00	0.00	H
ATOM	3542	CA	LYS	B	369	-13.225	-13.244	26.755	1.00	0.00	C
ATOM	3543	CB	LYS	B	369	-12.095	-12.421	26.138	1.00	0.00	C
ATOM	3544	CG	LYS	B	369	-10.736	-13.087	25.913	1.00	0.00	C
ATOM	3545	CD	LYS	B	369	-9.950	-13.208	27.207	1.00	0.00	C
ATOM	3546	CE	LYS	B	369	-8.489	-13.628	27.063	1.00	0.00	C
ATOM	3547	NZ	LYS	B	369	-7.863	-13.488	28.383	1.00	0.00	N
ATOM	3548	HZ1	LYS	B	369	-8.292	-14.155	29.059	1.00	0.00	H
ATOM	3549	HZ2	LYS	B	369	-6.839	-13.633	28.367	1.00	0.00	H
ATOM	3550	HZ3	LYS	B	369	-8.087	-12.552	28.787	1.00	0.00	H
ATOM	3551	C	LYS	B	369	-13.492	-12.802	28.184	1.00	0.00	C
ATOM	3552	O	LYS	B	369	-12.577	-12.531	28.955	1.00	0.00	O
ATOM	3553	N	LYS	B	370	-14.792	-12.681	28.489	1.00	0.00	N
ATOM	3554	H	LYS	B	370	-15.484	-13.035	27.858	1.00	0.00	H
ATOM	3555	CA	LYS	B	370	-15.195	-12.050	29.744	1.00	0.00	C
ATOM	3556	CB	LYS	B	370	-16.715	-11.912	29.755	1.00	0.00	C
ATOM	3557	CG	LYS	B	370	-17.294	-10.848	30.683	1.00	0.00	C
ATOM	3558	CD	LYS	B	370	-18.729	-10.574	30.246	1.00	0.00	C
ATOM	3559	CE	LYS	B	370	-19.410	-9.395	30.938	1.00	0.00	C
ATOM	3560	NZ	LYS	B	370	-20.569	-9.012	30.126	1.00	0.00	N

ATOM	3561	HZ1	LYS	B	370	-21.122	-9.846	29.826	1.00	0.00	H
ATOM	3562	HZ2	LYS	B	370	-21.192	-8.303	30.546	1.00	0.00	H
ATOM	3563	HZ3	LYS	B	370	-20.250	-8.675	29.189	1.00	0.00	H
ATOM	3564	C	LYS	B	370	-14.680	-12.751	30.979	1.00	0.00	C
ATOM	3565	O	LYS	B	370	-14.178	-12.140	31.914	1.00	0.00	O
ATOM	3566	N	ASP	B	371	-14.824	-14.076	30.883	1.00	0.00	N
ATOM	3567	H	ASP	B	371	-15.138	-14.467	30.016	1.00	0.00	H
ATOM	3568	CA	ASP	B	371	-14.370	-15.025	31.892	1.00	0.00	C
ATOM	3569	CB	ASP	B	371	-14.691	-16.457	31.392	1.00	0.00	C
ATOM	3570	CG	ASP	B	371	-14.043	-16.832	30.049	1.00	0.00	C
ATOM	3571	OD1	ASP	B	371	-13.663	-17.984	29.879	1.00	0.00	O
ATOM	3572	OD2	ASP	B	371	-13.854	-15.981	29.177	1.00	0.00	O
ATOM	3573	C	ASP	B	371	-12.919	-14.842	32.331	1.00	0.00	C
ATOM	3574	O	ASP	B	371	-12.602	-14.400	33.428	1.00	0.00	O
ATOM	3575	N	LYS	B	372	-12.023	-15.186	31.407	1.00	0.00	N
ATOM	3576	H	LYS	B	372	-12.377	-15.418	30.501	1.00	0.00	H
ATOM	3577	CA	LYS	B	372	-10.609	-15.144	31.754	1.00	0.00	C
ATOM	3578	CB	LYS	B	372	-9.859	-16.390	31.237	1.00	0.00	C
ATOM	3579	CG	LYS	B	372	-10.448	-17.184	30.055	1.00	0.00	C
ATOM	3580	CD	LYS	B	372	-10.551	-16.406	28.740	1.00	0.00	C
ATOM	3581	CE	LYS	B	372	-11.063	-17.195	27.529	1.00	0.00	C
ATOM	3582	NZ	LYS	B	372	-12.490	-17.554	27.581	1.00	0.00	N
ATOM	3583	HZ1	LYS	B	372	-13.116	-16.724	27.689	1.00	0.00	H
ATOM	3584	HZ2	LYS	B	372	-12.751	-18.083	26.721	1.00	0.00	H
ATOM	3585	HZ3	LYS	B	372	-12.716	-18.119	28.428	1.00	0.00	H
ATOM	3586	C	LYS	B	372	-9.938	-13.848	31.344	1.00	0.00	C
ATOM	3587	O	LYS	B	372	-9.007	-13.833	30.544	1.00	0.00	O
ATOM	3588	N	LYS	B	373	-10.482	-12.762	31.920	1.00	0.00	N
ATOM	3589	H	LYS	B	373	-11.216	-12.949	32.580	1.00	0.00	H
ATOM	3590	CA	LYS	B	373	-10.110	-11.378	31.597	1.00	0.00	C
ATOM	3591	CB	LYS	B	373	-8.869	-10.863	32.348	1.00	0.00	C
ATOM	3592	CG	LYS	B	373	-8.863	-9.326	32.286	1.00	0.00	C
ATOM	3593	CD	LYS	B	373	-7.572	-8.592	32.657	1.00	0.00	C
ATOM	3594	CE	LYS	B	373	-7.763	-7.088	32.419	1.00	0.00	C
ATOM	3595	NZ	LYS	B	373	-6.528	-6.335	32.672	1.00	0.00	N
ATOM	3596	HZ1	LYS	B	373	-6.678	-5.322	32.458	1.00	0.00	H
ATOM	3597	HZ2	LYS	B	373	-6.218	-6.412	33.657	1.00	0.00	H
ATOM	3598	HZ3	LYS	B	373	-5.764	-6.632	32.036	1.00	0.00	H
ATOM	3599	C	LYS	B	373	-9.994	-11.004	30.125	1.00	0.00	C
ATOM	3600	O	LYS	B	373	-9.039	-11.333	29.421	1.00	0.00	O
ATOM	3601	N	LYS	B	374	-11.018	-10.236	29.731	1.00	0.00	N
ATOM	3602	H	LYS	B	374	-11.787	-10.206	30.366	1.00	0.00	H
ATOM	3603	CA	LYS	B	374	-11.068	-9.567	28.429	1.00	0.00	C
ATOM	3604	CB	LYS	B	374	-12.170	-8.509	28.458	1.00	0.00	C
ATOM	3605	CG	LYS	B	374	-13.563	-9.097	28.664	1.00	0.00	C
ATOM	3606	CD	LYS	B	374	-14.618	-8.143	29.239	1.00	0.00	C
ATOM	3607	CE	LYS	B	374	-14.576	-7.951	30.765	1.00	0.00	C
ATOM	3608	NZ	LYS	B	374	-13.507	-7.040	31.181	1.00	0.00	N
ATOM	3609	HZ1	LYS	B	374	-13.445	-6.878	32.200	1.00	0.00	H
ATOM	3610	HZ2	LYS	B	374	-12.554	-7.340	30.875	1.00	0.00	H
ATOM	3611	HZ3	LYS	B	374	-13.563	-6.113	30.703	1.00	0.00	H
ATOM	3612	C	LYS	B	374	-9.768	-8.919	27.982	1.00	0.00	C
ATOM	3613	O	LYS	B	374	-9.000	-8.381	28.768	1.00	0.00	O
ATOM	3614	N	LYS	B	375	-9.565	-8.980	26.656	1.00	0.00	N
ATOM	3615	H	LYS	B	375	-10.231	-9.434	26.068	1.00	0.00	H
ATOM	3616	CA	LYS	B	375	-8.462	-8.163	26.147	1.00	0.00	C
ATOM	3617	CB	LYS	B	375	-7.896	-8.711	24.838	1.00	0.00	C
ATOM	3618	CG	LYS	B	375	-7.302	-10.104	25.027	1.00	0.00	C
ATOM	3619	CD	LYS	B	375	-6.554	-10.625	23.800	1.00	0.00	C

```
ATOM   3620  CE  LYS B 375      -6.008 -12.021  24.086  1.00  0.00           C
ATOM   3621  NZ  LYS B 375      -5.242 -12.534  22.949  1.00  0.00           N
ATOM   3622  HZ1 LYS B 375      -5.729 -12.369  22.042  1.00  0.00           H
ATOM   3623  HZ2 LYS B 375      -4.297 -12.115  22.892  1.00  0.00           H
ATOM   3624  HZ3 LYS B 375      -5.166 -13.569  23.043  1.00  0.00           H
ATOM   3625  C   LYS B 375      -8.838  -6.703  26.001  1.00  0.00           C
ATOM   3626  O   LYS B 375      -8.045  -5.806  26.262  1.00  0.00           O
ATOM   3627  N   ALA B 376     -10.120  -6.518  25.616  1.00  0.00           N
ATOM   3628  H   ALA B 376     -10.653  -7.308  25.328  1.00  0.00           H
ATOM   3629  CA  ALA B 376     -10.722  -5.185  25.494  1.00  0.00           C
ATOM   3630  CB  ALA B 376     -12.243  -5.287  25.575  1.00  0.00           C
ATOM   3631  C   ALA B 376     -10.260  -4.192  26.543  1.00  0.00           C
ATOM   3632  O   ALA B 376      -9.710  -3.142  26.238  1.00  0.00           O
ATOM   3633  N   ASP B 377     -10.423  -4.644  27.798  1.00  0.00           N
ATOM   3634  H   ASP B 377     -11.007  -5.435  27.978  1.00  0.00           H
ATOM   3635  CA  ASP B 377      -9.955  -3.913  28.977  1.00  0.00           C
ATOM   3636  CB  ASP B 377      -9.832  -4.842  30.199  1.00  0.00           C
ATOM   3637  CG  ASP B 377     -11.057  -5.711  30.449  1.00  0.00           C
ATOM   3638  OD1 ASP B 377     -12.106  -5.494  29.848  1.00  0.00           O
ATOM   3639  OD2 ASP B 377     -10.975  -6.640  31.250  1.00  0.00           O
ATOM   3640  C   ASP B 377      -8.630  -3.207  28.779  1.00  0.00           C
ATOM   3641  O   ASP B 377      -8.527  -1.990  28.817  1.00  0.00           O
ATOM   3642  N   GLU B 378      -7.607  -4.039  28.532  1.00  0.00           N
ATOM   3643  H   GLU B 378      -7.791  -5.002  28.336  1.00  0.00           H
ATOM   3644  CA  GLU B 378      -6.282  -3.448  28.358  1.00  0.00           C
ATOM   3645  CB  GLU B 378      -5.188  -4.500  28.539  1.00  0.00           C
ATOM   3646  CG  GLU B 378      -5.176  -5.156  29.924  1.00  0.00           C
ATOM   3647  CD  GLU B 378      -4.870  -4.145  31.020  1.00  0.00           C
ATOM   3648  OE1 GLU B 378      -5.664  -3.994  31.944  1.00  0.00           O
ATOM   3649  OE2 GLU B 378      -3.827  -3.509  30.974  1.00  0.00           O
ATOM   3650  C   GLU B 378      -6.095  -2.719  27.042  1.00  0.00           C
ATOM   3651  O   GLU B 378      -5.302  -1.793  26.926  1.00  0.00           O
ATOM   3652  N   THR B 379      -6.871  -3.167  26.049  1.00  0.00           N
ATOM   3653  H   THR B 379      -7.610  -3.813  26.238  1.00  0.00           H
ATOM   3654  CA  THR B 379      -6.707  -2.558  24.732  1.00  0.00           C
ATOM   3655  CB  THR B 379      -7.288  -3.482  23.653  1.00  0.00           C
ATOM   3656  OG1 THR B 379      -6.928  -4.847  23.926  1.00  0.00           O
ATOM   3657  HG1 THR B 379      -7.170  -5.017  24.829  1.00  0.00           H
ATOM   3658  CG2 THR B 379      -6.805  -3.093  22.252  1.00  0.00           C
ATOM   3659  C   THR B 379      -7.231  -1.125  24.639  1.00  0.00           C
ATOM   3660  O   THR B 379      -6.703  -0.293  23.912  1.00  0.00           O
ATOM   3661  N   GLN B 380      -8.257  -0.843  25.460  1.00  0.00           N
ATOM   3662  H   GLN B 380      -8.713  -1.569  25.974  1.00  0.00           H
ATOM   3663  CA  GLN B 380      -8.564   0.568  25.694  1.00  0.00           C
ATOM   3664  CB  GLN B 380      -9.969   0.783  26.244  1.00  0.00           C
ATOM   3665  CG  GLN B 380     -11.132   0.644  25.269  1.00  0.00           C
ATOM   3666  CD  GLN B 380     -12.304   1.377  25.891  1.00  0.00           C
ATOM   3667  OE1 GLN B 380     -12.145   2.437  26.493  1.00  0.00           O
ATOM   3668  NE2 GLN B 380     -13.476   0.763  25.746  1.00  0.00           N
ATOM   3669 HE21 GLN B 380     -14.358   1.100  26.069  1.00  0.00           H
ATOM   3670 HE22 GLN B 380     -13.606  -0.110  25.263  1.00  0.00           H
ATOM   3671  C   GLN B 380      -7.621   1.192  26.701  1.00  0.00           C
ATOM   3672  O   GLN B 380      -7.110   2.298  26.547  1.00  0.00           O
ATOM   3673  N   ALA B 381      -7.432   0.420  27.783  1.00  0.00           N
ATOM   3674  H   ALA B 381      -7.887  -0.469  27.845  1.00  0.00           H
ATOM   3675  CA  ALA B 381      -6.749   0.989  28.938  1.00  0.00           C
ATOM   3676  CB  ALA B 381      -6.649   0.002  30.097  1.00  0.00           C
ATOM   3677  C   ALA B 381      -5.374   1.538  28.664  1.00  0.00           C
ATOM   3678  O   ALA B 381      -5.077   2.646  29.071  1.00  0.00           O
```

ATOM	3679	N	LEU	B	382	-4.538	0.757	27.966	1.00	0.00	N
ATOM	3680	H	LEU	B	382	-4.861	-0.107	27.578	1.00	0.00	H
ATOM	3681	CA	LEU	B	382	-3.200	1.291	27.701	1.00	0.00	C
ATOM	3682	CB	LEU	B	382	-2.261	0.211	27.136	1.00	0.00	C
ATOM	3683	CG	LEU	B	382	-0.782	0.613	27.162	1.00	0.00	C
ATOM	3684	CD1	LEU	B	382	-0.277	0.877	28.582	1.00	0.00	C
ATOM	3685	CD2	LEU	B	382	0.090	-0.399	26.421	1.00	0.00	C
ATOM	3686	C	LEU	B	382	-3.136	2.638	26.967	1.00	0.00	C
ATOM	3687	O	LEU	B	382	-2.532	3.573	27.485	1.00	0.00	O
ATOM	3688	N	PRO	B	383	-3.798	2.770	25.779	1.00	0.00	N
ATOM	3689	CD	PRO	B	383	-4.396	1.748	24.920	1.00	0.00	C
ATOM	3690	CA	PRO	B	383	-3.969	4.117	25.213	1.00	0.00	C
ATOM	3691	CB	PRO	B	383	-4.941	3.888	24.052	1.00	0.00	C
ATOM	3692	CG	PRO	B	383	-4.657	2.460	23.601	1.00	0.00	C
ATOM	3693	C	PRO	B	383	-4.452	5.166	26.208	1.00	0.00	C
ATOM	3694	O	PRO	B	383	-3.827	6.204	26.400	1.00	0.00	O
ATOM	3695	N	GLN	B	384	-5.573	4.831	26.876	1.00	0.00	N
ATOM	3696	H	GLN	B	384	-6.037	3.963	26.677	1.00	0.00	H
ATOM	3697	CA	GLN	B	384	-6.098	5.768	27.873	1.00	0.00	C
ATOM	3698	CB	GLN	B	384	-7.338	5.224	28.582	1.00	0.00	C
ATOM	3699	CG	GLN	B	384	-8.604	5.086	27.732	1.00	0.00	C
ATOM	3700	CD	GLN	B	384	-9.777	4.967	28.685	1.00	0.00	C
ATOM	3701	OE1	GLN	B	384	-9.774	5.559	29.766	1.00	0.00	O
ATOM	3702	NE2	GLN	B	384	-10.774	4.188	28.261	1.00	0.00	N
ATOM	3703	HE21	GLN	B	384	-11.594	4.024	28.807	1.00	0.00	H
ATOM	3704	HE22	GLN	B	384	-10.799	3.715	27.373	1.00	0.00	H
ATOM	3705	C	GLN	B	384	-5.105	6.212	28.938	1.00	0.00	C
ATOM	3706	O	GLN	B	384	-5.026	7.380	29.296	1.00	0.00	O
ATOM	3707	N	ARG	B	385	-4.347	5.222	29.433	1.00	0.00	N
ATOM	3708	H	ARG	B	385	-4.407	4.329	28.995	1.00	0.00	H
ATOM	3709	CA	ARG	B	385	-3.354	5.474	30.476	1.00	0.00	C
ATOM	3710	CB	ARG	B	385	-2.640	4.177	30.896	1.00	0.00	C
ATOM	3711	CG	ARG	B	385	-3.498	3.145	31.641	1.00	0.00	C
ATOM	3712	CD	ARG	B	385	-2.811	1.776	31.769	1.00	0.00	C
ATOM	3713	NE	ARG	B	385	-3.690	0.784	32.396	1.00	0.00	N
ATOM	3714	HE	ARG	B	385	-4.245	1.086	33.172	1.00	0.00	H
ATOM	3715	CZ	ARG	B	385	-3.731	-0.506	31.973	1.00	0.00	C
ATOM	3716	NH1	ARG	B	385	-2.972	-0.925	30.966	1.00	0.00	N
ATOM	3717	HH11	ARG	B	385	-2.337	-0.326	30.484	1.00	0.00	H
ATOM	3718	HH12	ARG	B	385	-3.047	-1.894	30.687	1.00	0.00	H
ATOM	3719	NH2	ARG	B	385	-4.550	-1.376	32.559	1.00	0.00	N
ATOM	3720	HH21	ARG	B	385	-5.135	-1.142	33.331	1.00	0.00	H
ATOM	3721	HH22	ARG	B	385	-4.599	-2.329	32.207	1.00	0.00	H
ATOM	3722	C	ARG	B	385	-2.330	6.479	30.004	1.00	0.00	C
ATOM	3723	O	ARG	B	385	-2.001	7.442	30.680	1.00	0.00	O
ATOM	3724	N	GLN	B	386	-1.852	6.218	28.782	1.00	0.00	N
ATOM	3725	H	GLN	B	386	-2.238	5.464	28.245	1.00	0.00	H
ATOM	3726	CA	GLN	B	386	-0.792	7.081	28.270	1.00	0.00	C
ATOM	3727	CB	GLN	B	386	-0.063	6.379	27.127	1.00	0.00	C
ATOM	3728	CG	GLN	B	386	0.377	4.987	27.588	1.00	0.00	C
ATOM	3729	CD	GLN	B	386	1.228	4.323	26.535	1.00	0.00	C
ATOM	3730	OE1	GLN	B	386	2.451	4.370	26.584	1.00	0.00	O
ATOM	3731	NE2	GLN	B	386	0.535	3.693	25.585	1.00	0.00	N
ATOM	3732	HE21	GLN	B	386	1.031	3.221	24.858	1.00	0.00	H
ATOM	3733	HE22	GLN	B	386	-0.464	3.688	25.603	1.00	0.00	H
ATOM	3734	C	GLN	B	386	-1.237	8.490	27.917	1.00	0.00	C
ATOM	3735	O	GLN	B	386	-0.518	9.463	28.122	1.00	0.00	O
ATOM	3736	N	LYS	B	387	-2.488	8.568	27.427	1.00	0.00	N
ATOM	3737	H	LYS	B	387	-2.998	7.728	27.234	1.00	0.00	H

ATOM	3738	CA	LYS	B	387	-3.089	9.889	27.231	1.00	0.00	C
ATOM	3739	CB	LYS	B	387	-4.480	9.754	26.582	1.00	0.00	C
ATOM	3740	CG	LYS	B	387	-5.142	11.083	26.179	1.00	0.00	C
ATOM	3741	CD	LYS	B	387	-6.455	10.923	25.393	1.00	0.00	C
ATOM	3742	CE	LYS	B	387	-7.078	12.273	25.005	1.00	0.00	C
ATOM	3743	NZ	LYS	B	387	-8.225	12.102	24.104	1.00	0.00	N
ATOM	3744	HZ1	LYS	B	387	-9.105	11.764	24.551	1.00	0.00	H
ATOM	3745	HZ2	LYS	B	387	-8.537	13.027	23.739	1.00	0.00	H
ATOM	3746	HZ3	LYS	B	387	-8.015	11.532	23.262	1.00	0.00	H
ATOM	3747	C	LYS	B	387	-3.132	10.688	28.526	1.00	0.00	C
ATOM	3748	O	LYS	B	387	-2.646	11.810	28.621	1.00	0.00	O
ATOM	3749	N	LYS	B	388	-3.699	10.024	29.546	1.00	0.00	N
ATOM	3750	H	LYS	B	388	-4.031	9.090	29.402	1.00	0.00	H
ATOM	3751	CA	LYS	B	388	-3.804	10.681	30.849	1.00	0.00	C
ATOM	3752	CB	LYS	B	388	-4.641	9.811	31.793	1.00	0.00	C
ATOM	3753	CG	LYS	B	388	-6.064	9.671	31.236	1.00	0.00	C
ATOM	3754	CD	LYS	B	388	-6.900	8.542	31.843	1.00	0.00	C
ATOM	3755	CE	LYS	B	388	-8.199	8.348	31.051	1.00	0.00	C
ATOM	3756	NZ	LYS	B	388	-8.923	7.156	31.515	1.00	0.00	N
ATOM	3757	HZ1	LYS	B	388	-8.283	6.340	31.559	1.00	0.00	H
ATOM	3758	HZ2	LYS	B	388	-9.365	7.316	32.440	1.00	0.00	H
ATOM	3759	HZ3	LYS	B	388	-9.657	6.894	30.818	1.00	0.00	H
ATOM	3760	C	LYS	B	388	-2.468	11.107	31.440	1.00	0.00	C
ATOM	3761	O	LYS	B	388	-2.285	12.258	31.815	1.00	0.00	O
ATOM	3762	N	GLN	B	389	-1.513	10.152	31.430	1.00	0.00	N
ATOM	3763	H	GLN	B	389	-1.776	9.228	31.157	1.00	0.00	H
ATOM	3764	CA	GLN	B	389	-0.135	10.454	31.848	1.00	0.00	C
ATOM	3765	CB	GLN	B	389	0.865	9.375	31.408	1.00	0.00	C
ATOM	3766	CG	GLN	B	389	0.770	8.002	32.068	1.00	0.00	C
ATOM	3767	CD	GLN	B	389	1.875	7.104	31.531	1.00	0.00	C
ATOM	3768	OE1	GLN	B	389	2.417	7.271	30.439	1.00	0.00	O
ATOM	3769	NE2	GLN	B	389	2.209	6.125	32.374	1.00	0.00	N
ATOM	3770	HE21	GLN	B	389	2.940	5.481	32.152	1.00	0.00	H
ATOM	3771	HE22	GLN	B	389	1.737	6.031	33.250	1.00	0.00	H
ATOM	3772	C	GLN	B	389	0.390	11.755	31.276	1.00	0.00	C
ATOM	3773	O	GLN	B	389	0.850	12.659	31.965	1.00	0.00	O
ATOM	3774	N	GLN	B	390	0.301	11.796	29.942	1.00	0.00	N
ATOM	3775	H	GLN	B	390	-0.180	11.078	29.434	1.00	0.00	H
ATOM	3776	CA	GLN	B	390	0.929	12.931	29.280	1.00	0.00	C
ATOM	3777	CB	GLN	B	390	1.231	12.568	27.829	1.00	0.00	C
ATOM	3778	CG	GLN	B	390	2.150	11.344	27.898	1.00	0.00	C
ATOM	3779	CD	GLN	B	390	2.467	10.718	26.557	1.00	0.00	C
ATOM	3780	OE1	GLN	B	390	3.588	10.276	26.330	1.00	0.00	O
ATOM	3781	NE2	GLN	B	390	1.445	10.638	25.700	1.00	0.00	N
ATOM	3782	HE21	GLN	B	390	1.607	10.272	24.785	1.00	0.00	H
ATOM	3783	HE22	GLN	B	390	0.523	10.929	25.955	1.00	0.00	H
ATOM	3784	C	GLN	B	390	0.193	14.237	29.474	1.00	0.00	C
ATOM	3785	O	GLN	B	390	0.804	15.286	29.608	1.00	0.00	O
ATOM	3786	N	THR	B	391	-1.140	14.117	29.575	1.00	0.00	N
ATOM	3787	H	THR	B	391	-1.569	13.219	29.488	1.00	0.00	H
ATOM	3788	CA	THR	B	391	-1.913	15.306	29.942	1.00	0.00	C
ATOM	3789	CB	THR	B	391	-3.413	14.979	29.941	1.00	0.00	C
ATOM	3790	OG1	THR	B	391	-3.780	14.329	28.716	1.00	0.00	O
ATOM	3791	HG1	THR	B	391	-3.278	13.524	28.663	1.00	0.00	H
ATOM	3792	CG2	THR	B	391	-4.284	16.221	30.157	1.00	0.00	C
ATOM	3793	C	THR	B	391	-1.471	15.923	31.269	1.00	0.00	C
ATOM	3794	O	THR	B	391	-1.236	17.119	31.385	1.00	0.00	O
ATOM	3795	N	VAL	B	392	-1.300	15.028	32.260	1.00	0.00	N
ATOM	3796	H	VAL	B	392	-1.502	14.058	32.107	1.00	0.00	H

ATOM	3797	CA	VAL	B	392	-0.775	15.508	33.542	1.00	0.00	C
ATOM	3798	CB	VAL	B	392	-0.761	14.374	34.582	1.00	0.00	C
ATOM	3799	CG1	VAL	B	392	-0.229	14.835	35.944	1.00	0.00	C
ATOM	3800	CG2	VAL	B	392	-2.153	13.753	34.727	1.00	0.00	C
ATOM	3801	C	VAL	B	392	0.602	16.148	33.404	1.00	0.00	C
ATOM	3802	O	VAL	B	392	0.859	17.248	33.871	1.00	0.00	O
ATOM	3803	N	THR	B	393	1.466	15.412	32.689	1.00	0.00	N
ATOM	3804	H	THR	B	393	1.141	14.543	32.321	1.00	0.00	H
ATOM	3805	CA	THR	B	393	2.829	15.900	32.453	1.00	0.00	C
ATOM	3806	CB	THR	B	393	3.586	14.884	31.582	1.00	0.00	C
ATOM	3807	OG1	THR	B	393	3.459	13.560	32.123	1.00	0.00	O
ATOM	3808	HG1	THR	B	393	2.532	13.375	32.228	1.00	0.00	H
ATOM	3809	CG2	THR	B	393	5.067	15.234	31.405	1.00	0.00	C
ATOM	3810	C	THR	B	393	2.898	17.308	31.856	1.00	0.00	C
ATOM	3811	O	THR	B	393	3.732	18.136	32.209	1.00	0.00	O
ATOM	3812	N	LEU	B	394	1.946	17.546	30.938	1.00	0.00	N
ATOM	3813	H	LEU	B	394	1.279	16.839	30.700	1.00	0.00	H
ATOM	3814	CA	LEU	B	394	1.905	18.848	30.278	1.00	0.00	C
ATOM	3815	CB	LEU	B	394	0.812	18.888	29.208	1.00	0.00	C
ATOM	3816	CG	LEU	B	394	1.081	17.951	28.028	1.00	0.00	C
ATOM	3817	CD1	LEU	B	394	-0.127	17.856	27.096	1.00	0.00	C
ATOM	3818	CD2	LEU	B	394	2.366	18.313	27.280	1.00	0.00	C
ATOM	3819	C	LEU	B	394	1.767	20.041	31.201	1.00	0.00	C
ATOM	3820	O	LEU	B	394	2.348	21.087	30.946	1.00	0.00	O
ATOM	3821	N	LEU	B	395	0.986	19.851	32.281	1.00	0.00	N
ATOM	3822	H	LEU	B	395	0.623	18.946	32.511	1.00	0.00	H
ATOM	3823	CA	LEU	B	395	0.777	21.017	33.141	1.00	0.00	C
ATOM	3824	CB	LEU	B	395	-0.414	20.817	34.097	1.00	0.00	C
ATOM	3825	CG	LEU	B	395	-0.849	22.098	34.818	1.00	0.00	C
ATOM	3826	CD1	LEU	B	395	-1.275	23.200	33.844	1.00	0.00	C
ATOM	3827	CD2	LEU	B	395	-1.920	21.814	35.871	1.00	0.00	C
ATOM	3828	C	LEU	B	395	2.040	21.616	33.770	1.00	0.00	C
ATOM	3829	O	LEU	B	395	2.413	22.710	33.374	1.00	0.00	O
ATOM	3830	N	PRO	B	396	2.756	20.885	34.680	1.00	0.00	N
ATOM	3831	CD	PRO	B	396	2.512	19.547	35.217	1.00	0.00	C
ATOM	3832	CA	PRO	B	396	3.971	21.481	35.259	1.00	0.00	C
ATOM	3833	CB	PRO	B	396	4.561	20.346	36.101	1.00	0.00	C
ATOM	3834	CG	PRO	B	396	3.365	19.478	36.475	1.00	0.00	C
ATOM	3835	C	PRO	B	396	4.968	22.031	34.248	1.00	0.00	C
ATOM	3836	O	PRO	B	396	5.650	23.023	34.474	1.00	0.00	O
ATOM	3837	N	ALA	B	397	5.020	21.336	33.094	1.00	0.00	N
ATOM	3838	H	ALA	B	397	4.438	20.534	32.959	1.00	0.00	H
ATOM	3839	CA	ALA	B	397	5.831	21.879	32.007	1.00	0.00	C
ATOM	3840	CB	ALA	B	397	5.801	20.943	30.797	1.00	0.00	C
ATOM	3841	C	ALA	B	397	5.395	23.274	31.581	1.00	0.00	C
ATOM	3842	O	ALA	B	397	6.179	24.212	31.553	1.00	0.00	O
ATOM	3843	N	ALA	B	398	4.089	23.371	31.287	1.00	0.00	N
ATOM	3844	H	ALA	B	398	3.482	22.591	31.450	1.00	0.00	H
ATOM	3845	CA	ALA	B	398	3.521	24.659	30.898	1.00	0.00	C
ATOM	3846	CB	ALA	B	398	2.027	24.522	30.598	1.00	0.00	C
ATOM	3847	C	ALA	B	398	3.718	25.758	31.926	1.00	0.00	C
ATOM	3848	O	ALA	B	398	4.055	26.886	31.587	1.00	0.00	O
ATOM	3849	N	ASP	B	399	3.537	25.370	33.197	1.00	0.00	N
ATOM	3850	H	ASP	B	399	3.131	24.480	33.415	1.00	0.00	H
ATOM	3851	CA	ASP	B	399	3.775	26.315	34.288	1.00	0.00	C
ATOM	3852	CB	ASP	B	399	3.484	25.671	35.651	1.00	0.00	C
ATOM	3853	CG	ASP	B	399	2.058	25.157	35.739	1.00	0.00	C
ATOM	3854	OD1	ASP	B	399	1.132	25.927	35.488	1.00	0.00	O
ATOM	3855	OD2	ASP	B	399	1.881	23.985	36.069	1.00	0.00	O

ATOM	3856	C	ASP	B	399	5.182	26.884	34.290	1.00	0.00	C
ATOM	3857	O	ASP	B	399	5.407	28.089	34.307	1.00	0.00	O
ATOM	3858	N	LEU	B	400	6.145	25.949	34.242	1.00	0.00	N
ATOM	3859	H	LEU	B	400	5.883	24.982	34.185	1.00	0.00	H
ATOM	3860	CA	LEU	B	400	7.545	26.380	34.275	1.00	0.00	C
ATOM	3861	CB	LEU	B	400	8.470	25.178	34.472	1.00	0.00	C
ATOM	3862	CG	LEU	B	400	8.238	24.441	35.794	1.00	0.00	C
ATOM	3863	CD1	LEU	B	400	9.027	23.134	35.855	1.00	0.00	C
ATOM	3864	CD2	LEU	B	400	8.500	25.332	37.010	1.00	0.00	C
ATOM	3865	C	LEU	B	400	7.993	27.196	33.073	1.00	0.00	C
ATOM	3866	O	LEU	B	400	8.776	28.131	33.178	1.00	0.00	O
ATOM	3867	N	ASP	B	401	7.438	26.790	31.926	1.00	0.00	N
ATOM	3868	H	ASP	B	401	6.816	26.006	31.927	1.00	0.00	H
ATOM	3869	CA	ASP	B	401	7.727	27.469	30.662	1.00	0.00	C
ATOM	3870	CB	ASP	B	401	7.423	26.459	29.546	1.00	0.00	C
ATOM	3871	CG	ASP	B	401	8.031	26.806	28.199	1.00	0.00	C
ATOM	3872	OD1	ASP	B	401	9.104	27.403	28.150	1.00	0.00	O
ATOM	3873	OD2	ASP	B	401	7.422	26.463	27.188	1.00	0.00	O
ATOM	3874	C	ASP	B	401	6.963	28.783	30.485	1.00	0.00	C
ATOM	3875	O	ASP	B	401	7.169	29.570	29.567	1.00	0.00	O
ATOM	3876	N	ASP	B	402	6.004	28.981	31.414	1.00	0.00	N
ATOM	3877	H	ASP	B	402	5.911	28.355	32.187	1.00	0.00	H
ATOM	3878	CA	ASP	B	402	5.011	30.049	31.276	1.00	0.00	C
ATOM	3879	CB	ASP	B	402	5.573	31.423	31.664	1.00	0.00	C
ATOM	3880	CG	ASP	B	402	4.478	32.408	32.061	1.00	0.00	C
ATOM	3881	OD1	ASP	B	402	3.294	32.059	32.122	1.00	0.00	O
ATOM	3882	OD2	ASP	B	402	4.810	33.553	32.353	1.00	0.00	O
ATOM	3883	C	ASP	B	402	4.310	30.063	29.927	1.00	0.00	C
ATOM	3884	O	ASP	B	402	4.005	31.081	29.311	1.00	0.00	O
ATOM	3885	N	PHE	B	403	4.025	28.824	29.492	1.00	0.00	N
ATOM	3886	H	PHE	B	403	4.317	28.053	30.060	1.00	0.00	H
ATOM	3887	CA	PHE	B	403	3.323	28.642	28.224	1.00	0.00	C
ATOM	3888	CB	PHE	B	403	3.163	27.147	27.930	1.00	0.00	C
ATOM	3889	CG	PHE	B	403	2.633	26.913	26.533	1.00	0.00	C
ATOM	3890	CD1	PHE	B	403	3.410	27.289	25.415	1.00	0.00	C
ATOM	3891	CD2	PHE	B	403	1.364	26.316	26.374	1.00	0.00	C
ATOM	3892	CE1	PHE	B	403	2.910	27.065	24.118	1.00	0.00	C
ATOM	3893	CE2	PHE	B	403	0.864	26.089	25.078	1.00	0.00	C
ATOM	3894	CZ	PHE	B	403	1.642	26.466	23.963	1.00	0.00	C
ATOM	3895	C	PHE	B	403	1.999	29.389	28.175	1.00	0.00	C
ATOM	3896	O	PHE	B	403	1.571	29.911	27.156	1.00	0.00	O
ATOM	3897	N	SER	B	404	1.405	29.486	29.370	1.00	0.00	N
ATOM	3898	H	SER	B	404	1.720	28.936	30.144	1.00	0.00	H
ATOM	3899	CA	SER	B	404	0.273	30.381	29.578	1.00	0.00	C
ATOM	3900	CB	SER	B	404	-0.036	30.359	31.075	1.00	0.00	C
ATOM	3901	OG	SER	B	404	0.109	29.007	31.533	1.00	0.00	O
ATOM	3902	HG	SER	B	404	0.184	29.020	32.482	1.00	0.00	H
ATOM	3903	C	SER	B	404	0.451	31.800	29.038	1.00	0.00	C
ATOM	3904	O	SER	B	404	-0.342	32.301	28.245	1.00	0.00	O
ATOM	3905	N	LYS	B	405	1.541	32.447	29.485	1.00	0.00	N
ATOM	3906	H	LYS	B	405	2.261	32.009	30.035	1.00	0.00	H
ATOM	3907	CA	LYS	B	405	1.716	33.788	28.933	1.00	0.00	C
ATOM	3908	CB	LYS	B	405	2.420	34.730	29.908	1.00	0.00	C
ATOM	3909	CG	LYS	B	405	1.655	34.810	31.231	1.00	0.00	C
ATOM	3910	CD	LYS	B	405	2.298	35.747	32.255	1.00	0.00	C
ATOM	3911	CE	LYS	B	405	2.098	35.245	33.689	1.00	0.00	C
ATOM	3912	NZ	LYS	B	405	2.852	33.997	33.863	1.00	0.00	N
ATOM	3913	HZ1	LYS	B	405	3.869	34.157	33.687	1.00	0.00	H
ATOM	3914	HZ2	LYS	B	405	2.730	33.523	34.773	1.00	0.00	H

```
ATOM   3915  HZ3 LYS B 405       2.646  33.318  33.098  1.00  0.00           H
ATOM   3916  C   LYS B 405       2.330  33.836  27.549  1.00  0.00           C
ATOM   3917  O   LYS B 405       2.151  34.809  26.825  1.00  0.00           O
ATOM   3918  N   GLN B 406       2.997  32.731  27.172  1.00  0.00           N
ATOM   3919  H   GLN B 406       3.203  32.017  27.843  1.00  0.00           H
ATOM   3920  CA  GLN B 406       3.397  32.602  25.767  1.00  0.00           C
ATOM   3921  CB  GLN B 406       4.162  31.308  25.504  1.00  0.00           C
ATOM   3922  CG  GLN B 406       5.498  31.179  26.239  1.00  0.00           C
ATOM   3923  CD  GLN B 406       6.168  29.906  25.764  1.00  0.00           C
ATOM   3924  OE1 GLN B 406       6.036  29.512  24.612  1.00  0.00           O
ATOM   3925  NE2 GLN B 406       6.866  29.274  26.705  1.00  0.00           N
ATOM   3926 HE21 GLN B 406       7.298  28.377  26.551  1.00  0.00           H
ATOM   3927 HE22 GLN B 406       7.016  29.595  27.642  1.00  0.00           H
ATOM   3928  C   GLN B 406       2.217  32.698  24.813  1.00  0.00           C
ATOM   3929  O   GLN B 406       2.202  33.496  23.886  1.00  0.00           O
ATOM   3930  N   LEU B 407       1.182  31.906  25.145  1.00  0.00           N
ATOM   3931  H   LEU B 407       1.302  31.237  25.877  1.00  0.00           H
ATOM   3932  CA  LEU B 407      -0.085  32.003  24.417  1.00  0.00           C
ATOM   3933  CB  LEU B 407      -1.146  31.101  25.045  1.00  0.00           C
ATOM   3934  CG  LEU B 407      -0.897  29.608  24.840  1.00  0.00           C
ATOM   3935  CD1 LEU B 407      -1.879  28.766  25.654  1.00  0.00           C
ATOM   3936  CD2 LEU B 407      -0.908  29.225  23.359  1.00  0.00           C
ATOM   3937  C   LEU B 407      -0.627  33.416  24.331  1.00  0.00           C
ATOM   3938  O   LEU B 407      -1.089  33.887  23.300  1.00  0.00           O
ATOM   3939  N   GLN B 408      -0.518  34.103  25.475  1.00  0.00           N
ATOM   3940  H   GLN B 408      -0.161  33.646  26.291  1.00  0.00           H
ATOM   3941  CA  GLN B 408      -0.911  35.510  25.456  1.00  0.00           C
ATOM   3942  CB  GLN B 408      -0.935  36.062  26.881  1.00  0.00           C
ATOM   3943  CG  GLN B 408      -1.973  35.348  27.755  1.00  0.00           C
ATOM   3944  CD  GLN B 408      -3.376  35.765  27.349  1.00  0.00           C
ATOM   3945  OE1 GLN B 408      -3.891  36.782  27.787  1.00  0.00           O
ATOM   3946  NE2 GLN B 408      -3.994  34.935  26.502  1.00  0.00           N
ATOM   3947 HE21 GLN B 408      -4.924  35.173  26.233  1.00  0.00           H
ATOM   3948 HE22 GLN B 408      -3.595  34.104  26.108  1.00  0.00           H
ATOM   3949  C   GLN B 408      -0.116  36.392  24.503  1.00  0.00           C
ATOM   3950  O   GLN B 408      -0.661  37.280  23.864  1.00  0.00           O
ATOM   3951  N   GLN B 409       1.187  36.078  24.383  1.00  0.00           N
ATOM   3952  H   GLN B 409       1.566  35.298  24.884  1.00  0.00           H
ATOM   3953  CA  GLN B 409       1.962  36.786  23.359  1.00  0.00           C
ATOM   3954  CB  GLN B 409       3.465  36.479  23.439  1.00  0.00           C
ATOM   3955  CG  GLN B 409       4.092  36.452  24.838  1.00  0.00           C
ATOM   3956  CD  GLN B 409       3.843  37.750  25.579  1.00  0.00           C
ATOM   3957  OE1 GLN B 409       4.343  38.812  25.241  1.00  0.00           O
ATOM   3958  NE2 GLN B 409       3.040  37.611  26.636  1.00  0.00           N
ATOM   3959 HE21 GLN B 409       2.831  38.418  27.182  1.00  0.00           H
ATOM   3960 HE22 GLN B 409       2.662  36.709  26.852  1.00  0.00           H
ATOM   3961  C   GLN B 409       1.467  36.508  21.948  1.00  0.00           C
ATOM   3962  O   GLN B 409       1.390  37.380  21.093  1.00  0.00           O
ATOM   3963  N   SER B 410       1.076  35.243  21.752  1.00  0.00           N
ATOM   3964  H   SER B 410       1.184  34.519  22.438  1.00  0.00           H
ATOM   3965  CA  SER B 410       0.440  34.921  20.481  1.00  0.00           C
ATOM   3966  CB  SER B 410       0.781  33.475  20.112  1.00  0.00           C
ATOM   3967  OG  SER B 410       0.650  32.629  21.263  1.00  0.00           O
ATOM   3968  HG  SER B 410      -0.187  32.176  21.174  1.00  0.00           H
ATOM   3969  C   SER B 410      -1.063  35.169  20.425  1.00  0.00           C
ATOM   3970  O   SER B 410      -1.763  34.556  19.630  1.00  0.00           O
ATOM   3971  N   MET B 411      -1.546  36.068  21.311  1.00  0.00           N
ATOM   3972  H   MET B 411      -0.918  36.553  21.920  1.00  0.00           H
ATOM   3973  CA  MET B 411      -2.973  36.419  21.348  1.00  0.00           C
```

ATOM	3974	CB	MET	B	411	-3.316	37.415	20.234	1.00	0.00	C
ATOM	3975	CG	MET	B	411	-2.484	38.699	20.287	1.00	0.00	C
ATOM	3976	SD	MET	B	411	-2.769	39.673	21.776	1.00	0.00	S
ATOM	3977	CE	MET	B	411	-4.483	40.126	21.464	1.00	0.00	C
ATOM	3978	C	MET	B	411	-3.935	35.238	21.342	1.00	0.00	C
ATOM	3979	O	MET	B	411	-4.908	35.178	20.603	1.00	0.00	O
ATOM	3980	N	SER	B	412	-3.586	34.278	22.202	1.00	0.00	N
ATOM	3981	H	SER	B	412	-2.855	34.429	22.866	1.00	0.00	H
ATOM	3982	CA	SER	B	412	-4.245	32.984	22.103	1.00	0.00	C
ATOM	3983	CB	SER	B	412	-3.420	32.062	21.191	1.00	0.00	C
ATOM	3984	OG	SER	B	412	-2.036	32.075	21.572	1.00	0.00	O
ATOM	3985	HG	SER	B	412	-1.849	32.973	21.841	1.00	0.00	H
ATOM	3986	C	SER	B	412	-4.506	32.385	23.471	1.00	0.00	C
ATOM	3987	O	SER	B	412	-4.052	32.891	24.495	1.00	0.00	O
ATOM	3988	N	SER	B	413	-5.280	31.296	23.440	1.00	0.00	N
ATOM	3989	H	SER	B	413	-5.600	30.929	22.568	1.00	0.00	H
ATOM	3990	CA	SER	B	413	-5.814	30.729	24.671	1.00	0.00	C
ATOM	3991	CB	SER	B	413	-7.040	31.546	25.077	1.00	0.00	C
ATOM	3992	OG	SER	B	413	-8.034	31.465	24.040	1.00	0.00	O
ATOM	3993	HG	SER	B	413	-8.859	31.644	24.509	1.00	0.00	H
ATOM	3994	C	SER	B	413	-6.212	29.286	24.429	1.00	0.00	C
ATOM	3995	O	SER	B	413	-6.125	28.797	23.309	1.00	0.00	O
ATOM	3996	N	ALA	B	414	-6.726	28.643	25.494	1.00	0.00	N
ATOM	3997	H	ALA	B	414	-6.792	29.092	26.384	1.00	0.00	H
ATOM	3998	CA	ALA	B	414	-7.373	27.351	25.259	1.00	0.00	C
ATOM	3999	CB	ALA	B	414	-7.739	26.679	26.583	1.00	0.00	C
ATOM	4000	C	ALA	B	414	-8.628	27.453	24.402	1.00	0.00	C
ATOM	4001	O	ALA	B	414	-8.977	26.560	23.641	1.00	0.00	O
ATOM	4002	N	ASP	B	415	-9.286	28.616	24.520	1.00	0.00	N
ATOM	4003	H	ASP	B	415	-9.075	29.283	25.239	1.00	0.00	H
ATOM	4004	CA	ASP	B	415	-10.458	28.906	23.684	1.00	0.00	C
ATOM	4005	CB	ASP	B	415	-11.111	30.241	24.047	1.00	0.00	C
ATOM	4006	CG	ASP	B	415	-11.041	30.445	25.538	1.00	0.00	C
ATOM	4007	OD1	ASP	B	415	-10.052	31.022	25.989	1.00	0.00	O
ATOM	4008	OD2	ASP	B	415	-11.933	29.974	26.232	1.00	0.00	O
ATOM	4009	C	ASP	B	415	-10.133	28.933	22.209	1.00	0.00	C
ATOM	4010	O	ASP	B	415	-10.929	28.547	21.351	1.00	0.00	O
ATOM	4011	N	SER	B	416	-8.885	29.355	21.969	1.00	0.00	N
ATOM	4012	H	SER	B	416	-8.329	29.660	22.745	1.00	0.00	H
ATOM	4013	CA	SER	B	416	-8.273	29.201	20.654	1.00	0.00	C
ATOM	4014	CB	SER	B	416	-7.071	30.142	20.560	1.00	0.00	C
ATOM	4015	OG	SER	B	416	-7.367	31.419	21.155	1.00	0.00	O
ATOM	4016	HG	SER	B	416	-7.705	31.299	22.042	1.00	0.00	H
ATOM	4017	C	SER	B	416	-7.844	27.759	20.382	1.00	0.00	C
ATOM	4018	O	SER	B	416	-6.678	27.447	20.174	1.00	0.00	O
ATOM	4019	N	THR	B	417	-8.851	26.869	20.401	1.00	0.00	N
ATOM	4020	H	THR	B	417	-9.795	27.186	20.475	1.00	0.00	H
ATOM	4021	CA	THR	B	417	-8.597	25.435	20.564	1.00	0.00	C
ATOM	4022	CB	THR	B	417	-9.890	24.764	21.059	1.00	0.00	C
ATOM	4023	OG1	THR	B	417	-10.711	25.725	21.755	1.00	0.00	O
ATOM	4024	HG1	THR	B	417	-10.183	25.908	22.537	1.00	0.00	H
ATOM	4025	CG2	THR	B	417	-9.614	23.570	21.980	1.00	0.00	C
ATOM	4026	C	THR	B	417	-7.969	24.667	19.399	1.00	0.00	C
ATOM	4027	O	THR	B	417	-8.047	23.450	19.309	1.00	0.00	O
ATOM	4028	N	GLN	B	418	-7.318	25.426	18.505	1.00	0.00	N
ATOM	4029	H	GLN	B	418	-7.252	26.416	18.613	1.00	0.00	H
ATOM	4030	CA	GLN	B	418	-6.425	24.731	17.587	1.00	0.00	C
ATOM	4031	CB	GLN	B	418	-6.261	25.528	16.290	1.00	0.00	C
ATOM	4032	CG	GLN	B	418	-7.580	26.005	15.661	1.00	0.00	C

```
ATOM   4033  CD  GLN B 418      -8.463  24.828  15.283  1.00  0.00           C
ATOM   4034  OE1 GLN B 418      -9.510  24.578  15.870  1.00  0.00           O
ATOM   4035  NE2 GLN B 418      -8.003  24.120  14.249  1.00  0.00           N
ATOM   4036 HE21 GLN B 418      -8.551  23.367  13.883  1.00  0.00           H
ATOM   4037 HE22 GLN B 418      -7.126  24.321  13.813  1.00  0.00           H
ATOM   4038  C   GLN B 418      -5.072  24.447  18.228  1.00  0.00           C
ATOM   4039  O   GLN B 418      -4.349  23.536  17.853  1.00  0.00           O
ATOM   4040  N   ALA B 419      -4.759  25.276  19.244  1.00  0.00           N
ATOM   4041  H   ALA B 419      -5.377  25.999  19.554  1.00  0.00           H
ATOM   4042  CA  ALA B 419      -3.508  25.053  19.970  1.00  0.00           C
ATOM   4043  CB  ALA B 419      -3.289  26.169  20.997  1.00  0.00           C
ATOM   4044  C   ALA B 419      -3.450  23.712  20.684  1.00  0.00           C
ATOM   4045  O   ALA B 419      -4.031  23.528  21.748  1.00  0.00           O
END
```
